# Supplementary material for: Glycosyl ortho-(1-phenylvinyl)benzoates versatile glycosyl donors for highly efficient synthesis of both O-glycosides and nucleosides
Source: Nat Commun. 2020 Jan 21;11:405. doi: 10.1038/s41467-020-14295-z (PMC6972911; doi:10.1038/s41467-020-14295-z)
Supplement: Supplementary file 1 — Supplementary Information [file 41467_2020_14295_MOESM1_ESM.pdf]

Supplementary Information

Glycosyl *ortho*-(1-phenylvinyl)benzoates versatile glycosyl donors for highly efficient synthesis of both *O*-glycosides and nucleosides

Li et al.

Table of Contents

Supplementary Methods--- 2

General information --- 2

Preparation of the glycosyl PVB donors-- 2

Glycosylation reactions with the glycosyl PVB as donors ---17

The acceptors **2a-n** and **5a-i** ---17

General procedures for glycosylation with glycosyl PVB---17

Comparison of the donor reactivity---55

One-pot synthesis of oligosaccharides **13-16** ---57

Synthesis of Capecitabine **17**, Doxifluridine **18** and Galocitabine **19**--------------------------------64

X-ray crystal structure data of compound **4**--67

Supplementary Figures ---69

NMR spectra of new compounds ---70

Supplementary References--- 190

Supplementary Methods

General information. Commercial reagents, starting materials, and solvents were purchased and used as received or distilled from the appropriate drying agent. Reactions requiring anhydrous conditions were performed under an atmosphere of nitrogen or argon with dry solvents unless otherwise stated. Crushed 3Å or 4Å molecular sieves were activated by flame-drying immediately under high vacuum prior to use. Reactions were monitored by thin layer chromatography (TLC) carried out on TLC Silica Gel 60 F_{254} (EMD Millipore Corporation) using UV light as visualizing tool and $EtOH/H_2SO_4$ (8%, v/v) as the developing agent. Flash column chromatography was performed on Silica Gel 60 (200-300 mesh). NMR spectra were measured on Bruker AVANCE III 400 or 600 MHz NMR spectrometer by using $CDCl_3$ as internal references: the solvent ($CDCl_3$: δ_H = 7.26 ppm; δ_C = 77.16 ppm). Multiplicities for ^{1}H NMR signals are described using the following abbreviations: s = singlet, d = doublet, t = triplet, q = quartet, m = multiplet. High resolution mass spectra and elemental analysis was determined by an Agilent 1290 UPLC/6540 Q-TOF spectrometer. Optical rotations were measured on an Autopol VI (Serial #91058), using $CHCl_3$ as solvent. Single crystal X-ray data were collected on a Bruker APEX DUO diffractometer using Cu Kα radiation (λ = 1.54178 Å) at 100 K.

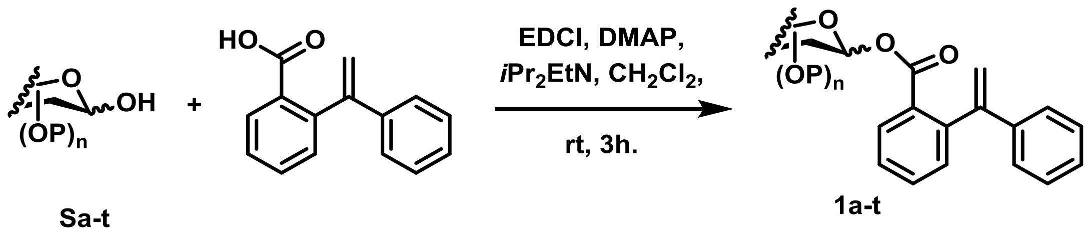

Supplementary Figure 1. Preparation of the glycosyl PVB donors **1a-t**.

General procedure A. To a solution of **Compound S** (1.0 eq.) and 2-(1-Phenylvinyl) benzoic acid[1] (1.1 eq.) in dry DCM (0.1 M) was added 4-dimethylaminopyridine (DMAP) (1.0 eq.), N-(3-dimethylaminopropyl)-N'-ethylcarbodiimide hydrochloride (EDCI) (1.8 eq.) and N,N-diisopropylethylamine (DIPEA) (3.0 eq.). The resulting

mixture was stirred at room temperature until TLC-analysis indicated the reaction to be complete. Then, the mixture was diluted with CH_2Cl_2, and washed with water and brine. The organic layer was dried over Na_2SO_4, filtered, and concentrated in vacuo. The residue was purified by flash column chromatography on silica gel to afford the glycosyl PVB donors.

2,3,4,6-tetra-*O*-benzoyl-D-glucopyranosyl 2-(1-phenylvinyl) benzoate (1a)

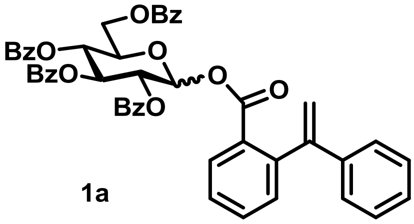

1a

Compound **1a** was prepared from **Sa**[2] (500 mg, 0.84 mmol) according to **General procedure A**. The crude product was purified by silica gel column chromatography (petroleum ether/EtOAc = 3:1) to afford **1a** (627 mg, 93%, α/β = 1:1.2) as a white solid. The α anomer: $[\alpha]_D^{25}$ = -2.4 (*c* 0.20, $CHCl_3$); ^1H NMR (400 MHz, $CDCl_3$): δ 8.02 (d, *J* = 7.7 Hz, 2H), 7.91–7.82 (m, 7H), 7.59–7.20 (m, 20H), 6.74 (d, *J* = 3.8 Hz, 1H), 6.04 (s, 1H), 5.97 (t, *J* = 10.0 Hz, 1H), 5.65 (t, *J* = 10.0 Hz, 1H), 5.52 (dd, *J* = 10.3, 3.9 Hz, 1H), 5.25 (s, 1H), 4.29 (dd, *J* = 12.4, 3.0 Hz, 1H), 4.11 (dd, *J* = 12.5, 3.8 Hz, 1H), 3.73 (dt, *J* = 10.2, 3.4 Hz, 1H); ^{13}C NMR (101 MHz, $CDCl_3$) δ 166.16, 166.04, 165.77, 165.36, 165.01, 148.01, 142.81, 139.81, 133.49, 133.27, 133.15, 132.19, 131.03, 130.71, 130.21, 129.90, 129.85, 129.80, 129.69, 129.58, 128.93, 128.77, 128.71, 128.60, 128.43, 128.40, 128.35, 128.02, 127.71, 127.02, 114.94, 90.22, 70.61, 70.40, 70.35, 68.39, 62.03; HRMS (ESI) calcd for $C_{49}H_{38}O_{11}Na$ $[M+Na]^+$ 825.2306, found 825.2317.

2,3,4,6-tetra-*O*-acetyl-D-glucopyranosyl 2-(1-phenylvinyl) benzoate (1b)

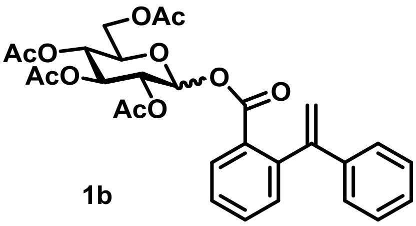

1b

Compound **1b** was prepared from **Sb**[3] (100 mg, 0.29 mmol) according to **General**

procedure A. The crude product was purified by silica gel column chromatography (petroleum ether/EtOAc = 3:1) to afford **1b** (155 mg, 97%, α/β = 1:2.4) as a colorless syrup. The α anomer: $[\alpha]_D^{25}$ = 103.6 (*c* 0.13, $CHCl_3$); ^{1}H NMR (400 MHz, $CDCl_3$): δ 7.93 (d, *J* = 7.9 Hz, 1H), 7.57 (td, *J* = 7.5, 1.4 Hz, 1H), 7.48 (td, *J* = 7.6, 1.4 Hz, 1H), 7.37 (d, *J* = 7.8 Hz, 2H), 7.34–7.24 (m, 4H), 6.41 (d, *J* = 3.7 Hz, 1H), 5.94 (s, 1H), 5.28 (t, *J* = 9.8 Hz, 1H), 5.20 (s, 1H), 5.05 (dd, *J* = 10.2, 3.7 Hz, 1H), 4.99 (t, *J* = 9.9 Hz, 1H), 3.96 (dd, *J* = 12.6, 3.6 Hz, 1H), 3.74 (dd, *J* = 12.5, 2.2 Hz, 1H), 3.41 (dt, *J* = 10.3, 2.9 Hz, 1H), 2.05 (s, 3H), 2.00 (s, 3H), 1.98 (s, 3H), 1.92 (s, 3H); ^{13}C NMR (101 MHz, $CDCl_3$) δ 170.60, 170.06, 169.72, 169.27, 165.94, 147.92, 142.90, 139.57, 132.27, 131.13, 130.60, 130.00, 128.44, 127.84, 127.72, 126.88, 114.59, 89.92, 70.01, 69.78, 69.39, 67.34, 61.00, 20.68, 20.56, 20.37; HRMS (ESI) calcd for $C_{29}H_{30}O_{11}Na$ $[M+Na]^+$ 577.1680, found 577.1690.

2,3,4,6-tetra-*O*-benzoyl -D-galactopyranosyl 2-(1-phenylvinyl) benzoate (1c)

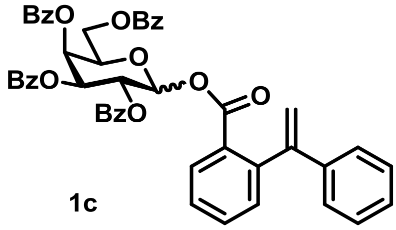

1c

Compound **1c** was prepared from **Sc**[4] (100 mg, 0.17 mmol) according to **General procedure A**. The crude product was purified by silica gel column chromatography (petroleum ether/EtOAc = 3:1) to afford **1c** (123 mg, 91%, α/β = 1:1.3) as a white solid. The α anomer: $[\alpha]_D^{25}$ = 155.6 (*c* 0.17, $CHCl_3$); ^{1}H NMR (400 MHz, $CDCl_3$): δ 7.95 (d, *J* = 7.5 Hz, 2H), 7.89 (d, *J* = 7.4 Hz, 2H), 7.83 (d, *J* = 7.9 Hz, 1H), 7.78 (d, *J* = 7.5 Hz, 2H), 7.71 (d, *J* = 7.6 Hz, 2H), 7.52 (t, *J* = 8.0 Hz, 1H), 7.48 (t, *J* = 8.0 Hz, 2H), 7.43–7.32 (m, 9H), 7.28–7.14 (m, 7H), 7.01 (t, *J* = 7.4 Hz, 1H), 6.74 (d, *J* = 2.2 Hz, 1H), 5.99 (s, 1H), 5.74 (d, *J* = 2.1 Hz, 2H), 5.62 (s, 1H), 5.15 (s, 1H), 4.25 (dd, *J* = 11.2, 6.3 Hz, 1H), 3.99 (dd, *J* = 11.3, 7.1 Hz, 1H), 3.57 (t, *J* = 6.7 Hz, 1H); ^{13}C NMR (151 MHz, $CDCl_3$) δ 166.10, 165.59, 165.49, 165.35, 147.91, 142.64, 139.19, 133.57, 133.38, 133.22, 133.19, 132.29, 131.12, 130.85, 130.01, 129.84, 129.72, 129.70, 129.68, 129.39, 128.94, 128.92, 128.78, 128.61, 128.56, 128.39, 128.36,

128.26, 128.13, 127.65, 126.61, 114.31, 90.80, 69.11, 68.40, 68.16, 67.83, 61.31; HRMS (ESI) calcd for $C_{49}H_{38}O_{11}Na$ [M+Na]$^+$ 825.2306, found 825.2305.

2,3,4,6-tetra-*O*-benzoyl-D-mannopyranosyl 2-(1-phenylvinyl) benzoate (1d)

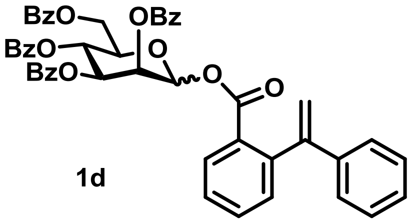

1d

Compound **1d** was prepared from **Sd**[5] (100 mg, 0.17 mmol) according to **General procedure A**. The crude product was purified by silica gel column chromatography (petroleum ether/EtOAc = 3:1) to afford **1d** (131 mg, 97%, α/β = 1:11.4) as a white solid. The β anomer: $[\alpha]_D^{25}$ = -8.2 (*c* 0.17, $CHCl_3$); ^{1}H NMR (400 MHz, $CDCl_3$): δ 8.07 (d, *J* = 7.8 Hz, 2H), 8.03 (d, *J* = 7.8 Hz, 2H), 8.00 (d, *J* = 7.7 Hz, 1H), 7.86 (d, *J* = 7.8 Hz, 2H), 7.82 (d, *J* = 7.7 Hz, 2H), 7.61–7.22 (m, 20H), 6.38 (s, 1H), 6.07 (t, *J* = 8.2 Hz, 1H), 6.04 (s, 1H), 5.68 (t, *J* = 3.6 Hz, 1H), 5.66 (s, 1H), 5.37 (s, 1H), 4.43 (dd, *J* = 12.4, 2.6 Hz, 1H), 4.22 (dd, *J* = 12.4, 3.5 Hz, 1H), 3.89 (dt, *J* = 10.3, 3.2 Hz, 1H); ^{13}C NMR (101 MHz, $CDCl_3$) δ 166.03, 165.68, 165.39, 165.21, 164.99, 148.47, 142.77, 139.86, 133.61, 133.52, 133.29, 133.09, 132.46, 131.20, 130.70, 130.05, 129.91, 129.84, 129.78, 129.74, 129.08, 128.96, 128.88, 128.67, 128.64, 128.45, 128.41, 128.37, 128.12, 127.91, 126.97, 115.06, 91.67, 71.04, 69.90, 69.27, 66.00, 62.16; HRMS (ESI) calcd for $C_{49}H_{38}O_{11}Na$ [M+Na]$^+$ 825.2306, found 825.2322.

2,3,4-tri-*O*-benzoyl-L-rhamnopyranosyl 2-(1-phenylvinyl) benzoate (1e)

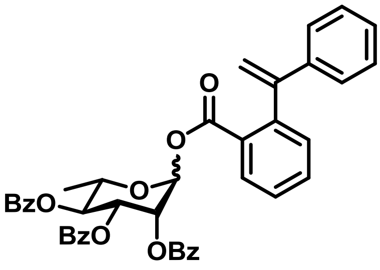

1e

Compound **1e** was prepared from **Se**[6] (100 mg, 0.21 mmol) according to **General procedure A**. The crude product was purified by silica gel column chromatography

(petroleum ether/EtOAc = 3:1) to afford **1e** (140 mg, 98%, α/β = 1:3.5) as a white solid. The β anomer: $[\alpha]_D^{25}$ = 52.8 (*c* 0.16, $CHCl_3$); ^1H NMR (400 MHz, $CDCl_3$): δ 8.06 (d, *J* = 7.3 Hz, 2H), 8.00 (d, *J* = 7.5 Hz, 1H), 7.89 (d, *J* = 7.5 Hz, 2H), 7.80 (d, *J* = 7.6 Hz, 2H), 7.63–7.57 (m, 2H), 7.54–7.38 (m, 10H), 7.36 (d, *J* = 5.2 Hz, 1H), 7.33 (d, *J* = 7.7 Hz, 1H), 7.27–7.23 (m, 3H), 6.27 (s, 1H), 6.02 (s, 1H), 5.61–5.55 (m, 3H), 5.36 (s, 1H), 3.84–3.71 (m, 1H), 1.17 (d, *J* = 6.2 Hz, 3H); ^{13}C NMR (101 MHz, $CDCl_3$) δ 165.70, 165.59, 165.37, 165.17, 148.61, 142.87, 139.93, 133.57, 133.40, 133.17, 132.33, 131.23, 130.61, 130.18, 129.96, 129.78, 129.68, 129.15, 129.09, 128.61, 128.54, 128.39, 128.32, 127.98, 127.83, 126.92, 114.84, 91.63, 71.03, 69.79, 69.56, 69.19, 17.47; HRMS (ESI) calcd for $C_{42}H_{34}O_9Na$ $[M+Na]^+$ 705.2095, found 705.2112.

2,3,4-tri-*O*-acetyl-L-rhamnopyranosyl 2-(1-phenylvinyl) benzoate (1f)

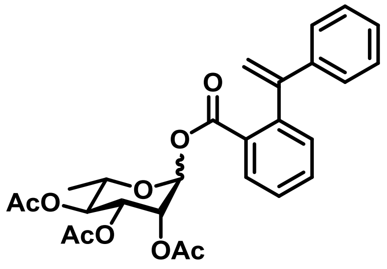

1f

Compound **1f** was prepared from **Sf**[7] (100 mg, 0.35 mmol) according to **General procedure A**. The crude product was purified by silica gel column chromatography (petroleum ether/EtOAc = 3:1) to afford **1f** (155 mg, 90%, α/β = 1:2.4) as a white solid. The β anomer: $[\alpha]_D^{25}$ = -80.9 (*c* 0.09, $CHCl_3$); ^1H NMR (400 MHz, $CDCl_3$): δ 7.91 (d, *J* = 7.7 Hz, 1H), 7.57 (t, *J* = 7.5 Hz, 1H), 7.46 (t, *J* = 7.6 Hz, 1H), 7.37–7.23 (m, 6H), 5.95 (s, 1H), 5.91 (s, 1H), 5.27 (s, 1H), 5.16–5.11 (m, 1H), 5.07 (dd, *J* = 10.1, 3.4 Hz, 1H), 4.97 (t, *J* = 9.9 Hz, 1H), 3.50–3.42 (m, 1H), 2.13 (s, 3H), 2.01 (s, 3H), 1.99 (s, 3H), 1.02 (d, *J* = 6.2 Hz, 3H); ^{13}C NMR (101 MHz, $CDCl_3$) δ 169.99, 169.91, 169.82, 165.54, 148.57, 142.97, 139.84, 132.49, 131.40, 130.61, 130.06, 128.55, 127.93, 127.88, 126.80, 114.73, 91.50, 70.40, 69.02, 68.78, 68.60, 53.56, 20.88, 20.81, 17.33; HRMS (ESI) calcd for $C_{27}H_{28}O_9Na$ $[M+Na]^+$ 519.1626, found 519.1621.

2,3,4-tri-*O*-benzoyl-D-xylopyranosyl 2-(1-phenylvinyl) benzoate (1g)

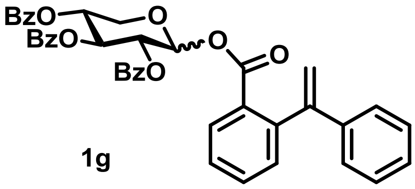

1g

Compound **1g** was prepared from **Sg**[2] (75 mg, 0.16 mmol) according to **General procedure A**. The crude product was purified by silica gel column chromatography (petroleum ether/EtOAc = 5:1) to afford **1g** (108 mg, 99%, α/β = 1:2) as a white solid. α and β anomers: ^{1}H NMR (400 MHz, $CDCl_3$): δ 7.87 (d, *J* = 7.8 Hz, 7H), 7.83 (dd, *J* = 8.0, 3.4 Hz, 6H), 7.78 (dd, *J* = 7.8, 3.6 Hz, 5H), 7.50–7.02 (m, 54H), 6.53 (d, *J* = 3.7 Hz, 1H), 5.99 (d, *J* = 3.9 Hz, 1H), 5.89 (t, *J* = 9.8 Hz, 1H), 5.87 (s, 1H), 5.61 (t, *J* = 5.6 Hz, 2H), 5.57 (s, 2H), 5.36 (d, *J* = 4.0 Hz, 1H), 5.23–5.19 (m, 3H), 5.14–5.12 (m, 2H), 5.07 (s, 2H), 4.21 (dd, *J* = 12.8, 3.5 Hz, 2H), 3.85 (dd, *J* = 11.2, 5.7 Hz, 1H), 3.74 (dd, *J* = 12.8, 4.8 Hz, 2H), 3.25 (t, *J* = 10.9 Hz, 1H); ^{13}C NMR (101 MHz, $CDCl_3$) δ 166.09, 165.77, 165.52, 165.43, 165.41, 164.93, 164.86, 164.85, 148.94, 148.25, 143.51, 143.04, 140.40, 139.86, 133.52, 133.49, 133.45, 133.33, 132.30, 132.18, 131.56, 131.20, 130.59, 130.15, 130.11, 130.02, 129.95, 129.93, 129.84, 129.78, 129.71, 129.68, 129.14, 129.12, 129.07, 128.95, 128.93, 128.91, 128.78, 128.75, 128.43, 128.26, 128.21, 127.86, 127.76, 127.70, 126.97, 126.75, 114.79, 114.65, 91.99, 90.40, 70.48, 69.88, 69.46, 68.28, 68.12, 67.84, 61.48, 61.01; HRMS (ESI) calcd for $C_{41}H_{32}O_9Na$ $[M+Na]^+$ 691.1939, found 691.1938.

2,3,4-tri-*O*-acetyl-D-xylopyranosyl 2-(1-phenylvinyl) benzoate 1h

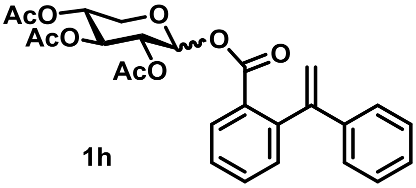

1h

Compound **1h** was prepared from **Sh**[8] (90 mg, 0.22 mmol) according to **General procedure A**. The crude product was purified by silica gel column chromatography (petroleum ether/EtOAc = 3:1) to afford **1h** (154 mg, 98%, α/β = 1:2.1) as a colorless

syrup. The β anomer: $[\alpha]_D^{25}$ = -104.0 (*c* 0.10, $CHCl_3$); ^{1}H NMR (400 MHz, $CDCl_3$): δ 7.91 (d, *J* = 7.7 Hz, 1H), 7.56 (t, *J* = 7.2 Hz, 1H), 7.43 (t, *J* = 7.5 Hz, 1H), 7.34 (d, *J* = 7.5 Hz, 1H), 7.31–7.20 (m, 5H), 5.73 (s, 1H), 5.72 (d, *J* = 5.9 Hz, 1H), 5.18 (s, 1H), 5.16 (t, *J* = 8.0 Hz, 1H), 5.05–5.00 (m, 1H), 4.91 (td, *J* = 7.7, 5.0 Hz, 1H), 4.00 (dd, *J* = 12.2, 4.8 Hz, 1H), 3.45 (dd, *J* = 12.2, 7.7 Hz, 1H), 2.05 (s, 3H), 2.03 (s, 3H), 1.95 (s, 3H); ^{13}C NMR (101 MHz, $CDCl_3$) δ 169.85, 169.73, 169.20, 164.57, 148.96, 143.84, 140.35, 132.57, 131.63, 130.38, 128.84, 128.21, 127.71, 127.63, 126.57, 114.26, 92.42, 70.70, 69.23, 68.36, 62.65, 20.75, 20.69, 20.54; HRMS (ESI) calcd for $C_{26}H_{26}O_9Na$ $[M+Na]^+$ 505.1469, found 505.1473.

2,3,4-tri-*O*-benzoyl-L-arabinopyranosyl 2-(1-phenylvinyl) benzoate (1i)

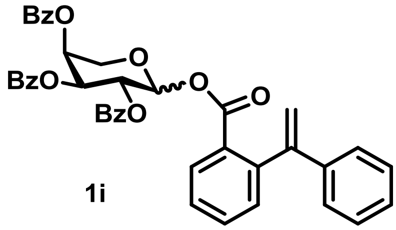

1i

Compound **1i** was prepared from **Si**[9] (70 mg, 0.15 mmol) according to **General procedure A**. The crude product was purified by silica gel column chromatography (petroleum ether/EtOAc = 5:1) to afford **1i** (97 mg, 96%, α/β = 1:4.6) as a white solid. The α anomer: $[\alpha]_D^{25}$ = 380.8 (*c* 0.11, $CHCl_3$); ^{1}H NMR (400 MHz, $CDCl_3$): δ 8.06 (d, *J* = 7.7 Hz, 2H), 7.92 (d, *J* = 7.8 Hz, 1H), 7.85 (t, *J* = 8.2 Hz, 3H), 7.63–7.55 (m, 2H), 7.53–7.41 (m, 7H), 7.38–7.24 (m, 9H), 6.68 (d, *J* = 3.5 Hz, 1H), 6.00 (s, 1H), 5.85 (dd, *J* = 10.7, 3.6 Hz, 1H), 5.77 (dd, *J* = 10.7, 3.4 Hz, 1H), 5.46 (d, *J* = 3.3 Hz, 1H), 5.24 (s, 1H), 3.73 (d, *J* = 12.6 Hz, 1H), 3.43 (d, *J* = 13.3 Hz, 1H); ^{13}C NMR (151 MHz, $CDCl_3$) δ 166.02, 165.66, 165.55, 165.47, 148.18, 142.82, 139.49, 133.46, 133.36, 133.26, 132.28, 131.29, 130.71, 130.01, 129.82, 129.72, 129.68, 129.35, 129.02, 128.89, 128.55, 128.50, 128.37, 128.32, 128.04, 127.66, 126.71, 114.24, 91.25, 69.51, 67.89, 62.77; HRMS (ESI) calcd for $C_{41}H_{32}O_9Na$ $[M+Na]^+$ 691.1939, found 691.1951.

2,3,4-tri-*O*-benzoyl-L-fucopyranosyl 2-(1-phenylvinyl) benzoate (1j)

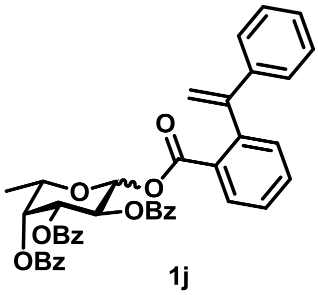

1j

Compound **1j** was prepared from **Sj**[10] (100 mg, 0.20 mmol) according to **General procedure A**. The crude product was purified by silica gel column chromatography (petroleum ether/EtOAc = 5:1) to afford **1j** (128 mg, 91%, α/β = 1:3.2) as a white solid. The α anomer: $[\alpha]_D^{25}$ = -271.3 (*c* 0.12, $CHCl_3$); ^{1}H NMR (400 MHz, $CDCl_3$): δ 7.98 (d, *J* = 7.3 Hz, 2H), 7.84 (dd, *J* = 7.9, 1.4 Hz, 1H), 7.78 (d, *J* = 7.4 Hz, 2H), 7.71 (d, *J* = 7.4 Hz, 2H), 7.55–7.47 (m, 2H), 7.44–7.33 (m, 7H), 7.31–7.23 (m, 5H), 7.21–7.15 (m, 3H), 6.63 (d, *J* = 1.9 Hz, 1H), 5.99 (s, 1H), 5.73–5.67 (m, 2H), 5.34–5.28 (m, 1H), 5.16 (s, 1H), 3.38 (q, *J* = 6.5 Hz, 1H), 0.87 (d, *J* = 6.4 Hz, 3H); ^{13}C NMR (101 MHz, $CDCl_3$) δ 165.13, 164.83, 164.56, 164.48, 147.03, 141.65, 138.36, 132.45, 132.29, 132.14, 131.22, 130.19, 129.82, 129.18, 128.84, 128.68, 128.64, 128.18, 128.08, 127.94, 127.57, 127.49, 127.33, 127.25, 127.04, 126.64, 125.66, 113.09, 90.03, 70.34, 67.66, 66.78, 66.58, 15.01; HRMS (ESI) calcd for $C_{42}H_{34}O_9Na$ $[M+Na]^+$ 705.2095, found 705.2105.

2,3,5-tri-*O*-benzoyl-L-arabinofuranosyl 2-(1-phenylvinyl) benzoate (1k)

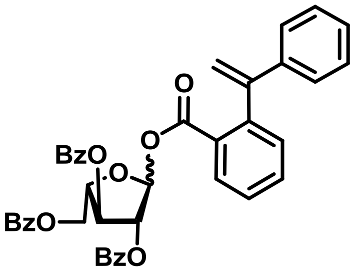

1k

Compound **1k** was prepared from **Sk**[11] (431 mg, 0.93 mmol) according to **General procedure A**. The crude product was purified by silica gel column chromatography (petroleum ether/EtOAc = 5:1) to afford **1k** (560 mg, 91%, α/β = 2.2:1) as a white foam. The α anomer: $[\alpha]_D^{25}$ = 2.5 (*c* 0.16, $CHCl_3$); ^{1}H NMR (400 MHz, $CDCl_3$): δ 8.00

(d, J = 7.8 Hz, 6H), 7.64–7.14 (m, 18H), 6.36 (s, 1H), 5.75 (s, 1H), 5.53 (d, J = 7.1 Hz, 2H), 5.25 (s, 1H), 4.67 (dd, J = 12.0, 4.0 Hz, 1H), 4.59 (dd, J = 12.0, 5.0 Hz, 1H), 4.43–4.38 (m, 1H); ^{13}C NMR (101 MHz, $CDCl_3$) δ 166.12, 165.64, 165.45, 164.99, 148.85, 143.15, 140.26, 133.70, 133.68, 133.10, 132.21, 131.44, 130.20, 130.11, 129.95, 129.75, 129.56, 128.99, 128.73, 128.61, 128.54, 128.50, 128.30, 128.23, 127.73, 127.63, 126.70, 114.60, 100.01, 83.10, 80.99, 77.42, 63.45; HRMS (ESI) calcd for $C_{41}H_{32}O_9Na$ $[M+Na]^+$ 691.1939, found 691.1938.

2,3,5-tri-*O*-benzoyl-D-ribofuranosyl 2-(1-phenylvinyl) benzoate (1l)

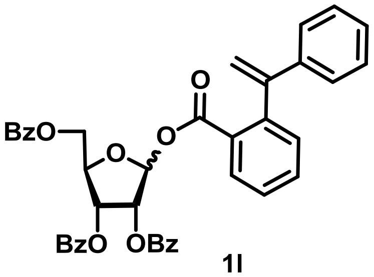

1l

Compound **1l** was prepared from **Sl**[11] (402 mg, 0.87 mmol) according to **General procedure A**. The crude product was purified by silica gel column chromatography (petroleum ether/EtOAc = 5:1) to afford **1l** (560 mg, 96%, α/β = 1:10) as a syrup. The β anomer: $[\alpha]_D^{25}$ = 26.7 (c 0.14, CHCl3); ^{1}H NMR (400 MHz, $CDCl_3$) δ 8.00 – 7.81 (m, 6H), 7.64–7.17 (m, 18H), 6.30 (s, 1H), 5.89 (s, 1H), 5.62 (dd, J = 7.3, 4.8 Hz, 1H), 5.52 (d, J = 4.8 Hz, 1H), 5.24 (s, 1H), 4.71–4.63 (m, 1H), 4.47–4.30 (m, 2H); ^{13}C NMR (101 MHz, $CDCl_3$) δ 166.02, 165.77, 165.12, 164.81, 148.86, 142.99, 139.88, 133.61, 133.47, 133.08, 132.37, 131.45, 130.46, 129.84, 129.74, 129.67, 129.47, 128.92, 128.74, 128.53, 128.46, 128.39, 128.26, 127.94, 127.80, 126.59, 114.35, 99.09, 79.64, 74.73, 71.54, 64.23; HRMS (ESI) calcd for $C_{41}H_{32}O_9Na$ $[M+Na]^+$ 691.1939, found 691.1933.

2,3,5,6-tetra-*O*-benzoyl-D-galactofuranosyl 2-(1-phenylvinyl) benzoate (1m)

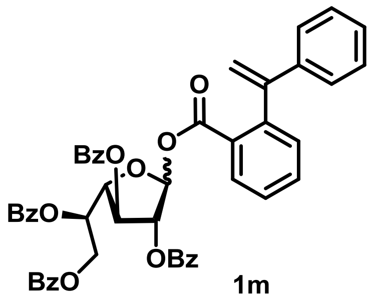

1m

Compound **1m** was prepared from **Sm**[12](100 mg, 0.17 mmol) according to **General procedure A**. The crude product was purified by silica gel column chromatography (petroleum ether/EtOAc = 4:1) to afford **1m** (108 mg, 80%, α/β = 1:4.6) as a white solid. The β anomer: $[\alpha]_D^{25}$ = -24.4 (c 0.10, CHCl3); ^{1}H NMR (400 MHz, $CDCl_3$) δ 7.92 (d, *J* = 7.8 Hz, 2H), 7.88 (d, *J* = 5.7 Hz, 1H), 7.85 (d, *J* = 7.4 Hz, 2H), 7.78 (d, *J* = 7.8 Hz, 2H), 7.52 (d, *J* = 7.7 Hz, 1H), 7.48 (d, *J* = 7.9 Hz, 1H), 7.45–7.14 (m, 16H), 7.09–7.06 (m, 2H), 6.34 (s, 1H), 5.91 (dt, *J* = 7.4, 3.8 Hz, 1H), 5.67 (s, 1H), 5.53 (d, *J* = 4.9 Hz, 1H), 5.42 (s, 1H), 5.16 (s, 1H), 4.59 (dd, *J* = 12.1, 4.0 Hz, 1H), 4.53 (dd, *J* = 12.0, 7.2 Hz, 1H), 4.38 (t, *J* = 4.3 Hz, 1H); ^{13}C NMR (101 MHz, $CDCl_3$) δ 166.07, 165.66, 165.57, 165.39, 165.01, 148.82, 143.15, 140.17, 133.65, 133.49, 133.26, 133.07, 132.25, 131.42, 130.26, 130.05, 130.03, 129.97, 129.95, 129.86, 129.73, 129.54, 129.31, 128.91, 128.56, 128.47, 128.44, 128.39, 128.36, 128.34, 128.28, 127.83, 127.65, 126.68, 114.58, 99.92, 83.62, 81.03, 69.98, 63.67; HRMS (ESI) calcd for $C_{49}H_{38}O_{11}Na$ $[M+Na]^+$ 825.2306, found 825.2316.

1,3,4,6-tetra-*O*-benzoyl-D-fructofuranosyl 2-(1-phenylvinyl) benzoate (1n)

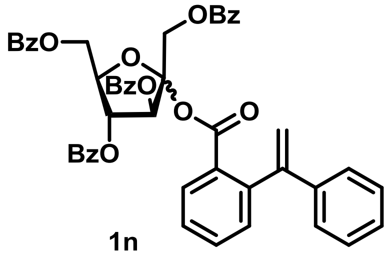

1n

Compound **1n** was prepared from **Sn**[13] (100 mg, 0.17 mmol) according to **General procedure A**. The crude product was purified by silica gel column chromatography (petroleum ether/EtOAc = 4:1) to afford **1n** (115 mg, 85%, α/β = 1:1) as a white solid. α and β anomers: ^{1}H NMR (400 MHz, $CDCl_3$) δ 7.92–7.78 (m, 17H), 7.73 (d, *J* = 7.7

Hz, 1H), 7.50–6.92 (m, 40H), 6.19–6.10 (m, 2H), 6.05 (d, *J* = 2.1 Hz, 1H), 5.55 (d, *J* = 3.5 Hz, 2H), 5.49 (d, *J* = 5.1 Hz, 1H), 5.13 (s, 1H), 4.98 (s, 1H), 4.91 (d, *J* = 12.0 Hz, 1H), 4.64 (d, *J* = 12.1 Hz, 1H), 4.58–4.38 (m, 7H), 4.03 (q, *J* = 4.3 Hz, 1H); ^{13}C NMR (101 MHz, $CDCl_3$) δ 166.09, 166.06, 165.45, 165.41, 165.32, 165.20, 164.88, 164.73, 164.36, 149.12, 148.89, 143.47, 143.38, 140.70, 140.19, 133.69, 133.64, 133.57, 133.23, 133.20, 133.07, 133.03, 132.32, 132.28, 131.71, 131.67, 130.44, 130.38, 130.27, 130.05, 130.02, 129.95, 129.90, 129.87, 129.75, 129.72, 129.66, 129.52, 129.50, 129.37, 128.98, 128.90, 128.75, 128.69, 128.62, 128.57, 128.50, 128.47, 128.42, 128.39, 128.31, 128.27, 128.18, 127.79, 127.65, 127.56, 126.81, 126.59, 114.78, 114.43, 109.49, 105.48, 82.58, 80.17, 79.95, 77.62, 77.51, 65.05, 64.90, 63.37, 61.96; HRMS (ESI) calcd for $C_{49}H_{38}O_{11}Na$ $[M+Na]^+$ 825.2306, found 825.2314.

2-*O*-benzoyl-3,4,6-tri-*O*-benzyl-D-glucopyranosyl 2-(1-phenylvinyl) benzoate (1o)

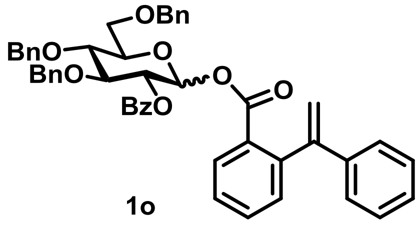

1o

Compound **1o** was prepared from **So**[14] (295 mg, 0.53 mmol) according to **General procedure A**. The crude product was purified by silica gel column chromatography (petroleum ether/EtOAc = 5:1) to afford **1o** (367 mg, 91%, α/β =1:10.6) as a white solid. The β anomer: $[\alpha]_D^{25}$ = 29.6 (c 0.11, CHCl3); ^{1}H NMR (400 MHz, $CDCl_3$) δ 7.90 (d, *J* = 7.8 Hz, 3H), 7.54 (t, *J* = 7.5 Hz, 1H), 7.47 (t, *J* = 7.5 Hz, 1H), 7.43–7.06 (m, 24H), 5.77 (d, *J* = 8.2 Hz, 1H), 5.57 (s, 1H), 5.45 (t, *J* = 8.6 Hz, 1H), 5.00 (s, 1H), 4.80 (d, *J* = 10.7 Hz, 1H), 4.73 (d, *J* = 11.1 Hz, 1H), 4.64 (d, *J* = 11.0 Hz, 1H), 4.62–4.54 (m, 2H), 4.47 (d, *J* = 12.1 Hz, 1H), 3.89 (t, *J* = 9.2 Hz, 1H), 3.82 (t, *J* = 8.9 Hz, 1H), 3.75 (dd, *J* = 11.1, 3.6 Hz, 1H), 3.68 (d, *J* = 11.8 Hz, 1H), 3.63–3.57 (m, 1H); ^{13}C NMR (101 MHz, $CDCl_3$) δ 165.12, 164.75, 149.01, 143.91, 140.33, 138.04, 137.94, 137.69, 133.25, 132.39, 131.39, 130.75, 129.86, 129.55, 128.88, 128.49, 128.44, 128.35, 128.33, 128.15, 128.07, 128.03, 127.96, 127.92, 127.81, 127.76,

127.73, 127.70, 127.45, 126.52, 114.02, 92.60, 82.68, 76.03, 75.18, 75.11, 73.64, 72.59, 68.12; HRMS (ESI) calcd for $C_{49}H_{44}O_8Na$ $[M+Na]^+$ 783.2928, found 783.2944.

2-*O*-benzoyl-3-*O*-benzyl-6-*O*-[tris(1-methylethyl)silyl]-D-arabinofuranosyl 2-(1-phenylvinyl) benzoate (1p)

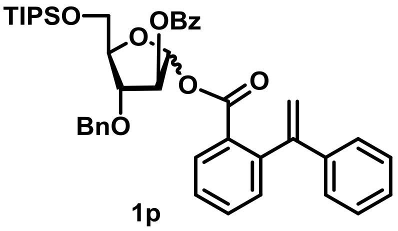

1p

Compound **1p** was prepared from **Sp**[15] (100 mg, 0.20 mmol) according to **General procedure A**. The crude product was purified by silica gel column chromatography (petroleum ether/EtOAc = 5:1) to afford **1p** (128mg, 91%, α/β = 3.3:1) as a white solid. The α anomer: $[\alpha]_D^{25}$ = 21.3 (c 0.16, $CHCl_3$); ^1H NMR (400 MHz, $CDCl_3$) δ 7.91 (d, *J* = 7.8 Hz, 2H), 7.83 (d, *J* = 7.7 Hz, 1H), 7.49 (t, *J* = 7.5 Hz, 1H), 7.43 (t, *J* = 7.5 Hz, 1H), 7.35 (t, *J* = 7.7 Hz, 2H), 7.30–7.17 (m, 9H), 7.15–7.08 (m, 3H), 6.12 (s, 1H), 5.64 (s, 1H), 5.30 (s, 1H), 5.15 (s, 1H), 4.69 (d, *J* = 11.9 Hz, 1H), 4.51 (d, *J* = 11.9 Hz, 1H), 4.14 (q, *J* = 4.8 Hz, 1H), 4.08 (d, *J* = 4.4 Hz, 1H), 3.74 (dd, *J* = 10.7, 4.3 Hz, 1H), 3.65 (dd, *J* = 10.7, 5.6 Hz, 1H), 0.89 (s, 21H); ^{13}C NMR (101 MHz, $CDCl_3$) δ 166.04, 165.21, 149.10, 143.24, 140.54, 137.90, 133.69, 133.57, 132.04, 131.37, 130.55, 130.40, 129.89, 129.35, 128.52, 128.41, 128.25, 127.88, 127.77, 127.68, 127.64, 126.91, 114.51, 100.71, 86.13, 82.73, 81.18, 72.19, 62.56, 18.00, 17.99, 11.96; HRMS (ESI) calcd for $C_{43}H_{50}O_7Si$ Na $[M+Na]^+$ 729.3218, found 729.3191.

2-*O*-benzoyl-3-*O*-benzyl-6-*O*-[tris(1-methylethyl)silyl]-L-arabinofuranosyl 2-(1-phenylvinyl) benzoate (1q)

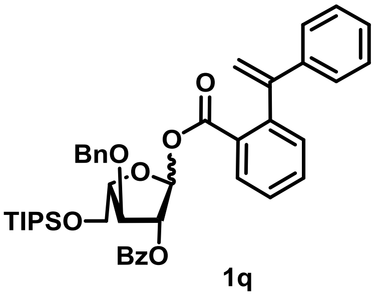

1q

Compound **1q** was prepared from **Sq**[16] (125 mg, 0.25 mmol) according to **General procedure A**. The crude product was purified by silica gel column chromatography (petroleum ether/EtOAc = 20:1) to afford **1q** (156mg, 88%, α/β = 2.2:1) as a white foam. The α anomer: $[\alpha]_D^{25}$ = -55.8 (c 0.10, $CHCl_3$); ^1H NMR (400 MHz, $CDCl_3$) δ 7.91 (d, *J* = 7.5 Hz, 2H), 7.83 (d, *J* = 7.6 Hz, 1H), 7.49 (t, *J* = 7.3 Hz, 1H), 7.43 (t, *J* = 7.4 Hz, 1H), 7.35 (t, *J* = 7.6 Hz, 2H), 7.29–7.08 (m, 12H), 6.12 (s, 1H), 5.64 (s, 1H), 5.29 (s, 1H), 5.15 (s, 1H), 4.69 (d, *J* = 11.9 Hz, 1H), 4.51 (d, *J* = 11.9 Hz, 1H), 4.17–4.11 (m, 1H), 4.08 (d, *J* = 4.0 Hz, 1H), 3.74 (dd, *J* = 10.7, 4.1 Hz, 1H), 3.65 (dd, *J* = 10.8, 5.6 Hz, 1H), 0.89 (s, 21H); ^{13}C NMR (151 MHz, $CDCl_3$) δ 166.04, 165.21, 149.10, 143.24, 140.54, 137.90, 133.57, 132.04, 131.37, 130.55, 130.39, 129.89, 129.34, 128.52, 128.41, 128.25, 127.88, 127.77, 127.68, 127.64, 126.91, 114.51, 100.70, 86.13, 82.71, 81.17, 72.18, 62.54, 17.99, 17.98, 11.95; HRMS (ESI) calcd for $C_{43}H_{50}O_7SiNa$ $[M+Na]^+$ 729.3218, found 729.3216.

2,3,4,6- tetra-*O*-benzyl-D-glucopyranosyl 2-(1-phenylvinyl) benzoate (1r)

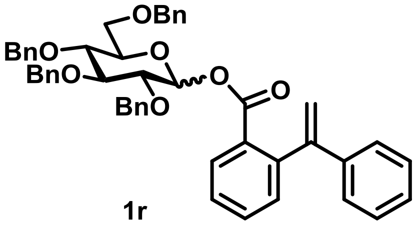

1r

Compound **1r** was prepared from **Sr**[16] (100 mg, 0.18 mmol) according to **General procedure A**. The crude product was purified by silica gel column chromatography (petroleum ether/EtOAc = 3:1) to afford **1r** (127mg, 92%, α/β = 1:4) as a white foam. α and β anomers: ^1H NMR (400 MHz, $CDCl_3$) δ 7.87 (d, *J* = 7.8 Hz, 4H), 7.82 (d, *J* = 7.7 Hz, 1H), 7.47–7.37 (m, 6H), 7.31 (t, *J* = 7.6 Hz, 6H), 7.23–7.06 (m, 128H), 6.32

(d, J = 3.4 Hz, 1H), 5.61 (s, 5H), 5.55 (d, J = 7.9 Hz, 4H), 5.08 (d, J = 7.8 Hz, 4H), 4.75 (d, J = 10.9 Hz, 4H), 4.73–4.67 (m, 10H), 4.55–4.31 (m, 27H), 3.71–3.33 (m, 30H); ^{13}C NMR (151 MHz, $CDCl_3$) δ 166.11, 164.95, 149.08, 148.50, 143.94, 143.11, 140.53, 140.31, 138.80, 138.48, 138.44, 138.17, 138.15, 138.06, 137.96, 137.90, 132.36, 131.86, 131.66, 131.18, 130.82, 130.63, 130.44, 129.49, 128.47, 128.44, 128.43, 128.38, 128.34, 128.27, 128.04, 127.99, 127.94, 127.85, 127.82, 127.76, 127.75, 127.70, 127.64, 127.61, 127.05, 126.63, 114.76, 114.29, 94.60, 90.77, 84.88, 81.65, 80.97, 78.93, 75.72, 75.66, 75.56, 75.07, 75.00, 74.82, 73.60, 73.58, 73.11, 72.86, 68.27, 68.04; HRMS (ESI) calcd for $C_{49}H_{46}O_7Na$ $[M+Na]^+$ 769.3136, found 769.3142.

3,4,6-tri-*O*-acetyl-2-deoxy-D-glucopyranosyl 2-(1-phenylvinyl) benzoate (1s)

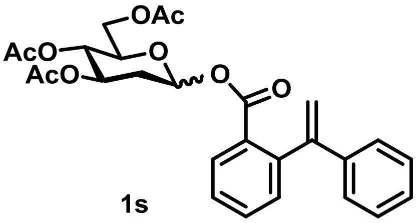

1s

Compound **1s** was prepared from **Ss**[17] (115 mg, 0.40 mmol) according to **General procedure A**. The crude product was purified by silica gel column chromatography (petroleum ether/EtOAc = 5:1) to afford **1s** (156mg, 79%, α/β = 1:4) as a colorless syrup. The β anomer: $[\alpha]_D^{25}$ = -10.7 (c 0.16, $CHCl_3$); ^{1}H NMR (400 MHz, $CDCl_3$) δ 7.89 (d, J = 7.7 Hz, 1H), 7.59–7.52 (m, 1H), 7.43 (t, J = 7.6 Hz, 1H), 7.37 (d, J = 7.6 Hz, 1H), 7.30–7.21 (m, 5H), 5.73 (s, 1H), 5.64 (dd, J = 9.9, 2.3 Hz, 1H), 5.23 (s, 1H), 4.99–4.93 (m, 2H), 4.26 (dd, J = 12.3, 4.7 Hz, 1H), 4.03 (dd, J = 12.3, 2.3 Hz, 1H), 3.69–3.63 (m, 1H), 2.12–2.08 (m, 1H), 2.06 (s, 3H), 2.02 (s, 6H), 1.70–1.65 (m, 1H); ^{13}C NMR (101 MHz, $CDCl_3$) δ 170.70, 170.01, 169.76, 164.98, 149.01, 143.22, 140.38, 132.33, 131.48, 130.23, 129.50, 128.19, 127.64, 126.67, 114.43, 91.67, 72.72, 70.12, 68.31, 62.02, 34.29, 20.86, 20.77, 20.71; HRMS (ESI) calcd for $C_{27}H_{28}O_9Na$ $[M+Na]^+$ 519.1625, found 519.1626.

2,3-di-*O*-benzoyl-4-methyl-D-ribofuranosyl 2-(1-phenylvinyl) benzoate (1t)

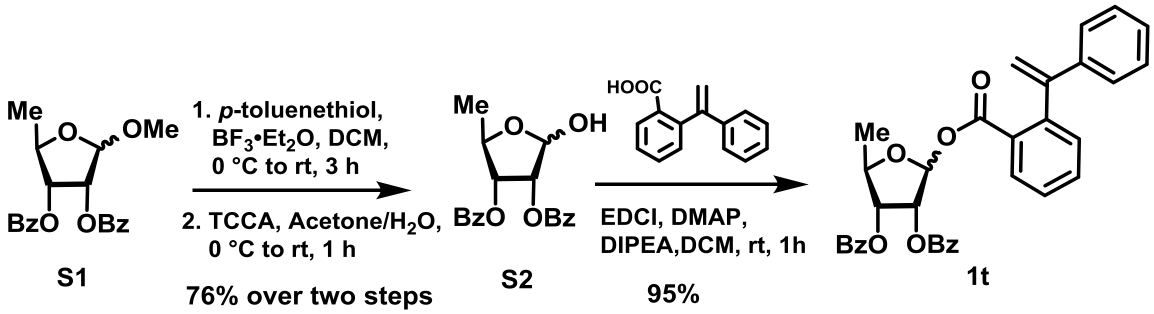

Supplementary Figure 2. Synthesis of compound **1t**.

To a solution of compound **S1**[18] (274 mg, 0.77 mmol) and *p*-toluenethiol (124 mg, 1.0 mmol) in dry CH_2Cl_2 (6 mL) was slowly added $BF_3 \cdot Et_2O$ (0.618 mL, 5 mmol) at 0 °C under argon atmosphere. The solution was allowed to stir at room temperature for 3 h. Then it was quenched with saturated aqueous $NaHCO_3$, extracted with CH_2Cl_2 and washed with brine. The organic phase was dried by Na_2SO_4, filtered, and concentrated *in vacuo*. Purification by silica gel column chromatography (EtOAc/Petroleum ether =1/7) afforded a light yellow liquid (296 mg, 86%). The light yellow liquid was dissolved in Acetone / H_2O (13 mL/3.3 mL), then trichloroisocyanuric acid (TCCA) (153mg, 0.66 mmol) was added at 0 °C. The solution was stirred for 1 h at 0 °C, then it was diluted with EtOAc and successively washed with saturated aqueous $NaHCO_3$, H_2O. The organic phase was dried by Na_2SO_4 and concentrated *in vacuo*. Purification by silica gel column chromatography (EtOAc/Petroleum ether =1/2.5) afforded **S2** (196 mg, 88%) as a syrup.

To a solution of compound **S2** (184 mg, 0.53 mmol) and 2-(1-Phenylvinyl) benzoic acid (132 mg, 0.58 mmol) in dry CH_2Cl_2 (5.4 mL) was added 4-dimethylaminopyridine (DMAP) (65 mg, 0.53 mmol), N-(3-dimethylaminopropyl)-N'-ethylcarbodiimide hydrochloride (EDCI) (186 mg, 0.95 mmol) and N,N-diisopropylethylamine (DIPEA) (0.266 mL, 1.59 mmol). The resulting mixture was stirred at room temperature until TLC-analysis indicated the reaction to be complete. Then, the mixture was diluted with CH_2Cl_2, and washed with water and brine. The organic layer was dried over Na_2SO_4, filtered, and concentrated *in vacuo*. The residue was purified by silica gel column chromatography to afford the **1t** (275 mg, 95%, α/β=1/4) as a colorless syrup. The data of β-anomer: $[\alpha]_D^{25}$ = 1.19 (c

0.24, $CHCl_3$); ^{1}H NMR (400 MHz, $CDCl_3$) δ 7.96 (d, J = 7.7 Hz, 3H), 7.86 (d, J = 7.7 Hz, 2H), 7.62–7.20 (m, 14H), 6.26 (s, 1H), 5.90 (s, 1H), 5.46 (d, J = 4.8 Hz, 1H), 5.28–5.24 (m, 2H), 4.48-4.45 (m, 1H), 1.28 (d, J = 6.5 Hz, 3H); ^{13}C NMR (100 MHz, $CDCl_3$) δ 166.07, 165.34, 164.90, 148.89, 142.98, 139.96, 133.49, 133.35, 132.27, 131.41, 130.49, 129.99, 129.82, 129.70, 129.08, 128.48, 128.42, 128.37, 127.87, 127.71, 126.65, 114.21, 99.23, 78.86, 75.75, 75.33, 19.72; HRMS (ESI) calcd for $C_{34}H_{28}O_7Na$ $[M+Na]^+$ 571.1727, found 571.1727.

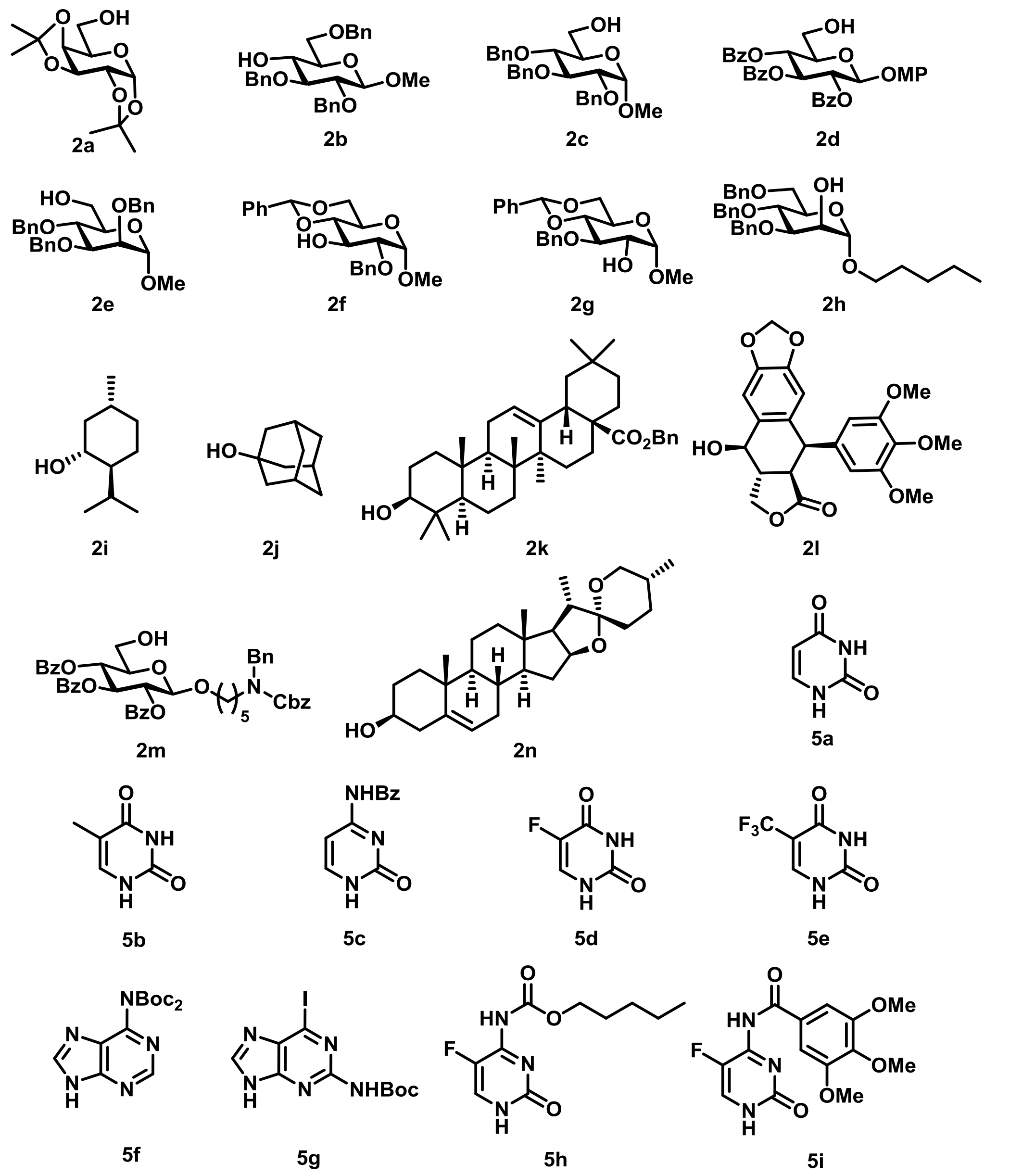

Supplementary Figure 3. The acceptors **2a-n** and **5a-i**. Compounds **2b**, **2c**, **2d**, **2e**, **2f**, **2g**, **2h**, **2k**, **2m**, **5f**, **5g**, **5h** and **5i** were synthesized following literature procedures.[19-28]

Compounds **2a**, **2i**, **2j**, **2l**, **2n**, **5a**, **5b**, **5c**, **5d** and **5e** are commercially available.

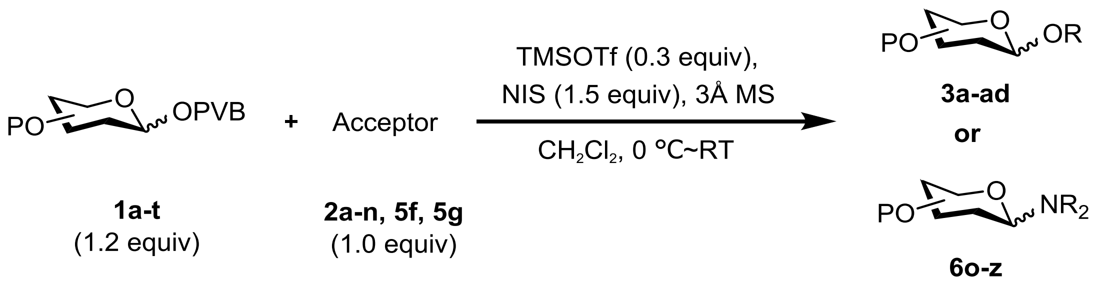

Supplementary Figure 4. Glycosylation of glycosyl PVB with alcoholic acceptors or purines nucleobases. Note: the synthesis of products **6o-z** performed at −20 °C for 3h.

General procedure B. A solution of glycosyl PVB donor **1** (1.2 eq.) and acceptor **2** (1.0 eq.) in dry CH_2Cl_2 (0.033 M) was stirred at room temperature for 30 mins in the presence of activated 3 Å MS (3.0 g/mmol) under Ar atmosphere. Then the vessel was chilled to 0 ℃, to which NIS (1.5 eq.) and TMSOTf (0.3 eq.) were added. The reaction mixture was stirred for 2 h after the temperature gradually rise to room temperature. Then Et_3N was added to quench the reaction and the solvent was removed under reduced pressure. The resulting residue was purified by silica gel column chromatography to afford the glycosylated product.

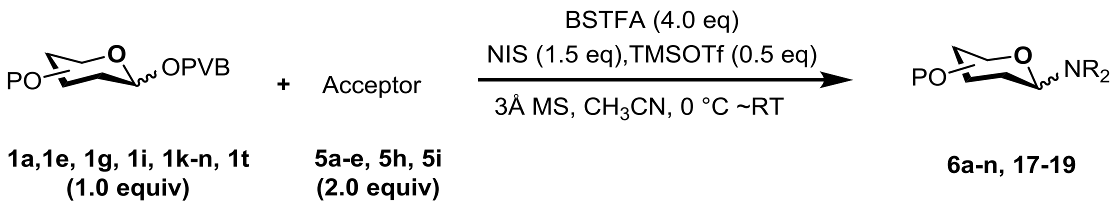

Supplementary Figure 5. Glycosylation of glycosyl PVB with pyrimidines nucleobases.

General procedure C. BSTFA (4.0 eq) was added to a stirred suspension of acceptor **5** (2.0 eq.) in dry CH_3CN (0.1 M) under Ar atmosphere. After the mixture was stirred at 50 ℃ for 30 mins, this solution was added to a solution of glycosyl donor **1** (1.0 eq) and activated 3Å MS (4.0 g/mmol) in dry CH_3CN (0.05 M), which has been stirred at room temperature for 30 mins under Ar atmosphere. The stirring was continued for 10 mins, then the vessel was chilled to 0 ℃, to which NIS (1.5 eq.) and TMSOTf (0.5 eq.)

were added. The reaction mixture was stirred for 3 h after the temperature gradually rise to room temperature. Et_3N was added to quench the reaction and the solvent was removed under reduced pressure. The resulting residue was purified by silica gel column chromatography to afford the glycosylated product.

Compound 3a

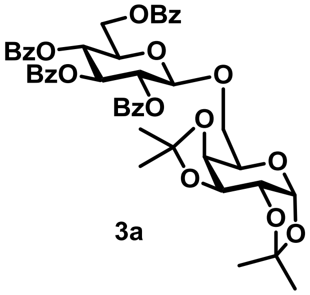

3a

Glycosylation of **1a** (65.9 mg, 0.08mmol) with **2a** (17.8 mg, 0.07 mmol) according to **General procedure B** afforded **3a**[29] (53.5 mg, 94%) as a white solid: $[\alpha]_D^{25}$ = -41.4 (*c* 0.15, $CHCl_3$); ^1H NMR (400 MHz, $CDCl_3$): δ 8.03 (d, *J* = 7.6 Hz, 2H), 7.97 (d, *J* = 7.6 Hz, 2H), 7.90 (d, *J* = 7.6 Hz, 2H), 7.83 (d, *J* = 7.6 Hz, 2H), 7.56–7.45 (m, 3H), 7.25–7.41 (m, 9H), 5.92 (t, *J* = 9.5 Hz, 1H), 5.70 (d, *J* = 9.6 Hz, 1H), 5.55 (t, *J* = 8.6 Hz, 1H), 5.43 (d, *J* = 4.7 Hz, 1H), 5.06 (d, *J* = 7.7 Hz, 1H), 4.65 (d, *J* = 10.3 Hz, 1H), 4.50 (dd, *J* = 12.0, 5.0 Hz, 1H), 4.43 (d, *J* = 7.5 Hz, 1H), 4.26–4.15 (m, 2H), 4.10 (d, *J* = 7.8 Hz, 1H), 4.07–3.98 (m, 1H), 3.93–3.83 (m, 2H), 1.37 (s, 3H), 1.24 (s, 3H), 1.20 (s, 6H); ^{13}C NMR (101 MHz, $CDCl_3$) δ 166.18, 165.82, 165.22, 165.18, 133.43, 133.21, 133.09, 130.03, 129.84, 129.78, 129.65, 129.38, 128.87, 128.84, 128.41, 128.37, 128.29, 128.22, 109.28, 108.50, 101.25, 96.18, 73.05, 72.19, 71.84, 71.01, 70.55, 70.36, 69.84, 68.31, 67.57, 63.27, 25.91, 25.70, 24.88, 24.27; HRMS (ESI) calcd for $C_{46}H_{46}O_{15}Na$ $[M+Na]^+$ 861.2729, found 861.2733.

Compound 3b

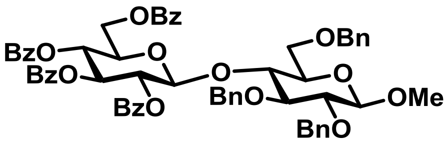

3b

Glycosylation of **1a** (62.0 mg, 0.08mmol) with **2b** (29.9 mg, 0.06 mmol) according to **General procedure B** afforded **3b**[30] (66.4 mg, 99%) as a white solid: $[\alpha]_D^{25}$ = -10.9 (*c* 0.20, $CHCl_3$); ^{1}H NMR (400 MHz, $CDCl_3$): δ 7.96 (d, *J* = 7.8 Hz, 2H), 7.90 (d, *J* = 7.8 Hz, 2H), 7.86 (d, *J* = 7.7 Hz, 2H), 7.80 (d, *J* = 7.8 Hz, 2H), 7.54–7.16 (m, 27H), 5.68 (t, *J* = 9.6 Hz, 1H), 5.57 (t, *J* = 9.6 Hz, 1H), 5.48 (t, *J* = 8.9 Hz, 1H), 5.06 (d, *J* = 11.2 Hz, 1H), 4.94 (d, *J* = 8.1 Hz, 1H), 4.82 (d, *J* = 7.5 Hz, 1H), 4.79 (d, *J* = 7.7 Hz, 1H), 4.72 (d, *J* = 12.1 Hz, 1H), 4.65 (d, *J* = 11.1 Hz, 1H), 4.42–4.37 (m, 2H), 4.24 (dd, *J* = 12.1, 5.0 Hz, 1H), 4.20 (d, *J* = 7.8 Hz, 1H), 4.04 (t, *J* = 9.3 Hz, 1H), 3.73–3.66 (m, 2H), 3.64–3.56 (m, 2H), 3.49 (s, 3H), 3.37 (t, *J* = 8.4 Hz, 1H), 3.23 (d, *J* = 9.7 Hz, 1H); ^{13}C NMR (101 MHz, $CDCl_3$) δ 166.04, 165.76, 165.11, 164.89, 139.02, 138.55, 138.15, 133.40, 133.21, 133.01, 129.77, 129.72, 129.62, 129.08, 128.89, 128.86, 128.73, 128.52, 128.41, 128.30, 128.24, 128.14, 128.12, 128.09, 127.96, 127.51, 127.43, 127.23, 104.57, 100.38, 82.48, 81.74, 75.23, 74.78, 74.30, 73.54, 73.15, 72.31, 71.96, 69.76, 67.83, 63.06, 57.07; HRMS (ESI) calcd for $C_{62}H_{58}O_{15}Na$ $[M+Na]^+$ 1065.3668, found 1065.3673.

Compound 3c

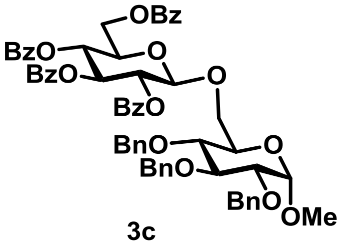

3c

Glycosylation of **1a** (62.0 mg, 0.08mmol) with **2c** (29.9 mg, 0.06 mmol) according to **General procedure B** afforded **3c**[31] (65.1 mg, 97%) as a white solid: $[\alpha]_D^{25}$ = 6.5 (*c* 0.10, $CHCl_3$); ^{1}H NMR (400 MHz, $CDCl_3$): δ 7.91 (d, *J* = 7.8 Hz, 2H), 7.81 (d, *J* = 7.8 Hz, 4H), 7.74 (d, *J* = 7.8 Hz, 2H), 7.45–7.38 (m, 2H), 7.31–7.12 (m, 23H), 7.01–6.94 (m, 2H), 5.81 (t, *J* = 9.6 Hz, 1H), 5.60 (t, *J* = 9.7 Hz, 1H), 5.52 (t, *J* = 8.7 Hz, 1H), 4.81 (d, *J* = 10.9 Hz, 1H), 4.74 (d, *J* = 7.8 Hz, 1H), 4.65 (d, *J* = 12.1 Hz, 1H), 4.60 (d, *J* = 10.9 Hz, 1H), 4.55–4.50 (m, 2H), 4.47–4.40 (m, 3H), 4.20 (d, *J* = 11.1 Hz, 1H), 4.07 (d, *J* = 9.8 Hz, 1H), 4.02 (dd, *J* = 9.7, 4.8 Hz, 1H), 3.80 (t, *J* = 9.3 Hz, 1H),

3.70–3.61 (m, 2H), 3.35 (dd, *J* = 9.7, 3.6 Hz, 1H), 3.30 (t, *J* = 9.3 Hz, 1H), 3.13 (s, 3H); ^{13}C NMR (101 MHz, $CDCl_3$) δ 165.07, 164.81, 164.12, 163.89, 137.74, 137.14, 137.09, 132.40, 132.22, 132.11, 132.07, 128.76, 128.70, 128.67, 128.50, 128.11, 127.73, 127.68, 127.39, 127.36, 127.31, 127.29, 127.26, 127.23, 127.07, 126.85, 126.83, 126.56, 126.42, 100.29, 96.90, 80.84, 78.72, 74.50, 73.66, 72.33, 71.82, 71.15, 70.77, 68.74, 68.43, 67.27, 62.21, 53.97; HRMS (ESI) calcd for $C_{62}H_{58}O_{15}Na$ $[M+Na]^+$ 1065.3668, found 1065.3667.

Compound 3d

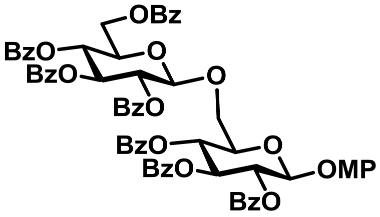

3d

Glycosylation of **1a** (56.3 mg, 0.07mmol) with **2d** (35.0 mg, 0.06 mmol) according to **General procedure B** afforded **3d** (65.4 mg, 95%) as a white solid: $[\alpha]_D^{25}$ = -2.27 (*c* 0.11, $CHCl_3$); ^{1}H NMR (400 MHz, $CDCl_3$): δ 8.04 (d, *J* = 7.7 Hz, 2H), 7.95 (d, *J* = 7.8 Hz, 2H), 7.90 (d, *J* = 7.7 Hz, 2H), 7.87–7.80 (m, 8H), 7.57–7.24 (m, 21H), 6.96 (d, *J* = 8.8 Hz, 2H), 6.86 (d, *J* = 8.8 Hz, 2H), 5.87 (d, *J* = 12.0 Hz, 1H), 5.84 (d, *J* = 12.0 Hz, 1H), 5.72–5.60 (m, 2H), 5.51 (dd, *J* = 9.7, 7.8 Hz, 1H), 5.40 (t, *J* = 9.7 Hz, 1H), 5.19 (d, *J* = 7.8 Hz, 1H), 5.06 (d, *J* = 7.9 Hz, 1H), 4.59 (dd, *J* = 12.2, 3.0 Hz, 1H), 4.42 (dd, *J* = 12.2, 5.1 Hz, 1H), 4.18–4.11 (m, 1H), 4.08–3.95 (m, 3H), 3.76 (s, 3H); ^{13}C NMR (101 MHz, $CDCl_3$) δ 166.09, 165.73, 165.69, 165.39, 165.20, 165.18, 165.08, 155.94, 150.95, 133.57, 133.48, 133.32, 133.27, 133.21, 129.86, 129.81, 129.76, 129.55, 129.24, 129.19, 128.82, 128.80, 128.77, 128.63, 128.48, 128.42, 128.36, 128.32, 118.94, 114.76, 100.75, 100.64, 74.84, 72.96, 72.74, 72.32, 71.97, 71.84, 69.61, 69.50, 67.86, 62.88, 55.67; HRMS (ESI) calcd for $C_{68}H_{56}O_{19}Na$ $[M+Na]^+$ 1199.3308, found 1199.3307.

Compound 3e

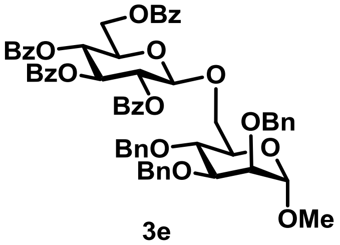

3e

Glycosylation of **1a** (62.0 mg, 0.08mmol) with **2e** (29.9 mg, 0.06 mmol) according to **General procedure B** afforded **3e**[32] (63.8 mg, 95%) as a white solid: $[\alpha]_D^{25}$ = 4.61 (*c* 0.18, $CHCl_3$); ^1H NMR (400 MHz, $CDCl_3$): δ 8.07 (d, *J* = 7.7 Hz, 2H), 7.95 (d, *J* = 7.8 Hz, 4H), 7.88 (d, *J* = 7.7 Hz, 2H), 7.56–7.25 (m, 27H), 5.97 (t, *J* = 9.7 Hz, 1H), 5.75 (t, *J* = 9.7 Hz, 1H), 5.65 (dd, *J* = 9.8, 7.8 Hz, 1H), 5.03 (d, *J* = 7.8 Hz, 1H), 4.79 (d, *J* = 11.1 Hz, 1H), 4.72 (s, 2H), 4.68 (dd, *J* = 12.1, 3.2 Hz, 1H), 4.63–4.54 (m, 4H), 4.48 (d, *J* = 11.1 Hz, 1H), 4.27 (d, *J* = 9.7 Hz, 1H), 4.24–4.16 (m, 1H), 3.87–3.69 (m, 5H), 3.03 (s, 3H); ^{13}C NMR (101 MHz, $CDCl_3$) δ 166.19, 165.91, 165.19, 165.05, 138.43, 138.35, 138.28, 133.44, 133.25, 133.11, 133.04, 129.84, 129.78, 129.61, 129.48, 128.86, 128.83, 128.43, 128.37, 128.33, 128.31, 128.27, 127.90, 127.75, 127.68, 127.64, 127.56, 127.54, 101.76, 98.66, 80.24, 75.04, 74.92, 74.30, 73.03, 72.67, 72.13, 72.04, 71.98, 71.36, 69.83, 69.62, 63.21, 54.33; HRMS (ESI) calcd for $C_{62}H_{58}O_{15}Na$ $[M+Na]^+$ 1065.3668, found 1065.3679.

Compound 3f

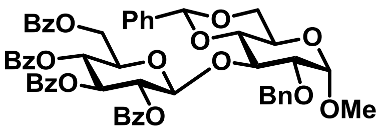

3f

Glycosylation of **1a** (70.1 mg, 0.08mmol) with **2f** (25.0 mg, 0.07 mmol) according to **General procedure B** afforded **3f**[33] (62.4 mg, 98%) as a white solid: $[\alpha]_D^{25}$ = -40.2 (*c* 0.10, $CHCl_3$); ^1H NMR (400 MHz, $CDCl_3$): δ 7.88 (t, *J* = 8.4 Hz, 4H), 7.77 (d, *J* = 7.7 Hz, 2H), 7.72 (d, *J* = 7.7 Hz, 2H), 7.44–7.39 (m, 3H), 7.39–7.34 (m, 2H), 7.32–7.20 (m, 9H), 7.19–7.14 (m, 6H), 7.05–6.99 (m, 2H), 5.80 (t, *J* = 9.6 Hz, 1H), 5.66–5.54 (m, 2H), 5.48 (s, 1H), 5.17 (d, *J* = 7.9 Hz, 1H), 4.51 (d, *J* = 12.5 Hz, 1H), 4.41 (dd, *J*

= 11.9, 2.8 Hz, 1H), 4.25–4.16 (m, 3H), 4.16–4.07 (m, 2H), 3.92–3.84 (m, 1H), 3.67 (dd, *J* = 9.6, 4.0 Hz, 1H), 3.60 (t, *J* = 10.0 Hz, 1H), 3.53 (t, *J* = 9.2 Hz, 1H), 3.35 (dd, *J* = 9.1, 3.5 Hz, 1H), 3.18 (s, 3H); ^{13}C NMR (151 MHz, $CDCl_3$) δ 166.23, 165.97, 165.41, 165.30, 138.20, 137.36, 133.46, 133.33, 133.30, 133.07, 129.96, 129.92, 129.84, 129.73, 129.44, 129.10, 128.99, 128.95, 128.54, 128.51, 128.48, 128.40, 128.36, 128.29, 128.05, 127.95, 126.17, 101.46, 101.24, 99.02, 79.85, 79.56, 77.86, 74.21, 73.38, 72.43, 71.99, 69.95, 69.06, 63.32, 62.37, 55.37; HRMS (ESI) calcd for $C_{55}H_{50}O_{15}Na$ $[M+Na]^+$ 973.3042, found 973.3030.

Compound 3g

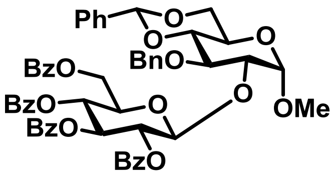

3g

Glycosylation of **1a** (50.0 mg, 0.06mmol) with **2g** (19.3 mg, 0.05 mmol) according to **General procedure B** afforded **3g**[34] (46.2 mg, 94%) as a white solid: $[\alpha]_D^{25}$ = -7.2 (*c* 0.11, $CHCl_3$); ^{1}H NMR (400 MHz, $CDCl_3$): δ 7.97 (d, *J* = 7.7 Hz, 2H), 7.85 (d, *J* = 7.8 Hz, 2H), 7.81 (d, *J* = 7.7 Hz, 2H), 7.73 (d, *J* = 7.8 Hz, 2H), 7.49 (t, *J* = 7.4 Hz, 1H), 7.42 (t, *J* = 7.4 Hz, 1H), 7.35–7.25 (m, 10H), 7.20–7.12 (m, 5H), 7.09–7.03 (m, 3H), 6.91 (d, *J* = 6.5 Hz, 2H), 5.84 (t, *J* = 9.6 Hz, 1H), 5.67–5.59 (m, 2H), 5.42 (s, 1H), 5.12 (d, *J* = 7.8 Hz, 1H), 4.89 (d, *J* = 3.6 Hz, 1H), 4.66 (dd, *J* = 12.2, 3.1 Hz, 1H), 4.46 (d, *J* = 11.7 Hz, 1H), 4.38 (dd, *J* = 12.2, 5.3 Hz, 1H), 4.32 (d, *J* = 11.7 Hz, 1H), 4.18 (dd, *J* = 10.1, 4.7 Hz, 1H), 4.09–4.04 (m, 1H), 3.85 (t, *J* = 9.2 Hz, 1H), 3.75–3.69 (m, 2H), 3.61 (t, *J* = 10.2 Hz, 1H), 3.45 (t, *J* = 9.3 Hz, 1H), 3.29 (s, 3H); ^{13}C NMR (101 MHz, $CDCl_3$) δ 166.05, 165.83, 165.23, 165.05, 138.39, 137.31, 133.54, 133.27, 133.19, 129.88, 129.82, 129.78, 129.74, 129.49, 129.13, 128.90, 128.75, 128.73, 128.48, 128.38, 128.31, 128.15, 128.09, 127.41, 127.30, 126.07, 102.38, 101.37, 100.30, 82.07, 80.83, 74.75, 73.14, 72.35, 72.03, 69.56, 69.15, 62.75, 62.11, 55.53; HRMS (ESI) calcd for $C_{55}H_{50}O_{15}Na$ $[M+Na]^+$ 973.3042, found 973.3035.

Compound 3h

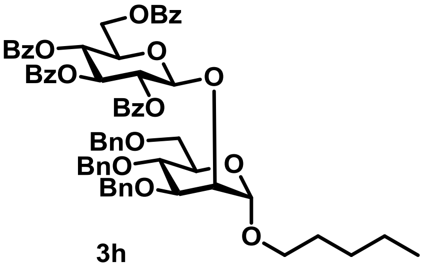

3h

Glycosylation of **1a** (67.9 mg, 0.09 mmol) with **2h** (40.0 mg, 0.08 mmol) according to **General procedure B** afforded **3h** (77.7 mg, 92%) as a colorless syrup: $[\alpha]_D^{25}$ = -15.7 (*c* 0.14, $CHCl_3$); ^{1}H NMR (400 MHz, $CDCl_3$): δ 7.90 (d, *J* = 7.6 Hz, 2H), 7.83 (t, *J* = 7.3 Hz, 4H), 7.77 (d, *J* = 7.5 Hz, 2H), 7.45–7.38 (m, 2H), 7.38–7.31 (m, 2H), 7.29–7.05 (m, 23H), 5.84 (t, *J* = 9.6 Hz, 1H), 5.69–5.53 (m, 2H), 5.01–4.92 (d, *J* = 7.6 Hz, 1H), 4.71 (s, 1H), 4.69(s, 2H), 4.63–4.54 (m, 1H), 4.50–4.40 (m, 2H), 4.31 (d, *J* = 10.8 Hz, 1H), 4.21–4.12 (m, 2H), 4.12–4.05 (m, 2H), 3.81 (s, 1H), 3.57 (d, *J* = 4.5 Hz, 2H), 3.51–3.41 (m, 2H), 3.22 (d, *J* = 6.6 Hz, 1H), 3.18–3.10 (m, 1H), 1.47–1.30 (m, 2H), 1.22–1.11 (m, 4H), 0.79 (t, *J* = 6.8 Hz, 3H); ^{13}C NMR (151 MHz, $CDCl_3$) δ 166.24, 165.95, 165.32, 165.00, 138.68, 138.46, 138.33, 133.54, 133.34, 133.25, 133.11, 129.93, 129.90, 129.86, 129.83, 129.62, 129.54, 128.92, 128.89, 128.52, 128.47, 128.40, 128.37, 128.35, 128.33, 128.27, 128.20, 128.14, 127.65, 127.59, 127.39, 100.29, 97.18, 78.26, 74.93, 73.16, 73.04, 72.53, 72.09, 72.00, 71.36, 70.00, 69.91, 67.83, 63.48, 29.16, 28.34, 22.54, 14.12; HRMS (ESI) calcd for $C_{66}H_{66}O_{15}Na$ $[M+Na]^+$ 1121.4294, found 1121.4274.

Compound 3i

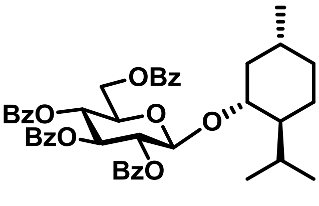

3i

Glycosylation of **1a** (184.9 mg, 0.23mmol) with **2i** (30.0 mg, 0.19 mmol) according to **General procedure B** afforded **3i**[35] (134.3 mg, 95%) as a white solid: $[\alpha]_D^{25}$ = -43.2 (*c*

0.10, $CHCl_3$); ^{1}H NMR (400 MHz, $CDCl_3$): δ 8.01 (d, *J* = 7.8 Hz, 2H), 7.97 (d, *J* = 7.8 Hz, 2H), 7.91 (d, *J* = 7.8 Hz, 2H), 7.84 (d, *J* = 7.7 Hz, 2H), 7.54–7.45 (m, 3H), 7.42–7.31 (m, 7H), 7.27 (t, *J* = 7.5 Hz, 2H), 5.91 (t, *J* = 9.7 Hz, 1H), 5.65 (t, *J* = 9.7 Hz, 1H), 5.51 (dd, *J* = 9.8, 7.9 Hz, 1H), 4.94 (d, *J* = 7.9 Hz, 1H), 4.64 (dd, *J* = 12.0, 3.4 Hz, 1H), 4.50 (dd, *J* = 12.0, 5.7 Hz, 1H), 4.15 (ddd, *J* = 9.5, 5.6, 3.4 Hz, 1H), 3.49 (td, *J* = 10.6, 4.1 Hz, 1H), 2.26 (dtd, *J* = 13.9, 7.1, 2.6 Hz, 1H), 1.95 (d, *J* = 12.5 Hz, 1H), 1.61–1.53 (m, 2H), 1.32–1.23 (m, 1H), 1.22–1.13 (m, 1H), 0.96–0.85 (m, 1H), 0.82 (d, *J* = 7.1 Hz, 3H), 0.75 (d, *J* = 6.4 Hz, 3H), 0.74–0.65 (m, 5H); ^{13}C NMR (101 MHz, $CDCl_3$) δ 166.13, 165.87, 165.32, 165.08, 133.41, 133.19, 133.13, 133.10, 129.84, 129.77, 129.73, 129.63, 129.53, 128.93, 128.87, 128.41, 128.31, 128.29, 99.03, 79.11, 73.24, 72.17, 72.03, 70.21, 63.48, 47.33, 40.79, 34.10, 31.39, 25.15, 23.04, 22.05, 20.82, 15.64; HRMS (ESI) calcd for $C_{44}H_{46}O_{10}Na$ $[M+Na]^+$ 757.2983, found 757.2988.

Compound 3j

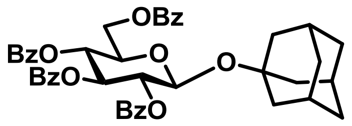

3j

Glycosylation of **1a** (126.6 mg, 0.16mmol) with **2j** (20.0 mg, 0.13 mmol) according to **General procedure B** afforded **3j**[36] (91.3 mg, 95%) as a white solid: $[\alpha]_D^{25}$ = -9.0 (*c* 0.13, $CHCl_3$); ^{1}H NMR (400 MHz, $CDCl_3$): δ 8.02 (d, *J* = 7.8 Hz, 2H), 7.96 (d, *J* = 7.8 Hz, 2H), 7.92 (d, *J* = 7.7 Hz, 2H), 7.83 (d, *J* = 7.7 Hz, 2H), 7.56–7.47 (m, 3H), 7.44–7.33 (m, 7H), 7.31–7.25 (m, 2H), 5.93 (t, *J* = 9.6 Hz, 1H), 5.56 (t, *J* = 9.7 Hz, 1H), 5.50 (t, *J* = 8.8 Hz, 1H), 5.13 (d, *J* = 7.9 Hz, 1H), 4.59 (dd, *J* = 12.0, 3.2 Hz, 1H), 4.49 (dd, *J* = 11.9, 7.1 Hz, 1H), 4.18 (ddd, *J* = 12.0, 7.3, 3.8 Hz, 1H), 2.05–1.99 (m, 3H), 1.82 (d, *J* = 11.7 Hz, 3H), 1.67 (s, 3H), 1.59–1.45 (m, 6H); ^{13}C NMR (101 MHz, $CDCl_3$) δ 166.06, 165.86, 165.35, 164.92, 133.44, 133.18, 133.09, 129.85, 129.77, 129.71, 129.66, 129.53, 128.91, 128.81, 128.43, 128.35, 128.31, 128.28, 94.33, 75.88, 73.27, 72.09, 71.93, 70.33, 63.71, 42.36, 36.03, 30.55; HRMS (ESI) calcd for

$C_{44}H_{42}O_{10}Na$ [M+Na]$^+$ 753.2670, found 753.2665.

Compound 3k

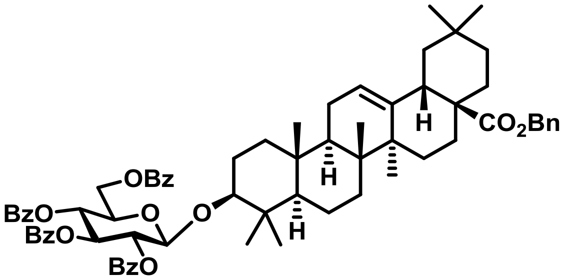

3k

Glycosylation of **1a** (80.0 mg, 0.10 mmol) with **2k** (44.2 mg, 0.08 mmol) according to **General procedure B** afforded **3k**[37] (78.8 mg, 87%) as a white solid: $[\alpha]_D^{25}$ = 22.3 (*c* 0.10, $CHCl_3$); ^{1}H NMR (400 MHz, $CDCl_3$): δ 8.05 (d, *J* = 7.7 Hz, 2H), 8.00–7.93 (m, 4H), 7.86 (d, *J* = 7.7 Hz, 2H), 7.59–7.50 (m, 3H), 7.44–7.30 (m, 14H), 5.95 (t, *J* = 9.7 Hz, 1H), 5.63 (t, *J* = 8.0 Hz, 1H), 5.59 (d, *J* = 6.9 Hz, 1H), 5.33 (d, *J* = 3.6 Hz, 1H), 5.12 (d, *J* = 12.5 Hz, 1H), 5.06 (d, *J* = 12.5 Hz, 1H), 4.89 (d, *J* = 7.9 Hz, 1H), 4.63 (dd, *J* = 12.0, 3.5 Hz, 1H), 4.58 (dd, *J* = 12.0, 6.3 Hz, 1H), 4.18 (ddd, *J* = 10.0, 6.4, 3.5 Hz, 1H), 3.13 (dd, *J* = 11.7, 4.5 Hz, 1H), 2.94 (dd, *J* = 13.7, 4.4 Hz, 1H), 2.00 (td, *J* = 12.0, 2.0 Hz, 1H), 1.88–0.60 (m, 21H), 1.12 (s, 3H), 0.96 (s, 3H), 0.94 (s, 3H), 0.83 (s, 3H), 0.71 (s, 3H), 0.66 (s, 3H), 0.58 (s, 3H); ^{13}C NMR (101 MHz, $CDCl_3$) δ 177.41, 166.03, 165.88, 165.32, 165.02, 143.71, 136.40, 133.47, 133.23, 133.11, 133.09, 129.86, 129.76, 129.74, 129.69, 129.41, 128.85, 128.79, 128.44, 128.41, 128.38, 128.30, 127.99, 127.92, 122.50, 103.32, 90.79, 72.99, 72.16, 72.00, 70.31, 65.95, 63.45, 55.45, 47.56, 46.73, 45.93, 41.63, 41.38, 39.26, 38.74, 38.24, 36.62, 33.88, 33.16, 32.62, 32.39, 30.74, 27.68, 27.60, 25.88, 25.84, 23.68, 23.38, 23.04, 18.08, 16.83, 16.21, 15.18; HRMS (ESI) calcd for $C_{71}H_{80}O_{12}Na$ [M+Na]$^+$ 1147.5542, found 1147.5552.

Compound 3l

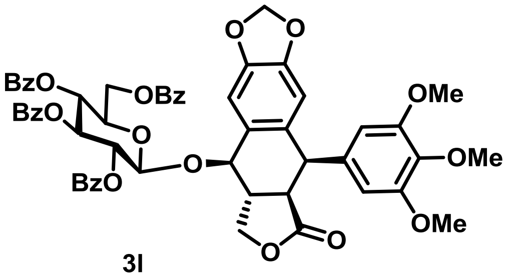

3l

Glycosylation of **1a** (69.7 mg, 0.09 mmol) with **2l** (30.0 mg, 0.07 mmol) according to **General procedure B** afforded **3l**[38] (67.3 mg, 94%) as a white solid: $[\alpha]_D^{25}$ = -80.6 (*c* 0.14, $CHCl_3$); ^{1}H NMR (400 MHz, $CDCl_3$): δ 7.92 (t, *J* = 6.8 Hz, 4H), 7.87 (d, *J* = 7.7 Hz, 2H), 7.81 (d, *J* = 7.6 Hz, 2H), 7.56–7.48 (m, 3H), 7.46–7.31 (m, 7H), 7.30–7.25 (m, 2H), 7.12 (s, 1H), 6.48 (s, 1H), 6.34 (s, 2H), 5.99–5.88 (m, 3H), 5.72–5.59 (m, 2H), 4.99 (d, *J* = 7.6 Hz, 1H), .4.98 (d, *J* = 10.0 Hz, 1H), 4.69 (dd, *J* = 12.1, 2.0 Hz, 1H), 4.59–4.44 (m, 3H), 4.23–4.14 (m, 1H), 4.07 (t, *J* = 9.4 Hz, 1H), 3.73 (s, 6H), 3.67 (s, 3H), 2.99–2.86 (m, 1H), 2.72 (dd, *J* = 14.4, 4.5 Hz, 1H); ^{13}C NMR (151 MHz, $CDCl_3$) δ 173.83, 166.13, 165.88, 165.24, 165.03, 152.69, 148.18, 147.74, 137.48, 135.16, 133.72, 133.48, 133.29, 132.43, 129.91, 129.83, 129.76, 129.72, 129.23, 129.19, 128.75, 128.70, 128.65, 128.64, 128.60, 128.53, 128.44, 109.59, 108.65, 108.08, 101.57, 99.06, 80.04, 72.81, 72.73, 71.83, 71.16, 69.64, 62.97, 60.68, 56.49, 45.51, 44.01, 38.61; HRMS (ESI) calcd for $C_{56}H_{48}O_{17}Na$ $[M+Na]^+$ 1015.2784, found 1015.2767.

Compound 3m

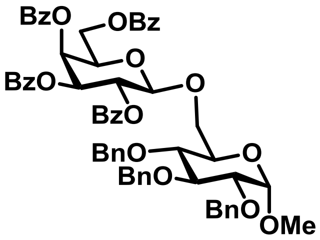

3m

Glycosylation of **1c** (107.8 mg, 0.13mmol) with **2c** (52.0 mg, 0.11 mmol) according to **General procedure B** afforded **3m**[39] (108.7 mg, 93%) as a white solid: $[\alpha]_D^{25}$ = 72.4 (*c* 0.11, $CHCl_3$); ^{1}H NMR (400 MHz, $CDCl_3$): δ 8.08 (d, *J* = 7.7 Hz, 2H), 8.02 (d, *J* = 7.8 Hz, 2H), 7.89 (d, *J* = 7.8 Hz, 2H), 7.77 (d, *J* = 7.8 Hz, 2H), 7.60 (t, *J* = 7.3 Hz, 1H), 7.53 (t, *J* = 7.4 Hz, 1H), 7.46 (t, *J* = 7.7 Hz, 2H), 7.39 (t, *J* = 7.9 Hz, 4H), 7.34–7.19 (m, 17H), 7.13 (d, *J* = 7.4 Hz, 2H), 5.98 (d, *J* = 3.5 Hz, 1H), 5.86 (dd, *J* = 10.4, 7.9 Hz, 1H), 5.61 (dd, *J* = 10.4, 3.5 Hz, 1H), 4.90 (d, *J* = 10.9 Hz, 1H), 4.80–4.65 (m, 4H), 4.62–4.54 (m, 2H), 4.51 (d, *J* = 3.5 Hz, 1H), 4.44–4.35 (m, 2H), 4.30–4.18 (m, 2H),

3.91 (t, J = 9.2 Hz, 1H), 3.82–3.72 (m, 2H), 3.45–3.34 (m, 2H), 3.21 (s, 3H); ^{13}C NMR (101 MHz, $CDCl_3$) δ 166.05, 165.66, 165.61, 165.18, 138.82, 138.26, 138.18, 133.63, 133.34, 133.19, 130.08, 129.79, 129.73, 129.44, 129.30, 129.04, 128.73, 128.65, 128.51, 128.48, 128.44, 128.41, 128.32, 128.12, 127.93, 127.90, 127.74, 127.59, 127.54, 102.05, 97.93, 81.95, 79.89, 75.57, 74.74, 73.39, 71.67, 71.40, 69.79, 69.64, 68.73, 68.14, 61.94, 55.05; HRMS (ESI) calcd for $C_{62}H_{58}O_{15}Na$ $[M+Na]^+$ 1065.3668, found 1065.3693.

Compound 3n

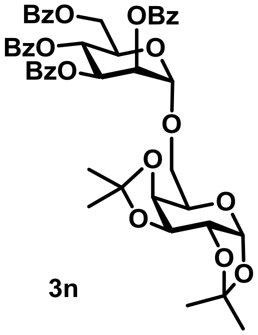

3n

Glycosylation of **1d** (132.1 mg, 0.16mmol) with **2a** (35.7 mg, 0.14 mmol) according to **General procedure B** afforded **3n**[39] (110.5 mg, 96%) as a white solid: $[\alpha]_D^{25}$ = -71.6 (*c* 0.18, $CHCl_3$); ^1H NMR (400 MHz, $CDCl_3$): δ 8.13 (d, J = 7.7 Hz, 2H), 8.06 (d, J = 7.7 Hz, 2H), 7.96 (d, J = 7.7 Hz, 2H), 7.84 (d, J = 7.7 Hz, 2H), 7.58 (q, J = 7.2 Hz, 2H), 7.50 (t, J = 7.4 Hz, 1H), 7.44–7.34 (m, 7H), 7.26 (t, J = 7.7 Hz, 2H), 6.15 (t, J = 10.1 Hz, 1H), 5.93 (dd, J = 10.1, 3.3 Hz, 1H), 5.80–5.74 (m, 1H), 5.57 (d, J = 5.0 Hz, 1H), 5.18 (d, J = 1.8 Hz, 1H), 4.73–4.65 (m, 2H), 4.61 (dt, J = 10.2, 3.2 Hz, 1H), 4.51 (dd, J = 12.2, 3.9 Hz, 1H), 4.38–4.33 (m, 2H), 4.13 (td, J = 6.1, 1.8 Hz, 1H), 3.98 (dd, J = 10.5, 6.4 Hz, 1H), 3.90 (dd, J = 10.5, 5.9 Hz, 1H), 1.64 (s, 3H), 1.44 (s, 3H), 1.36 (s, 6H); ^{13}C NMR (101 MHz, $CDCl_3$) δ 166.23, 165.54, 165.42, 165.35, 133.44, 133.39, 133.16, 132.99, 129.98, 129.85, 129.81, 129.79, 129.73, 129.38, 129.14, 129.10, 128.59, 128.45, 128.42, 128.30, 109.49, 108.78, 97.88, 96.36, 70.95, 70.67, 70.38, 70.25, 68.83, 67.56, 66.83, 66.75, 62.88, 26.25, 25.99, 25.03, 24.44; HRMS (ESI) calcd for $C_{46}H_{46}O_{15}Na$ $[M+Na]^+$ 861.2729, found 861.2757.

Compound 3o

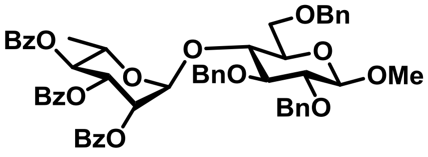

3o

Glycosylation of **1e** (100.2 mg, 0.15mmol) with **2b** (56.8 mg, 0.12 mmol) according to **General procedure B** afforded **3o**[40] (105.4 mg, 97%) as a white solid: $[\alpha]_D^{25}$ = 39.1 (*c* 0.17, $CHCl_3$); ^1H NMR (400 MHz, $CDCl_3$): δ 7.98 (d, *J* = 7.7 Hz, 2H), 7.78 (d, *J* = 8.8 Hz, 2H), 7.76 (d, *J* = 8.7 Hz, 2H), 7.50 (d, *J* = 7.2 Hz, 1H), 7.44–7.10 (m, 19H), 7.08–7.01 (m, 4H), 5.70 (dd, *J* = 10.2, 3.4 Hz, 1H), 5.55–5.47 (m, 2H), 5.25–5.17 (m, 1H), 5.08 (d, *J* = 11.1 Hz, 1H), 4.85 (d, *J* = 10.9 Hz, 1H), 4.74 (d, *J* = 11.2 Hz, 1H), 4.61 (d, *J* = 11.0 Hz, 1H), 4.56–4.48 (m, 2H), 4.34–4.25 (m, 2H), 3.97 (t, *J* = 9.4 Hz, 1H), 3.80 (dd, *J* = 11.2, 3.3 Hz, 1H), 3.74 (dd, *J* = 11.3, 2.0 Hz, 1H), 3.59 (t, *J* = 9.1 Hz, 1H), 3.50 (s, 3H), 3.47 (t, *J* = 8.4 Hz, 1H), 3.44–3.39 (m, 1H), 0.81 (d, *J* = 6.1 Hz, 3H); ^{13}C NMR (101 MHz, $CDCl_3$) δ 165.77, 138.48, 138.34, 138.04, 133.51, 133.28, 133.22, 129.94, 129.73, 129.69, 129.44, 129.24, 128.60, 128.42, 128.36, 128.23, 128.20, 127.74, 127.72, 127.43, 127.36, 104.92, 97.07, 82.56, 82.44, 75.35, 74.89, 74.72, 74.70, 73.19, 71.80, 71.21, 70.10, 68.26, 67.09, 57.12, 17.21; HRMS (ESI) calcd for $C_{55}H_{54}O_{13}Na$ $[M+Na]^+$ 945.3457, found 945.3479.

Compound 3p

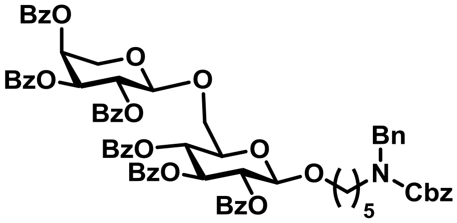

3p

Glycosylation of **1i** (65.0 mg, 0.10mmol) with **2m** (65.0 mg, 0.08 mmol) according to **General procedure B** afforded **3p** (100.2 mg, 99%) as a colorless syrup: $[\alpha]_D^{25}$ = 35.7 (*c* 0.18, $CHCl_3$); ^1H NMR (400 MHz, $CDCl_3$): δ 8.00 (t, *J* = 8.1 Hz, 4H), 7.96–7.85 (m, 6H), 7.78 (d, *J* = 7.6 Hz, 2H), 7.54 (t, *J* = 7.3 Hz, 1H), 7.47 (t, *J* = 7.2 Hz, 3H), 7.44–7.17 (m, 23H), 7.15–7.08 (m, 1H), 5.83 (t, *J* = 9.6 Hz, 1H), 5.73 (dd, *J* = 8.2, 6.4

Hz, 1H), 5.67 (s, 1H), 5.62 (dd, *J* = 8.4, 3.1 Hz, 1H), 5.38 (t, *J* = 9.3 Hz, 2H), 5.15 (d, J = 5.2 Hz, 2H), 4.84 (d, *J* = 5.9 Hz, 1H), 4.60 (dd, *J* = 19.5, 7.1 Hz, 1H), 4.42–4.32 (m, 2H), 4.25 (dd, *J* = 12.8, 4.0 Hz, 1H), 4.10 (d, *J* = 10.9 Hz, 1H), 4.00 (t, *J* = 7.9 Hz, 1H), 3.89–3.78 (m, 2H), 3.59–3.48 (m, 1H), 3.21–3.08 (m, 1H), 3.02–2.84 (m, 2H), 1.34–1.21 (m, 4H), 1.10–0.90 (m, 2H); ^{13}C NMR (101 MHz, $CDCl_3$) δ 165.81, 165.68, 165.58, 165.42, 165.21, 164.99, 156.68, 156.11, 138.05, 138.01, 136.96, 136.92, 136.85, 133.55, 133.44, 133.24, 133.21, 129.92, 129.89, 129.82, 129.77, 129.70, 129.42, 129.37, 129.34, 129.13, 128.88, 128.78, 128.57, 128.53, 128.50, 128.48, 128.37, 128.31, 127.88, 127.78, 127.32, 127.30, 127.16, 101.06, 100.94, 73.92, 72.95, 71.98, 70.42, 69.92, 69.84, 69.67, 68.41, 68.36, 67.15, 62.44, 50.60, 50.28, 47.16, 46.18, 29.76, 28.94, 27.75, 27.37, 23.08; HRMS (ESI) calcd for $C_{73}H_{67}NO_{18}Na$ $[M+Na]^+$ 1268.4250, found 1268.4220.

Compound 3q

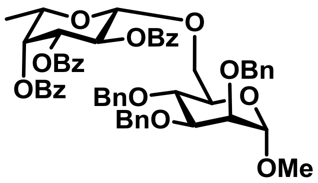

3q

Glycosylation of **1j** (74.8 mg, 0.12mmol) with **2e** (42.4 mg, 0.10 mmol) according to **General procedure B** afforded **3q** (73.5 mg, 88%) as a white solid: $[\alpha]_D^{25}$ = -171.0 (*c* 0.12, $CHCl_3$); ^{1}H NMR (400 MHz, $CDCl_3$): δ 8.09 (d, *J* = 7.2 Hz, 2H), 7.94 (d, *J* = 7.3 Hz, 2H), 7.79 (d, *J* = 7.5 Hz, 2H), 7.57 (t, *J* = 7.4 Hz, 1H), 7.43 (t, *J* = 7.7 Hz, 2H), 7.41–7.19 (m, 21H), 5.81 (dd, *J* = 10.5, 7.9 Hz, 1H), 5.73 (d, *J* = 3.4 Hz, 1H), 5.50 (dd, *J* = 10.5, 3.5 Hz, 1H), 5.08 (d, *J* = 8.0 Hz, 1H), 4.68 (d, *J* = 9.2 Hz, 3H), 4.62 (d, *J* = 10.8 Hz, 1H), 4.58–4.52 (m, 2H), 4.47 (d, *J* = 10.8 Hz, 1H), 4.11 (dd, *J* = 12.0, 5.6 Hz, 1H), 4.06–3.99 (m, 2H), 3.93 (t, *J* = 9.6 Hz, 1H), 3.81 (dd, *J* = 9.4, 3.2 Hz, 1H), 3.75 (t, *J* = 2.4 Hz, 1H), 3.66 (dd, *J* = 10.1, 5.3 Hz, 1H), 3.10 (s, 3H), 1.34 (d, *J* = 6.3 Hz, 3H); ^{13}C NMR (101 MHz, $CDCl_3$) δ 166.12, 165.71, 165.39, 138.64, 138.57, 138.31, 133.40, 133.16, 132.94, 130.06, 129.89, 129.77, 129.55, 129.31, 129.04,

128.55, 128.44, 128.35, 128.33, 128.28, 127.93, 127.81, 127.76, 127.62, 127.53, 100.49, 98.70, 80.10, 75.01, 74.72, 74.64, 72.73, 72.41, 72.04, 71.98, 71.23, 70.03, 69.71, 67.43, 54.51, 16.36; HRMS (ESI) calcd for $C_{55}H_{54}O_{13}Na$ $[M+Na]^+$ 945.3457, found 945.3479.

Compound 3r

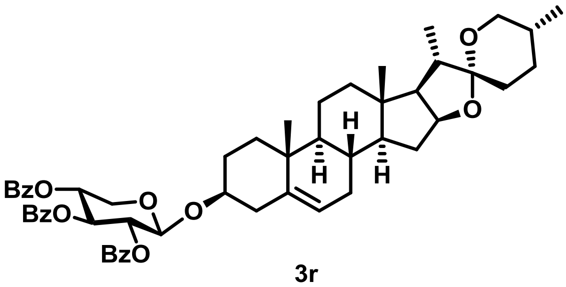

3r

Glycosylation of **1g** (100.0 mg, 0.15mmol) with **2n** (51.7 mg, 0.12 mmol) according to **General procedure B** afforded **3r** (98.7 mg, 92%) as a white solid: $[\alpha]_D^{25}$ = -53.3 (*c* 0.16, $CHCl_3$); ^1H NMR (400 MHz, $CDCl_3$): δ 7.99 (dd, *J* = 7.9, 3.0 Hz, 6H), 7.56–7.47 (m, 3H), 7.40–7.32 (m, 6H), 5.77 (t, *J* = 7.4 Hz, 1H), 5.35 (dd, *J* = 7.5, 5.6 Hz, 1H), 5.33–5.26 (m, 2H), 4.96 (d, *J* = 5.6 Hz, 1H), 4.44 (dd, *J* = 12.1, 4.5 Hz, 1H), 4.40 (t, *J* = 8.0 Hz, 1H), 3.69 (dd, *J* = 12.1, 7.3 Hz, 1H), 3.58 (dt, *J* = 11.4, 6.3 Hz, 1H), 3.47 (dd, *J* = 11.6, 5.2 Hz, 1H), 3.37 (t, *J* = 10.9 Hz, 1H), 2.29 (dd, *J* = 13.4, 2.9 Hz, 1H), 2.15 (dd, *J* = 13.4, 2.9 Hz, 1H), 2.04–0.87 (m, 22H), 0.99–0.95 (m, 6H), 0.80–0.76 (m, 6H); ^{13}C NMR (101 MHz, $CDCl_3$) δ 165.56, 165.44, 165.20, 140.32, 133.36, 133.30, 133.22, 129.89, 129.81, 129.43, 129.24, 129.21, 128.42, 128.35, 121.76, 109.29, 98.63, 80.81, 78.76, 70.71, 70.53, 69.29, 66.85, 62.09, 61.37, 56.47, 50.05, 41.61, 40.26, 39.75, 38.61, 37.23, 36.86, 32.07, 31.85, 31.40, 30.30, 29.54, 28.81, 20.84, 19.36, 17.16, 16.29, 14.55; HRMS (ESI) calcd for $C_{53}H_{62}O_{10}Na$ $[M+Na]^+$ 881.4235, found 881.4239.

Compound 3s

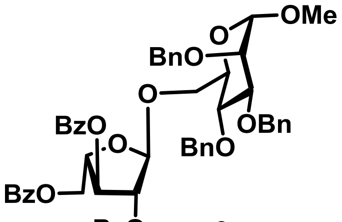

Glycosylation of **1k** (50.0 mg, 0.07 mmol) with **2e** (29.0 mg, 0.06 mmol) according to **General procedure B** to afford **3s** (55.0 mg, 98%) as a colorless syrup: $[\alpha]_D^{25}$ = 17.4 (c 0.13, $CHCl_3$); ^1H NMR (400 MHz, $CDCl_3$) δ 8.06–7.87 (m, 6H), 7.49–7.44 (m, 3H), 7.35–7.12 (m, 21H), 5.47 (d, *J* = 3.2 Hz, 2H), 5.29 (s, 1H), 4.84 (d, *J* = 10.9 Hz, 1H), 4.73–4.68 (m, 1H), 4.68–4.59 (m, 2H), 4.59–4.48 (m, 5H), 3.98 (d, *J* = 11.0 Hz, 1H), 3.88 (d, *J* = 9.5 Hz, 1H), 3.81 (dd, *J* = 8.7, 3.3 Hz, 2H), 3.73–3.65 (m, 2H), 3.20 (s, 3H); ^{13}C NMR (101 MHz, $CDCl_3$) δ 166.27, 165.85, 165.31, 138.48, 138.43, 138.17, 133.42, 133.39, 133.01, 130.02, 129.88, 129.81, 129.77, 129.16, 129.04, 128.50, 128.43, 128.38, 128.37, 128.32, 128.30, 128.24, 127.96, 127.84, 127.70, 127.68, 127.66, 127.59, 105.86, 98.94, 82.05, 80.81, 80.24, 77.85, 75.17, 74.92, 74.56, 72.72, 72.13, 71.61, 67.08, 63.76, 54.67; HRMS (ESI) calcd for $C_{54}H_{52}O_{13}Na$ $[M+Na]^+$ 931.3300, found 931.3322.

Compound 3t

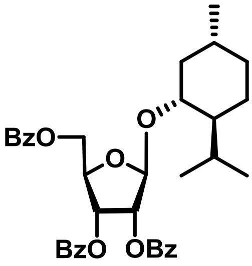

3t

Glycosylation of **1l** (43.0 mg, 0.06 mmol) with **2i** (8.5 mg, 0.05 mmol) according to **General procedure B** to afford **3t**[37] (31.0 mg, 98%) as a white solid: $[\alpha]_D^{25}$ = -26.9 (c 0.25, $CHCl_3$); ^1H NMR (400 MHz, $CDCl_3$) δ 8.05–7.92 (m, 4H), 7.87–7.78 (m, 2H), 7.55–7.20 (m, 9H), 5.72 (dd, *J* = 7.1, 4.7 Hz, 1H), 5.54 (d, *J* = 4.7 Hz, 1H), 5.40 (s, 1H), 4.69–4.55 (m, 2H), 4.45 (dd, *J* = 11.5, 6.4 Hz, 1H), 3.46 (td, *J* = 10.6, 4.2 Hz, 1H), 2.19–2.01 (m, 2H), 1.60–1.54 (m, 3H), 1.36–1.11 (m, 2H), 0.93–0.87 (m, 2H),

0.85 (d, J = 6.4 Hz, 3H), 0.76 (d, J = 7.0 Hz, 3H), 0.70 (d, J = 6.9 Hz, 3H); ^{13}C NMR (101 MHz, $CDCl_3$) δ 166.20, 165.36, 165.34, 133.43, 133.34, 133.06, 129.80, 129.73, 129.67, 129.33, 129.01, 128.48, 128.34, 128.31, 102.47, 78.07, 76.93, 76.24, 73.09, 65.89, 47.74, 40.04, 34.35, 31.46, 25.23, 22.84, 22.30, 21.02, 15.63; HRMS (ESI) calcd for $C_{36}H_{40}O_8Na$ [M+Na]$^+$ 623.2615, found 623.2617.

Compound 3u

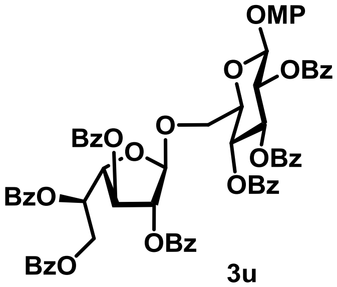

3u

Glycosylation of **1m** (57.9 mg, 0.07 mmol) with **2d** (36.0 mg, 0.06 mmol) according to **General procedure B** to afford **3u** (70.2 mg, 99%) as a white solid: $[\alpha]_D^{25}$ = -5.8 (c 0.16, $CHCl_3$); ^{1}H NMR (400 MHz, $CDCl_3$) δ 8.01 (d, J = 6.9 Hz, 2H), 7.97–7.81 (m, 8H), 7.75 (d, J = 6.0 Hz, 4H), 7.49–7.15 (m, 21H), 6.86 (d, J = 7.9 Hz, 2H), 6.57 (d, J = 7.9 Hz, 2H), 5.98–5.82 (m, 2H), 5.69–5.60 (m, 1H), 5.60–5.49 (m, 2H), 5.44 (s, 1H), 5.34 (s, 1H), 5.20 (d, J = 7.2 Hz, 1H), 4.67–4.46 (m, 3H), 4.17–4.05 (m, 1H), 3.96 (d, J = 10.9 Hz, 1H), 3.90–3.79 (m, 1H), 3.46 (s, 3H); ^{13}C NMR (151 MHz, $CDCl_3$) δ 166.17, 165.90, 165.83, 165.75, 165.31, 165.31, 165.17, 155.81, 151.09, 133.63, 133.59, 133.39, 133.28, 133.14, 130.12, 130.02, 129.99, 129.92, 129.82, 129.67, 129.52, 129.33, 129.14, 128.98, 128.91, 128.65, 128.57, 128.52, 128.45, 128.42, 118.91, 114.62, 106.30, 100.97, 81.94, 81.91, 77.62, 74.06, 73.03, 71.99, 70.39, 69.70, 66.29, 63.75, 55.47; HRMS (ESI) calcd for $C_{68}H_{56}O_{19}Na$ [M+Na]$^+$ 1199.3308, found 1199.3284.

Compound 3v

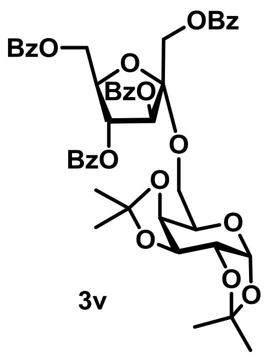

3v

Glycosylation of **1n** (111.0 mg, 0.14 mmol) with **2a** (30.0 mg, 0.12 mmol) according to **General procedure B** to afford **3v**[41] (96.1 mg, 99%) as a colorless syrup: $[\alpha]_D^{25}$ = -6.5 (c 0.27, $CHCl_3$); ^1H NMR (400 MHz, $CDCl_3$) δ 8.15 (d, *J* = 7.5 Hz, 2H), 8.02 (d, *J* = 7.5 Hz, 2H), 7.94 (d, *J* = 7.7 Hz, 4H), 7.58 (t, *J* = 7.3 Hz, 1H), 7.54 – 7.43 (m, 5H), 7.38 – 7.31 (m, 4H), 7.24 (t, *J* = 7.6 Hz, 2H), 5.92 (s, 1H), 5.56 (d, *J* = 4.4 Hz, 1H), 5.50 (d, *J* = 4.9 Hz, 1H), 4.97 (d, *J* = 12.2 Hz, 1H), 4.85 (dd, *J* = 11.5, 2.0 Hz, 1H), 4.77–4.66 (m, 2H), 4.53 (dd, *J* = 7.6, 1.7 Hz, 1H), 4.40 (d, *J* = 12.2 Hz, 1H), 4.30–4.25 (m, 2H), 3.99 (t, *J* = 5.3 Hz, 1H), 3.95–3.85 (m, 2H), 1.38 (s, 3H), 1.37 (s, 3H), 1.28 (s, 3H), 1.12 (s, 3H); ^{13}C NMR (151 MHz, $CDCl_3$) δ 166.25, 165.95, 165.70, 164.79, 133.54, 133.47, 133.13, 133.10, 130.24, 129.83, 129.80, 129.76, 129.53, 129.35, 129.09, 128.62, 128.46, 128.41, 128.37, 128.36, 109.35, 108.55, 107.34, 96.40, 81.52, 81.13, 78.99, 71.15, 70.65, 70.54, 66.96, 63.72, 60.66, 59.63, 26.05, 25.98, 25.02, 24.27; HRMS (ESI) calcd for $C_{46}H_{46}O_{15}Na$ $[M+Na]^+$ 861.2729, found 861.2749.

Compound 3w

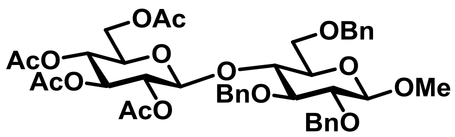

3w

Glycosylation of **1b** (50.0 mg, 0.09 mmol) with **2b** (34.9 mg, 0.08 mmol) according to **General procedure B** to afford **3w** (58.7 mg, 98%) as a white solid: $[\alpha]_D^{25}$ = -19.8 (c 0.10, $CHCl_3$); ^1H NMR (400 MHz, $CDCl_3$) δ 7.42–7.36 (m, 4H), 7.35–7.31 (m, 3H), 7.30–7.22 (m, 8H), 5.06–4.99 (m, 2H), 4.96 (d, *J* = 11.2 Hz, 1H), 4.91 (t, *J* = 8.5 Hz,

1H), 4.83 (d, J = 11.0 Hz, 1H), 4.76 (dd, J = 11.7, 7.6 Hz, 2H), 4.68–4.63 (m, 2H), 4.50 (d, J = 12.1 Hz, 1H), 4.27 (d, J = 7.7 Hz, 1H), 4.12 (dd, J = 12.5, 4.1 Hz, 1H), 3.94 (t, J = 9.3 Hz, 1H), 3.87 (dd, J = 12.4, 2.3 Hz, 1H), 3.79–3.71 (m, 2H), 3.61–3.51 (m, 4H), 3.38 (d, J = 8.0 Hz, 1H), 3.34 (dt, J = 9.8, 3.0 Hz, 1H), 3.29 (qd, J = 5.8, 3.1 Hz, 1H), 1.99 (s, 3H), 1.98 (s, 3H), 1.96 (s, 3H), 1.94 (s, 3H); ^{13}C NMR (151 MHz, $CDCl_3$) δ 170.65, 170.25, 169.37, 169.15, 139.07, 138.46, 137.94, 128.57, 128.24, 128.10, 127.99, 127.53, 127.30, 127.26, 104.65, 100.00, 82.59, 81.71, 75.06, 74.77, 74.61, 73.64, 73.15, 71.94, 71.57, 68.01, 67.75, 61.57, 57.09, 20.69, 20.64, 20.63, 20.58; HRMS (ESI) calcd for $C_{42}H_{50}O_{15}Na$ $[M+Na]^+$ 817.3042, found 817.3041.

Compound 3x

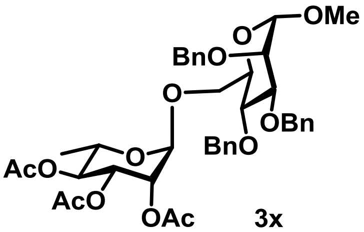

3x

Glycosylation of **1f** (29.0 mg, 0.06 mmol) with **2e** (37.2 mg, 0.07 mmol) according to **General procedure B** to afford **3x** (43.0 mg, 96%) as a colorless syrup: $[\alpha]_D^{25}$ = -23.7 (c 0.16, $CHCl_3$); ^{1}H NMR (400 MHz, $CDCl_3$) δ 7.44–7.24 (m, 15H), 5.35–5.25 (m, 2H), 5.07 (d, J = 9.7 Hz, 1H), 4.96 (d, J = 11.1 Hz, 1H), 4.81 (s, 1H), 4.78–4.67 (m, 3H), 4.64–4.54 (m, 3H), 3.97–3.91 (m, 3H), 3.83–3.71 (m, 3H), 3.69–3.61 (m, 1H), 3.33 (s, 3H), 2.02 (s, 3H), 2.18 (s, 3H), 1.97 (s, J = 15.2 Hz, 6H), 1.18 (d, J = 6.2 Hz, 3H); ^{13}C NMR (101 MHz, $CDCl_3$) δ 170.07, 170.05, 169.89, 138.40, 138.37, 138.25, 128.38, 128.37, 127.87, 127.84, 127.67, 127.64, 127.60, 98.81, 97.76, 80.25, 75.11, 75.02, 74.42, 72.68, 72.08, 71.65, 71.16, 69.87, 69.16, 67.35, 66.32, 54.80, 20.95, 20.82, 20.74, 17.37; HRMS (ESI) calcd for $C_{40}H_{48}O_{13}Na$ $[M+Na]^+$ 759.2987, found 759.3010.

Compound 3y

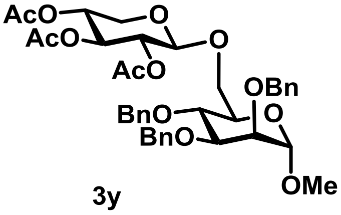

3y

Glycosylation of **1h** (100.0 mg, 0.21 mmol) with **2e** (80.0 mg, 0.17 mmol) according to **General procedure B** to afford **3y** (123.1 mg, 99%) as a colorless syrup: $[\alpha]_D^{25}$ = -24.4 (c 0.14, $CHCl_3$); ^{1}H NMR (400 MHz, $CDCl_3$) δ 7.38–7.24 (m, 15H), 5.11 (t, *J* = 8.1 Hz, 1H), 4.97–4.87 (m, 3H), 4.73–4.68 (m, 3H), 4.62–4.58 (m, 2H), 4.57 (d, *J* = 13.1 Hz, 1H), 4.54 (d, *J* = 6.4 Hz, 0H), 4.13 (dd, *J* = 11.9, 4.9 Hz, 1H), 4.04 (dd, *J* = 9.5, 4.0 Hz, 1H), 3.87 (dd, *J* = 9.1, 3.0 Hz, 1H), 3.83 (d, *J* = 9.2 Hz, 1H), 3.79–3.76 (m, 1H), 3.75–3.69 (m, 2H), 3.36–3.31 (m, 1H), 3.30 (s, 3H), 2.04 (s, 3H), 2.00 (s, 6H); ^{13}C NMR (101 MHz, $CDCl_3$) δ 170.13, 169.84, 169.26, 138.44, 138.20, 128.44, 128.38, 127.91, 127.87, 127.72, 127.70, 127.58, 100.55, 98.92, 80.23, 74.99, 74.76, 74.56, 72.80, 72.01, 71.42, 71.12, 70.52, 68.78, 68.48, 61.62, 54.68, 20.81, 20.72; HRMS (ESI) calcd for $C_{39}H_{46}O_{13}Na$ $[M+Na]^+$ 745.2831, found 745.2834.

Compound 3z

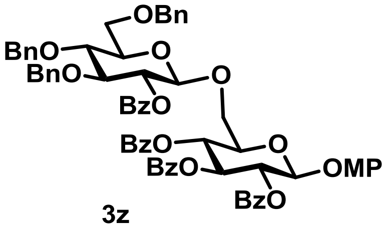

3z

Glycosylation of **1o** (54.6 mg, 0.07 mmol) with **2d** (35.8 mg, 0.06 mmol) according to **General procedure B** to afford **3z** (67.2 mg, 99%) as a colorless syrup: $[\alpha]_D^{25}$ = -1.3 (c 0.10, $CHCl_3$); ^{1}H NMR (400 MHz, $CDCl_3$) δ 7.93 (d, *J* = 8.7 Hz, 2H), 7.91 (d, *J* = 7.8 Hz, 2H), 7.87 (d, *J* = 7.8 Hz, 2H), 7.80 (d, *J* = 7.8 Hz, 2H), 7.55–7.46 (m, 3H), 7.42 (t, *J* = 7.4 Hz, 1H), 7.39–7.25 (m, 17H), 7.19–7.11 (m, 6H), 6.90 (d, *J* = 8.6 Hz, 2H), 6.74 (d, *J* = 8.8 Hz, 2H), 5.83 (t, *J* = 9.6 Hz, 1H), 5.63 (t, *J* = 8.8 Hz, 1H), 5.39 (t, *J* = 9.7 Hz, 1H), 5.26 (t, *J* = 8.1 Hz, 1H), 5.11 (d, *J* = 7.8 Hz, 1H), 4.78 (d, *J* = 10.8 Hz, 1H), 4.71 (d, *J* = 11.0 Hz, 1H), 4.67 (d, *J* = 7.9 Hz, 1H), 4.62 (d, *J* = 10.9 Hz, 1H),

4.54 (d, *J* = 10.7 Hz, 2H), 4.43 (d, *J* = 12.2 Hz, 1H), 4.08 (t, *J* = 8.6 Hz, 1H), 3.97 (d, *J* = 11.0 Hz, 1H), 3.91 (dd, *J* = 11.6, 7.7 Hz, 1H), 3.77–3.71 (m, 2H), 3.69 (s, 3H), 3.68–3.62 (m, 2H), 3.47 (dt, *J* = 9.6, 3.2 Hz, 1H); ^{13}C NMR (101 MHz, $CDCl_3$) δ 165.69, 165.40, 165.24, 165.07, 155.76, 151.08, 138.00, 137.92, 137.79, 133.49, 133.27, 133.22, 133.00, 129.93, 129.88, 129.79, 129.76, 129.71, 129.20, 128.81, 128.70, 128.43, 128.40, 128.33, 128.28, 128.25, 128.00, 127.93, 127.83, 127.80, 127.66, 127.63, 119.19, 114.63, 100.97, 100.75, 83.03, 77.81, 75.22, 75.19, 75.01, 74.80, 73.61, 73.53, 72.84, 71.81, 69.61, 68.51, 67.58, 55.58; HRMS (ESI) calcd for $C_{68}H_{62}O_{16}Na$ $[M+Na]^+$ 1157.3930, found 1157.3972.

Compound 3aa

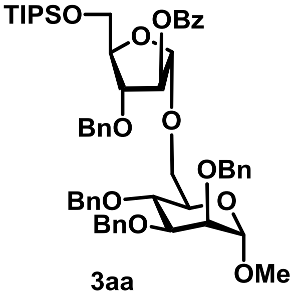

3aa

Glycosylation of **1p** (69.4 mg, 0.10 mmol) with **2e** (38.0 mg, 0.08 mmol) according to **General procedure B** to afford **3aa** (69.5 mg, 90%) as a colorless syrup: $[\alpha]_D^{25}$ = 38.0 (c 0.13, $CHCl_3$); ^{1}H NMR (400 MHz, $CDCl_3$) δ 7.94 (d, *J* = 7.5 Hz, 2H), 7.49 (t, *J* = 7.1 Hz, 1H), 7.35 (t, *J* = 7.5 Hz, 2H), 7.29–7.10 (m, 20H), 5.47 (s, 1H), 5.27 (s, 1H), 4.75 (t, *J* = 11.9 Hz, 2H), 4.69–4.63 (m, 3H), 4.59 (d, *J* = 11.1 Hz, 1H), 4.54–4.46 (m, 3H), 4.17–4.12 (m, 1H), 4.07–4.00 (m, 2H), 3.97 (d, *J* = 9.6 Hz, 1H), 3.78 (d, *J* = 9.5 Hz, 1H), 3.75–3.63 (m, 5H), 3.20 (s, 3H), 0.95–0.87 (m, 21H); ^{13}C NMR (151 MHz, $CDCl_3$) δ 165.42, 138.99, 138.76, 138.53, 138.11, 133.36, 129.88, 129.81, 128.47, 128.42, 128.41, 128.36, 128.10, 127.95, 127.88, 127.71, 127.65, 127.58, 127.49, 106.38, 99.16, 84.48, 83.48, 81.84, 80.43, 75.08, 74.84, 74.62, 72.65, 72.32, 72.25, 71.34, 66.10, 63.02, 54.81, 18.04, 18.03, 12.02; HRMS (ESI) calcd for $C_{56}H_{70}O_{11}SiNa$ $[M+Na]^+$ 969.4580, found 969.4568.

Compound 3ab

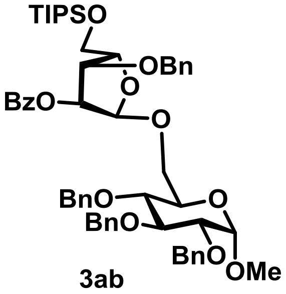

Glycosylation of **1q** (54.8 mg, 0.08 mmol) with **2c** (30 mg, 0.06 mmol) according to **General procedure B** to afford **3ab** (53.3 mg, 87%) as a colorless syrup: $[\alpha]_D^{25}$ = 38.0 (c 0.13, $CHCl_3$); ^1H NMR (400 MHz, $CDCl_3$) δ 7.91 (d, *J* = 7.4 Hz, 2H), 7.49 (t, *J* = 7.0 Hz, 1H), 7.34 (t, *J* = 7.4 Hz, 2H), 7.31–7.09 (m, 20H), 5.34 (s, 1H), 5.04 (s, 1H), 4.90 (d, *J* = 10.8 Hz, 1H), 4.80 (d, *J* = 11.0 Hz, 1H), 4.74 (d, *J* = 10.9 Hz, 1H), 4.72–4.63 (m, 2H), 4.62–4.47 (m, 4H), 4.19–4.13 (m, 1H), 4.09 (d, *J* = 4.2 Hz, 1H), 3.97–3.85 (m, 2H), 3.78 (d, *J* = 3.4 Hz, 2H), 3.75–3.69 (m, 1H), 3.56 (m , 1H), 3.47 (t, *J* = 9.3 Hz, 2H), 3.26 (s, 3H), 0.95 (s, 21H); ^{13}C NMR (101 MHz, $CDCl_3$) δ 165.46, 138.97, 138.53, 138.34, 137.97, 133.41, 129.87, 129.67, 128.55, 128.48, 128.42, 128.20, 128.07, 127.96, 127.91, 127.90, 127.74, 127.66, 106.54, 97.99, 83.62, 83.00, 82.27, 80.15, 78.06, 75.82, 75.06, 73.47, 72.35, 70.18, 66.18, 62.82, 55.17, 18.07, 18.06, 12.05; HRMS (ESI) calcd for $C_{56}H_{70}O_{11}SiNa$ $[M+Na]^+$ 969.4580, found 969.4578.

Compound 3ac

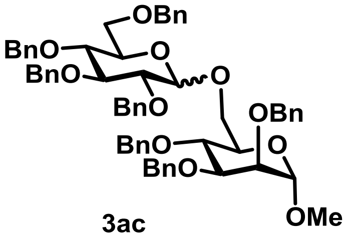

Glycosylation of **1r** (120.0 mg, 0.16 mmol) with **2e** (62.0 mg, 0.13 mmol) according to **General procedure B** to afford **3ac**[42] (130.8 mg, 99%, α/β = 1:1) as a white solid; α and β anomers: ^1H NMR (400 MHz, $CDCl_3$) δ 7.38–7.10 (m, 70H), 5.11 (d, *J* = 3.4 Hz, 1H), 5.06 (d, *J* = 10.9 Hz, 1H), 4.95 (d, *J* = 7.4 Hz, 1H), 4.93 (d, *J* = 4.9 Hz, 1H), 4.91 (d, *J* = 8.3 Hz, 1H), 4.84 (d, *J* = 5.2 Hz, 1H), 4.81 (d, *J* = 6.4 Hz, 2H), 4.76 (d, *J*

= 11.8 Hz, 3H), 4.69 (d, *J* = 9.7 Hz, 5H), 4.67–4.57 (m, 9H), 4.54 (d, *J* = 10.5 Hz, 3H), 4.48 (d, *J* = 9.1 Hz, 1H), 4.45 (d, *J* = 3.4 Hz, 1H), 4.41 (d, *J* = 7.3 Hz, 1H), 4.27 (d, *J* = 10.5 Hz, 1H), 4.04–3.97 (m, 2H), 3.95 (d, *J* = 9.4 Hz, 1H), 3.91–3.44 (m, 21H), 3.27 (s, 3H), 3.25 (s, 3H); ^{13}C NMR (101 MHz, $CDCl_3$) δ 138.97, 138.82, 138.73, 138.68, 138.66, 138.62, 138.56, 138.35, 138.33, 138.27, 138.11, 128.46, 128.44, 128.41, 128.40, 128.37, 128.35, 128.30, 128.23, 128.09, 128.03, 128.00, 127.99, 127.96, 127.93, 127.87, 127.80, 127.76, 127.70, 127.69, 127.65, 127.63, 127.59, 127.53, 127.48, 104.22, 99.03, 98.98, 96.61, 84.76, 82.25, 81.74, 80.40, 80.31, 80.16, 77.98, 77.73, 75.80, 75.52, 75.13, 75.09, 75.02, 74.95, 74.87, 74.64, 73.55, 73.48, 72.88, 72.84, 72.62, 72.21, 72.08, 71.81, 71.47, 70.19, 69.14, 68.58, 66.04, 54.84, 54.80; HRMS (ESI) calcd for $C_{62}H_{66}O_{11}Na$ $[M+Na]^+$ 1009.4497, found 1009.4507.

Compound 3ad

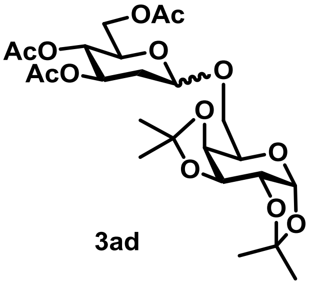

3ad

Glycosylation of **1s** (53.0 mg, 0.11 mmol) with **2a** (23.0 mg, 0.09 mmol) according to **General procedure B** to afford **3ad**[31] (40.0 mg, 86%, α/β = 3.3:1) as a colorless syrup; α and β anomers: ^{1}H NMR (400 MHz, $CDCl_3$) δ 5.51 (d, *J* = 5.0 Hz, 1H), 5.31 (ddd, *J* = 11.5, 9.3, 5.4 Hz, 1H), 5.05–4.95 (m, 2H), 4.62 (dd, *J* = 7.9, 2.5 Hz, 1H), 4.36–4.29 (m, 3H), 4.25 (dd, *J* = 7.9, 2.0 Hz, 1H), 4.09–3.99 (m, 2H), 3.99–3.92 (m, 1H), 3.75 (dd, *J* = 10.1, 6.3 Hz, 1H), 3.66 (dd, *J* = 10.3, 6.8 Hz, 1H), 2.28 (dd, *J* = 13.0, 5.4 Hz, 1H), 2.10 (s, 2H), 2.09 (s, 1H), 2.04 (s, 2H), 2.03 (s, 1H), 2.02 (s, 1H), 2.01 (s, 3H), 1.84 (dd, *J* = 12.3, 3.7 Hz, 1H), 1.81–1.78 (m, 1H), 1.56 (s, 3H), 1.53 (s, 1H), 1.45 (s, 1H), 1.43 (s, 3H), 1.34 (d, *J* = 2.1 Hz, 7H), 1.26 (s, 1H); ^{13}C NMR (101 MHz, $CDCl_3$) δ 170.82, 170.24, 169.95, 169.82, 109.41, 109.30, 108.66, 100.14, 97.08, 96.29, 71.93, 71.61, 71.36, 70.91, 70.69, 70.60, 70.57, 70.36, 69.25, 69.17,

69.12, 68.05, 67.88, 67.72, 66.26, 66.18, 62.46, 62.35, 62.26, 36.07, 34.95, 26.12, 26.03, 25.97, 24.93, 24.51, 24.37, 24.29, 20.99, 20.90, 20.79, 20.75, 20.73; HRMS (ESI) calcd for $C_{24}H_{36}O_{13}Na$ $[M+Na]^+$ 555.2048, found 555.2048.

Compound 6a

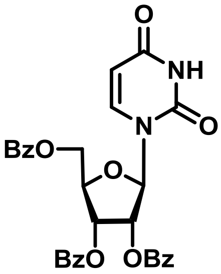

6a

Glycosylation of **1l** (40 mg, 0.06 mmol) with **5a** (13.5 mg, 0.12 mmol) according to **General procedure C** afforded **6a**[27] (31 mg, 93%) as a white solid: $[\alpha]_D^{25}$ = -56.37 (*c* 0.15, CHCl3); ^{1}H NMR (400 MHz, $CDCl_3$) δ 9.01 (s, 1H), 8.10 (d, *J* = 7.8 Hz, 2H), 7.98 (d, *J* = 7.8 Hz, 2H), 7.94 (d, *J* = 7.8 Hz, 2H), 7.54 (m, 5H), 7.38 (m, 5H), 6.32 (d, *J* = 5.5 Hz, 1H), 5.89 (t, *J* = 5.3 Hz, 1H), 5.76 (t, *J* = 5.8 Hz, 1H), 5.62 (dd, *J* = 8.2, 2.0 Hz, 1H), 4.84 (dd, *J* = 12.1, 2.7 Hz, 1H), 4.72 (dd, *J* = 6.9, 3.9 Hz, 1H), 4.67 (dd, *J* = 12.2, 3.9 Hz, 1H); ^{13}C NMR (101 MHz, $CDCl_3$) δ 166.06, 165.34, 165.30, 162.69, 150.01, 139.62, 133.85, 133.79, 133.69, 129.93, 129.84, 129.64, 129.20, 128.79, 128.57, 128.33, 103.41, 88.10, 80.55, 73.75, 71.15, 63.73; HRMS (ESI) calcd for $C_{30}H_{24}N_2O_9Na$ $[M+Na]^+$ 579.1374, found 579.1376.

Compound 6b

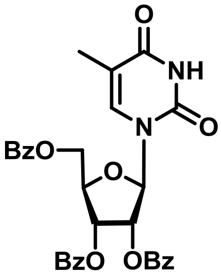

6b

Glycosylation of **1l** (40 mg, 0.06 mmol) with **5b** (15 mg, 0.12 mmol) according to **General procedure C** afforded **6b**[27,43] (28 mg, 85%) as a white foam: $[\alpha]_D^{25}$ = -59.58 (*c* 0.20, $CHCl_3$); ^{1}H NMR (400 MHz, $CDCl_3$) δ 8.58 (s, 1H), 8.15 (d, *J* = 7.4 Hz, 2H),

7.99 (d, *J* = 7.7 Hz, 2H), 7.95 (d, *J* = 7.7 Hz, 2H), 7.66–7.48 (m, 5H), 7.39 (m, 4H), 7.16 (s, 1H), 6.43 (d, *J* = 6.4 Hz, 1H), 5.92 (m, 1H), 5.76 (t, *J* = 6.2 Hz, 1H), 4.89 (dd, *J* = 12.1, 2.6 Hz, 1H), 4.74–4.61 (m, 2H), 1.59 (s, 3H); ^{13}C NMR (101 MHz, $CDCl_3$) δ 166.00, 165.42, 165.34, 163.19, 150.23, 134.81, 133.82, 133.79, 133.74, 129.94, 129.84, 129.65, 129.20, 128.89, 128.62, 128.58, 128.55, 128.34, 112.21, 86.89, 80.62, 73.36, 71.44, 63.94, 12.11; HRMS (ESI) calcd for $C_{31}H_{26}N_2O_9Na$ $[M+Na]^+$ 593.1531, found 593.1535.

Compound 6c

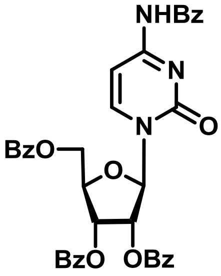

6c

Glycosylation of **1l** (40 mg, 0.06 mmol) with **5c** (26 mg, 0.12 mmol) according to **General procedure C** afforded **6c**[27] (38 mg, 96%) as a white solid: $[\alpha]_D^{25}$ = -42.47 (*c* 0.16, $CHCl_3$); ^{1}H NMR (400 MHz, $CDCl_3$) δ 8.83 (s, 1H), 8.11 (d, *J* = 7.7 Hz, 2H), 8.01–7.87 (m, 7H), 7.66–7.47 (m, 9H), 7.36 (t, *J* = 7.6 Hz, 4H), 6.46 (d, *J* = 4.4 Hz, 1H), 5.96- 5.79 (m, 2H), 4.92–4.68 (m, 3H); ^{13}C NMR (101 MHz, $CDCl_3$) δ 166.13, 165.28, 165.21, 133.67, 133.25, 129.97, 129.84, 129.67, 129.23, 129.03, 128.78, 128.62, 128.59, 128.49, 128.48, 89.55, 80.64, 74.75, 70.94, 63.60; HRMS (ESI) calcd for $C_{37}H_{29}N_3O_9Na$ $[M+Na]^+$ 682.1796, found 682.1796.

Compound 6d

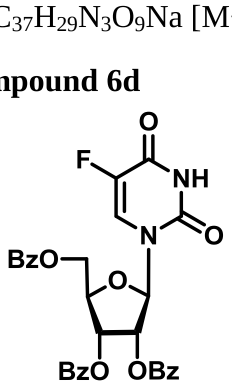

6d

Glycosylation of **1l** (40 mg, 0.06 mmol) with **5d** (16 mg, 0.12 mmol) according to **General procedure C** afforded **6d**[44] (33 mg, 96%) as a white solid: $[\alpha]_D^{25}$ = -56.64 (*c* 0.13, $CHCl_3$); ^1H NMR (400 MHz, DMSO-d_6) δ 12.04 (s, 1H), 8.23 (d, *J* = 6.8 Hz, 1H), 8.06–7.96 (m, 2H), 7.89 (d, *J* = 7.7 Hz, 4H), 7.72–7.58 (m, 3H), 7.55–7.39 (m, 6H), 6.19 (d, *J* = 3.4 Hz, 1H), 5.93 (m, 2H), 4.73 (m, 3H); ^{13}C NMR (101 MHz, DMSO-d_6) δ 165.94, 165.03, 157.66, 157.40, 149.50, 141.82, 139.52, 134.42, 134.31, 134.02, 129.82, 129.77, 129.63, 129.21, 129.16, 128.99, 128.89, 126.71, 126.37, 89.35, 79.36, 73.56, 70.72, 64.12; HRMS (ESI) calcd for $C_{30}H_{23}N_2O_9FNa$ $[M+Na]^+$ 597.1280, found 597.1285.

Compound 6e

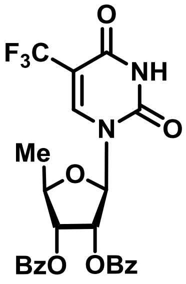

6e

Glycosylation of **1t** (40 mg, 0.073 mmol) with **5e** (26 mg, 0.146 mmol) according to **General procedure C** afforded **6e** (34 mg, 93%) as a white solid: $[\alpha]_D^{25}$ = -56.37 (*c* 0.13, $CHCl_3$); ^1H NMR (400 MHz, Chloroform-*d*) δ 9.38 (s, 1H), 8.05–7.88 (m, 5H), 7.63–7.49 (m, 2H), 7.49–7.33 (m, 4H), 6.08 (d, *J* = 4.6 Hz, 1H), 5.73 (t, *J* = 5.3 Hz, 1H), 5.45 (t, *J* = 5.9 Hz, 1H), 4.52 (m, 1H), 1.59 (d, *J* = 6.4 Hz, 3H); ^{13}C NMR (101 MHz, $CDCl_3$) δ 165.47, 165.41, 158.22, 149.01, 141.27, 141.21, 133.85, 133.72, 129.89, 129.79, 128.72, 128.55, 128.53, 128.31, 122.93, 106.22, 105.89, 90.00, 78.96, 74.67, 74.20, 18.58; HRMS (ESI) calcd for $C_{24}H_{19}N_2O_7F_3Na$ $[M+Na]^+$ 527.1037, found 527.1034.

Compound 6f

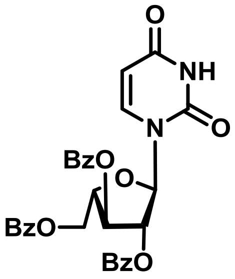

6f

Glycosylation of **1k** (40 mg, 0.06 mmol) with **5a** (13.5 mg, 0.12 mmol) according to **General procedure C** afforded **6f** (30 mg, 91%) as a white solid: $[\alpha]_D^{25}$ = 5.6 (*c* 0.16, $CHCl_3$); ^{1}H NMR (400 MHz, $CDCl_3$) δ 8.80 (s, 1H), 8.13–7.96 (m, 6H), 7.67–7.35 (m, 10H), 6.21 (d, *J* = 3.2 Hz, 1H), 5.96 (t, *J* = 3.0 Hz, 1H), 5.84–5.71 (m, 2H), 4.97 (q, *J* = 4.6 Hz, 1H), 4.81–4.59 (m, 2H); ^{13}C NMR (101 MHz, $CDCl_3$) δ 166.10, 165.37, 165.25, 162.81, 149.95, 140.27, 134.02, 133.97, 133.37, 130.05, 129.86, 129.82, 129.35, 128.72, 128.69, 128.50, 128.42, 128.30, 102.71, 91.75, 83.80, 80.55,77.23, 63.89; HRMS (ESI) calcd for $C_{30}H_{24}N_2O_9Na$ $[M+Na]^+$ 579.1374, found 579.1370.

Compound 6g

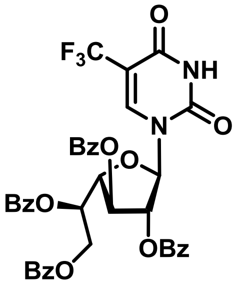

6g

Glycosylation of **1m** (48 mg, 0.06 mmol) with **5e** (22 mg, 0.12 mmol) according to **General procedure C** afforded **6g** (44 mg, 96%) as a white solid: $[\alpha]_D^{25}$ = -5.11 (*c* 0.16, $CHCl_3$); ^{1}H NMR (400 MHz, $CDCl_3$) δ 9.46 (s, 1H), 8.10 (d, *J* = 7.7 Hz, 2H), 8.04–7.94 (m, 3H), 7.90 (m, 4H), 7.61 (d, *J* = 7.4 Hz, 1H), 7.51 (m, 3H), 7.47–7.28 (m, 8H), 6.40 (d, *J* = 2.5 Hz, 1H), 6.09–6.01 (m, 1H), 5.86 (d, *J* = 2.2 Hz, 2H), 5.03–4.95 (m, 1H), 4.85 (dd, *J* = 12.2, 4.3 Hz, 1H), 4.70 (dd, *J* = 12.2, 6.1 Hz, 1H); ^{13}C NMR (101 MHz, $CDCl_3$) δ 166.09, 165.69, 165.42, 165.30, 158.30, 148.92, 140.80, 134.18, 133.85, 133.64, 133.29, 130.03, 130.00, 129.79, 129.68, 129.21, 128.98, 128.76, 128.62, 128.57, 128.40, 128.05, 127.92, 122.97, 120.29, 105.93,

105.60, 91.81, 85.26, 80.81, 77.60, 70.96, 63.15; HRMS (ESI) calcd for $C_{39}H_{29}N_2O_{11}F_3Na$ $[M+Na]^+$ 781.1616, found 781.1615.

Compound 6h

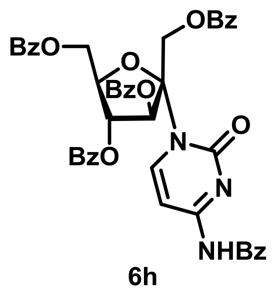

6h

Glycosylation of **1n** (48 mg, 0.06 mmol) with **5c** (26 mg, 0.12 mmol) according to **General procedure C** afforded **6h** (45 mg, 96%) as a white solid: $[\alpha]_D^{25}$ = -4.66 (*c* 0.20, $CHCl_3$); ^1H NMR (400 MHz, $CDCl_3$) δ 9.69 (s, 1H), 8.23 (d, *J* = 7.6 Hz, 1H), 8.07 (d, *J* = 7.7 Hz, 2H), 8.04–7.86 (m, 6H), 7.65 (m, 4H), 7.56–7.34 (m, 10H), 7.29 (m, 4H), 6.81 (s, 1H), 5.61 (d, *J* = 2.5 Hz, 1H), 5.30 (d, *J* = 11.8 Hz, 1H), 5.18 (d, *J* = 11.8 Hz, 1H), 4.71 (m, 3H); ^{13}C NMR (101 MHz, $CDCl_3$) δ 166.06, 165.48, 164.77, 164.27, 163.34, 144.45, 133.91, 133.84, 133.35, 133.23, 133.15, 130.04, 129.77, 129.68, 129.58, 129.29, 129.25, 128.83, 128.79, 128.59, 128.55, 128.40, 128.35, 128.13, 127.83, 99.33, 83.86, 79.59, 77.00, 63.37, 63.27; HRMS (ESI) calcd for $C_{45}H_{35}N_3O_{11}Na$ $[M+Na]^+$:816.2164, found: 816.2165.

Compound 6i

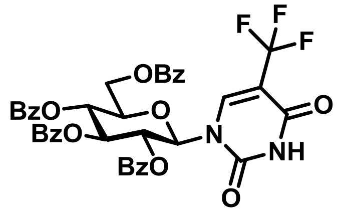

6i

Glycosylation of **1a** (50.0 mg, 0.06mmol) with **5e** (22.4 mg, 0.12 mmol) according to **General procedure C** afforded **6i** (41.9 mg, 89%) as a white solid: $[\alpha]_D^{25}$ = 3.1 (*c* 0.10, CHCl3); ^1H NMR (600 MHz, $CDCl_3$): δ 8.03 (d, *J* = 7.1 Hz, 2H), 7.98 (s, 1H), 7.93 (d, *J* = 7.2 Hz, 2H), 7.87 (d, *J* = 7.2 Hz, 2H), 7.81 (d, *J* = 7.2 Hz, 2H), 7.58 (t, *J* = 7.4 Hz, 1H), 7.55–7.49 (m, 2H), 7.46–7.42 (m, 3H), 7.40–7.34 (m, 4H), 7.30–7.27 (m,

2H), 6.24 (d, *J* = 9.4 Hz, 1H), 6.13 (t, *J* = 9.7 Hz, 1H), 5.79 (t, *J* = 9.9 Hz, 1H), 5.59 (t, *J* = 9.5 Hz, 1H), 4.68 (dd, *J* = 12.5, 2.7 Hz, 1H), 4.51 (dd, *J* = 12.5, 4.9 Hz, 1H), 4.42 (ddd, *J* = 10.0, 4.9, 2.8 Hz, 1H); ^{13}C NMR (151 MHz, $CDCl_3$) δ 166.15, 165.59, 165.51, 165.21, 157.36, 148.99, 134.23, 133.94, 133.74, 133.59, 130.17, 130.04, 129.90, 129.87, 129.31, 128.75, 128.70, 128.66, 128.56, 128.40, 127.67, 121.49 (q, J = 268.5 Hz), 107.00 (q, J = 34.5 Hz), 81.13, 75.82, 72.50, 70.77, 68.63, 62.43; HRMS (ESI) calcd for $C_{39}H_{29}F_3N_2O_{11}Na$ $[M+Na]^+$ 781.1616, found 781.1603.

Compound 6j

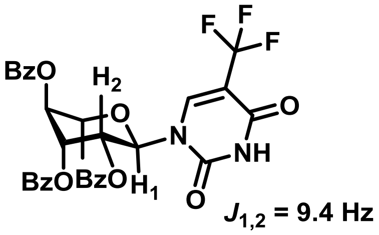

Glycosylation of **1e** (50.0 mg, 0.07mmol) with **5e** (26.4 mg, 0.14 mmol) according to **General procedure C** afforded **6j** (46.5 mg, 99%) as a white solid: $[\alpha]_D^{25}$ = -72.0 (*c* 0.10, CHCl3); ^{1}H NMR (400 MHz, $CDCl_3$): δ 9.22 (s, 1H), 8.16–8.07 (m, 4H), 8.03 (s, 1H), 7.82 (d, *J* = 7.7 Hz, 2H), 7.67 (t, *J* = 7.3 Hz, 2H), 7.58–7.48 (m, 5H), 7.32 (t, *J* = 7.6 Hz, 2H), 6.63 (d, *J* = 9.4 Hz, 1H), 6.02 (t, *J* = 3.5 Hz, 1H), 5.68 (dd, *J* = 9.4, 3.3 Hz, 1H), 5.40–5.32 (m, 1H), 4.64 (q, *J* = 6.8 Hz, 1H), 1.75 (d, *J* = 7.2 Hz, 3H); ^{13}C NMR (101 MHz, $CDCl_3$) δ 165.32, 165.19, 165.10, 159.34, 158.27, 149.37, 141.00, 134.17, 134.15, 134.08, 130.01, 129.97, 129.09, 128.94, 128.91, 128.77, 128.74, 128.11, 119.19, 106.50, 74.98, 74.92, 71.77, 69.60, 67.91, 16.14; HRMS (ESI) calcd for $C_{32}H_{25}F_3N_2O_9Na$ $[M+Na]^+$ 661.1404, found 661.1407.

Compound 6k

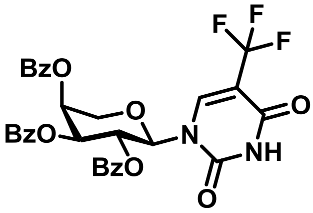

6k

Glycosylation of **1i** (40 mg, 0.06 mmol) with **5e** (22 mg, 0.12 mmol) according to **General procedure C** afforded **6k** (35 mg, 93%) as a white solid: $[\alpha]_D^{25}$ = -117.86 (*c*

0.19, $CHCl_3$); ^1H NMR (400 MHz, $CDCl_3$) δ 8.91 (s, 1H), 8.16–8.03 (m, 3H), 7.86 (dd, *J* = 17.0, 7.8 Hz, 4H), 7.66 (t, *J* = 7.2 Hz, 1H), 7.51 (m, 4H), 7.45–7.19 (m, 5H), 6.12 (d, *J* = 7.6 Hz, 1H), 5.83 (s, 3H), 4.42 (d, *J* = 13.5 Hz, 1H), 4.15 (d, *J* = 13.5 Hz, 1H); ^{13}C NMR (101 MHz, $CDCl_3$) δ 165.72, 165.33, 157.55, 149.16, 140.54, 134.03, 133.95, 133.66, 129.95, 129.78, 129.69, 129.04, 128.88, 128.63, 128.46, 128.44, 127.79, 122.81, 107.04, 106.70, 81.58, 71.00, 69.00, 68.68, 67.32. HRMS (ESI) calcd for $C_{31}H_{23}N_2O_9F_3Na$ $[M+Na]^+$ 647.1248, found 647.1248.

Compound 6l

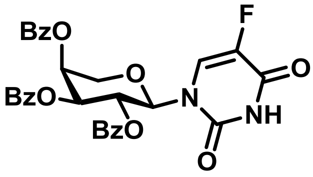

6l

Glycosylation of **1i** (40.0 mg, 0.06 mmol) with **5d** (15.6 mg, 0.12 mmol) according to **General procedure C** afforded **6l** (33.8 mg, 98%) as a white solid: $[\alpha]_D^{25}$ = 268.5 (*c* 0.10, CHCl3); ^1H NMR (400 MHz, $CDCl_3$): δ 9.20 (s, 1H), 8.11 (d, *J* = 7.5 Hz, 2H), 7.88 (d, *J* = 7.6 Hz, 2H), 7.84 (d, *J* = 7.6 Hz, 2H), 7.67 (t, *J* = 7.3 Hz, 1H), 7.61–7.53 (m, 3H), 7.52–7.44 (m, 2H), 7.34 (t, *J* = 7.8 Hz, 2H), 7.29 (t, *J* = 7.8 Hz, 2H), 6.09 (d, *J* = 8.6 Hz, 1H), 5.89–5.76 (m, 3H), 4.42 (d, *J* = 13.3 Hz, 1H), 4.15 (d, *J* = 13.6 Hz, 1H); ^{13}C NMR (101 MHz, $CDCl_3$) δ 165.71, 165.64, 165.42, 156.58, 156.31, 149.08, 142.21, 139.82, 134.03, 133.97, 133.71, 130.06, 129.88, 129.26, 128.97, 128.72, 128.64, 128.56, 128.08, 123.85, 123.51, 81.78, 71.35, 69.09, 68.45, 67.54; HRMS (ESI) calcd for $C_{30}H_{23}FN_2O_9Na$ $[M+Na]^+$ 597.1280, found 597.1280.

Compound 6m

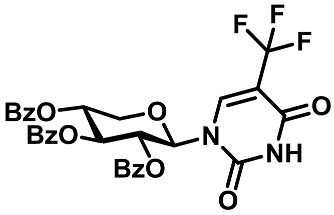

6m

Glycosylation of **1g** (40 mg, 0.06 mmol) with **5e** (22 mg, 0.12 mmol) according to **General procedure C** afforded **6m** (34 mg, 92%) as a white solid: $[\alpha]_D^{25}$ = -2.18 (*c* 0.21, $CHCl_3$); ^{1}H NMR (400 MHz, Chloroform-*d*) δ 9.09 (s, 1H), 8.10–7.85 (m, 6H), 7.57–7.27 (m, 10H), 6.14 (d, *J* = 9.6 Hz, 1H), 6.14 (t, *J* = 9.2 Hz, 1H), 5.63–5.45 (m, 2H), 4.56 (dd, *J* = 11.5, 5.7 Hz, 1H), 3.82 (t, *J* = 11.5 Hz, 1H); ^{13}C NMR (101 MHz, $CDCl_3$) δ 165.51, 165.45, 157.68, 149.18, 140.70, 140.64, 134.01, 133.79, 133.60, 129.98, 129.89, 129.73, 128.61, 128.58, 128.47, 128.45, 127.61, 122.78, 120.10, 106.92, 106.58, 81.61, 72.07, 70.61, 69.27, 65.98; HRMS (ESI) calcd for $C_{31}H_{23}N_2O_9F_3Na$ $[M+Na]^+$ 647.1248, found 647.1245.

Compound 6n

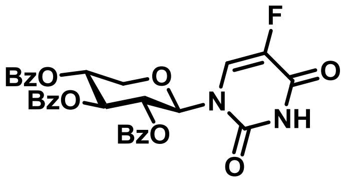

6n

Glycosylation of **1g** (60.0 mg, 0.09 mmol) with **5d** (23.3 mg, 0.18 mmol) according to **General procedure C** afforded **6n** (51.1 mg, 99%) as a white solid: $[\alpha]_D^{25}$ = 14.7 (*c* 0.16, CHCl3); ^{1}H NMR (400 MHz, $CDCl_3$): δ 9.42 (s, 1H), 7.97 (d, *J* = 7.5 Hz, 2H), 7.90–7.82 (m, 4H), 7.61 (d, *J* = 5.6 Hz, 1H), 7.54 (t, *J* = 7.4 Hz, 1H), 7.51–7.38 (m, 4H), 7.35–7.27 (m, 4H), 6.12 (d, *J* = 8.8 Hz, 1H), 6.11 (t, *J* = 9.6 Hz, 1H), 5.57 (t, *J* = 9.4 Hz, 1H), 5.49 (td, *J* = 10.1, 5.7 Hz, 1H), 4.53 (dd, *J* = 11.4, 5.6 Hz, 1H), 3.81 (t, *J* = 11.0 Hz, 1H); ^{13}C NMR (101 MHz, $CDCl_3$) δ 165.67, 165.61, 165.48, 156.77, 156.50, 149.13, 142.16, 139.77, 134.03, 133.87, 133.66, 130.08, 129.99, 129.83, 128.71, 128.67, 128.62, 128.55, 127.91, 123.97, 123.63, 81.76, 72.37, 70.35, 69.49, 65.93; HRMS (ESI) calcd for $C_{30}H_{24}FN_2O_9$ $[M+H]^+$ 575.1460, found 575.1459.

Compound 6o

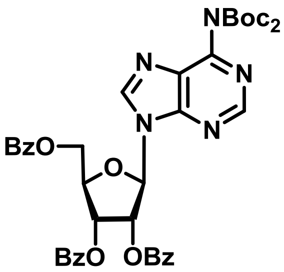

6o

Glycosylation of **1l** (40 mg, 0.06 mmol) with **5f** (16.8 mg, 0.05 mmol) according to **General procedure B** afforded **6o**[27] (33 mg, 84%) as a white solid: $[\alpha]_D^{25}$ = -89.46 (*c* 0.13, $CHCl_3$); ^{1}H NMR (400 MHz, $CDCl_3$) δ 8.76 (s, 1H), 8.24 (s, 1H), 8.17–8.08 (m, 2H), 8.06–7.98 (m, 2H), 7.97–7.86 (m, 2H), 7.61–7.53 (m, 3H), 7.48–7.34 (m, 6H), 6.50 (d, *J* = 5.3 Hz, 1H), 6.40 (d, *J* = 5.6 Hz, 1H), 6.26 (t, *J* = 5.2 Hz, 1H), 4.96–4.84 (m, 2H), 4.73 (dd, *J* = 12.1, 4.2 Hz, 1H), 1.43 (s, 18H); ^{13}C NMR (101 MHz, $CDCl_3$) δ 166.16, 165.33, 165.07, 152.85, 152.46, 150.73, 150.32, 143.30, 133.85, 133.79, 133.49, 129.83, 129.77, 129.31, 128.67, 128.65, 128.59, 128.53, 128.30, 86.99, 83.92, 80.88, 73.87, 71.48, 63.59, 27.78; HRMS (ESI) calcd for $C_{41}H_{41}N_5O_{11}Na$ $[M+Na]^+$ 802.2695, found 802.2710.

Compound 6p

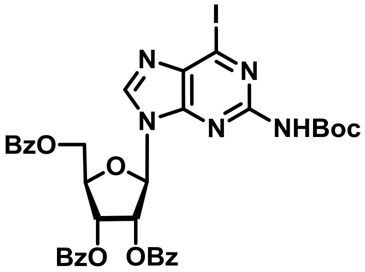

6p

Glycosylation of **1l** (40 mg, 0.06 mmol) with **5g** (18 mg, 0.05 mmol) according to **General procedure B** afforded **6p**[27] (38 mg, 85%) as a white solid: $[\alpha]_D^{25}$ = -46.29 (*c* 0.19, $CHCl_3$); ^{1}H NMR (400 MHz, $CDCl_3$) δ 8.09 (s, 1H), 8.02 (t, *J* = 7.3 Hz, 4H), 7.96–7.91 (m, 2H), 7.62–7.51 (m, 4H), 7.43–7.33 (m, 6H), 6.64 (t, *J* = 5.8 Hz, 1H), 6.27 (d, *J* = 6.8 Hz, 2H), 4.96 (dd, *J* = 11.8, 3.9 Hz, 1H), 4.94–4.86 (m, 1H), 4.79 (dd, *J* = 11.8, 5.5 Hz, 1H), 1.46 (s, 9H); ^{13}C NMR (101 MHz, $CDCl_3$) δ 166.18, 165.34, 165.15, 152.14, 149.57, 148.12, 142.28, 135.40, 133.80, 133.53, 133.30, 129.87,

129.81, 129.63, 129.45, 128.94, 128.52, 128.49, 128.46, 122.59, 88.35, 81.50, 80.86, 74.85, 71.81, 64.00, 28.08; HRMS (ESI) calcd for $C_{36}H_{32}N_5O_9INa$ $[M+Na]^+$ 828.1137, found 828.1135.

Compound 6q

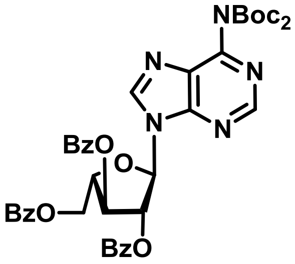

6q

Glycosylation of **1k** (40 mg, 0.06 mmol) with **5f** (17 mg, 0.05 mmol) according to **General procedure B** afforded **6q**[27] (32 mg, 83%) as a white solid: $[\alpha]_D^{25}$ = -13.76 (*c* 0.21, $CHCl_3$); ^1H NMR (400 MHz, $CDCl_3$) δ 8.89 (s, 1H), 8.36 (s, 1H), 8.13–8.00 (m, 4H), 7.96–7.86 (m, 2H), 7.68–7.34 (m, 9H), 6.57–6.52 (m, 2H), 5.95–5.91 (m, 1H), 5.11 (d, *J* = 4.8 Hz, 1H), 4.82–4.79 (m, 2H), 1.46 (s, 18H); ^{13}C NMR (101 MHz, $CDCl_3$) δ 166.14, 165.47, 165.27, 152.82, 152.46, 150.73, 150.37, 143.13, 134.02, 133.94, 133.35, 130.03, 129.83, 129.40, 129.30, 128.75, 128.71, 128.47, 128.41, 128.25, 89.25, 83.89, 83.31, 80.55, 63.59, 27.81; HRMS (ESI) calcd for $C_{41}H_{41}N_5O_{11}Na$ $[M+Na]^+$ 802.2695, found 802.2693.

Compound 6r

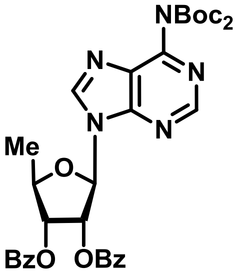

6r

Glycosylation of **1t** (40 mg, 0.073 mmol) with **5f** (21 mg, 0.061 mmol) according to **General procedure B** afforded **6r** (40 mg, 83%) as a white solid: $[\alpha]_D^{25}$ = -65.02 (*c* 0.20, $CHCl_3$); ^1H NMR (400 MHz, $CDCl_3$) δ 8.91 (s, 1H), 8.27 (s, 1H), 8.01 (d, *J* = 7.8 Hz, 2H), 7.89 (d, *J* = 7.8 Hz, 2H), 7.61–7.51 (m, 2H), 7.45–7.32 (m, 4H), 6.39 (d,

J = 4.8 Hz, 1H), 6.31 (d, *J* = 5.4 Hz, 1H), 5.83 (t, *J* = 5.5 Hz, 1H), 4.69–4.57 (m, 1H), 1.65 (d, *J* = 6.4 Hz, 3H), 1.46 (s, 18H); ^{13}C NMR (101 MHz, $CDCl_3$) δ 165.42, 165.10, 152.84, 152.41, 150.68, 150.36, 143.42, 133.74, 133.64, 129.80, 129.77, 129.57, 128.94, 128.54, 128.48, 87.28, 83.89, 79.19, 75.13, 74.10, 27.80, 18.83; HRMS (ESI) calcd for $C_{34}H_{37}N_5O_9Na$ $[M+Na]^+$:682.2483, found: 682.2487.

Compound 6s

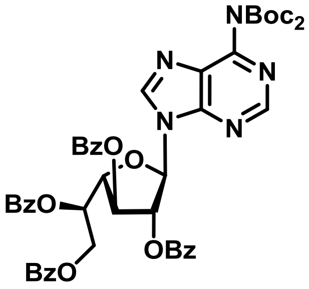

6s

Glycosylation of **1m** (48 mg, 0.06 mmol) with **5f** (17 mg, 0.05 mmol) according to **General procedure B** afforded **6s** (37 mg, 82%) as a white solid: $[\alpha]_D^{25}$ = -19.09 (*c* 0.15, $CHCl_3$); ^{1}H NMR (400 MHz, $CDCl_3$) δ 8.87 (s, 1H), 8.36 (s, 1H), 8.13 (d, *J* = 7.7 Hz, 2H), 8.06–7.80 (m, 6H), 7.66–7.32 (m, 12H), 6.64 (d, *J* = 3.1 Hz, 1H), 6.46–6.42 (m, 1H), 6.14–6.03 (m, 1H), 6.02 - 5.98 (m, 1H), 5.15 (t, *J* = 4.5 Hz, 1H), 4.87–4.68 (m, 2H), 1.45 (s, 18H); ^{13}C NMR (101 MHz, $CDCl_3$) δ 166.04, 165.70, 165.54, 165.32, 152.85, 152.47, 150.71, 150.34, 143.17, 133.93, 133.79, 133.54, 133.24, 130.03, 129.96, 129.88, 129.69, 129.34, 129.26, 129.17, 128.70, 128.57, 128.54, 128.40, 128.09, 89.29, 83.89, 80.76, 77.74 , 70.78, 63.20, 27.81; HRMS (ESI) calcd for $C_{49}H_{47}N_5O_{13}Na$ $[M+Na]^+$ 936.3063, found 936.3062.

Compound 6t

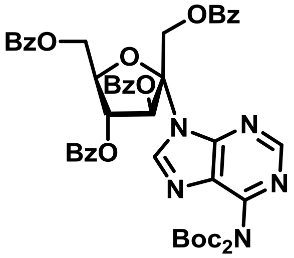

6t

Glycosylation of **1n** (48 mg, 0.06 mmol) with **5f** (17 mg, 0.05 mmol) according to **General procedure B** afforded **6t** (38 mg, 84%) as a white solid: $[\alpha]_D^{25}$ = -2.44 (*c* 0.16, $CHCl_3$); ^1H NMR (400 MHz, $CDCl_3$) δ 8.84 (s, 1H), 8.54 (s, 1H), 8.09 (d, *J* = 7.9 Hz, 2H), 8.06 (dd, *J* = 7.9 Hz, 2H), 7.77 (d, *J* = 7.8 Hz, 2H), 7.63 (t, *J* = 7.9 Hz, 1H), 7.56 (s, 1H), 7.48 (dt, *J* = 15.2, 6.2 Hz, 5H), 7.38–7.28 (m, 8H), 5.77 (d, *J* = 3.1 Hz, 1H), 5.08 (m, 2H), 4.85 (d, *J* = 5.2 Hz, 2H), 4.83–4.75 (m, 1H), 1.38 (s, 18H); ^{13}C NMR (101 MHz, $CDCl_3$) δ 166.10, 165.23, 165.03, 164.50, 152.64, 152.25, 150.86, 150.33, 142.63, 134.10, 133.70, 133.45, 133.29, 130.02, 129.79, 129.70, 129.43, 129.30, 128.89, 128.73, 128.59, 128.42, 128.39, 128.30, 127.95, 97.76, 84.47, 83.78, 78.60, 77.84, 64.49, 63.33, 27.71; HRMS (ESI) calcd for $C_{49}H_{47}N_5O_{13}Na$ $[M+Na]^+$ 936.3063, found 936.3066.

Compound 6u

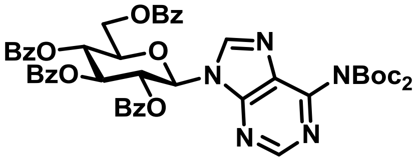

6u

Glycosylation of **1a** (50.0 mg, 0.06mmol) with **5f** (22.4 mg, 0.12 mmol) according to **General procedure B** afforded **6u**[27] (41.9 mg, 89%) as a white solid: $[\alpha]_D^{25}$ = -16.8 (*c* 0.11, $CHCl_3$); ^1H NMR (400 MHz, $CDCl_3$): δ 8.71 (s, 1H), 8.35 (s, 1H), 7.94 (d, *J* = 7.8 Hz, 2H), 7.87 (d, *J* = 7.8 Hz, 2H), 7.75 (d, *J* = 7.8 Hz, 2H), 7.61 (d, *J* = 7.8 Hz, 2H), 7.51–7.13 (m, 12H), 6.28 (d, *J* = 8.3 Hz, 1H), 6.16–6.03 (m, 2H), 5.86 (t, *J* = 8.9 Hz, 1H), 4.63 (d, *J* = 10.3 Hz, 1H), 4.52–4.38 (m, 2H), 1.25 (s, 18H); ^{13}C NMR (101 MHz, $CDCl_3$) δ 166.12, 165.67, 165.23, 164.85, 153.21, 152.54, 150.77, 150.11, 142.27, 133.86, 133.83, 133.61, 133.42, 130.01, 129.89, 129.85, 129.40, 128.63, 128.60, 128.55, 128.53, 128.49, 128.44, 127.68, 83.86, 81.14, 75.74, 73.15, 71.14, 68.98, 62.59, 27.73; HRMS (ESI) calcd for $C_{49}H_{47}N_5O_{13}Na$ $[M+Na]^+$ 936.3063, found 936.3064.

Compound 6v

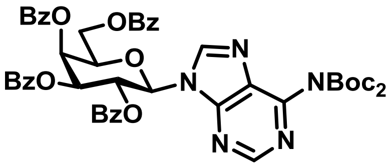

6v

Glycosylation of **1c** (65.0 mg, 0.08mmol) with **5f** (22.6 mg, 0.07 mmol) according to **General procedure B** afforded **6v** (41.6 mg, 67%) as a white solid: $[\alpha]_D^{25}$ = 150.2 (*c* 0.20, $CHCl_3$); ^1H NMR (400 MHz, $CDCl_3$): δ 8.84 (s, 1H), 8.48 (s, 1H), 8.18 (d, *J* = 7.2 Hz, 2H), 7.99 (d, *J* = 6.9 Hz, 2H), 7.79 (d, *J* = 7.2 Hz, 2H), 7.73–7.67 (m, 3H), 7.61–7.53 (m, 3H), 7.49–7.38 (m, 4H), 7.31–7.21 (m, 4H), 6.47 (t, *J* = 9.7 Hz, 1H), 6.28 (d, *J* = 9.4 Hz, 1H), 6.18 (d, *J* = 3.3 Hz, 1H), 5.88 (dd, *J* = 10.1, 3.3 Hz, 1H), 4.74–4.61 (m, 2H), 4.51 (dd, *J* = 10.6, 5.1 Hz, 1H), 1.35 (s, 24H); ^{13}C NMR (101 MHz, $CDCl_3$) δ 166.13, 165.47, 165.44, 165.00, 153.26, 152.60, 150.83, 150.19, 142.50, 134.10, 133.90, 133.69, 133.57, 130.14, 129.96, 129.93, 129.83, 129.23, 129.02, 128.95, 128.76, 128.62, 128.55, 128.53, 128.50, 127.83, 83.91, 81.67, 74.72, 72.05, 68.87, 68.11, 62.04, 27.79; HRMS (ESI) calcd for $C_{49}H_{48}N_5O_{13}$ $[M+H]^+$ 914.3243, found 914.3238.

Compound 6w

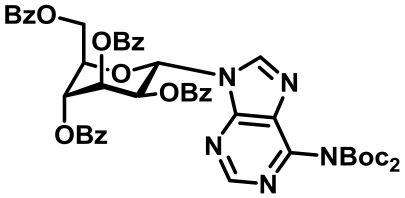

6w

Glycosylation of **1d** (60.0 mg, 0.07mmol) with **5f** (20.9 mg, 0.06 mmol) according to **General procedure B** afforded **6w** (43.3 mg, 76%) as a white solid: $[\alpha]_D^{25}$ = 29.5 (*c* 0.17, $CHCl_3$); ^1H NMR (400 MHz, $CDCl_3$): δ 8.90 (s, 1H), 8.43 (s, 1H), 8.15 (d, *J* = 7.8 Hz, 2H), 8.03 (t, *J* = 8.3 Hz, 4H), 7.86 (d, *J* = 7.8 Hz, 2H), 7.65–7.30 (m, 12H), 6.93 (dd, *J* = 6.4, 3.3 Hz, 1H), 6.62 (d, *J* = 6.3 Hz, 1H), 6.24 (dd, *J* = 6.0, 3.4 Hz, 1H), 5.88 (t, *J* = 5.7 Hz, 1H), 5.22 (dd, *J* = 12.3, 7.7 Hz, 1H), 4.75–4.67 (m, 1H), 4.56 (dd, *J* = 12.3, 3.3 Hz, 1H), 1.37 (s, 18H); ^{13}C NMR (101 MHz, $CDCl_3$) δ 166.46, 165.31, 165.14, 164.94, 153.25, 152.72, 150.92, 150.33, 143.92, 134.04, 133.97, 133.89,

133.51, 130.20, 130.00, 129.88, 129.86, 129.42, 129.35, 128.89, 128.81, 128.76, 128.72, 128.66, 128.62, 128.47, 83.93, 79.68, 74.68, 69.38, 67.98, 67.77, 61.31, 27.82; HRMS (ESI) calcd for $C_{49}H_{48}N_5O_{13}$ $[M+H]^+$ 914.3243, found 914.3237.

Compound 6x

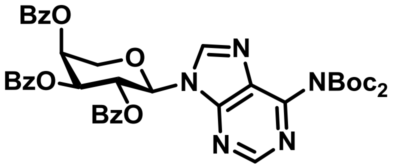

6x

Glycosylation of **1i** (55.0 mg, 0.08 mmol) with **5f** (23.0 mg, 0.07 mmol) according to **General procedure B** afforded **6x** (36.6 mg, 68%) as a white solid: $[\alpha]_D^{25}$ = 45.4 (*c* 0.12, $CHCl_3$); ^{1}H NMR (400 MHz, $CDCl_3$): δ 8.83 (s, 1H), 8.43 (s, 1H), 8.21 (d, *J* = 7.4 Hz, 2H), 7.85 (d, *J* = 7.4 Hz, 2H), 7.74–7.65 (m, 3H), 7.57 (t, *J* = 7.6 Hz, 2H), 7.50–7.40 (m, 2H), 7.32–7.22 (m, 4H), 6.48 (t, *J* = 9.7 Hz, 1H), 6.16 (d, *J* = 9.3 Hz, 1H), 5.93–5.88 (m, 1H), 5.83 (dd, *J* = 10.0, 3.4 Hz, 1H), 4.52 (dd, *J* = 13.6, 2.1 Hz, 1H), 4.25 (d, *J* = 13.5 Hz, 1H), 1.32 (s, 18H); ^{13}C NMR (151 MHz, $CDCl_3$) δ 165.72, 165.56, 165.06, 153.21, 152.56, 150.83, 150.05, 142.51, 133.96, 133.89, 133.73, 130.08, 129.93, 129.81, 129.34, 128.94, 128.77, 128.64, 128.58, 128.50, 127.89, 83.83, 82.09, 71.87, 69.08, 69.02, 67.72, 27.76; HRMS (ESI) calcd for $C_{41}H_{42}N_5O_{11}$ $[M+H]^+$ 780.2875, found 780.2878.

Compound 6y

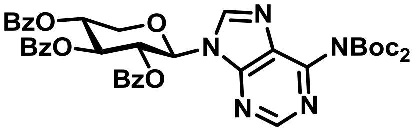

6y

Glycosylation of **1g** (40 mg, 0.06 mmol) with **5f** (17 mg, 0.05 mmol) according to **General procedure B** afforded **6y** (30 mg, 76%) as a white solid: $[\alpha]_D^{25}$ = -11.75 (*c* 0.17, $CHCl_3$); ^{1}H NMR (400 MHz, $CDCl_3$) δ 8.81 (s, 1H), 8.42 (s, 1H), 8.05–7.94 (m, 2H), 7.94–7.84 (m, 2H), 7.75–7.62 (m, 2H), 7.55 (t, *J* = 7.5 Hz, 1H), 7.50–7.22 (m, 8H), 6.26–6.08 (m, 3H), 5.65 (td, *J* = 9.8, 5.6 Hz, 1H), 4.63 (dd, *J* = 11.6, 5.6 Hz, 1H),

3.91 (t, J = 11.6 Hz, 1H), 1.32 (s, 18H); ^{13}C NMR (101 MHz, $CDCl_3$) δ 165.56, 165.47, 164.76, 153.07, 152.44, 150.68, 149.92, 142.24, 133.73, 133.53, 129.89, 129.76, 129.73, 128.60, 128.58, 128.53, 128.43, 128.33, 127.62, 83.72, 81.71, 72.66, 70.89, 69.52, 66.19, 27.62; HRMS (ESI) calcd for $C_{41}H_{41}N_5O_{11}Na$ $[M+Na]^+$ 802.2695, found 802.2694.

Compound 6z

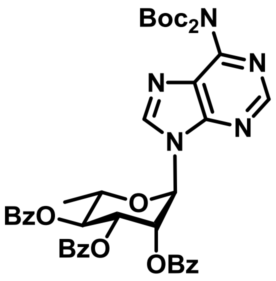

6z

Glycosylation of **1e** (55.0 mg, 0.08mmol) with **5f** (22.5 mg, 0.06 mmol) according to **General procedure B** afforded **6z**[27] (35.4 mg, 66%) as a white solid: $[\alpha]_D^{25}$ = -3.0 (c 0.13, $CHCl_3$); ^{1}H NMR (400 MHz, $CDCl_3$): δ 8.94 (s, 1H), 8.43 (s, 1H), 8.09 (d, J = 7.5 Hz, 2H), 8.00 (d, J = 7.5 Hz, 2H), 7.89 (d, J = 7.5 Hz, 2H), 7.64–7.34 (m, 9H), 6.61 (dd, J = 5.4, 3.7 Hz, 1H), 6.50 (d, J = 5.6 Hz, 1H), 6.15 (dd, J = 6.2, 3.5 Hz, 1H), 5.63 (t, J = 6.1 Hz, 1H), 4.46–4.37 (m, 1H), 1.62 (d, J = 6.6 Hz, 3H), 1.41 (s, 18H); ^{13}C NMR (101 MHz, $CDCl_3$) δ 165.39, 165.16, 165.05, 153.21, 152.63, 150.77, 150.31, 143.07, 133.78, 133.74, 133.67, 129.93, 129.85, 129.83, 128.94, 128.92, 128.83, 128.64, 128.53, 128.38, 83.87, 78.99, 72.11, 71.80, 69.74, 68.62, 27.74, 16.97; HRMS (ESI) calcd for $C_{42}H_{44}N_5O_{11}$ $[M+H]^+$ 794.3032, found 794.3035.

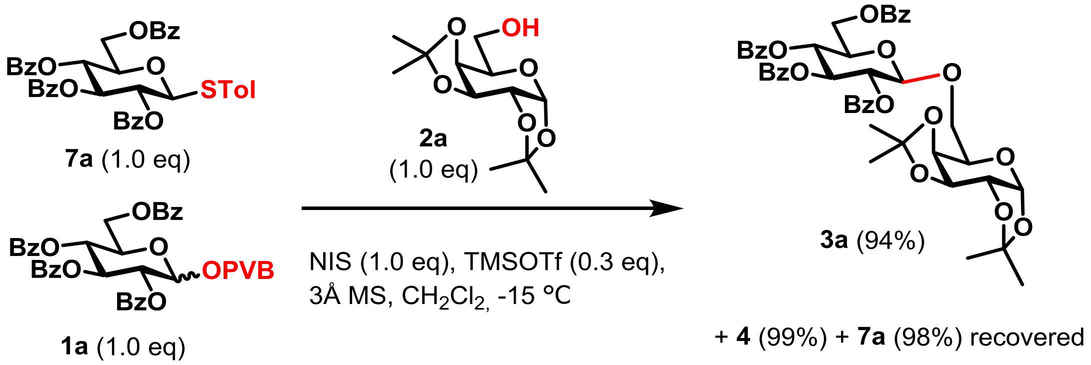

Supplementary Figure 6. Comparison of the donor reactivity of glycosyl PVB and

thioglycoside.

A solution of glycosyl donor **1a** (51.2 mg, 0.06 mmol), thioglycoside **7a**[45] (44.8 mg, 0.06 mmol), and acceptor **2a** (16.6 mg, 0.06 mmol) in dry CH_2Cl_2 (2 mL) was stirred at room temperature for 30 mins in the presence of activated 3Å MS (120 mg) under Ar atmosphere. Then the vessel was chilled to -15 ℃, to which NIS (14.3 mg, 0.06 mmol) and TMSOTf (3.5 μL, 0.02 mmol) were added. The reaction mixture was stirred for 2 h at -15 ℃. Then, Et_3N was added to quench the reaction. After the solvent was removed under reduced pressure, the resulting residue was purified by silica gel column chromatography (petroleum ether/EtOAc = 5:1 to 3:1) to afford **3a** (50.4 mg, 94%), **4** (22.2 mg, 99%), and **7a** (43.8 mg, 98%).

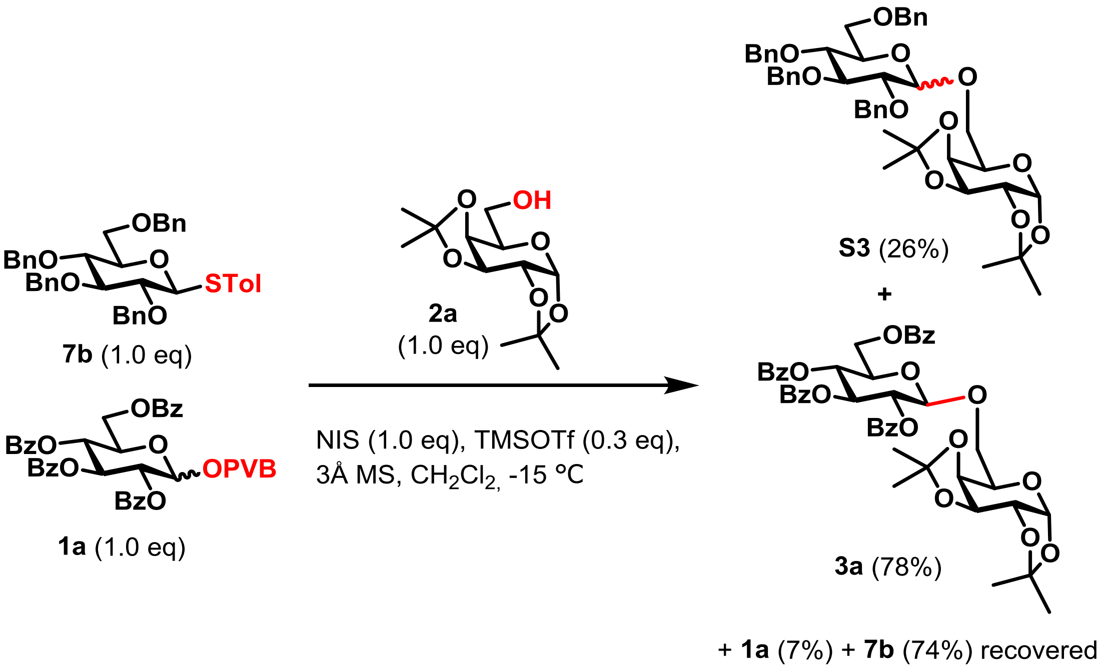

Supplementary Figure 7. Comparison of the donor reactivity of glycosyl PVB and thioglycoside.

A solution of glycosyl donor **1a** (61.7 mg, 0.08 mmol), thioglycoside **7b**[46] (49.7 mg, 0.08 mmol), and acceptor **2a** (20.0 mg, 0.08 mmol) in dry CH_2Cl_2 (2.3 mL) was stirred at room temperature for 30 mins in the presence of activated 3Å MS (130 mg) under Ar atmosphere. Then the vessel was chilled to -15 ℃, to which NIS (17.3 mg, 0.08 mmol) and TMSOTf (4.2 μL, 0.02 mmol) were added. The reaction mixture was

stirred for 2 h at -15 ℃. Then, Et_3N was added to quench the reaction. After the solvent was removed under reduced pressure, the resulting residue was purified by silica gel column chromatography (petroleum ether/EtOAc = 5:1 to 4:1 to 3:1) to afford **S3**[47] (15.9 mg, 26%), **3a** (50.2 mg, 78%), **1a** (4.7 mg, 7%), and **7b** (36.8 mg, 74%).

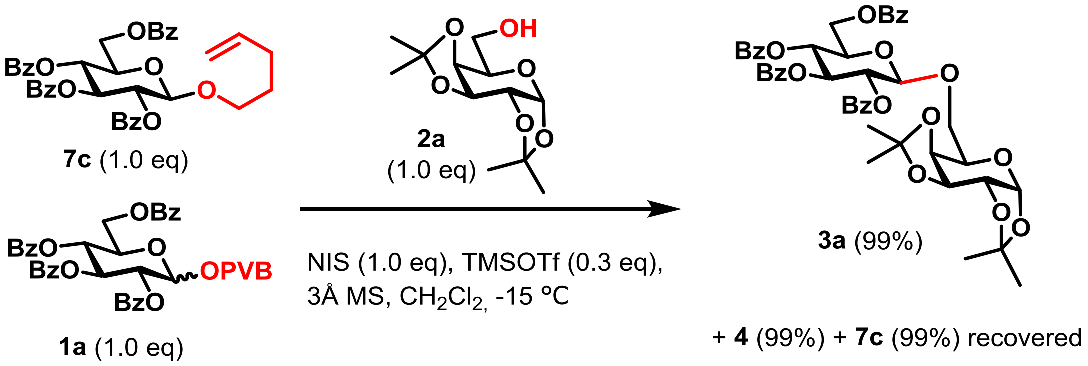

Supplementary Figure 8. Comparison of the donor reactivity of glycosyl PVB and *n*-pentenyl glycoside.

A solution of glycosyl donor **1a** (55.5 mg, 0.07 mmol), *n*-Pen glycoside **7c**[36] (46.0 mg, 0.07 mmol), and acceptor **2a** (18.0 mg, 0.07 mmol) in dry CH_2Cl_2 (2.1 mL) was stirred at room temperature for 30 mins in the presence of activated 3Å MS (120 mg) under Ar atmosphere. Then the vessel was chilled to -15 ℃, to which NIS (15.6 mg, 0.07 mmol) and TMSOTf (3.8 μL, 0.02 mmol) were added. The reaction mixture was stirred for 2 h at -15 ℃. Then, Et_3N was added to quench the reaction. After the solvent was removed under reduced pressure, the resulting residue was purified by silica gel column chromatography (petroleum ether/EtOAc = 4:1 to 2.5:1) to afford **3a** (57.5 mg, 99%), **4** (24.0 mg, 99%), and **7c** (45.7 mg, 99%).

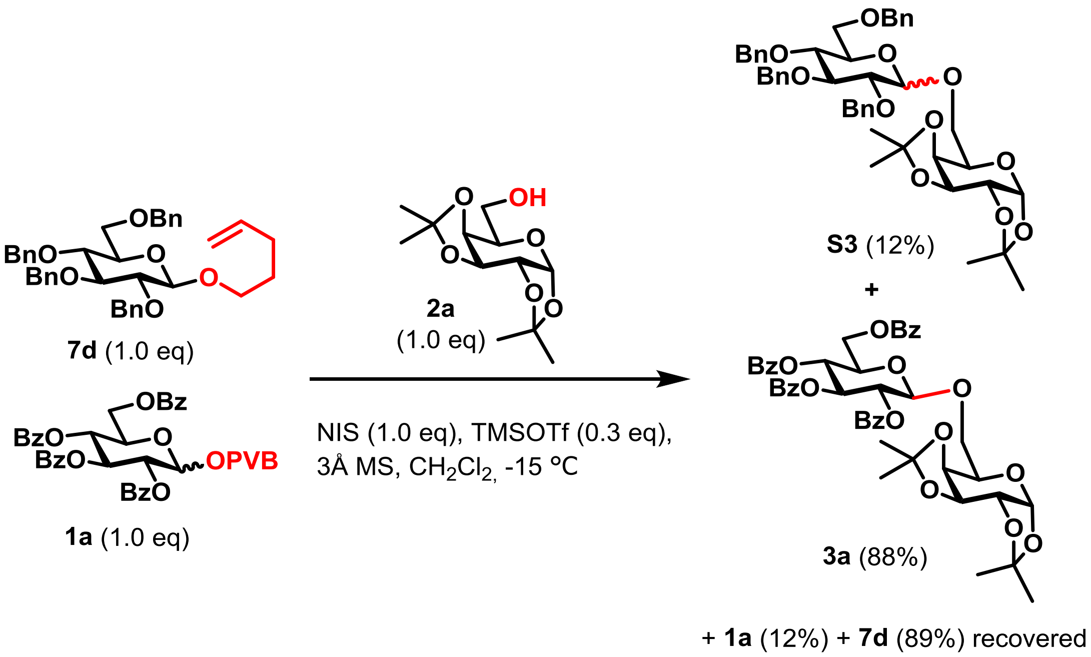

Supplementary Figure 9. Comparison of the donor reactivity of glycosyl PVB and *n*-pentenyl glycoside.

A solution of glycosyl donor **1a** (67.5 mg, 0.08 mmol), *n*-Pen glycoside **7d**[48] (51.2 mg, 0.08 mmol), and acceptor **2a** (21.9 mg, 0.08 mmol) in dry CH_2Cl_2 (2.6 mL) was stirred at room temperature for 30 mins in the presence of activated 3Å MS (150 mg) under Ar atmosphere. Then the vessel was chilled to -15 ℃, to which NIS (18.9 mg, 0.08 mmol) and TMSOTf (4.6 μL, 0.03 mmol) were added. The reaction mixture was stirred for 2 h at -15 ℃. Then, Et_3N was added to quench the reaction. After the solvent was removed under reduced pressure, the resulting residue was purified by silica gel column chromatography (petroleum ether/EtOAc = 4:1 to 2.5:1) to afford **S3** (7.8 mg, 12%), **3a** (62.2 mg, 88%), **1a** (8.0 mg, 12%), and **7d** (45.6 mg, 89%).

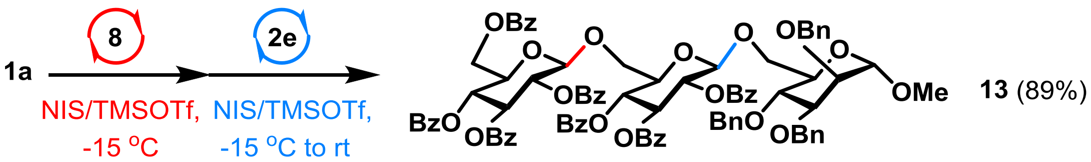

Supplementary Figure 10. One-pot synthesis of trisaccharide **13**.

A solution of glycosyl donor **1a** (33.5 mg, 0.042 mmol) and acceptor **8**[49] (25.0 mg, 0.042mmol) in dry CH_2Cl_2 (1.3 mL) was stirred at room temperature for 30 mins in

the presence of activated 3Å MS (80 mg) under Ar atmosphere. Then the vessel was chilled to -15 ℃, to which NIS (9.4 mg, 0.042 mmol) and TMSOTf (2.3 μL, 0.013 mmol) were added. The reaction mixture was stirred for 2 h at -15 ℃. Acceptor **2e** (17.6 mg, 0.038 mmol) was added to the reaction mixture, to which another portion of NIS (12.8 mg, 0.057 mmol) and TMSOTf (2.1 μL, 0.011 mmol) were added. The reaction mixture was warmed gradually to room temperature and stirred for 2 h. Et_3N was added to quench the reaction and the solvent was removed under reduced pressure. The resulting residue was purified by silica gel column chromatography (petroleum ether/EtOAc = 3:1 to 1.8:1) to afford trisaccharide **13** (51.0 mg, 89%) as a white solid: $[\alpha]_D^{25}$ = -1.6 (c 0.15, $CHCl_3$); ^{1}H NMR (400 MHz, $CDCl_3$) δ 8.07 (d, *J* = 7.8 Hz, 2H), 8.00 (d, *J* = 7.7 Hz, 2H), 7.93–7.87 (m, 6H), 7.85 (d, *J* = 7.7 Hz, 2H), 7.81 (d, *J* = 7.8 Hz, 2H), 7.58 (t, *J* = 7.5 Hz, 1H), 7.54 (d, *J* = 7.2 Hz, 1H), 7.51 (d, *J* = 7.3 Hz, 1H), 7.49–7.19 (m, 33H), 5.86 (d, *J* = 9.4 Hz, 1H), 5.82 (d, *J* = 9.2 Hz, 1H), 5.62 (t, *J* = 9.7 Hz, 1H), 5.52 (ddd, *J* = 10.3, 7.9, 3.3 Hz, 2H), 5.38 (t, *J* = 9.7 Hz, 1H), 5.00 (d, *J* = 7.8 Hz, 1H), 4.78–4.70 (m, 3H), 4.68 (d, *J* = 10.8 Hz, 2H), 4.60 (dd, *J* = 12.2, 3.2 Hz, 1H), 4.56 (s, 2H), 4.49–4.40 (m, 2H), 4.14 (d, *J* = 10.4 Hz, 1H), 4.10 (d, *J* = 11.0 Hz, 1H), 4.07–3.98 (ddd, *J* = 16.8, 9.6, 3.0 Hz, 2H), 3.93 (dd, *J* = 11.5, 7.1 Hz, 1H), 3.85–3.73 (m, 3H), 3.68–3.54 (m, 2H), 3.12 (s, 3H); ^{13}C NMR (101 MHz, $CDCl_3$) δ 166.20, 165.85, 165.81, 165.40, 165.22, 164.98, 138.77, 138.66, 138.50, 133.53, 133.51, 133.38, 133.32, 133.24, 133.00, 129.95, 129.87, 129.82, 129.67, 129.55, 129.36, 128.95, 128.90, 128.88, 128.58, 128.54, 128.52, 128.48, 128.41, 128.38, 128.34, 128.31, 127.93, 127.70, 127.65, 127.57, 127.51, 101.41, 101.24, 98.89, 80.31, 74.78, 74.67, 74.45, 74.18, 72.98, 72.85, 72.61, 72.30, 72.01, 71.92, 71.30, 69.97, 69.60, 68.88, 68.46, 63.08, 54.62; HRMS (ESI) calcd for $C_{89}H_{80}O_{23}Na$ $[M+Na]^+$ 1539.4983, found 1539.5008.

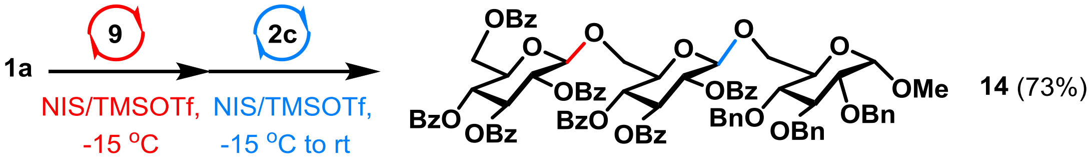

Supplementary Figure 11. One-pot synthesis of trisaccharide **14**.

A solution of glycosyl donor **1a** (54.5 mg, 0.068 mmol) and acceptor **9**[26] (45.7 mg, 0.081mmol) in dry CH_2Cl_2 (2.1 mL) was stirred at room temperature for 30 mins in the presence of activated 3Å MS (120 mg) under Ar atmosphere. Then the vessel was chilled to -15 ℃, to which NIS (18.3 mg, 0.081 mmol) and TMSOTf (3.7 μL, 0.020 mmol) were added. The reaction mixture was stirred for 2 h at -15 ℃. Acceptor **2c** (34.7 mg, 0.075 mmol) was added to the reaction mixture, to which another portion of NIS (22.9 mg, 0.102 mmol) and TMSOTf (3.7 μL, 0.020 mmol) were added. The reaction mixture was warmed gradually to room temperature and stirred for 2 h. Et_3N was added to quench the reaction and the solvent was removed under reduced pressure. The resulting residue was purified by silica gel column chromatography (petroleum ether/EtOAc = 5:1 to 2.5:1) to afford trisaccharide **14**[S4h] (74.9 mg, 73%) as a white solid: $[\alpha]_D^{25}$ = 4.54 (c 0.22, $CHCl_3$); ^1H NMR (400 MHz, $CDCl_3$) δ 8.08 (d, *J* = 7.8 Hz, 2H), 8.00 (d, *J* = 7.7 Hz, 2H), 7.96–7.88 (m, 6H), 7.86 (d, *J* = 7.8 Hz, 2H), 7.82 (d, *J* = 7.7 Hz, 2H), 7.58 (t, *J* = 7.4 Hz, 1H), 7.54–7.23 (m, 31H), 7.19 (t, *J* = 7.7 Hz, 2H), 7.03 (d, *J* = 6.8 Hz, 2H), 5.93 (t, *J* = 9.7 Hz, 1H), 5.84 (t, *J* = 9.6 Hz, 1H), 5.69 (t, *J* = 9.7 Hz, 1H), 5.62–5.51 (m, 2H), 5.38 (t, *J* = 9.1 Hz, 1H), 5.08 (d, *J* = 7.9 Hz, 1H), 4.95 (d, *J* = 11.0 Hz, 1H), 4.84–4.72 (m, 2H), 4.71–4.62 (m, 3H), 4.58 (d, *J* = 7.9 Hz, 1H), 4.48 (dd, *J* = 12.3, 5.1 Hz, 1H), 4.45 (d, *J* = 11.4 Hz, 1H), 4.24 (d, *J* = 11.3 Hz, 1H), 4.18–4.06 (m, 2H), 4.04–3.90 (m, 4H), 3.58 (d, *J* = 10.0 Hz, 1H), 3.54–3.43 (m, 3H), 3.41 (s, 3H); ^{13}C NMR (101 MHz, $CDCl_3$) δ 166.06, 165.71, 165.33, 165.12, 165.07, 164.83, 138.87, 138.37, 138.18, 133.52, 133.49, 133.36, 133.28, 133.21, 133.18, 133.11, 129.81, 129.76, 129.73, 129.68, 129.65, 129.53, 129.28, 129.07, 128.70, 128.67, 128.63, 128.49, 128.45, 128.41, 128.35, 128.31, 128.26, 128.24, 128.14, 127.90, 127.83, 127.51, 127.44, 127.32, 101.42, 100.72, 98.19, 81.86, 79.69, 75.46, 74.49, 74.40, 73.44, 72.73, 72.70, 72.30, 72.03, 71.74, 69.62, 69.48, 69.36, 68.47, 67.52, 62.95, 55.35; HRMS (ESI) calcd for $C_{89}H_{80}O_{23}Na$ $[M+Na]^+$ 1539.4983, found 1539.4996.

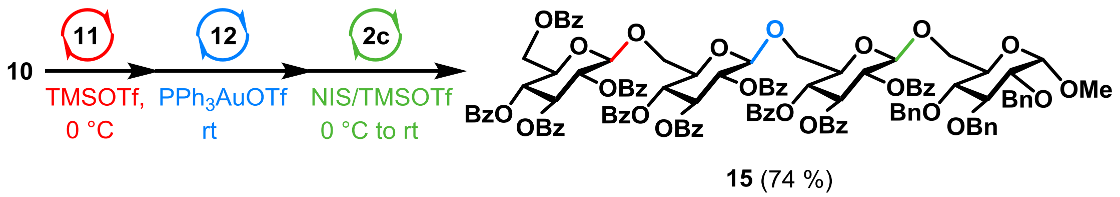

Supplementary Figure 12. One-pot synthesis of tetrasaccharide **15**.

A suspension of glucosyl trichloroacetimidate **10**[50] (73 mg, 0.084 mmol), glucosyl ABz acceptor **11**[26] (50 mg, 0.076 mmol), and activated 4Å MS (200 mg) in dry CH_2Cl_2 (1.5 mL) was stirred at room temperature for 30 min and was then cooled to 0 ℃. A solution of TMSOTf in CH_2Cl_2 (0.2 mL, 13.5μL in 1 mL CH_2Cl_2, 0.0152mmol) was added to the mixture dropwise. After being stirred at 0 ℃ for another 1.5 h, the reaction mixture was warmed to room temperature, to which glucosyl acceptor **12** (48.0 mg, 0.069 mmol) and a freshly prepared solution of PPh_3AuOTf in CH_2Cl_2 (1 mL, 0.0138M) were added successively. The resulting mixture was stirred at room temperature for another 1.5 h, then acceptor **2c**[51] (29 mg, 0.065 mmol), NIS (22 mg, 0.0975 mmol) and TMSOTf (0.1 mL, 12 μL in 1 mL CH_2Cl_2, 0.0065 mmol) were added successively at 0 ℃. The resulting mixture was stirred for 1.5 h, then was quenched with Et_3N, filtered with Celite and concentrated in vacuo. The residue was purified by flash column chromatography (EtOAc/Petroleum ether =1/2) to give **15**[26] (96 mg, 74 %) as a white foam: $[\alpha]_D^{25}$ = -9.9 (c 0.37, $CHCl_3$); ^{1}H NMR (400 MHz, $CDCl_3$) δ 8.08 (d, J = 7.6 Hz, 4H), 8.04–7.89 (m, 11H), 7.86 (t, J = 8.9 Hz, 4H), 7.81 (s, 2H), 7.61–7.26 (m, 42H), 7.26–7.13 (m, 7H), 7.08 (d, J = 6.7 Hz, 2H), 6.19 (t, J = 9.7 Hz, 1H), 5.89 (t, J = 9.6 Hz, 1H), 5.78–5.52 (m, 5H), 5.30 (t, J = 8.8 Hz, 1H), 5.17 (t, J = 8.7 Hz, 2H), 4.96 (d, J = 11.0 Hz, 1H), 4.83–4.58 (m, 8H), 4.52 (d, J = 11.6 Hz, 2H), 4.33 (t, J = 11.6 Hz, 2H), 4.16–3.99 (m, 3H), 3.90 (dd, J = 20.7, 9.2 Hz, 4H), 3.82–3.69 (m, 1H), 3.61 (d, J = 10.1 Hz, 1H), 3.53 (dd, J = 11.0, 3.4 Hz, 1H), 3.45 (d, J = 9.6 Hz, 2H), 3.37 (s, 3H); ^{13}C NMR (101 MHz, $CDCl_3$) δ 166.15, 165.83, 165.76, 165.62, 165.50, 165.33, 165.26, 164.96, 164.92, 138.94, 138.44, 138.21, 133.52, 133.33, 133.24, 133.20, 133.15, 133.07, 130.03, 129.93, 129.85, 129.82, 129.77, 129.73, 129.69, 129.66,

129.41, 129.33, 129.04, 128.92, 128.88, 128.85, 128.81, 128.72, 128.57, 128.53, 128.48, 128.46, 128.40, 128.38, 128.35, 128.33, 128.29, 128.24, 128.17, 128.14, 128.01, 127.93, 127.86, 127.61, 127.48, 127.42, 101.30, 101.11, 100.90, 98.09, 81.92, 79.88, 77.37, 75.48, 74.57, 74.31, 73.79, 73.43, 72.91, 72.75, 72.61, 72.26, 72.12, 71.97, 71.84, 70.65, 69.70, 69.60, 69.55, 68.75, 68.14, 67.77, 63.27, 55.31; HRMS (ESI) calcd for $C_{116}H_{102}O_{31}Na$ $[M+Na]^+$ 2013.6297, found 2013.6313.

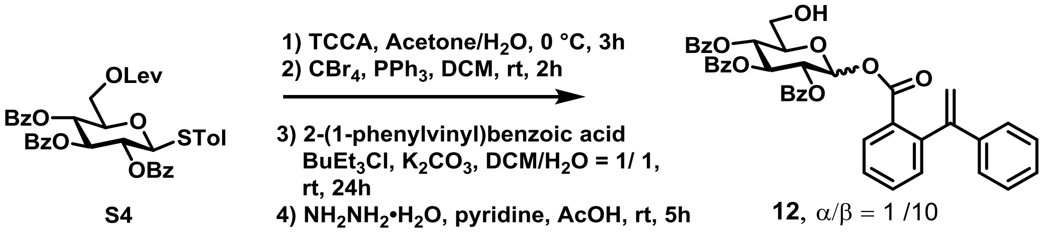

Supplementary Figure 13. Synthesis of compound **12**.

To a solution of compound **S4**[52] (1.75 g, 2.51 mmol) in Acetone (50 mL) /H_2O (12.5 mL) was added Trichloroisocyanuric acid (812 mg, 2.51 mmol) at 0 ℃. The mixture was stirred for 3 h at 0 ℃. Then EtOAc was added to this mixture and washed sequentially with saturated aqueous $NaHCO_3$, H_2O, and brine. The organic phase was dried by Na_2SO_4 and concentrated *in vacuo*. Purification by flash chromatography (EtOAc/Petroleum ether =1/3) afforded a syrup (1.37 g, 93%). A solution of the above syrup (352 mg, 0.597 mmol), CBr_4 (990 mg, 2.98 mmol), and PPh_3 (781 mg, 2.98 mmol) in CH_2Cl_2 (6 mL) was stirred at room temperature for 2 h. Then the mixture was diluted with CH_2Cl_2 and washed sequentially with saturated aqueous $NaHCO_3$ and brine. The organic phase was dried by Na_2SO_4 and concentrated *in vacuo*. The resulting residue was purified by flash chromatography (EtOAc/Petroleum ether =1/3) afforded a white foam (304 mg, 78%). And then, to a solution of above product (245 mg, 0.375 mmol) and 2-(1-phenylvinyl) benzoic acid **6** (109 mg, 0.488 mmol) in CH_2Cl_2 /H_2O = 1/1 (3.8 mL) was added $BnNEt_3Cl$ (17 mg, 0.075 mmol) and K_2CO_3 (257 mg, 1.86 mmol). The reaction mixture was stirred at room temperature for 24 h before diluted with brine and extracted with CH_2Cl_2. The organic phase was dried by

Na_2SO_4 and concentrated *in vacuo*. The resulting residue was purified by flash chromatography (EtOAc/Petroleum ether =1/2.5) afforded the intermediate (242 mg, 81%) as a white foam. The above intermediate (227 mg, 0.285 mmol) was then dissolved in pyridine (1.8 mL) and AcOH (1.2 mL), and hydrazine hydrate (7 μL, 0.142 mmol) was added dropwise. After stirring at room temperature for 5 h, the reaction mixture was quenched with acetone. The solution was concentrated *in vacuo* and diluted with CH_2Cl_2, washed sequentially with 1 N HCl and brine. The organic phase was dried by Na_2SO_4 and concentrated *in vacuo*. The resulting residue was purified by flash chromatography (EtOAc/Petroleum ether =1/2.5) afforded **12** (183 mg, 94%) as a white foam. The β anomer: $[\alpha]_D^{25}$ = 3.34 (*c* 0.23, $CHCl_3$); ^1H NMR (400 MHz, $CDCl_3$) δ 7.93 (d, *J* = 7.8 Hz, 2H), 7.90–7.80 (m, 6H), 7.56–7.47 (m, 4H), 7.45–7.24 (m, 12H), 5.97 (d, *J* = 8.3 Hz, 1H), 5.91 (d, *J* = 9.7 Hz, 1H), 5.73–5.62 (m, 2H), 5.49 (t, *J* = 9.7 Hz, 1H), 5.13 (s, 1H), 3.90–3.81 (m, 1H), 3.81–3.71 (m, 2H), 3.61 (dd, *J* = 13.0, 4.4 Hz, 1H), 2.30 (s, 1H); ^{13}C NMR (101 MHz, $CDCl_3$) δ 165.94, 165.67, 164.92, 164.61, 149.00, 143.89, 140.47, 133.75, 133.42, 133.33, 132.75, 131.65, 130.62, 129.95, 129.83, 129.75, 128.79, 128.72, 128.53, 128.45, 128.40, 128.34, 128.13, 127.82, 127.46, 126.55, 114.34, 92.33, 75.37, 72.78, 70.67, 68.96, 60.97. HRMS (ESI) calcd for $C_{41}H_{32}O_9Na$ $[M+Na]^+$ 721.2044, found 721.2048.

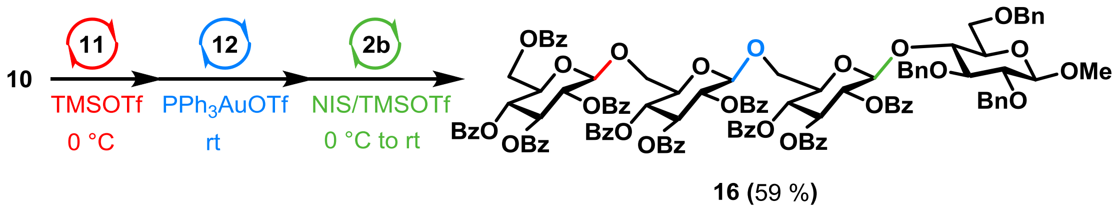

Supplementary Figure 14. One-pot synthesis of tetrasaccharide **16**.

A suspension of glucosyl trichloroacetimidate **10** (53 mg, 0.072 mmol), glucosyl ABz acceptor **11** (40 mg, 0.061 mmol), and activated 4Å MS (170 mg) in dry CH_2Cl_2 (1.3 mL) was stirred at room temperature for 30 min and was then cooled to 0 ℃. A solution of TMSOTf in CH_2Cl_2 (0.2 mL, 11μL in 1 mL CH_2Cl_2, 0.0122 mmol) was

added to the mixture dropwise. After being stirred at 0 ℃ for another 1.5 h, the reaction mixture was warmed to room temperature, to which glucosyl acceptor **12** (38.0 mg, 0.055 mmol) and a freshly prepared solution of PPh_3AuOTf in CH_2Cl_2 (1 mL, 0.011M) were added successively. The resulting mixture was stirred at room temperature for another 1.5 h, then acceptor **2b** (23 mg, 0.05 mmol), NIS (17 mg, 0.075 mmol) and TMSOTf (0.1 mL, 9 μL in 1 mL CH_2Cl_2, 0.005 mmol) were added successively at 0 ℃. The resulting mixture was stirred for 1.5 h, then was quenched with Et_3N, filtered with Celite and concentrated in vacuo. The residue was purified by flash column chromatography (EtOAc/Petroleum ether =1/2) to give **16** (58 mg, 59 %) as a white foam: $[\alpha]_D^{25}$ = -25.4 (c 0.12, $CHCl_3$); ^1H NMR (600 MHz, $CDCl_3$) δ 8.02–7.97 (m, 6H), 7.95 (d, *J* = 7.7 Hz, 2H), 7.89 (d, *J* = 7.8 Hz, 2H), 7.85 (d, *J* = 7.8 Hz, 2H), 7.82–7.77 (m, 4H), 7.76 (d, *J* = 7.8 Hz, 2H), 7.68 (d, *J* = 7.8 Hz, 2H), 7.59–7.17 (m, 45H), 5.93 (t, *J* = 9.5 Hz, 1H), 5.70 (t, *J* = 9.7 Hz, 1H), 5.57 (td, *J* = 9.7, 5.5 Hz, 2H), 5.49 (t, *J* = 8.7 Hz, 1H), 5.37 (t, *J* = 9.0 Hz, 1H), 5.29 (t, *J* = 9.7 Hz, 1H), 5.15 (t, *J* = 8.9 Hz, 1H), 5.08 (t, *J* = 9.7 Hz, 1H), 5.04 (d, *J* = 11.9 Hz, 1H), 4.98 (d, *J* = 11.0 Hz, 1H), 4.92 (d, *J* = 7.8 Hz, 1H), 4.89 (d, *J* = 11.0 Hz, 1H), 4.86–4.80 (m, 2H), 4.75 (d, *J* = 8.1 Hz, 1H), 4.56 (dd, *J* = 12.2, 3.3 Hz, 1H), 4.43 (dd, *J* = 12.1, 5.2 Hz, 1H), 4.31 (d, *J* = 12.1 Hz, 1H), 4.24 (d, *J* = 7.3 Hz, 1H), 4.18–4.10 (m, 2H), 3.94 (t, *J* = 9.2 Hz, 1H), 3.85 (d, *J* = 10.8 Hz, 1H), 3.79 (d, *J* = 9.0 Hz, 1H), 3.67–3.56 (m, 4H), 3.55 (s, 3H), 3.52–3.44 (m, 3H), 3.38 (t, *J* = 8.1 Hz, 1H), 3.19 (d, *J* = 9.4 Hz, 1H); ^{13}C NMR (151 MHz, $CDCl_3$) δ 166.21, 165.83, 165.79, 165.51, 165.46, 165.34, 165.32, 165.21, 165.14, 164.88, 139.49, 139.17, 138.07, 133.52, 133.44, 133.38, 133.31, 133.28, 133.22, 133.18, 133.08, 133.02, 133.01, 130.24, 130.08, 130.06, 129.97, 129.89, 129.87, 129.84, 129.83, 129.77, 129.74, 129.49, 129.31, 129.26, 129.24, 129.18, 129.13, 129.06, 129.02, 129.00, 128.82, 128.67, 128.62, 128.56, 128.49, 128.45, 128.40, 128.38, 128.36, 128.34, 128.25, 128.22, 128.17, 128.08, 127.58, 127.48, 104.86, 101.08, 100.23, 100.17, 83.50, 81.79, 75.97, 74.99, 74.40, 74.06, 73.43, 73.15, 73.02, 72.82, 72.33, 72.24, 72.14, 71.93, 70.46, 69.86, 69.79, 67.99, 67.69, 67.23, 63.34, 57.03; HRMS (ESI) calcd for $C_{116}H_{102}O_{31}Na$ $[M+Na]^+$

2013.6297, found 2013.6247.

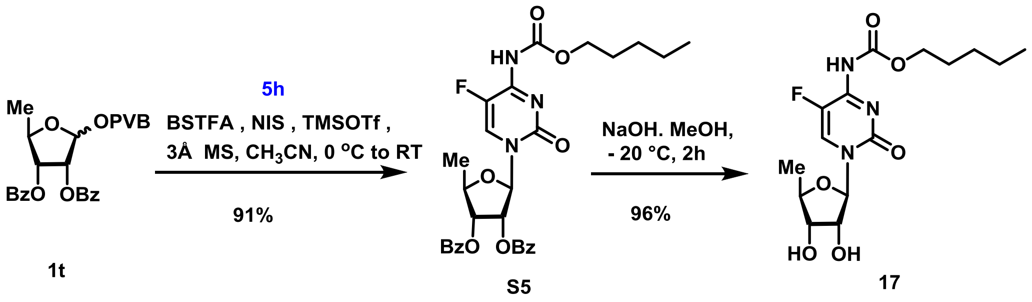

Supplementary Figure 15. Synthesis of Capecitabine **17**

Glycosylation of **1t** (50 mg, 0.091 mmol) with **5h** (44 mg, 0.182 mmol) according to **General procedure C** afforded **S5** (46 mg, 91%) as a light yellow liquid: $[\alpha]_D^{25}$ = -42.7 (c 0.18, $CHCl_3$); ^{1}H NMR (400 MHz, $CDCl_3$) δ 12.03 (s, 1H), 8.01–7.87 (m, 4H), 7.59–7.50 (m, 3H), 7.43–7.32 (m, 4H), 6.19 (d, *J* = 5.3 Hz, 1H), 5.63 (t, *J* = 5.6 Hz, 1H), 5.43 (t, *J* = 5.6 Hz, 1H), 4.57–4.43 (m, 1H), 4.17 (t, *J* = 6.8 Hz, 2H), 1.74–1.67 (m, 2H), 1.59 (d, *J* = 6.4 Hz, 3H), 1.36 (m, 4H), 0.90 (t, *J* = 6.8 Hz, 3H); ^{13}C NMR (100 MHz, $CDCl_3$) δ 165.36, 165.29, 163.47, 153.40, 153.22, 146.23, 133.75, 133.67, 129.88, 129.77, 128.76, 128.53, 128.49, 128.38, 124.13, 123.79, 88.55, 78.83, 74.66, 73.75, 66.70, 28.25, 27.93, 22.32, 18.88; HRMS (ESI) calcd for $C_{29}H_{29}N_3O_8F$ $[M-H]^-$ 566.1944, found 566.1944.

To a solution of **S5** (27 mg, 0.048 mmol) in 1 mL CH_3OH was slowly added NaOH (aq.) (2.1 mg NaOH in 0.5 mL H_2O) at - 20 ℃. The solution was stirred for another 2h under the same temperature. Then it was quenched with 3 N HCl, the mixture was concentrated *in vacuo*. Purification by silica gel column chromatography (CH_2Cl_2/CH_3OH =10/1) afforded Capecitabine **17**[28] (16 mg, 96%) as a light yellow solid. $[\alpha]_D^{25}$ = 81.01 (c 0.18, CH_3OH); ^{1}H NMR (400 MHz, Methanol-d_4) δ 7.97 (d, *J* = 6.4 Hz, 1H), 5.76–5.67 (m, 1H), 4.24–4.15 (m, 3H), 4.08 (d, *J* = 6.6 Hz, 1H), 3.73 (dd, *J* = 7.2, 5.2 Hz, 1H), 1.71 (dd, *J* = 9.3, 5.0 Hz, 2H), 1.43 (d, *J* = 6.3 Hz, 3H), 1.41–1.36 (m, 4H), 0.96–0.90 (m, 3H); ^{13}C NMR (100 MHz, Methanol-d_4) δ 154.12, 128.92, 92.29, 79.58, 74.64, 74.52, 66.00, 28.08, 27.65, 21.98, 17.03, 12.90; HRMS

(ESI) calcd for $C_{15}H_{22}N_3O_6FNa$ $[M+Na]^+$ 382.1385, found 382.1384.

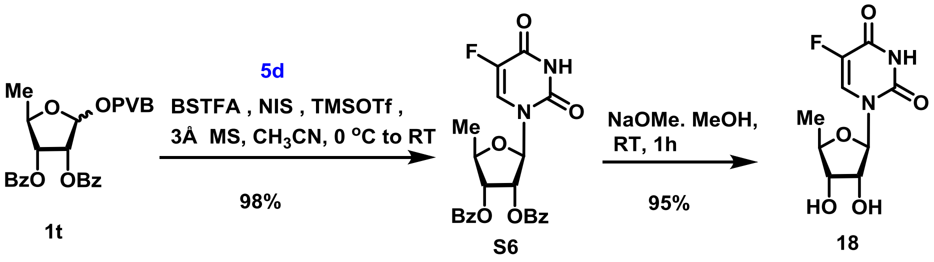

Supplementary Figure 16. Synthesis of Doxifluridine **18**

Glycosylation of **1t** (49 mg, 0.089 mmol) with **5d** (23 mg, 0.179 mmol) according to **General procedure C** afforded **S6** (39 mg, 98%) as a colorless liquid: ^{1}H NMR (400 MHz, Chloroform-*d*) δ 9.10 (s, 1H), 7.98 (d, *J* = 7.7 Hz, 2H), 7.90 (d, *J* = 7.8 Hz, 2H), 7.50–7.6 (m, 2H), 7.49–7.31 (m, 5H), 6.17 (d, *J* = 5.3 Hz, 1H), 5.63 (t, *J* = 5.7 Hz, 1H), 5.42 (t, *J* = 5.7 Hz, 1H), 4.54–4.40 (m, 1H), 1.59 (d, *J* = 6.4 Hz, 3H); ^{13}C NMR (101 MHz, $CDCl_3$) δ 165.41, 165.36, 156.60, 156.34, 148.69, 142.05, 139.67, 133.78, 133.68, 129.89, 129.80, 128.76, 128.54, 128.51, 128.36, 124.17, 123.83, 88.39, 78.67, 74.64, 73.65, 18.86; HRMS (ESI) calcd for $C_{23}H_{19}N_2O_7FNa$ $[M+Na]^+$ 477.1069, found 477.1068.

To a solution of **S6** (37 mg, 0.081 mmol) in 0.8 mL CH_3OH was slowly added NaOMe (4.5 mg, 0.081mmol) at room temperature. The solution was stirred for another 1h under the same temperature. Then it was quenched with 3N HCl, the mixture was concentrated *in vacuo*. Purification by silica gel column chromatography (CH_2Cl_2/CH_3OH =10/1) afforded compound **18**[28] (19 mg, 95%) as a white solid. $[\alpha]_D^{25}$ = 4.59 (c 0.14, CH_3OH); ^{1}H NMR (400 MHz, Methanol-d_4) δ 7.73 (d, *J* = 6.5 Hz, 1H), 5.76 (dd, *J* = 4.0, 1.5 Hz, 1H), 4.16 (dd, *J* = 5.6, 4.1 Hz, 1H), 4.00 (p, *J* = 6.3 Hz, 1H), 3.79 (t, *J* = 5.8 Hz, 1H), 1.39 (d, *J* = 6.4 Hz, 3H); ^{13}C NMR (101 MHz, Methanol-d_4) δ 158.14, 157.88, 149.49, 141.65, 139.33, 124.93, 124.58, 90.37, 79.68, 74.48, 73.66, 17.36.; HRMS (ESI) calcd for $C_9H_{10}N_2O_5FNa$ $[M-H]^-$ 245.0579, found 245.0578.

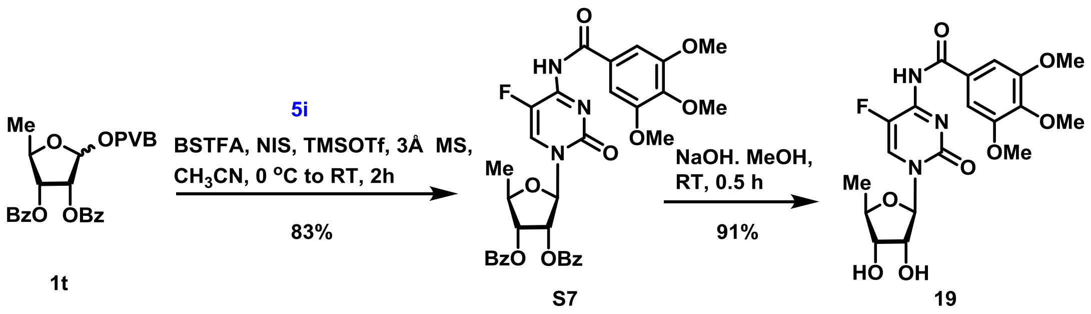

Supplementary Figure 17. Synthesis of Galocitabine **19**

Glycosylation of **1t** (54 mg, 0.098 mmol) with **5i** (64 mg, 0.197 mmol) according to **General procedure C** afforded **S7** (52 mg, 83%) as a light yellow liquid: $[\alpha]_D^{25}$ = -88.18 (c 0.14, $CHCl_3$); ^{1}H NMR (400 MHz, $CDCl_3$) δ 12.80 (s, 1H), 7.98 (d, J = 7.7 Hz, 2H), 7.90 (d, J = 7.7 Hz, 2H), 7.66–7.50 (m, 5H), 7.44–7.32 (m, 4H), 6.23 (d, *J* = 5.2 Hz, 1H), 5.67 (t, *J* = 5.6 Hz, 1H), 5.45 (t, *J* = 5.6 Hz, 1H), 4.57–4.44 (m, 1H), 3.93 (d, *J* = 2.1 Hz, 9H), 1.61 (d, *J* = 6.4 Hz, 3H); ^{13}C NMR (101 MHz, $CDCl_3$) δ 165.37, 165.34, 152.82, 152.16, 151.97, 146.66, 142.55, 141.48, 139.10, 133.78, 133.70, 131.18, 129.89, 129.78, 128.75, 128.55, 128.51, 128.37, 124.78, 124.44, 107.36, 88.58, 78.81, 74.66, 73.81, 60.94, 56.20, 18.90; HRMS (ESI) calcd for $C_{33}H_{29}N_3O_{10}F$ $[M-H]^-$ 646.1842, found 646.1843.

To a solution of **S7** (44 mg, 0.068 mmol) in 1.2 mL CH_3OH was slowly added NaOH (aq.) (5.5 mg NaOH in 0.6 mL H_2O) at room temperature. The solution was stirred for another 30 mins under the same temperature. Then it was quenched with 3N HCl, the mixture was concentrated *in vacuo*. Purification by silica gel column chromatography (CH_2Cl_2/CH_3OH =15/1) afforded **19**[28] (26 mg, 91%) as a white solid. $[\alpha]_D^{25}$ = 24.93 (c 0.11, CH_3OH); ^{1}H NMR (400 MHz, Methanol-d_4) δ 7.96 (d, *J* = 6.2 Hz, 1H), 7.40 (s, 2H), 5.77 (dd, *J* = 2.7, 1.2 Hz, 1H), 4.20 (dd, *J* = 5.4, 2.8 Hz, 1H), 4.13–4.07 (m, 1H), 3.90 (s, 6H), 3.83 (s, 3H), 3.79–3.75 (m, 1H), 1.44 (d, *J* = 6.3 Hz, 3H); ^{13}C NMR (101 MHz, Methanol-d_4) δ 152.89, 141.94, 129.61, 106.39, 91.98, 79.67, 74.54, 59.77, 55.30, 17.12; HRMS (ESI) calcd for $C_{19}H_{22}N_3O_8FNa$ $[M+Na]^+$ 462.1283, found 462.1281.

X-ray crystal structure data of compound 4. Single crystals suitable for X-ray studies were grown by slow evaporation of a solution of compound **4** in a mixture of petroleum ether and EtOAc at room temperature. The Xray data of compound **4** is deposited in the Cambridge Crystallographic Data Center with a number of CCDC 1939909.

Crystal data for compound **4**: $C_{15}H_{11}IO_2$, M = 350.14, a = 12.7649(3) Å, b = 11.7345(2) Å, c = 17.2336(3) Å, α = 90 °, β = 90 °, γ = 90 °, V = 2581.42(9) Å^3, T = 100.(2) K, space group $Pca21$, Z = 8, μ(Cu Kα) = 19.420 mm^{-1}, 15737 reflections measured, 4832 independent reflections (R_{int} = 0.0449). The final R_1 values were 0.0363 ($I > 2\sigma(I)$). The final $wR(F^2)$ values were 0.0929 ($I > 2\sigma(I)$). The final R_1 values were 0.0388 (all data). The final $wR(F^2)$ values were 0.0952 (all data). The goodness of fit on F^2 was 1.029. Flack parameter = 0.416(6).

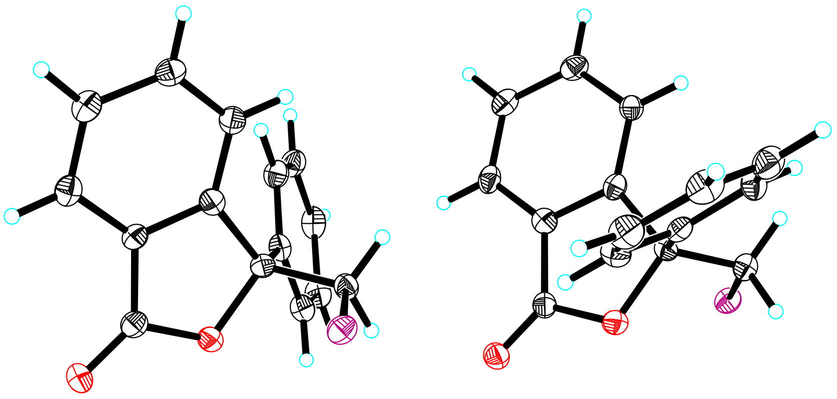

Supplementary Figure 18. X-ray crystal structure data of compound **4**. View of the molecules in an asymmetric unit. Displacement ellipsoids are drawn at the 30% probability level.

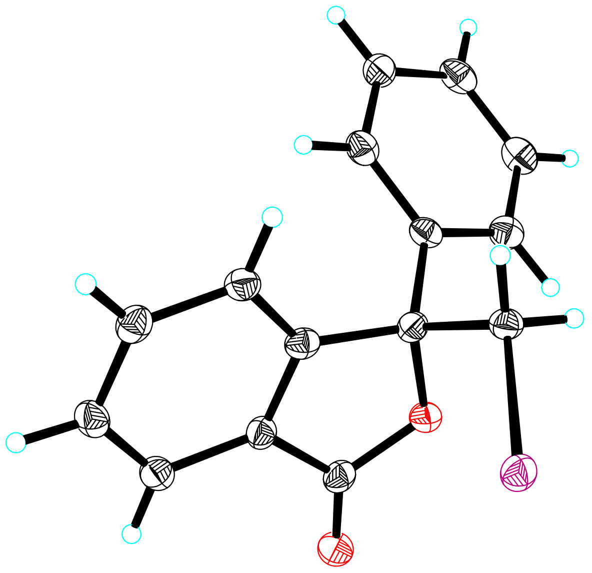

Supplementary Figure 19. X-ray crystal structure data of compound **4**. View of a molecule of compound **4** with the atom-labelling scheme. Displacement ellipsoids are drawn at the 30% probability level.

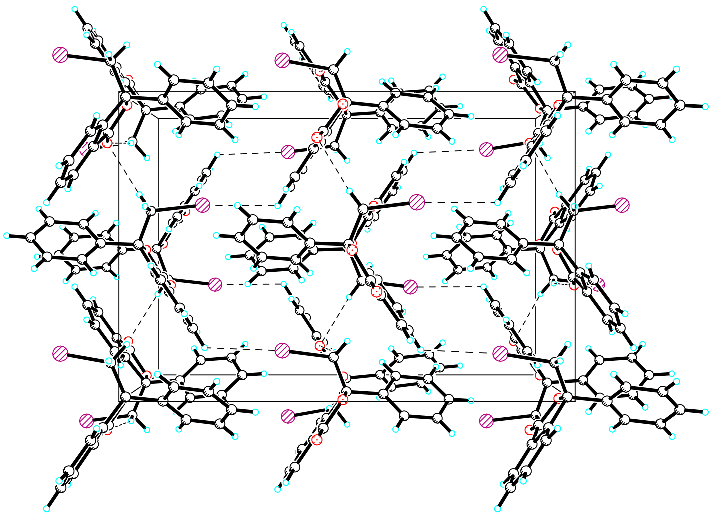

Supplementary Figure 20. X-ray crystal structure data of compound **4**. View of the pack drawing of compound **4**. Hydrogen-bonds are shown as dashed lines.

Supplementary Table 1. Crystal data and structure refinement for compound 4.

Identification code	CCDC 1939909
Empirical formula	$C_{15}H_{11}IO_2$
Formula weight	350.14
Temperature	100(2) K
Wavelength	1.54178 Å
Crystal system	Orthorhombic
Space group	$Pca2_1$
Unit cell dimensions	a = 12.7649(3) Å α= 90 °.<br>b = 11.7345(2) Å β= 90 °.<br>c = 17.2336(3) Åγ = 90 °.
Volume	2581.42(9) Å3
Z	8
Density (calculated)	1.802 Mg/m^3
Absorption coefficient	19.420 mm^{-1}
F(000)	1360
Crystal size	0.580 x 0.050 x 0.020 mm^3
Theta range for data collection	3.77 to 72.49 °
Index ranges	-15<=h<=12, -14<=k<=14, -21<=l<=21
Reflections collected	15737
Independent reflections	4832 [R(int) = 0.0449]
Completeness to theta = 72.49 °	99.8 %
Absorption correction	Semi-empirical from equivalents
Max. and min. transmission	0.70 and 0.12
Refinement method	Full-matrix least-squares on F^2
Data / restraints / parameters	4832 / 1 / 325
Goodness-of-fit on F^2	1.029
Final R indices [I>2sigma(I)]	R1 = 0.0363, wR2 = 0.0929
R indices (all data)	R1 = 0.0388, wR2 = 0.0952
Absolute structure parameter	0.416(6)
Largest diff. peak and hole	0.930 and -0.558 e.$Å^{-3}$

Supplementary Figures

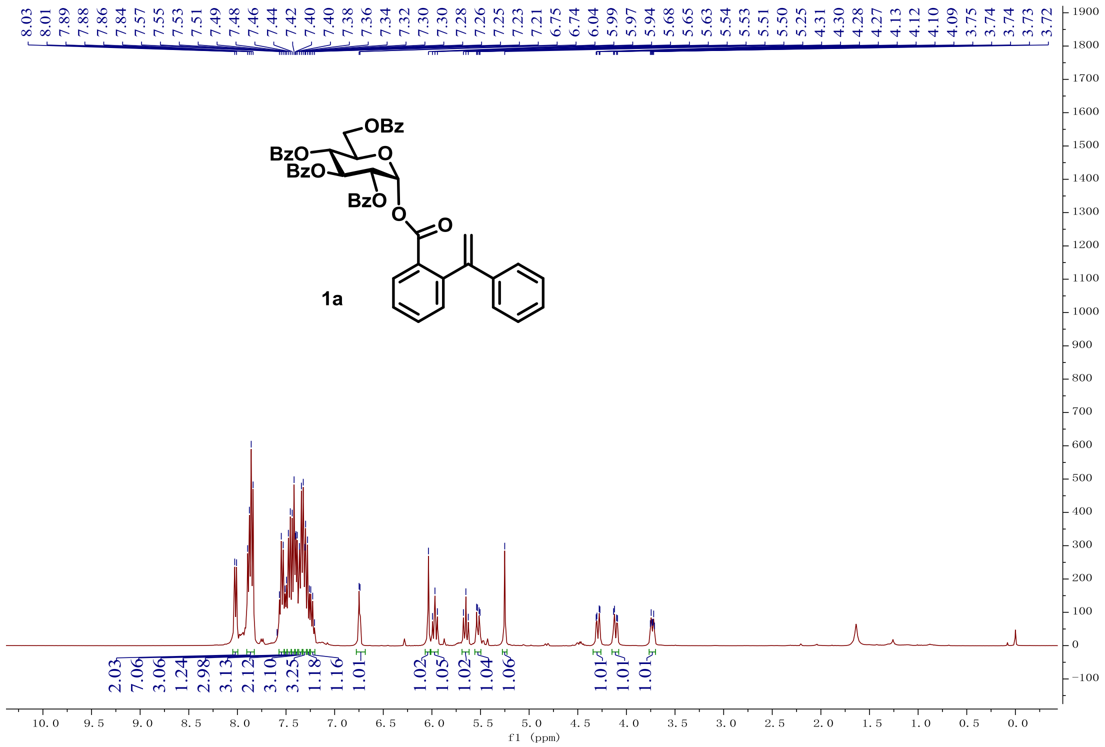

Supplementary Figure 21. ^{1}H NMR Spectrum of Compound **1a**

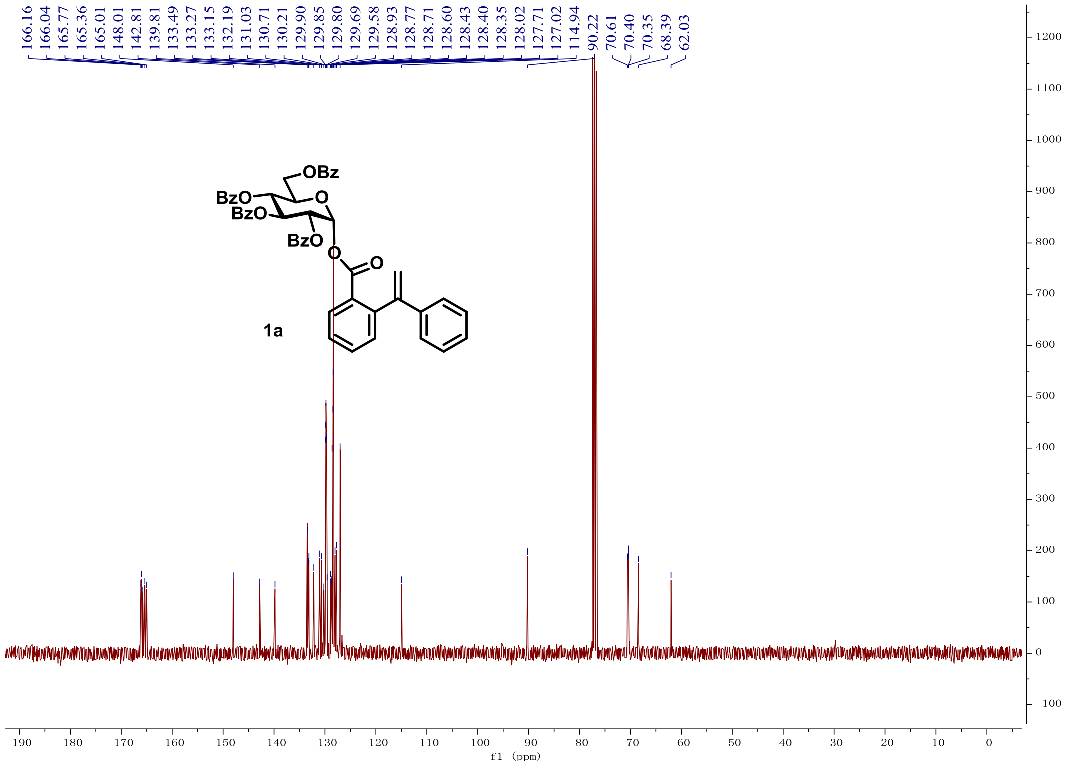

Supplementary Figure 22. ^{13}C NMR Spectrum of Compound **1a**

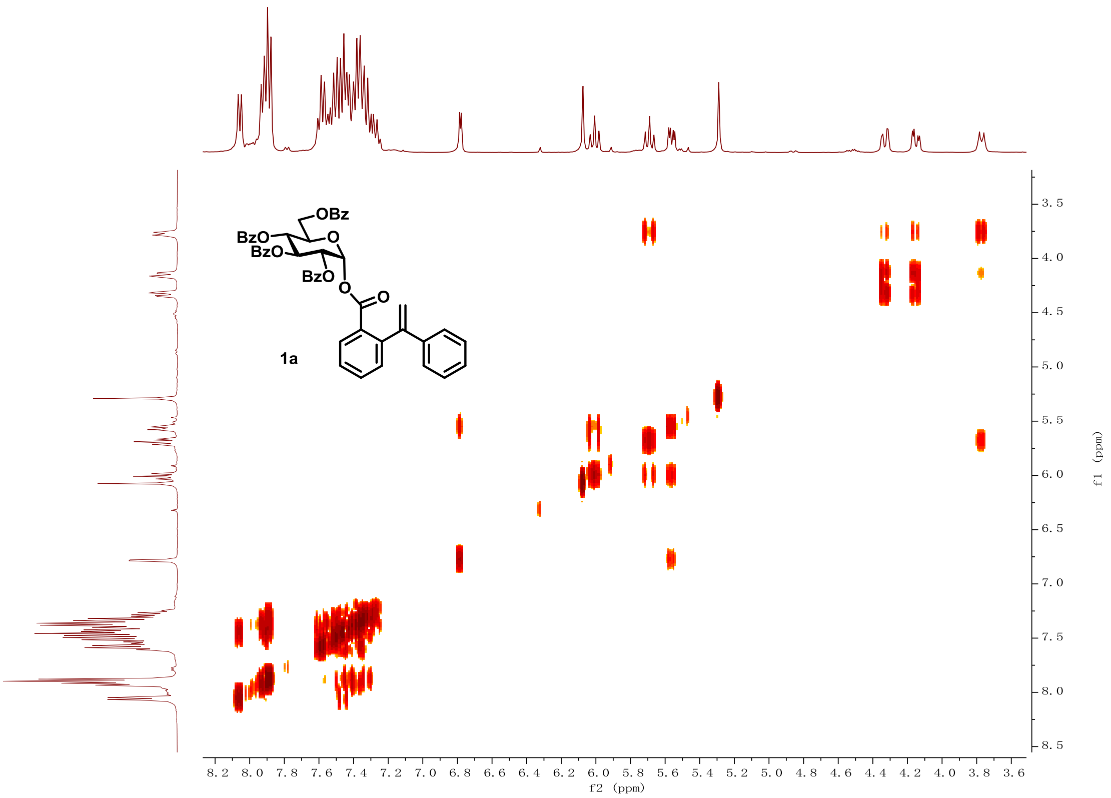

Supplementary Figure 23. COSY NMR Spectrum of Compound **1a**

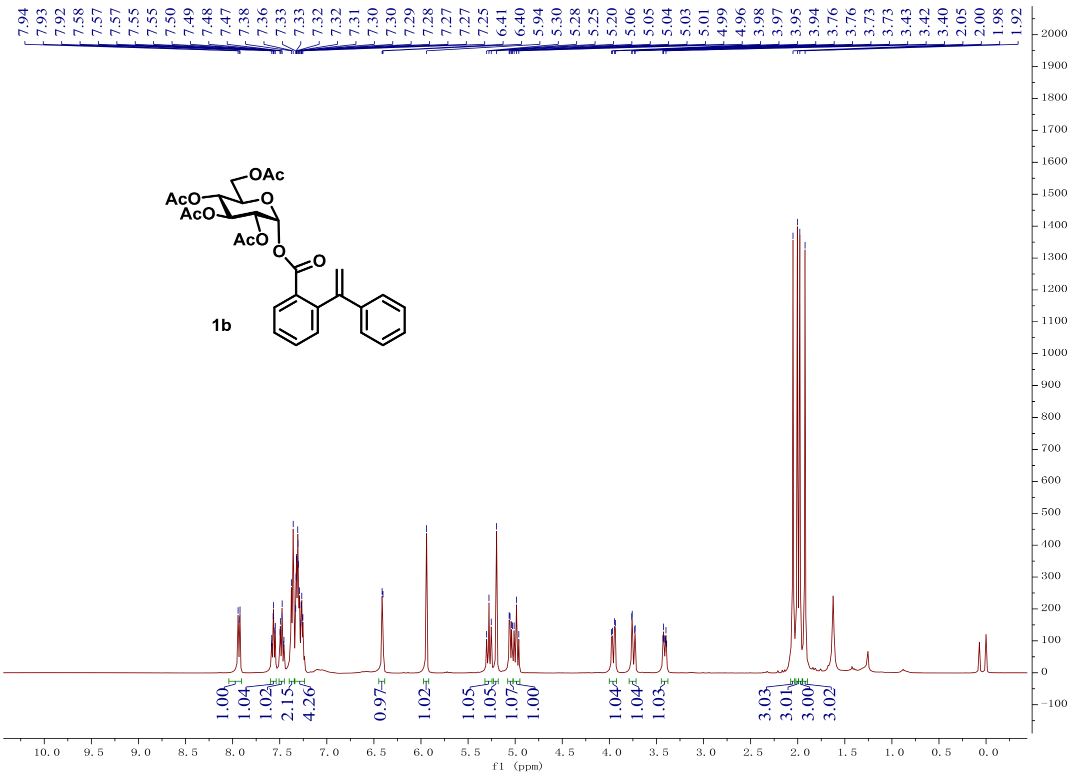

Supplementary Figure 24. ^{1}H NMR Spectrum of Compound **1b**

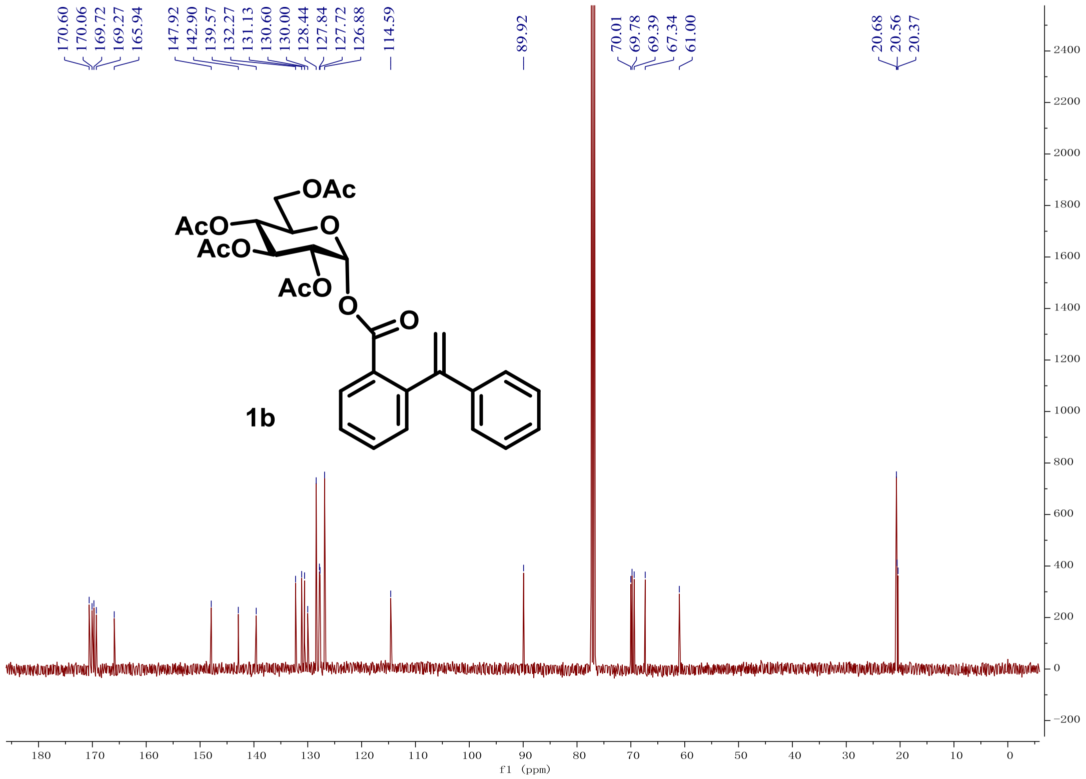

Supplementary Figure 25. ^{13}C NMR Spectrum of Compound **1b**

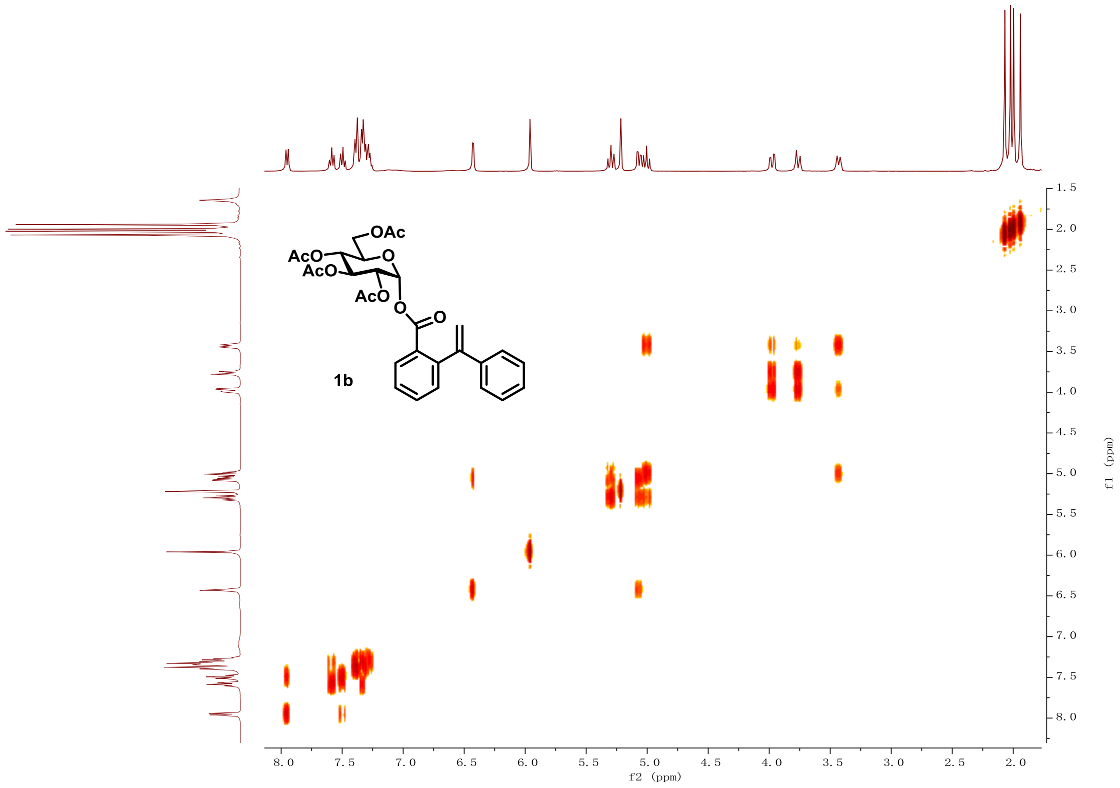

Supplementary Figure 26. COSY NMR Spectrum of Compound **1b**

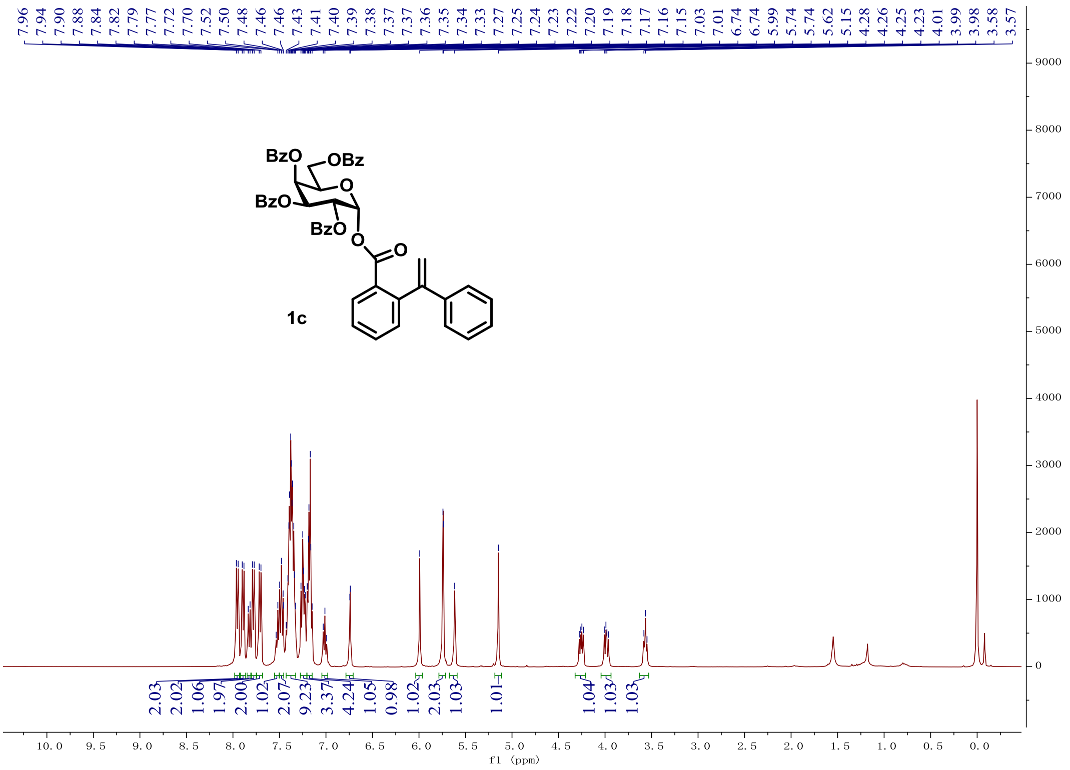

Supplementary Figure 27. ^{1}H NMR Spectrum of Compound **1c**

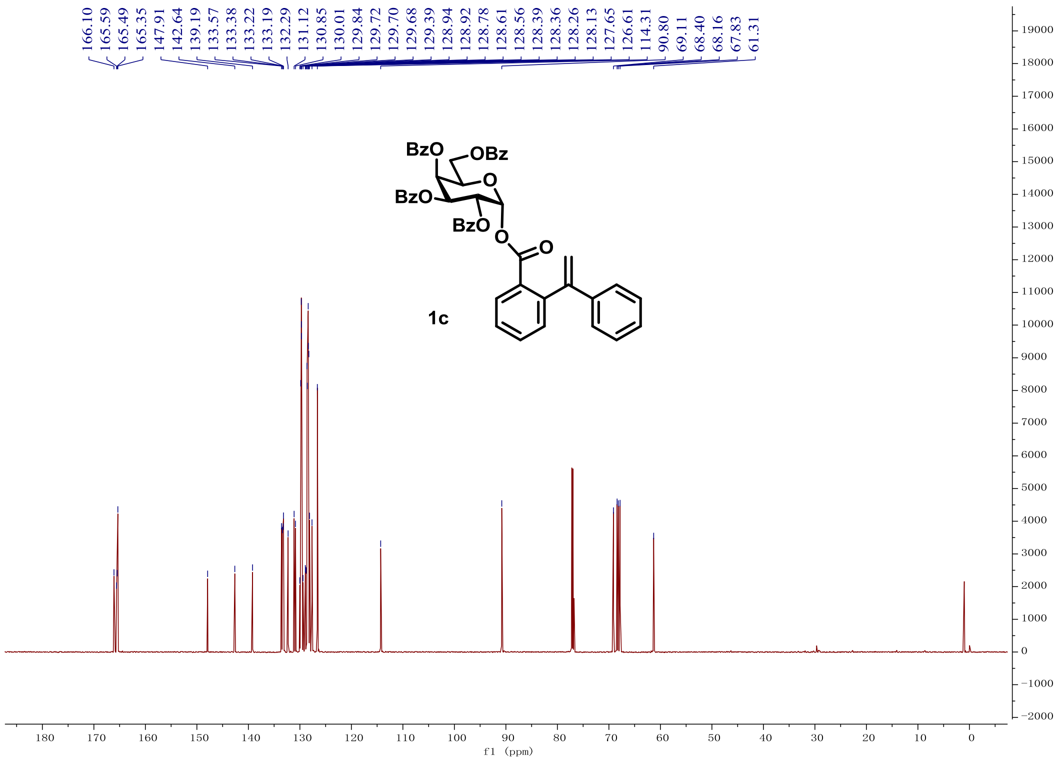

Supplementary Figure 28. ^{13}C NMR Spectrum of Compound **1c**

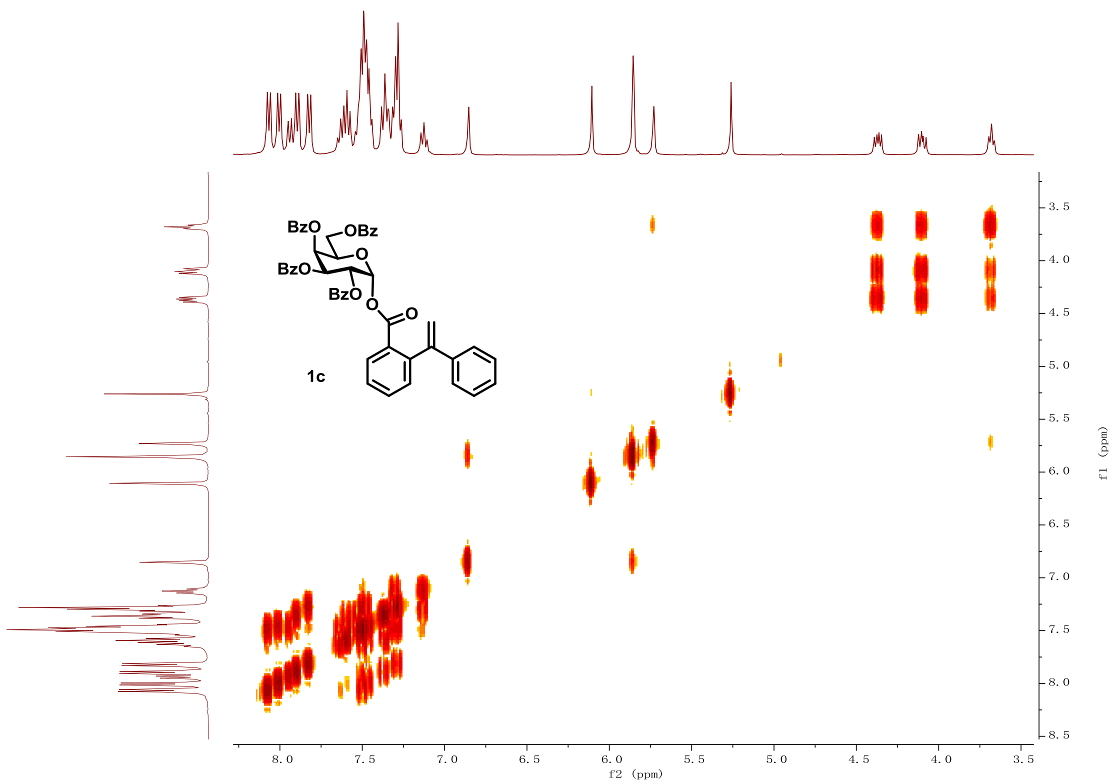

Supplementary Figure 29. COSY NMR Spectrum of Compound **1c**

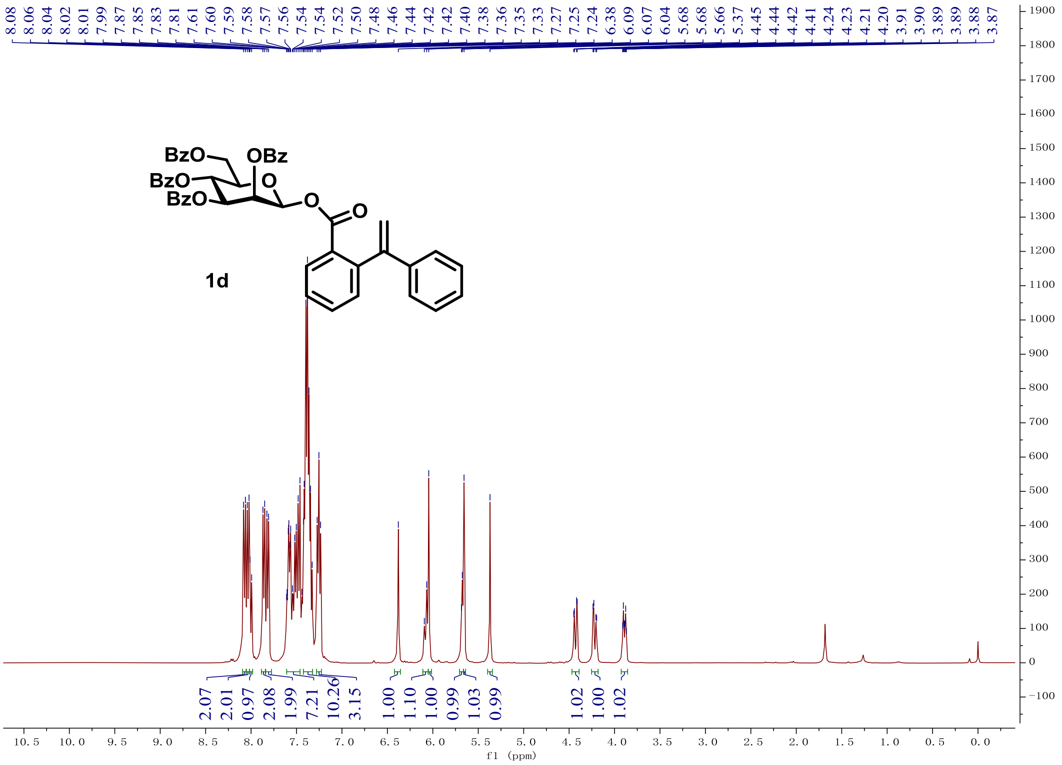

Supplementary Figure 30. ^{1}H NMR Spectrum of Compound **1d**

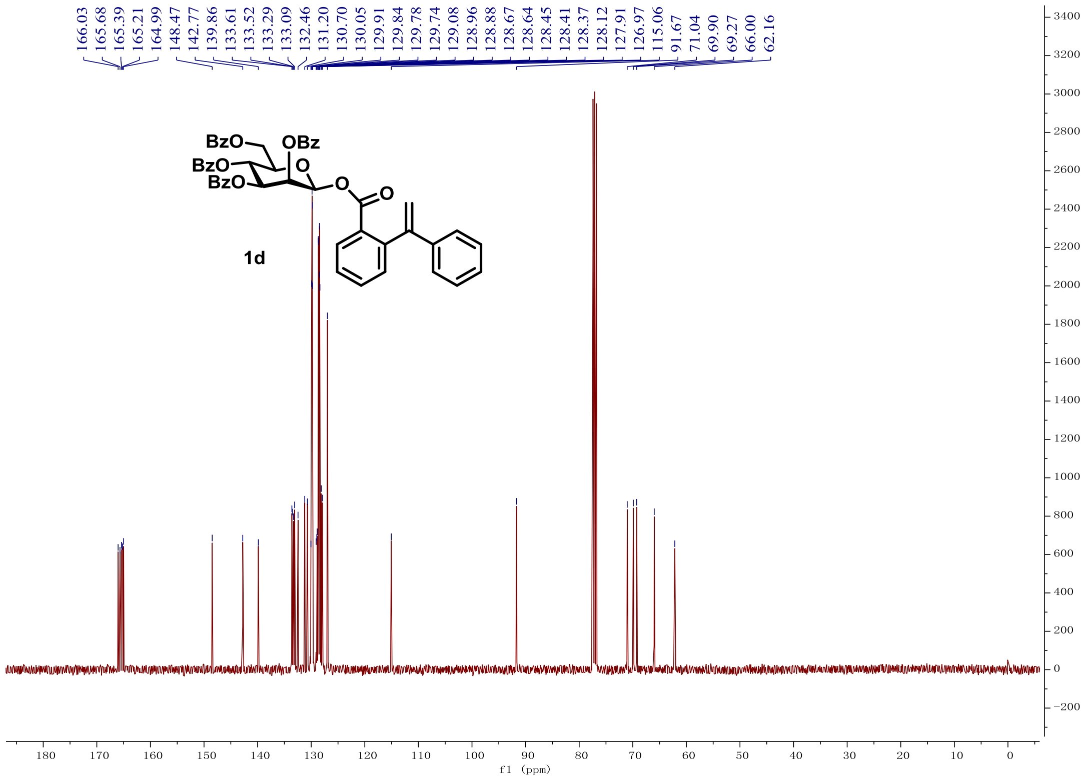

Supplementary Figure 31. ^{13}C NMR Spectrum of Compound **1d**

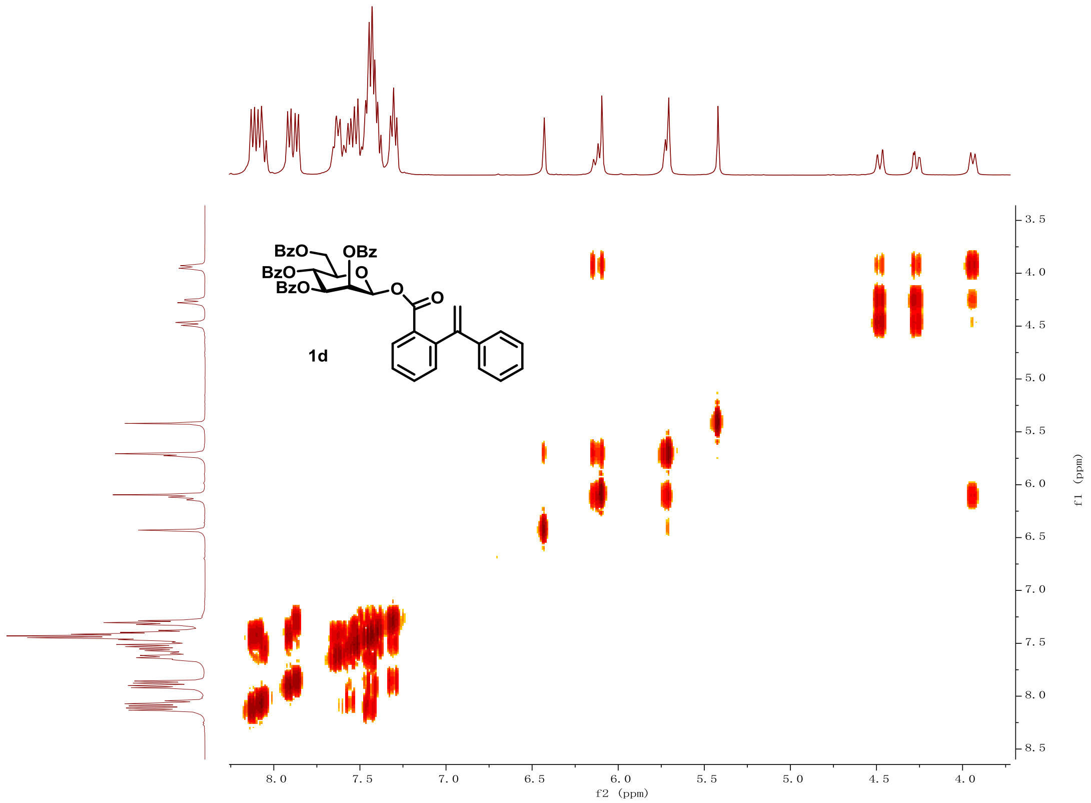

Supplementary Figure 32. COSY NMR Spectrum of Compound **1d**

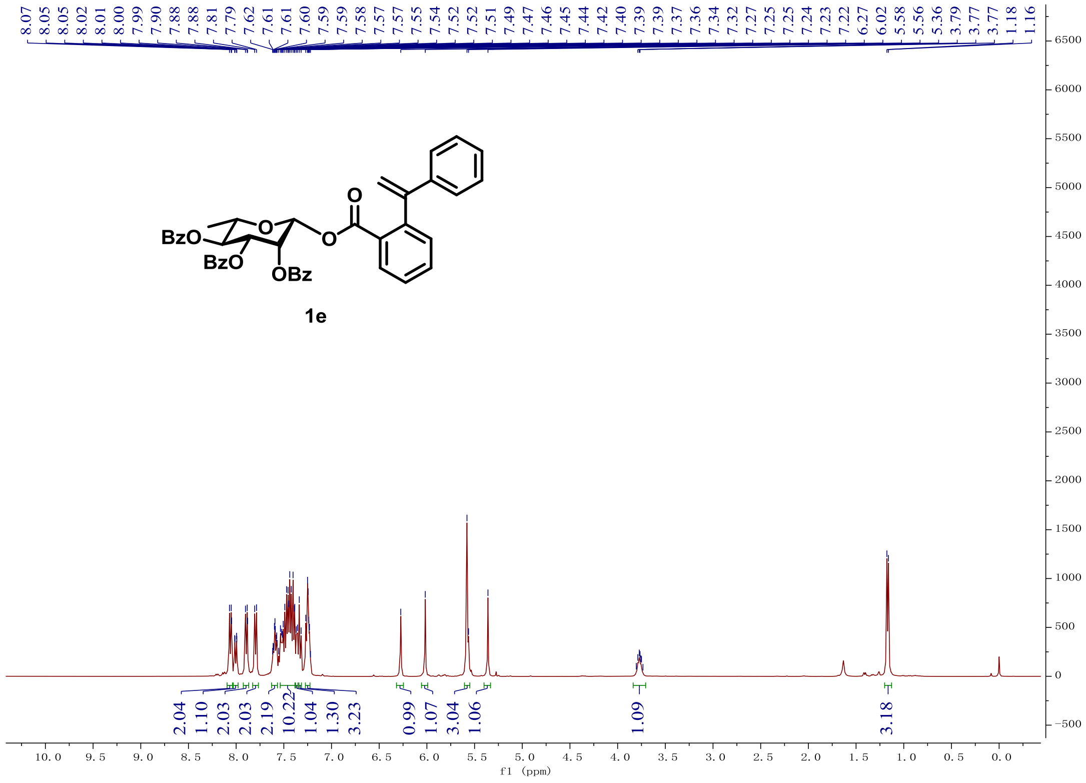

Supplementary Figure 33. ^{1}H NMR Spectrum of Compound **1e**

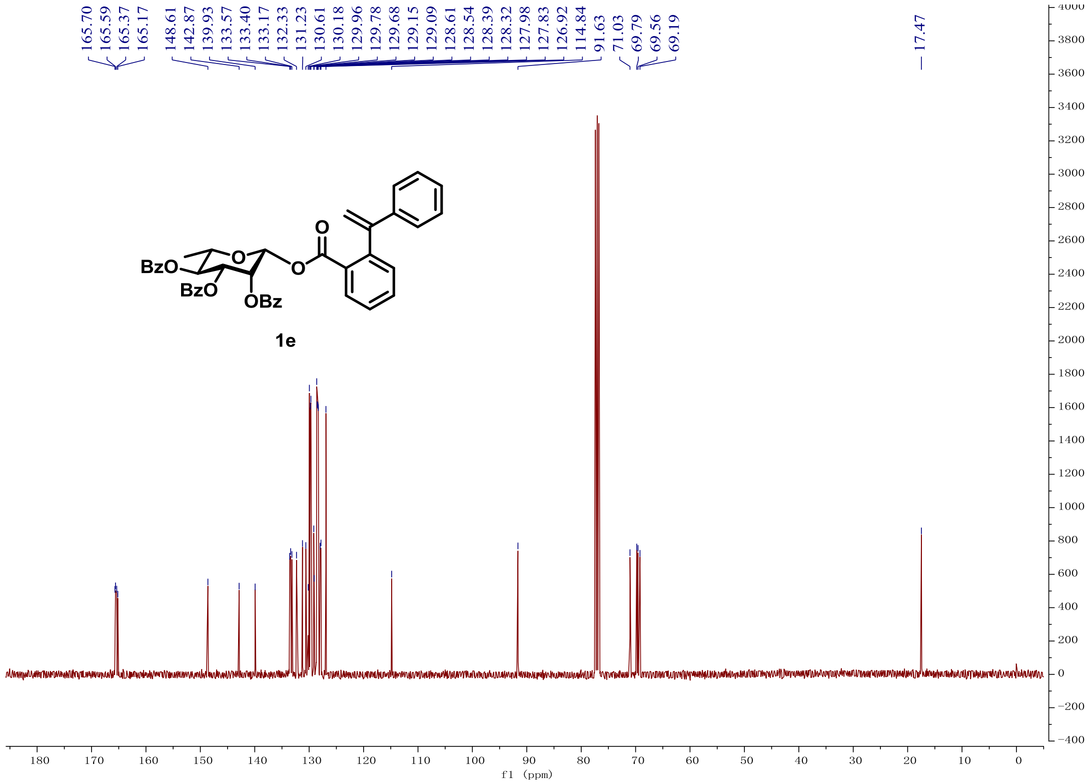

Supplementary Figure 34. ^{13}C NMR Spectrum of Compound **1e**

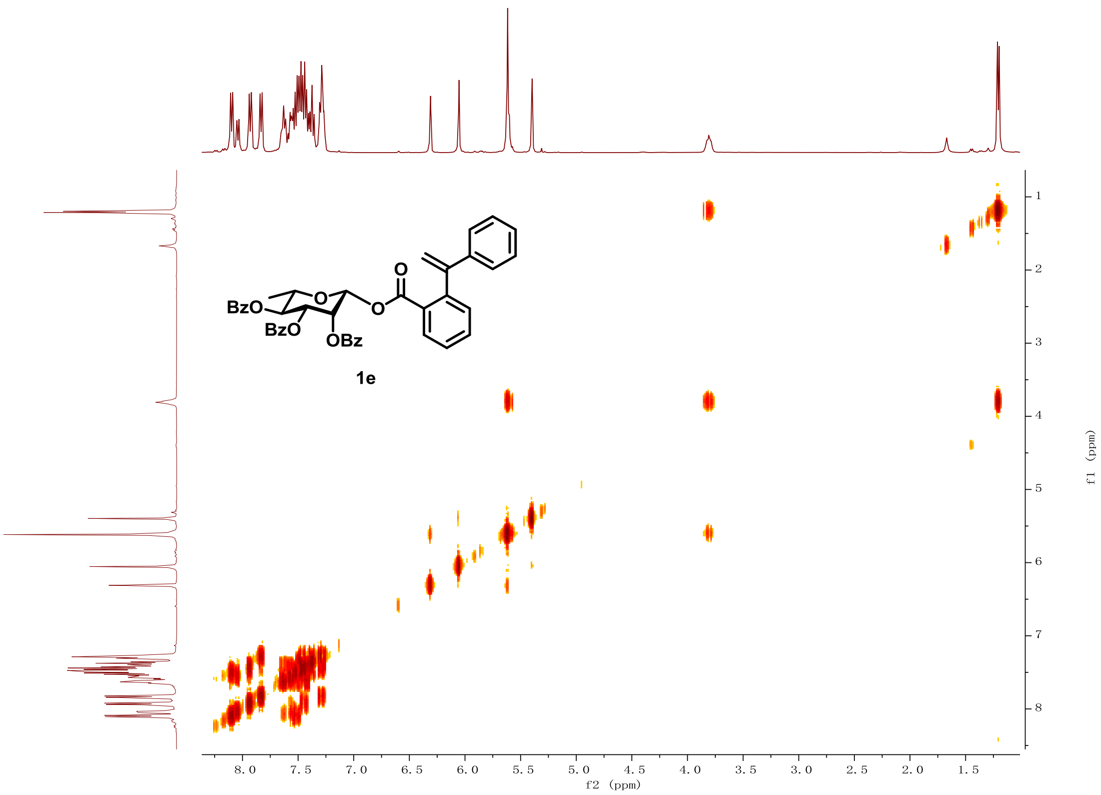

Supplementary Figure 35. COSY NMR Spectrum of Compound **1e**

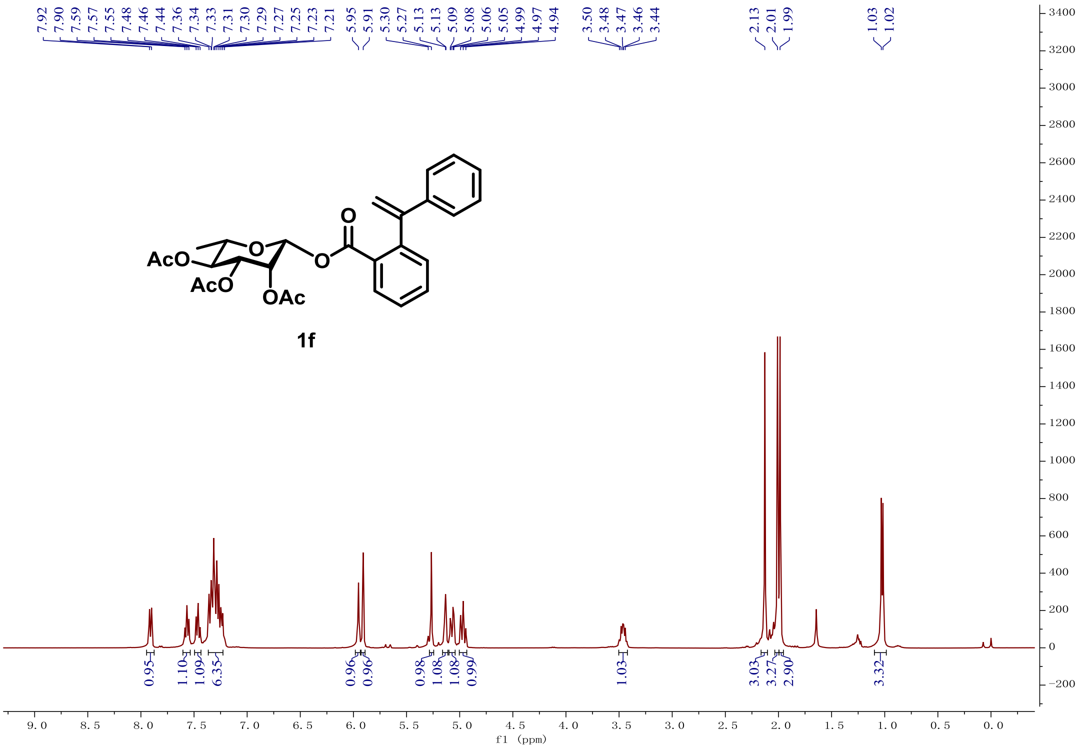

Supplementary Figure 36. ^{1}H NMR Spectrum of Compound **1f**

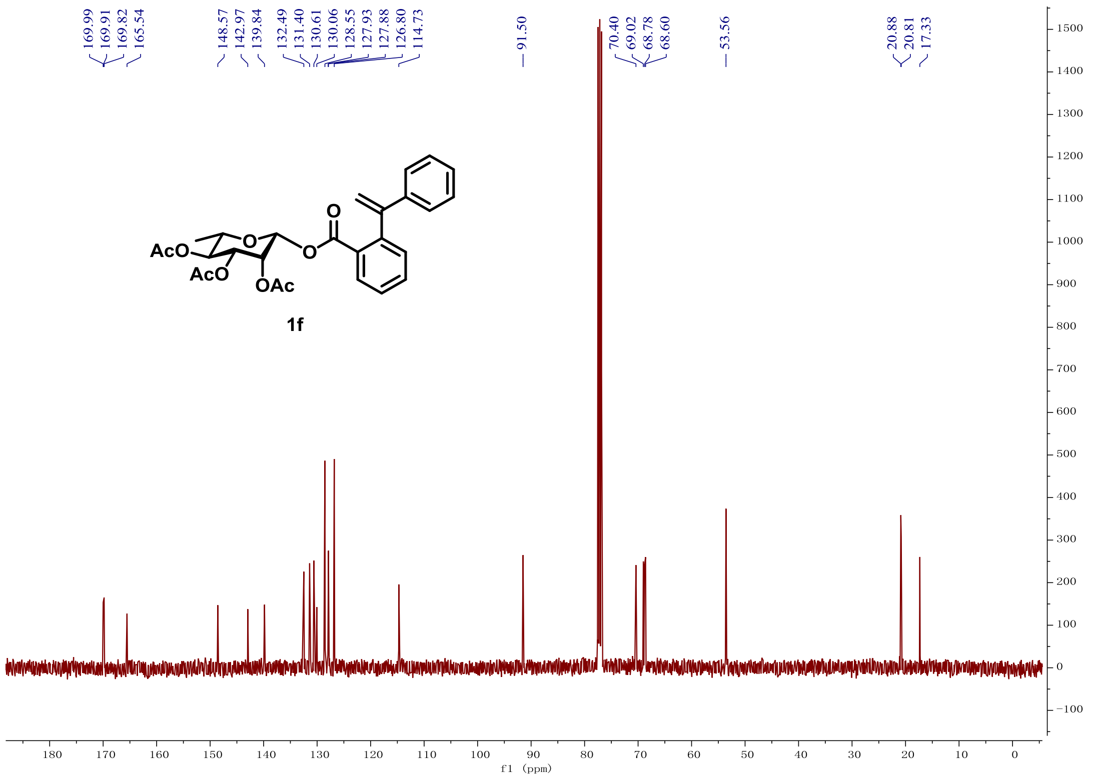

Supplementary Figure 37. ^{13}C NMR Spectrum of Compound **1f**

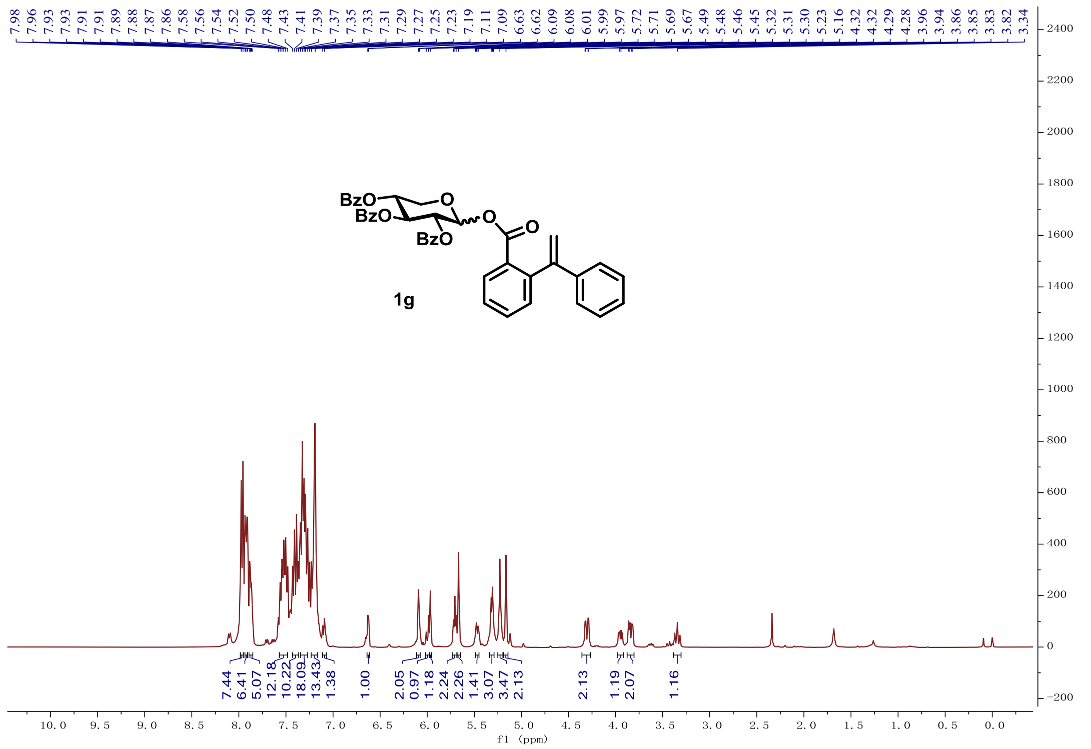

Supplementary Figure 38. ^{1}H NMR Spectrum of Compound **1g**

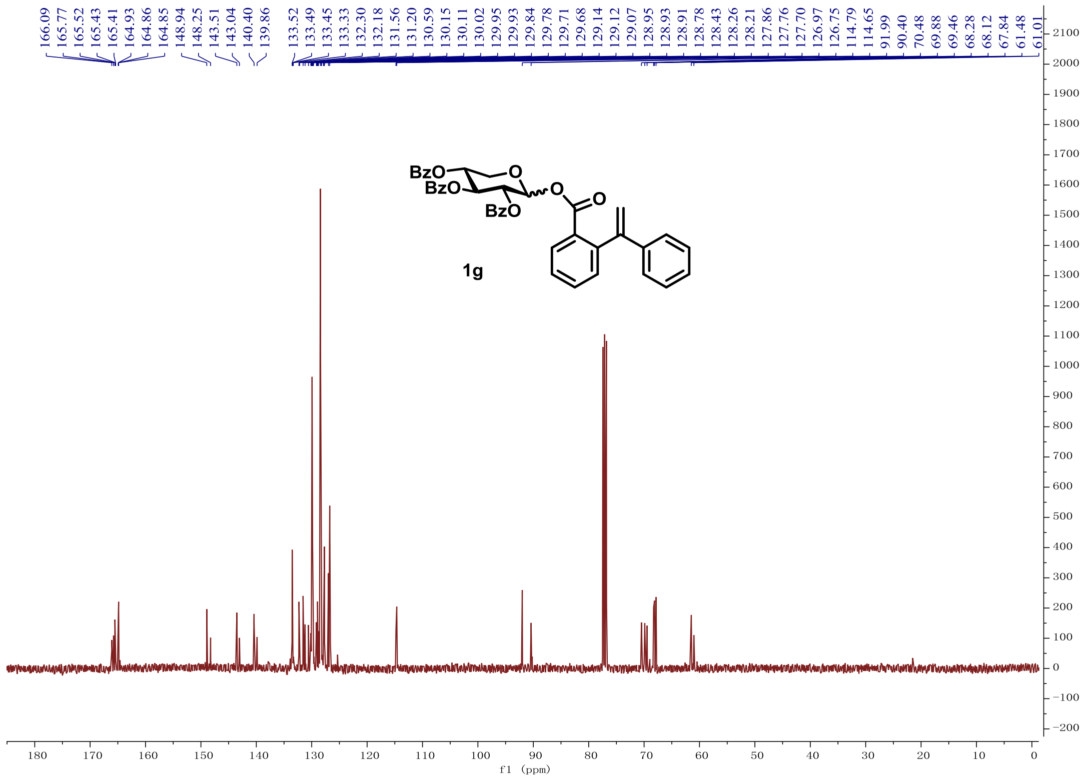

Supplementary Figure 39. ^{13}C NMR Spectrum of Compound **1g**

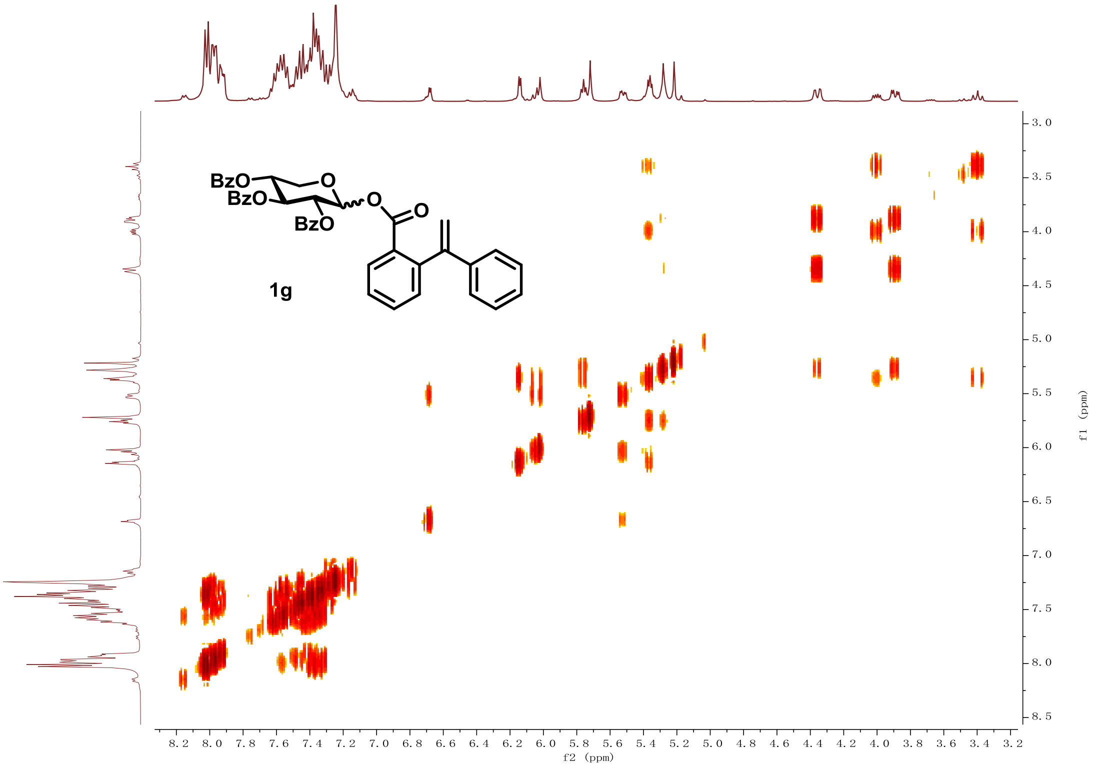

Supplementary Figure 40. COSY NMR Spectrum of Compound **1g**

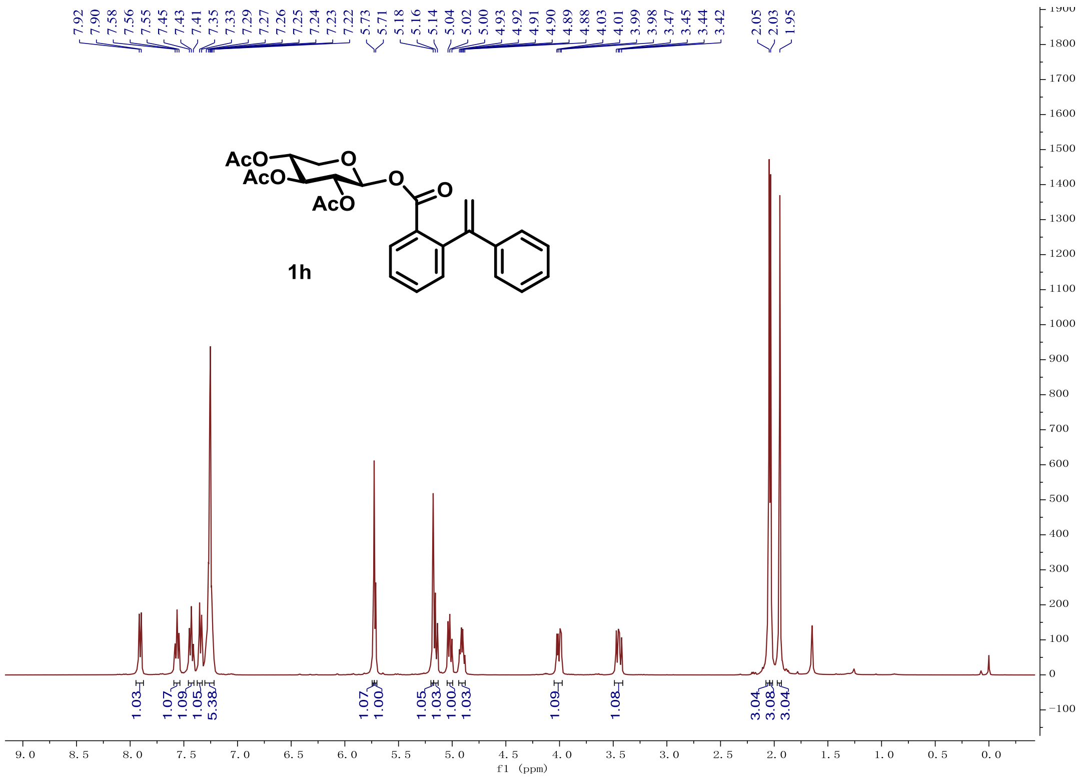

Supplementary Figure 41. ^{1}H NMR Spectrum of Compound **1h**

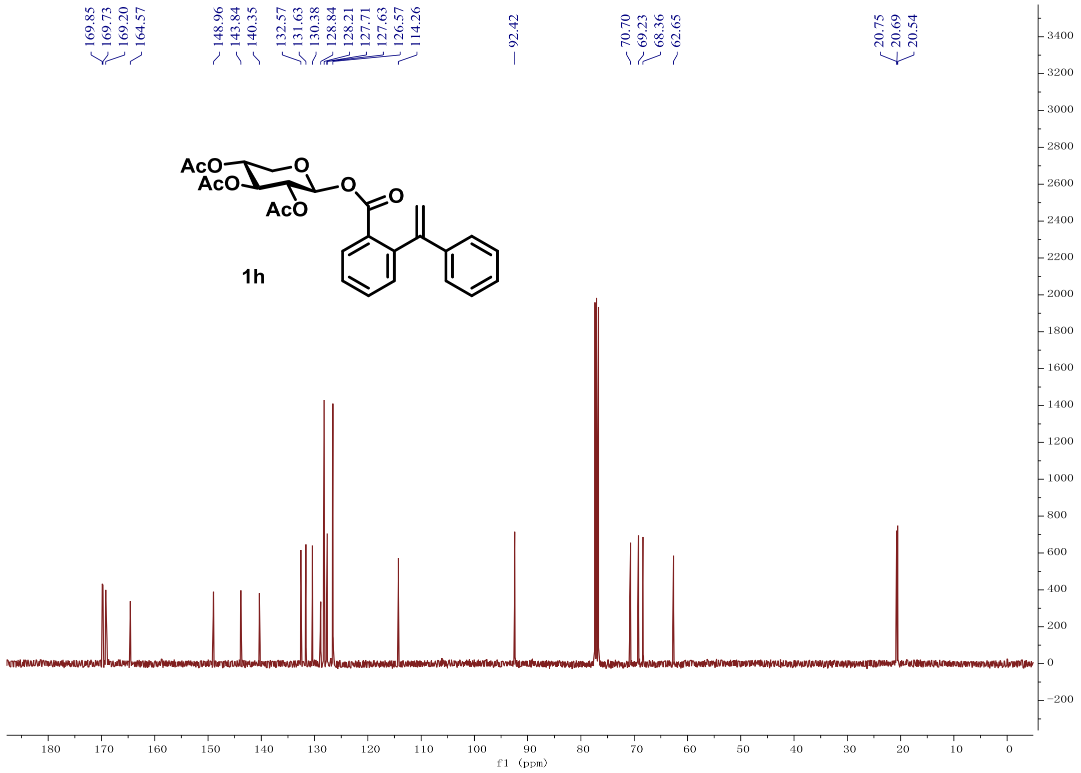

Supplementary Figure 42. ^{13}C NMR Spectrum of Compound **1h**

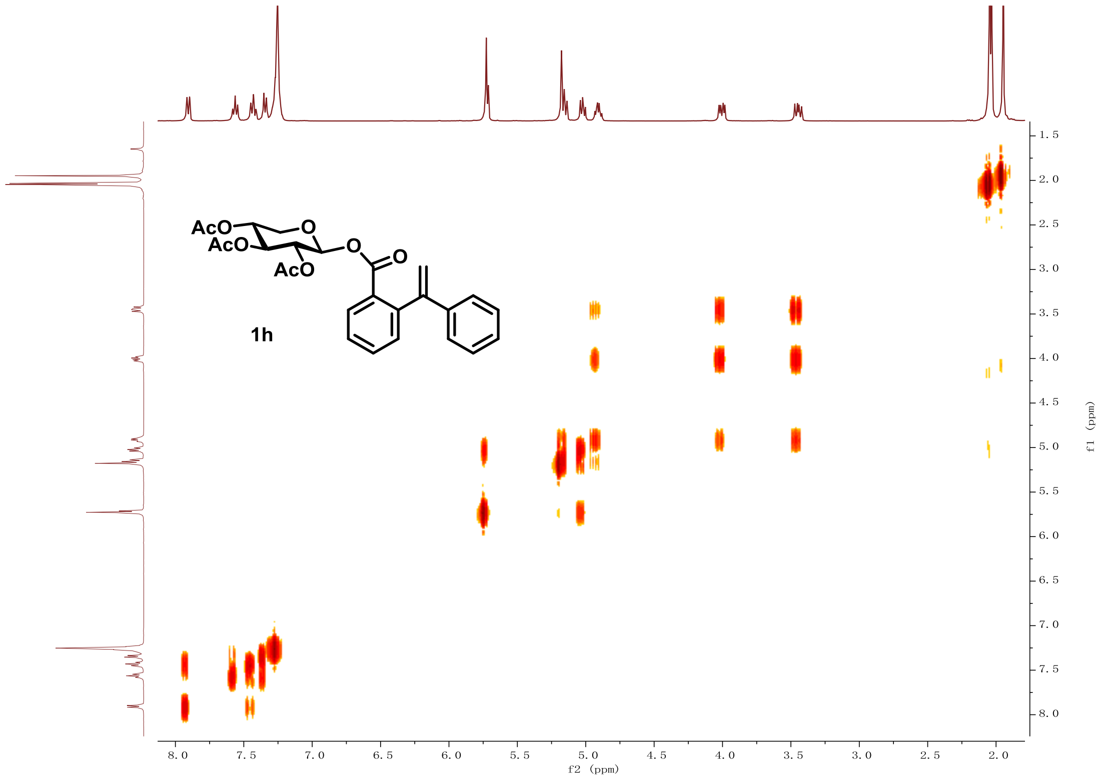

Supplementary Figure 43. COSY NMR Spectrum of Compound **1h**

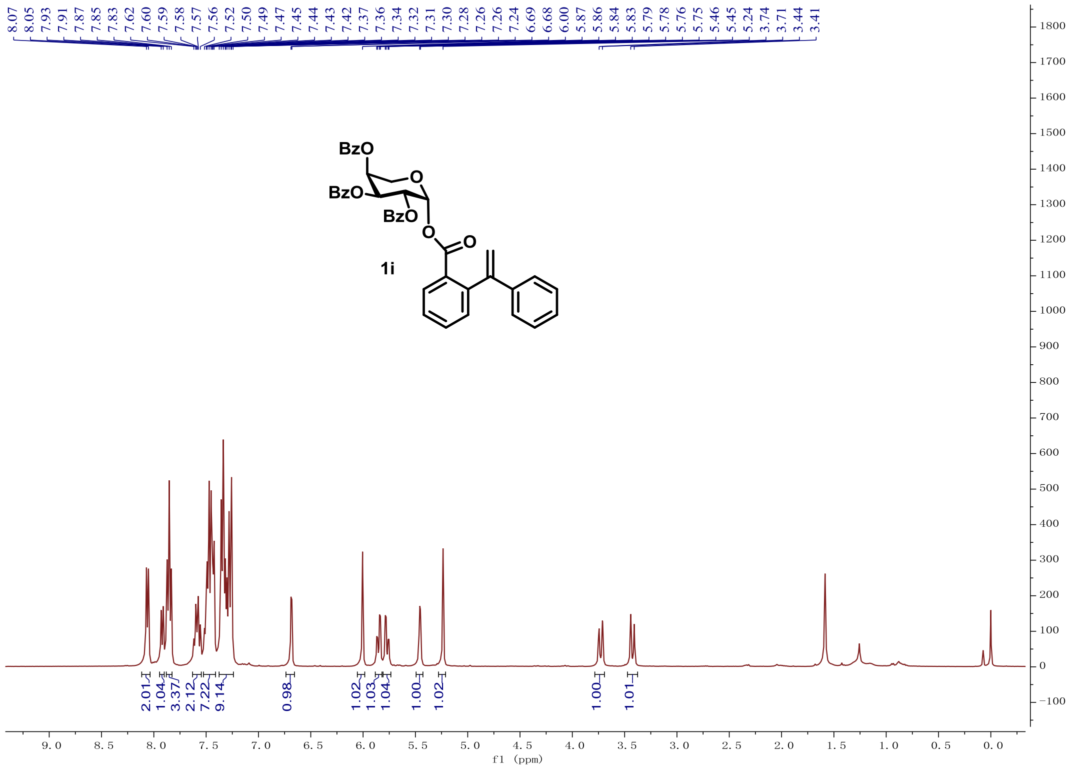

Supplementary Figure 44. ^{1}H NMR Spectrum of Compound **1i**

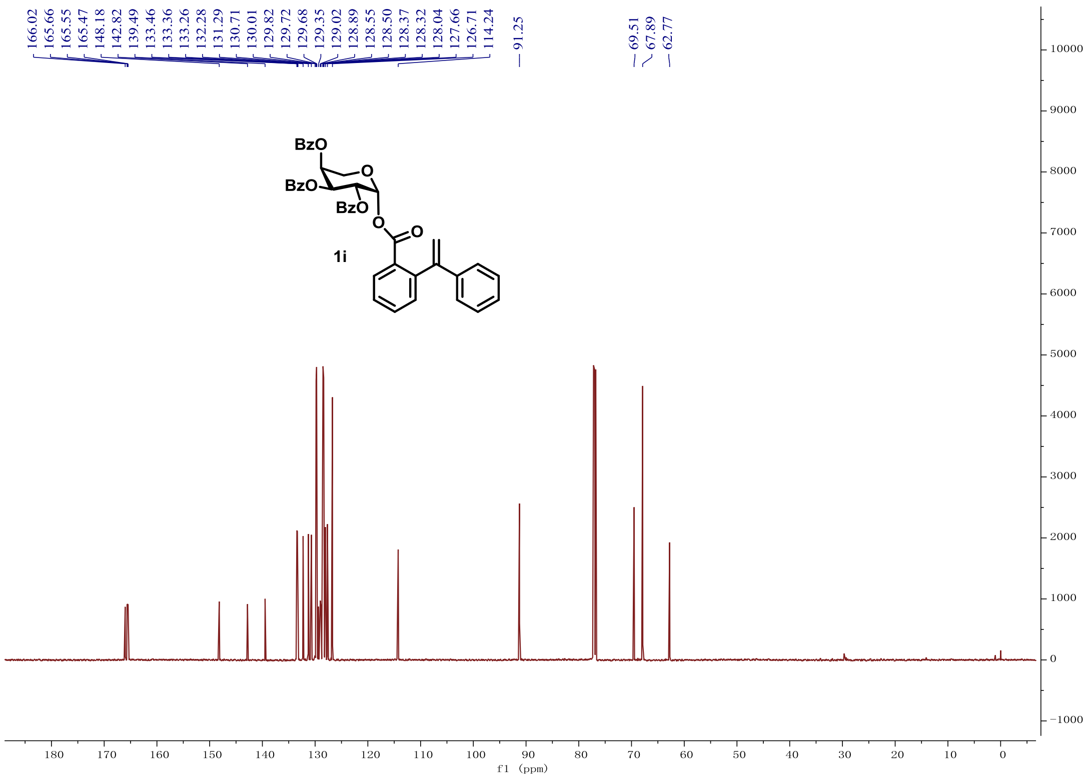

Supplementary Figure 45. ^{13}C NMR Spectrum of Compound **1i**

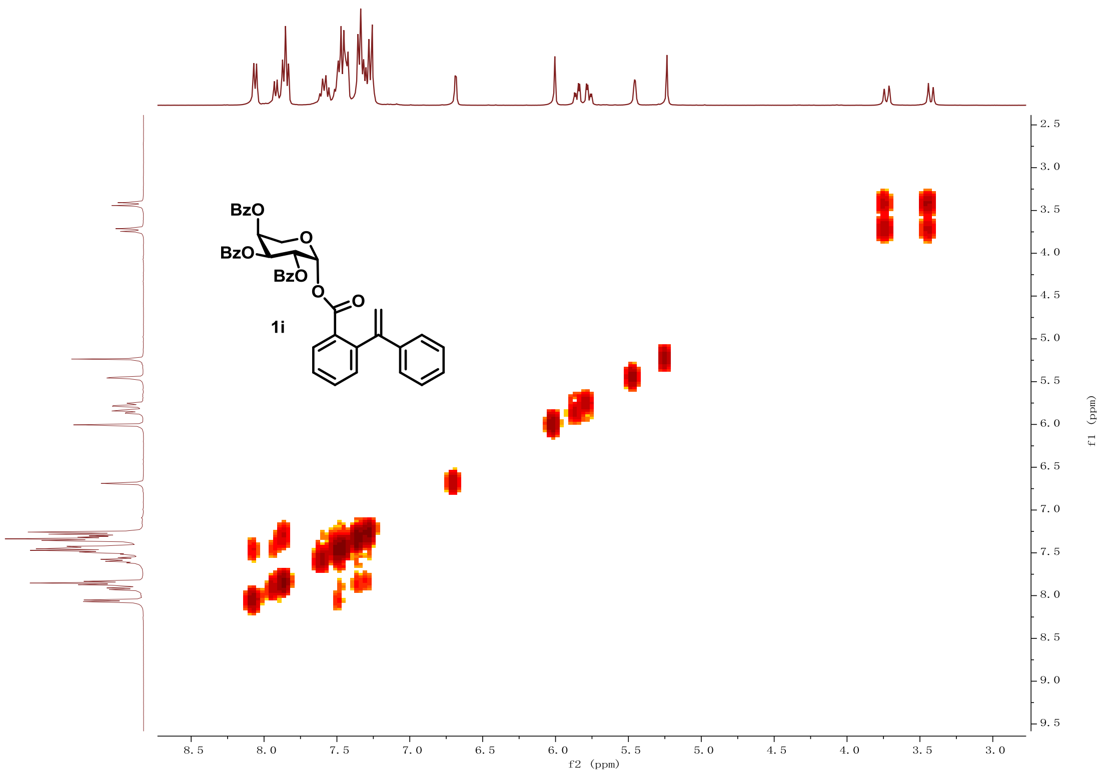

Supplementary Figure 46. COSY NMR Spectrum of Compound **1i**

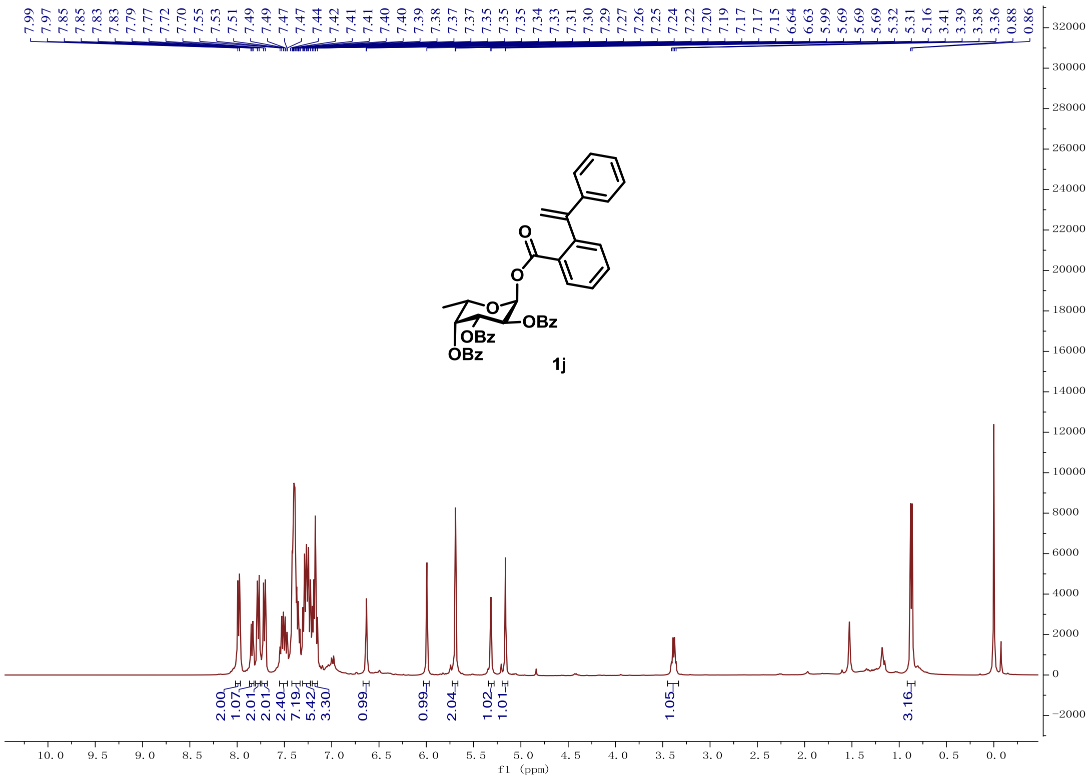

Supplementary Figure 47. ^{1}H NMR Spectrum of Compound **1j**

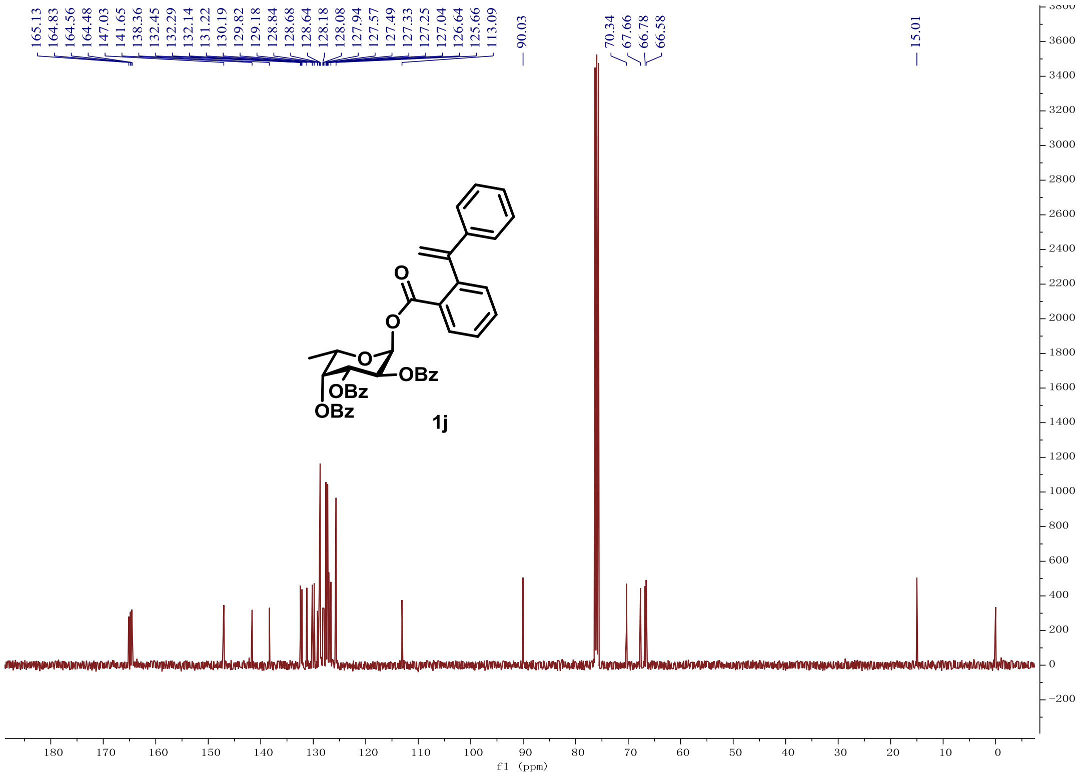

Supplementary Figure 48. ^{13}C NMR Spectrum of Compound **1j**

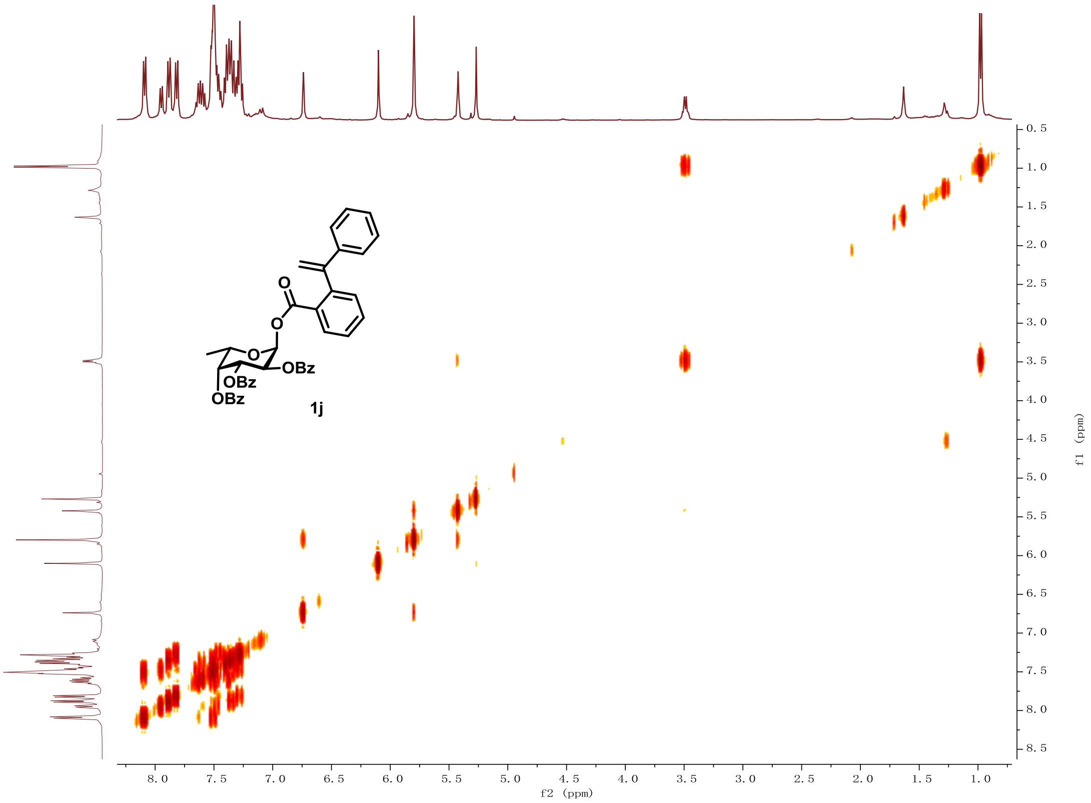

Supplementary Figure 49. COSY NMR Spectrum of Compound **1j**

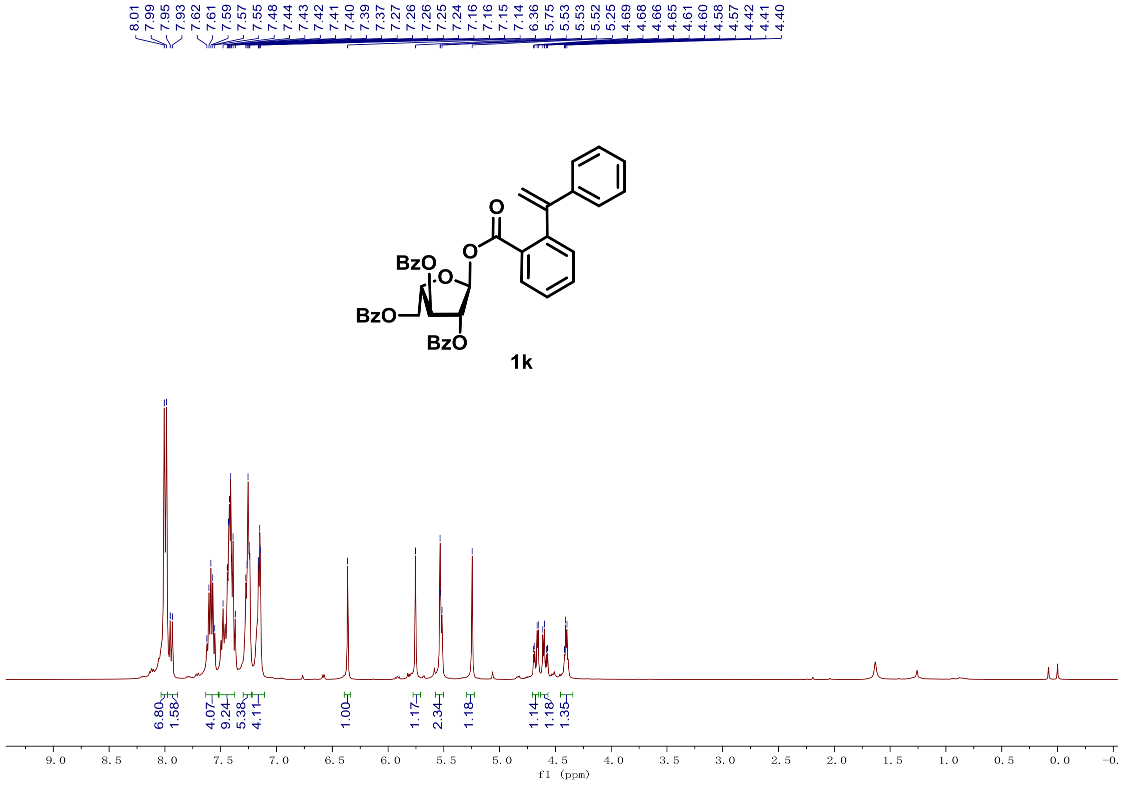

Supplementary Figure 50. ^{1}H NMR Spectrum of Compound **1k**

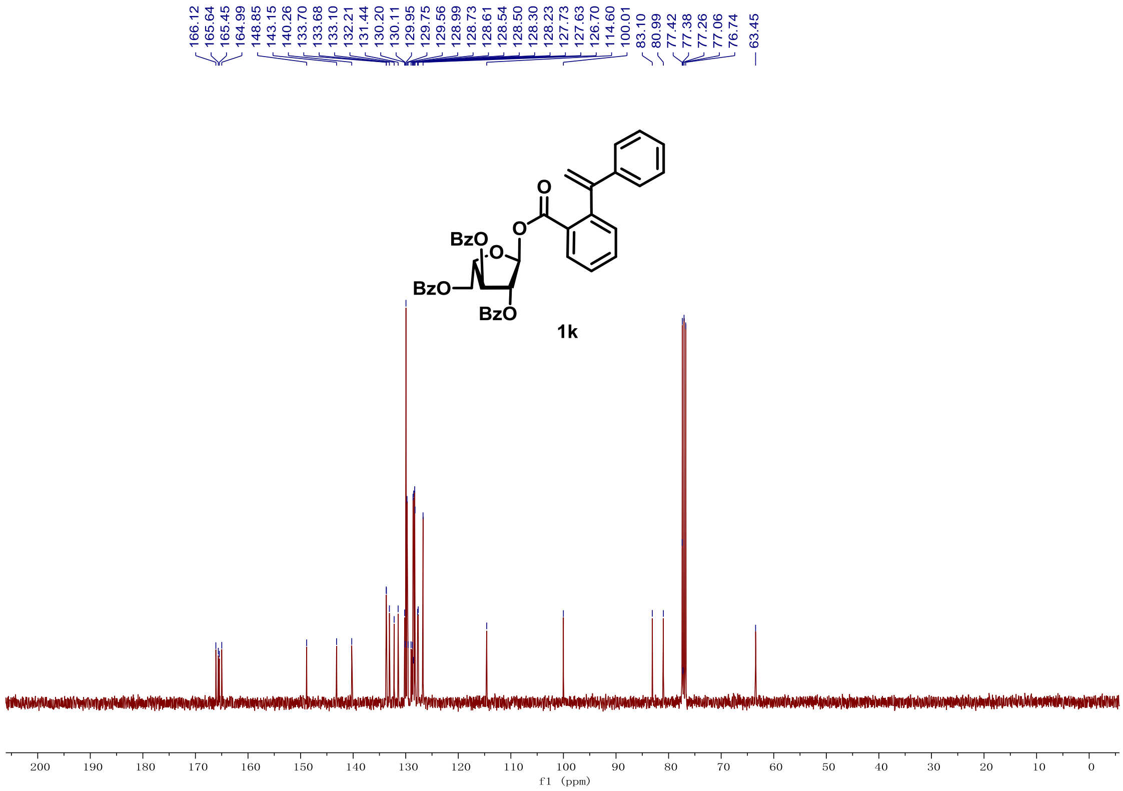

Supplementary Figure 51. ^{13}C NMR Spectrum of Compound **1k**

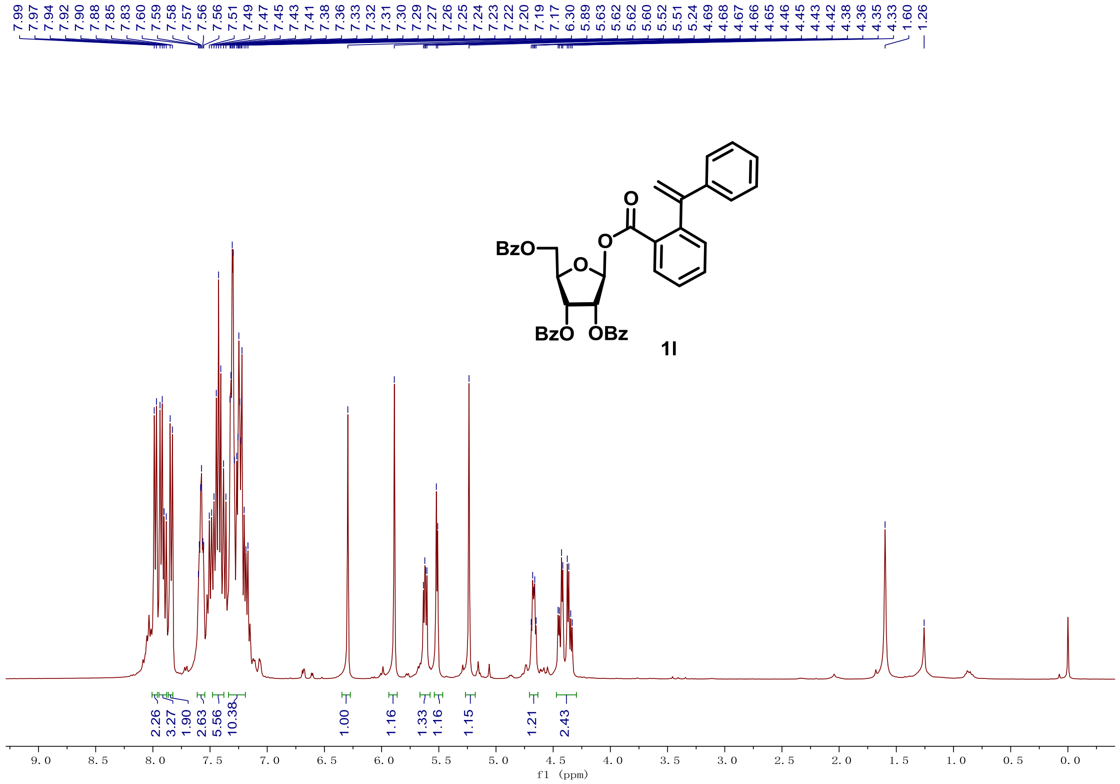

Supplementary Figure 52. ^{1}H NMR Spectrum of Compound **1l**

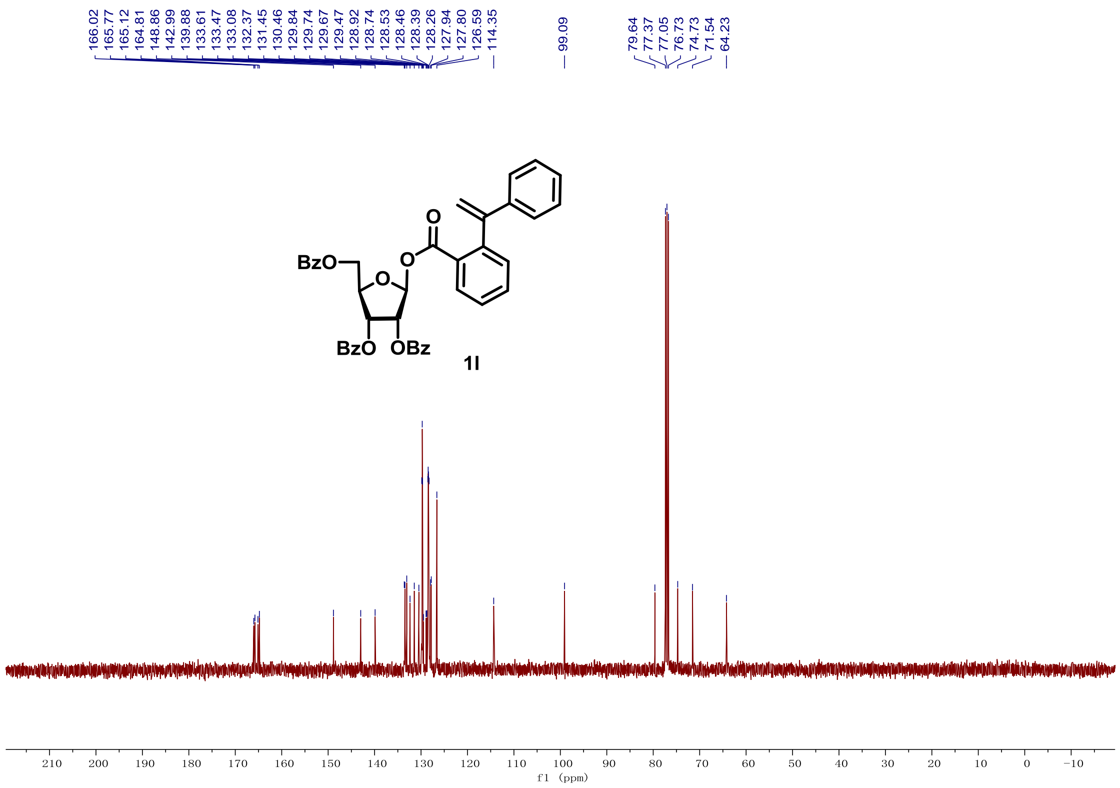

Supplementary Figure 53. ^{13}C NMR Spectrum of Compound **1l**

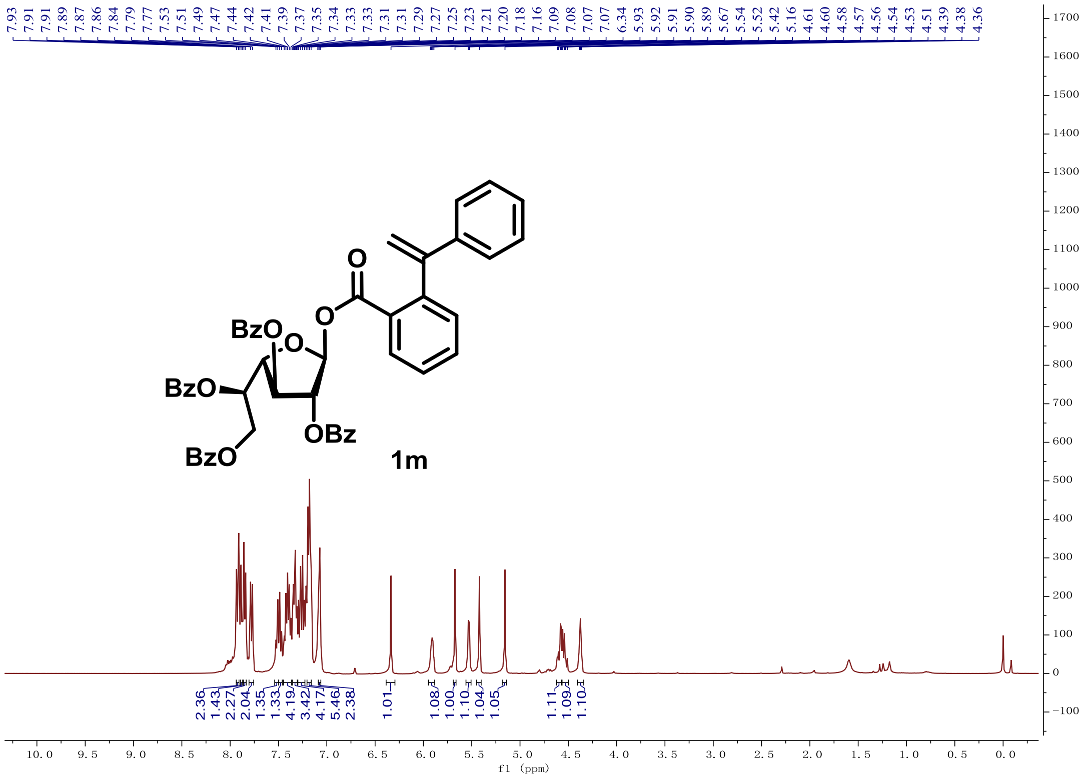

Supplementary Figure 54. ^{1}H NMR Spectrum of Compound **1m**

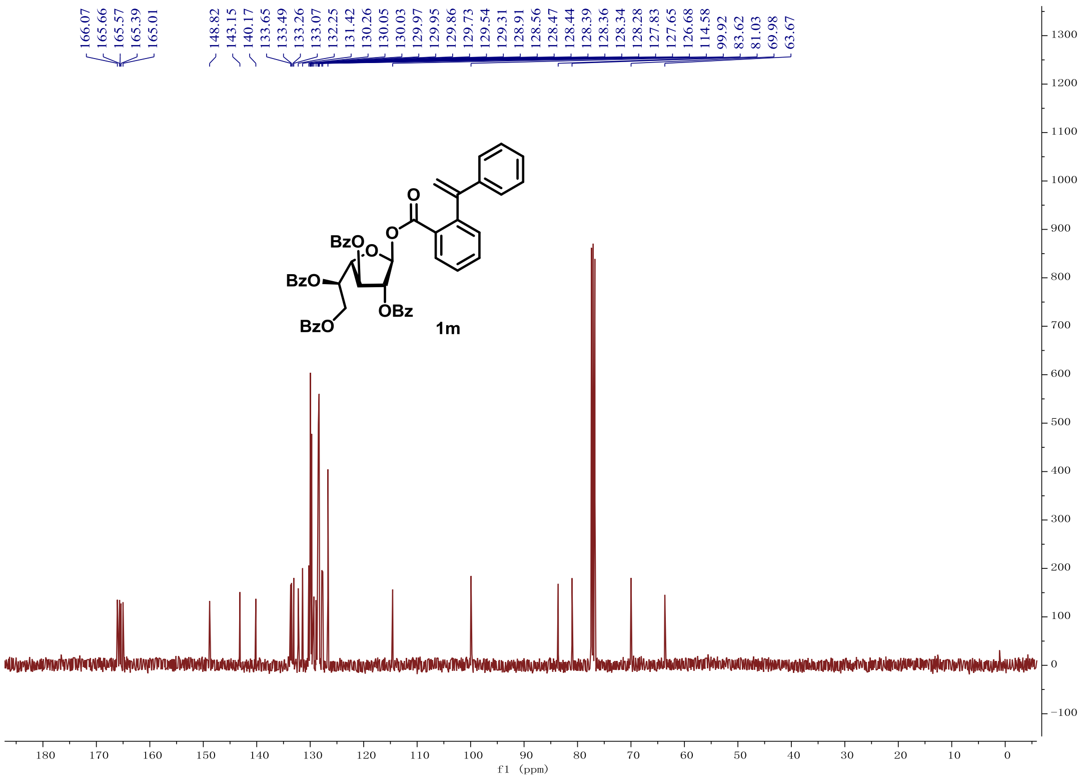

Supplementary Figure 55. ^{13}C NMR Spectrum of Compound **1m**

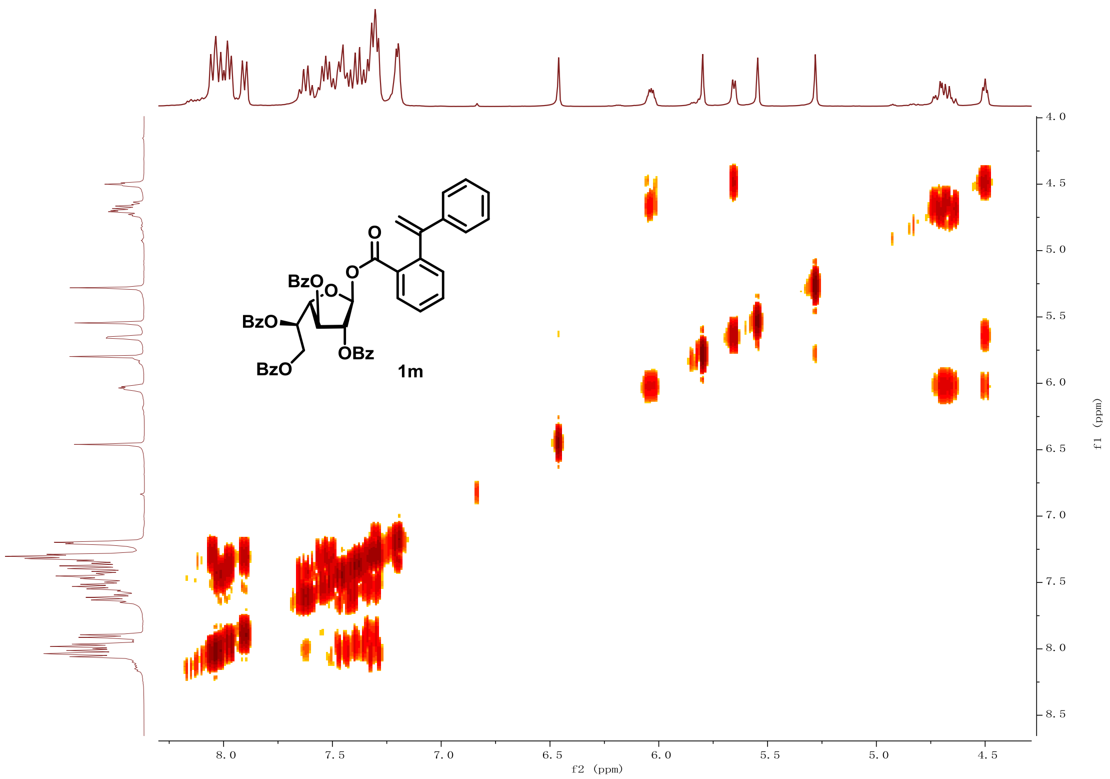

Supplementary Figure 56. COSY NMR Spectrum of Compound **1m**

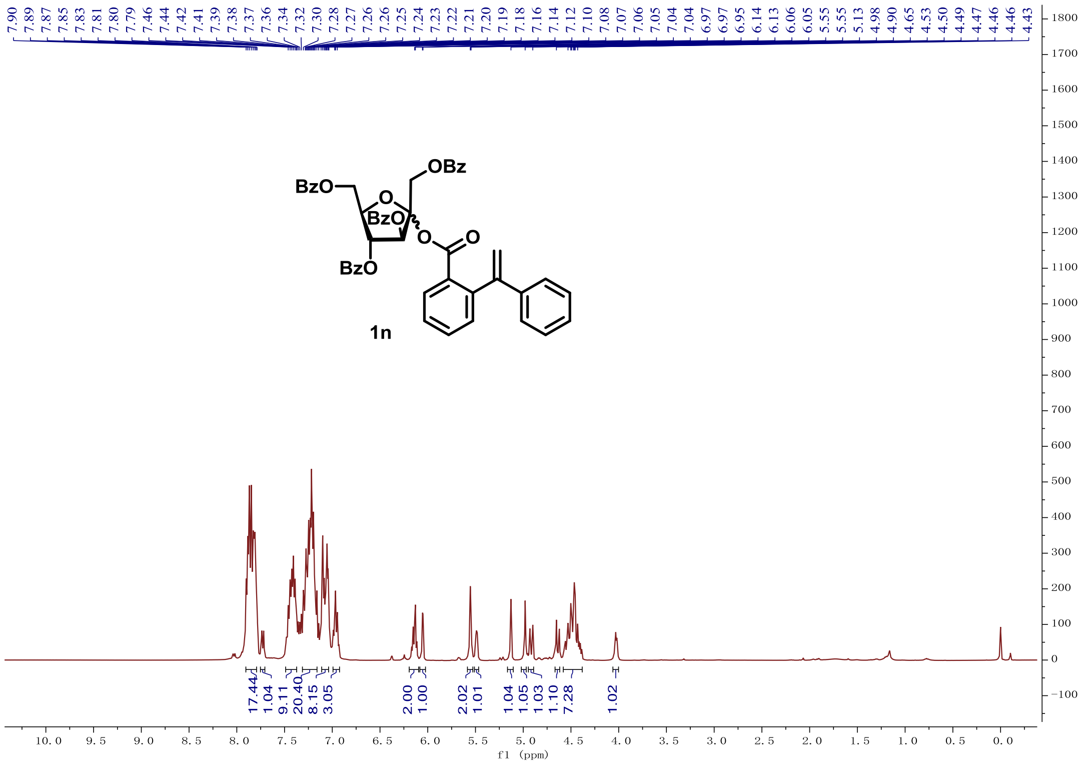

Supplementary Figure 57. ^{1}H NMR Spectrum of Compound **1n**

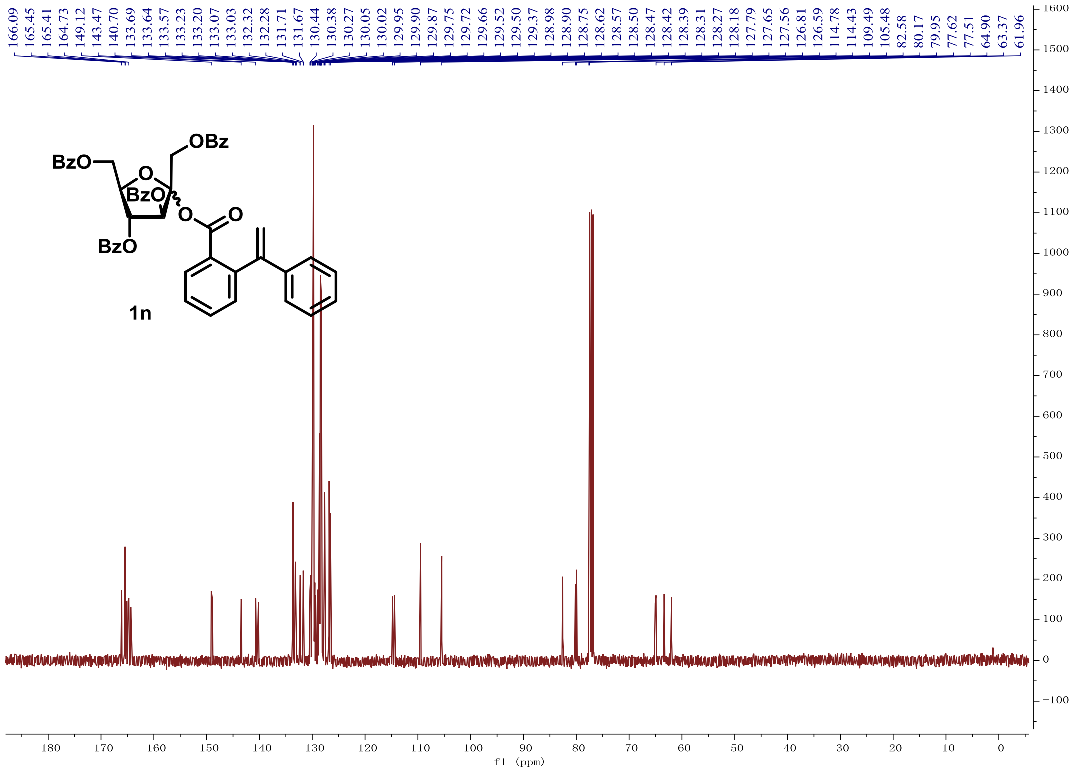

Supplementary Figure 58. ^{13}C NMR Spectrum of Compound **1n**

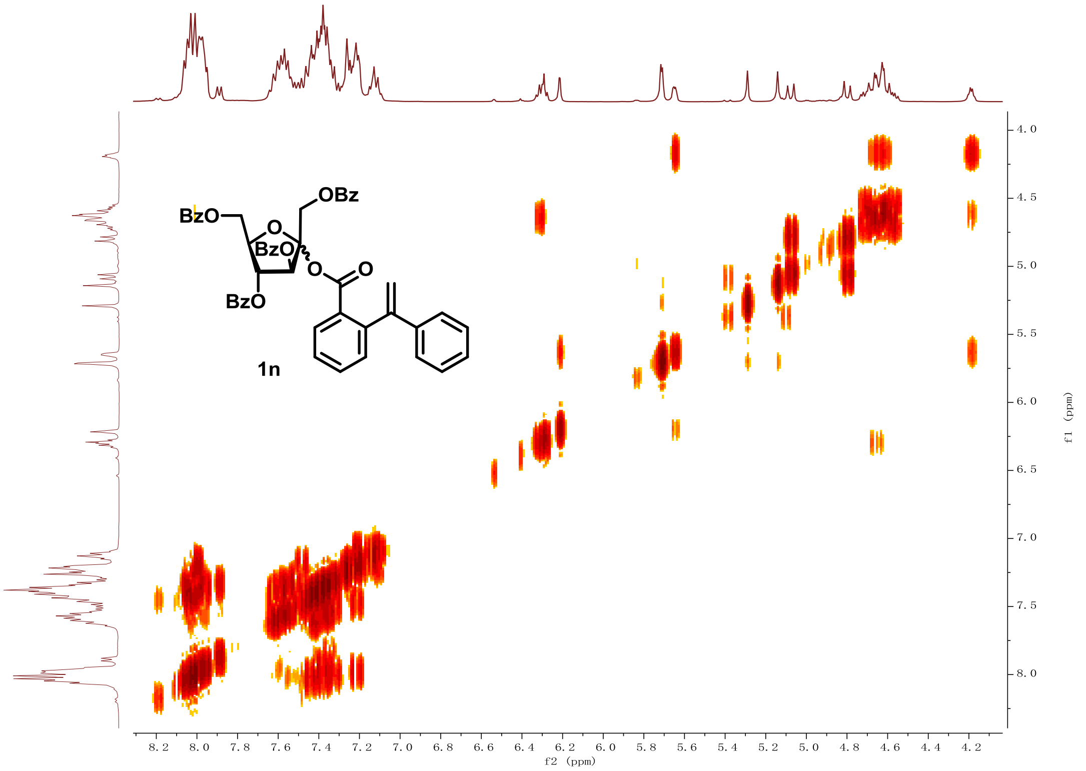

Supplementary Figure 59. COSY NMR Spectrum of Compound **1n**

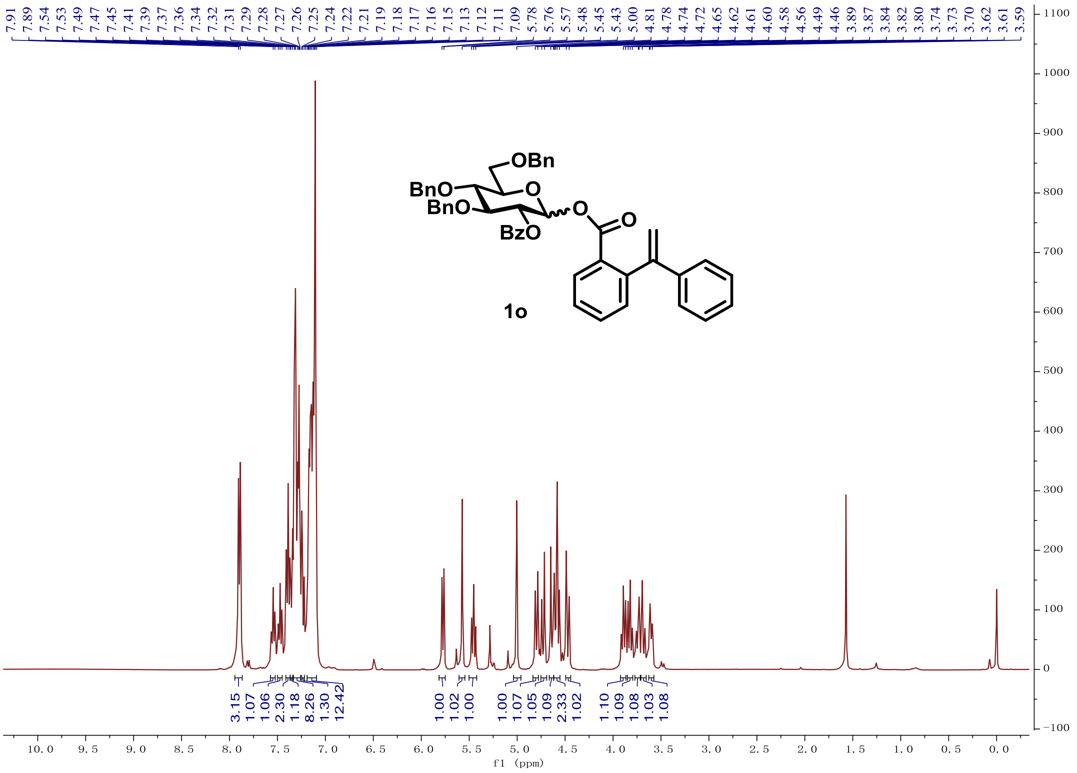

Supplementary Figure 60. ^{1}H NMR Spectrum of Compound **1o**

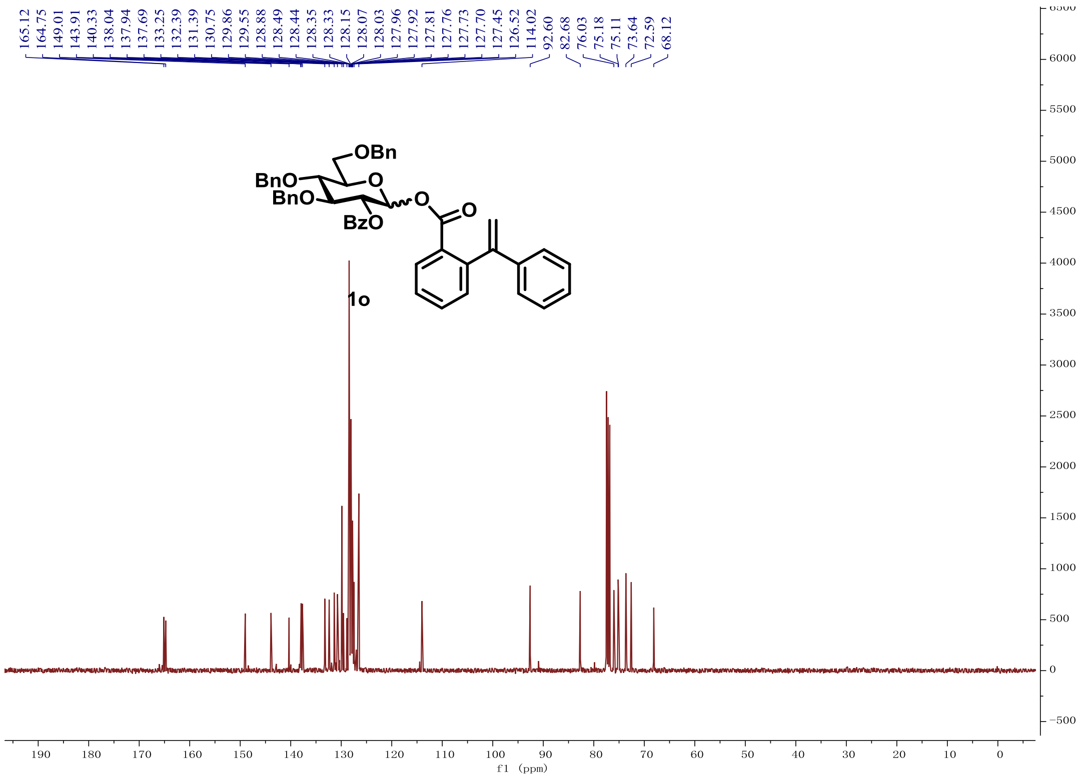

Supplementary Figure 61. ^{13}C NMR Spectrum of Compound **1o**

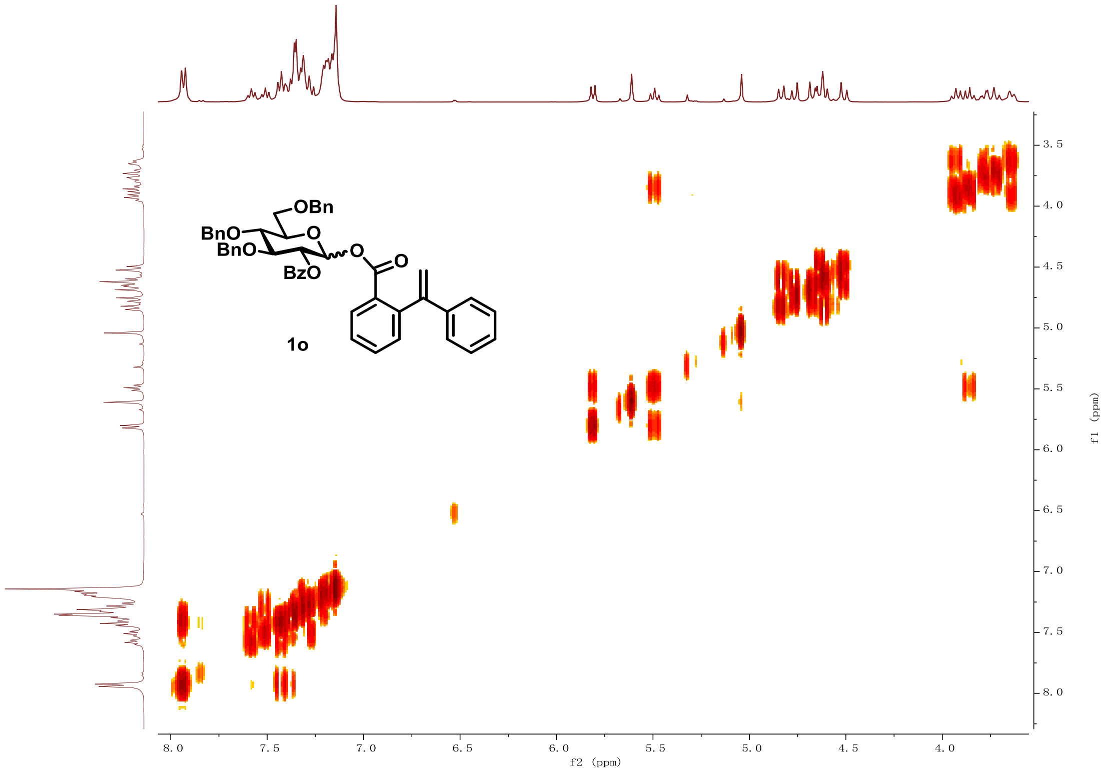

Supplementary Figure 62. COSY NMR Spectrum of Compound **1o**

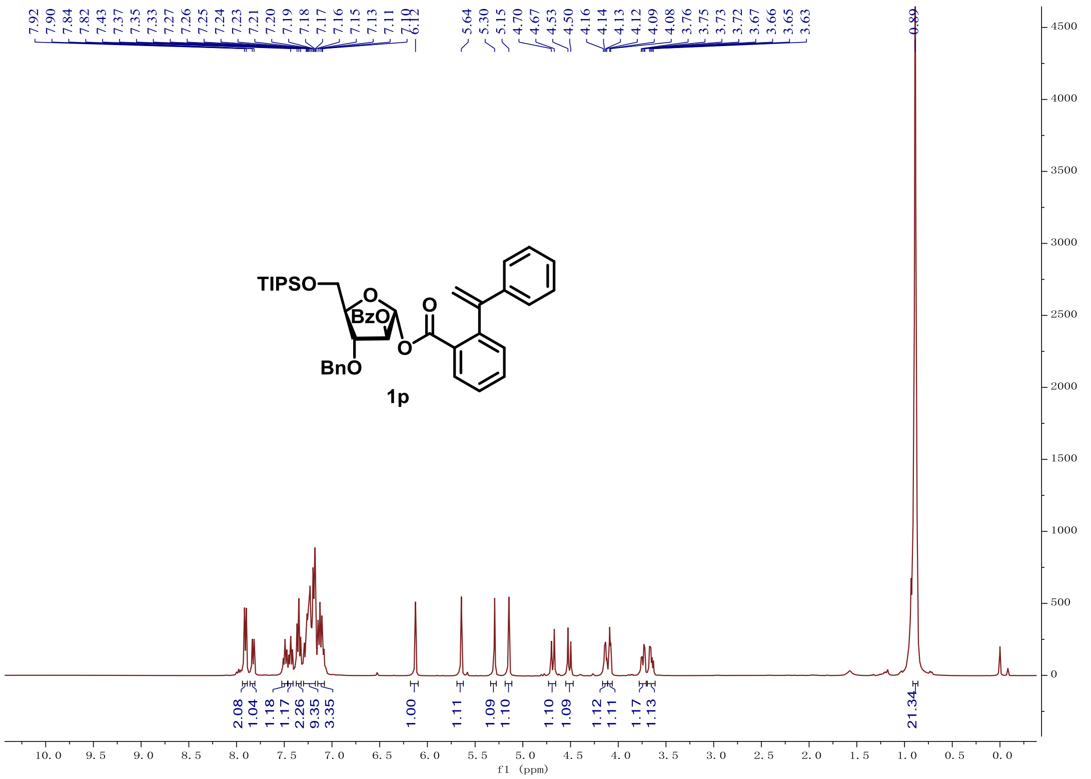

Supplementary Figure 63. ^{1}H NMR Spectrum of Compound **1p**

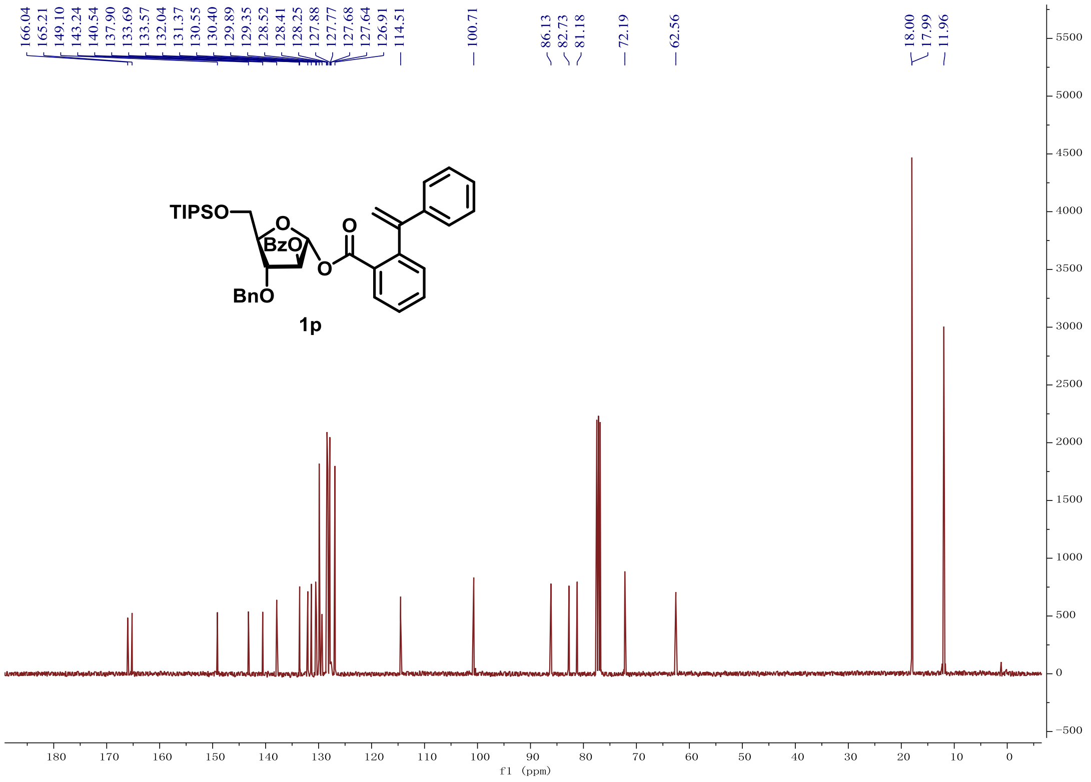

Supplementary Figure 64. ^{13}C NMR Spectrum of Compound **1p**

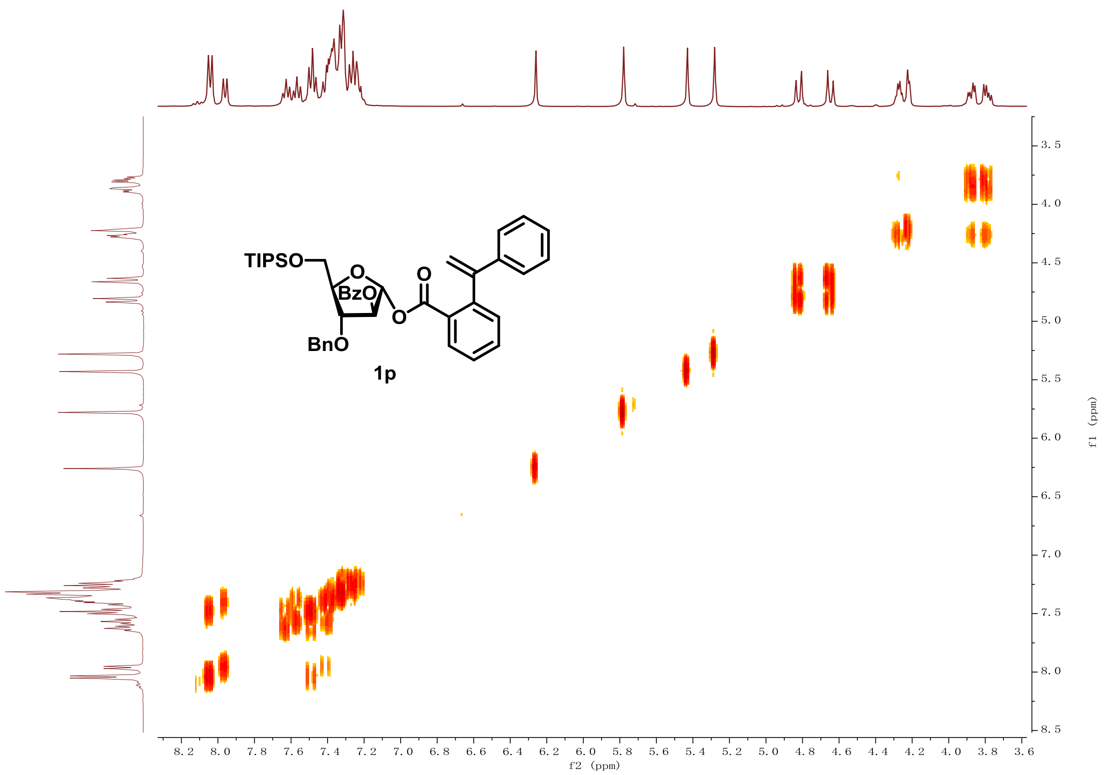

Supplementary Figure 65. COSY NMR Spectrum of Compound **1p**

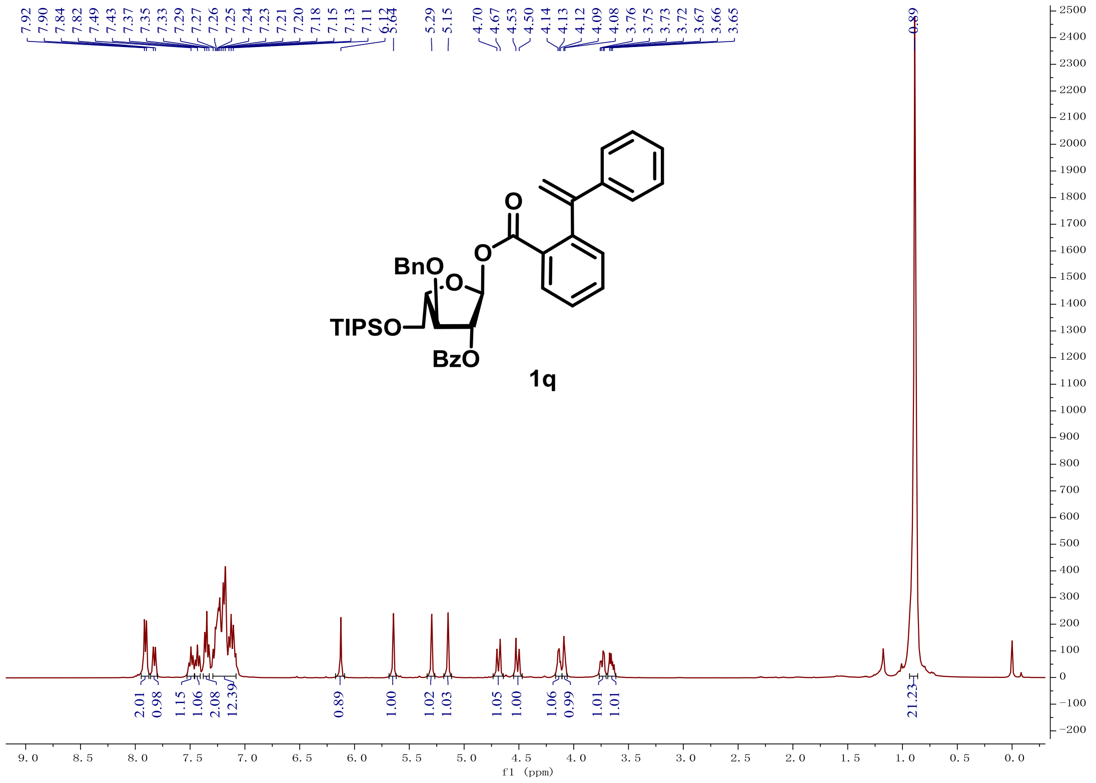

Supplementary Figure 66. ^{1}H NMR Spectrum of Compound **1q**

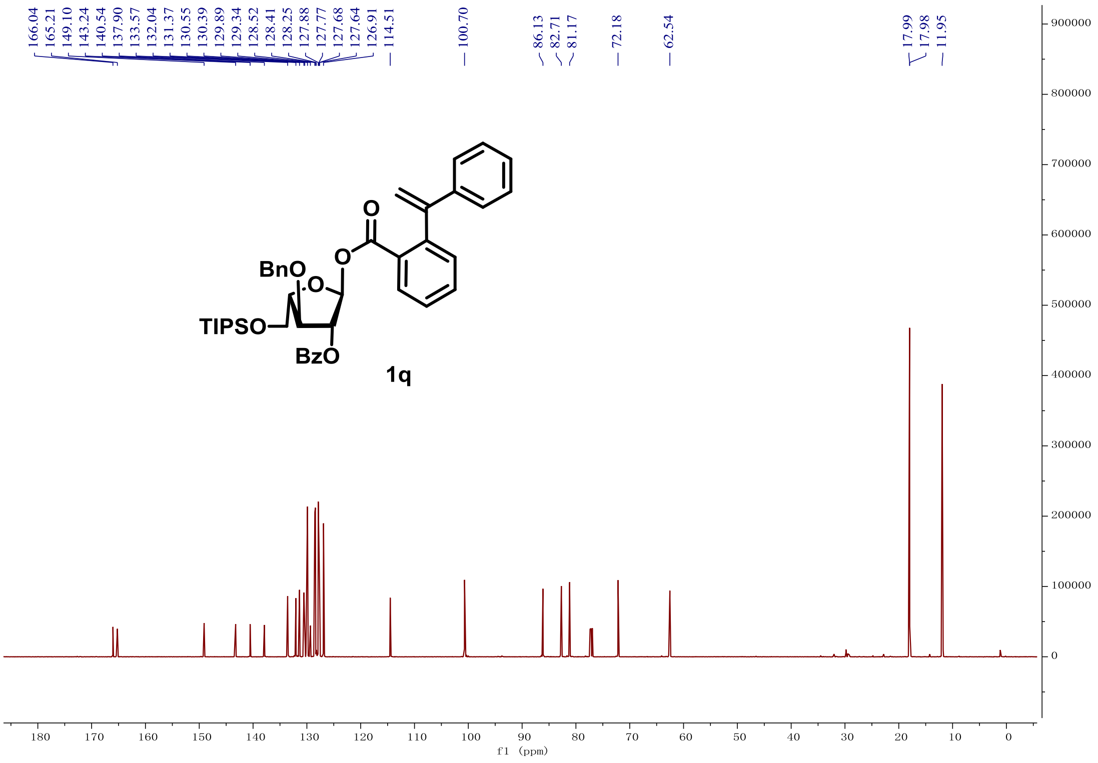

Supplementary Figure 67. ^{13}C NMR Spectrum of Compound **1q**

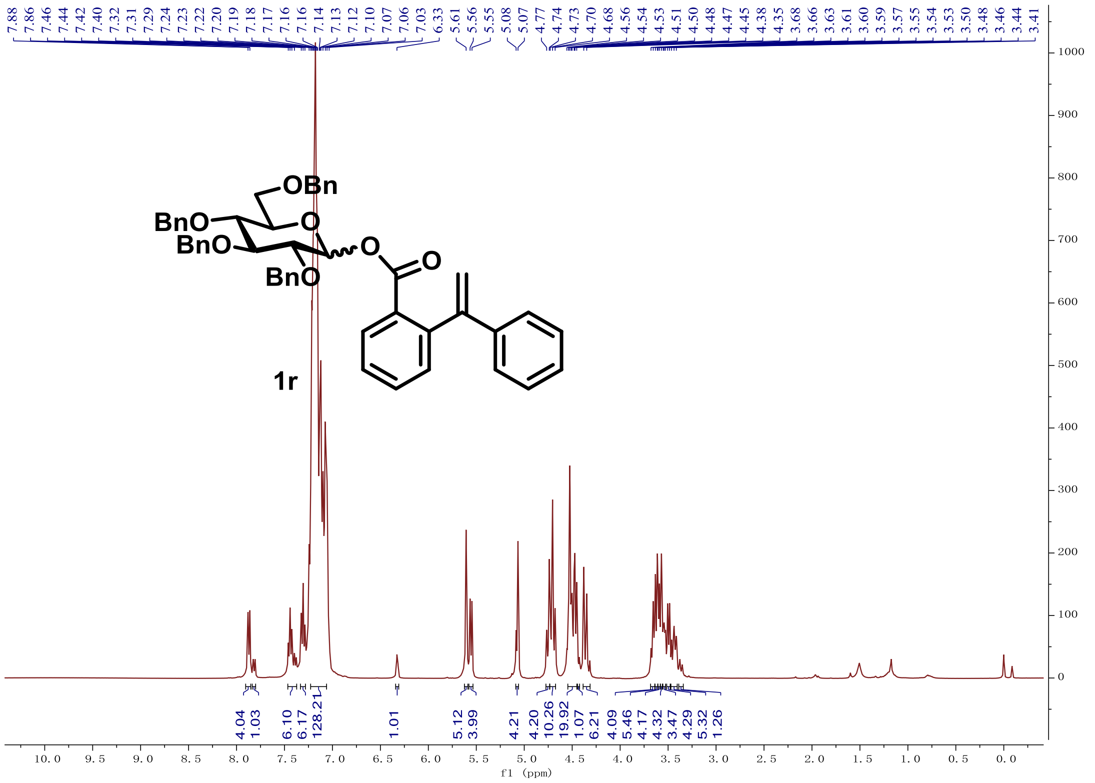

Supplementary Figure 68. ^{1}H NMR Spectrum of Compound **1r**

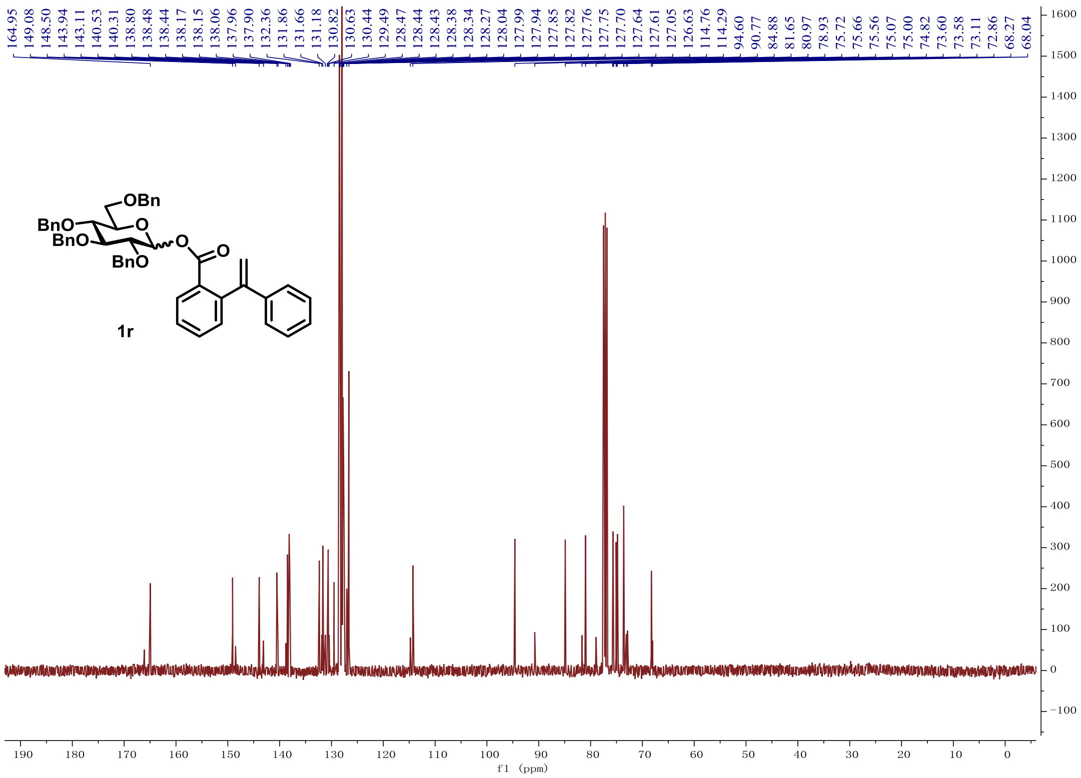

Supplementary Figure 69. ^{13}C NMR Spectrum of Compound **1r**

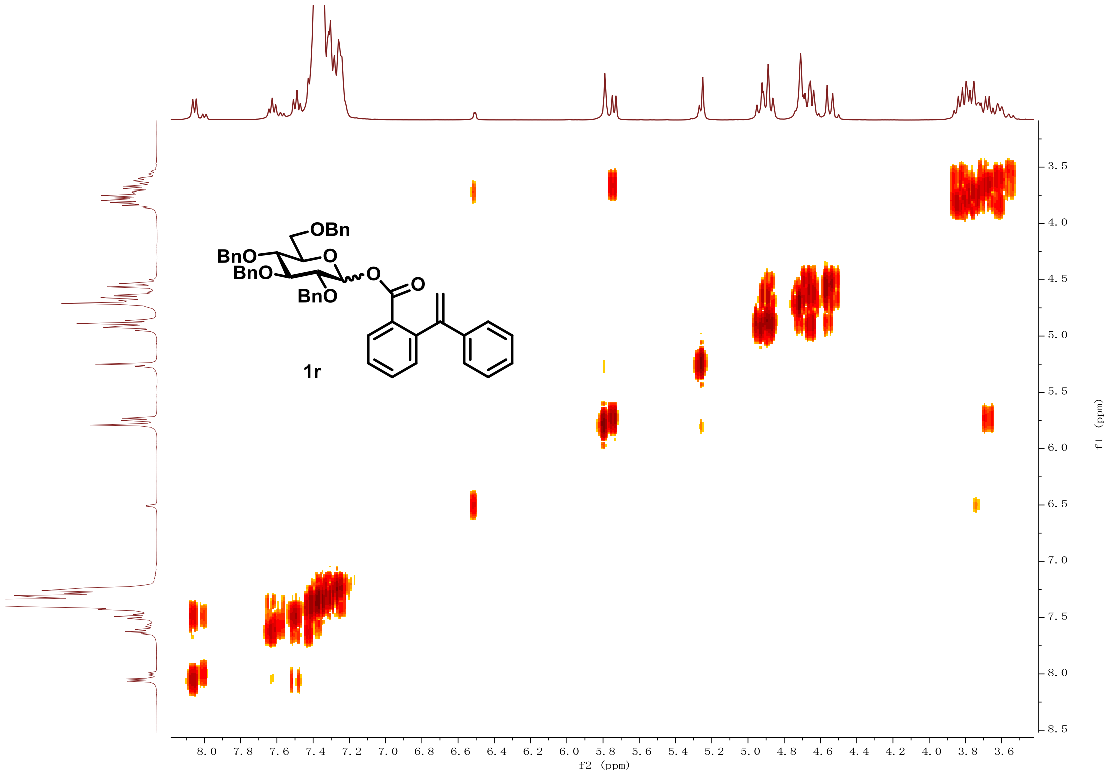

Supplementary Figure 70. COSY NMR Spectrum of Compound **1r**

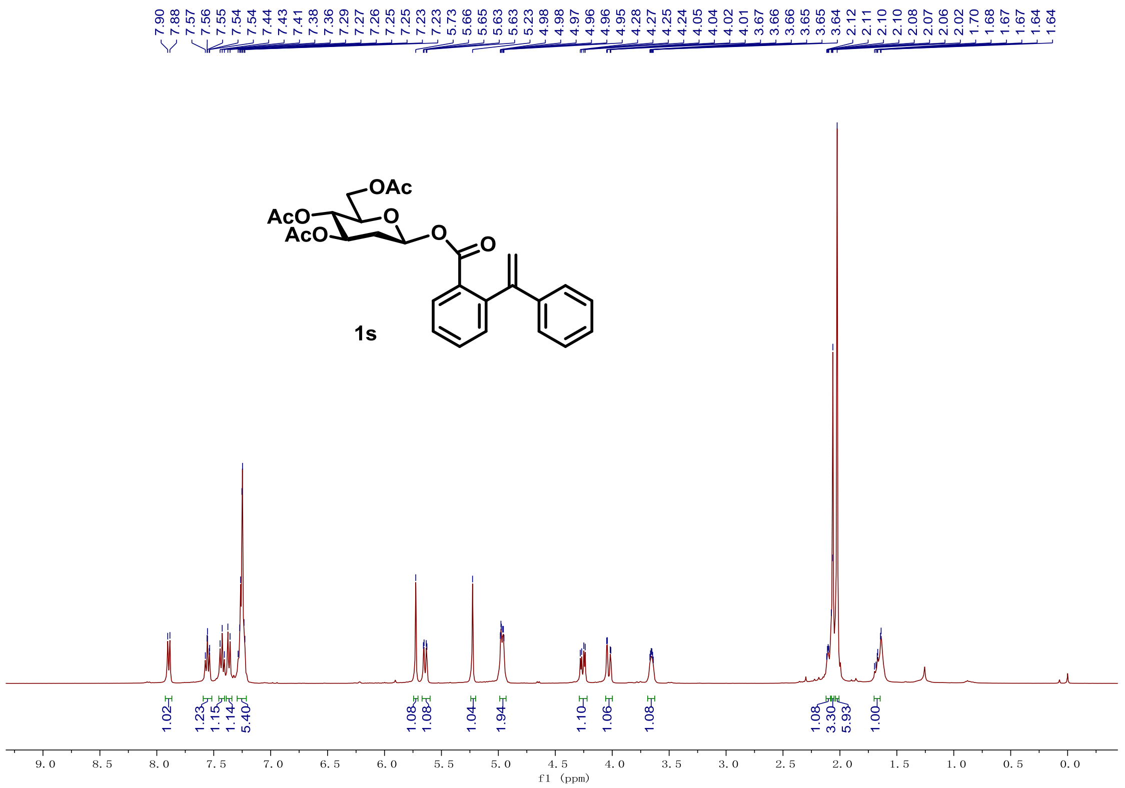

Supplementary Figure 71. ^{1}H NMR Spectrum of Compound **1s**

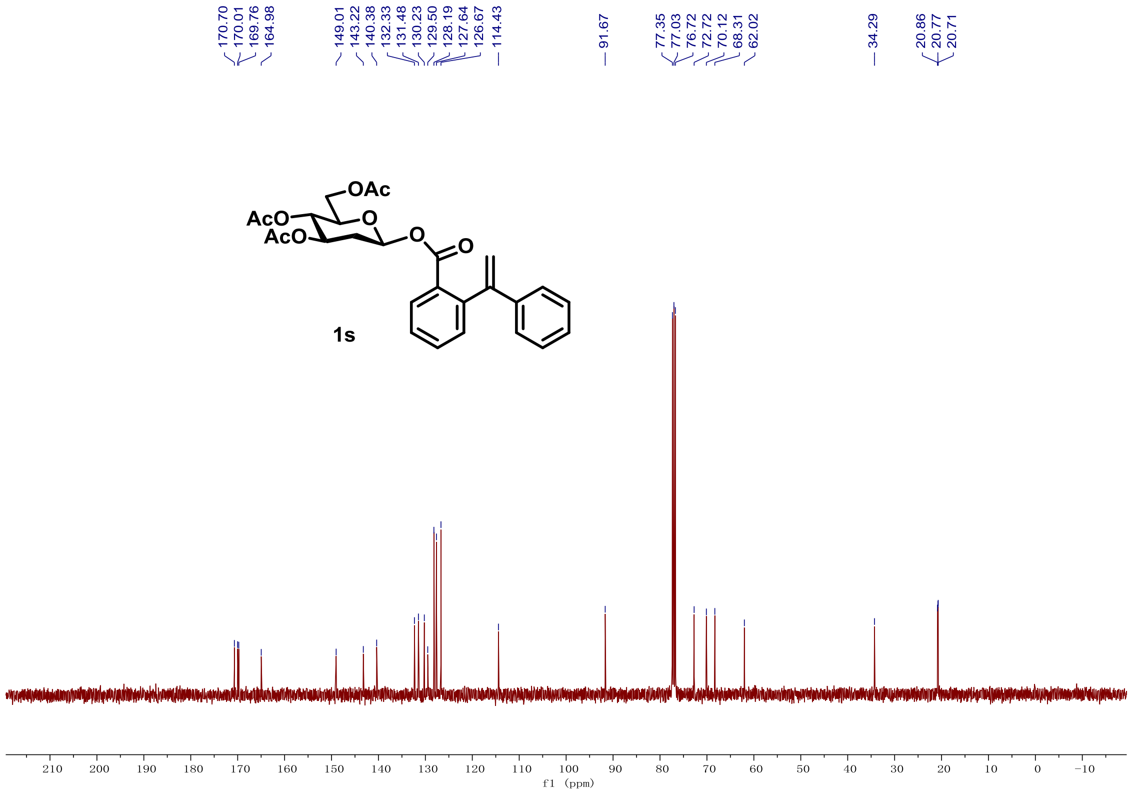

Supplementary Figure 72. ^{13}C NMR Spectrum of Compound **1s**

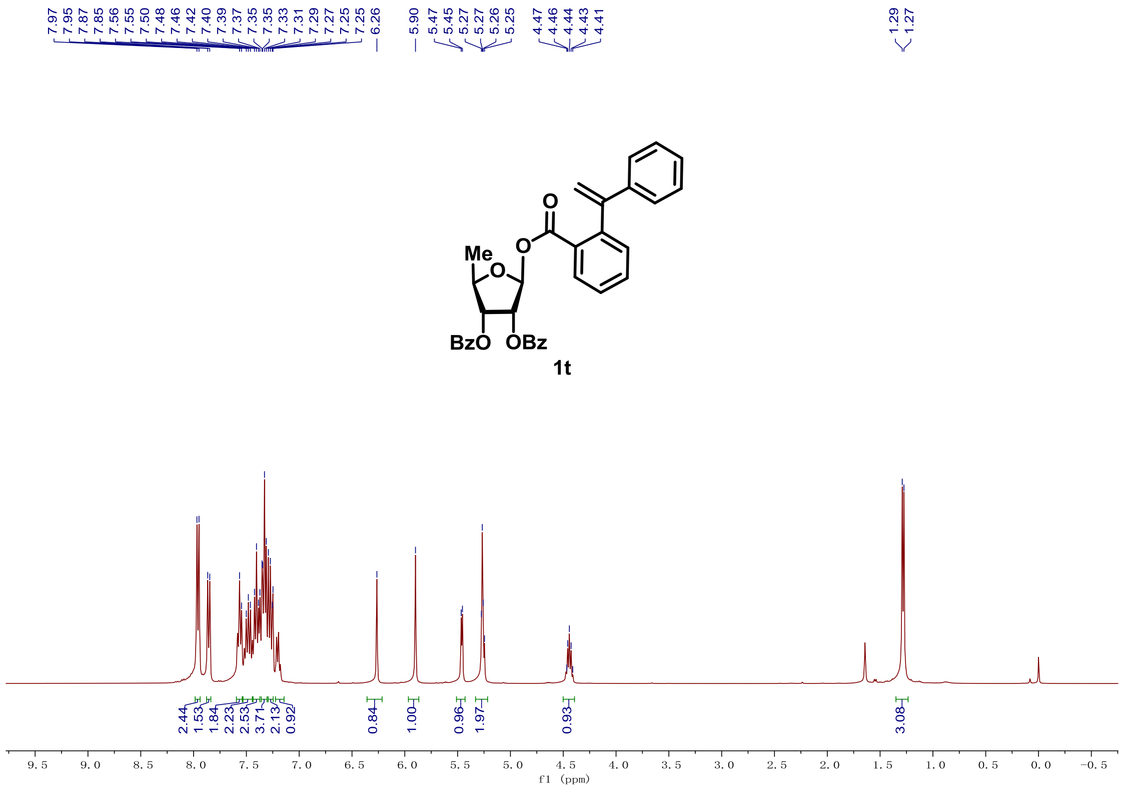

Supplementary Figure 73. ^{1}H NMR Spectrum of Compound **1t**

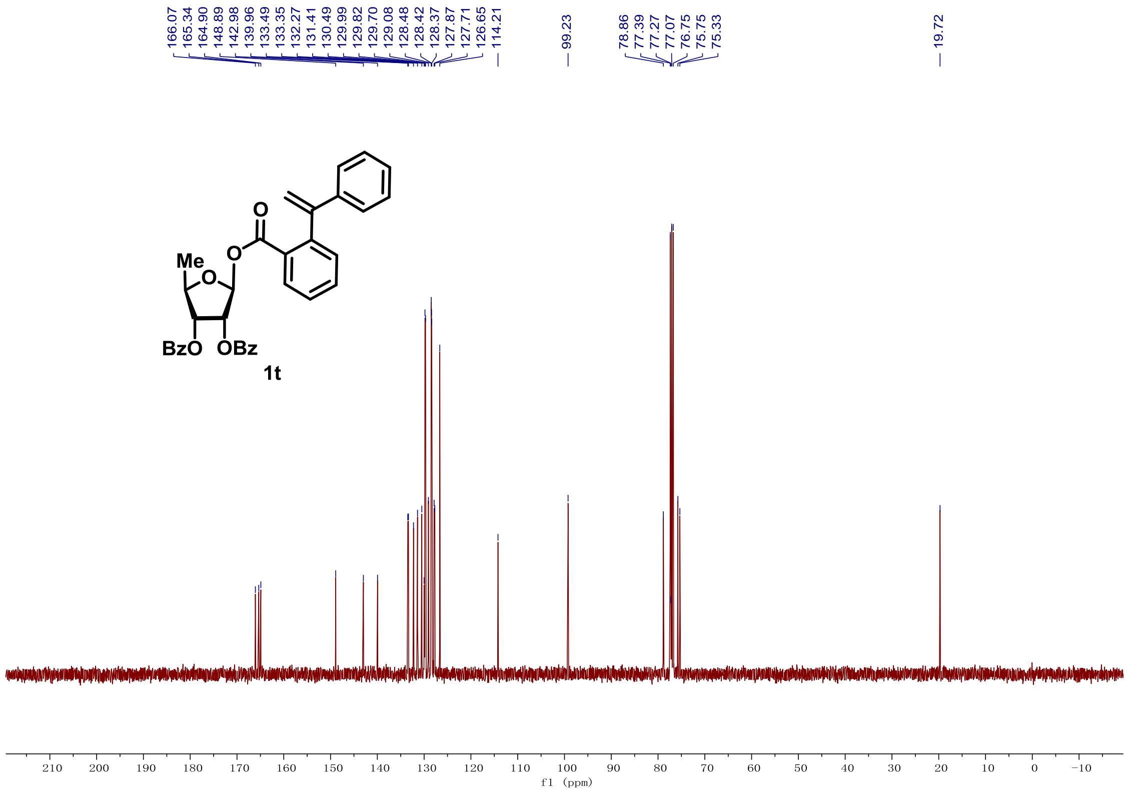

Supplementary Figure 74. ^{13}C NMR Spectrum of Compound **1t**

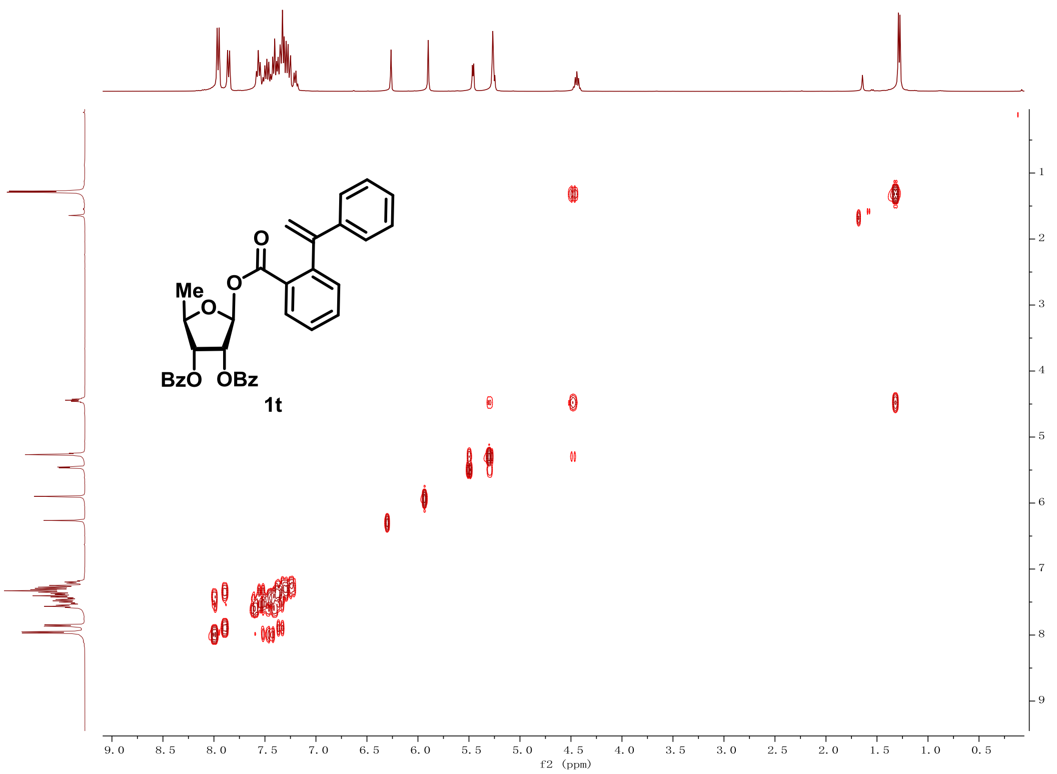

Supplementary Figure 75. COSY NMR Spectrum of Compound **1t**

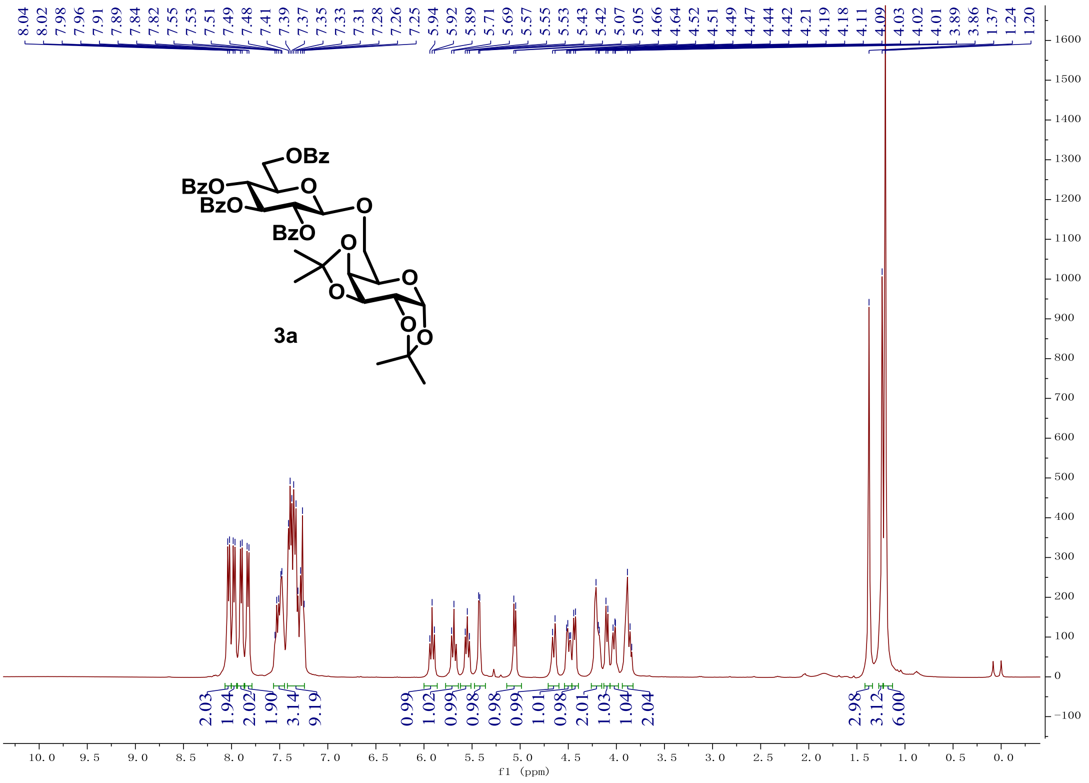

Supplementary Figure 76. ^{1}H NMR Spectrum of Compound **3a**

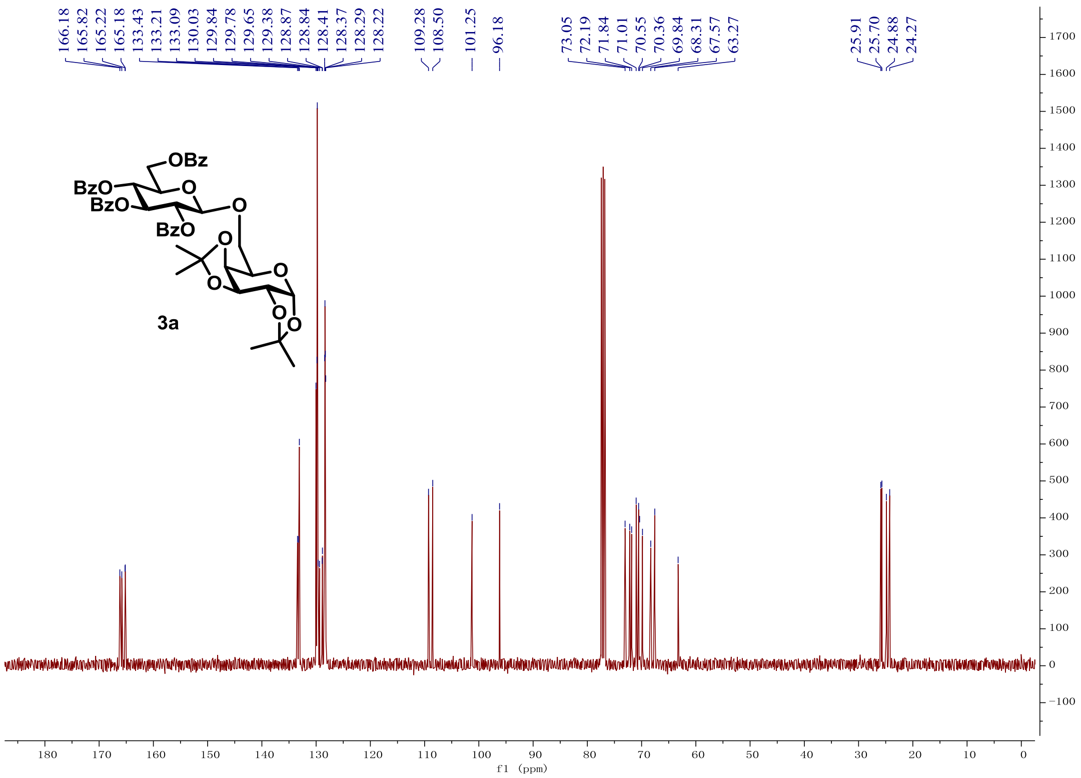

Supplementary Figure 77. ^{13}C NMR Spectrum of Compound **3a**

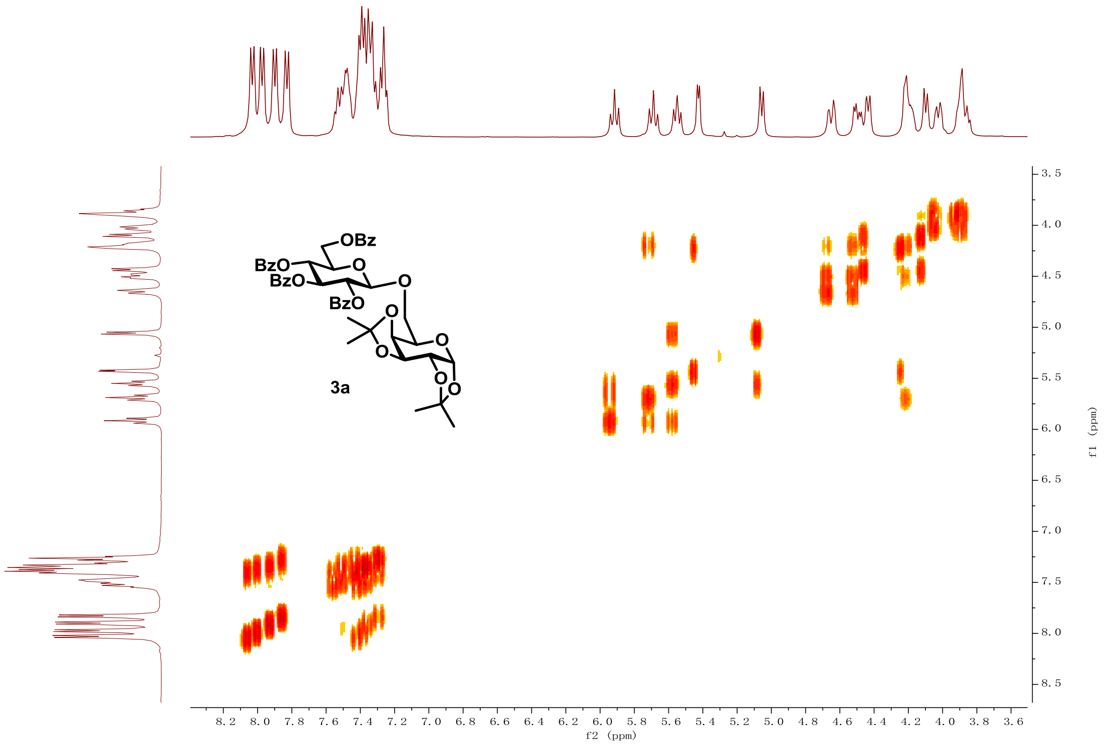

Supplementary Figure 78. COSY NMR Spectrum of Compound **3a**

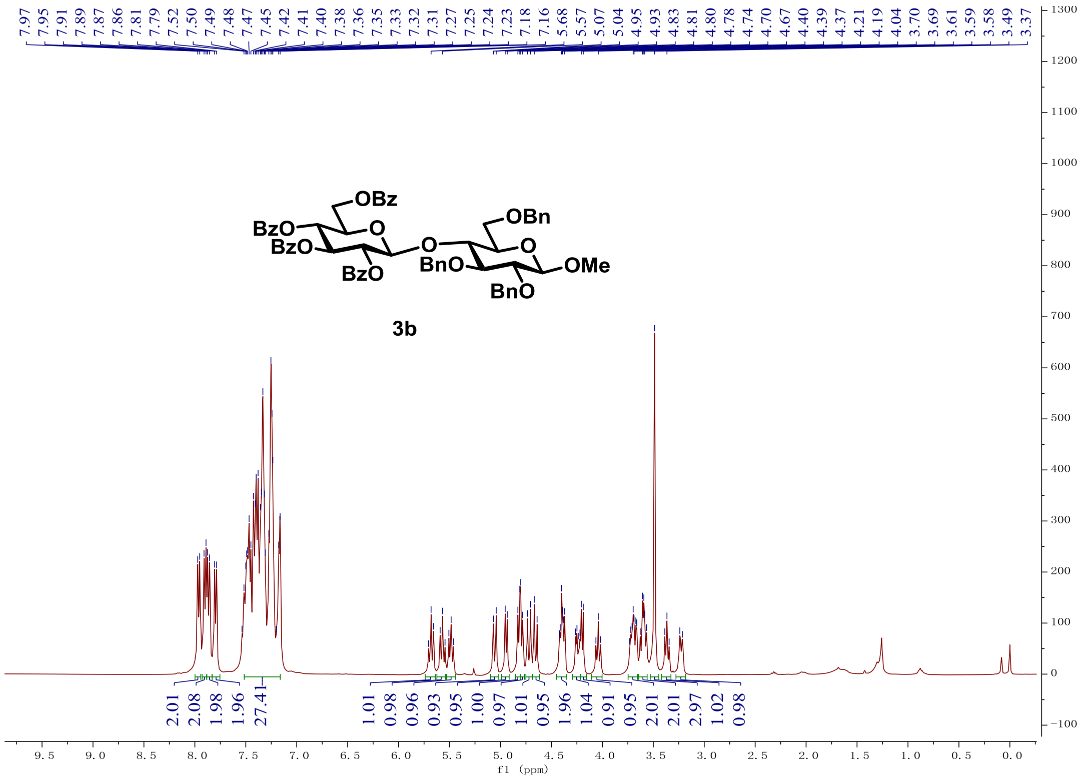

Supplementary Figure 79. ^{1}H NMR Spectrum of Compound **3b**

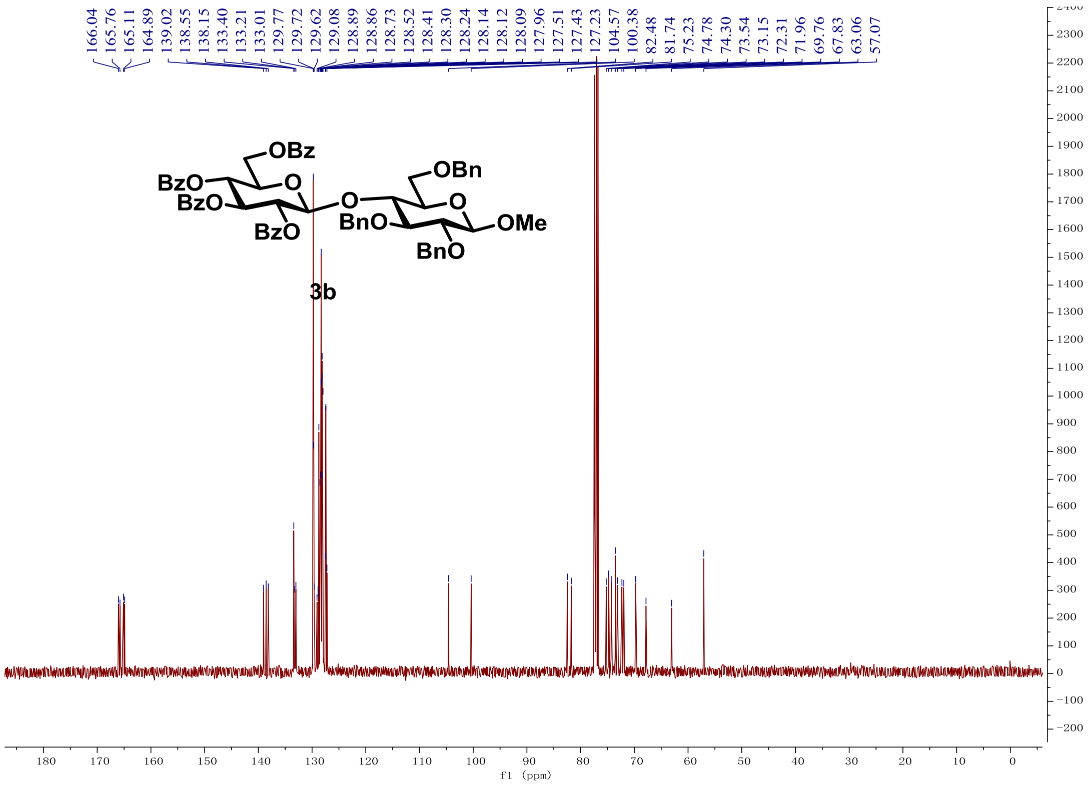

Supplementary Figure 80. ^{13}C NMR Spectrum of Compound **3b**

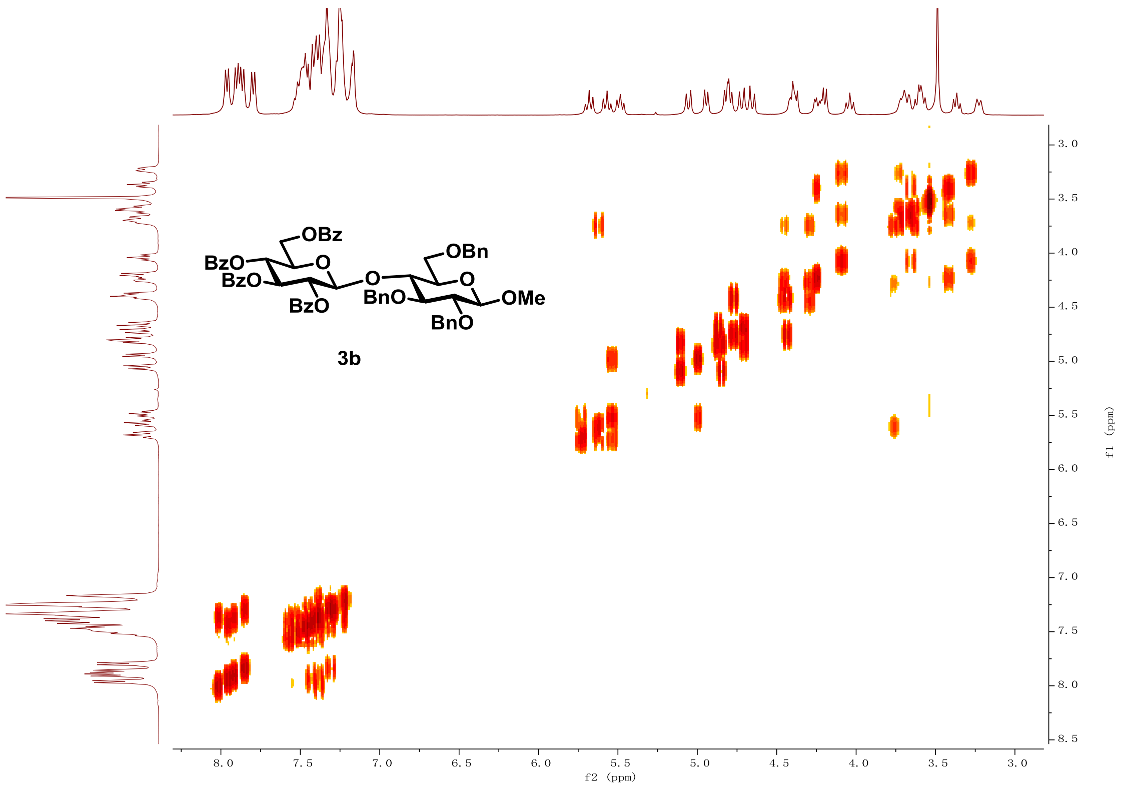

Supplementary Figure 81. COSY NMR Spectrum of Compound **3b**

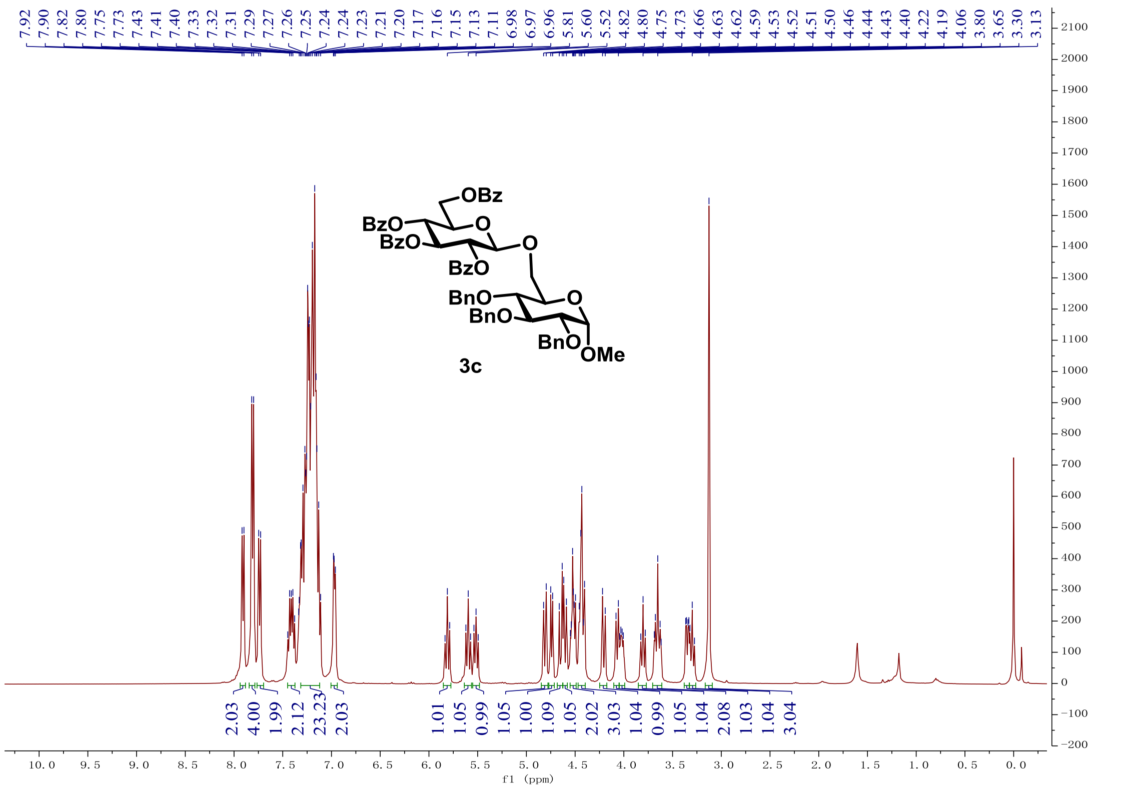

Supplementary Figure 82. ^{1}H NMR Spectrum of Compound **3c**

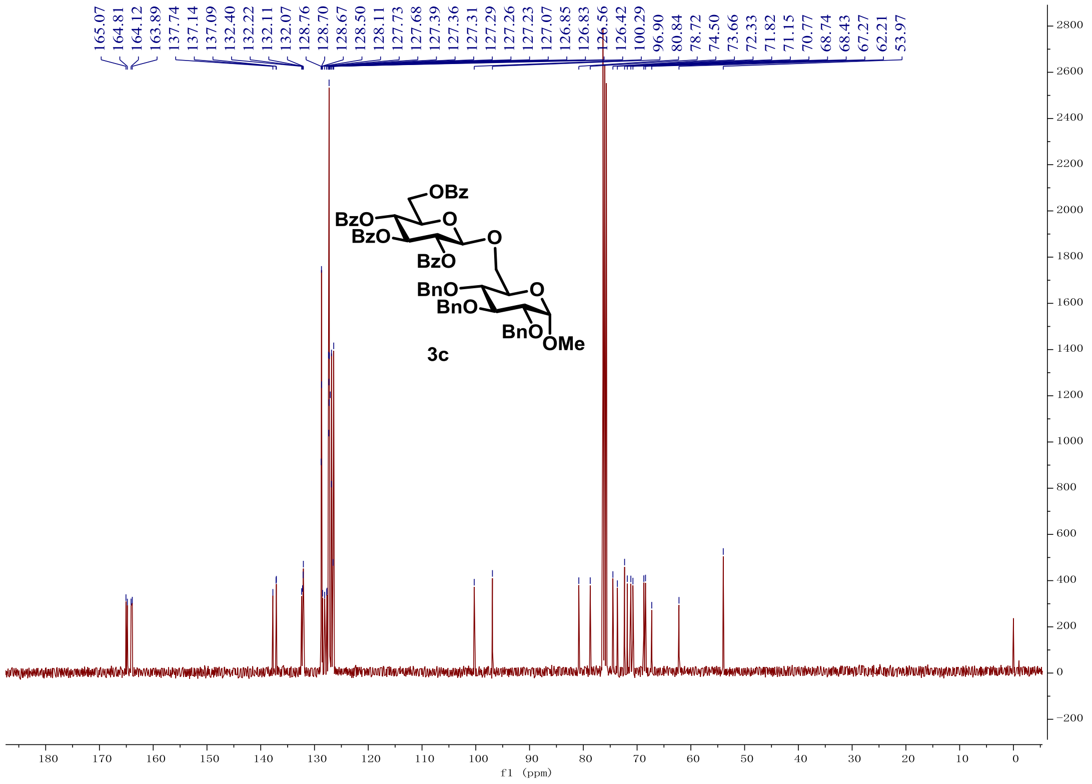

Supplementary Figure 83. ^{13}C NMR Spectrum of Compound **3c**

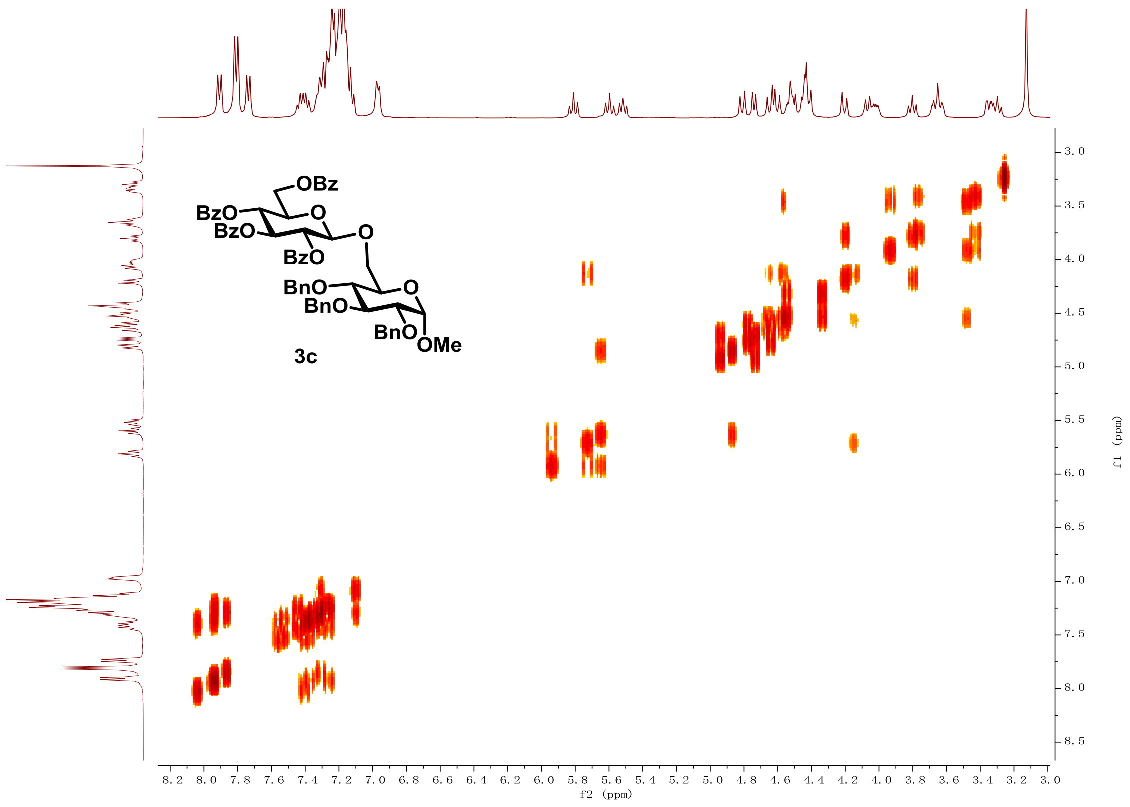

Supplementary Figure 84. COSY NMR Spectrum of Compound **3c**

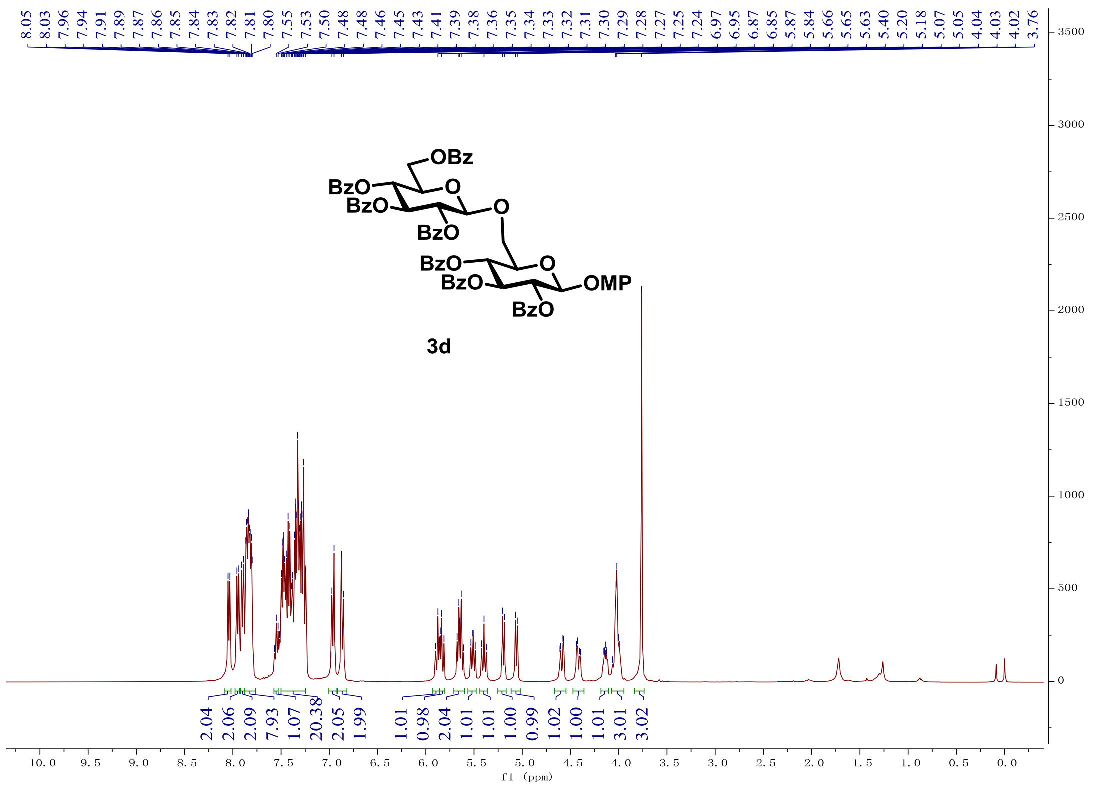

Supplementary Figure 85. ^{1}H NMR Spectrum of Compound **3d**

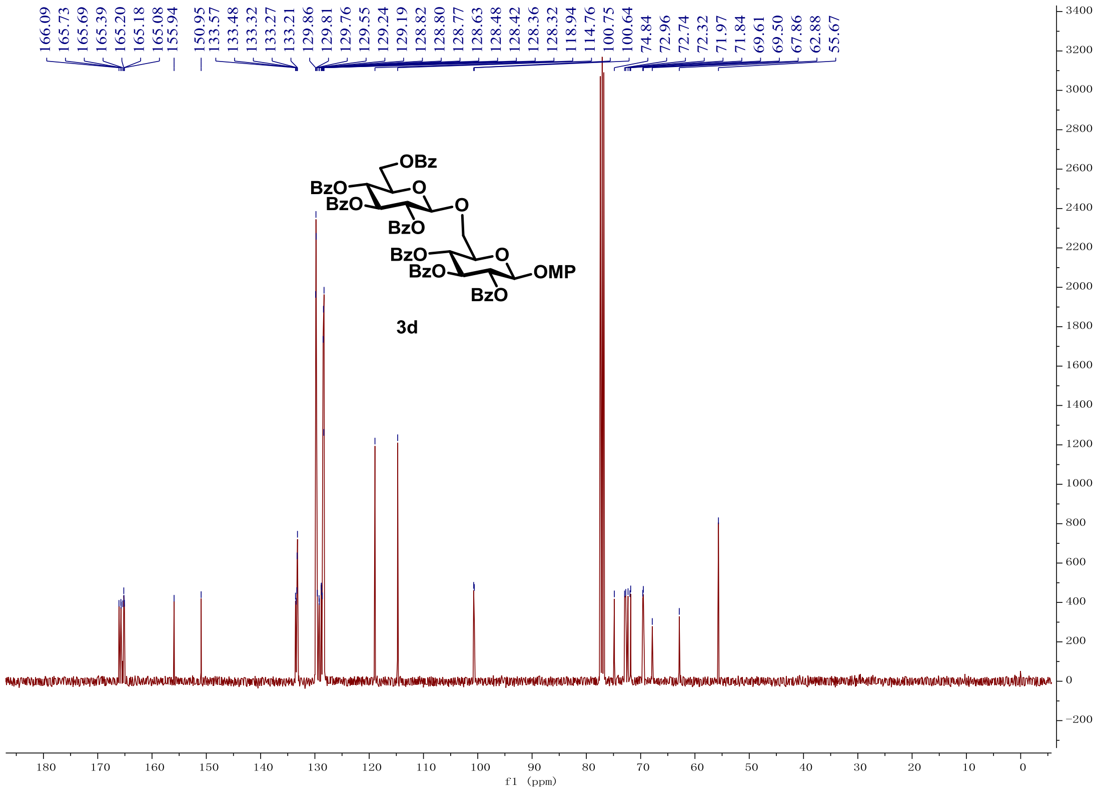

Supplementary Figure 86. ^{13}C NMR Spectrum of Compound **3d**

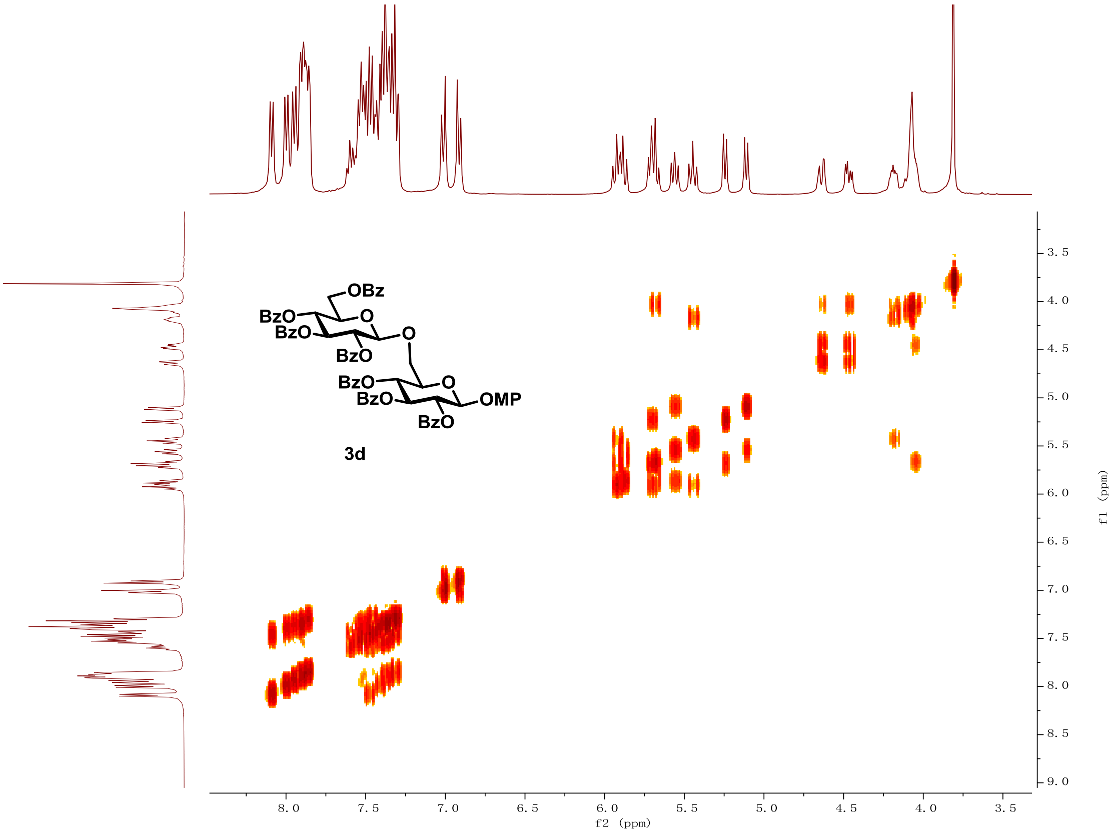

Supplementary Figure 87. COSY NMR Spectrum of Compound **3d**

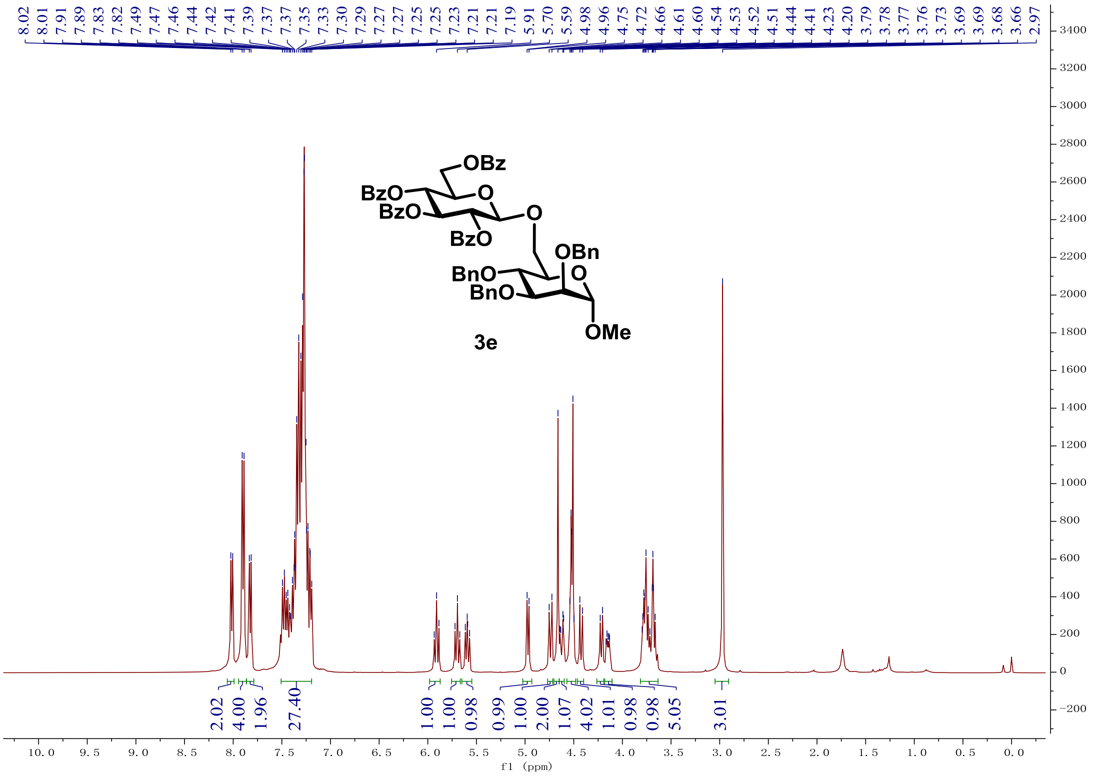

Supplementary Figure 88. ^{1}H NMR Spectrum of Compound **3e**

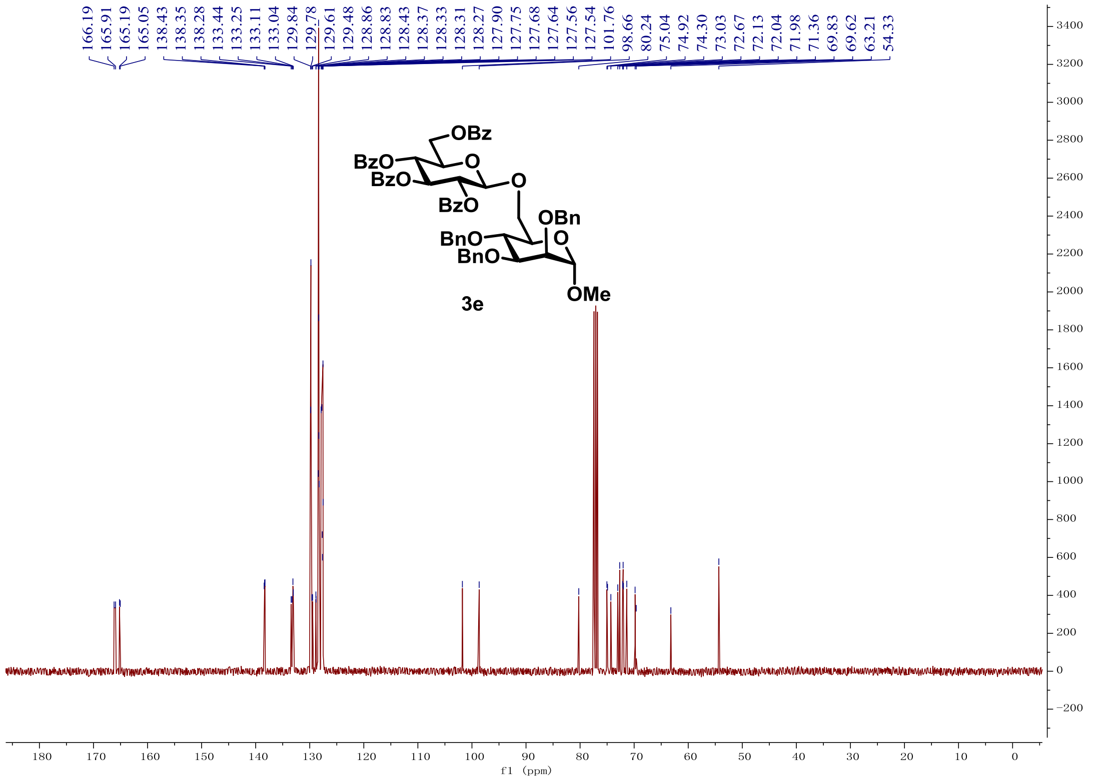

Supplementary Figure 89. ^{13}C NMR Spectrum of Compound **3e**

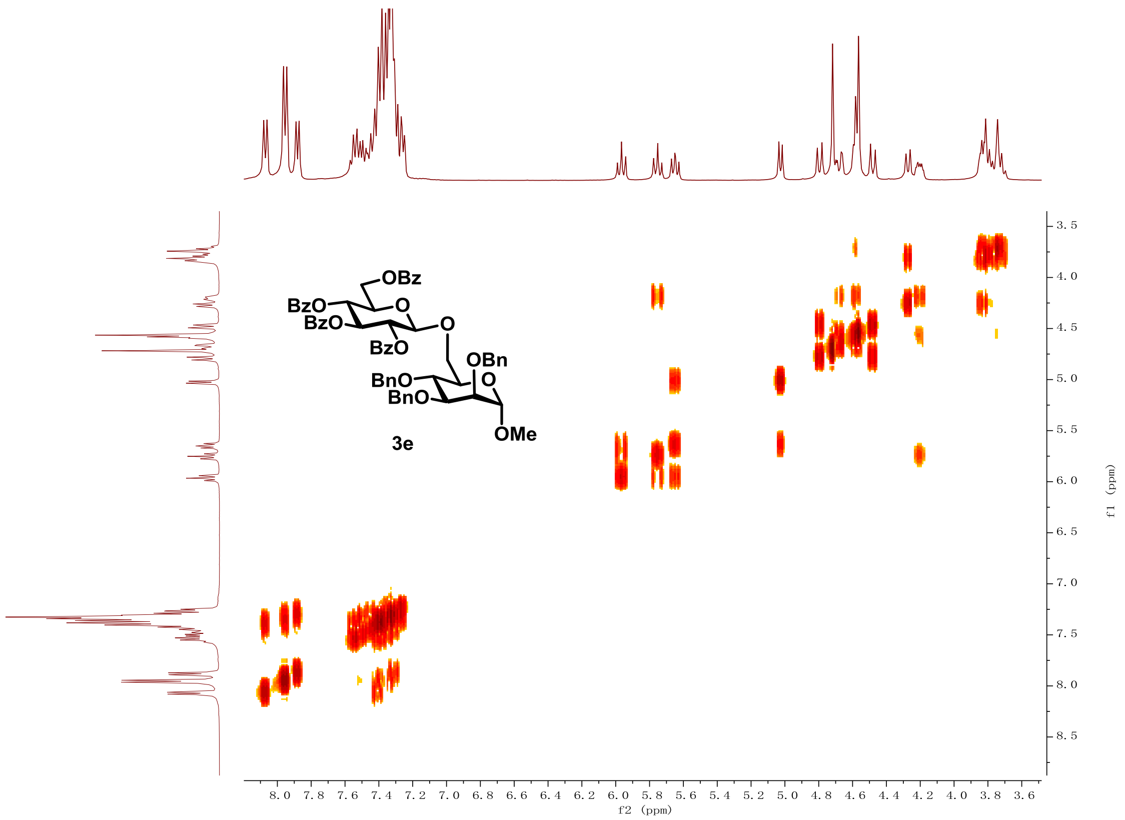

Supplementary Figure 90. COSY NMR Spectrum of Compound **3e**

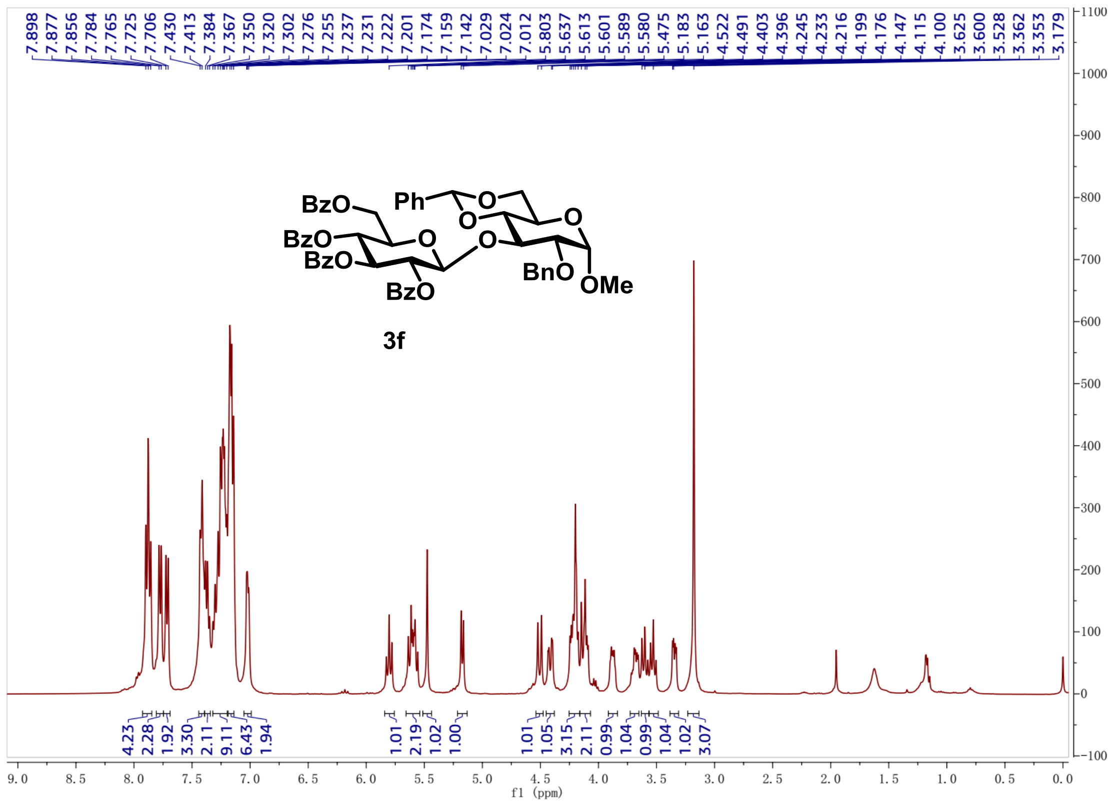

Supplementary Figure 91. ^{1}H NMR Spectrum of Compound **3f**

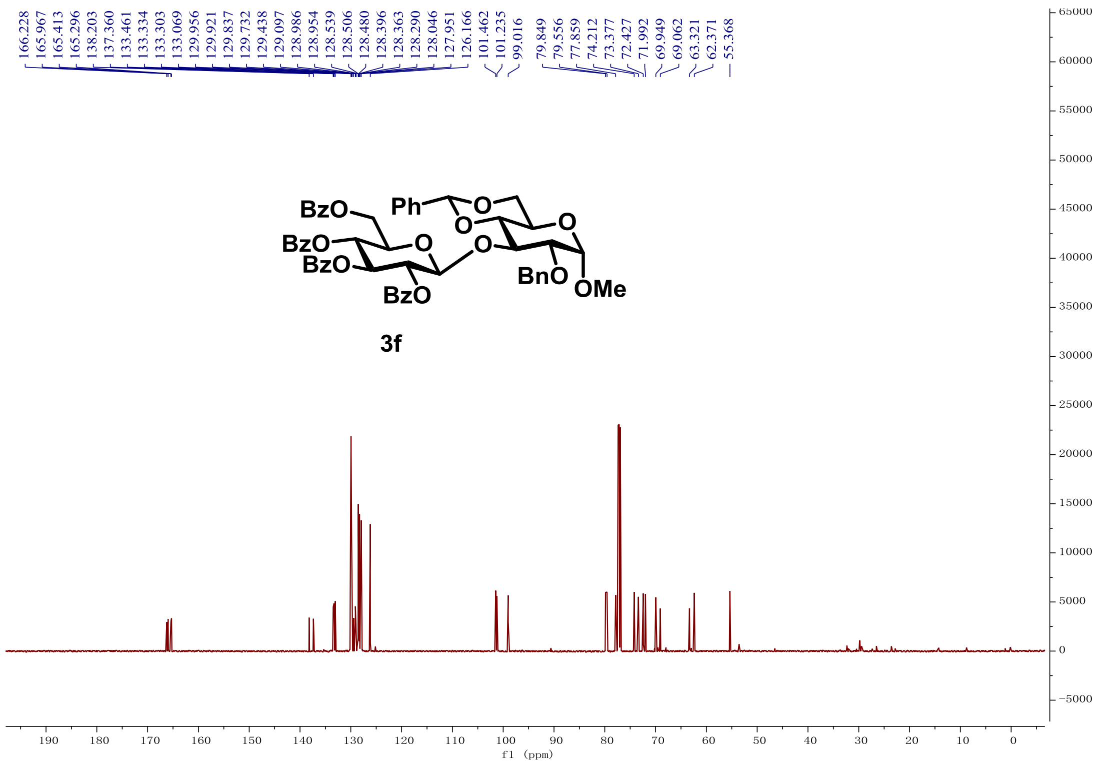

Supplementary Figure 92. ^{13}C NMR Spectrum of Compound **3f**

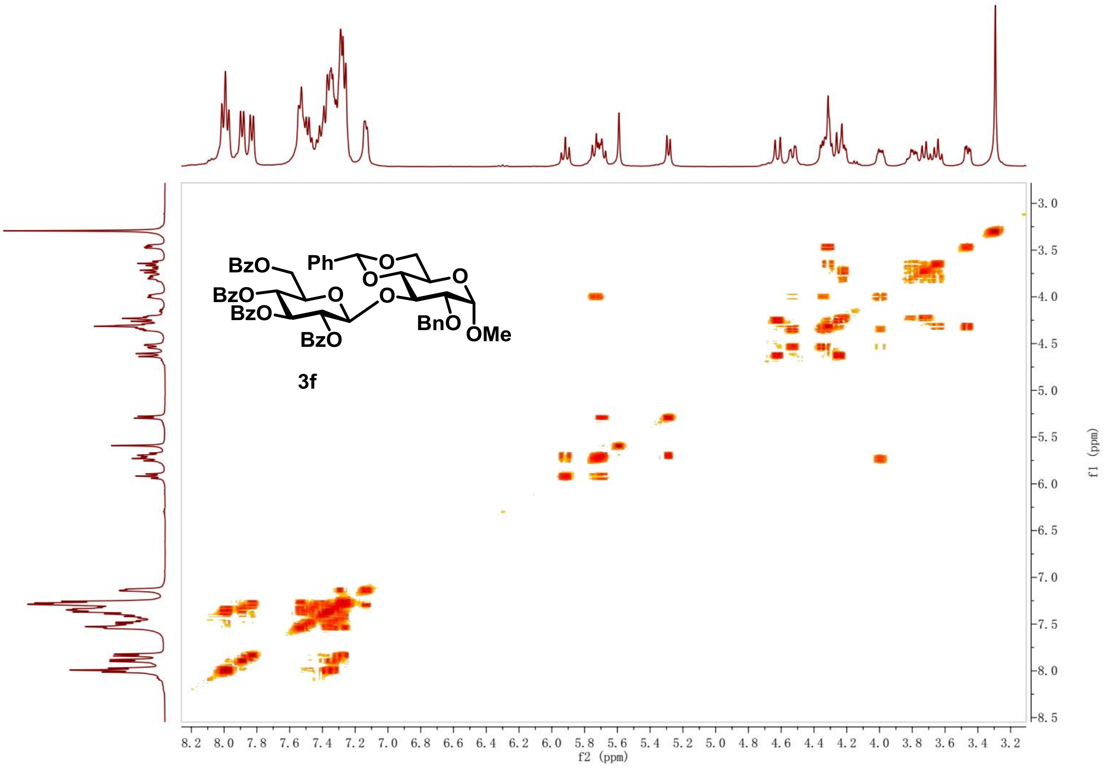

Supplementary Figure 93. COSY NMR Spectrum of Compound **3f**

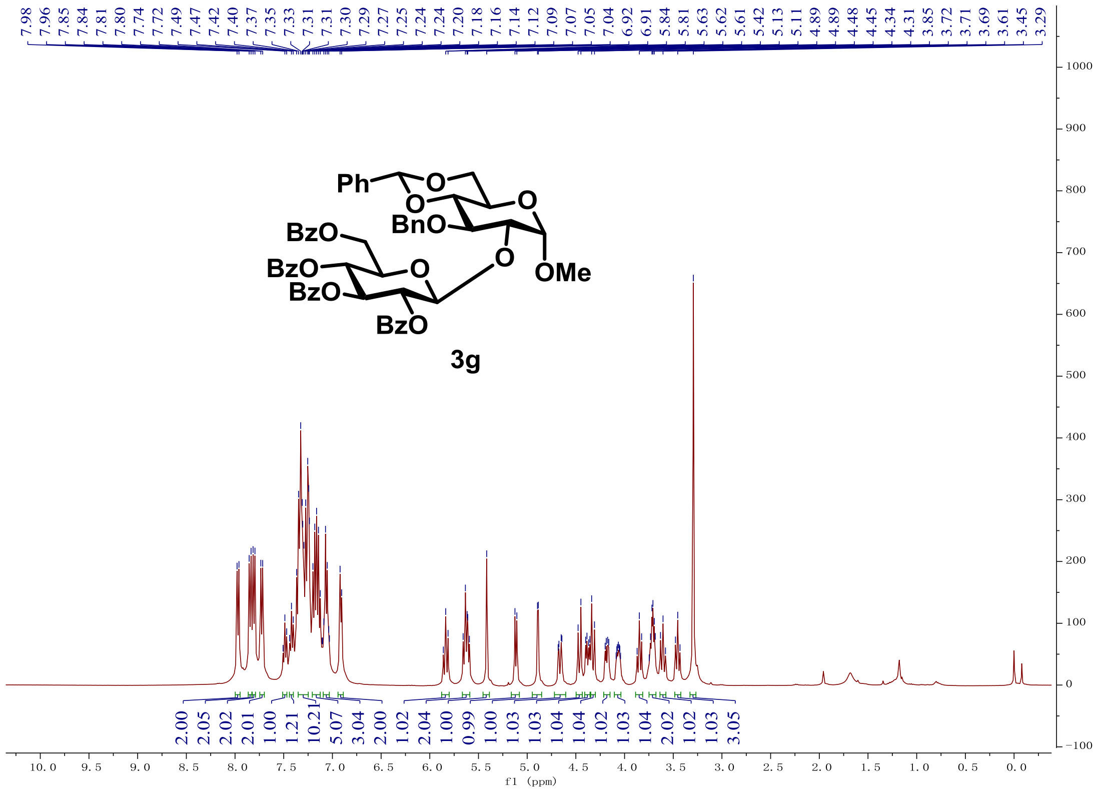

Supplementary Figure 94. ^{1}H NMR Spectrum of Compound **3g**

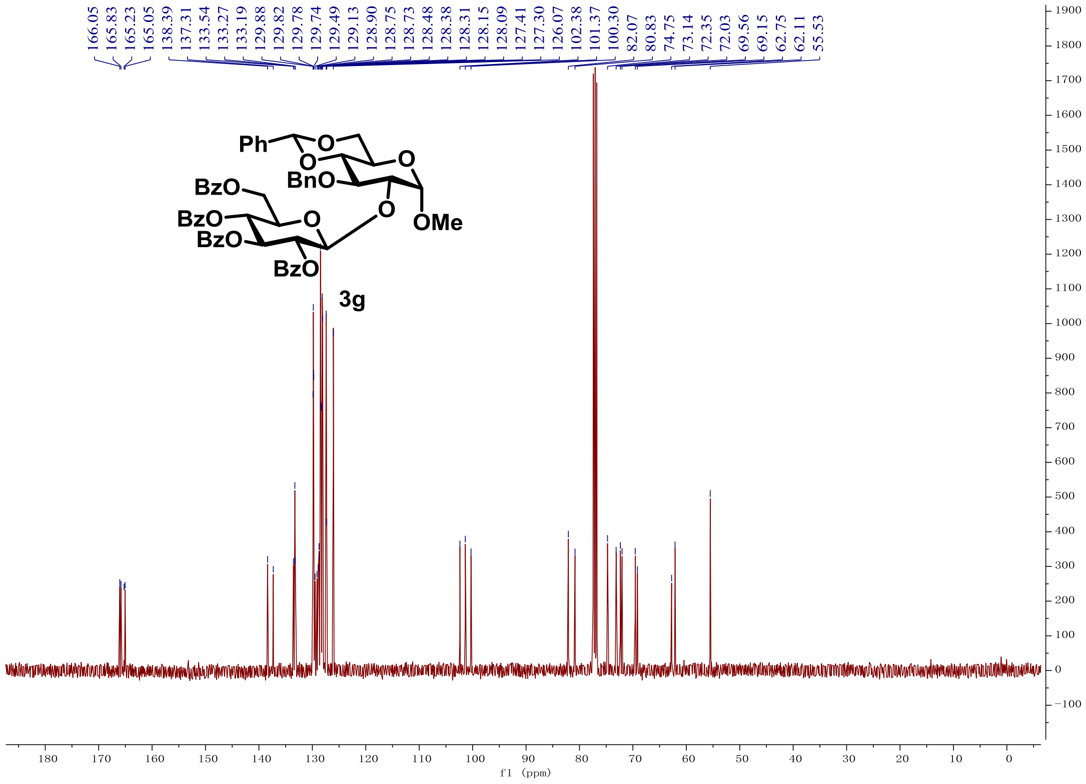

Supplementary Figure 95. ^{13}C NMR Spectrum of Compound **3g**

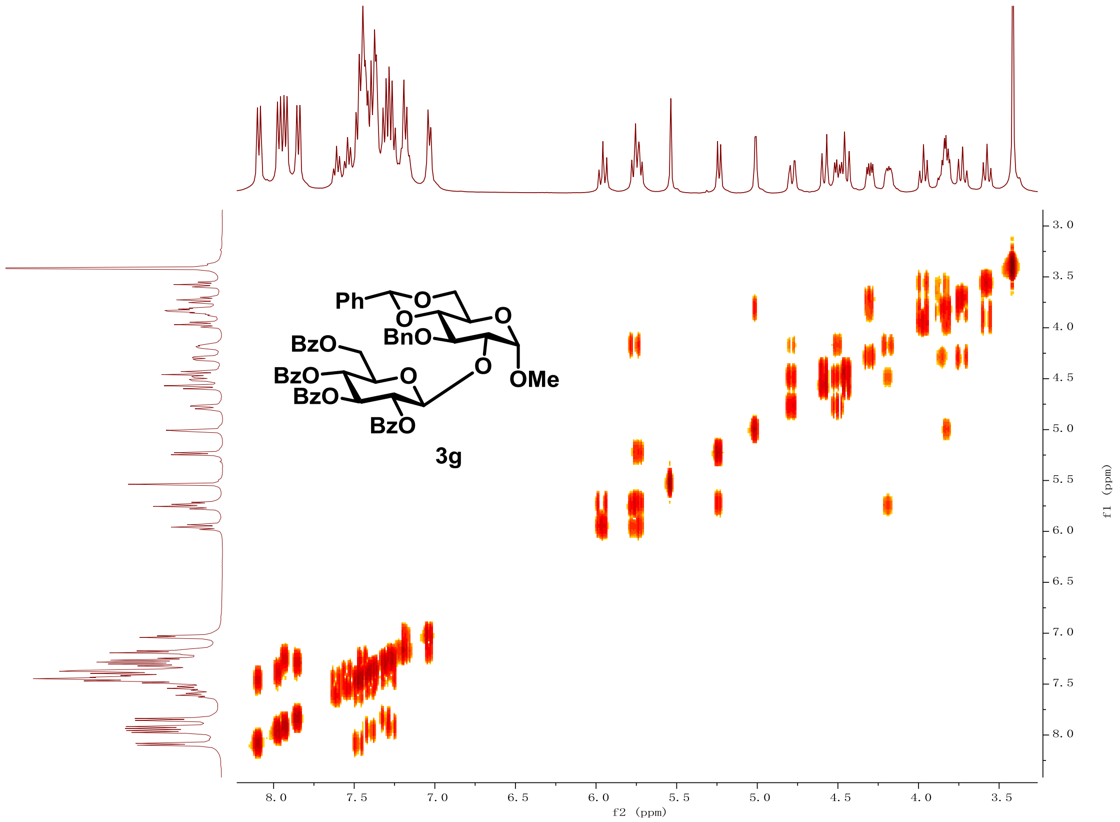

Supplementary Figure 96. COSY NMR Spectrum of Compound **3g**

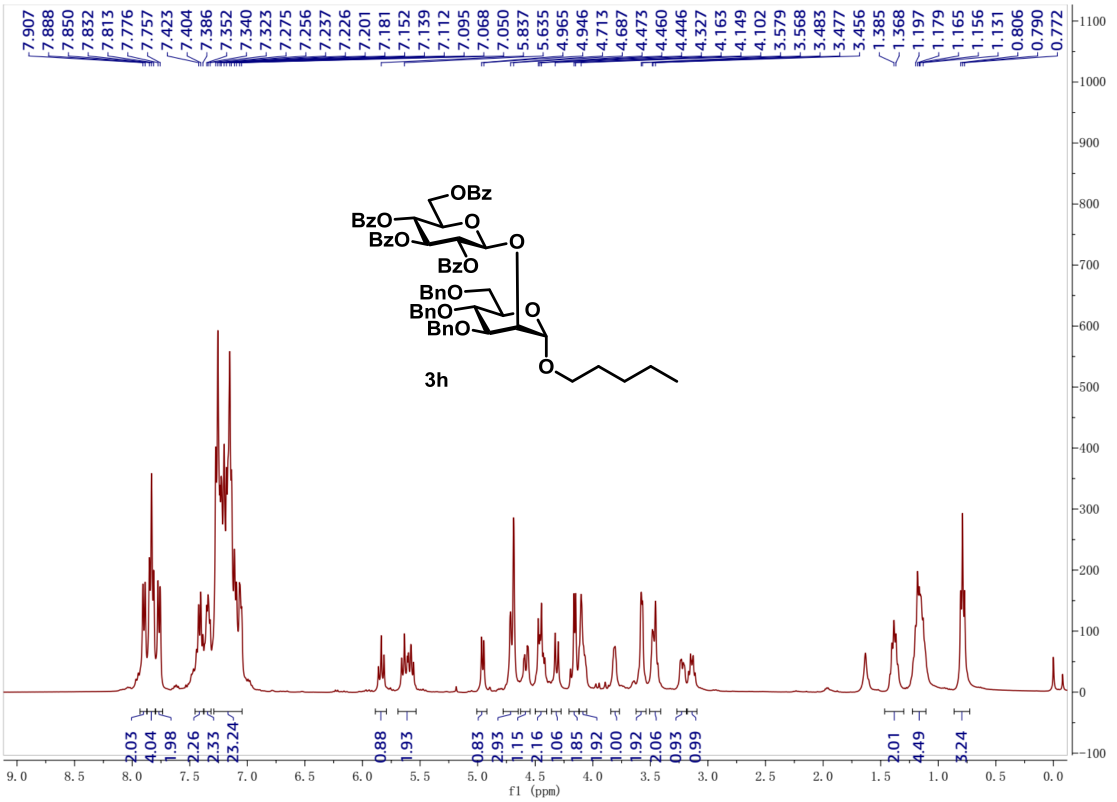

Supplementary Figure 97. ^{1}H NMR Spectrum of Compound **3h**

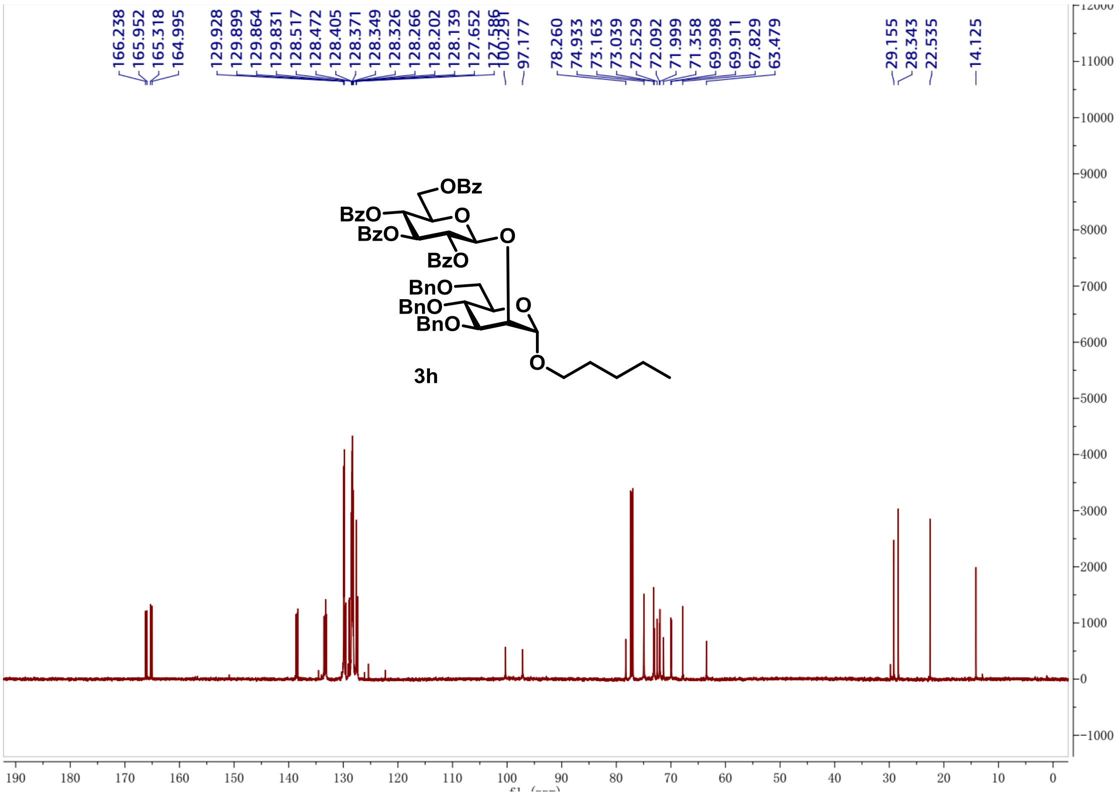

Supplementary Figure 98. ^{13}C NMR Spectrum of Compound **3h**

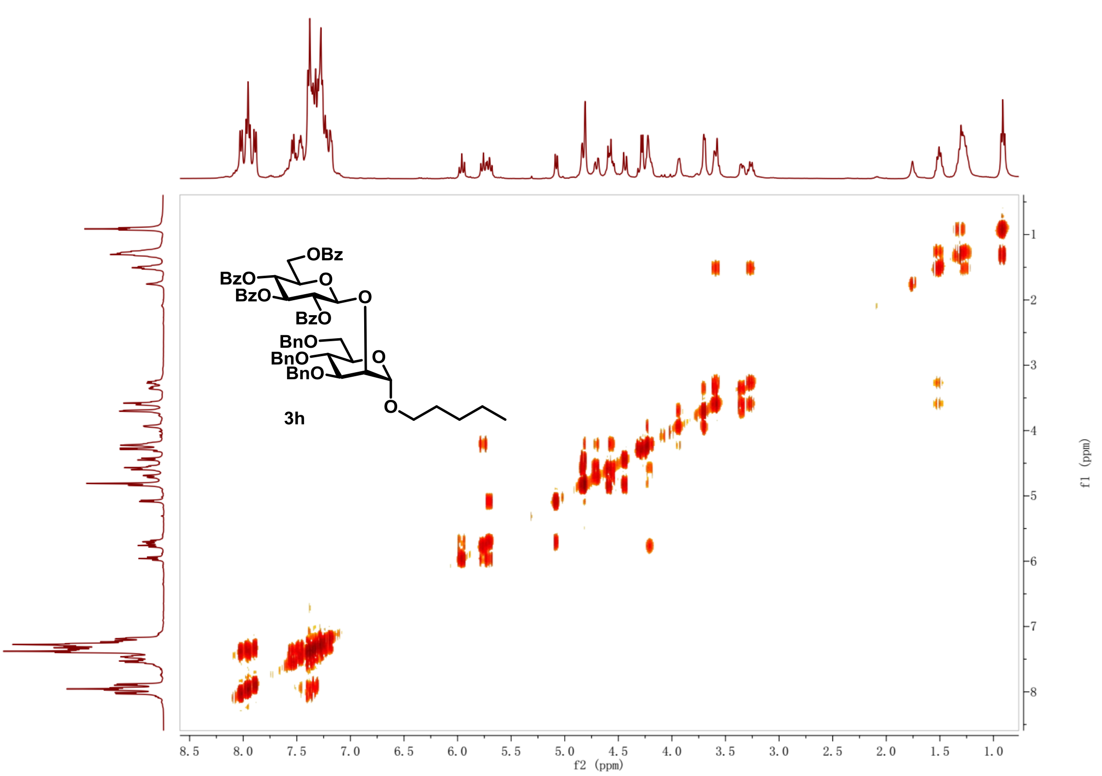

Supplementary Figure 99. COSY NMR Spectrum of Compound **3h**

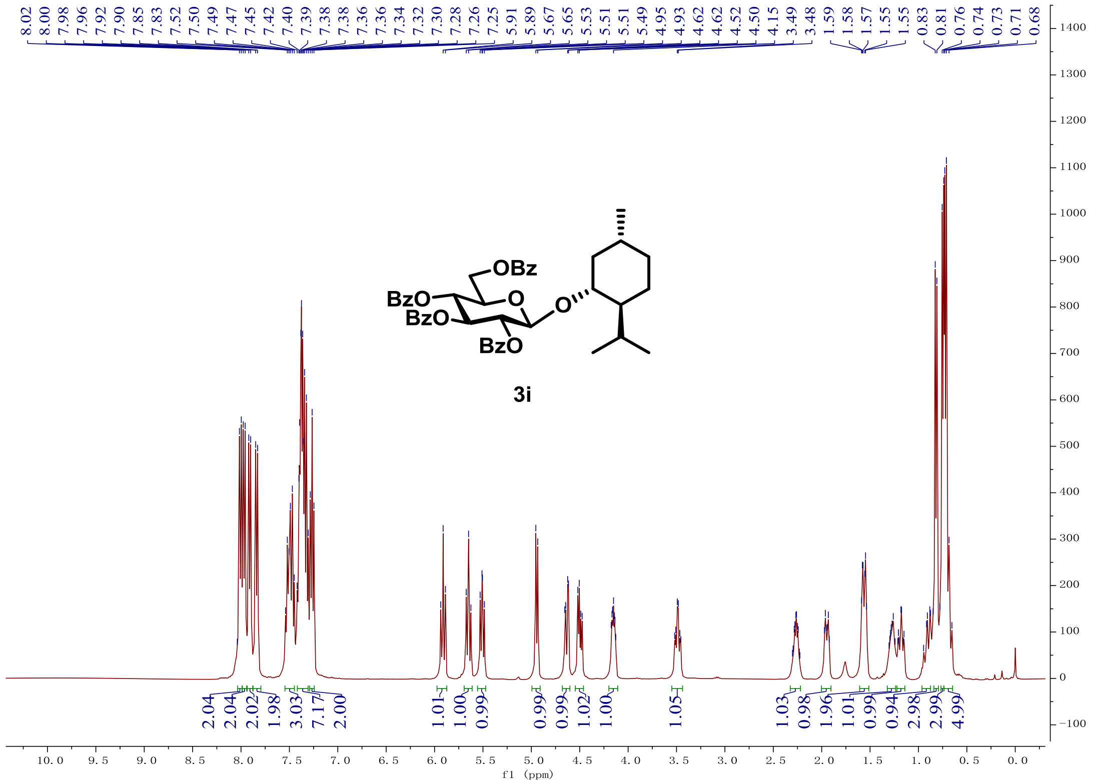

Supplementary Figure 100. ^{1}H NMR Spectrum of Compound **3i**

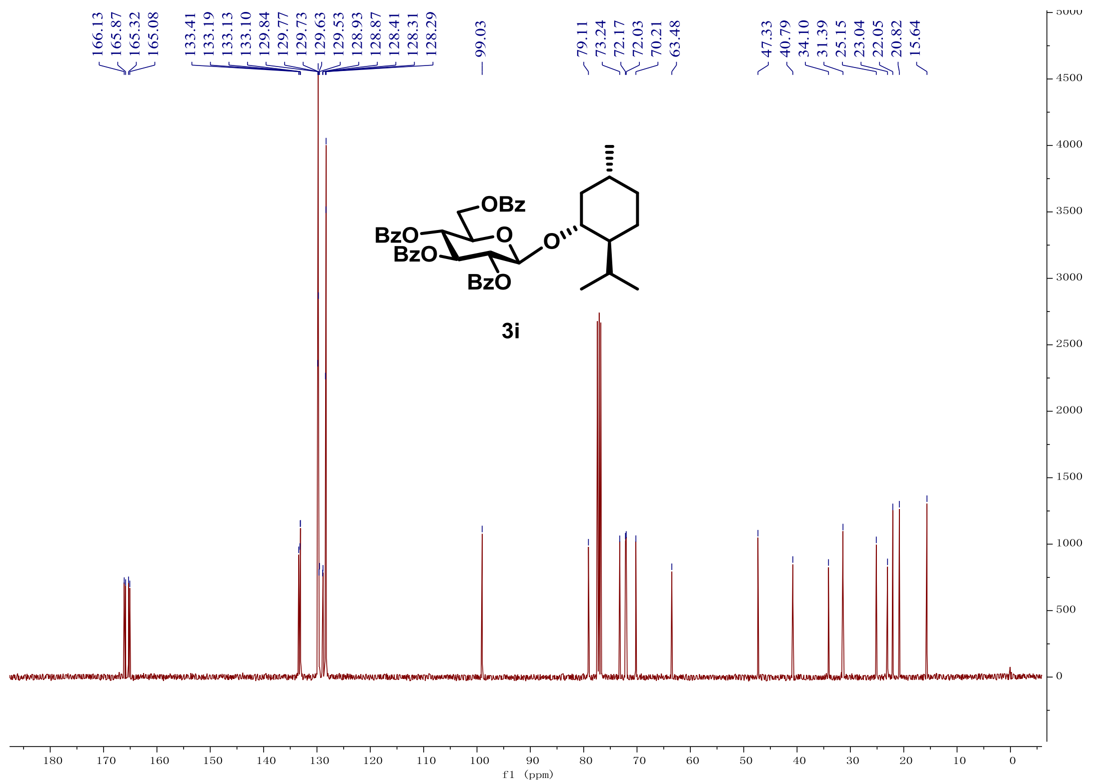

Supplementary Figure 101. ^{13}C NMR Spectrum of Compound **3i**

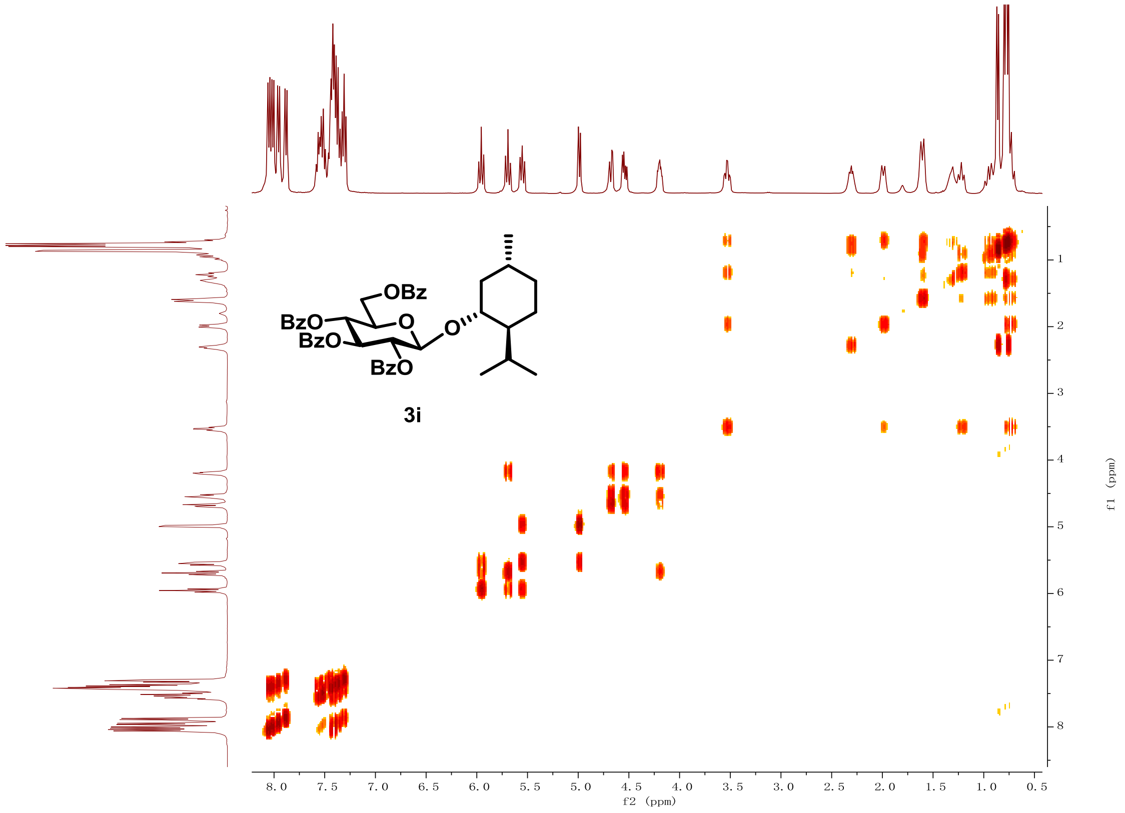

Supplementary Figure 102. COSY NMR Spectrum of Compound **3i**

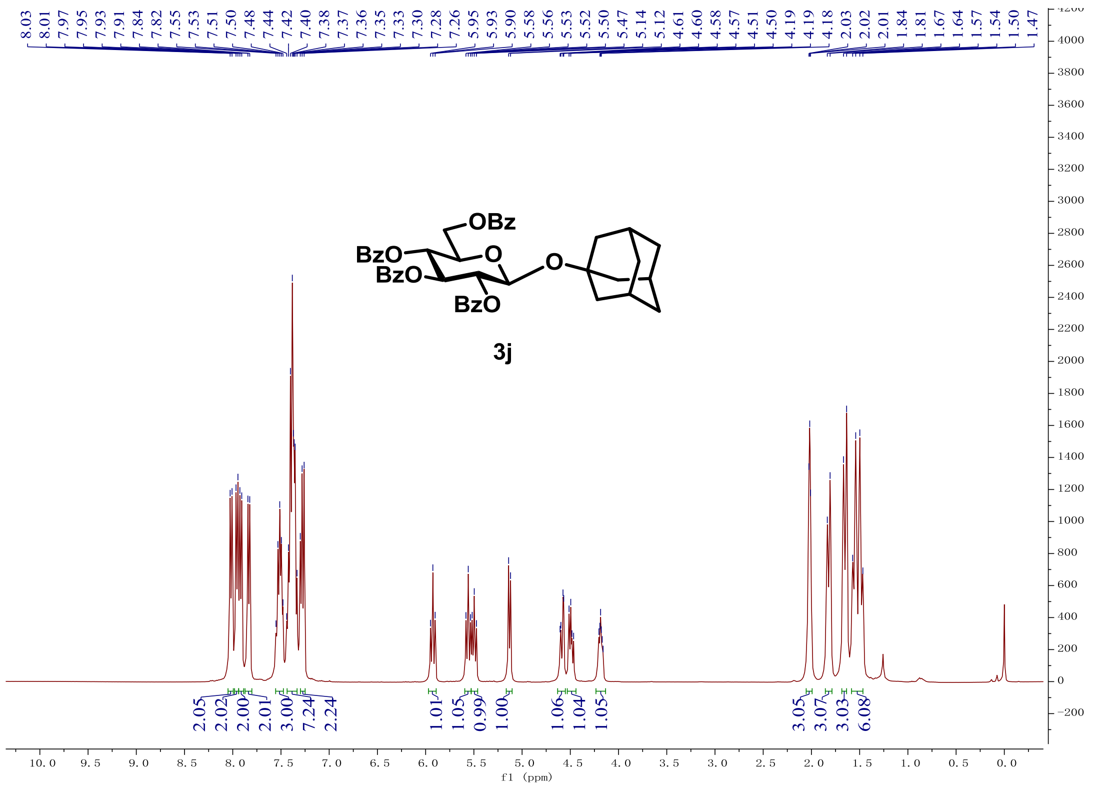

Supplementary Figure 103. ^{1}H NMR Spectrum of Compound **3j**

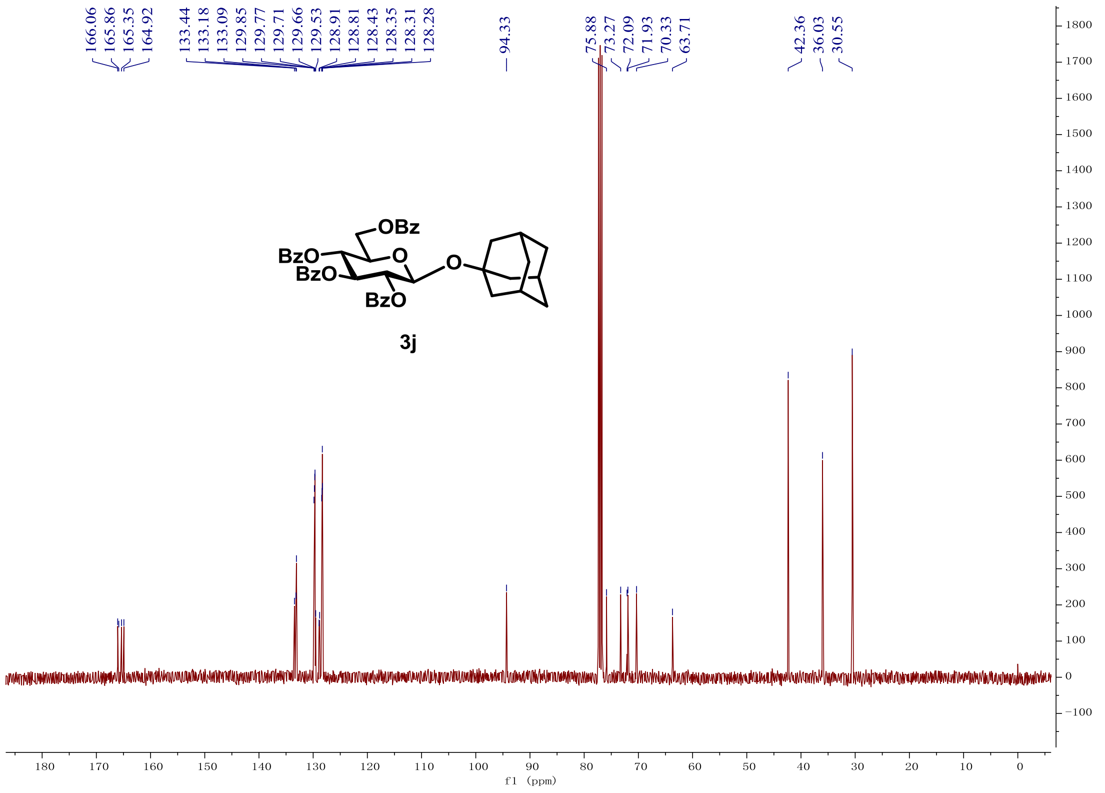

Supplementary Figure 104. ^{13}C NMR Spectrum of Compound **3j**

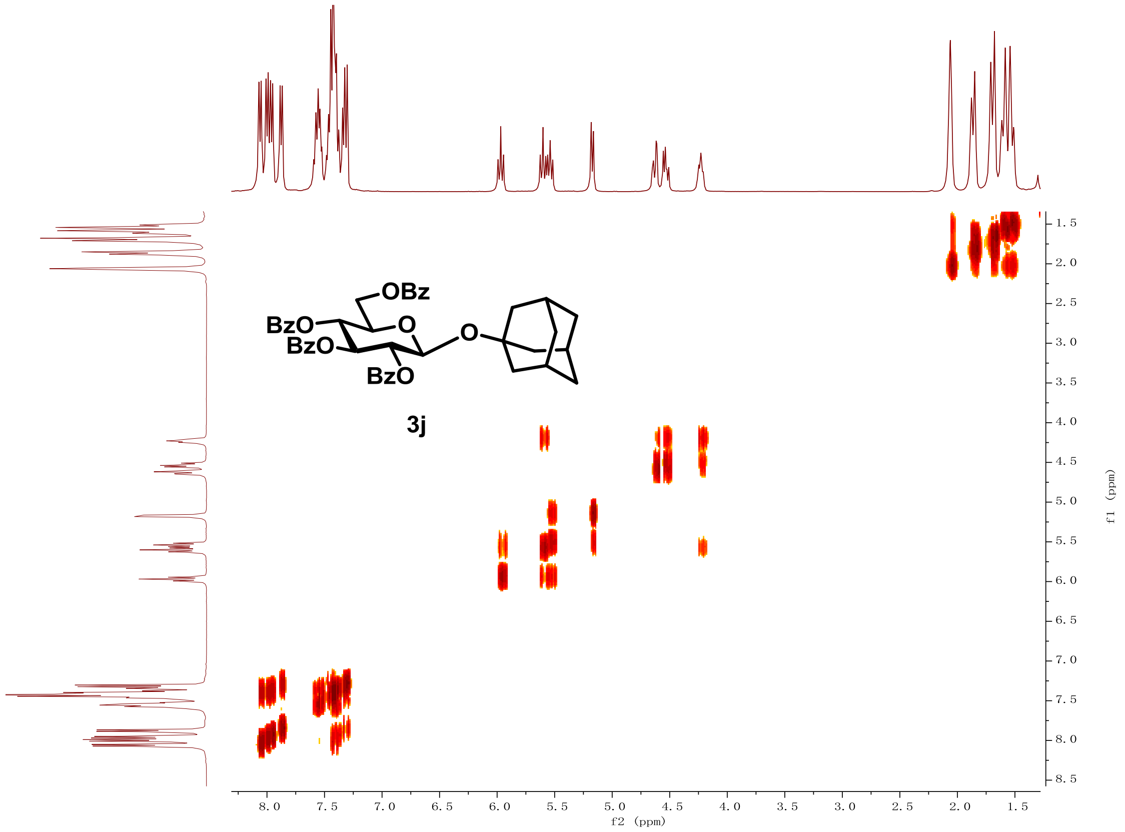

Supplementary Figure 105. COSY NMR Spectrum of Compound **3j**

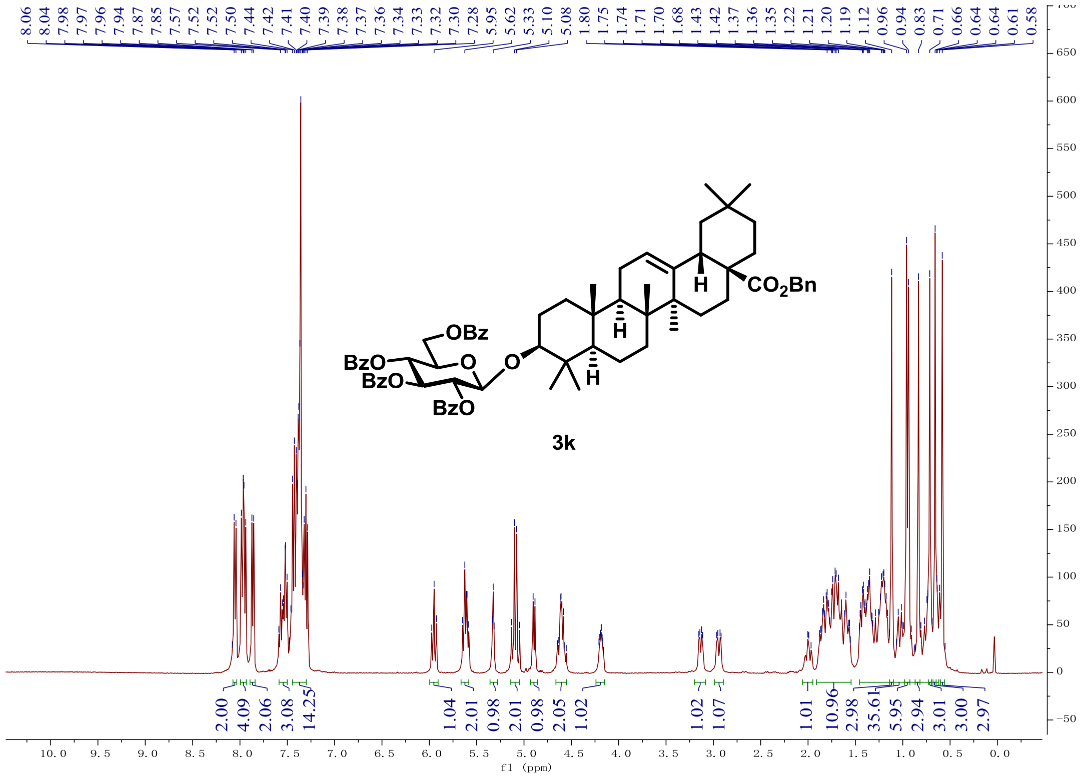

Supplementary Figure 106. ^{1}H NMR Spectrum of Compound **3k**

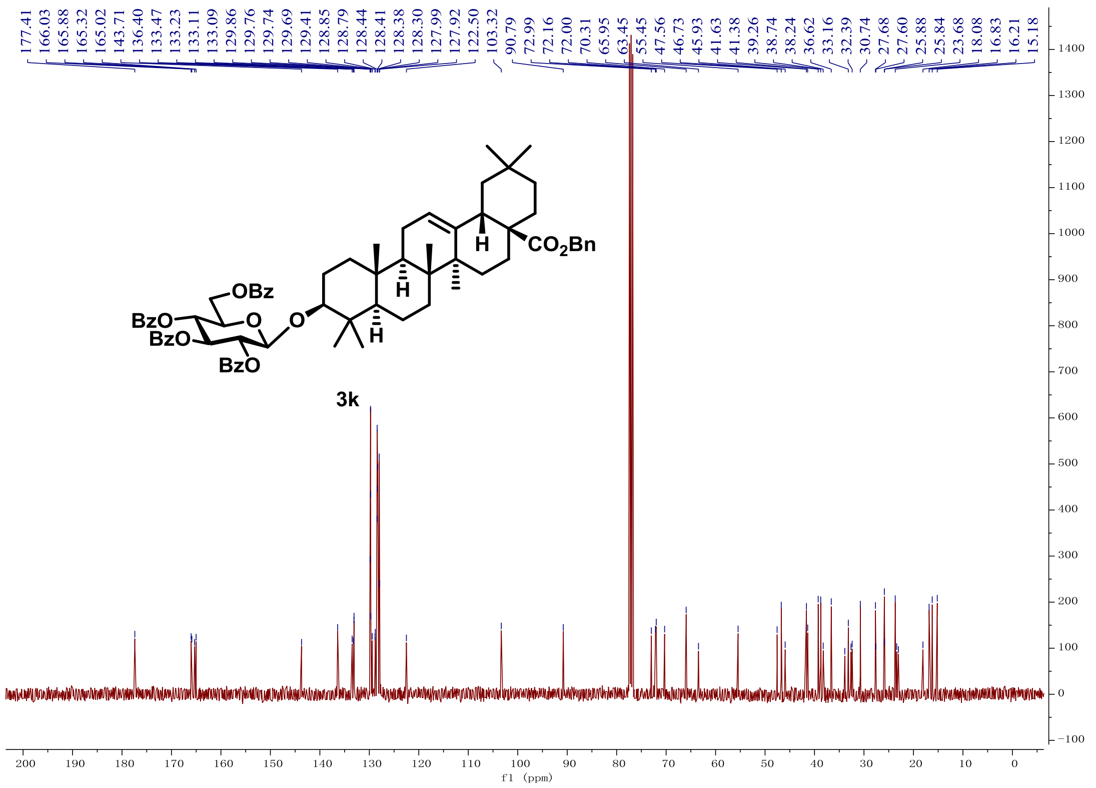

Supplementary Figure 107. ^{13}C NMR Spectrum of Compound **3k**

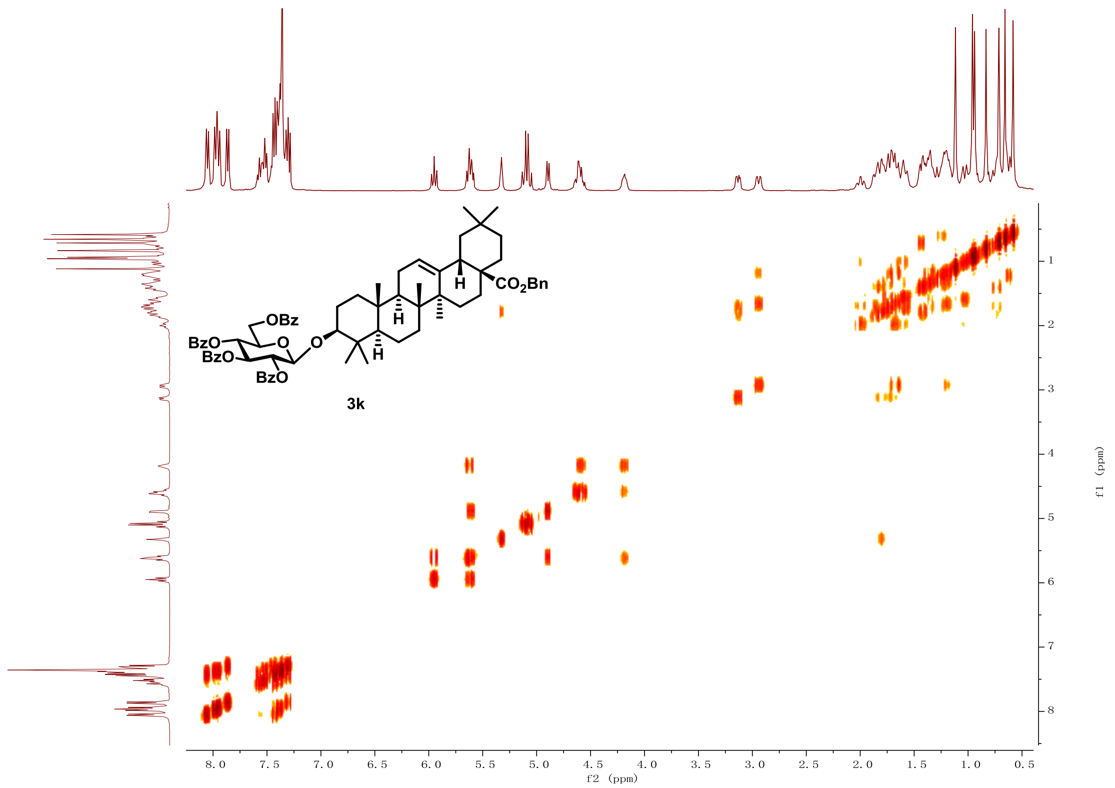

Supplementary Figure 108. COSY NMR Spectrum of Compound **3k**

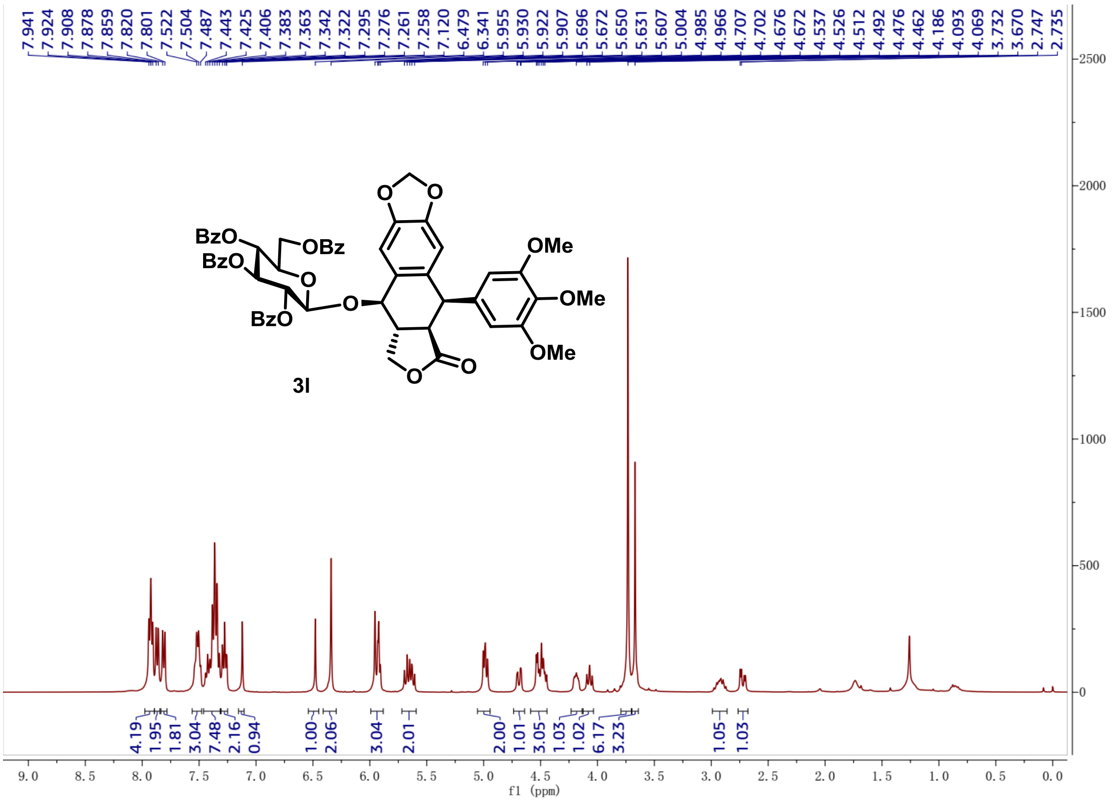

Supplementary Figure 109. ^{1}H NMR Spectrum of Compound **3l**

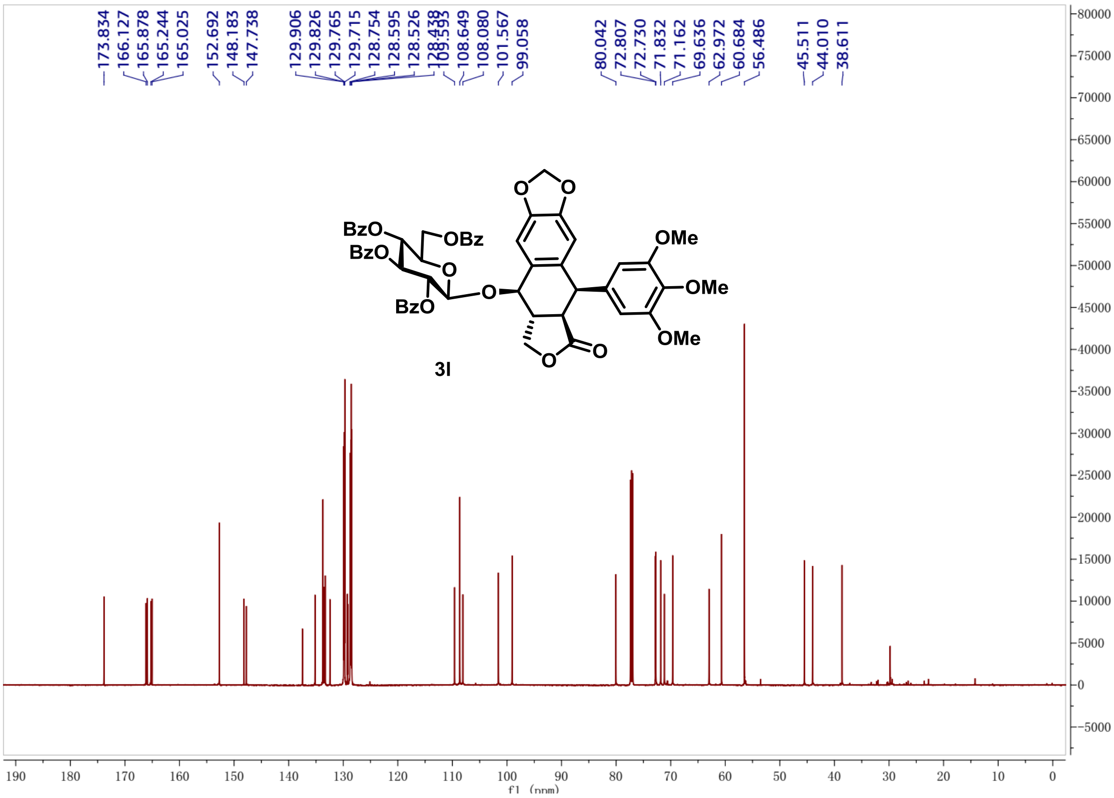

Supplementary Figure 110. ^{13}C NMR Spectrum of Compound **3l**

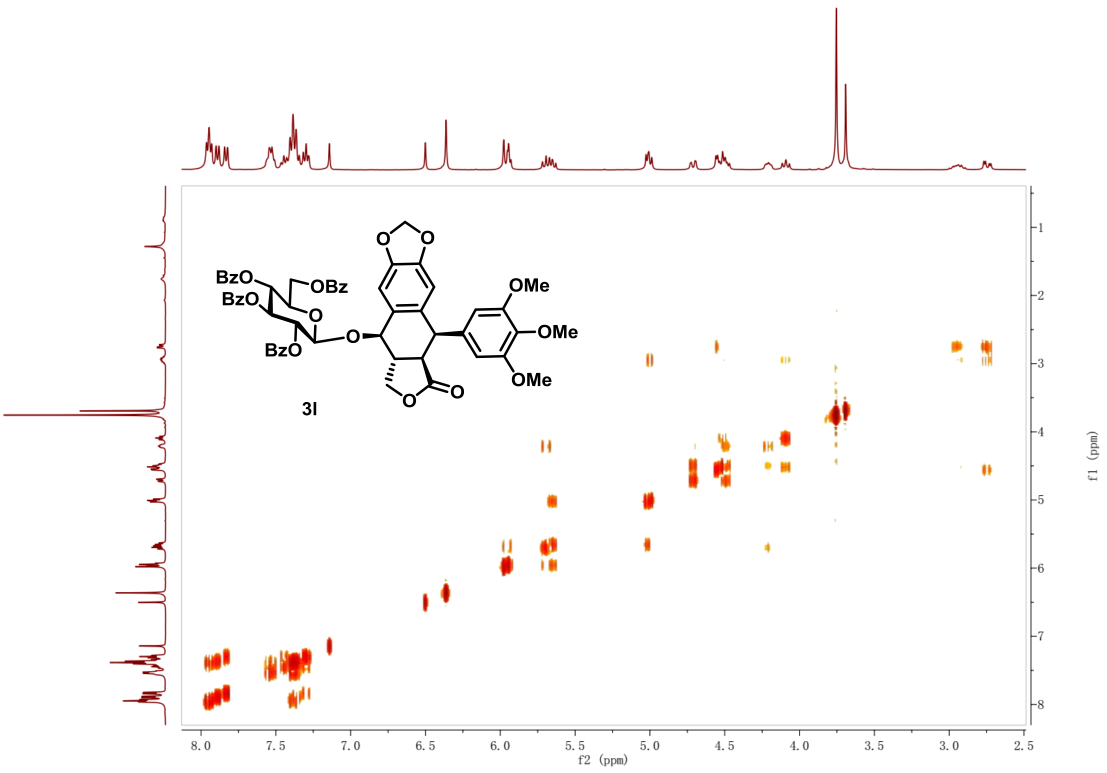

Supplementary Figure 111. COSY NMR Spectrum of Compound **3l**

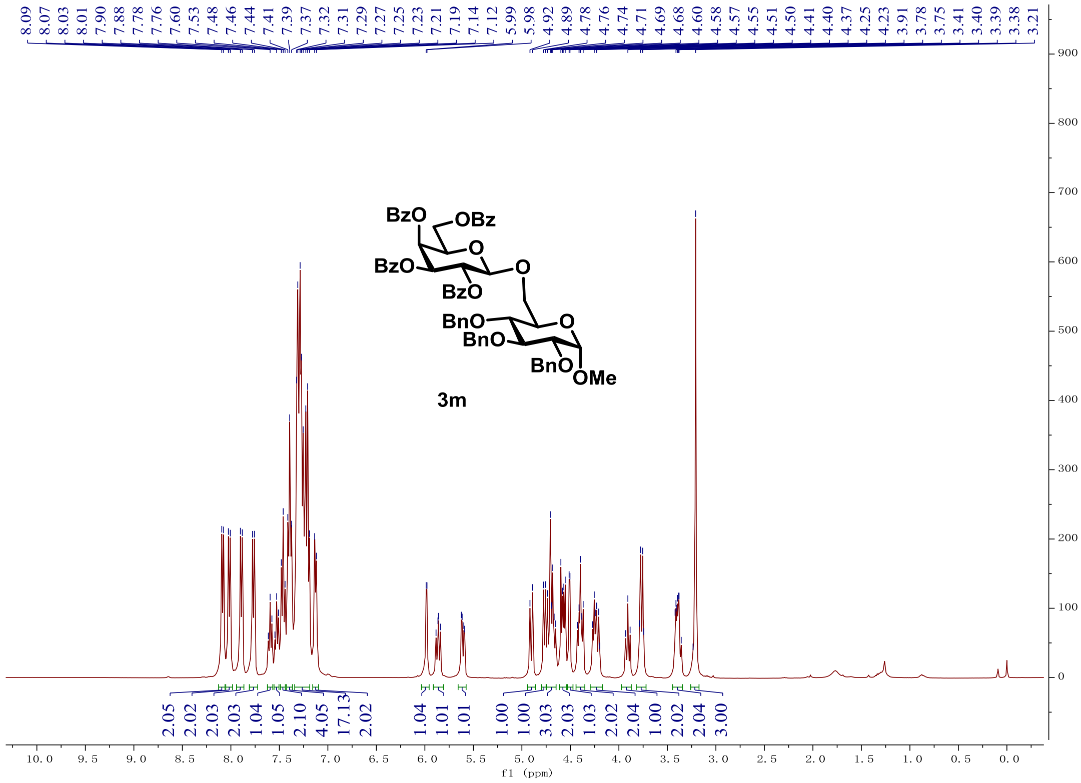

Supplementary Figure 112. ^{1}H NMR Spectrum of Compound **3m**

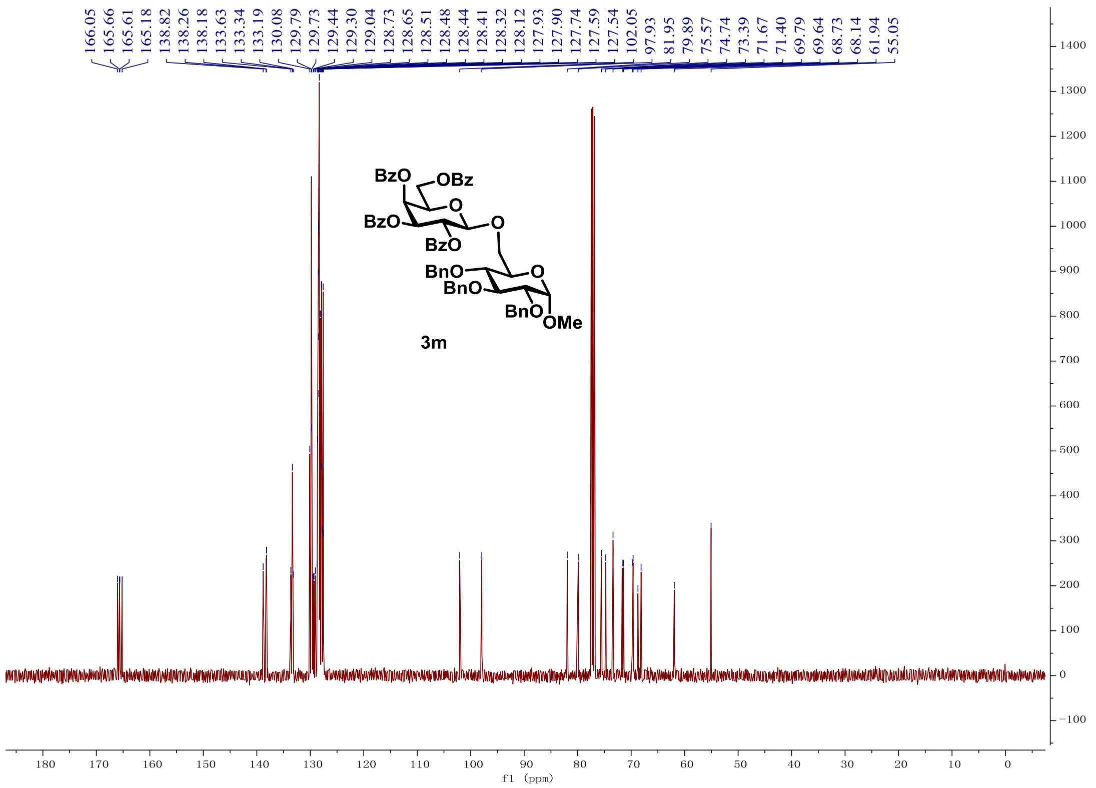

Supplementary Figure 113. ^{13}C NMR Spectrum of Compound **3m**

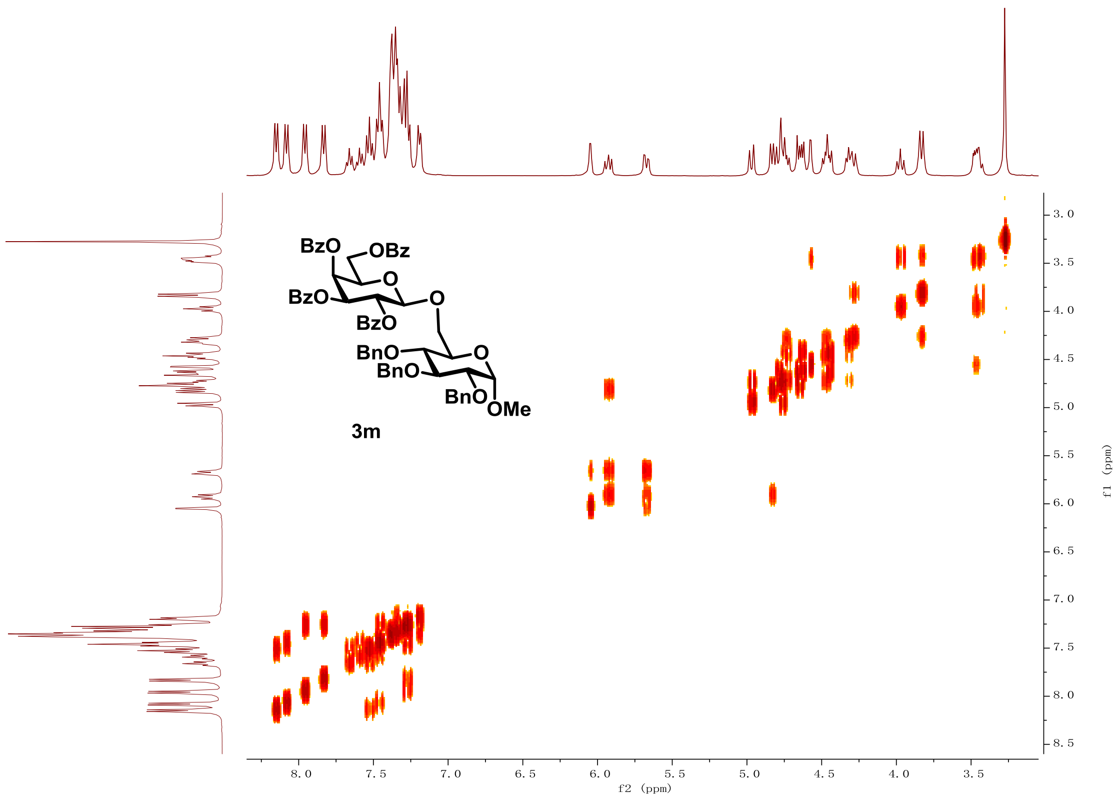

Supplementary Figure 114. COSY NMR Spectrum of Compound **3m**

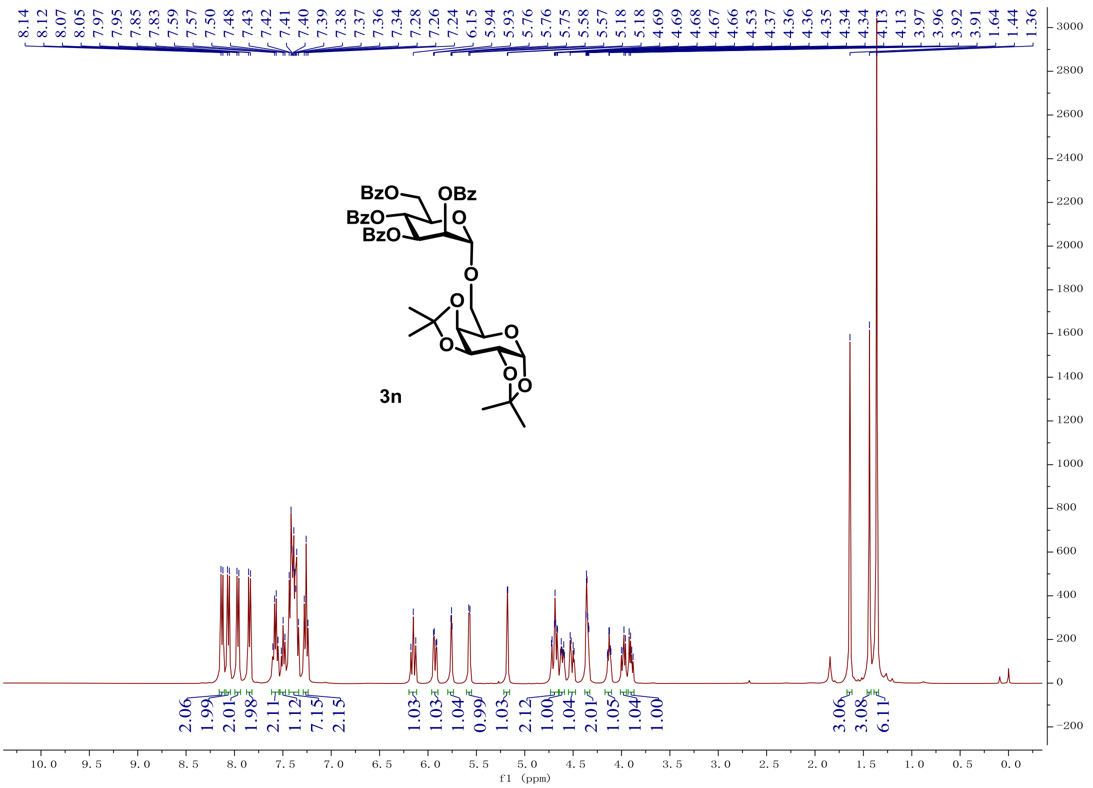

Supplementary Figure 115. ^{1}H NMR Spectrum of Compound **3n**

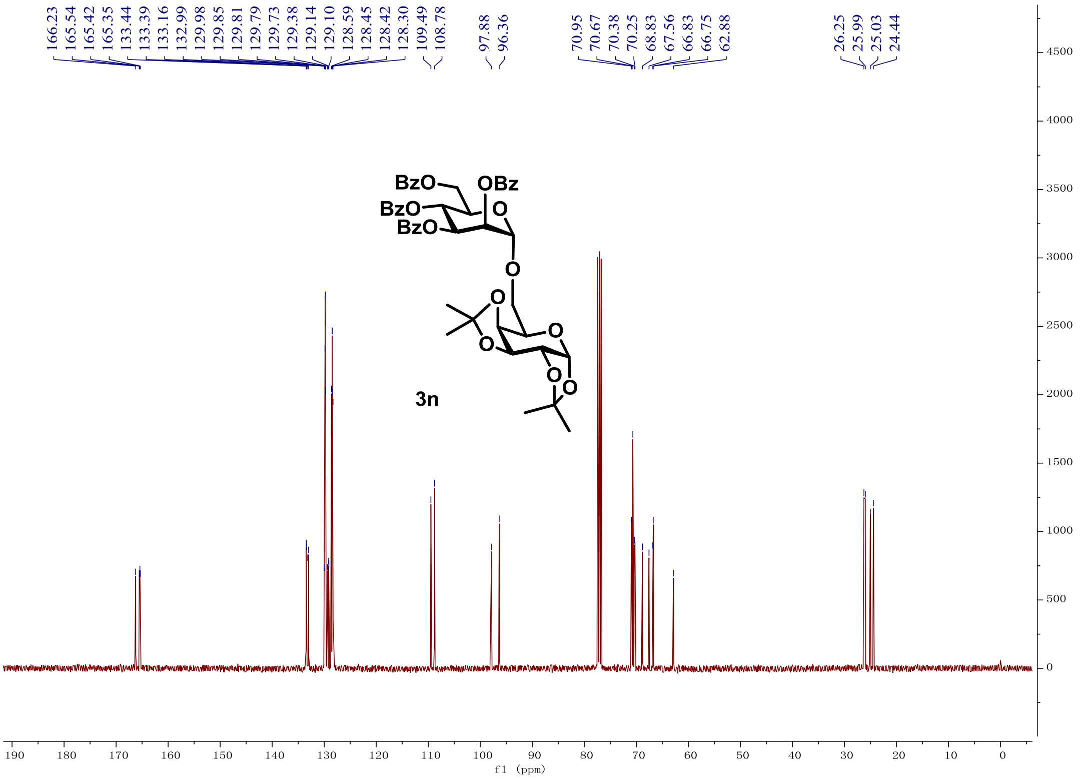

Supplementary Figure 116. ^{13}C NMR Spectrum of Compound **3n**

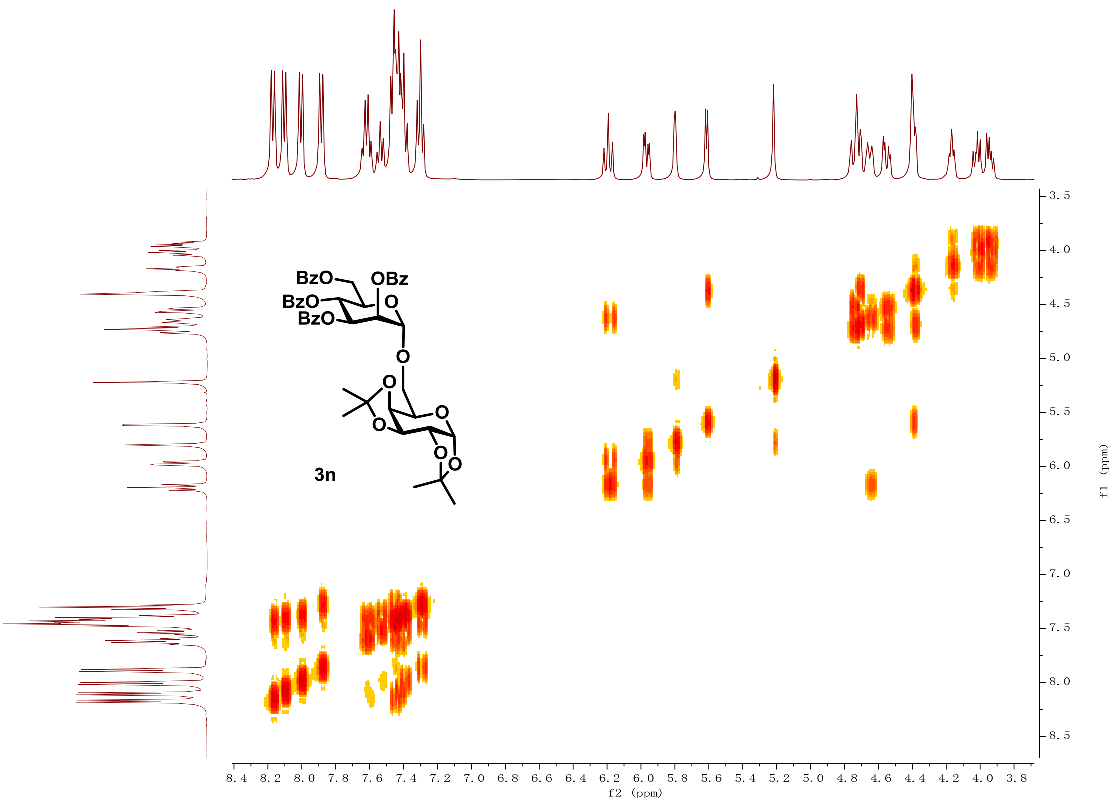

Supplementary Figure 117. COSY NMR Spectrum of Compound **3n**

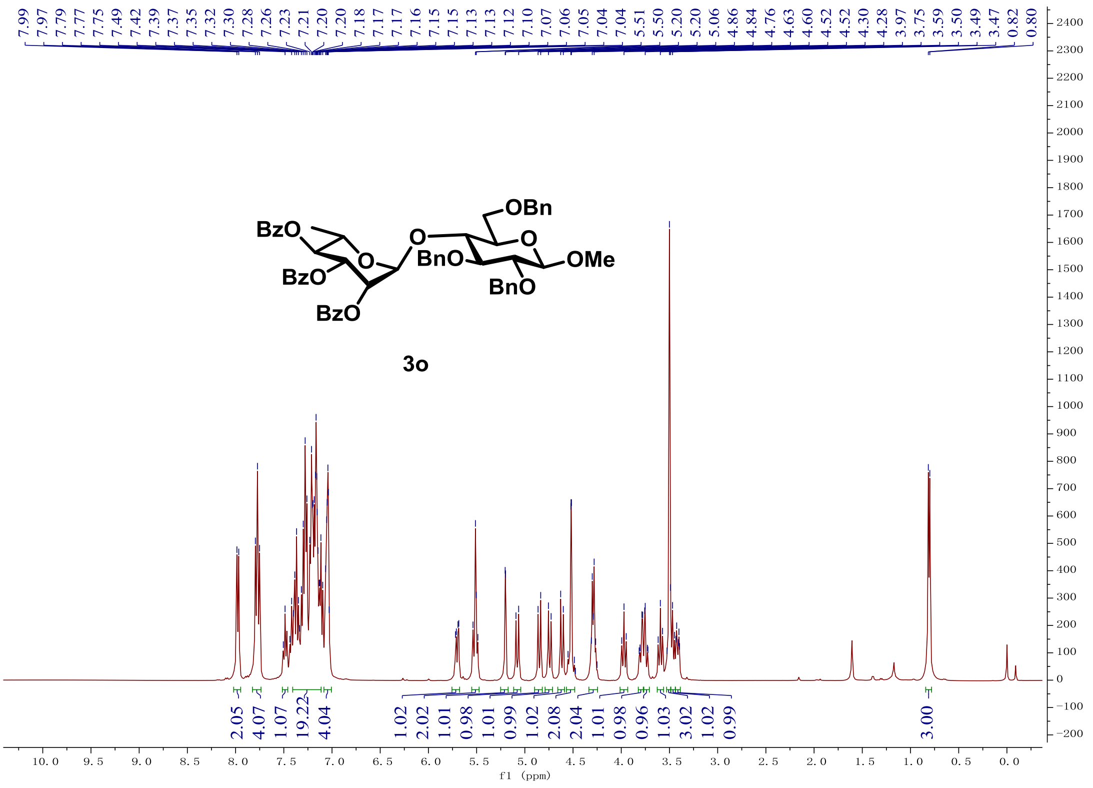

Supplementary Figure 118. ^{1}H NMR Spectrum of Compound **3o**

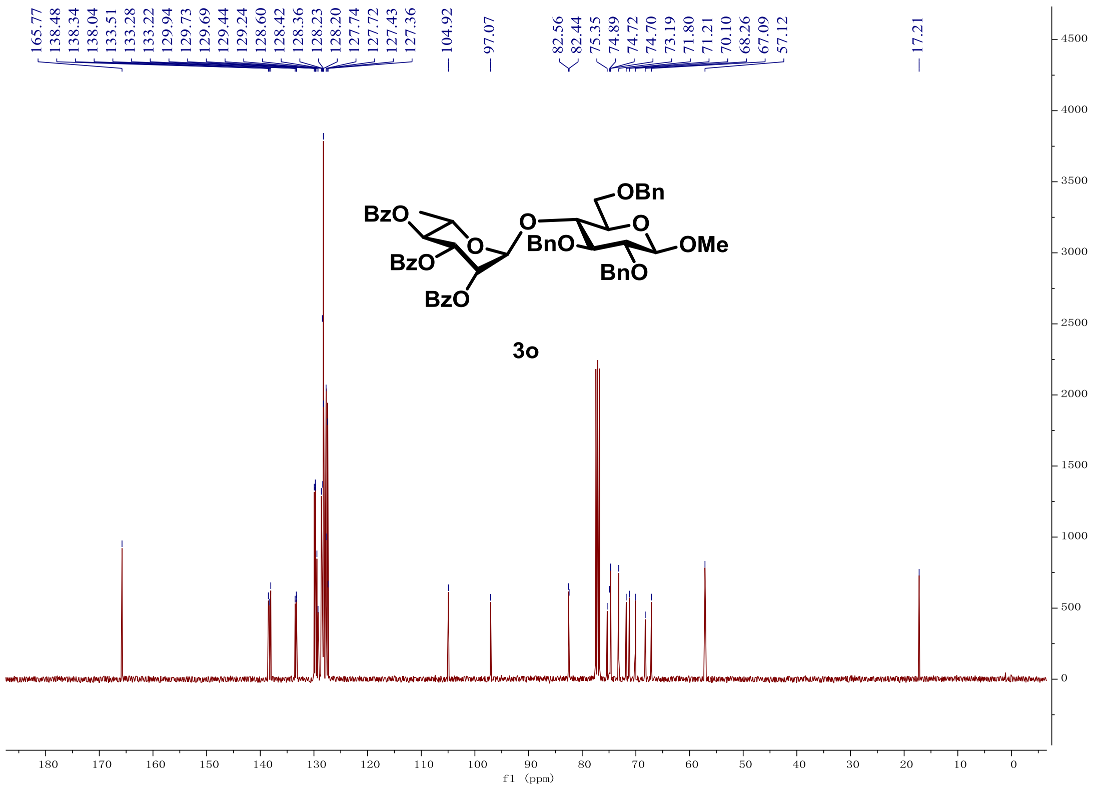

Supplementary Figure 119. ^{13}C NMR Spectrum of Compound **3o**

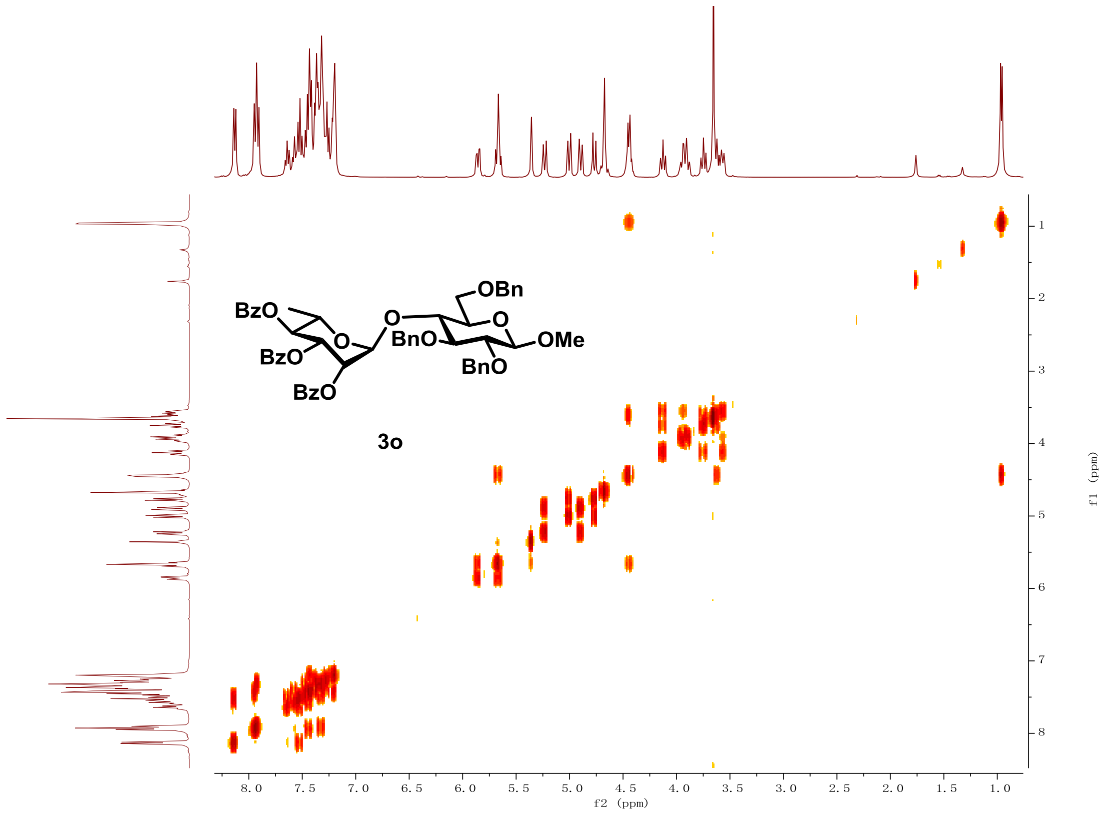

Supplementary Figure 120. COSY NMR Spectrum of Compound **3o**

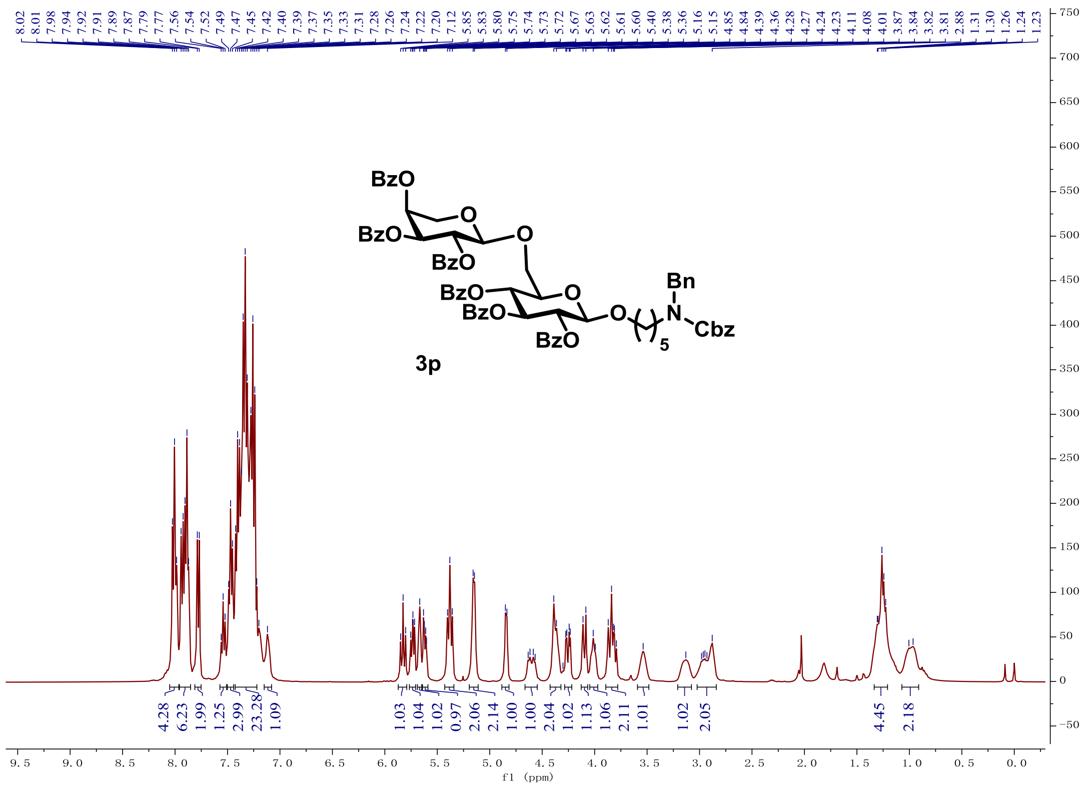

Supplementary Figure 121. ^{1}H NMR Spectrum of Compound **3p**

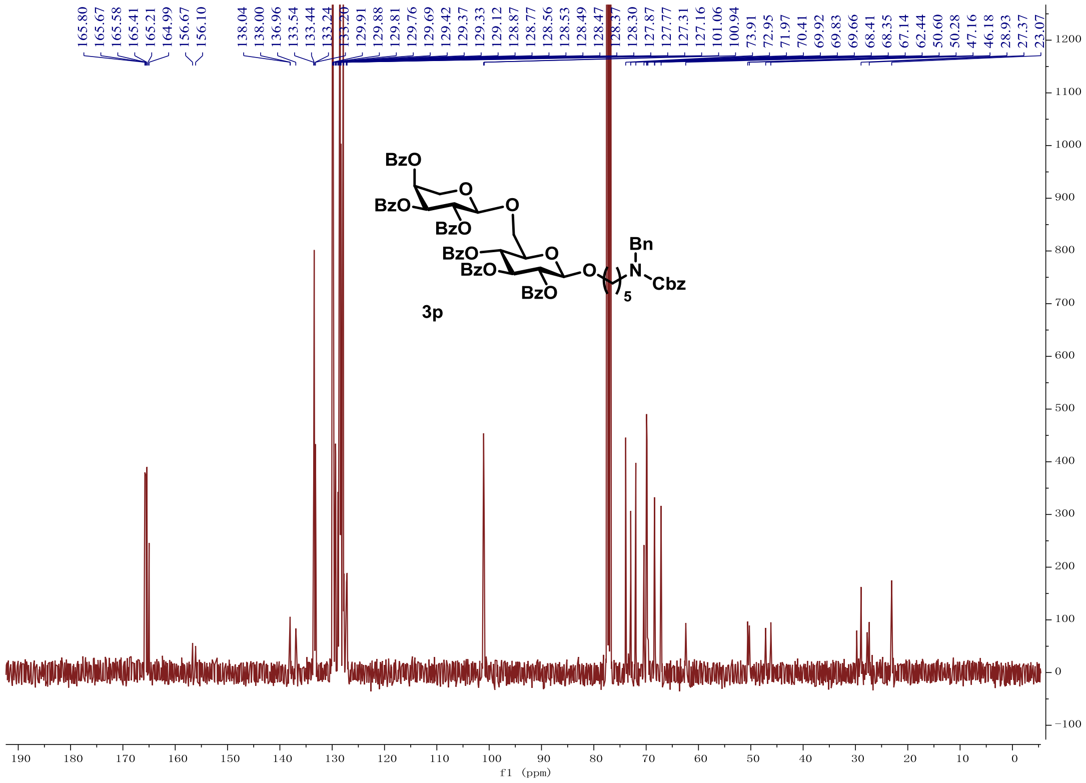

Supplementary Figure 122. ^{13}C NMR Spectrum of Compound **3p**

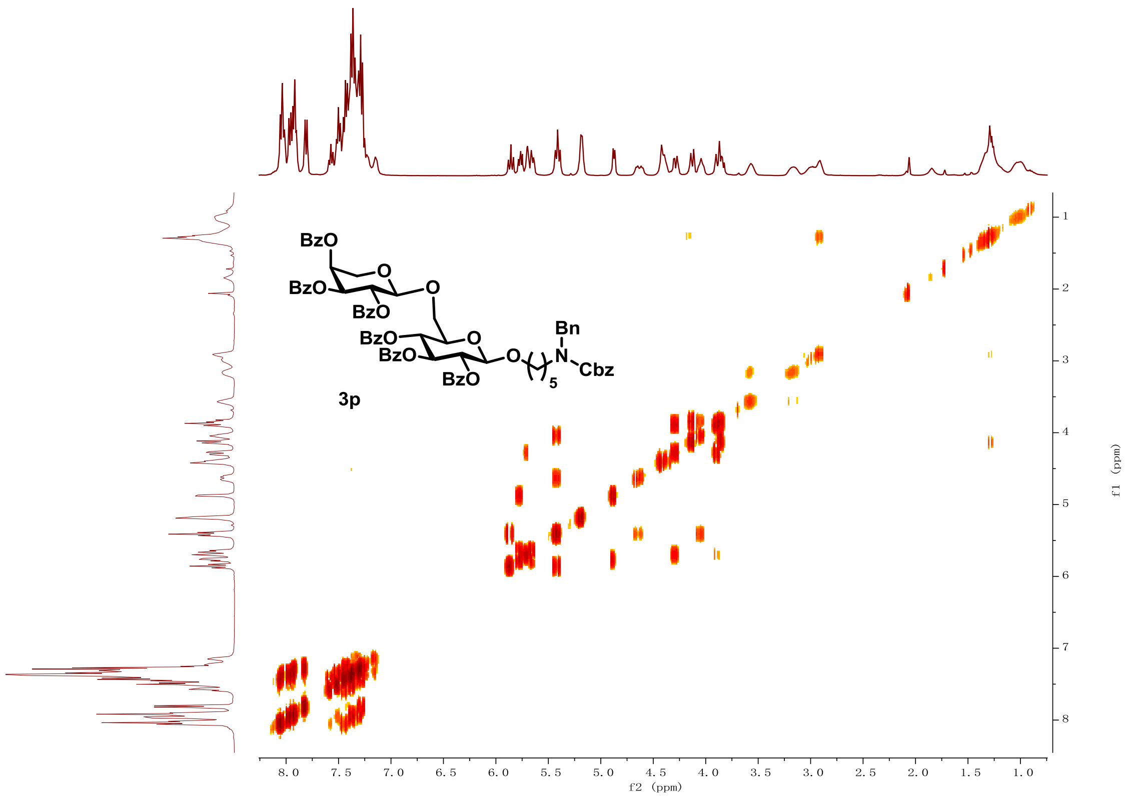

Supplementary Figure 123. COSY NMR Spectrum of Compound **3p**

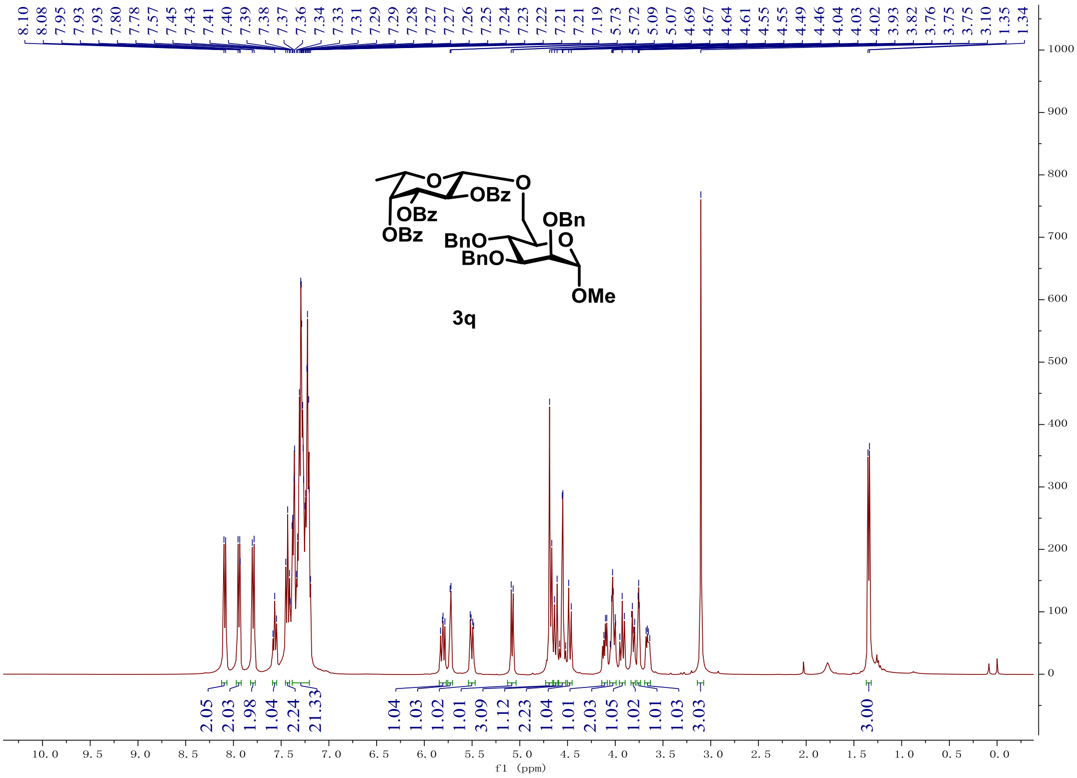

Supplementary Figure 124. ^{1}H NMR Spectrum of Compound **3q**

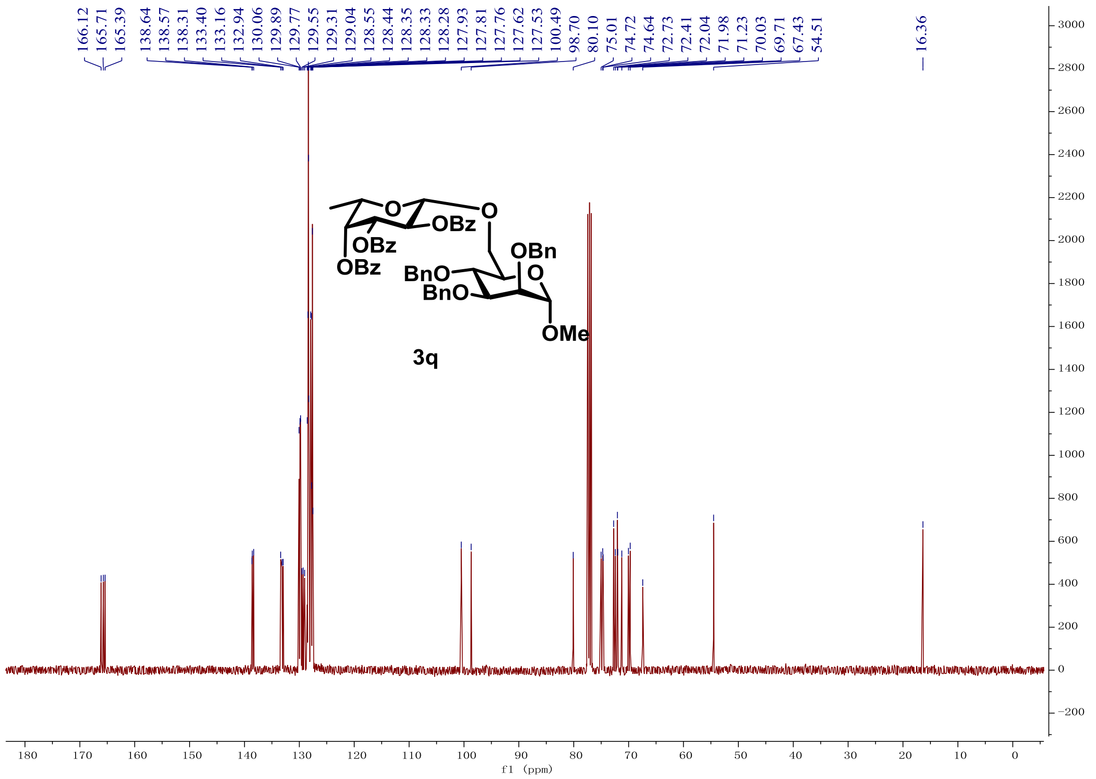

Supplementary Figure 125. ^{13}C NMR Spectrum of Compound **3q**

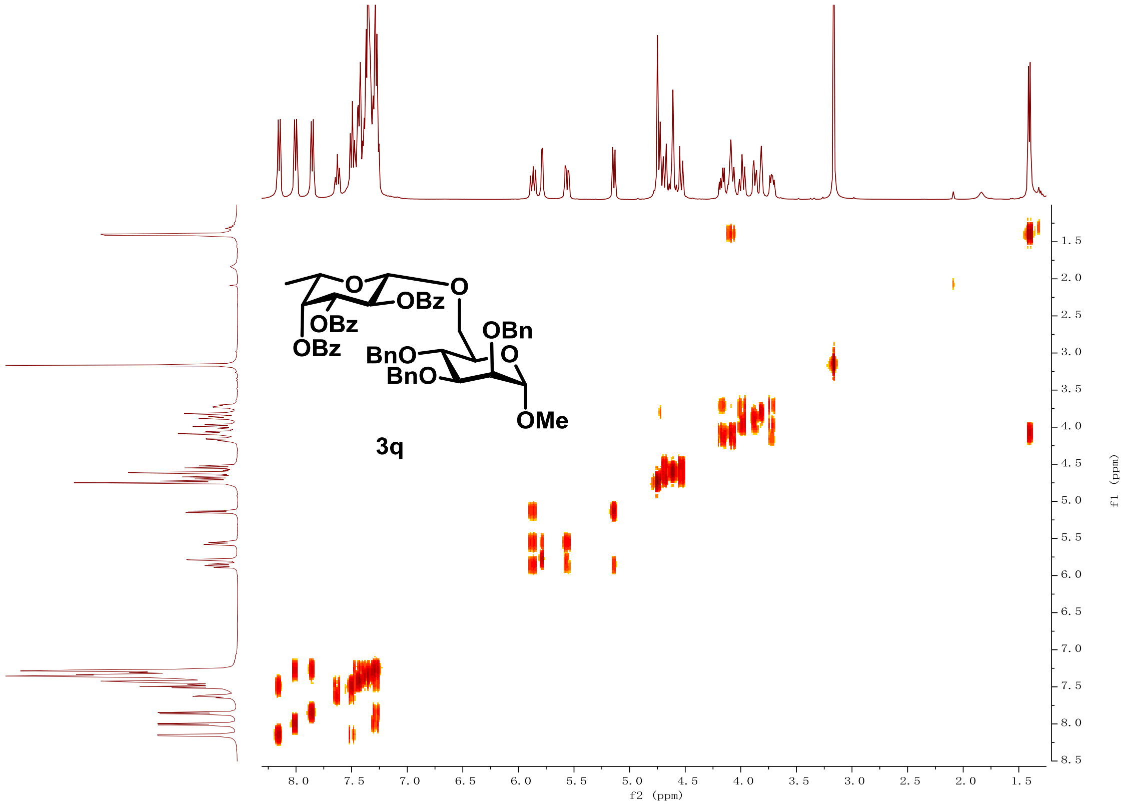

Supplementary Figure 126. COSY NMR Spectrum of Compound **3q**

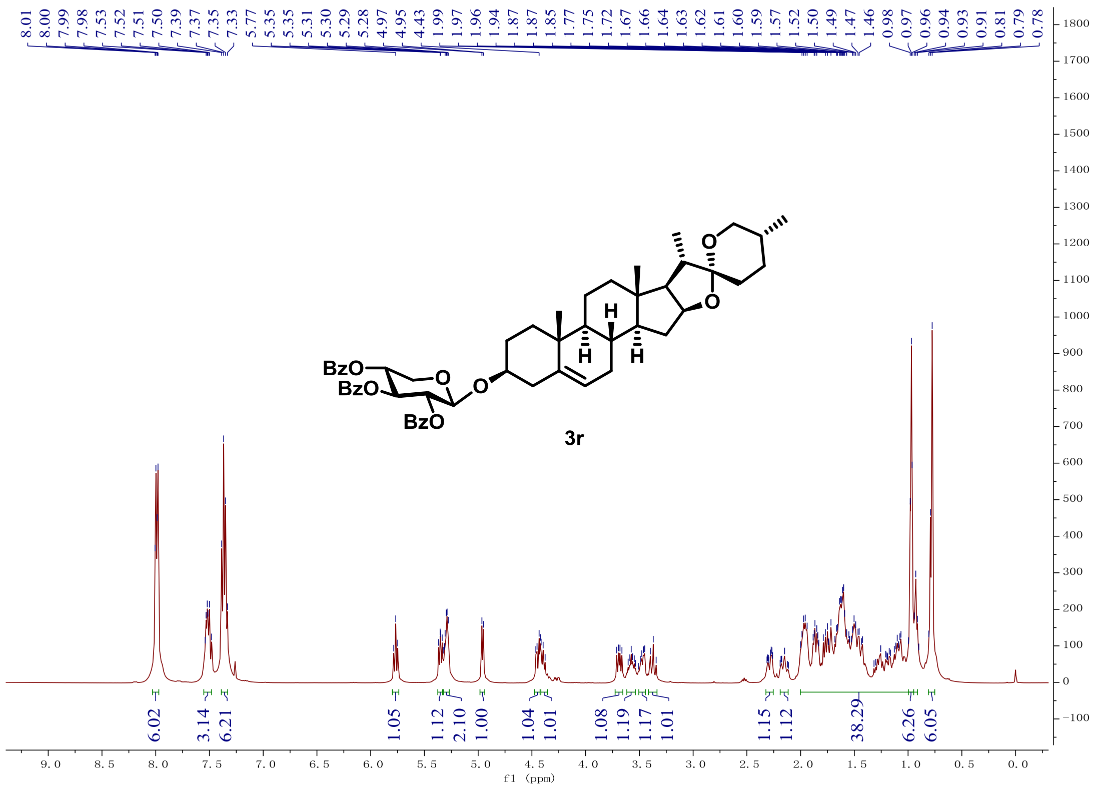

Supplementary Figure 127. ^{1}H NMR Spectrum of Compound **3r**

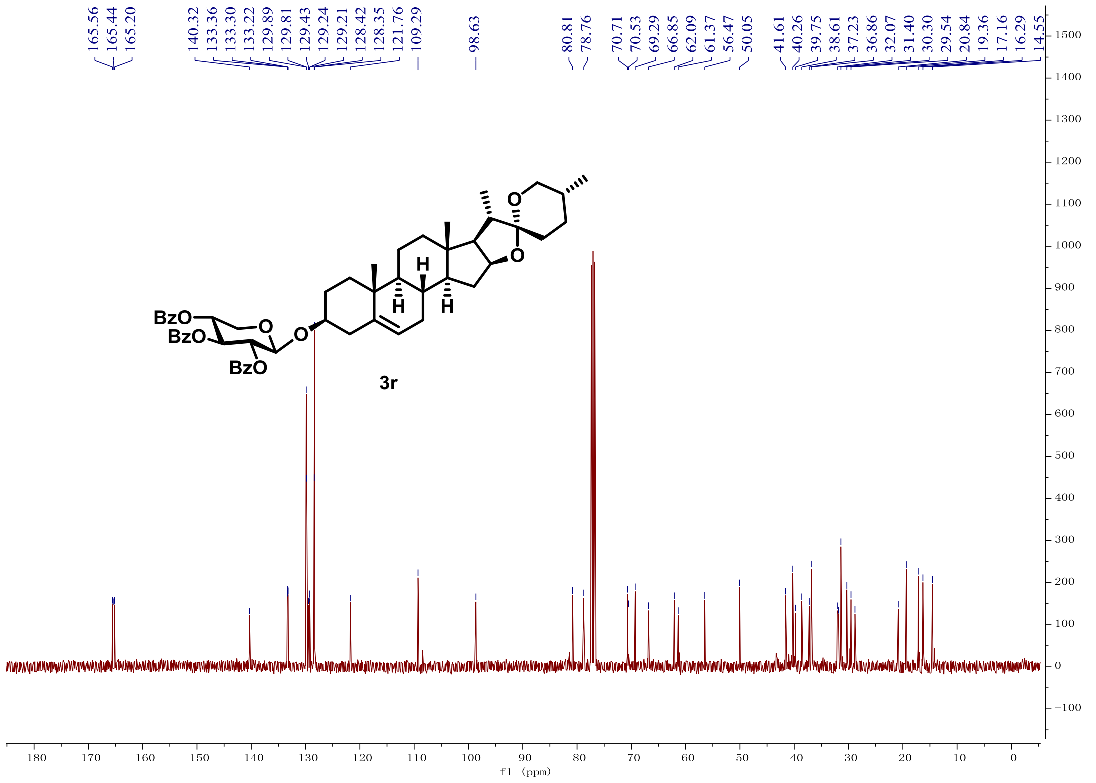

Supplementary Figure 128. ^{13}C NMR Spectrum of Compound **3r**

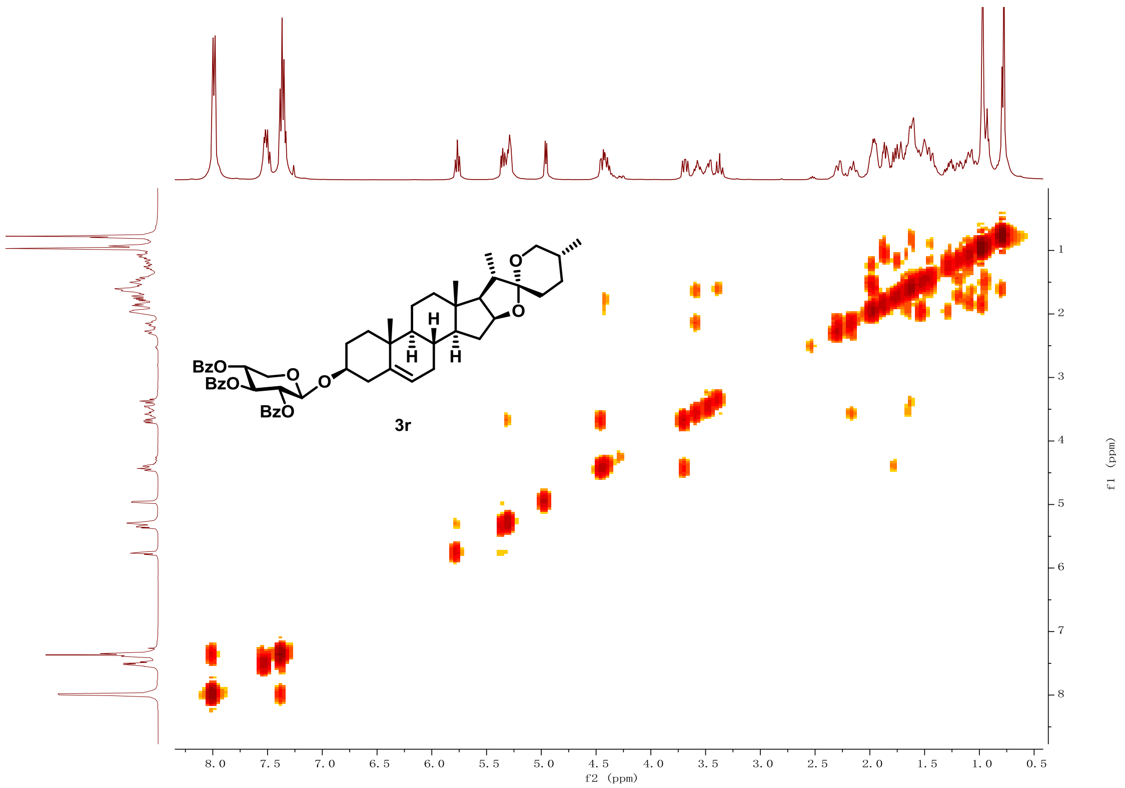

Supplementary Figure 129. COSY NMR Spectrum of Compound **3r**

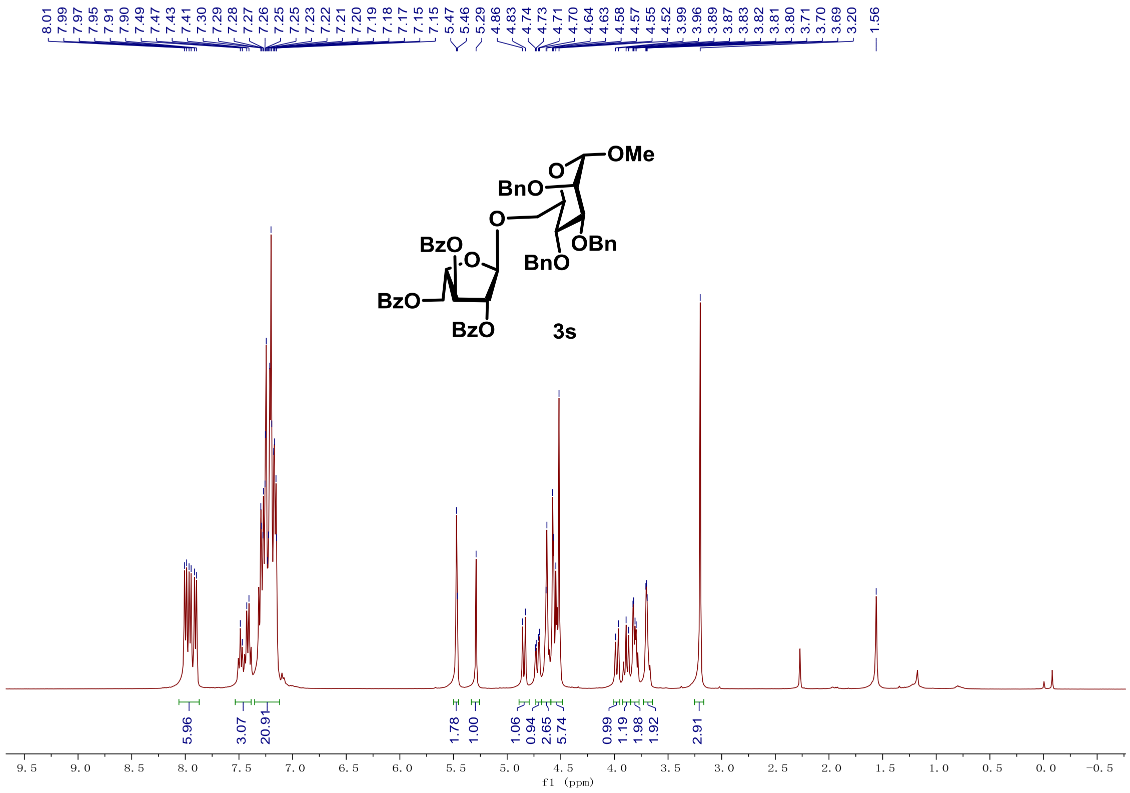

Supplementary Figure 130. ^{1}H NMR Spectrum of Compound **3s**

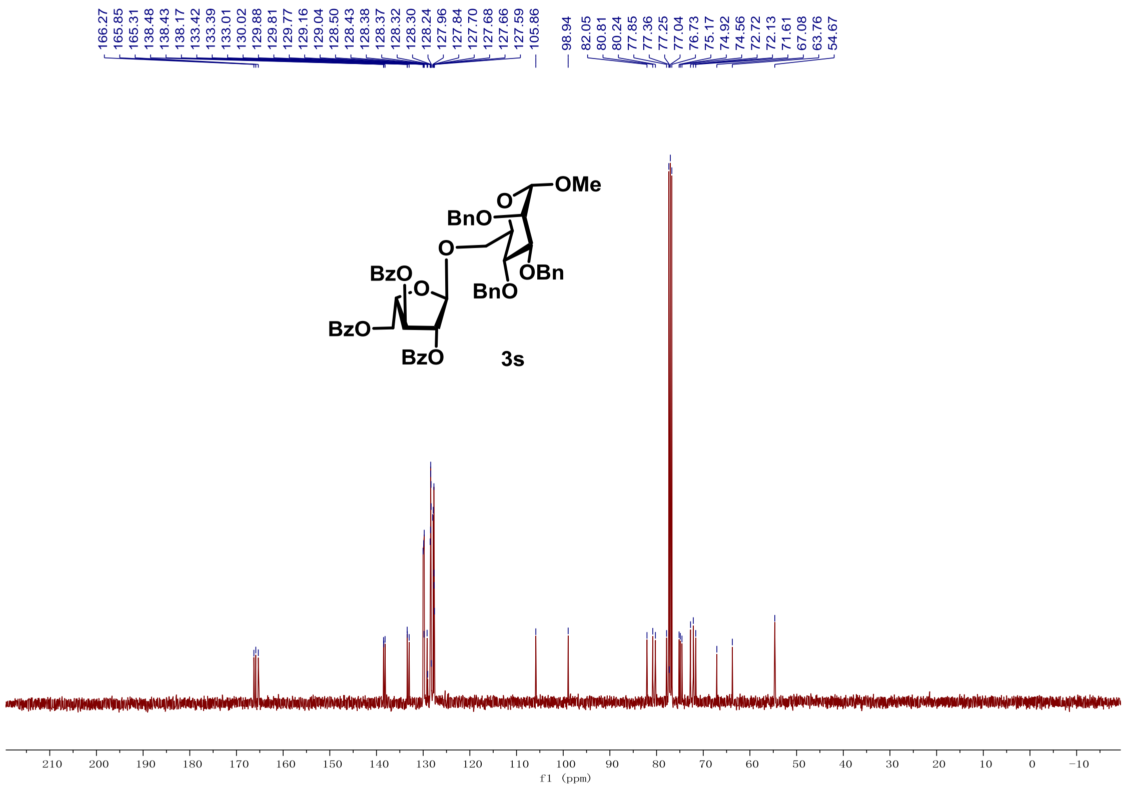

Supplementary Figure 131. ^{13}C NMR Spectrum of Compound **3s**

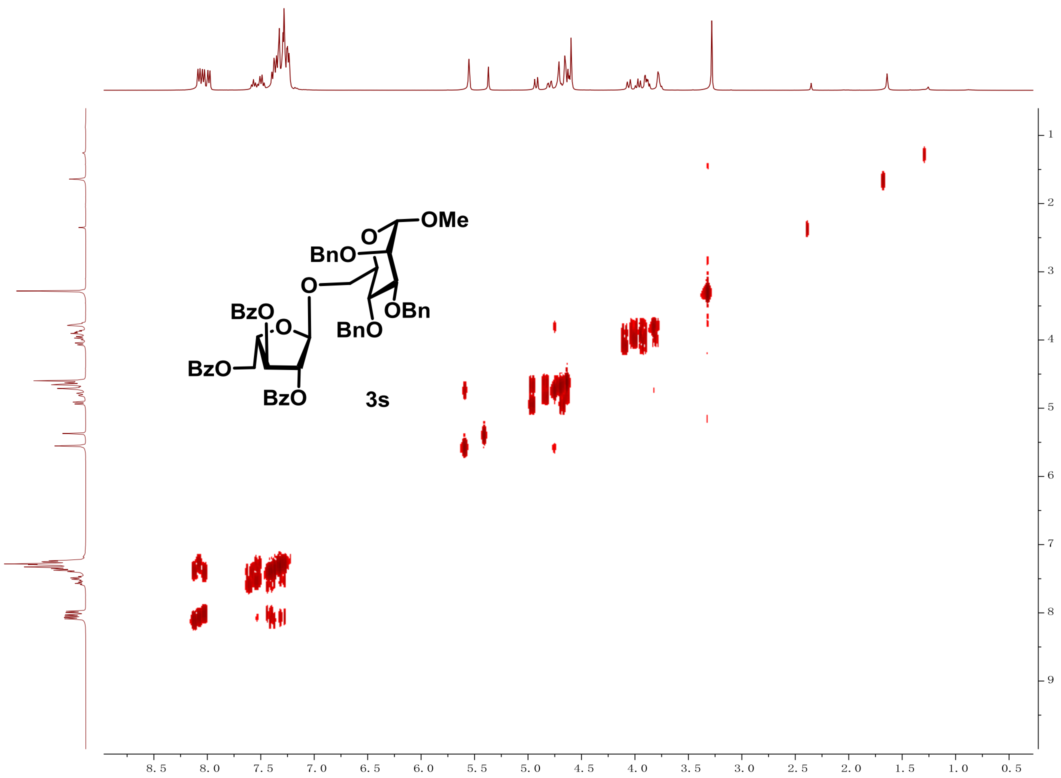

Supplementary Figure 132. COSY NMR Spectrum of Compound **3s**

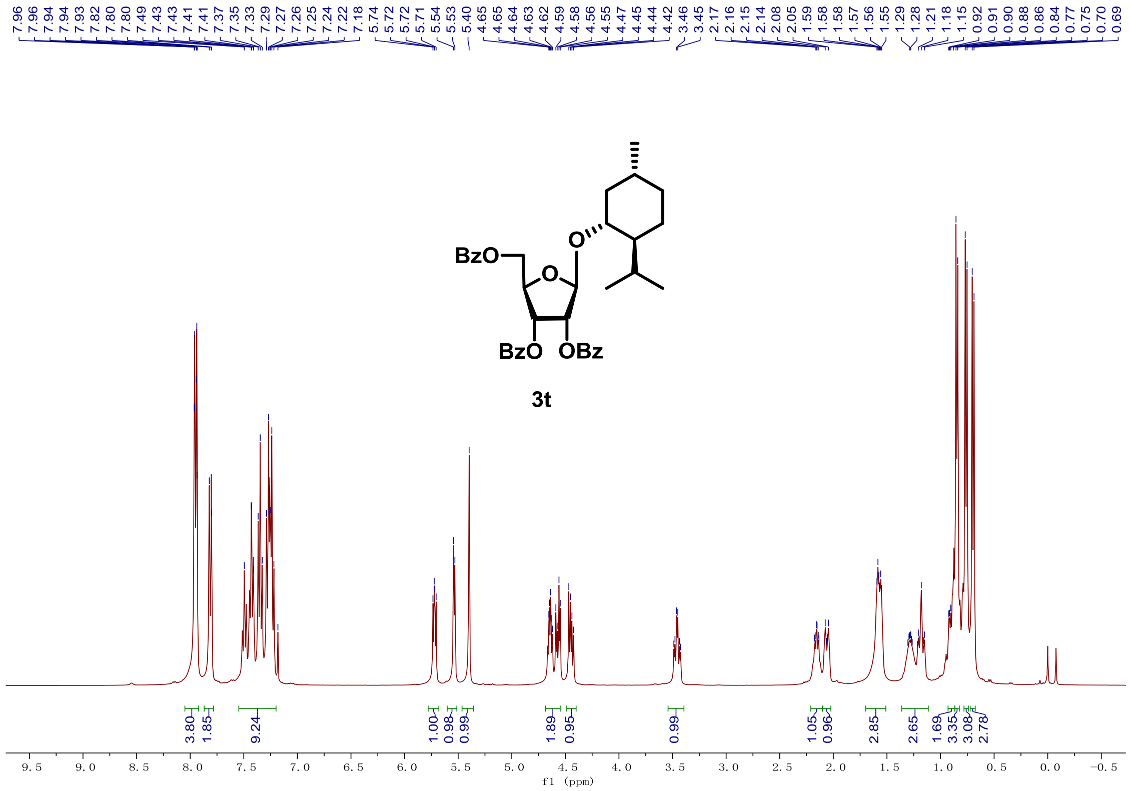

Supplementary Figure 133. ^{1}H NMR Spectrum of Compound **3t**

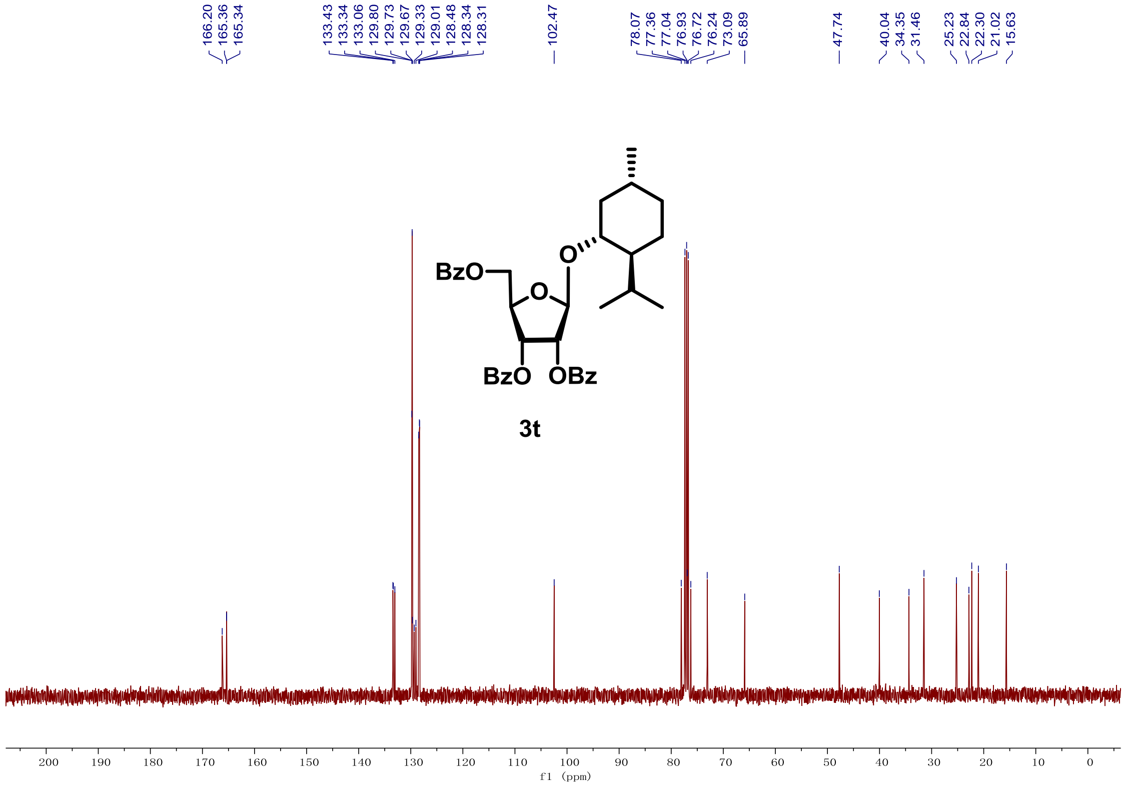

Supplementary Figure 134. ^{13}C NMR Spectrum of Compound **3t**

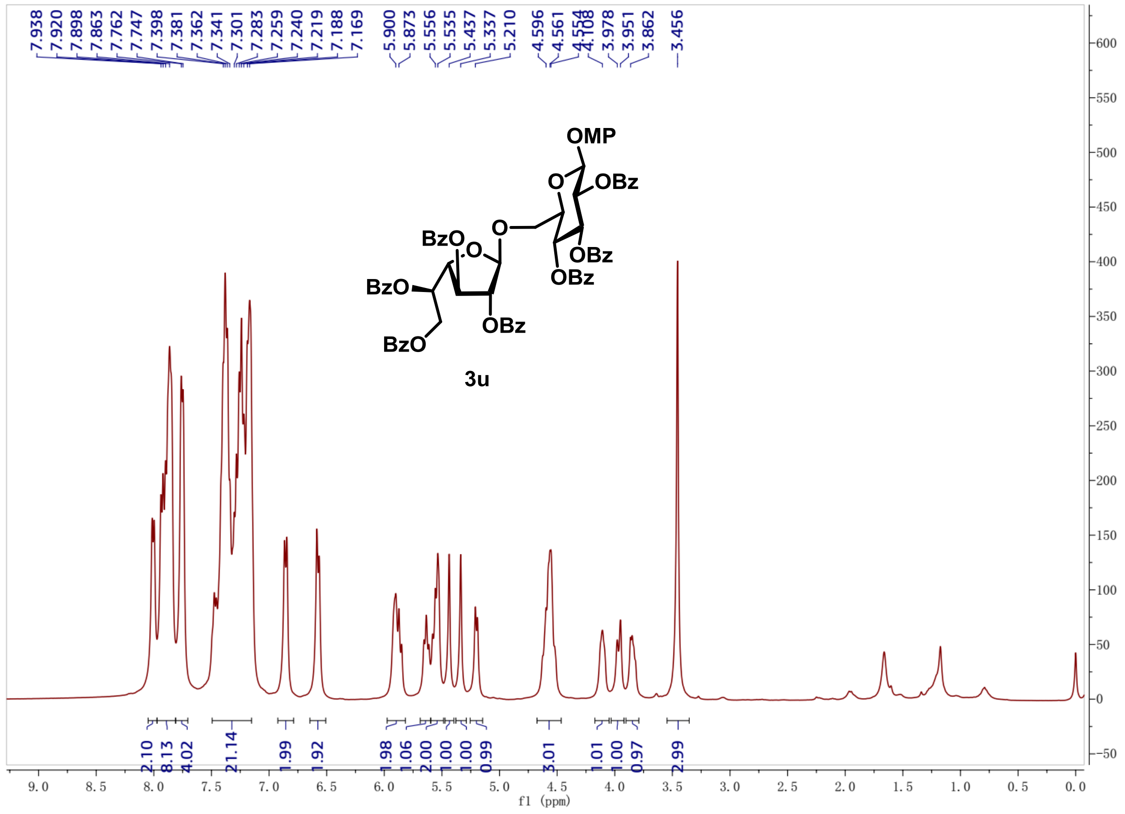

Supplementary Figure 135. ^{1}H NMR Spectrum of Compound **3u**

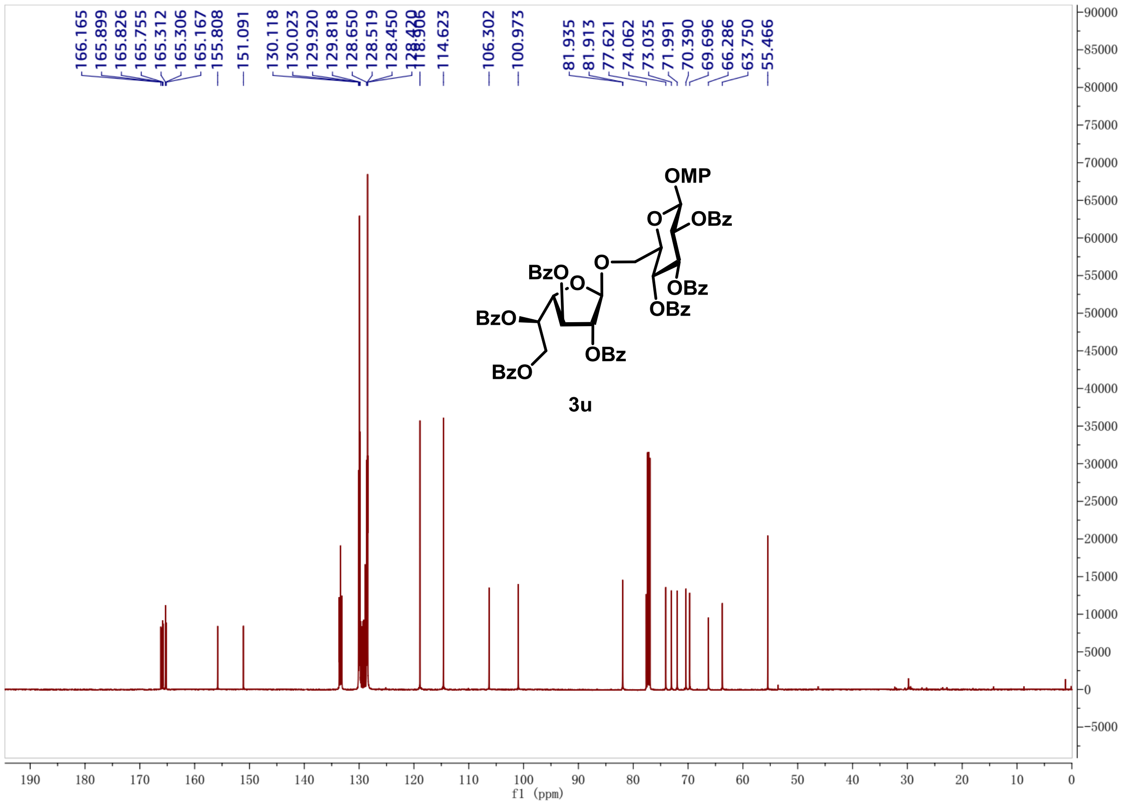

Supplementary Figure 136. ^{13}C NMR Spectrum of Compound **3u**

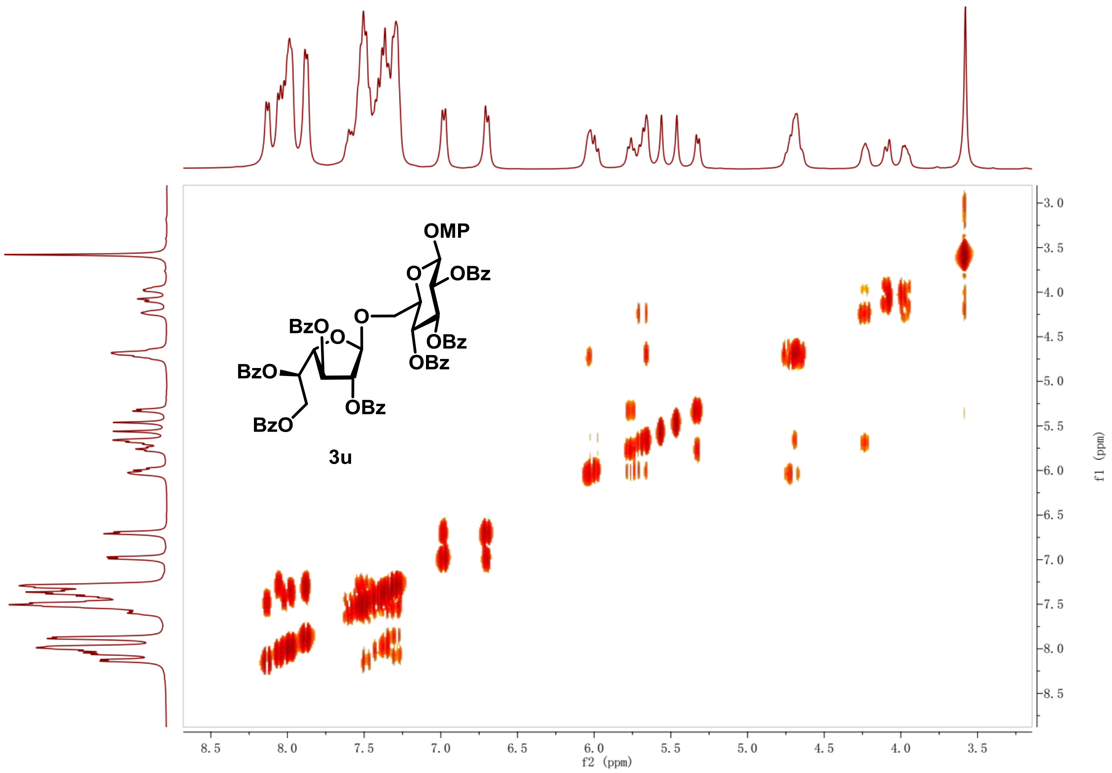

Supplementary Figure 137. COSY NMR Spectrum of Compound **3u**

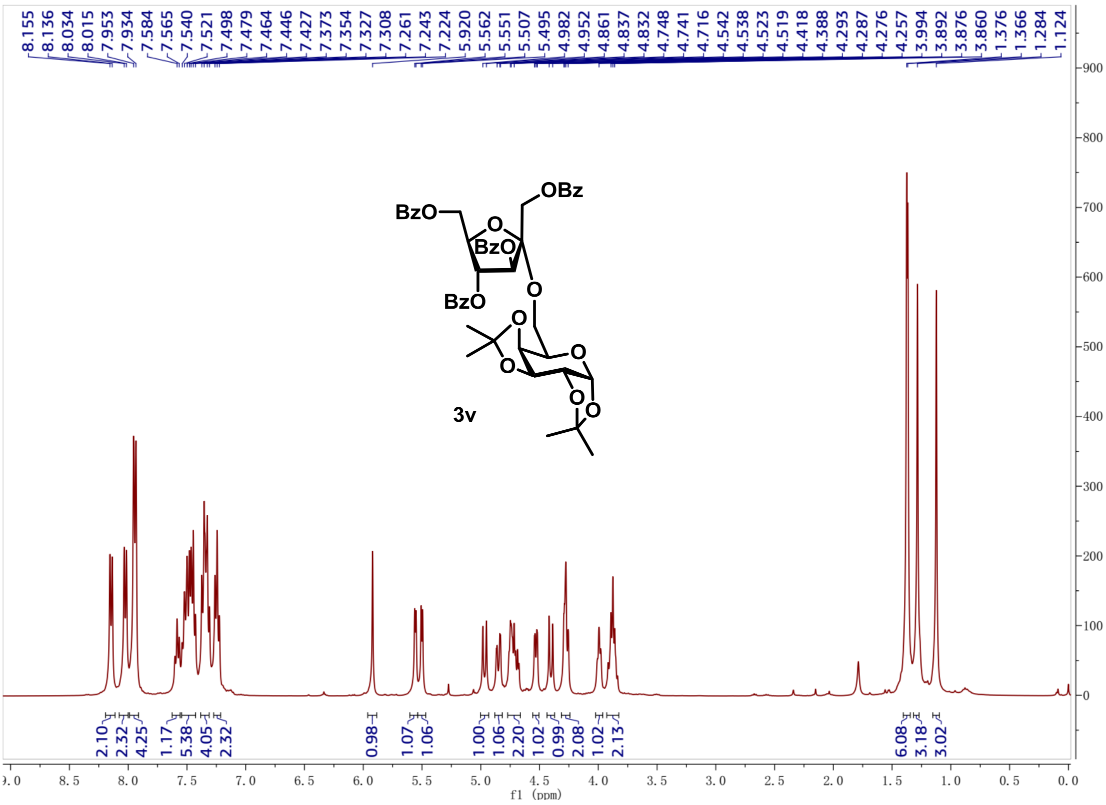

Supplementary Figure 138. ^{1}H NMR Spectrum of Compound **3v**

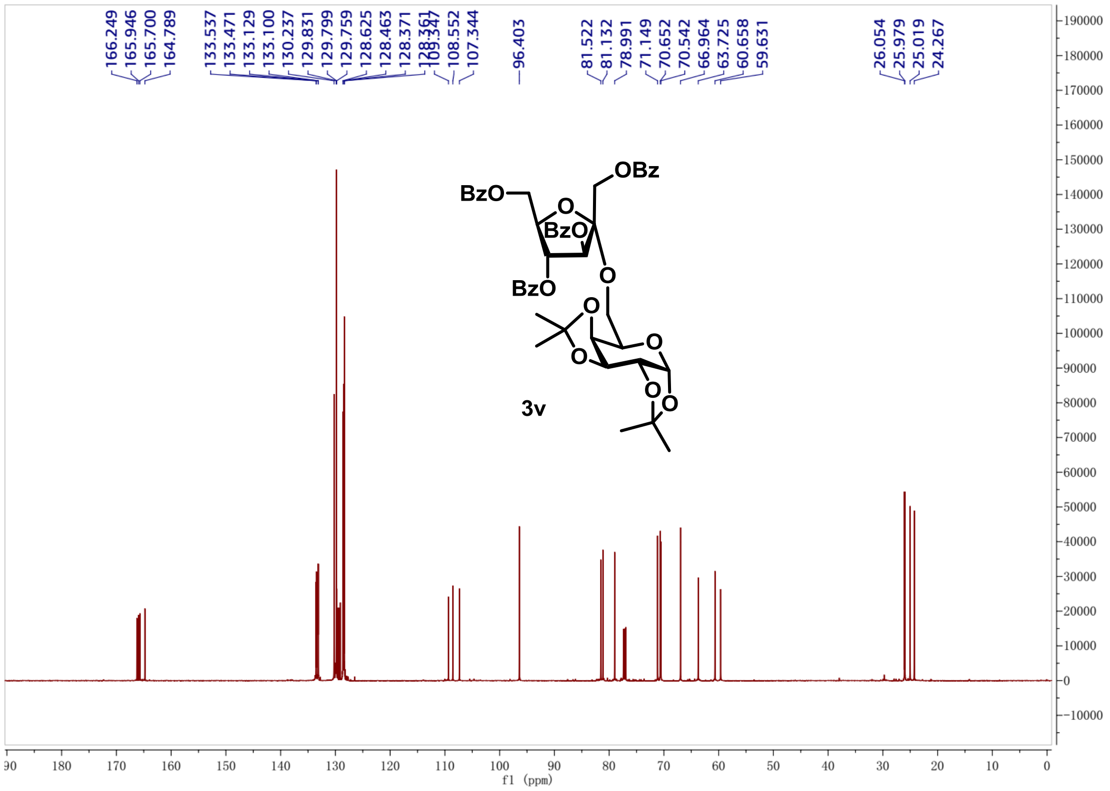

Supplementary Figure 139. ^{13}C NMR Spectrum of Compound **3v**

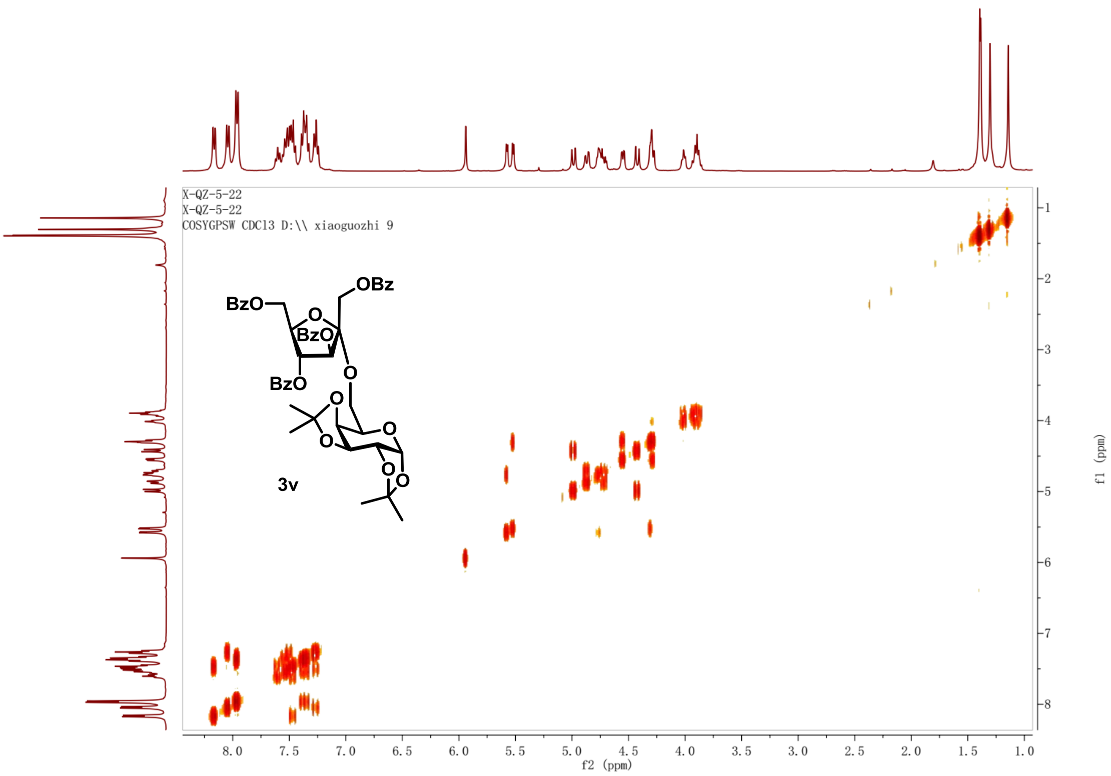

Supplementary Figure 140. COSY NMR Spectrum of Compound **3v**

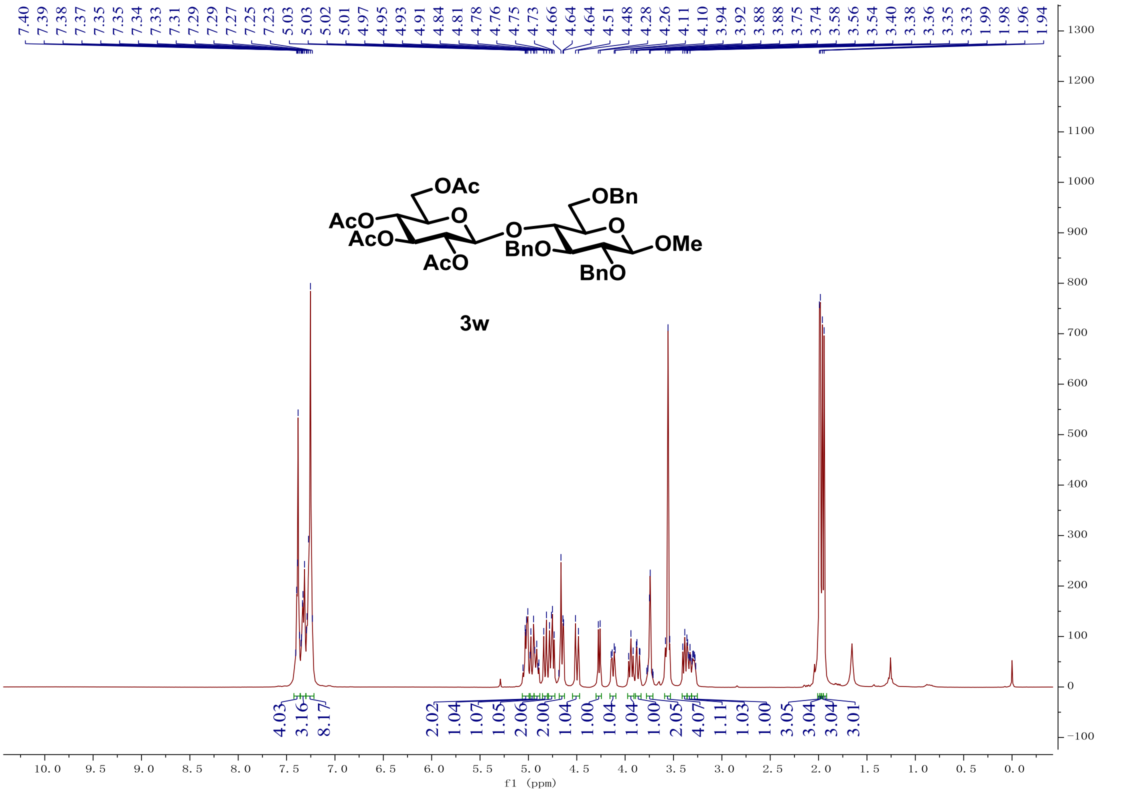

Supplementary Figure 141. ^{1}H NMR Spectrum of Compound **3w**

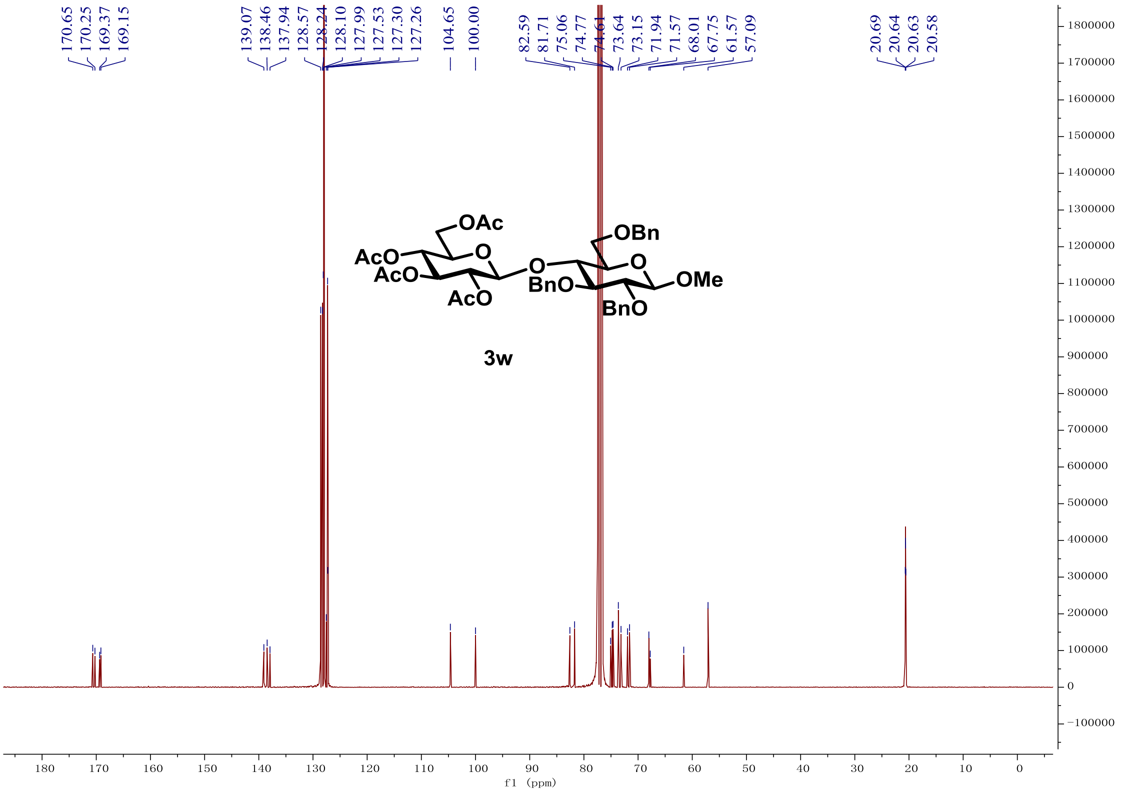

Supplementary Figure 142. ^{13}C NMR Spectrum of Compound **3w**

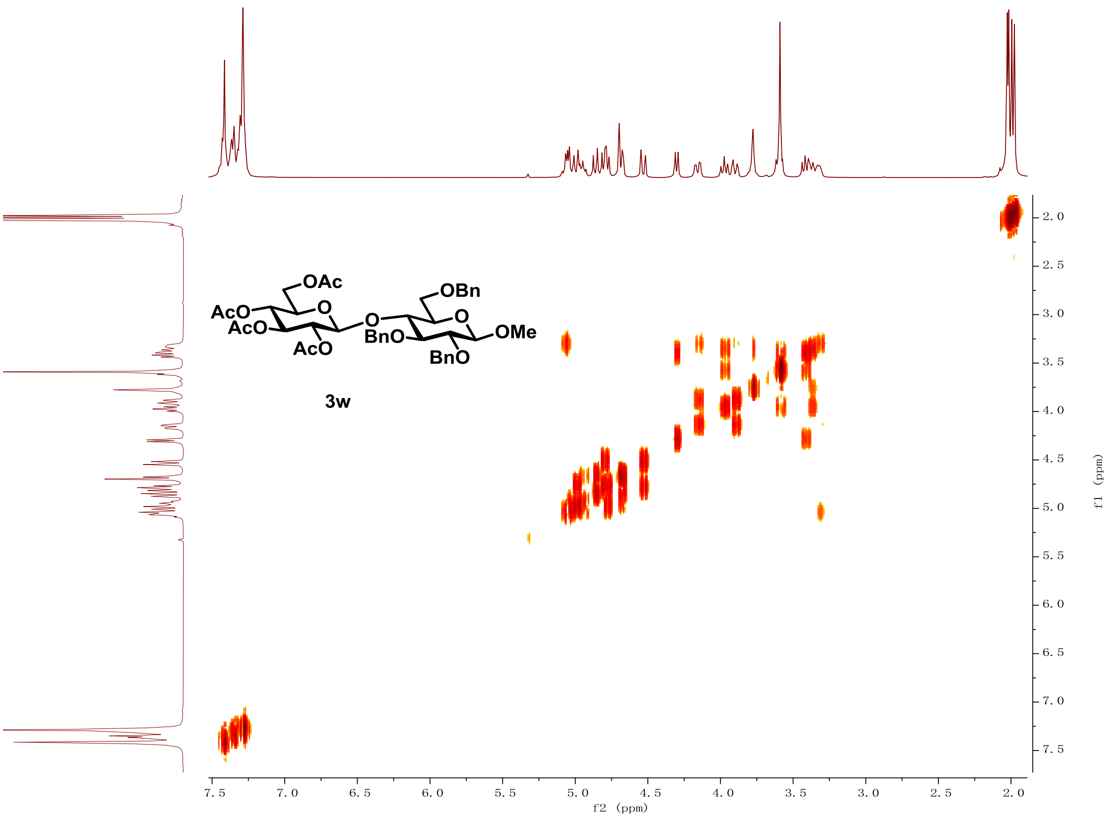

Supplementary Figure 143. COSY NMR Spectrum of Compound **3w**

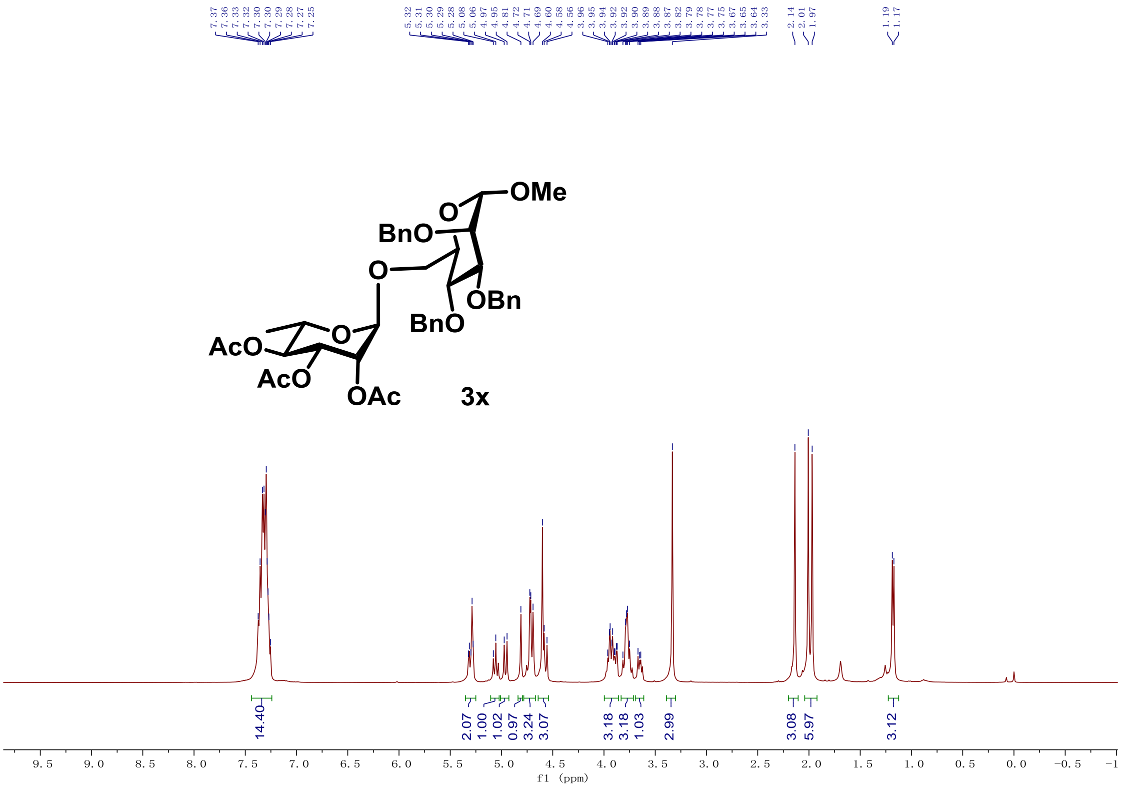

Supplementary Figure 144. ^{1}H NMR Spectrum of Compound **3x**

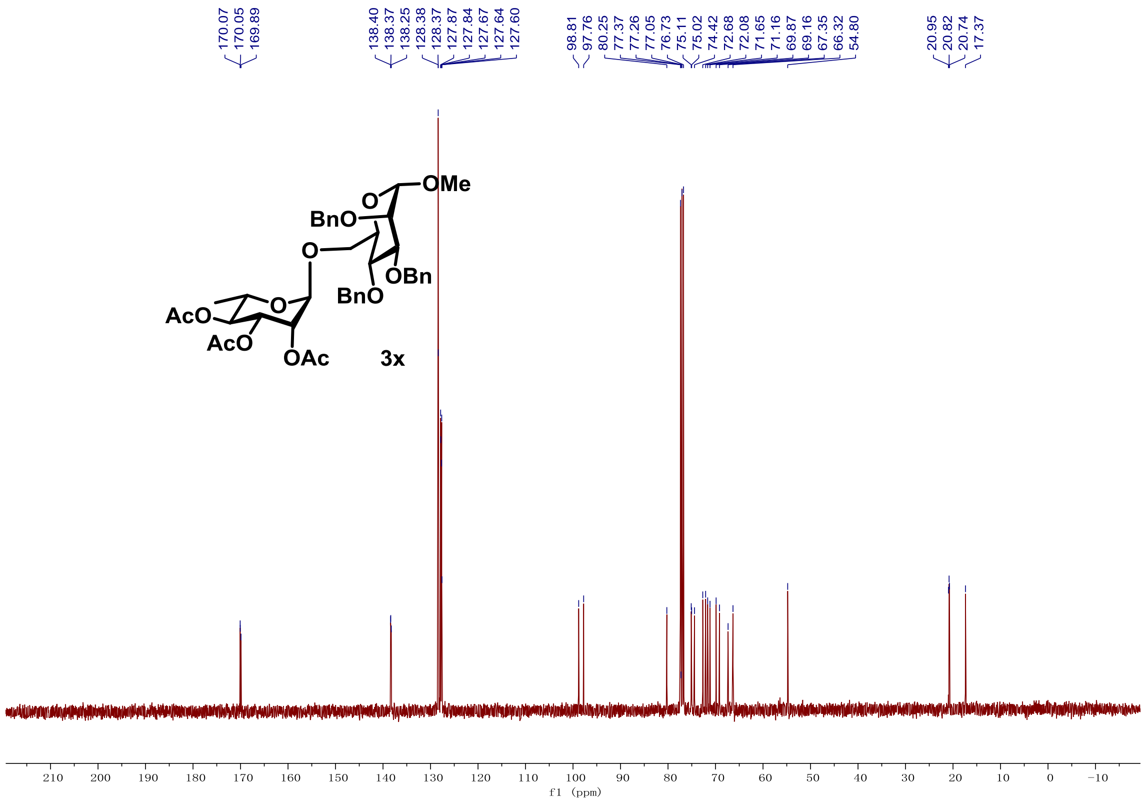

Supplementary Figure 145. ^{13}C NMR Spectrum of Compound **3x**

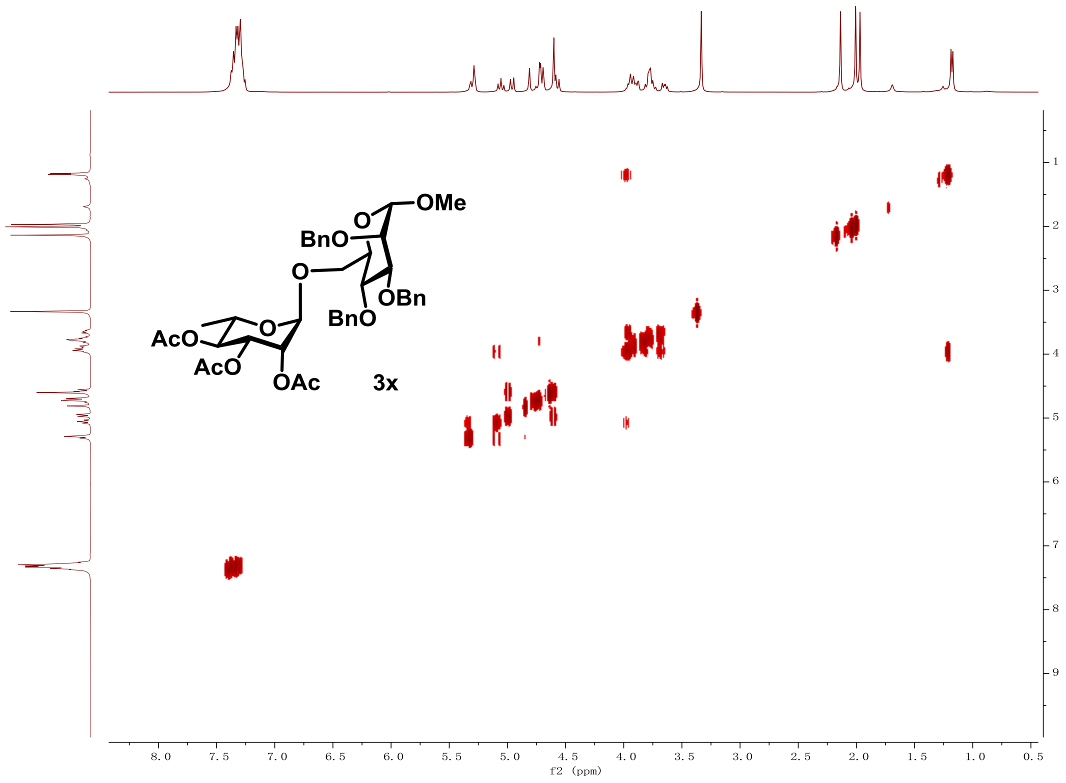

Supplementary Figure 146. COSY NMR Spectrum of Compound **3x**

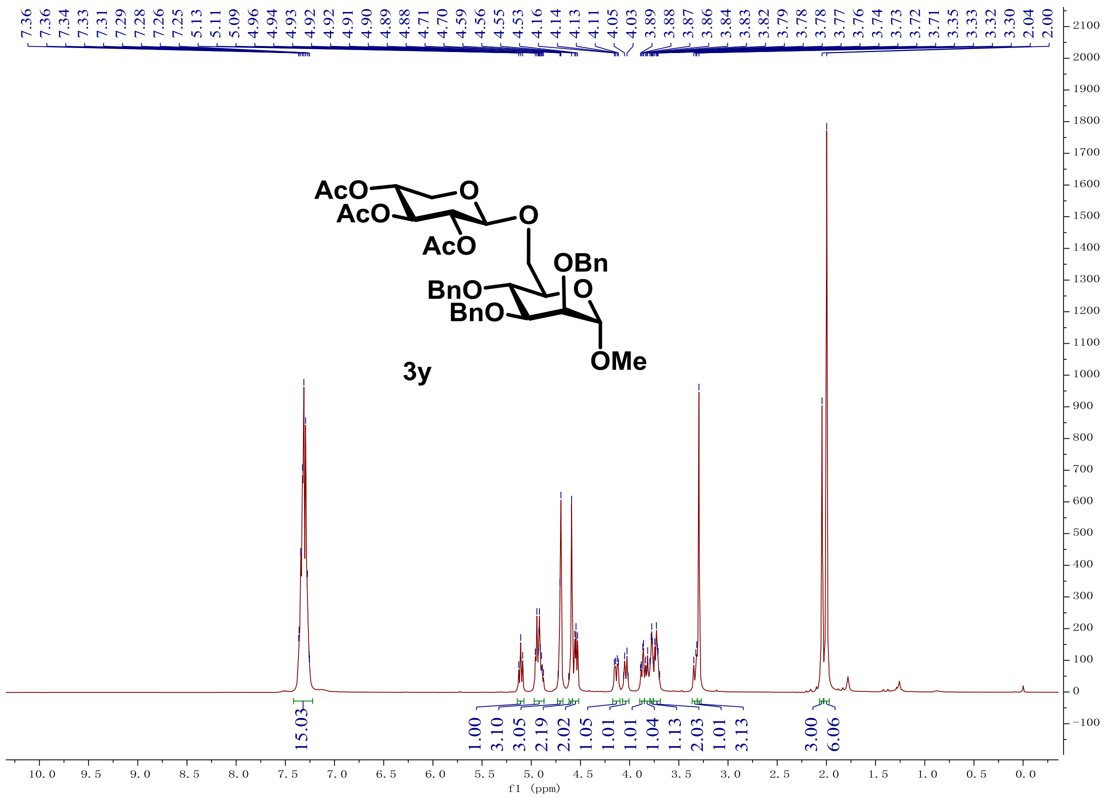

Supplementary Figure 147. ^{1}H NMR Spectrum of Compound **3y**

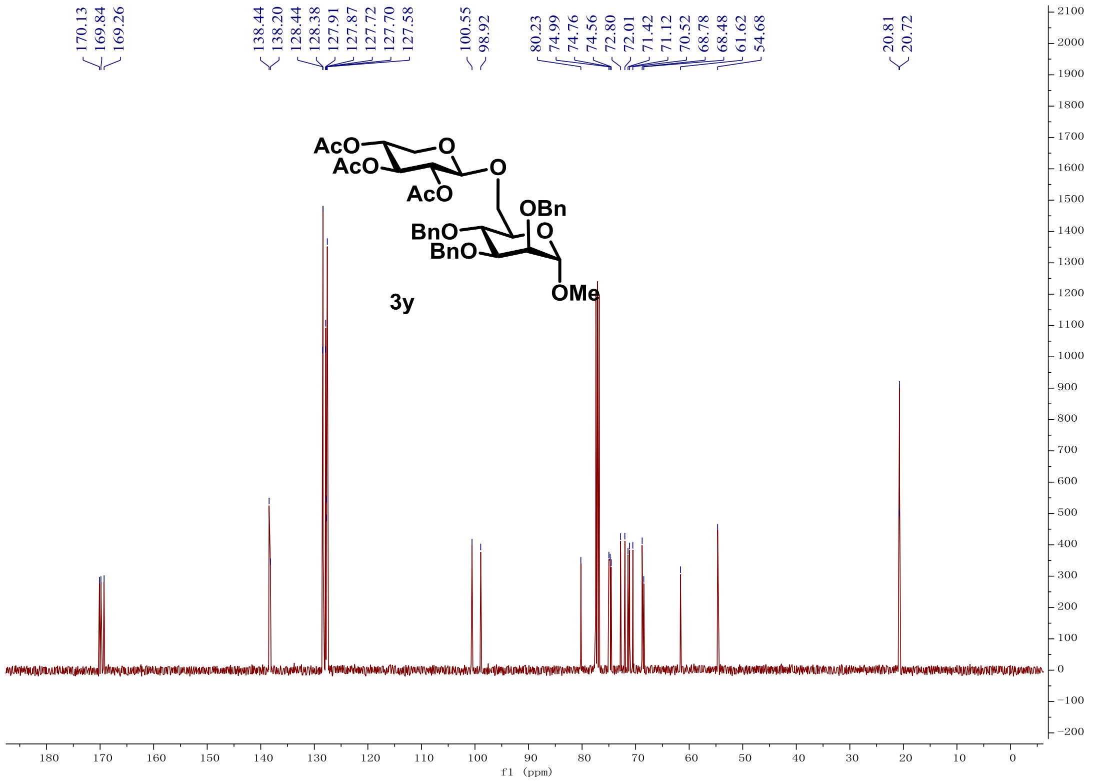

Supplementary Figure 148. ^{13}C NMR Spectrum of Compound **3y**

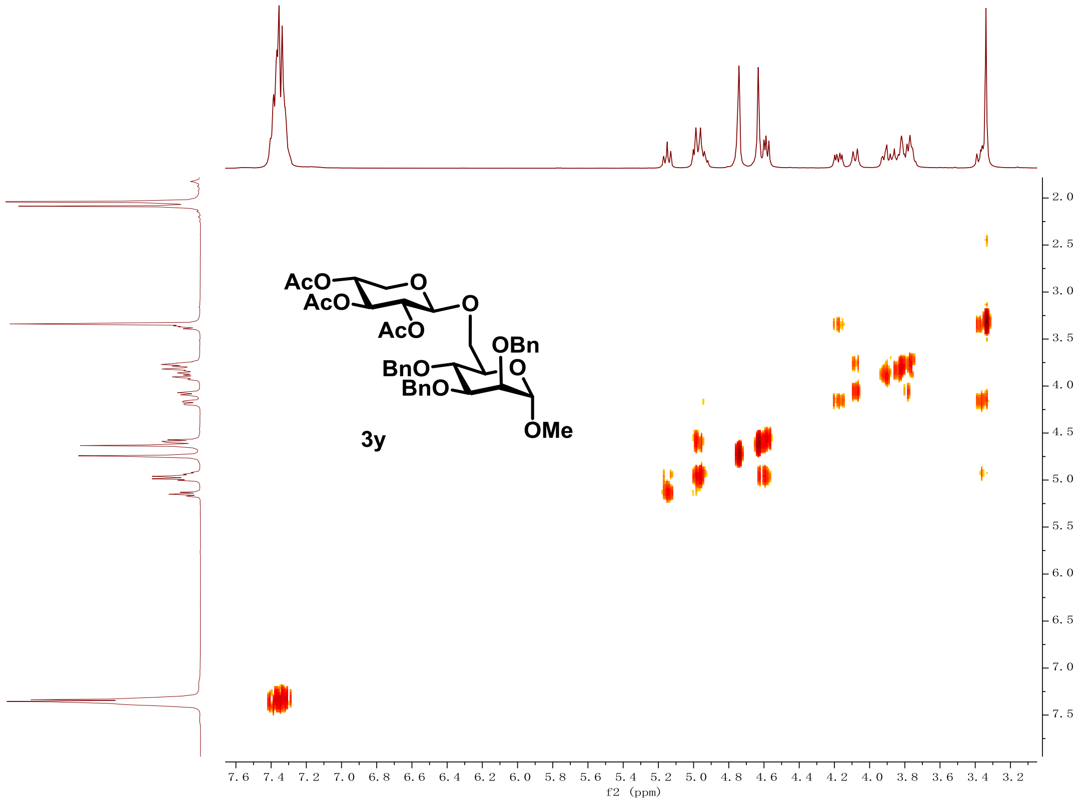

Supplementary Figure 149. COSY NMR Spectrum of Compound **3y**

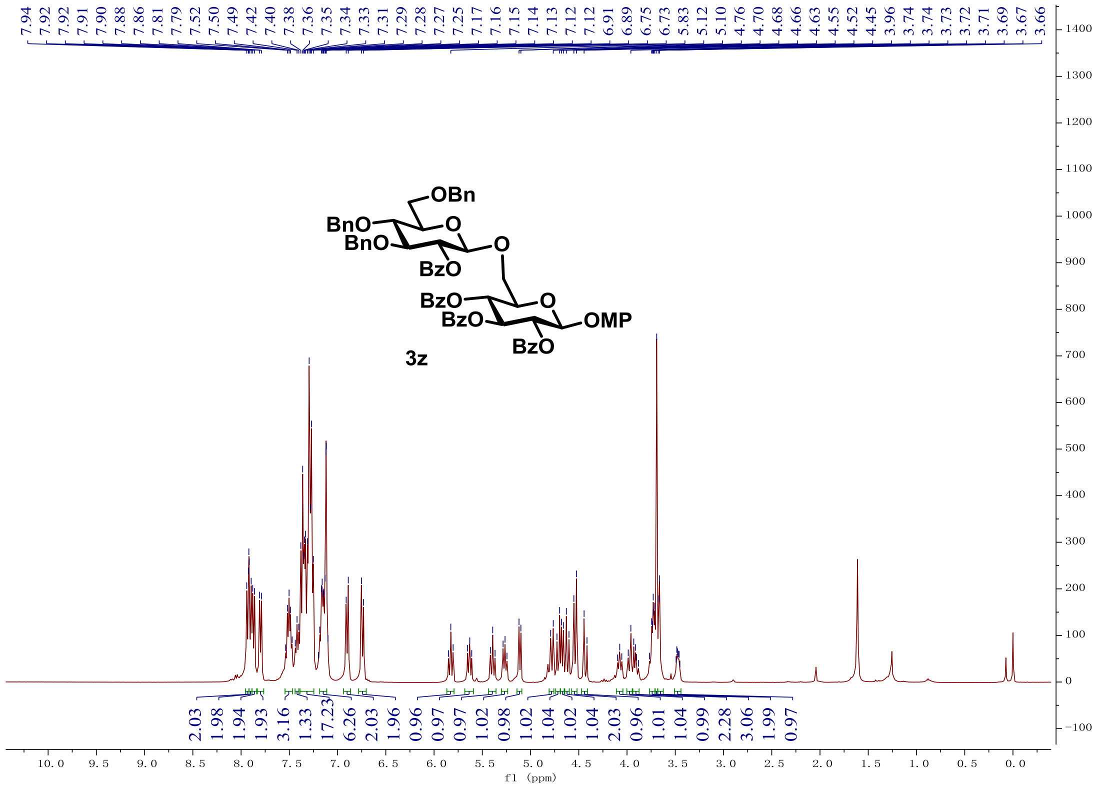

Supplementary Figure 150. ^{1}H NMR Spectrum of Compound **3z**

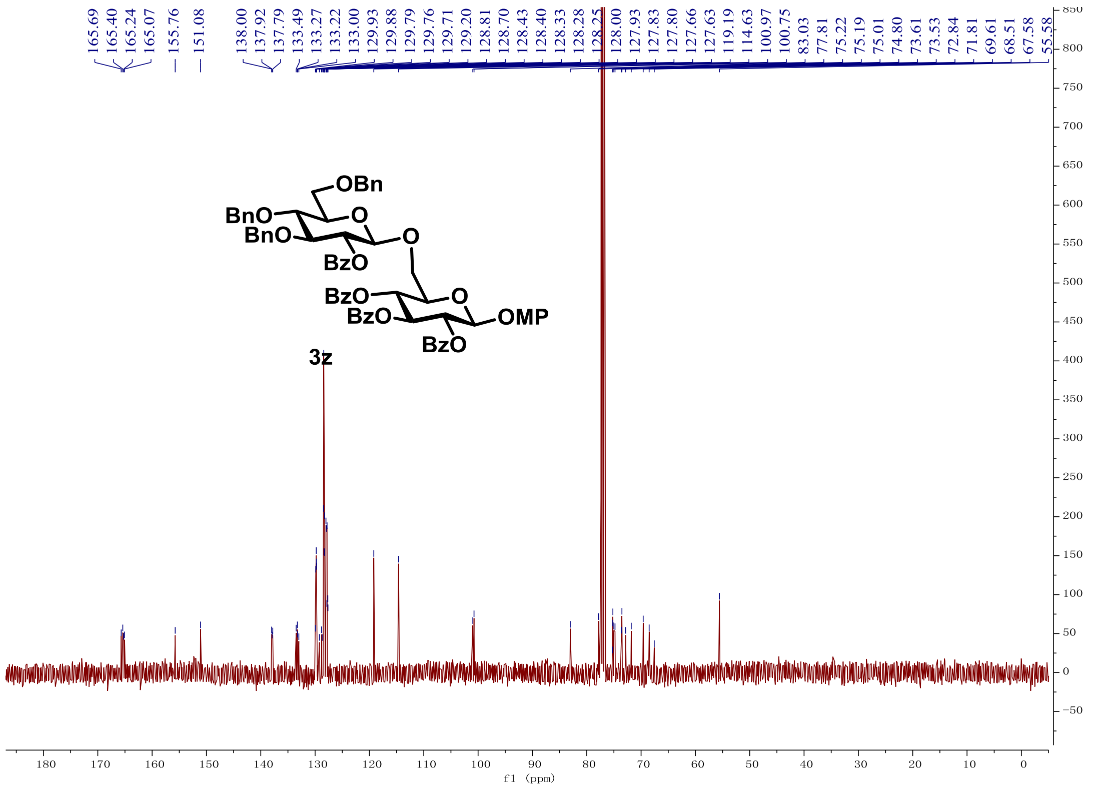

Supplementary Figure 151. ^{13}C NMR Spectrum of Compound **3z**

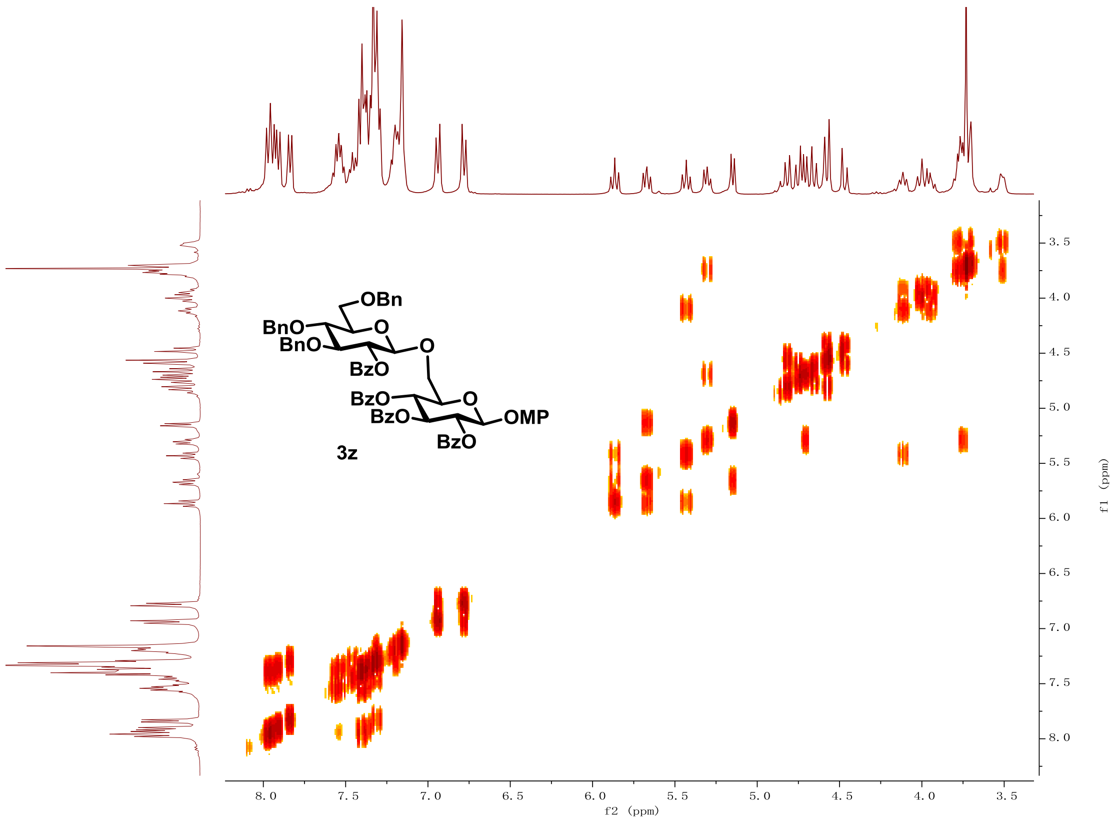

Supplementary Figure 152. COSY NMR Spectrum of Compound **3z**

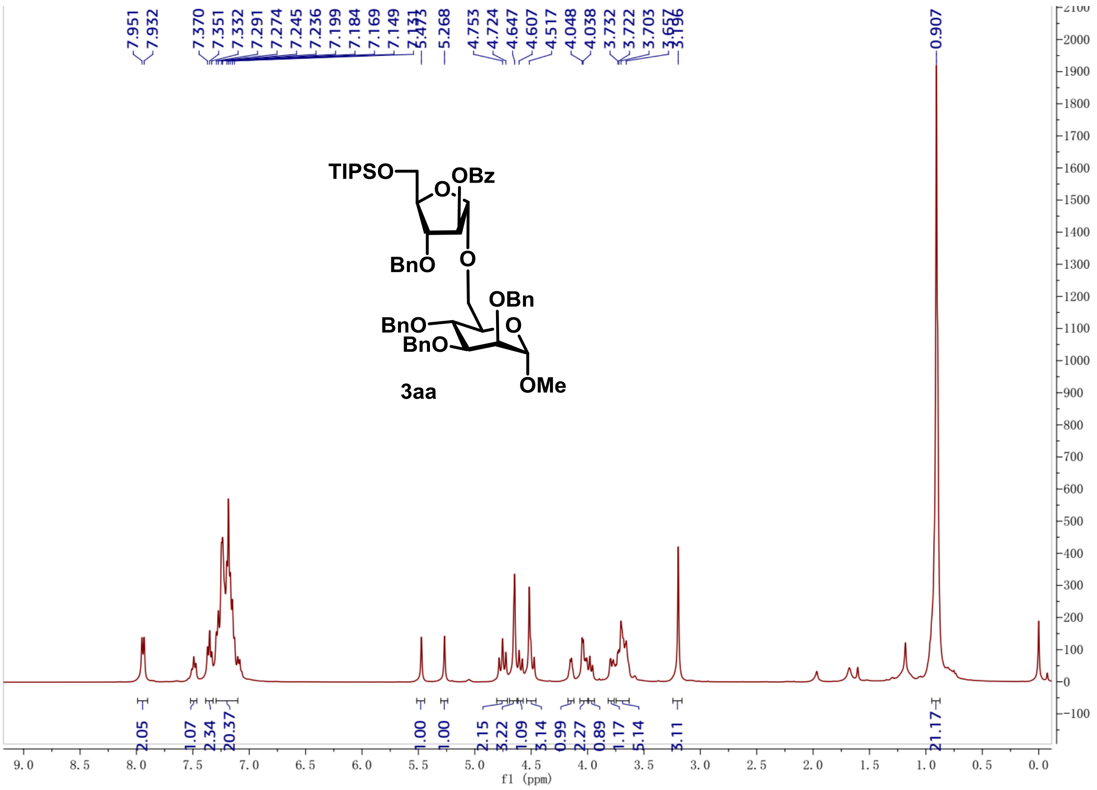

Supplementary Figure 153. ^{1}H NMR Spectrum of Compound **3aa**

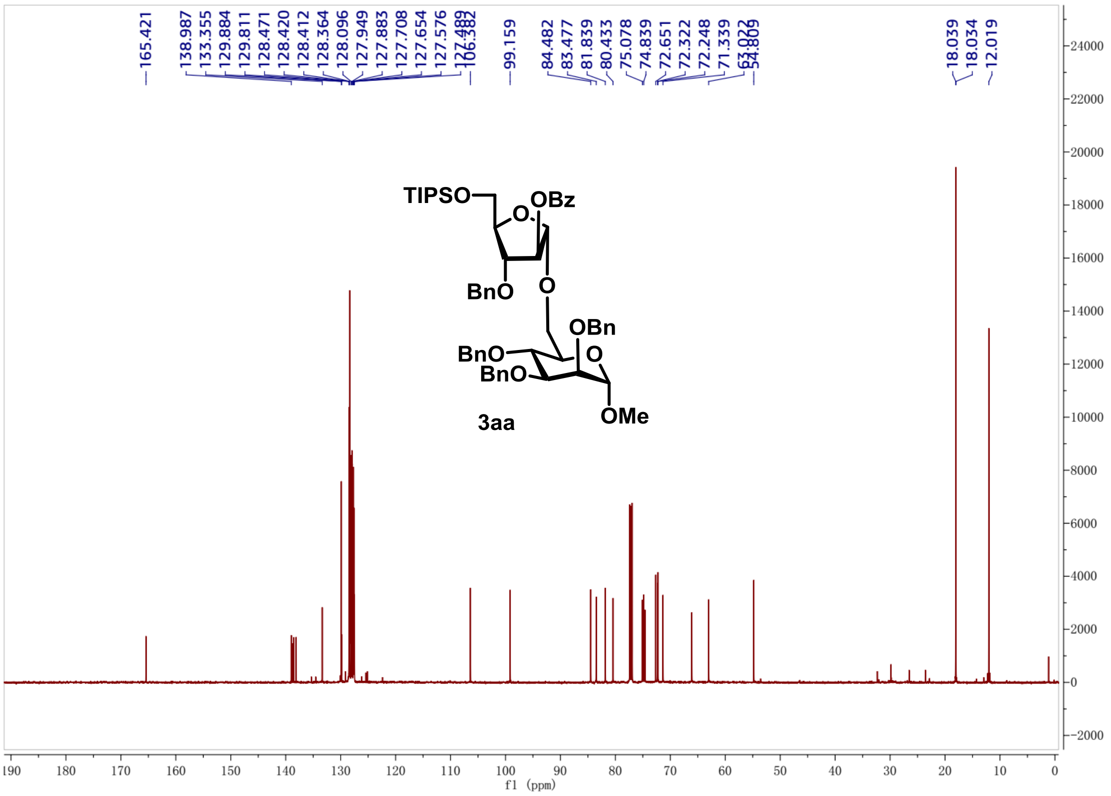

Supplementary Figure 154. ^{13}C NMR Spectrum of Compound **3aa**

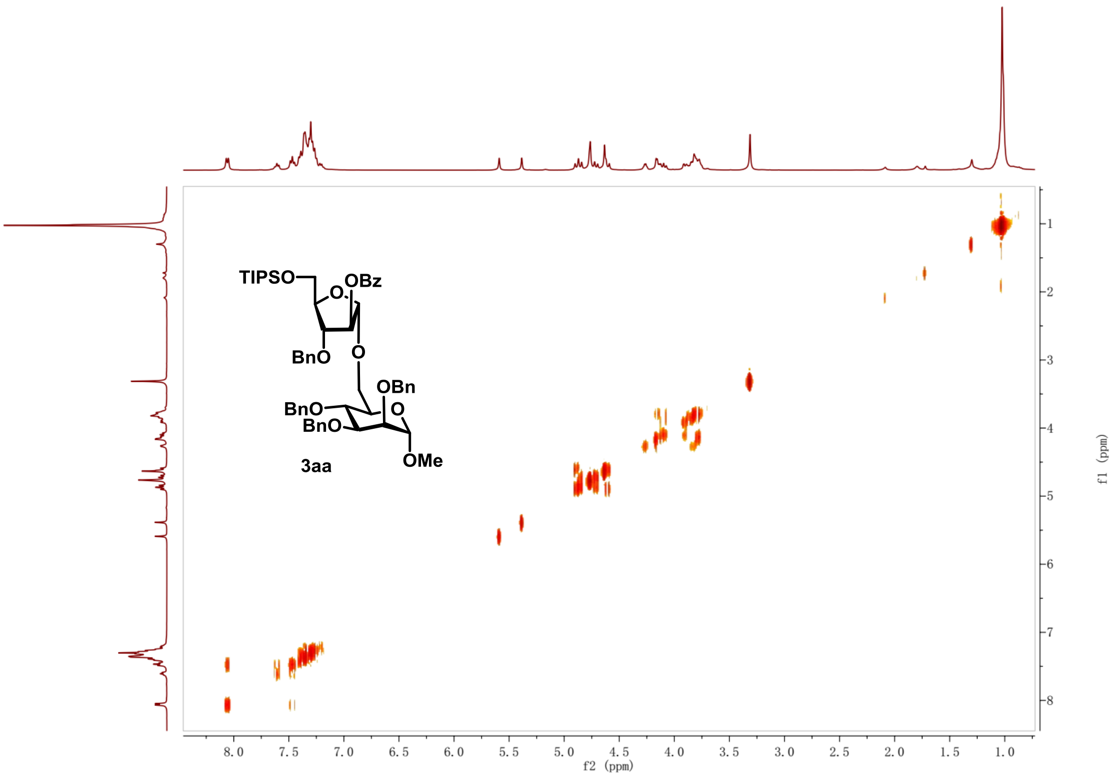

Supplementary Figure 155. COSY NMR Spectrum of Compound **3aa**

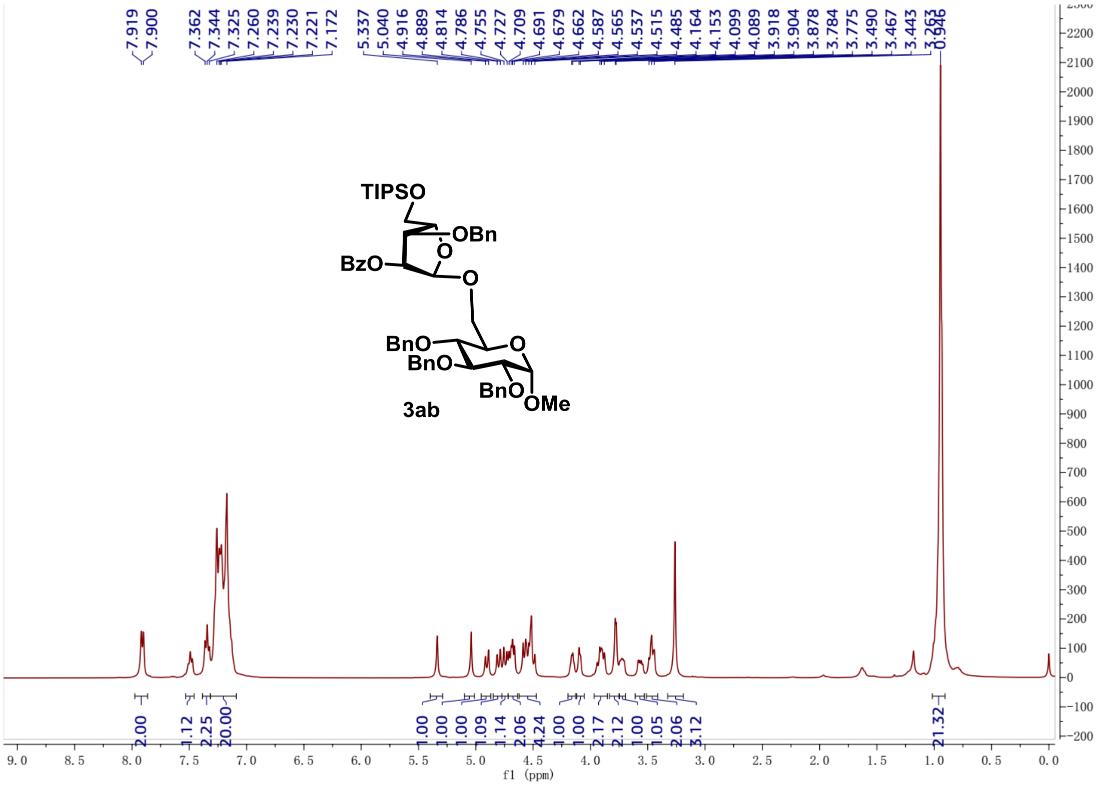

Supplementary Figure 156. ^{1}H NMR Spectrum of Compound **3ab**

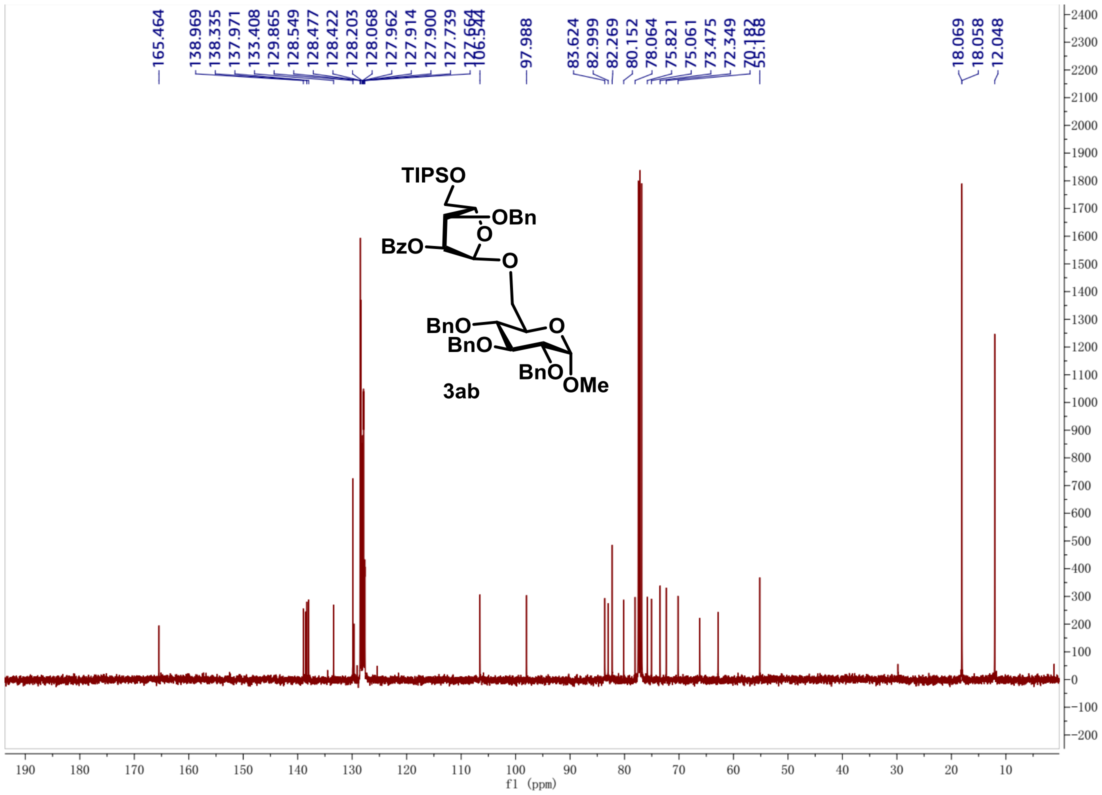

Supplementary Figure 157. ^{13}C NMR Spectrum of Compound **3ab**

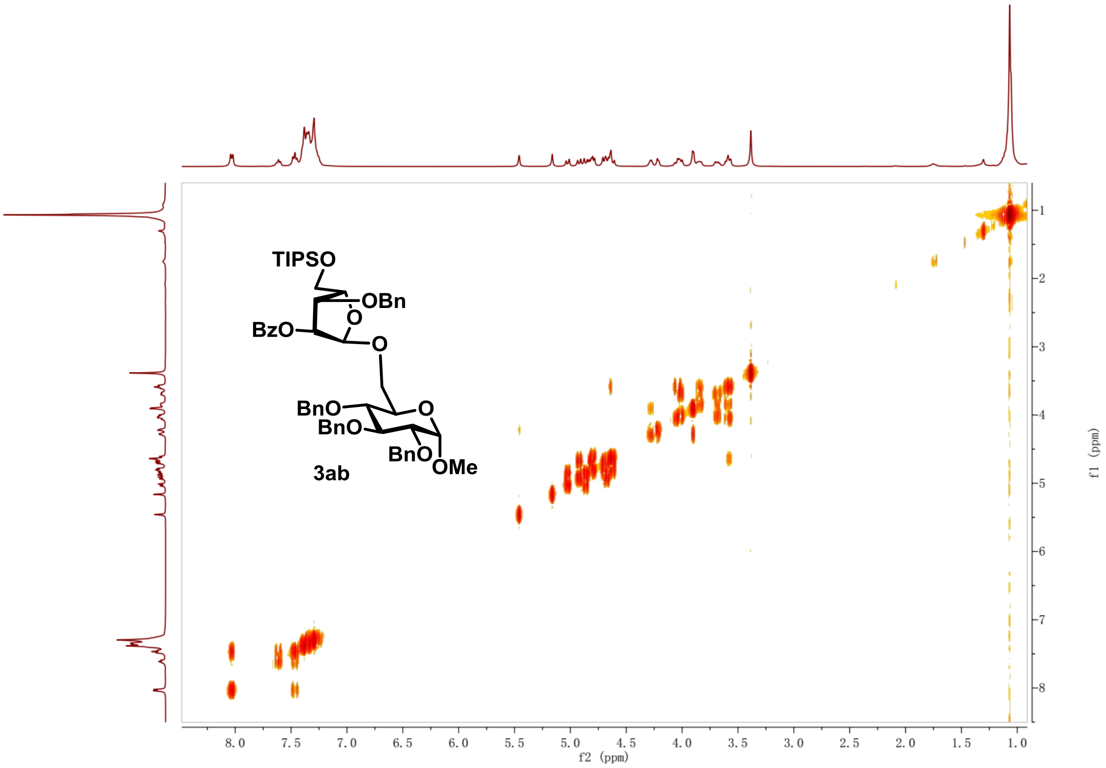

Supplementary Figure 158. COSY NMR Spectrum of Compound **3ab**

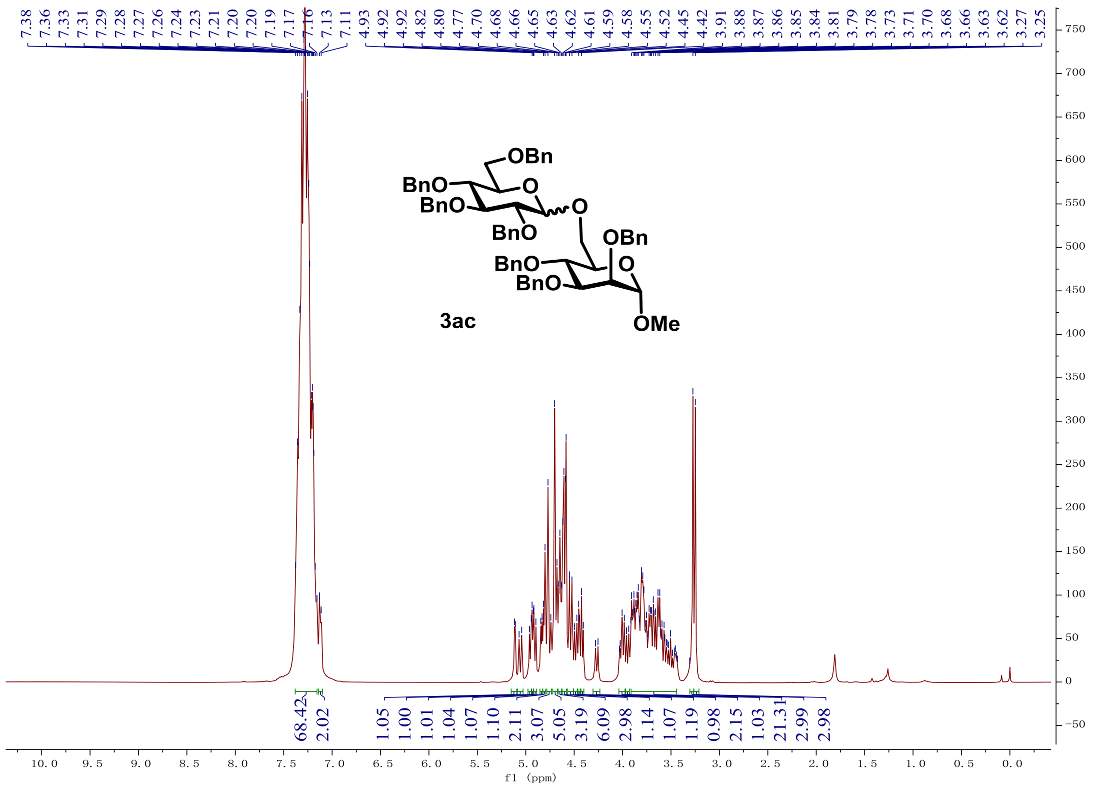

Supplementary Figure 159. ^{1}H NMR Spectrum of Compound **3ac**

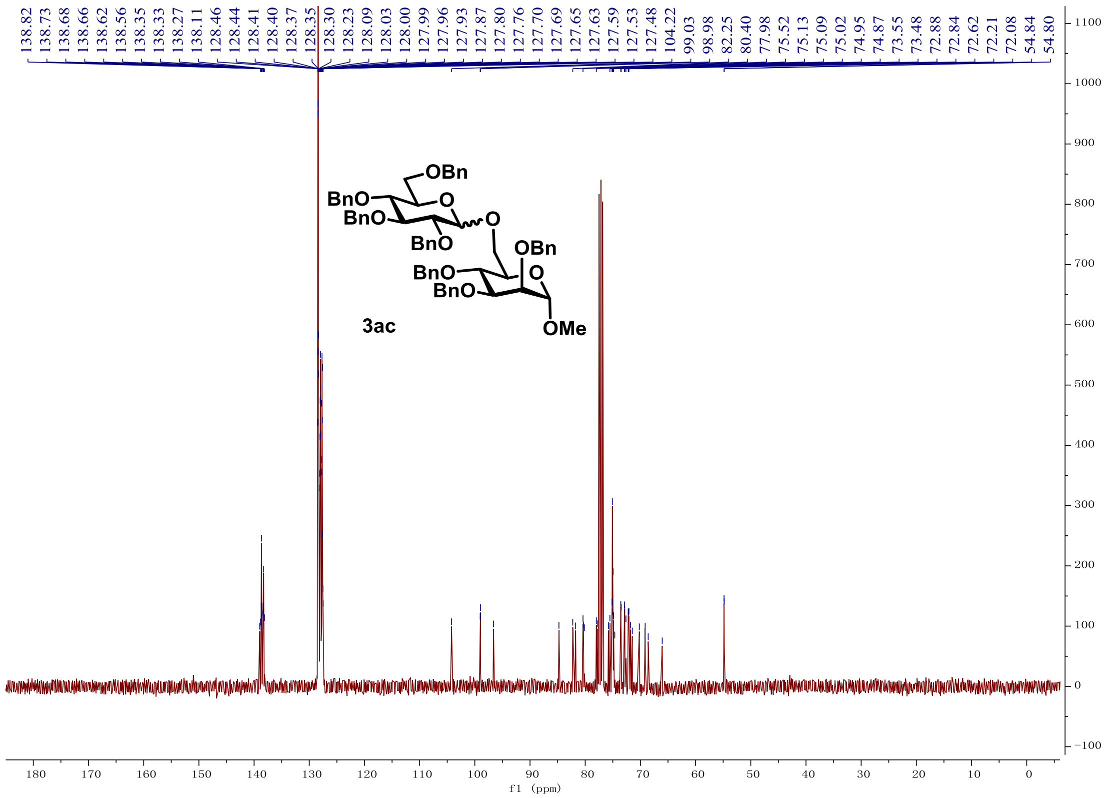

Supplementary Figure 160. ^{13}C NMR Spectrum of Compound **3ac**

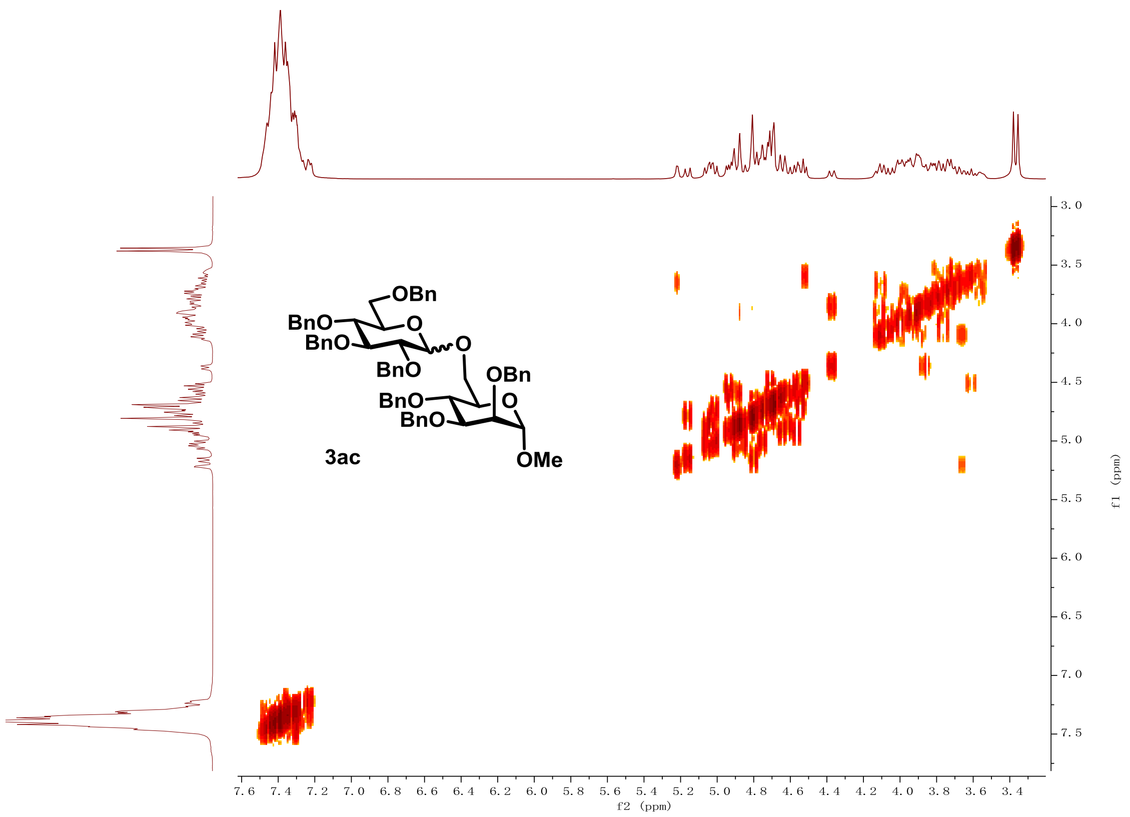

Supplementary Figure 161. COSY NMR Spectrum of Compound **3ac**

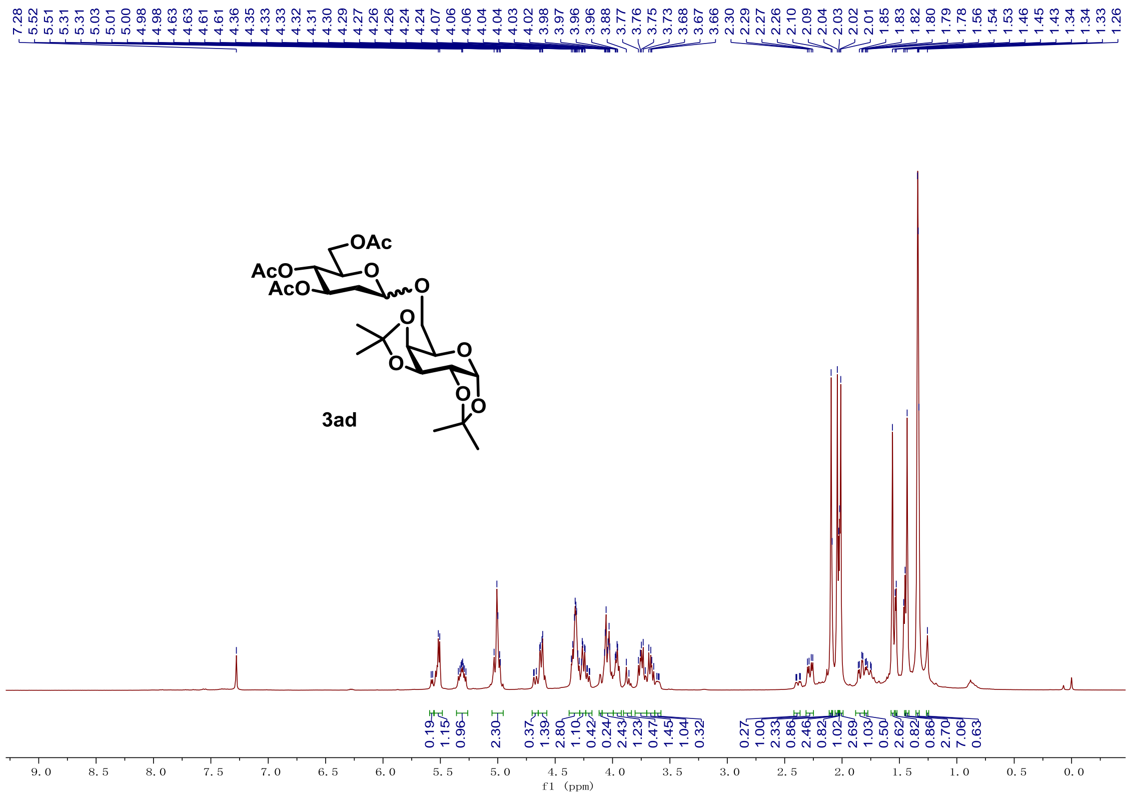

Supplementary Figure 162. ^{1}H NMR Spectrum of Compound **3ad**

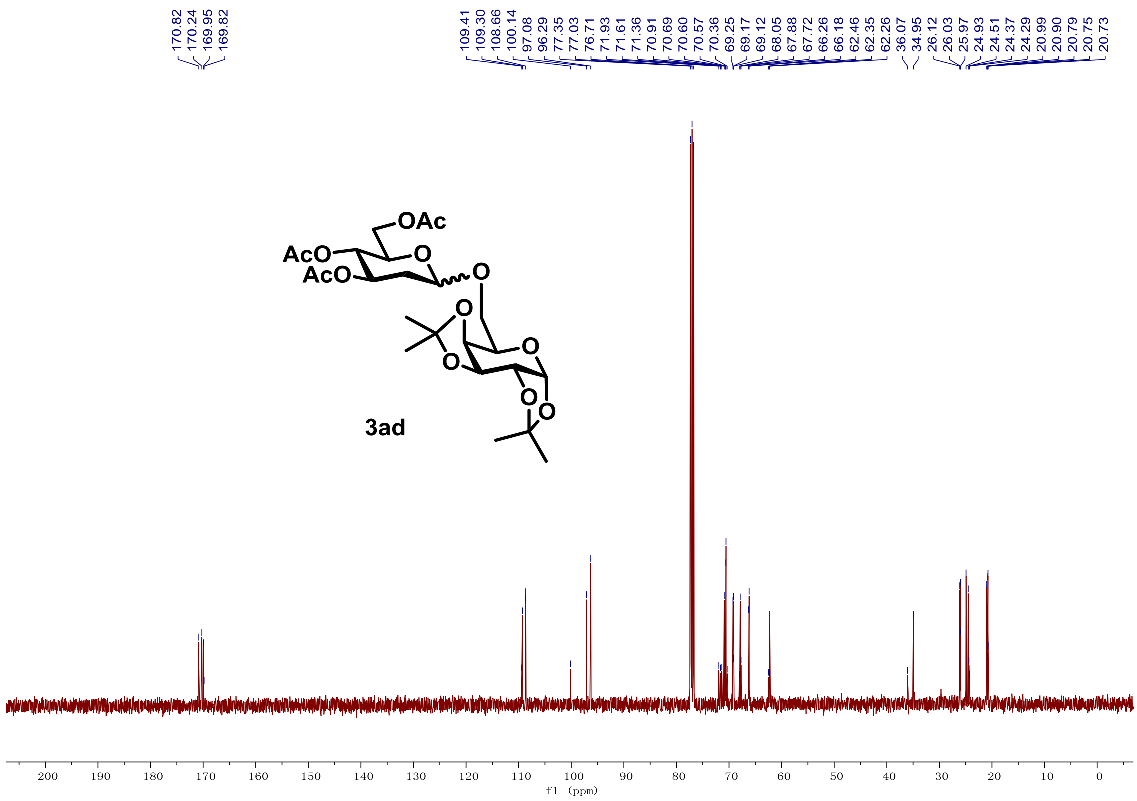

Supplementary Figure 163. ^{13}C NMR Spectrum of Compound **3ad**

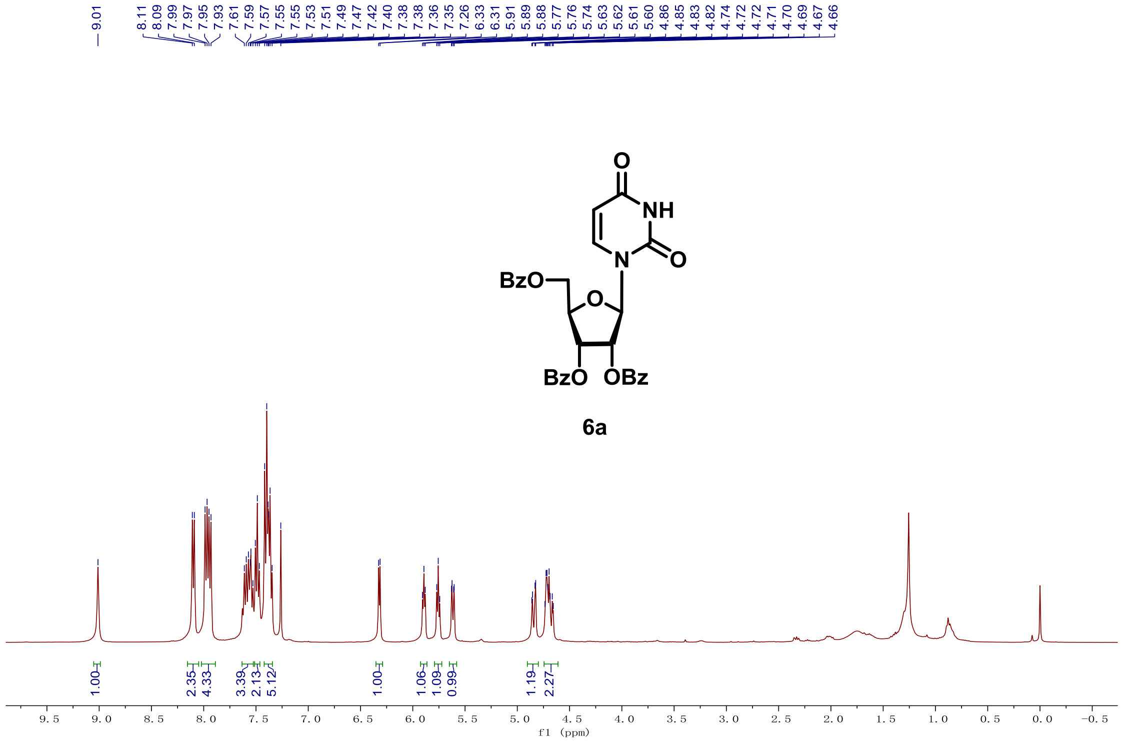

Supplementary Figure 164. ^{1}H NMR Spectrum of Compound **6a**

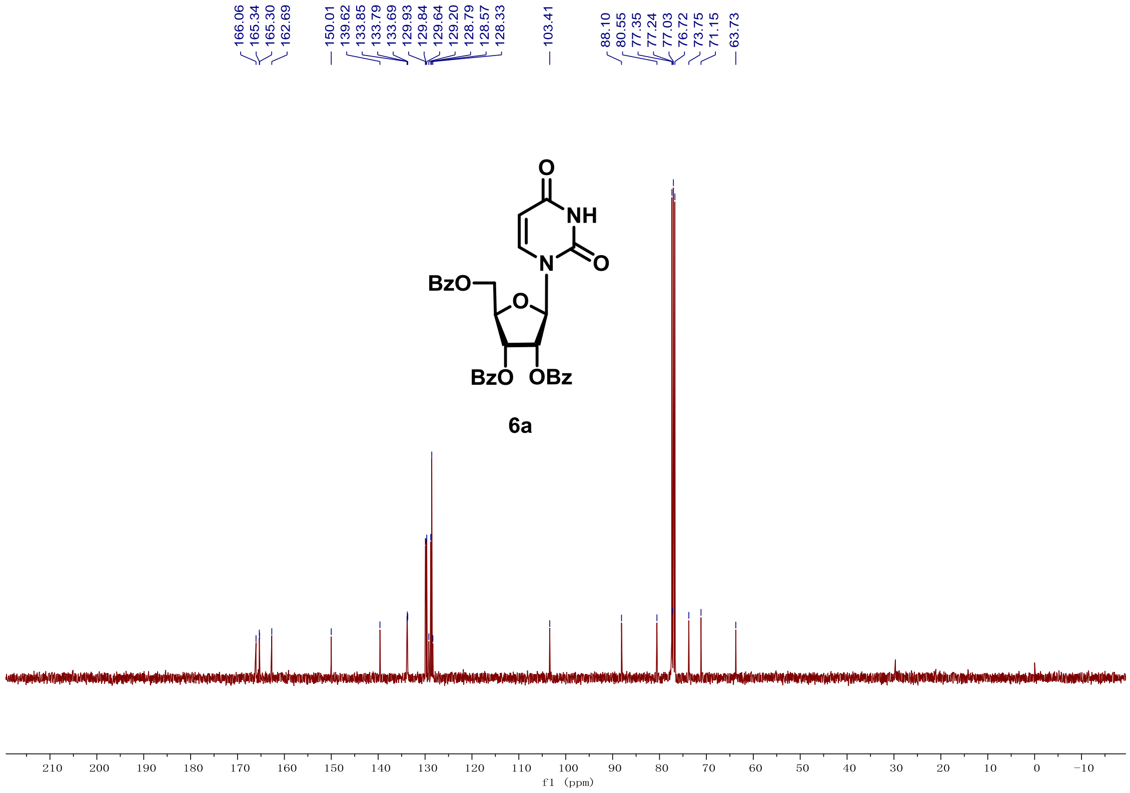

Supplementary Figure 165. ^{13}C NMR Spectrum of Compound **6a**

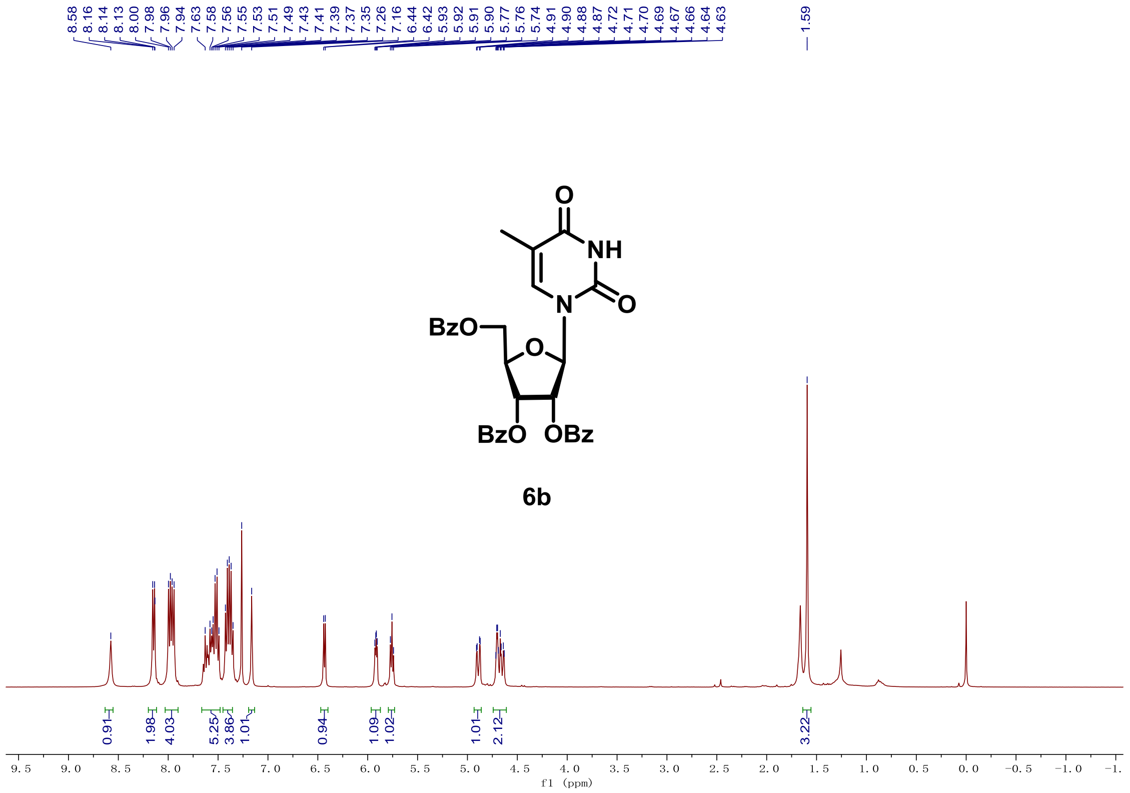

Supplementary Figure 166. ^{1}H NMR Spectrum of Compound **6b**

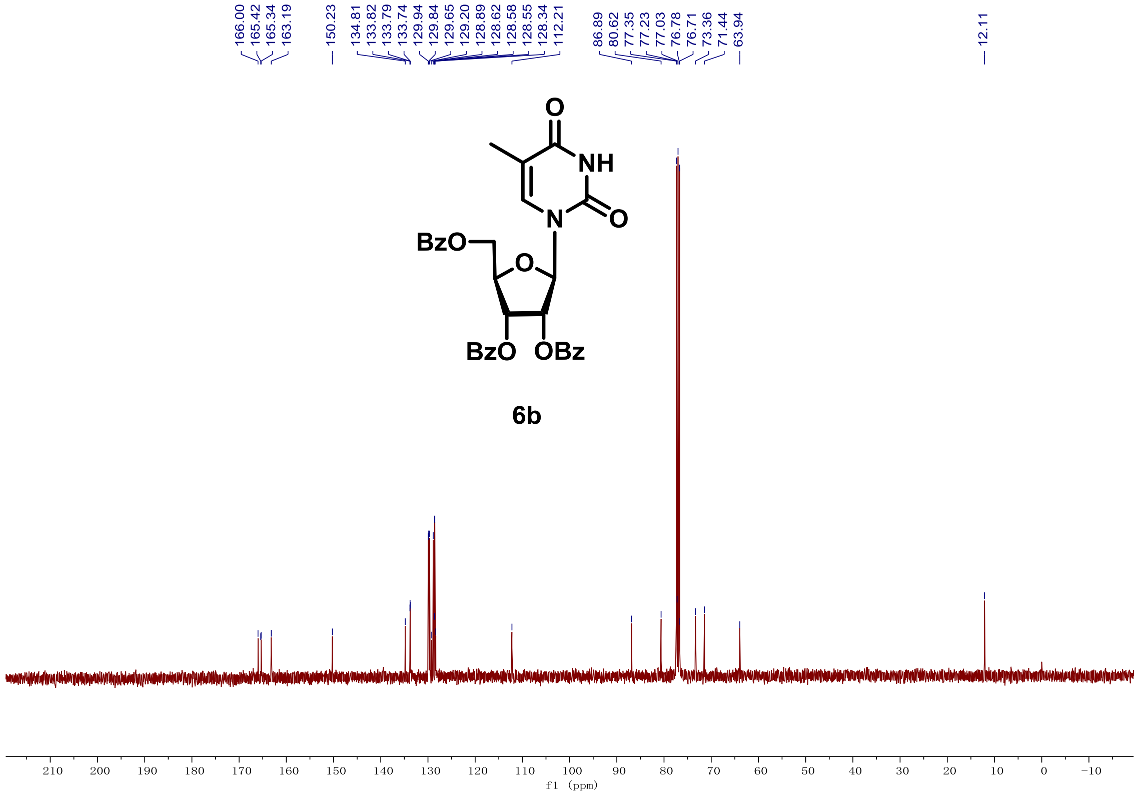

Supplementary Figure 167. ^{13}C NMR Spectrum of Compound **6b**

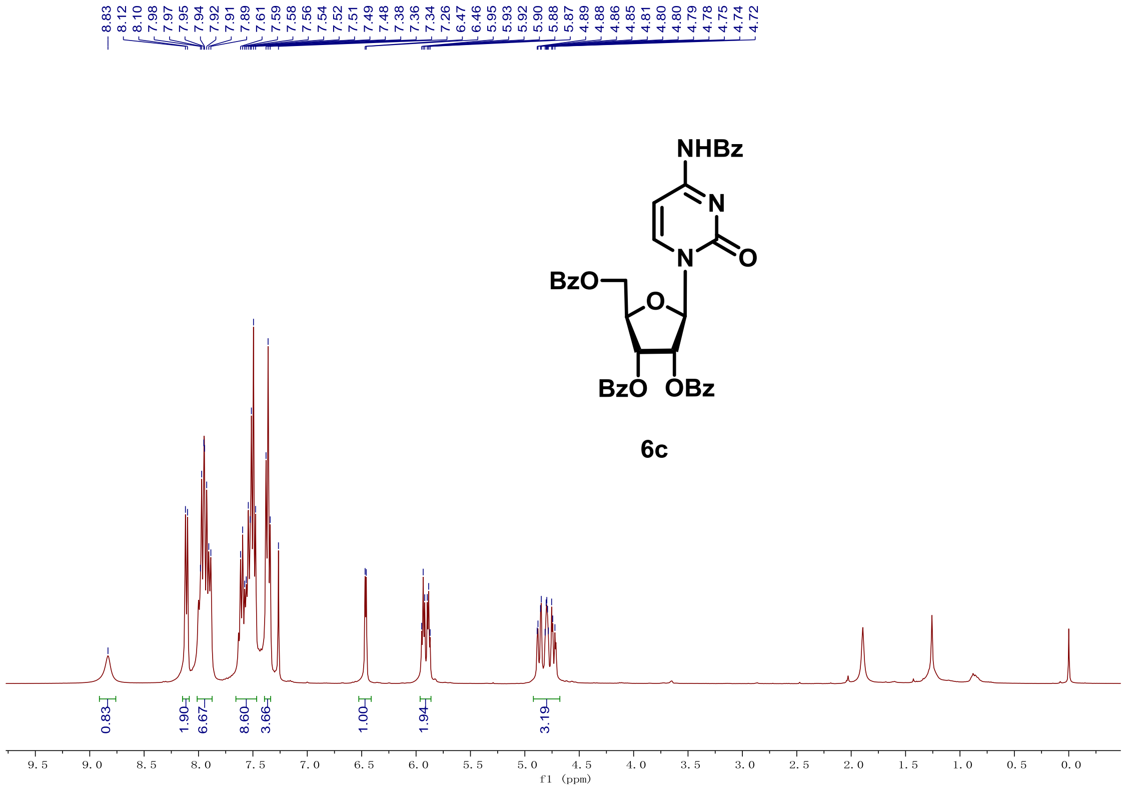

Supplementary Figure 168. ^{1}H NMR Spectrum of Compound **6c**

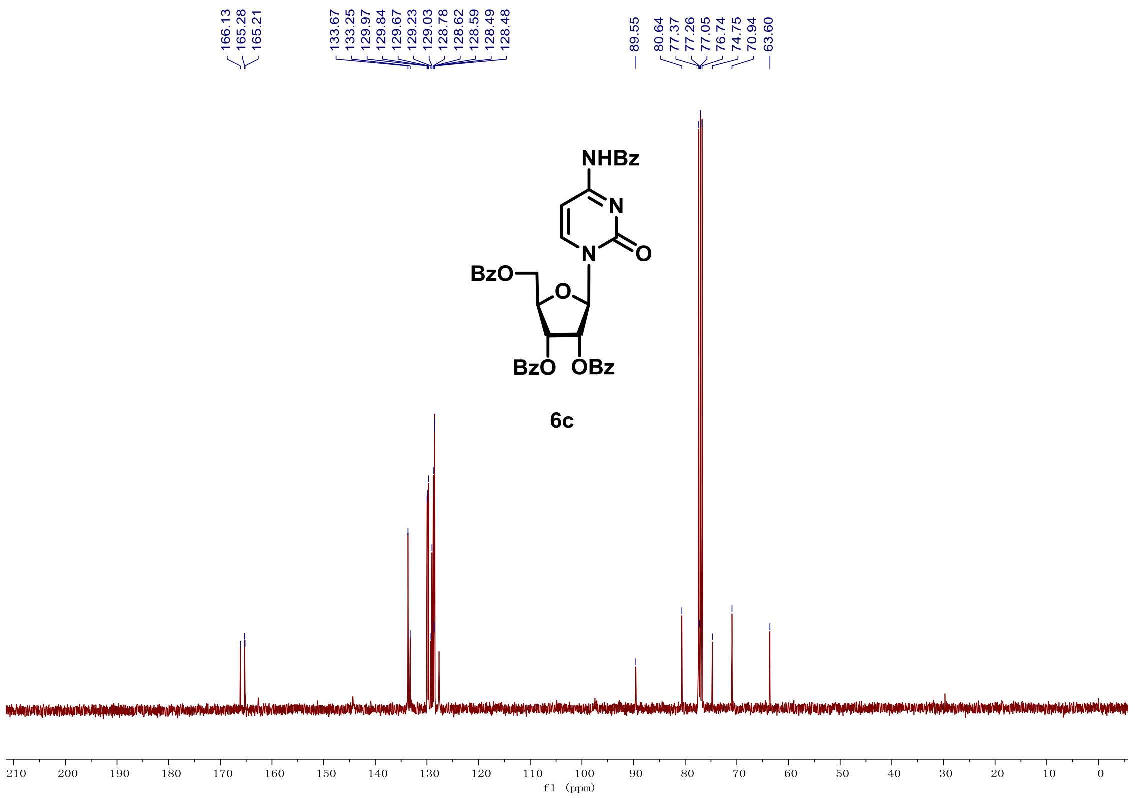

Supplementary Figure 169. ^{13}C NMR Spectrum of Compound **6c**

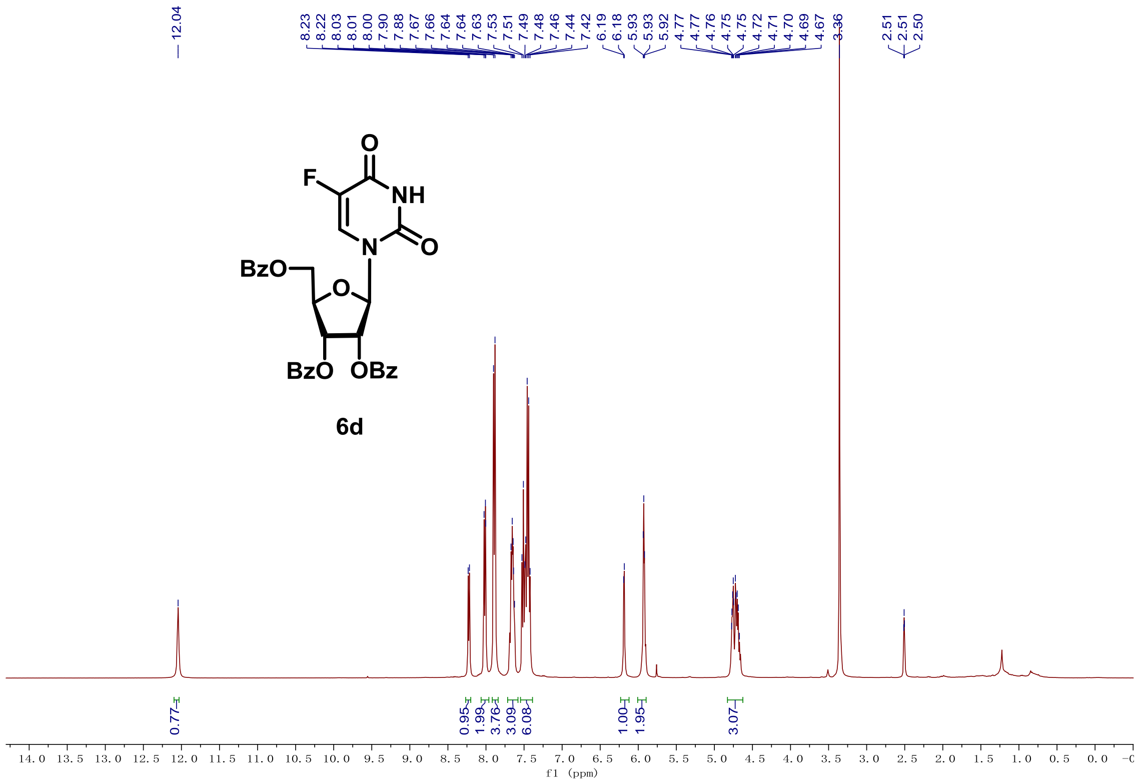

Supplementary Figure 170. ^{1}H NMR Spectrum of Compound **6d**

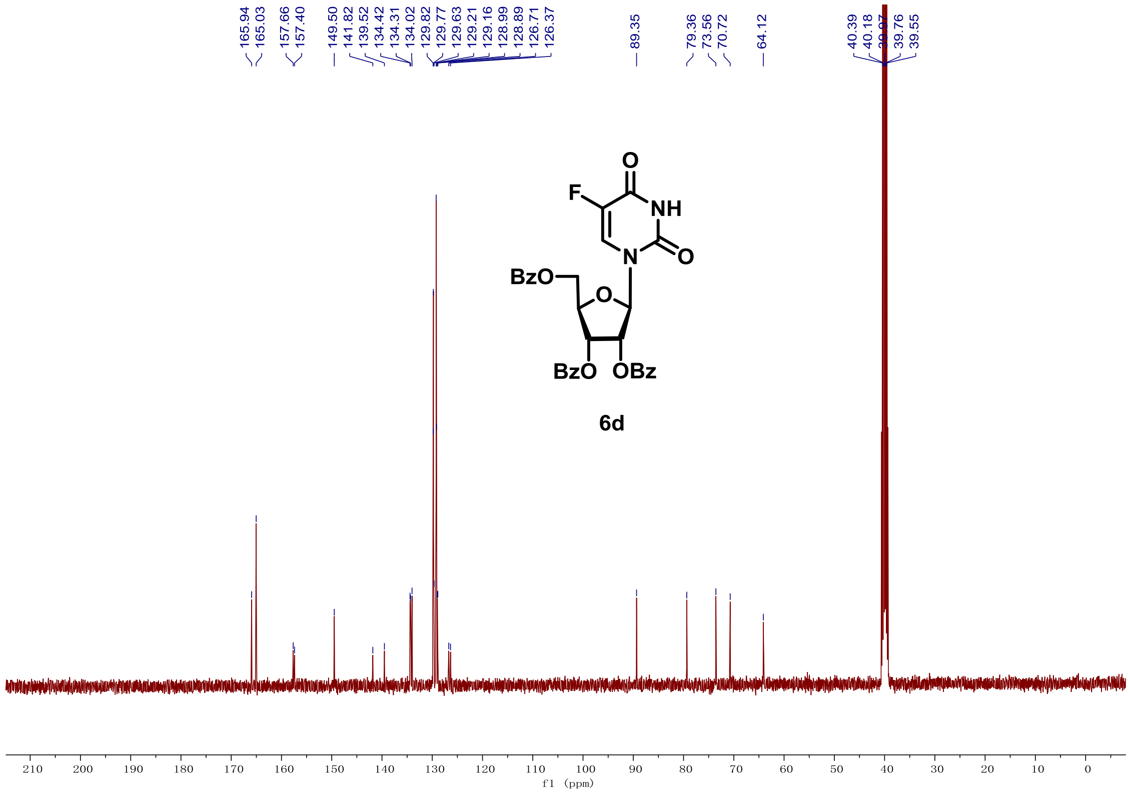

Supplementary Figure 171. ^{13}C NMR Spectrum of Compound **6d**

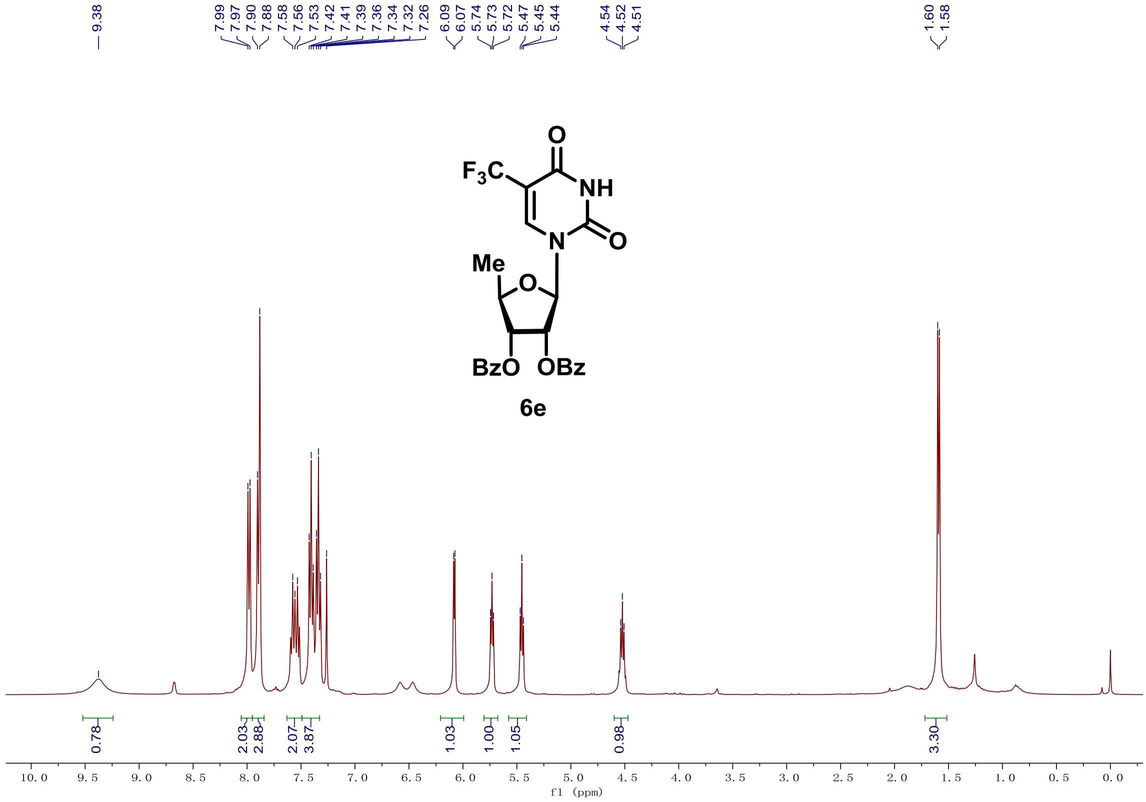

Supplementary Figure 172. ^{1}H NMR Spectrum of Compound **6e**

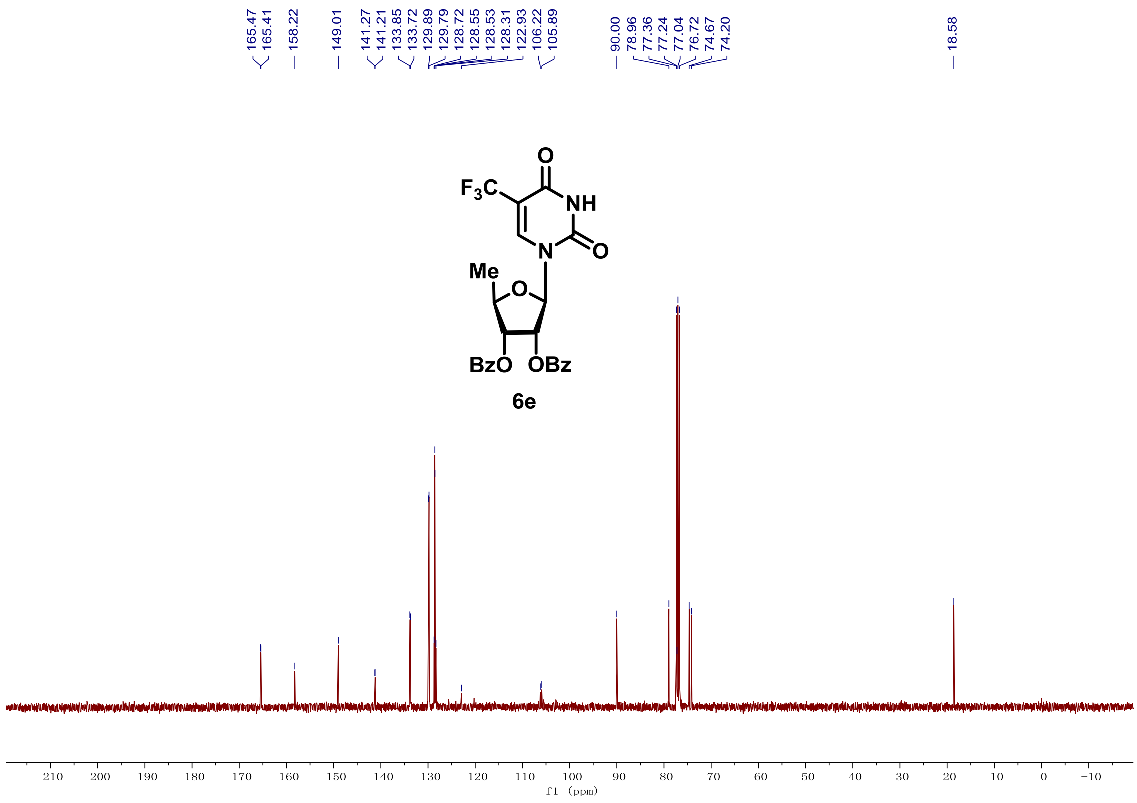

Supplementary Figure 173. ^{13}C NMR Spectrum of Compound **6e**

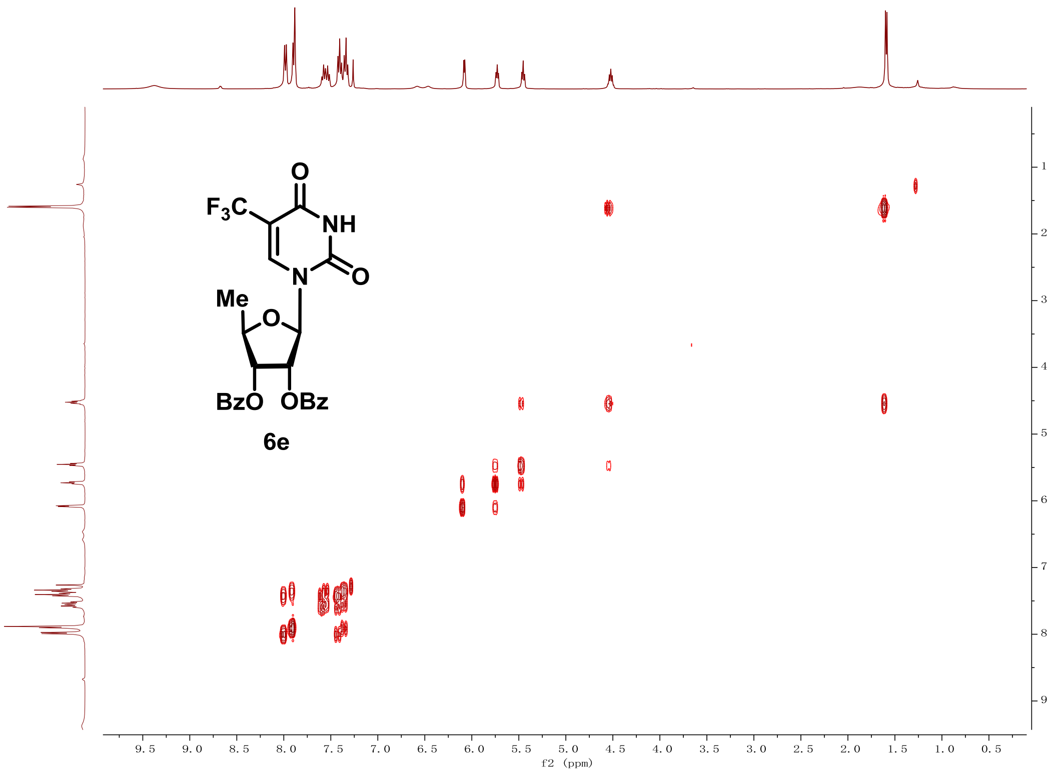

Supplementary Figure 174. COSY NMR Spectrum of Compound **6e**

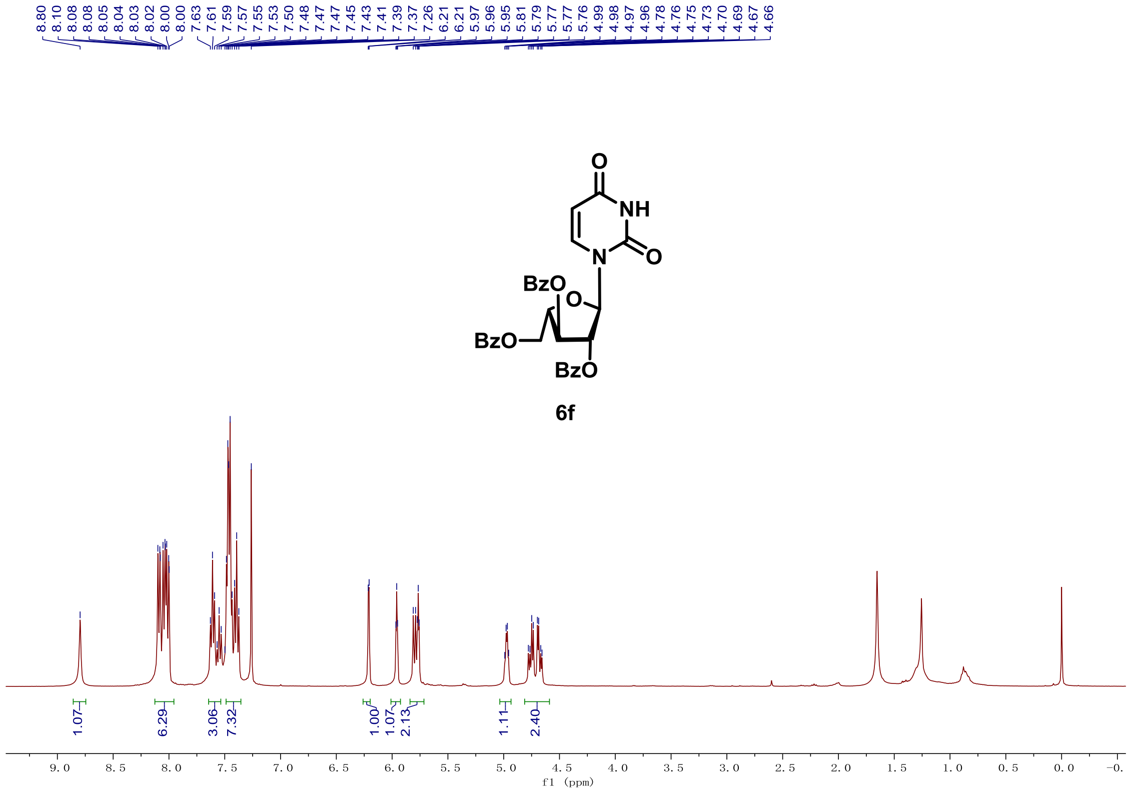

Supplementary Figure 175. ^{1}H NMR Spectrum of Compound **6f**

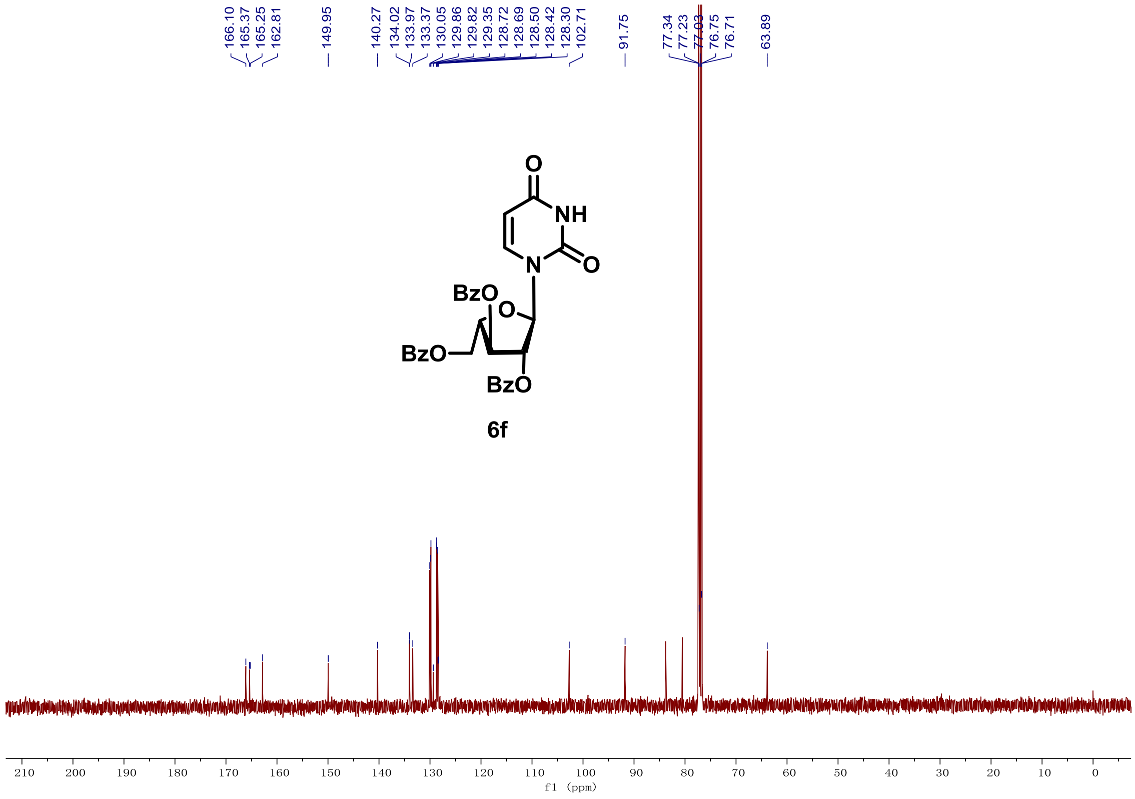

Supplementary Figure 176. ^{13}C NMR Spectrum of Compound **6f**

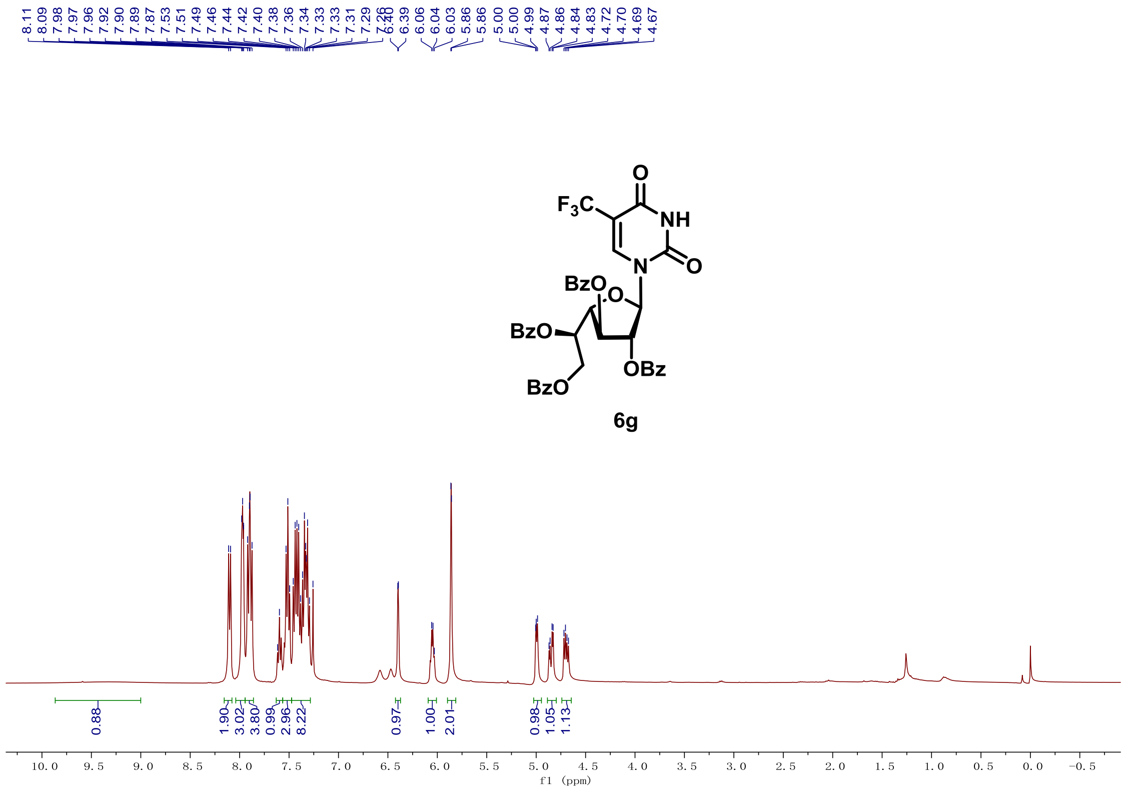

Supplementary Figure 177. ^{1}H NMR Spectrum of Compound **6g**

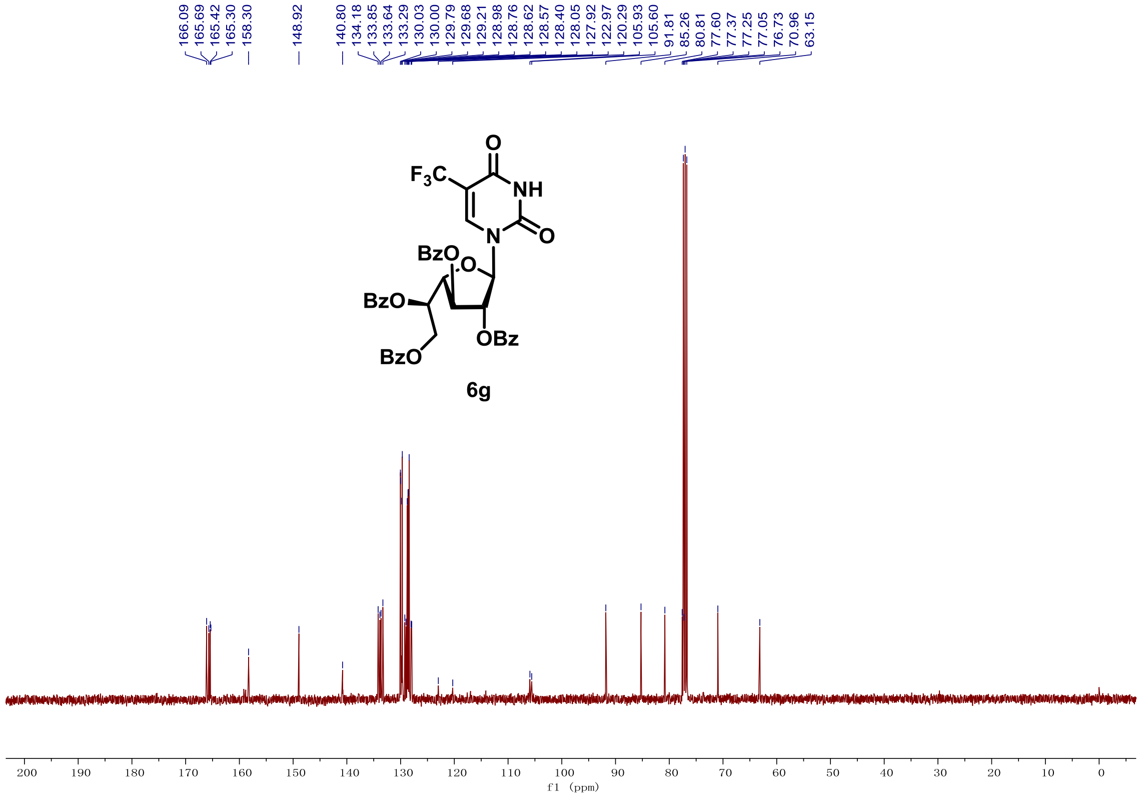

Supplementary Figure 178. ^{13}C NMR Spectrum of Compound **6g**

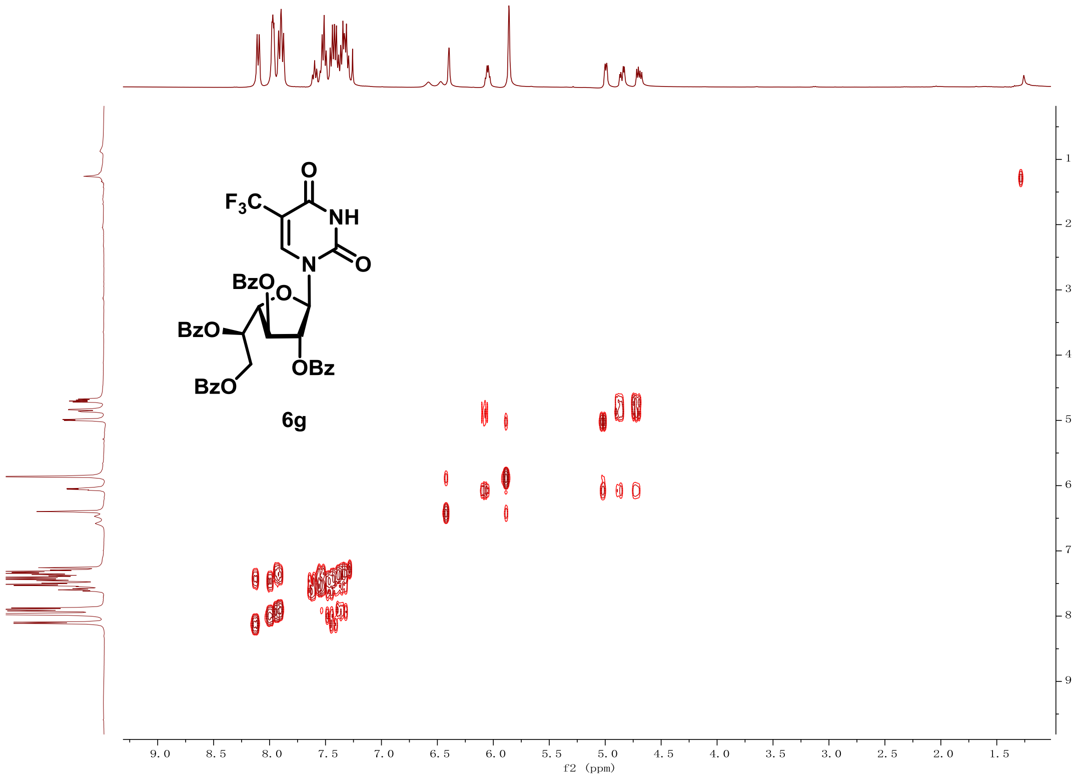

Supplementary Figure 179. COSY NMR Spectrum of Compound **6g**

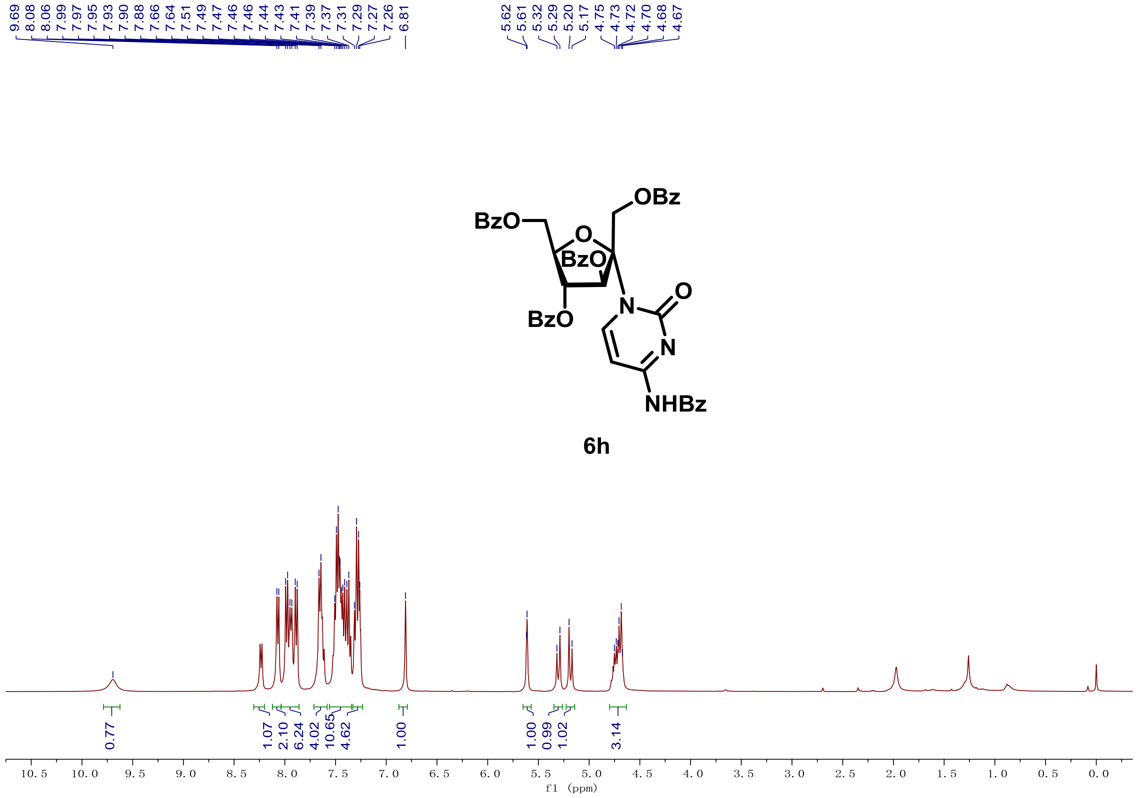

Supplementary Figure 180. ^{1}H NMR Spectrum of Compound **6h**

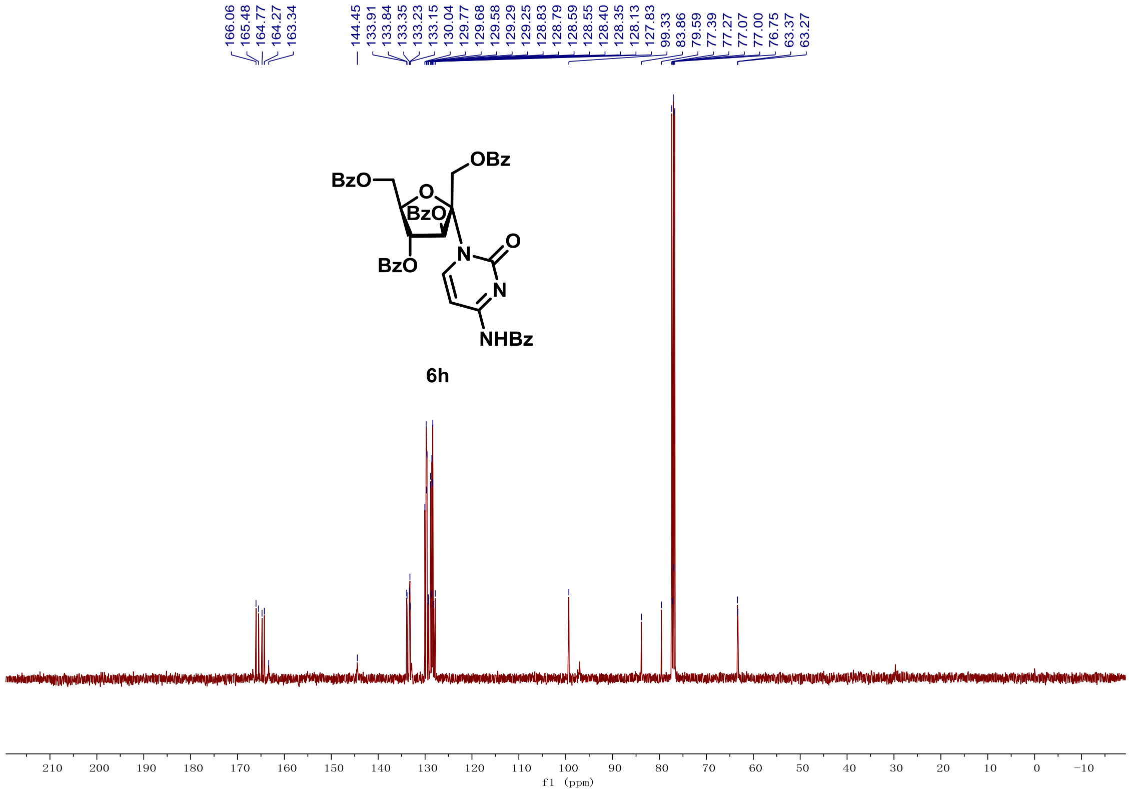

Supplementary Figure 181. ^{13}C NMR Spectrum of Compound **6h**

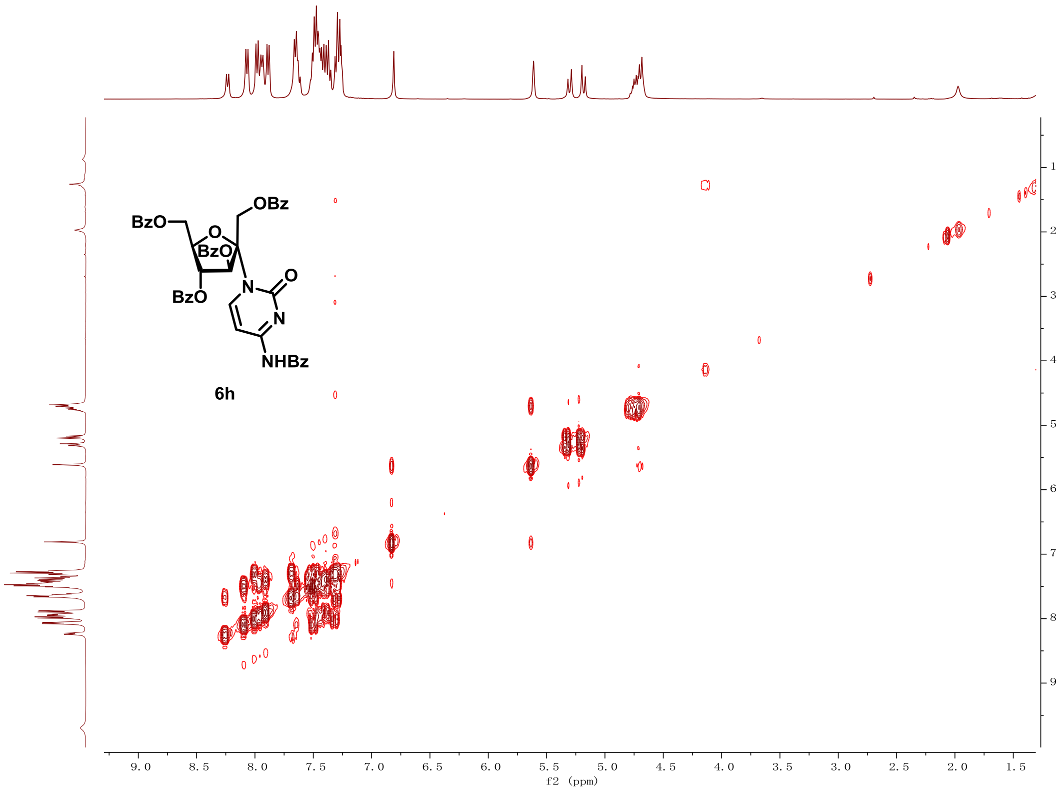

Supplementary Figure 182. COSY NMR Spectrum of Compound **6h**

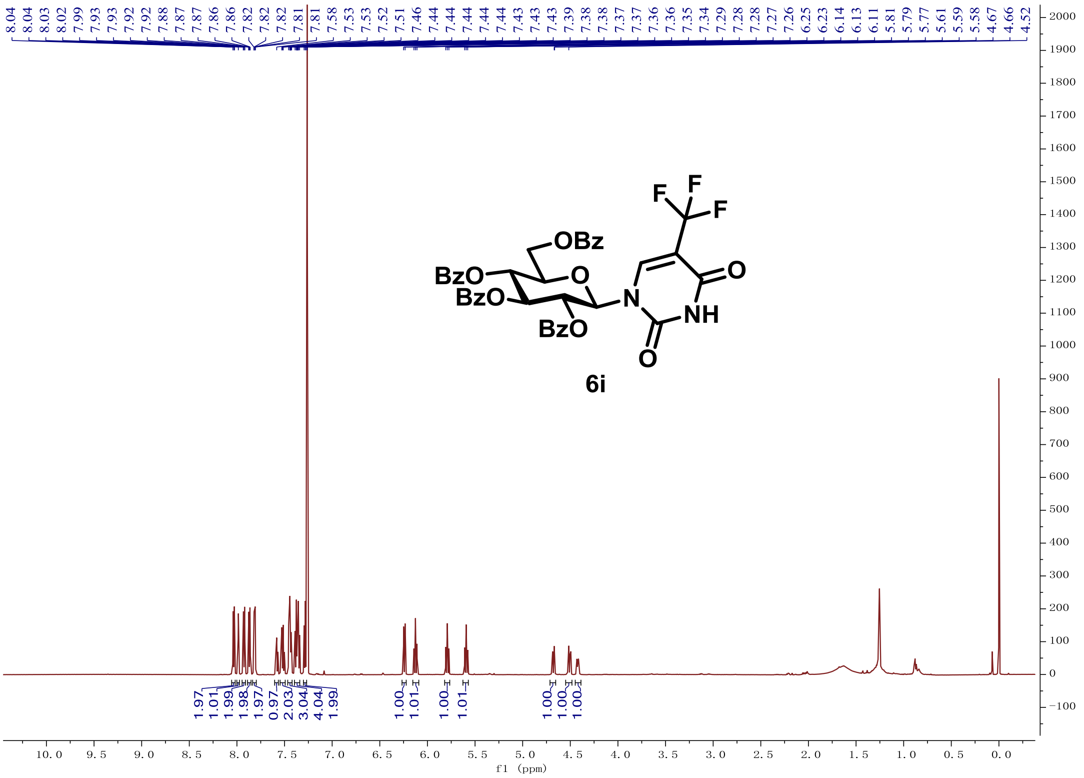

Supplementary Figure 183. ^{1}H NMR Spectrum of Compound **6i**

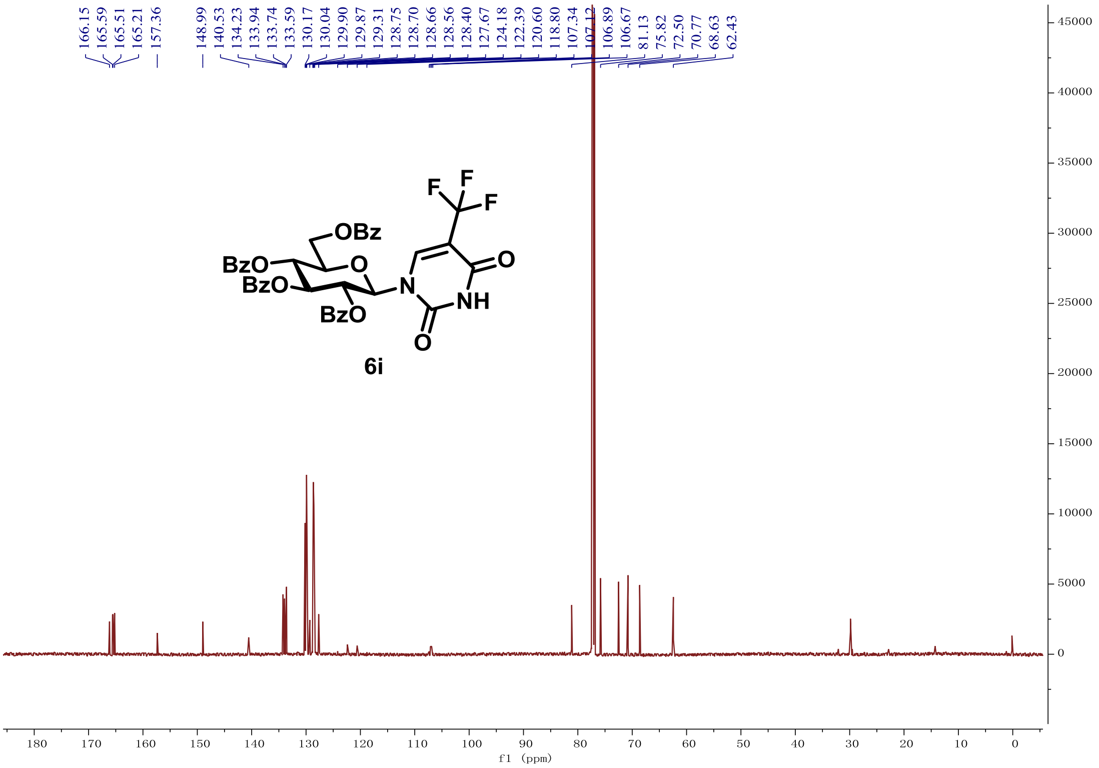

Supplementary Figure 184. ^{13}C NMR Spectrum of Compound **6i**

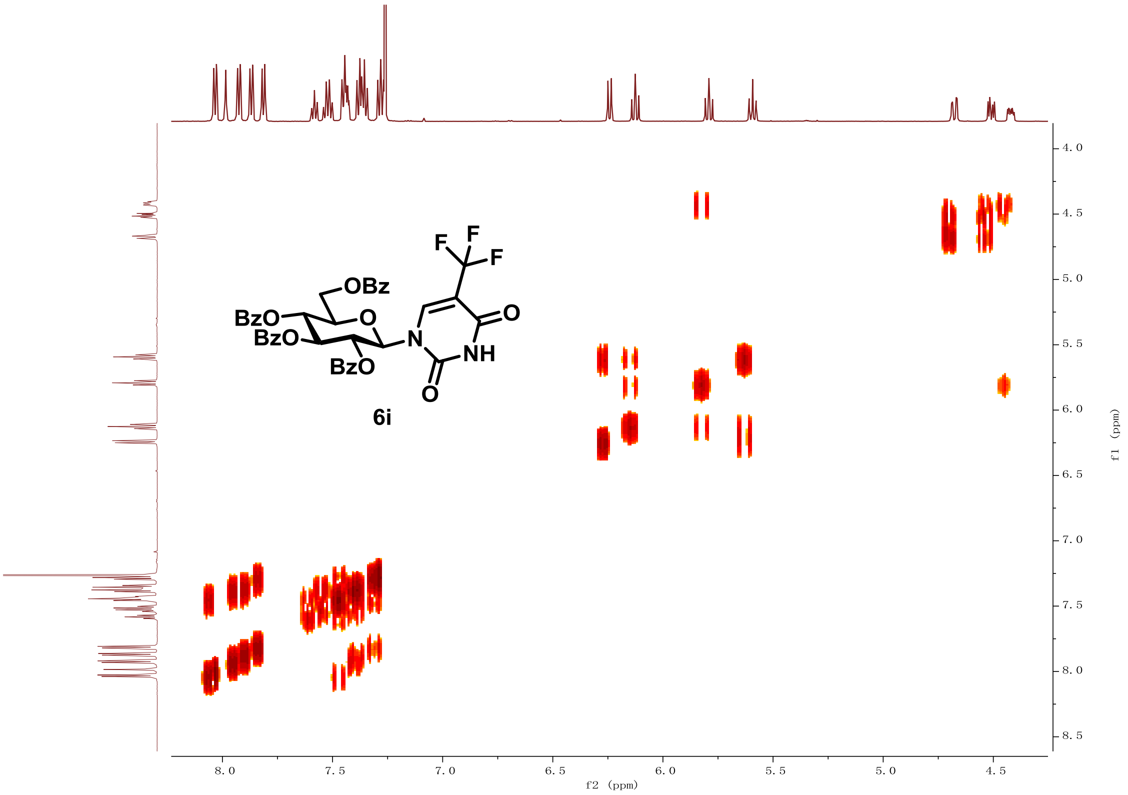

Supplementary Figure 185. COSY NMR Spectrum of Compound **6i**

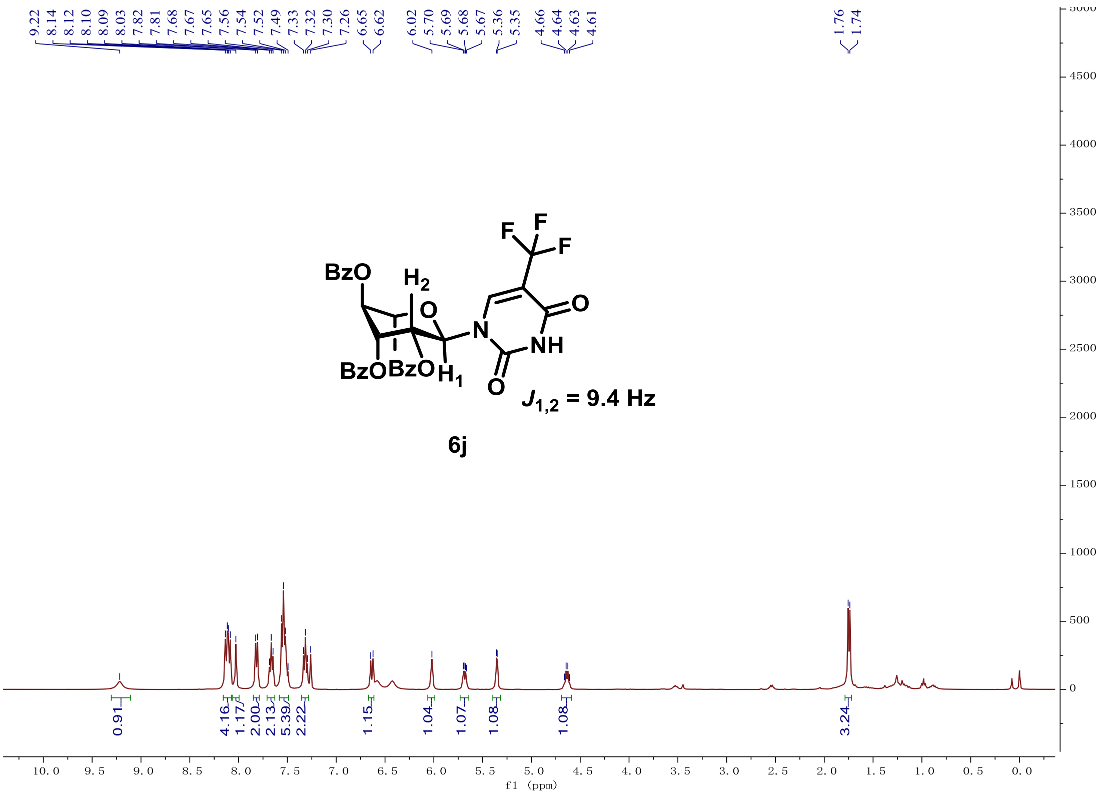

Supplementary Figure 186. ^{1}H NMR Spectrum of Compound **6j**

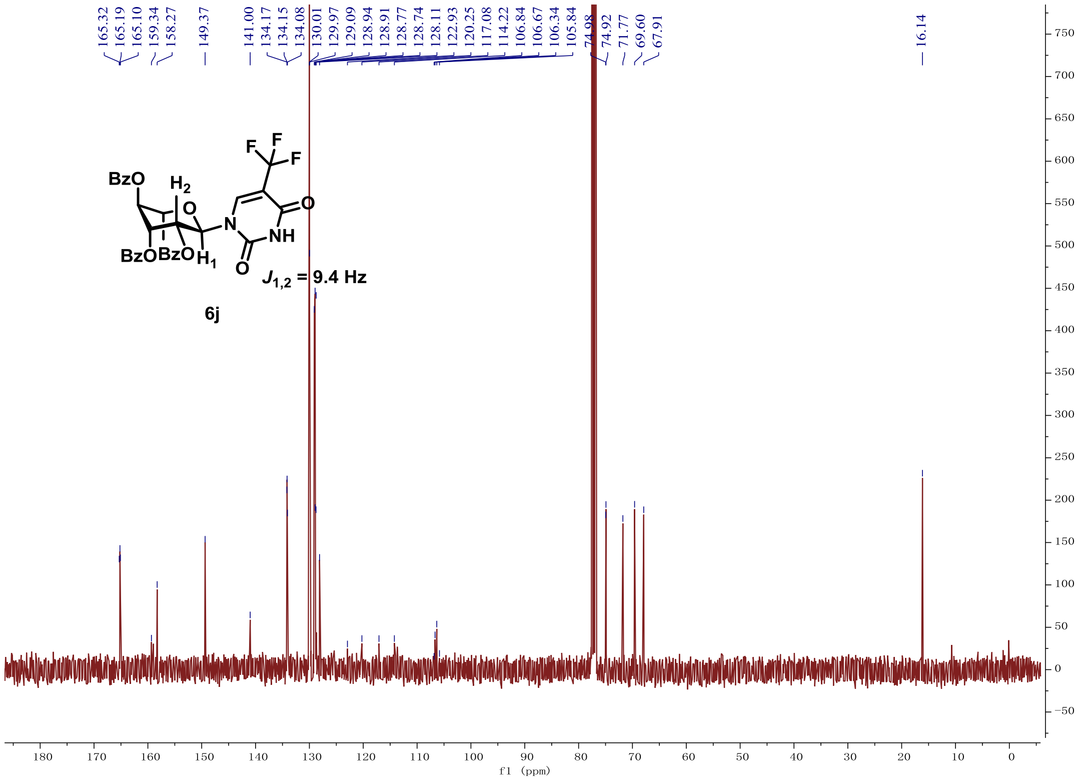

Supplementary Figure 187. ^{13}C NMR Spectrum of Compound **6j**

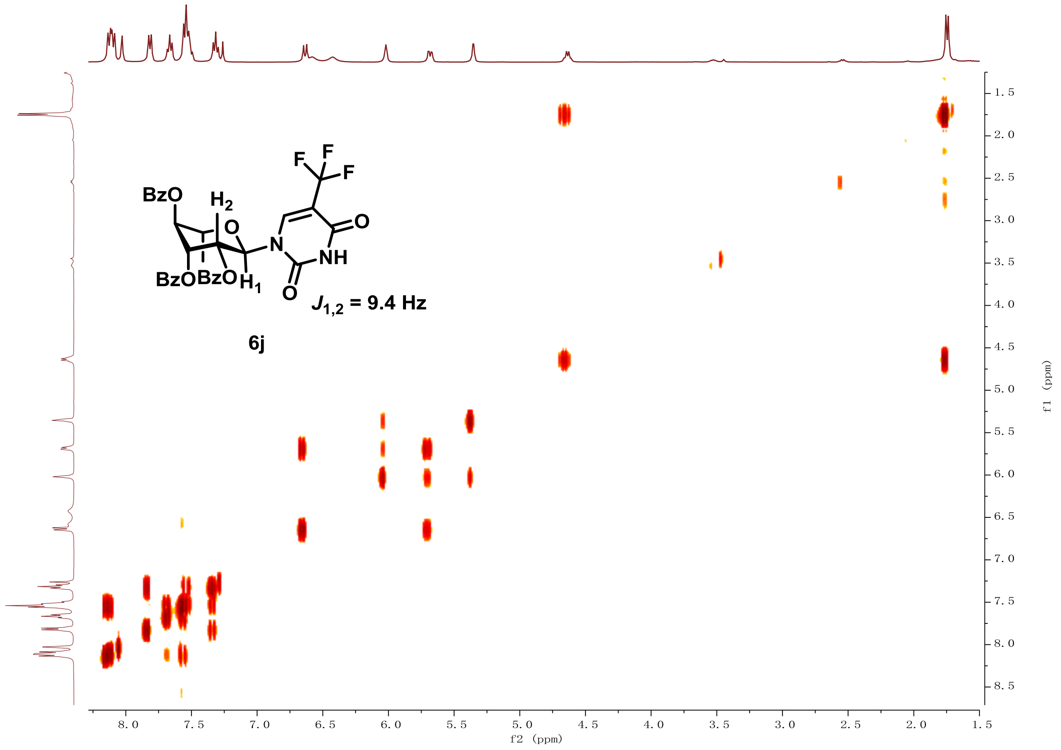

Supplementary Figure 188. COSY NMR Spectrum of Compound **6j**

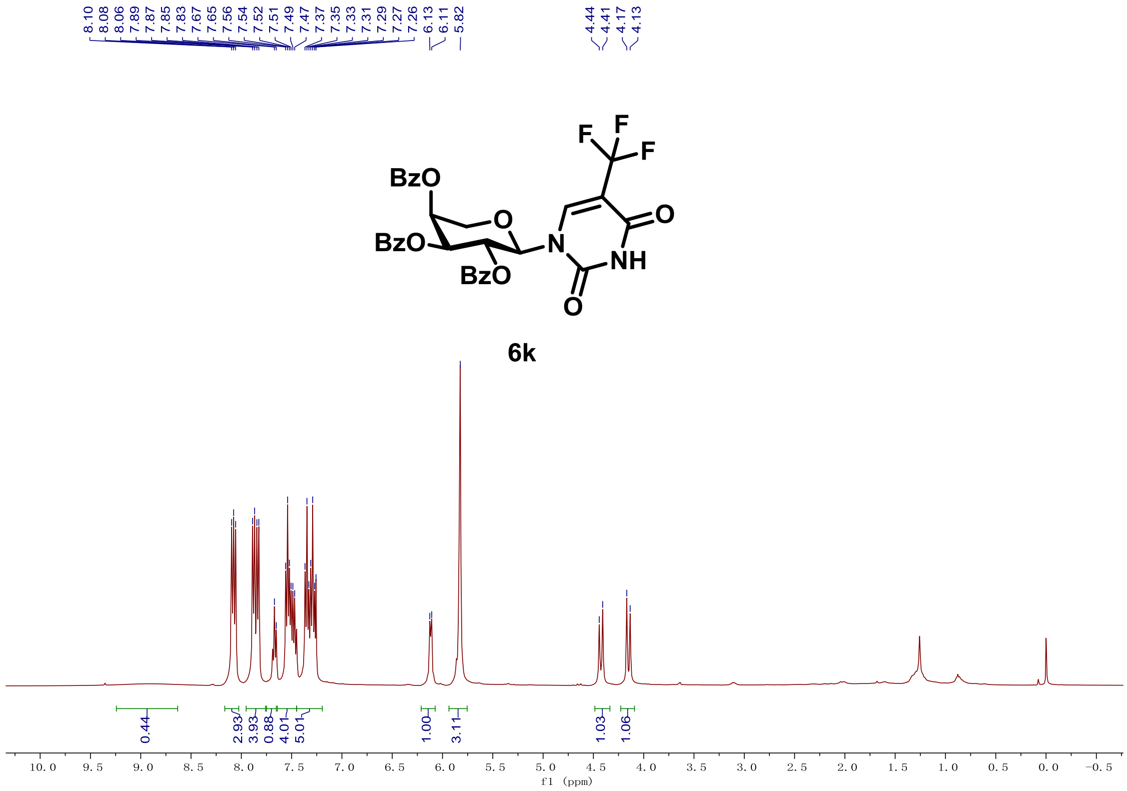

Supplementary Figure 189. ^{1}H NMR Spectrum of Compound **6k**

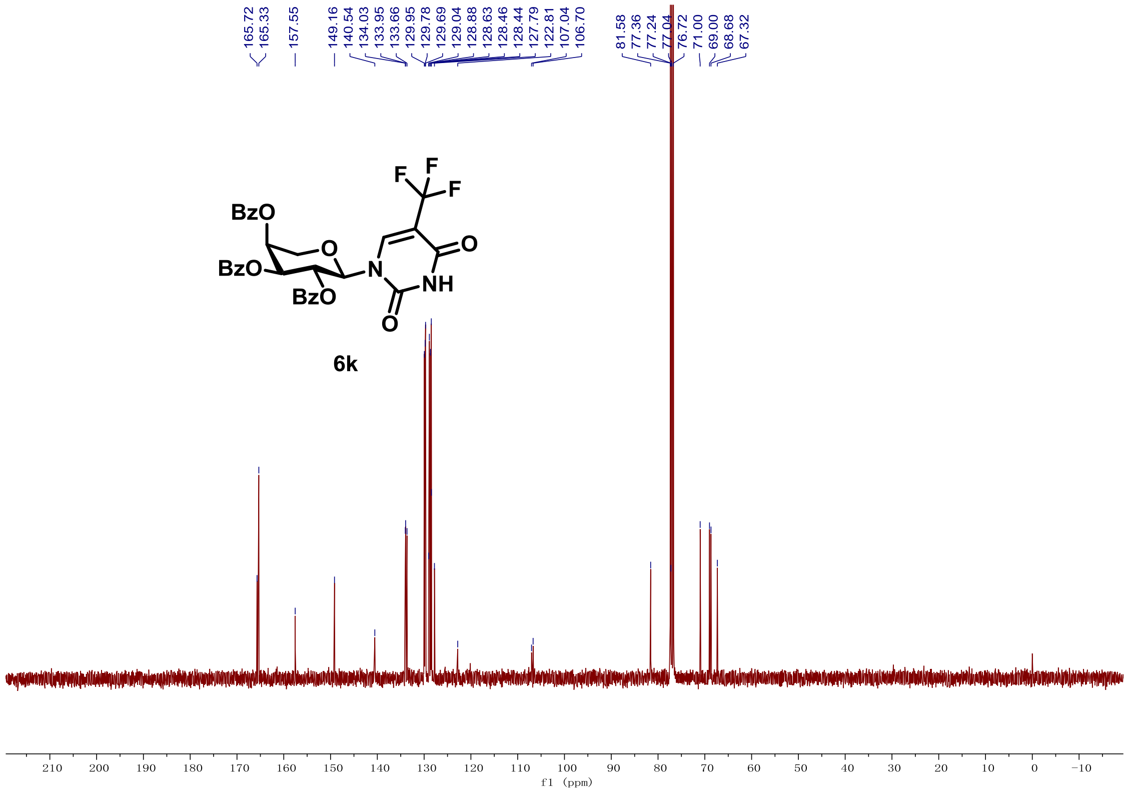

Supplementary Figure 190. ^{13}C NMR Spectrum of Compound **6k**

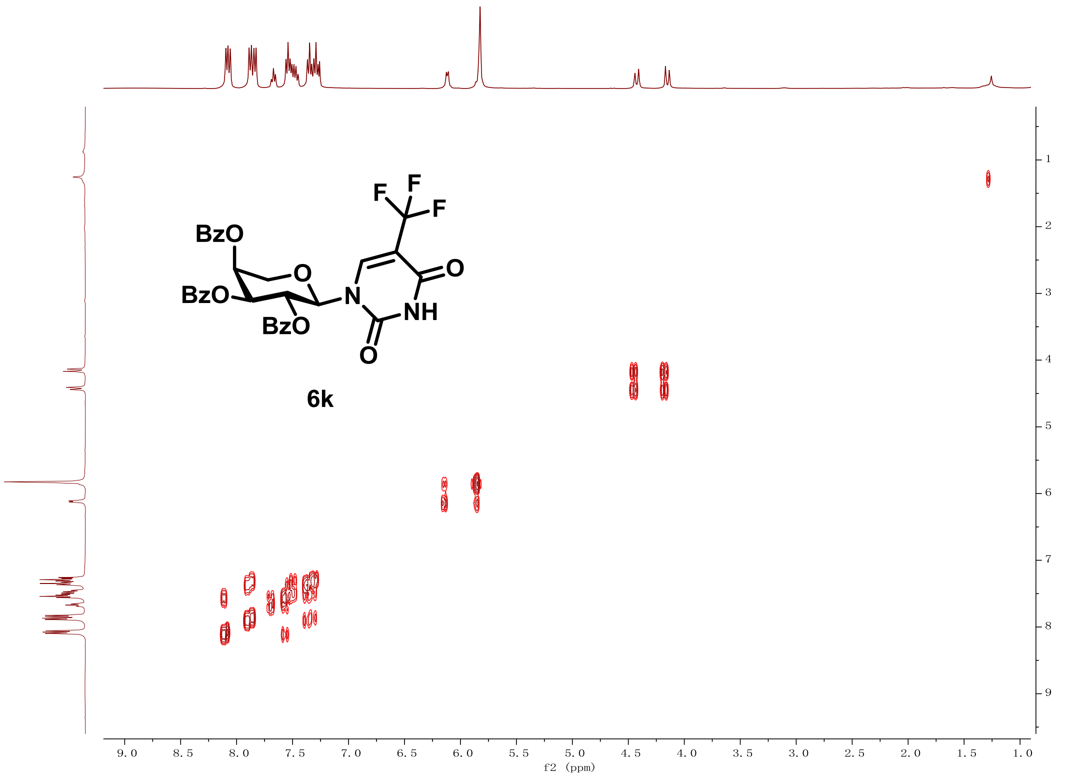

Supplementary Figure 191. COSY NMR Spectrum of Compound **6k**

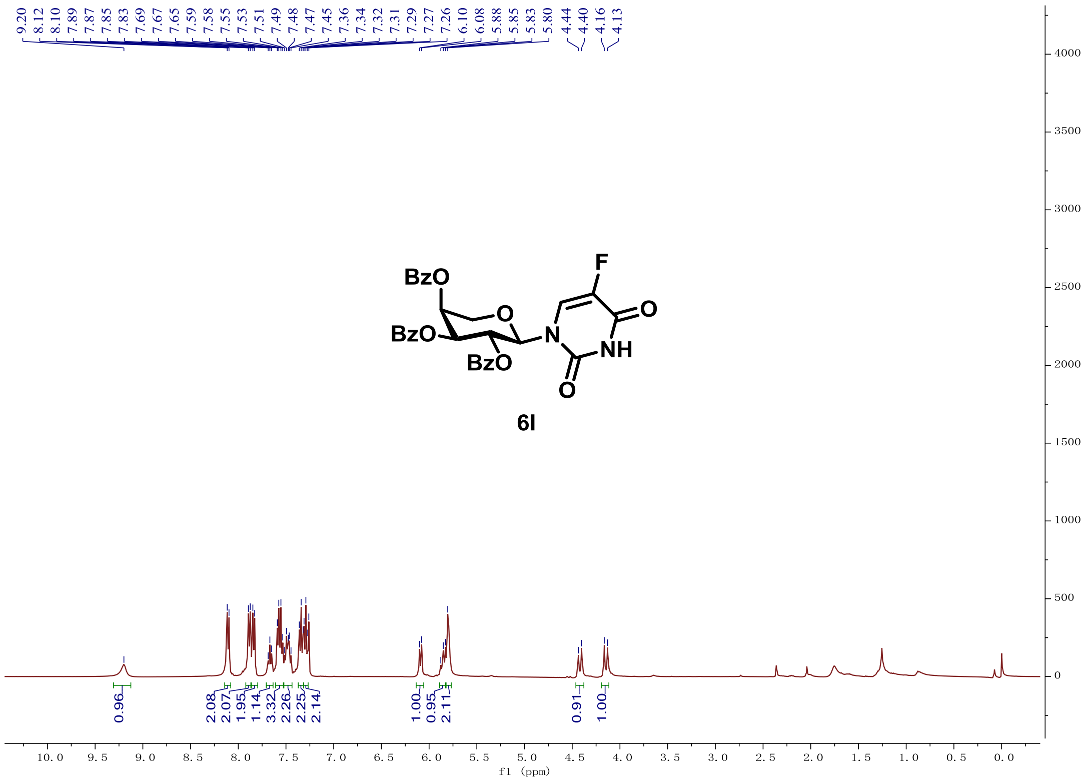

Supplementary Figure 192. ^{1}H NMR Spectrum of Compound **6l**

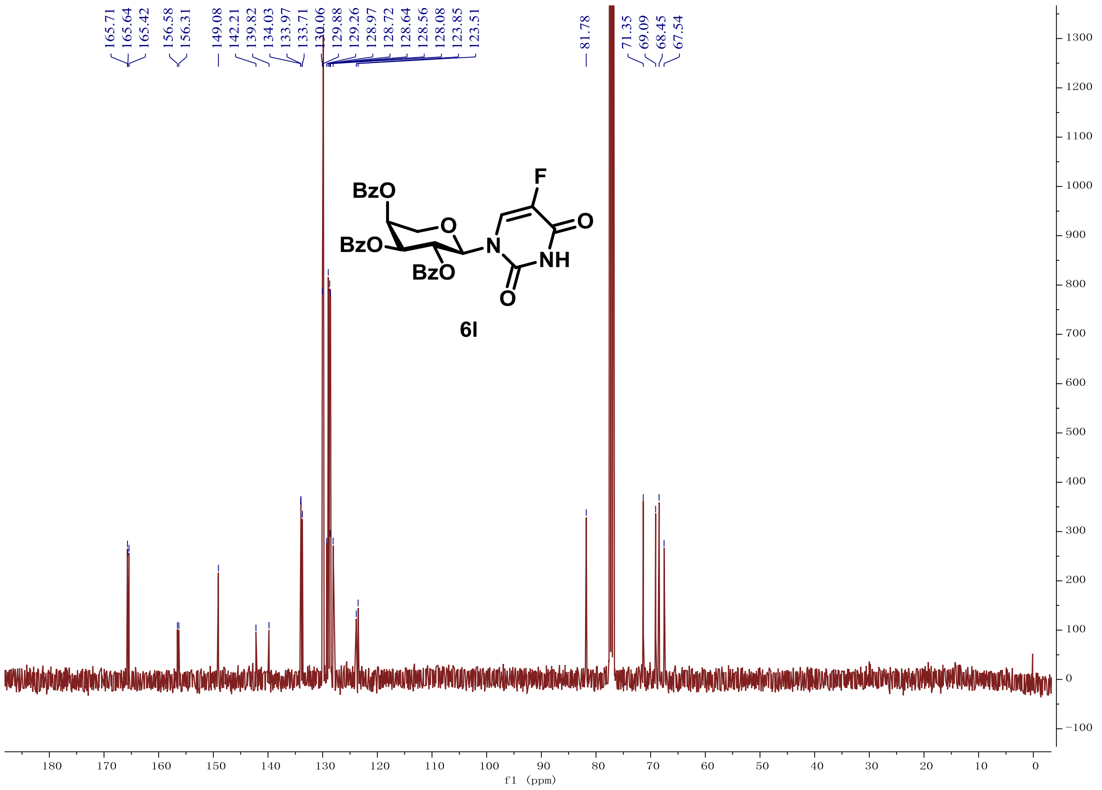

Supplementary Figure 193. ^{13}C NMR Spectrum of Compound **6l**

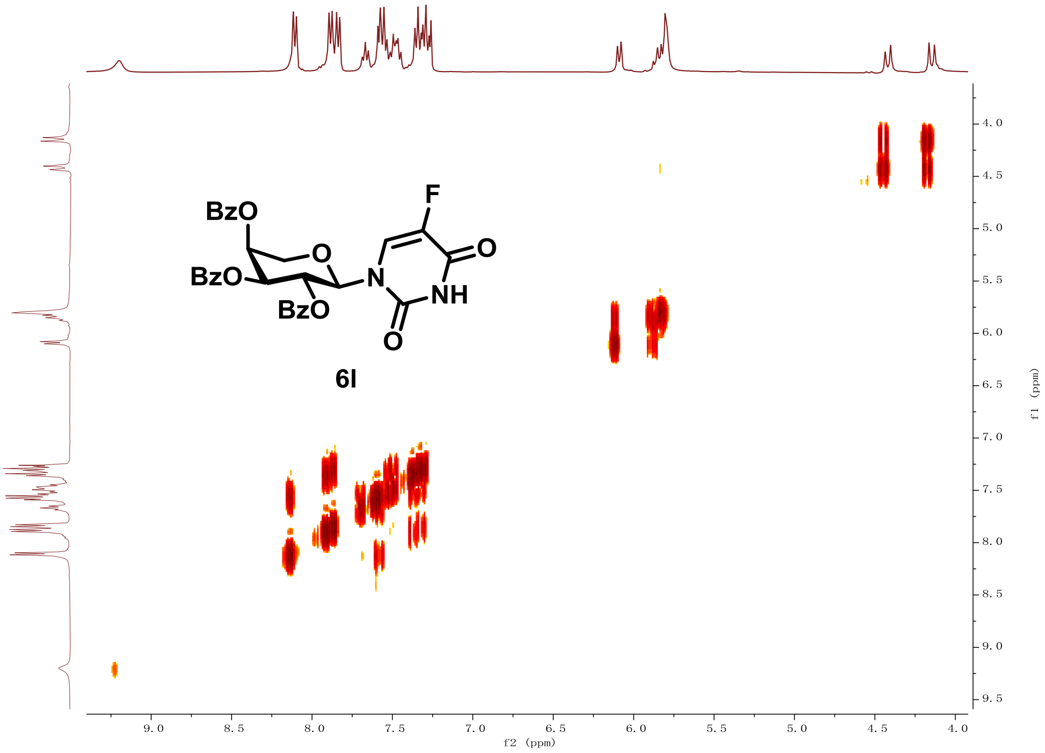

Supplementary Figure 194. COSY NMR Spectrum of Compound **6l**

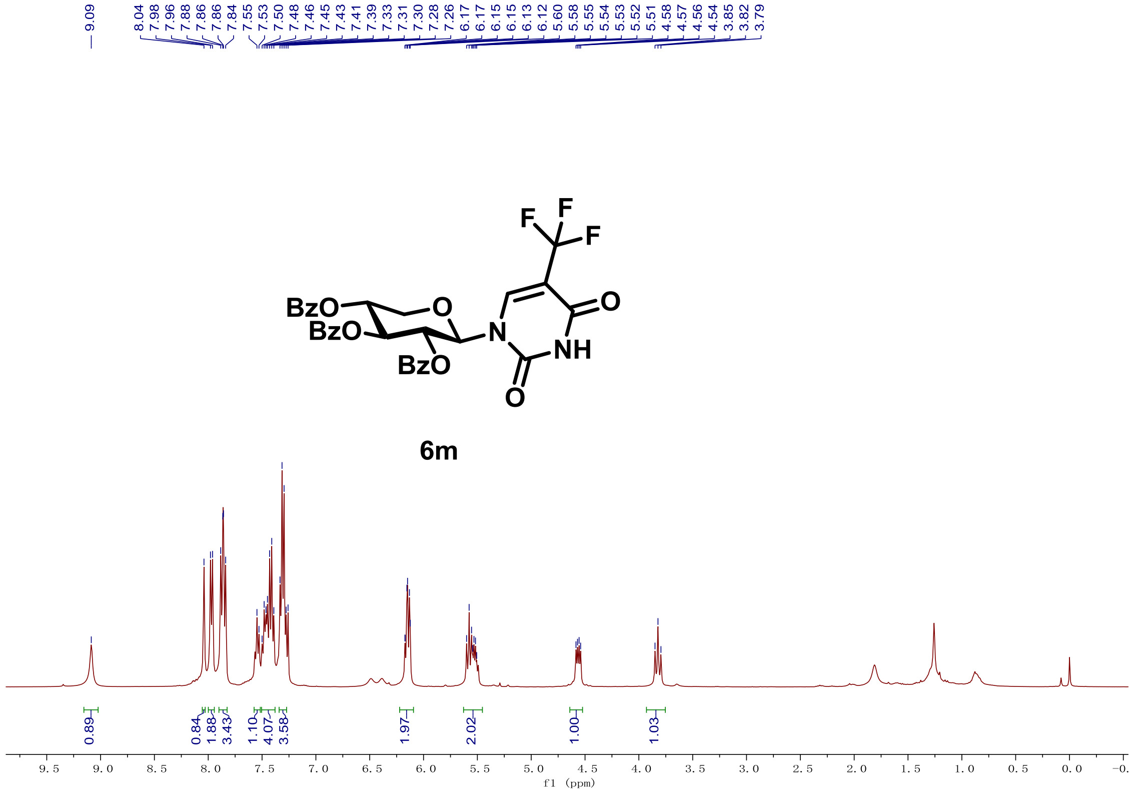

Supplementary Figure 195. ^{1}H NMR Spectrum of Compound **6m**

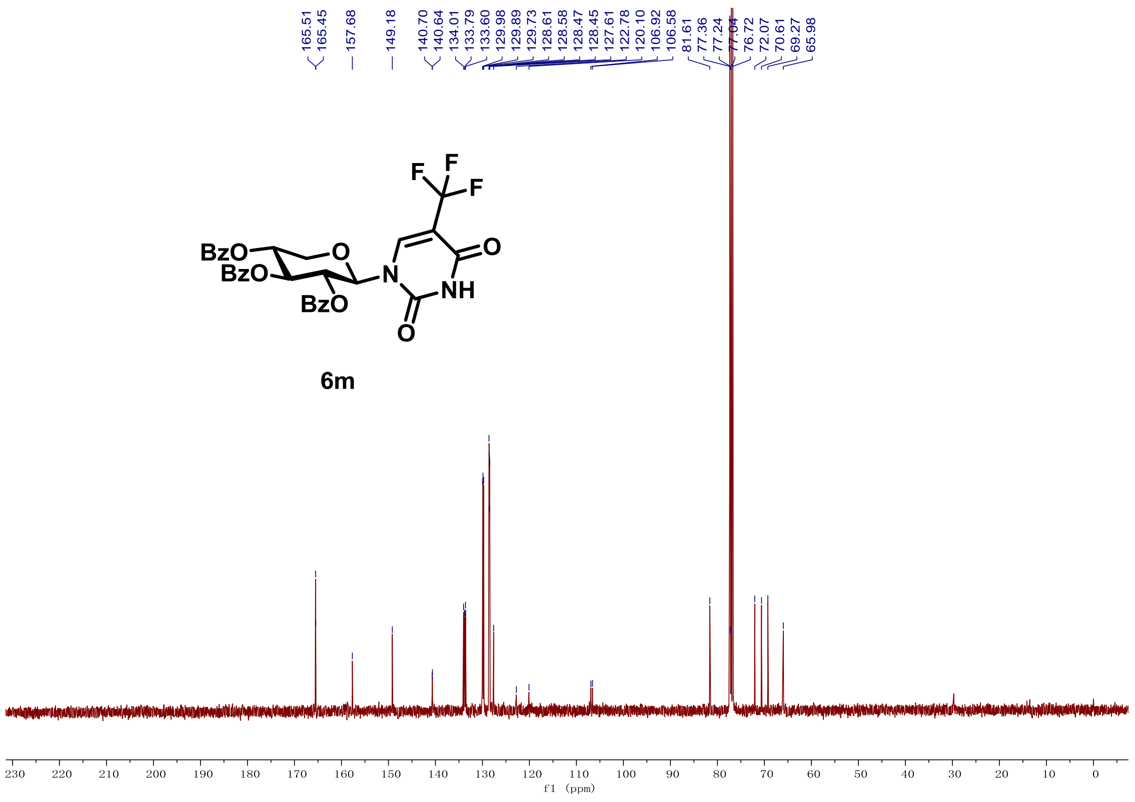

Supplementary Figure 196. ^{13}C NMR Spectrum of Compound **6m**

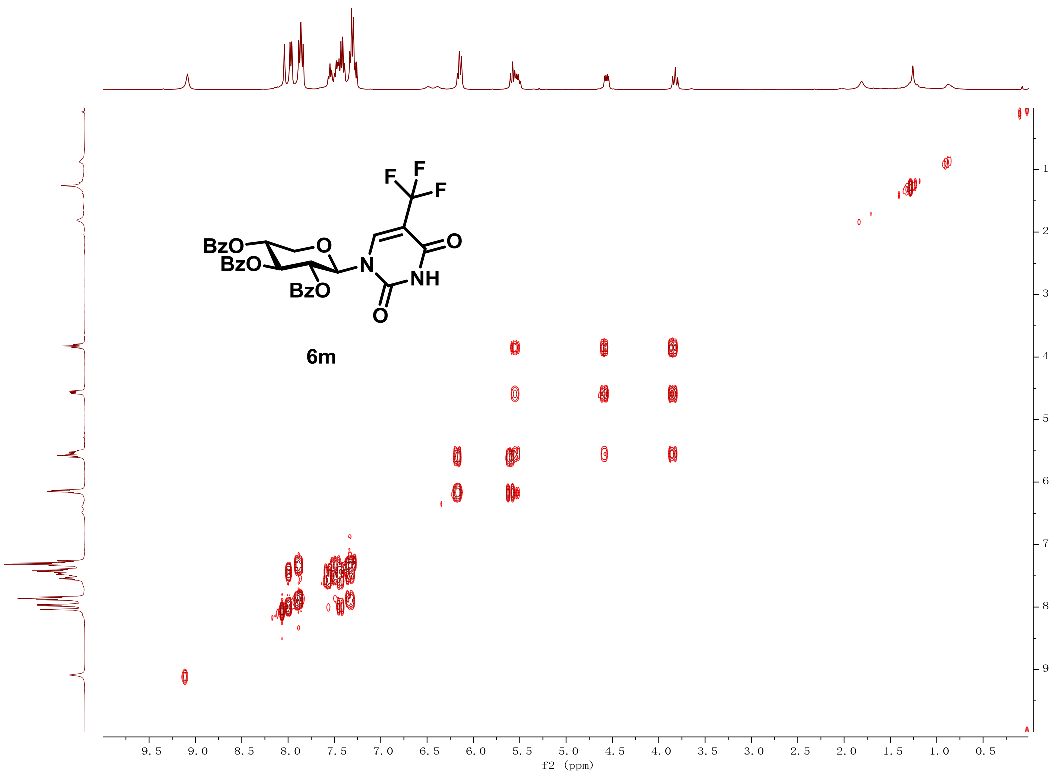

Supplementary Figure 197. COSY NMR Spectrum of Compound **6m**

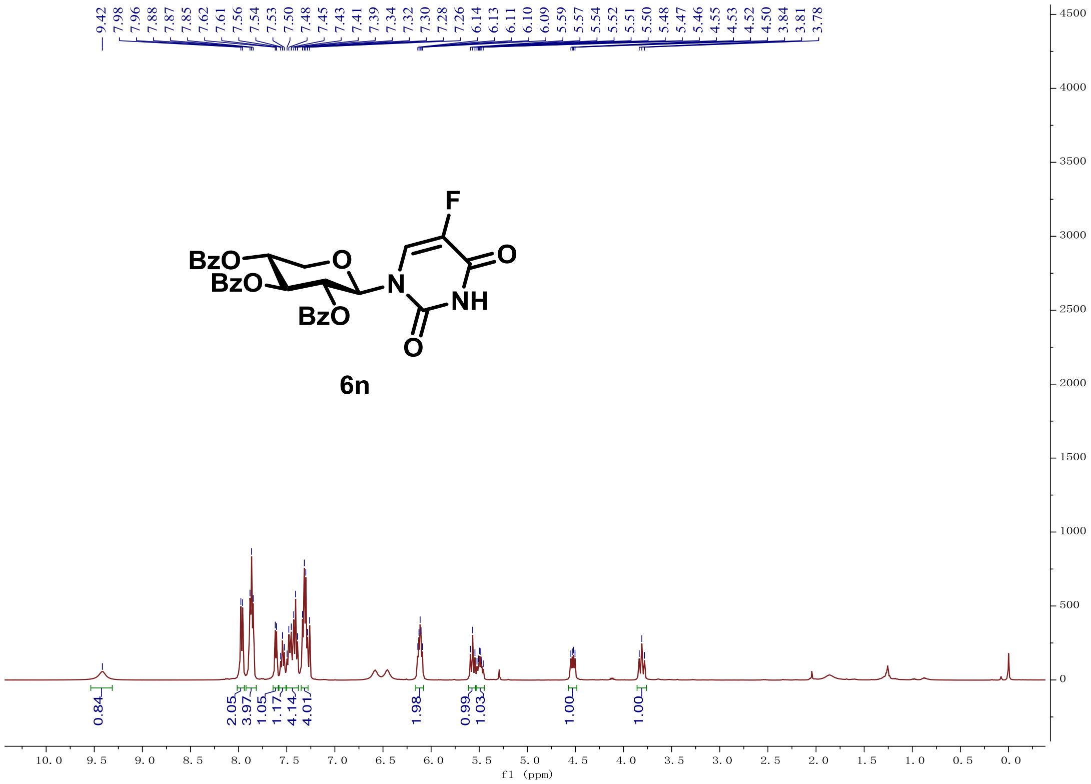

Supplementary Figure 198. ^{1}H NMR Spectrum of Compound **6n**

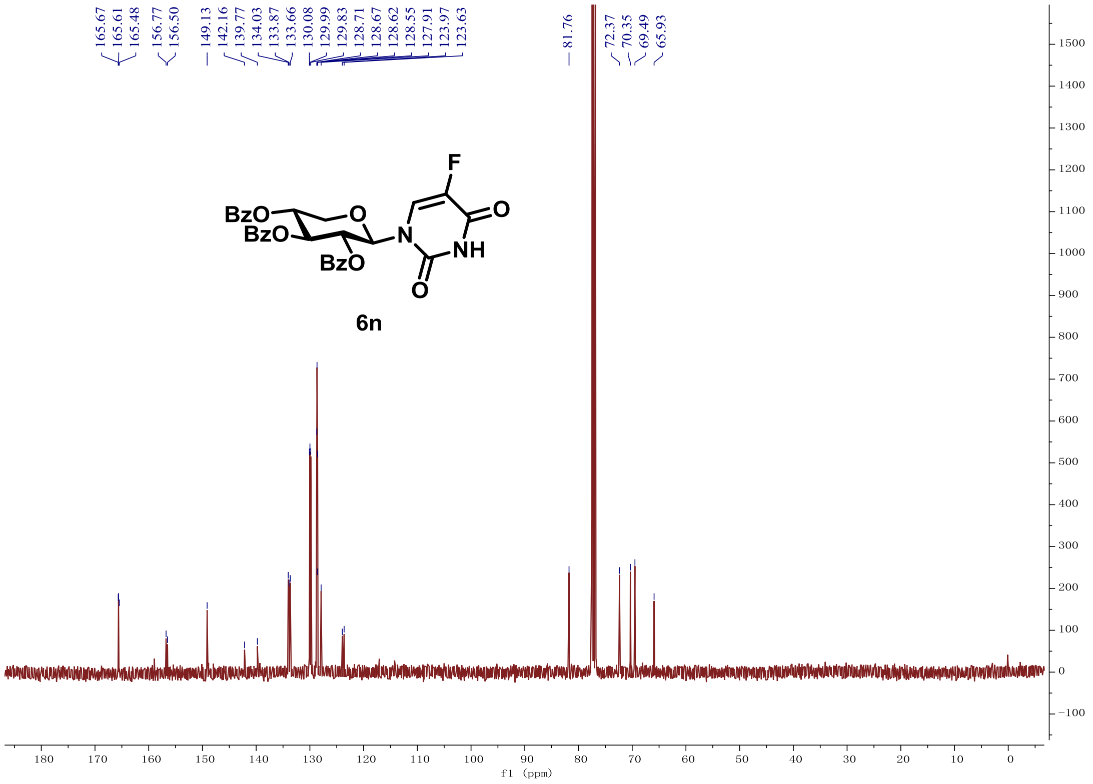

Supplementary Figure 199. ^{13}C NMR Spectrum of Compound **6n**

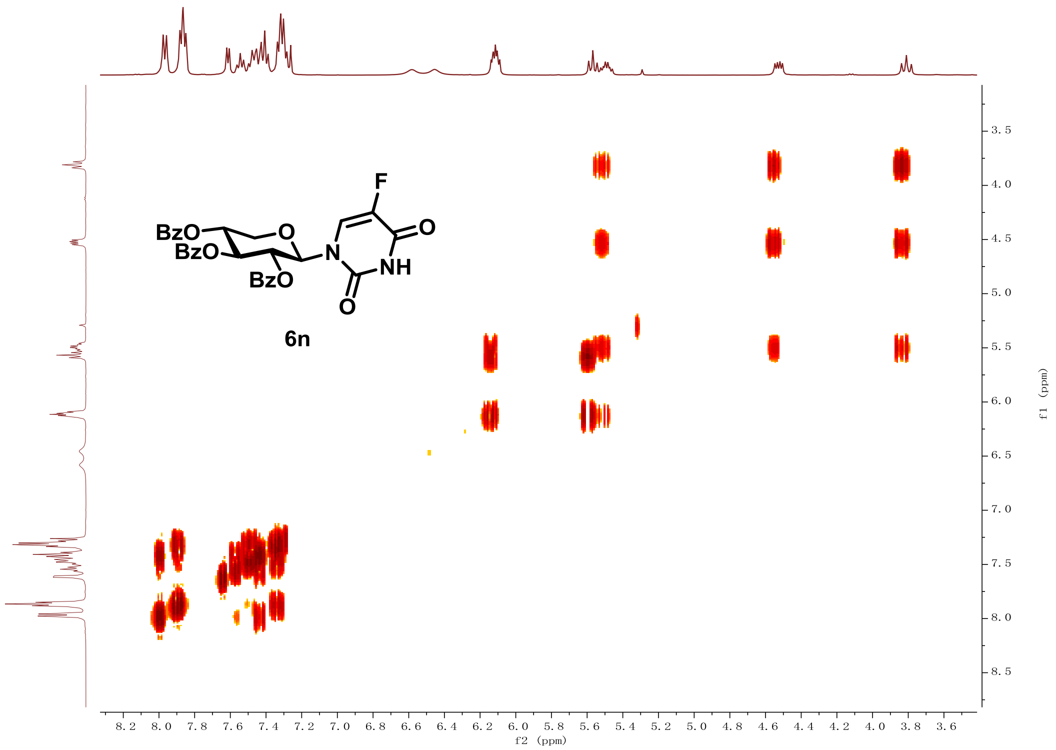

Supplementary Figure 200. COSY NMR Spectrum of Compound **6n**

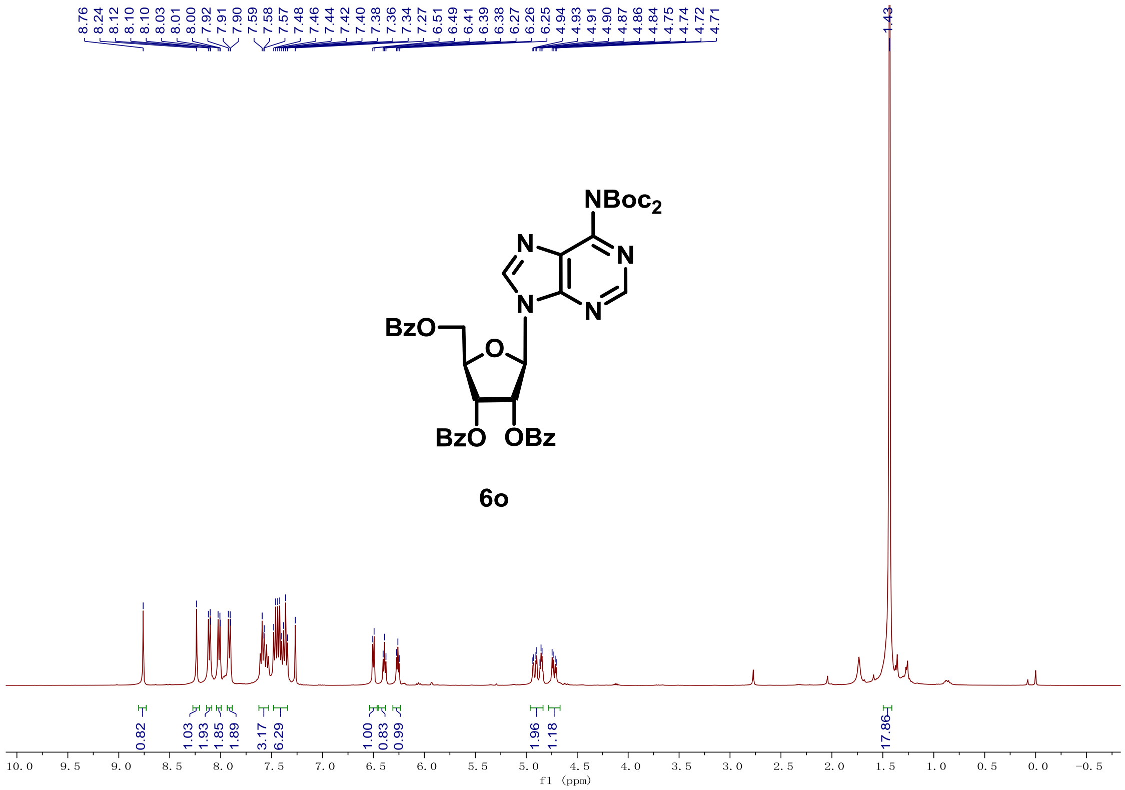

Supplementary Figure 201. ^{1}H NMR Spectrum of Compound **6o**

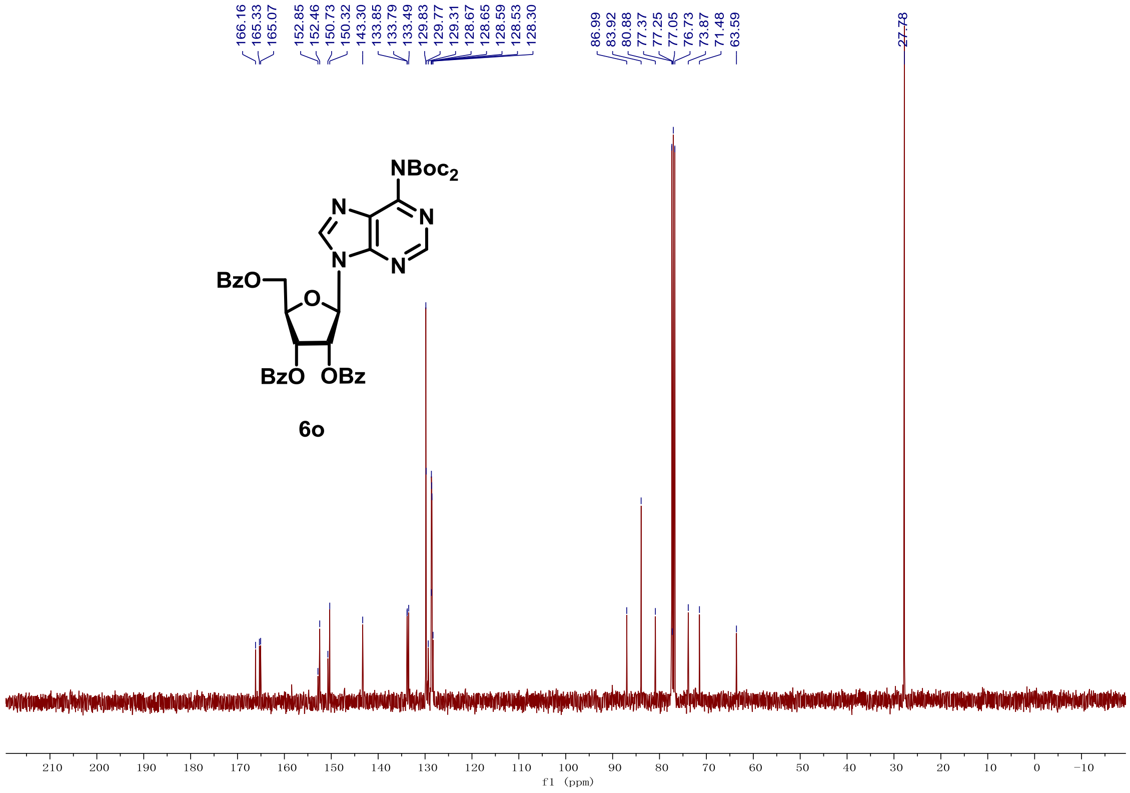

Supplementary Figure 202. ^{13}C NMR Spectrum of Compound **6o**

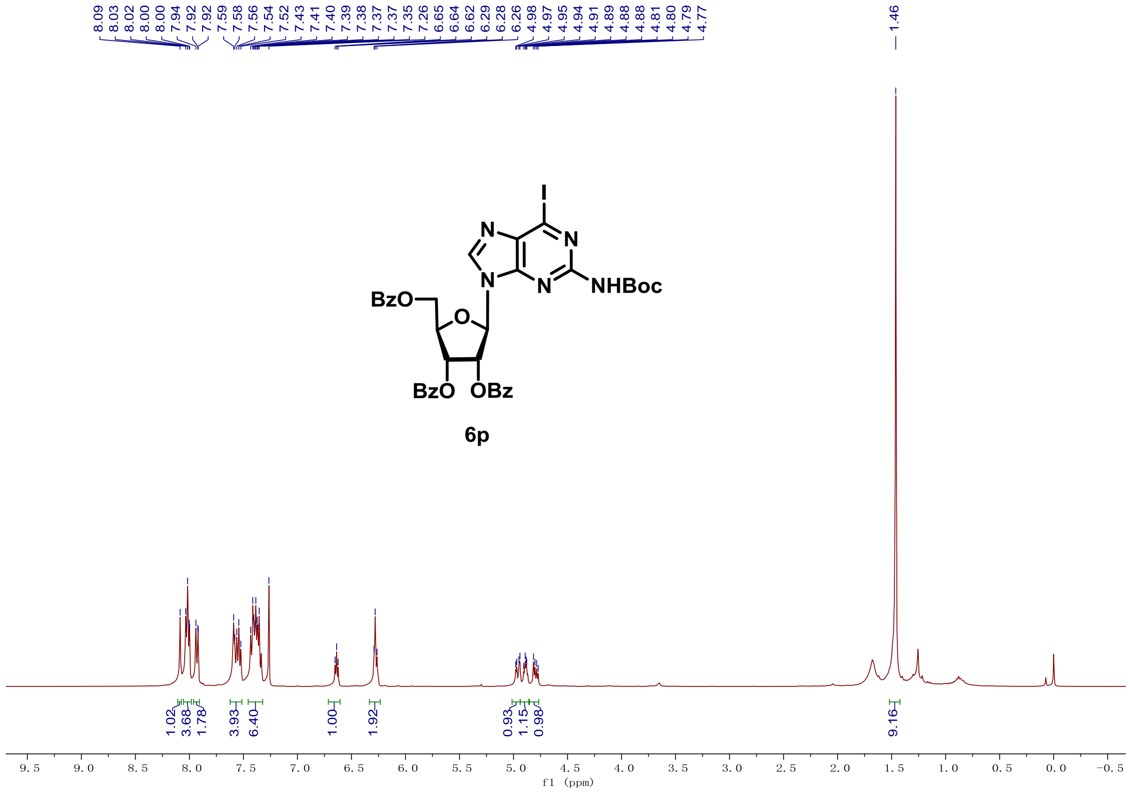

Supplementary Figure 203. ^{1}H NMR Spectrum of Compound **6p**

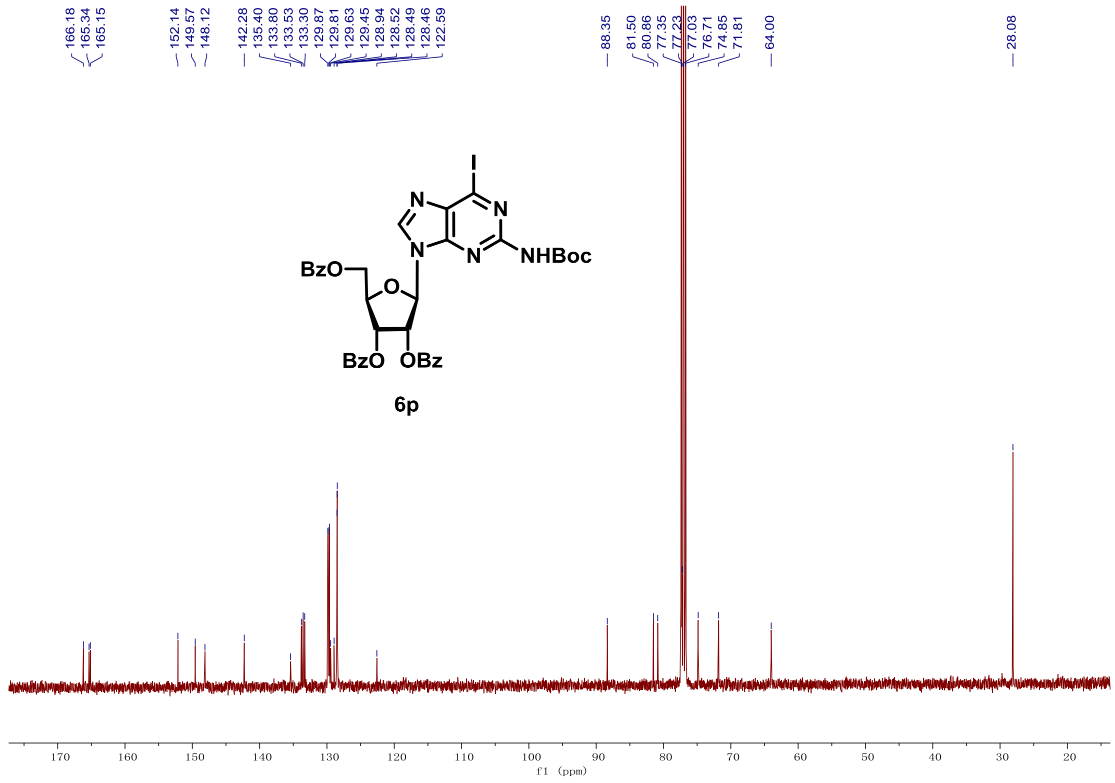

Supplementary Figure 204. ^{13}C NMR Spectrum of Compound **6p**

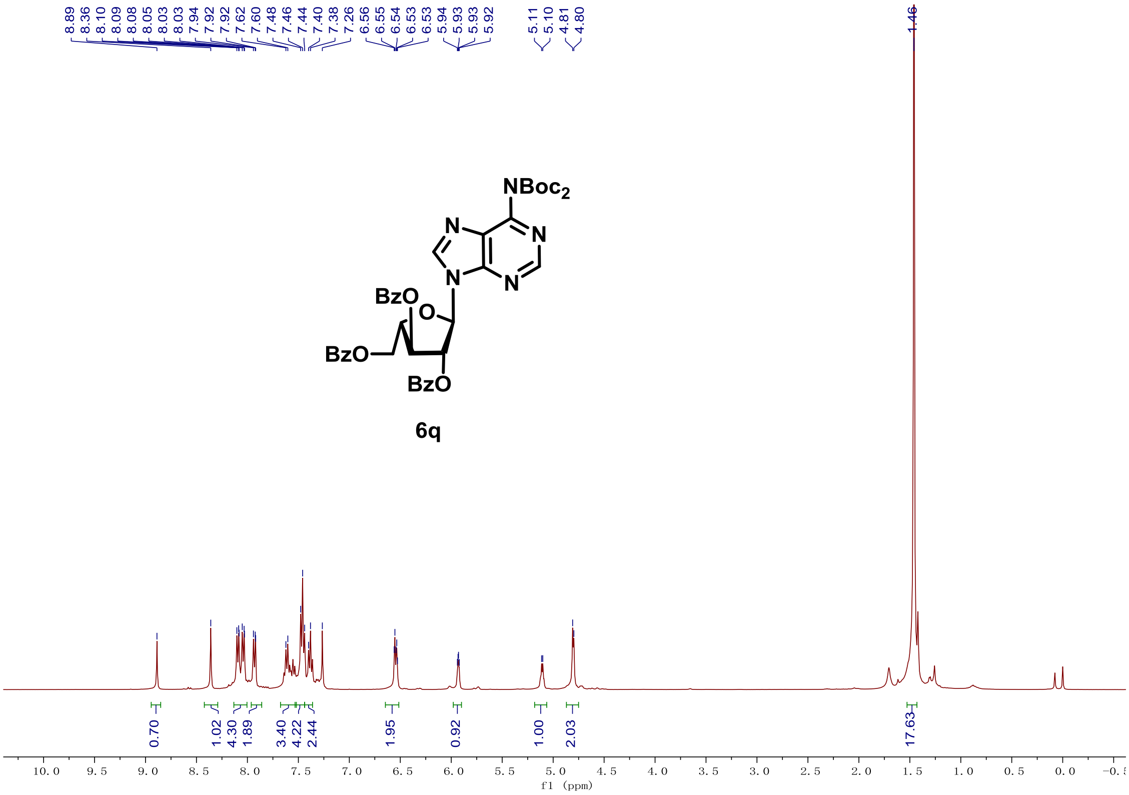

Supplementary Figure 205. ^{1}H NMR Spectrum of Compound **6q**

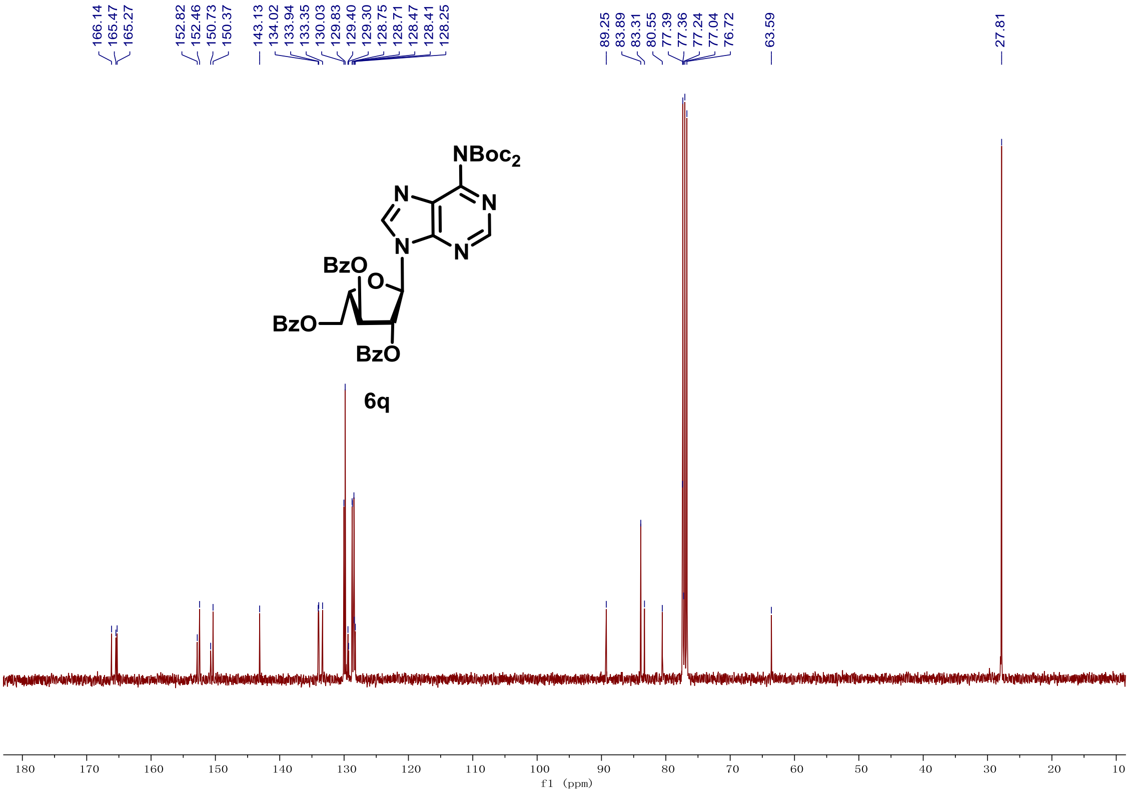

Supplementary Figure 206. ^{13}C NMR Spectrum of Compound **6q**

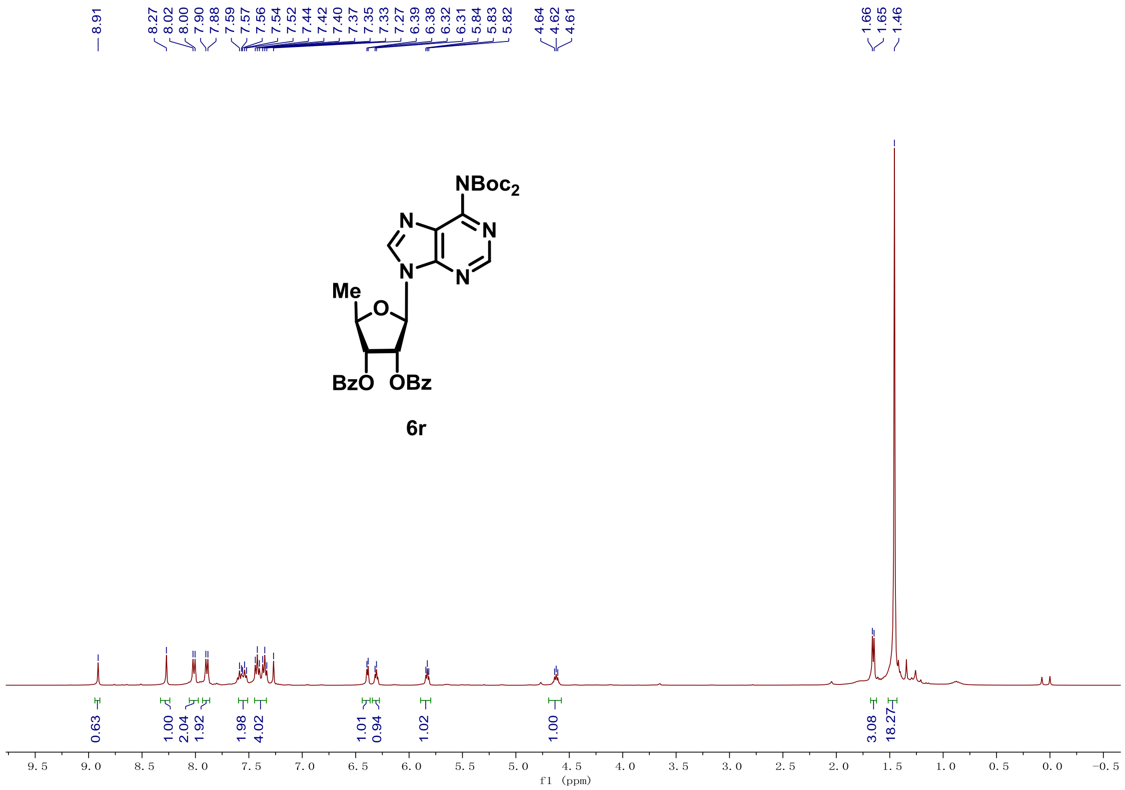

Supplementary Figure 207. ^{1}H NMR Spectrum of Compound **6r**

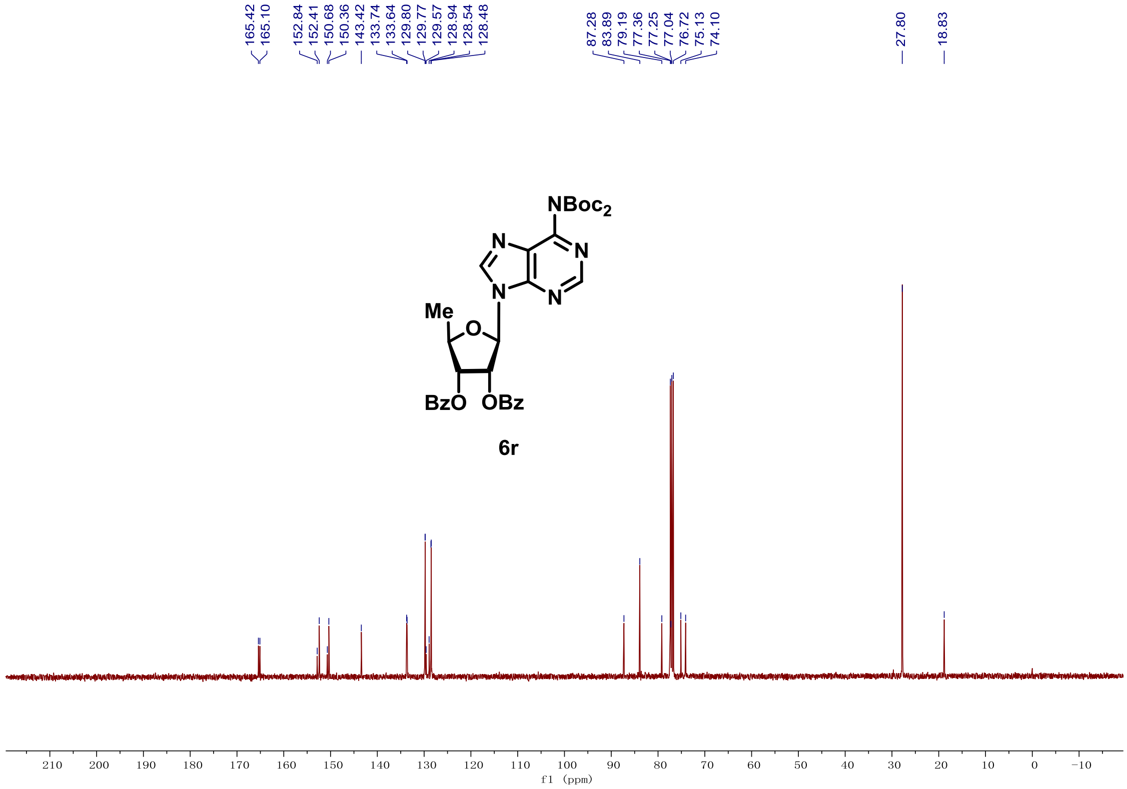

Supplementary Figure 208. ^{13}C NMR Spectrum of Compound **6r**

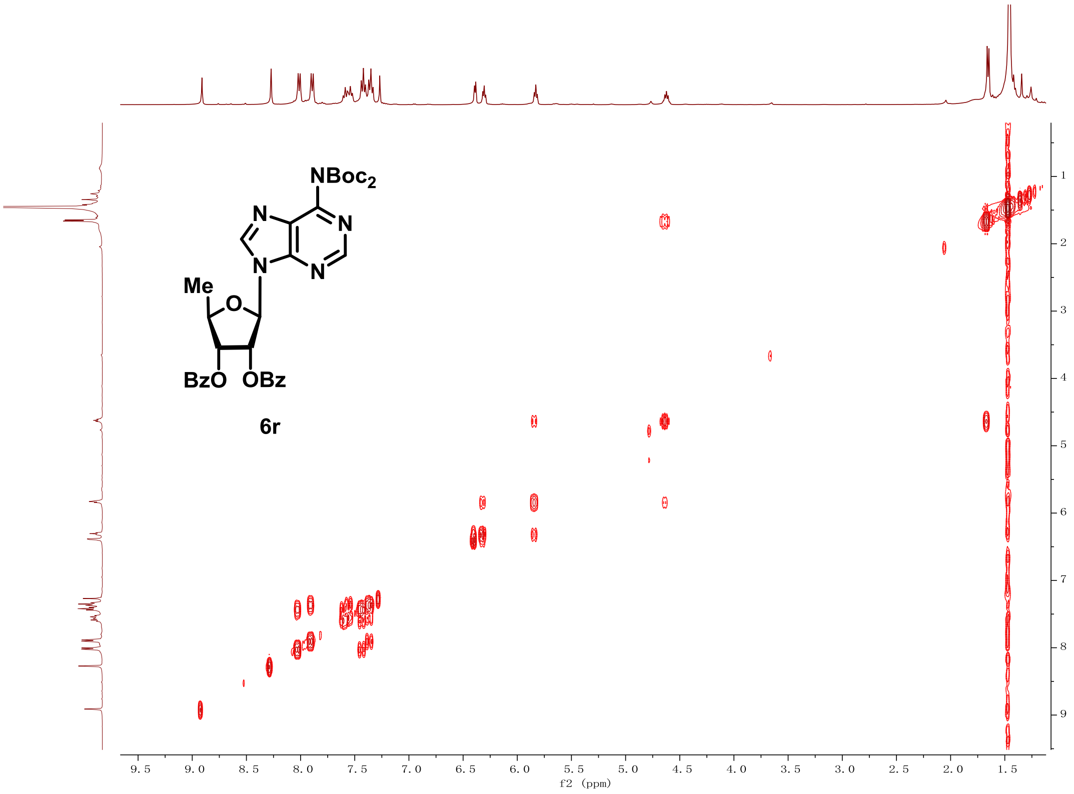

Supplementary Figure 209. COSY NMR Spectrum of Compound **6r**

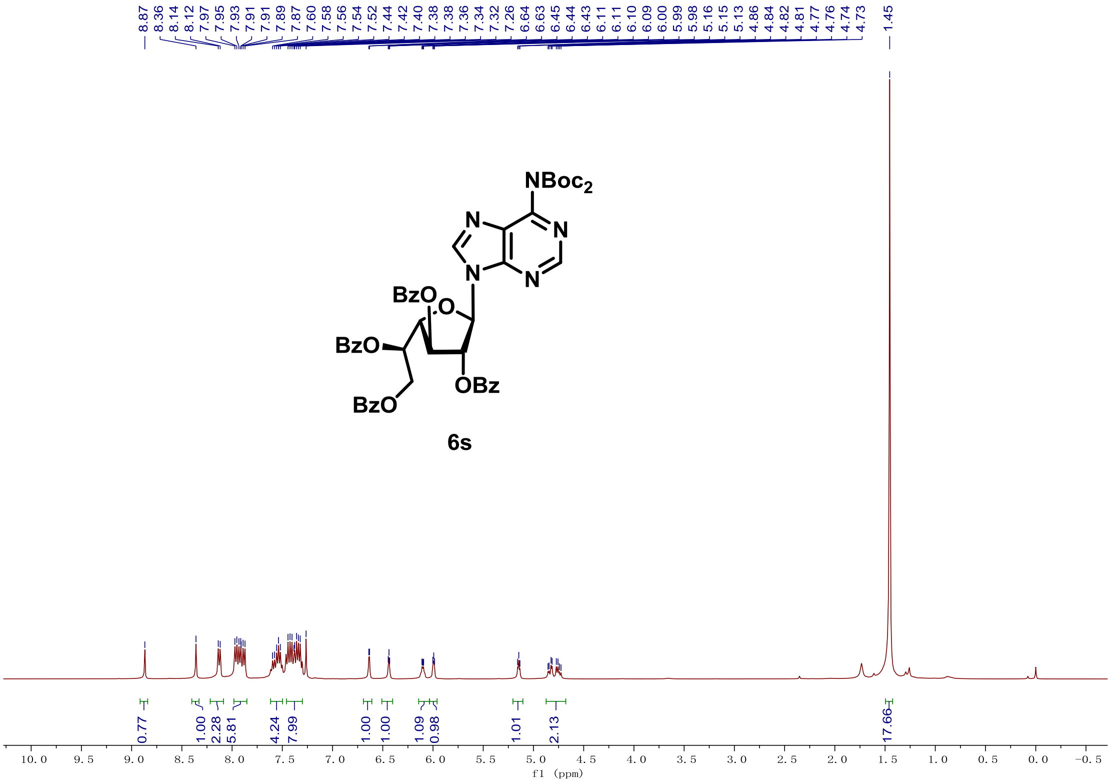

Supplementary Figure 210. ^{1}H NMR Spectrum of Compound **6s**

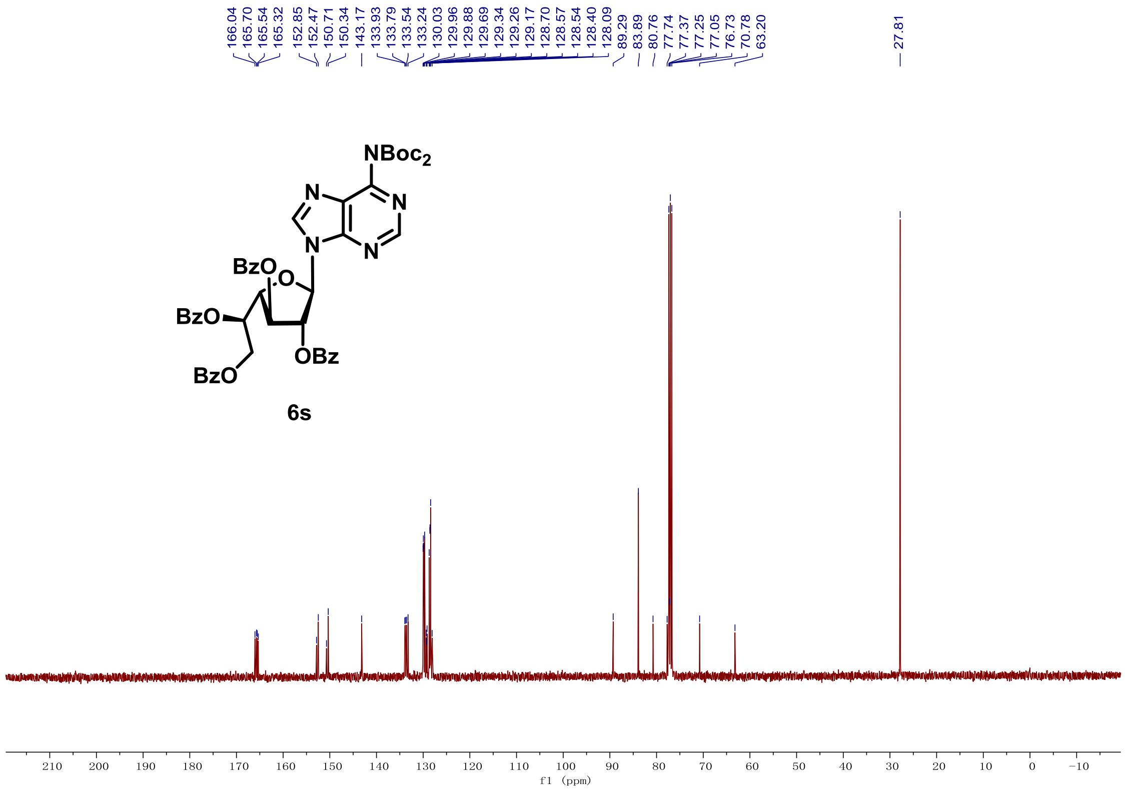

Supplementary Figure 211. ^{13}C NMR Spectrum of Compound **6s**

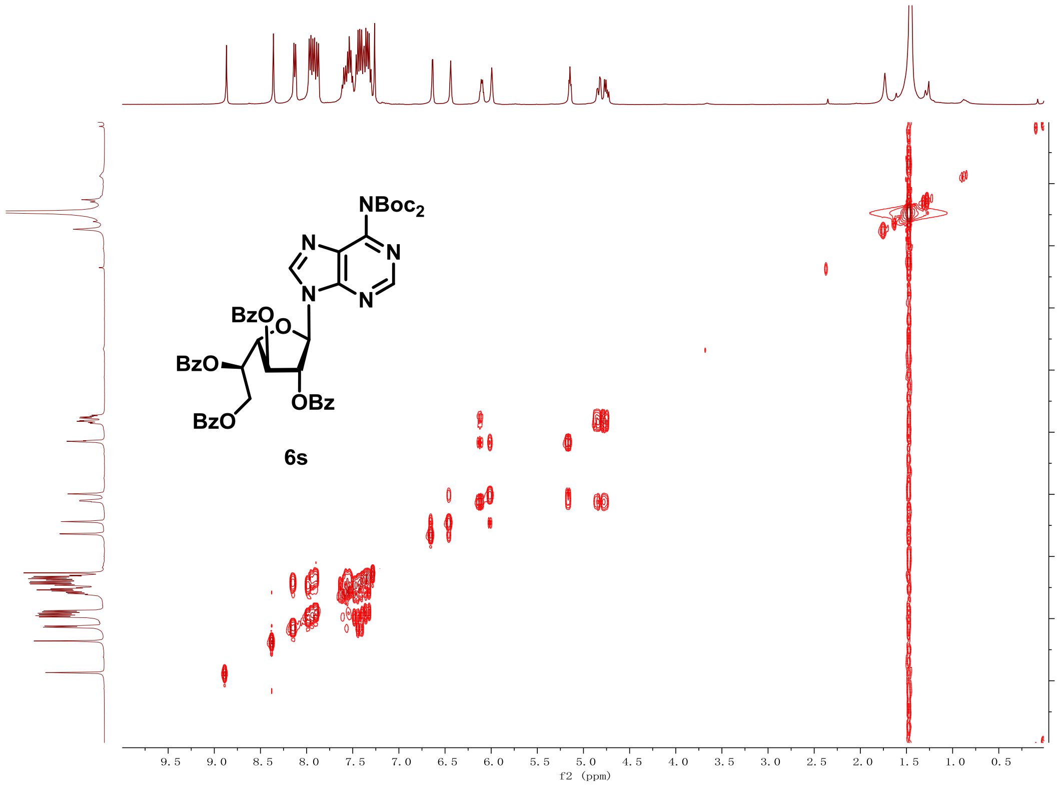

Supplementary Figure 212. COSY NMR Spectrum of Compound **6s**

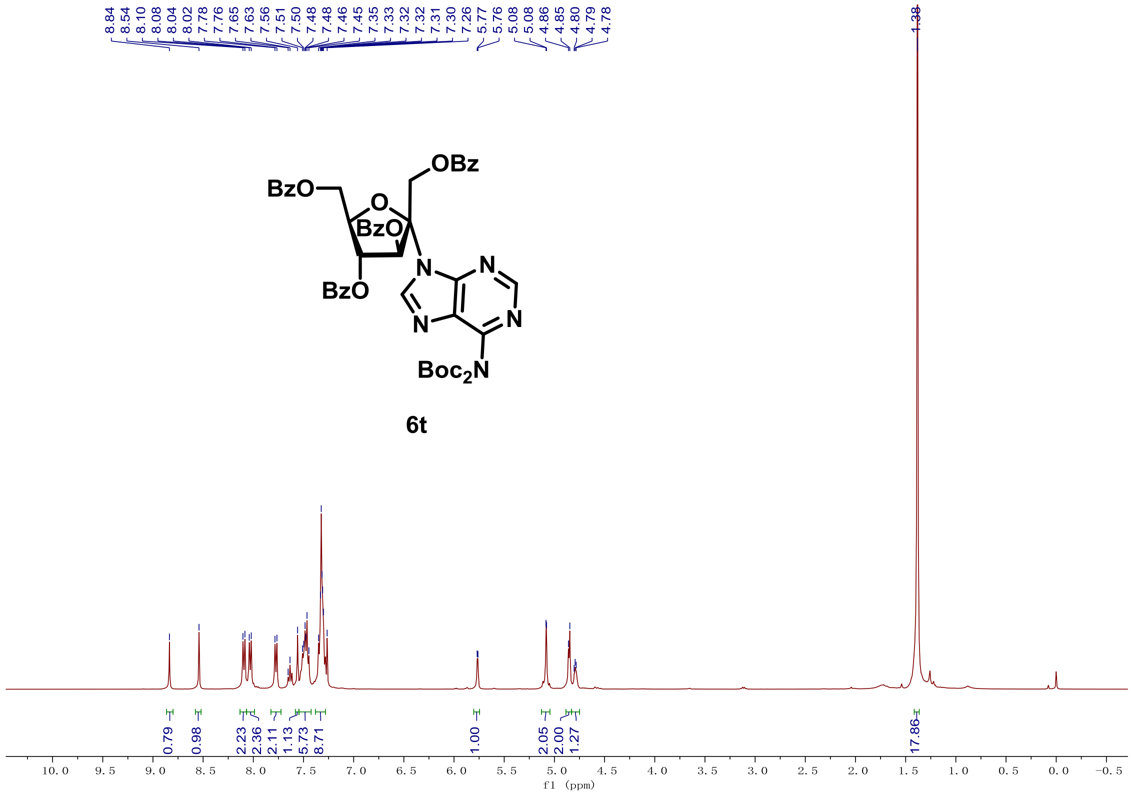

Supplementary Figure 213. ^{1}H NMR Spectrum of Compound **6t**

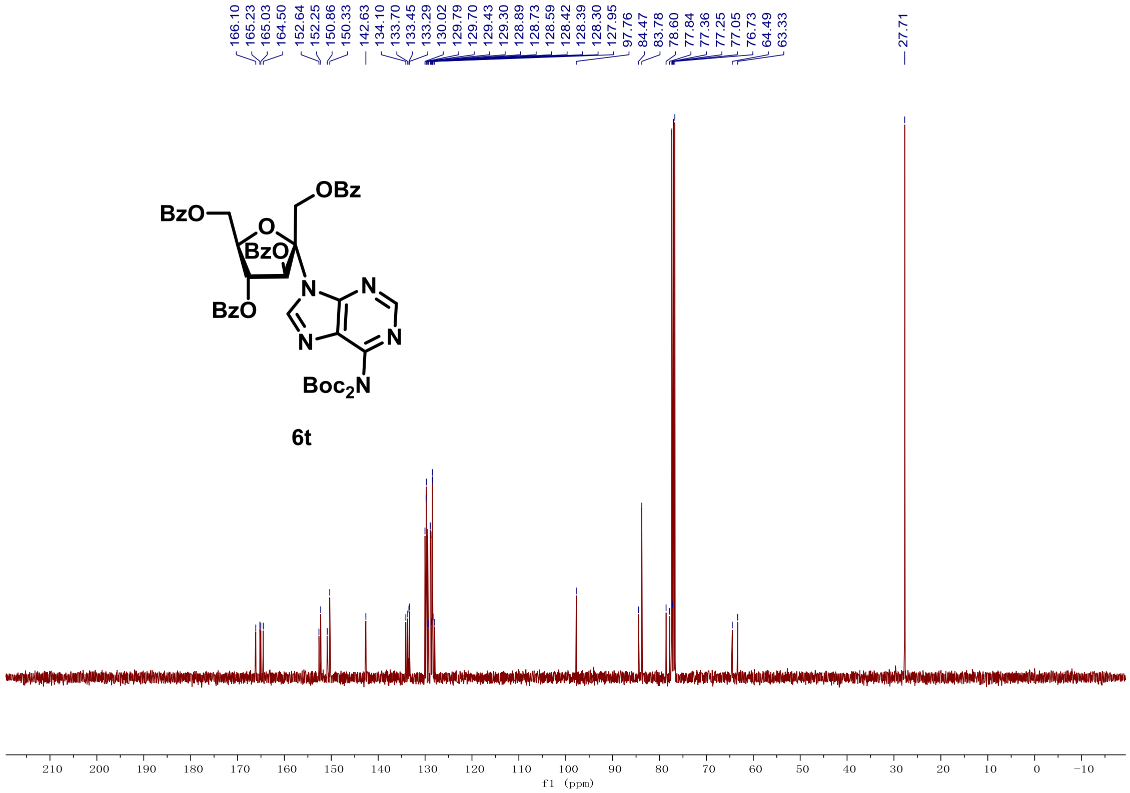

Supplementary Figure 214. ^{13}C NMR Spectrum of Compound **6t**

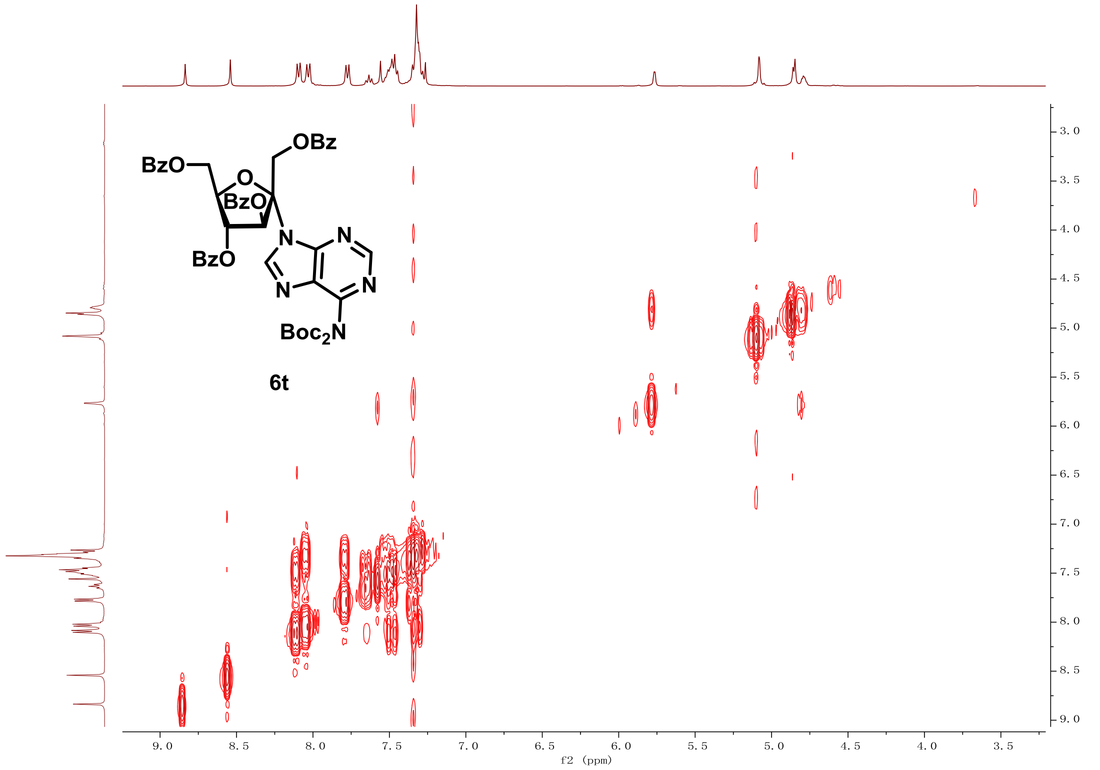

Supplementary Figure 215. COSY NMR Spectrum of Compound **6t**

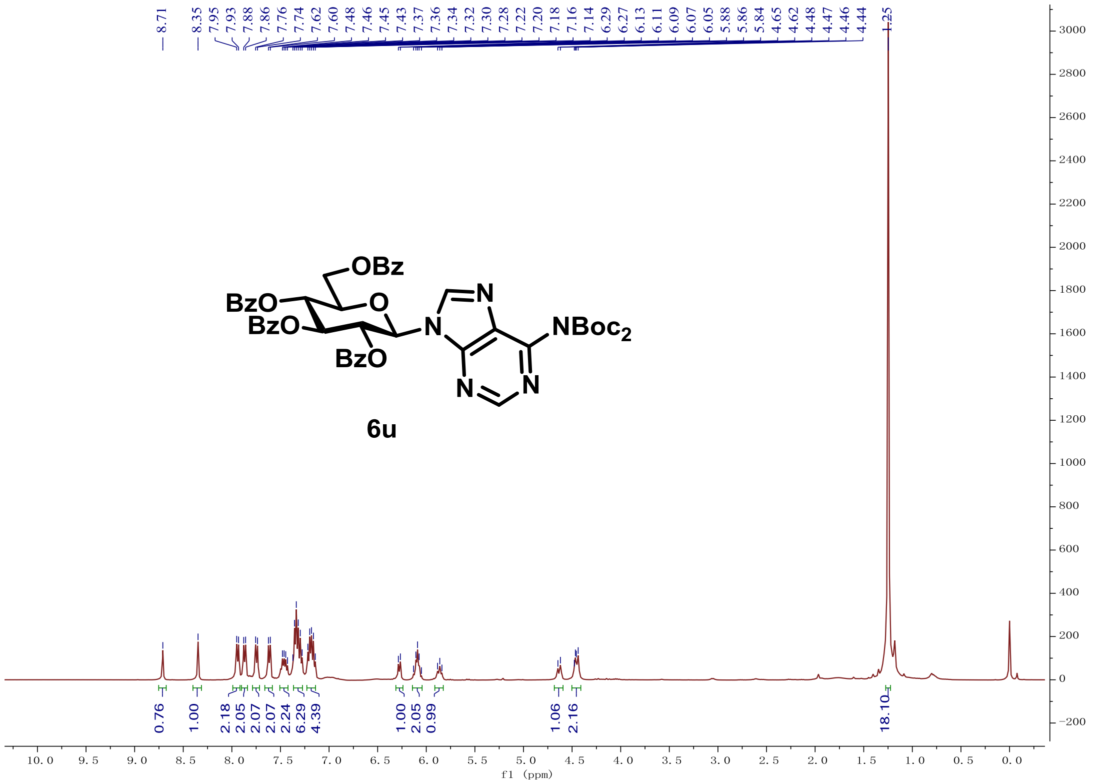

Supplementary Figure 216. ^{1}H NMR Spectrum of Compound **6u**

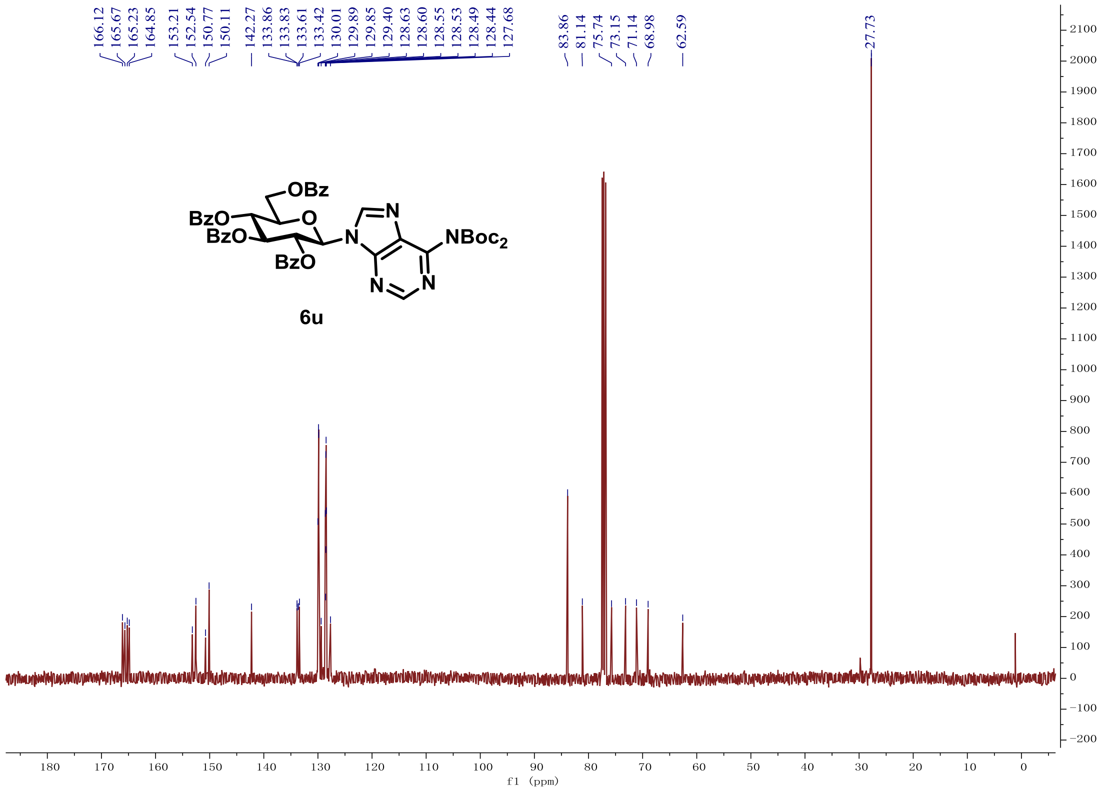

Supplementary Figure 217. ^{13}C NMR Spectrum of Compound **6u**

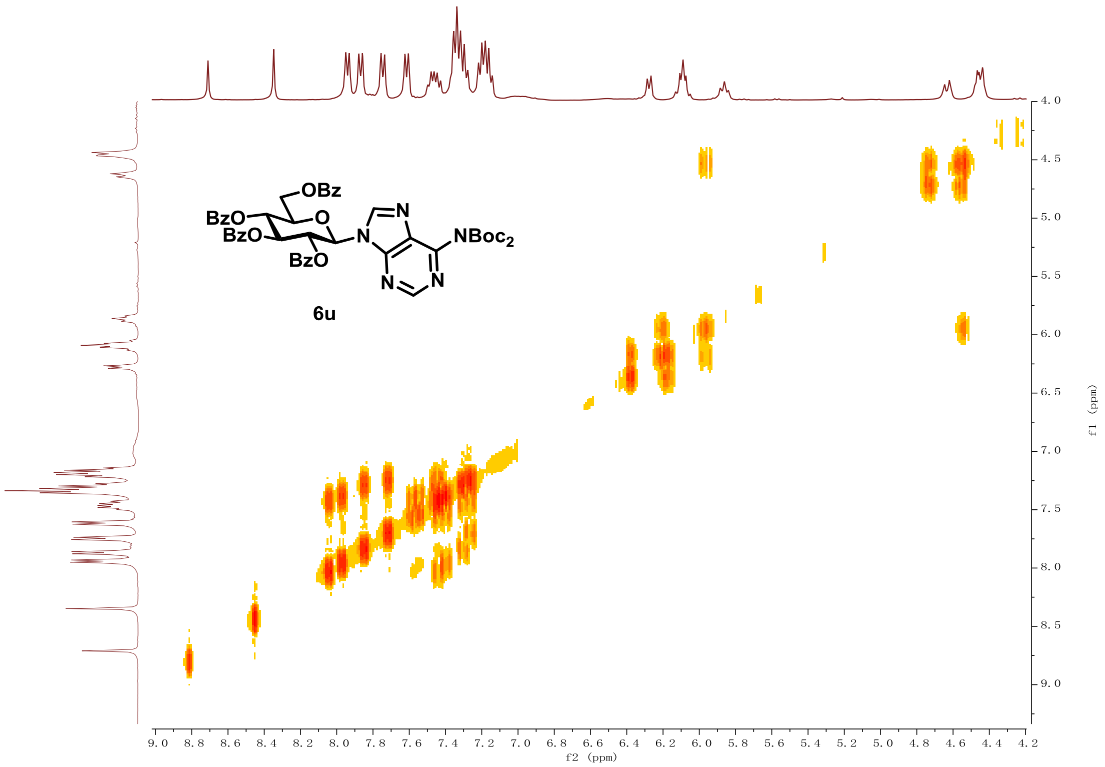

Supplementary Figure 218. COSY NMR Spectrum of Compound **6u**

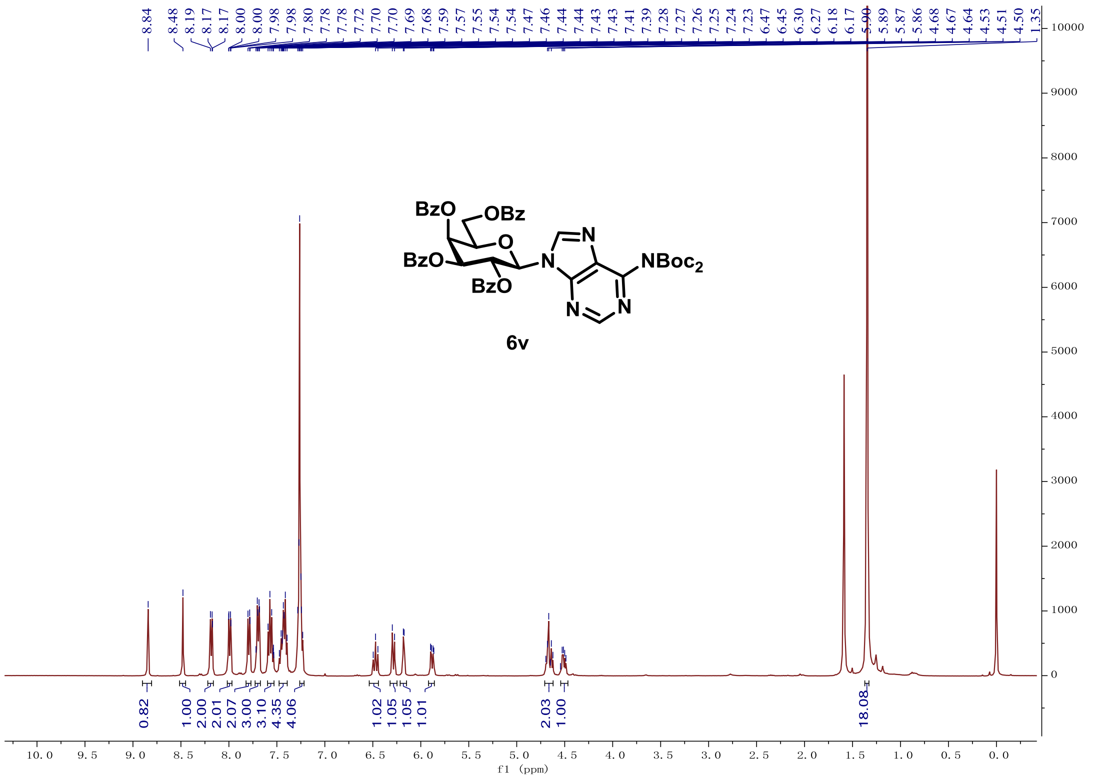

Supplementary Figure 219. ^{1}H NMR Spectrum of Compound **6v**

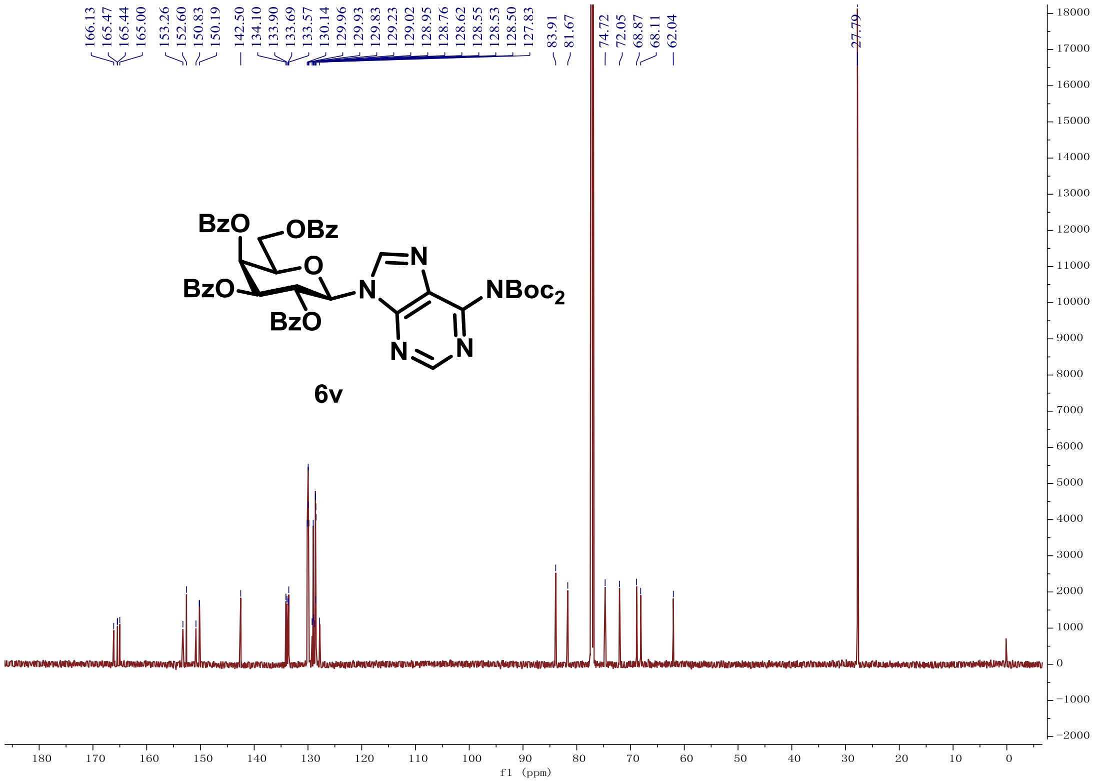

Supplementary Figure 220. ^{13}C NMR Spectrum of Compound **6v**

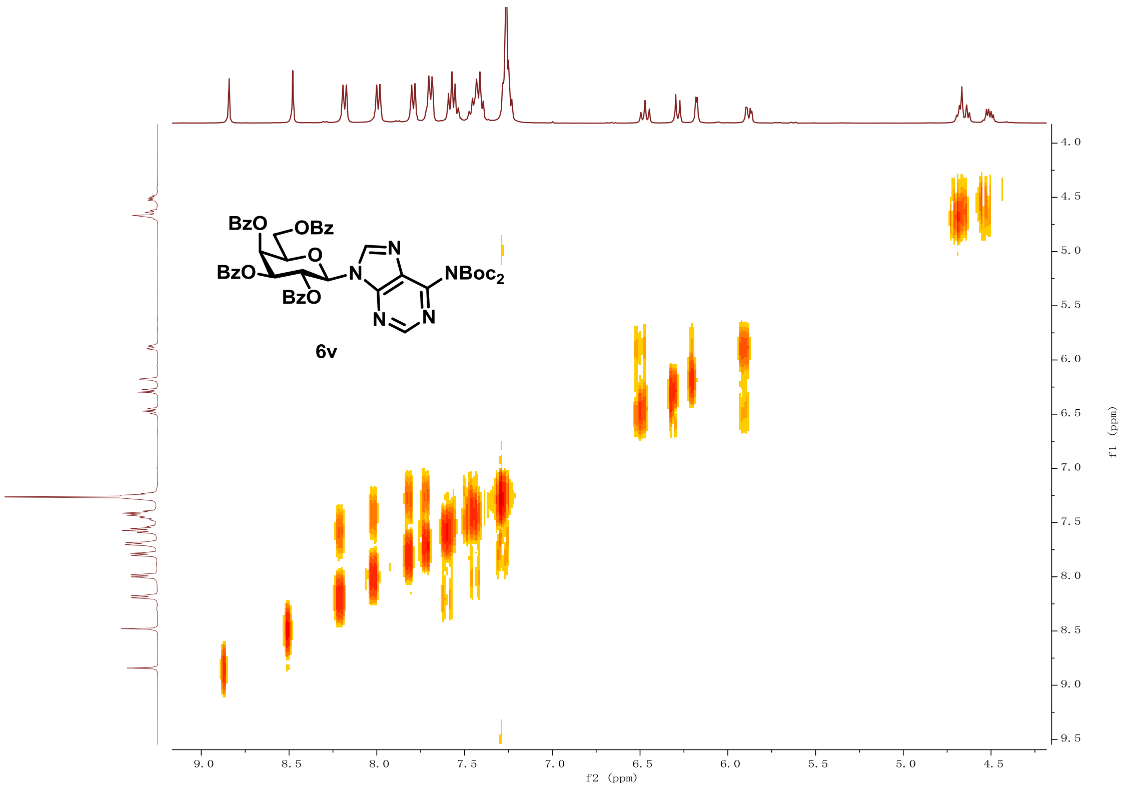

Supplementary Figure 221. COSY NMR Spectrum of Compound **6v**

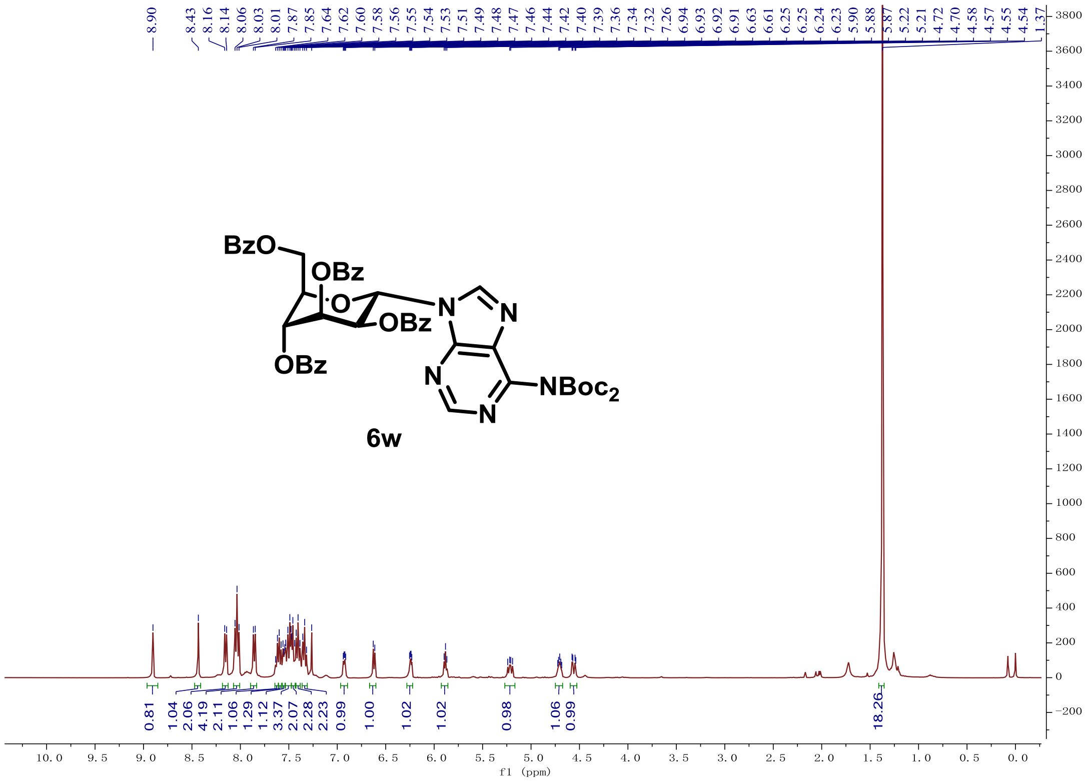

Supplementary Figure 222. ^{1}H NMR Spectrum of Compound **6w**

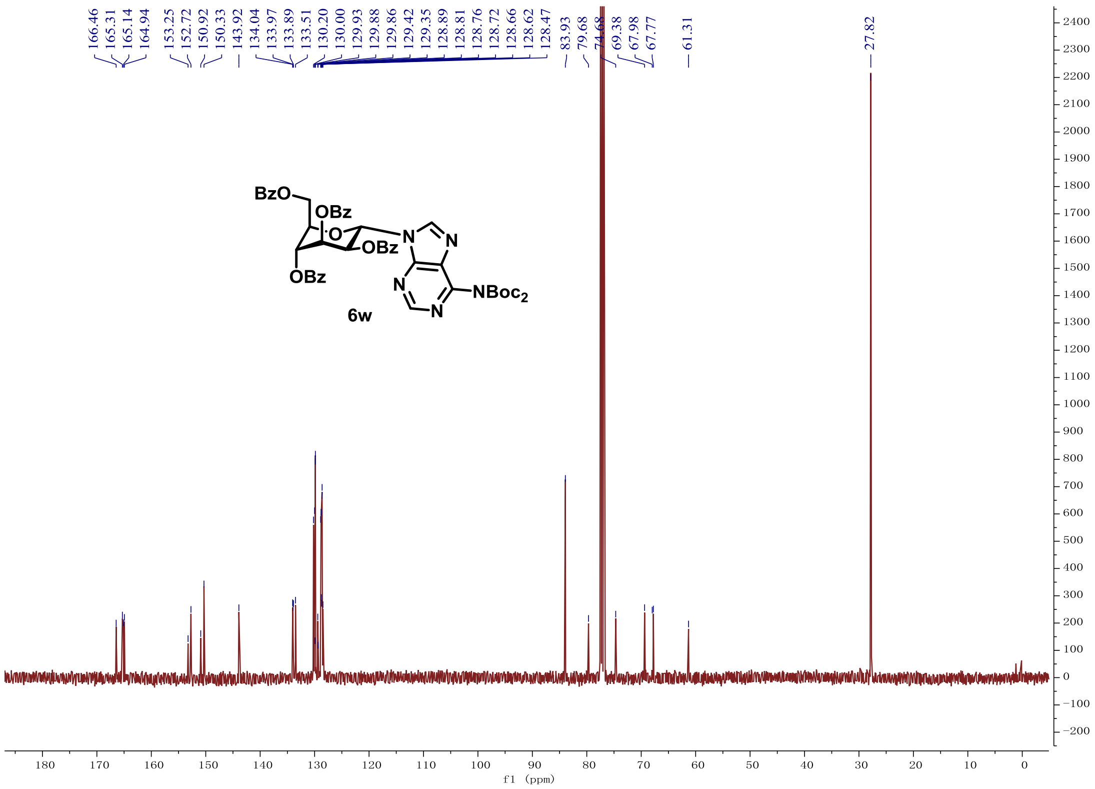

Supplementary Figure 223. ^{13}C NMR Spectrum of Compound **6w**

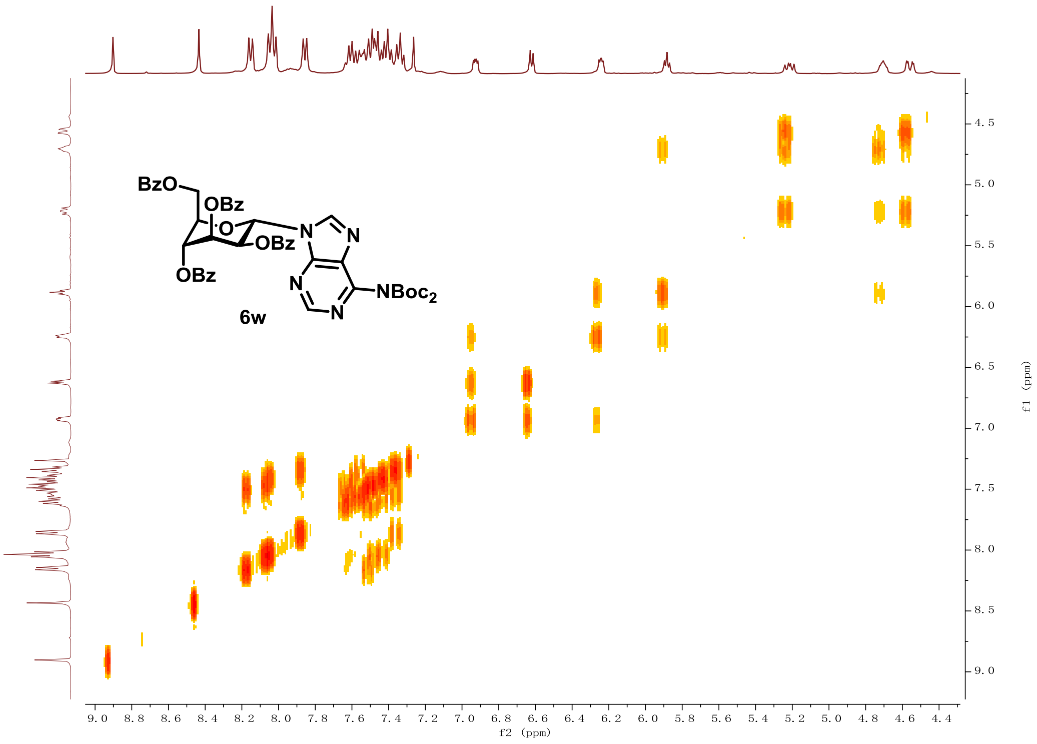

Supplementary Figure 224. COSY NMR Spectrum of Compound **6w**

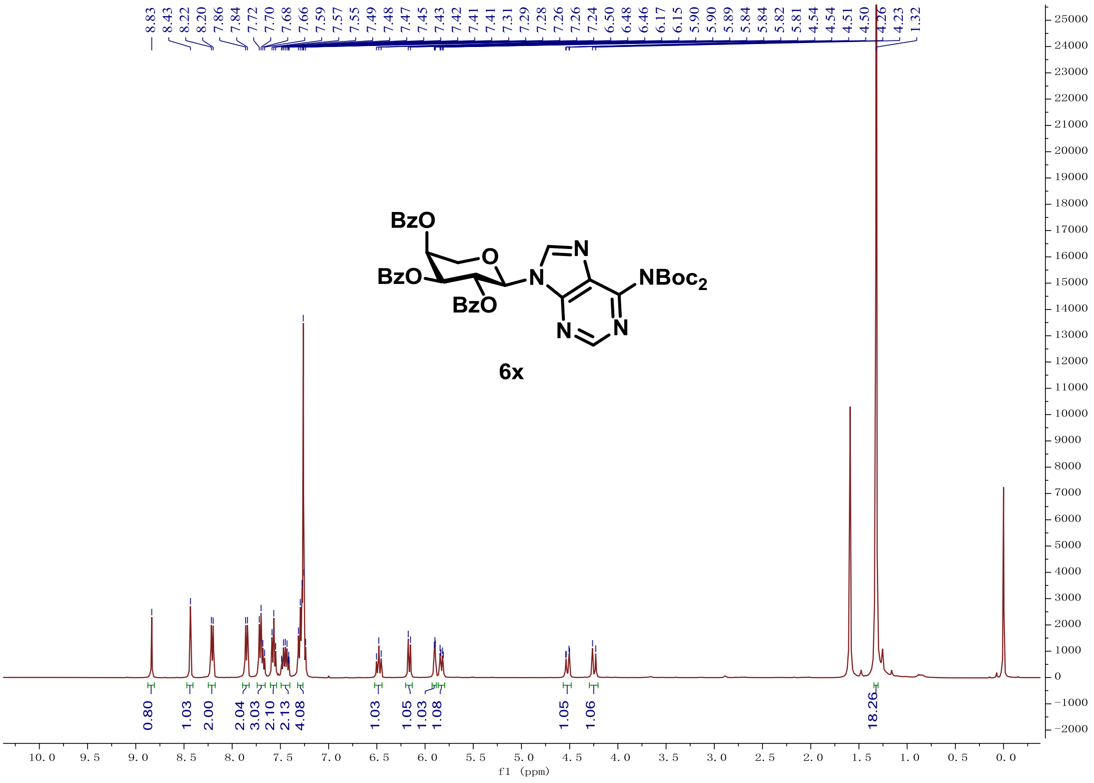

Supplementary Figure 225. ^{1}H NMR Spectrum of Compound **6x**

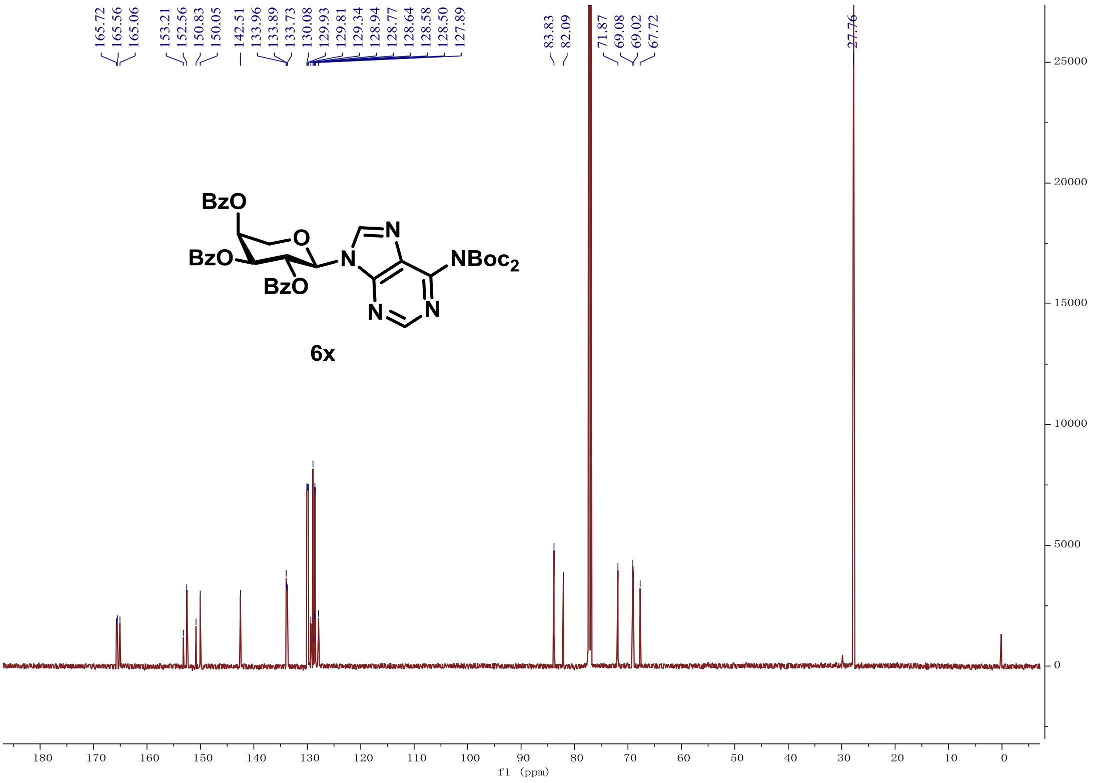

Supplementary Figure 226. ^{13}C NMR Spectrum of Compound **6x**

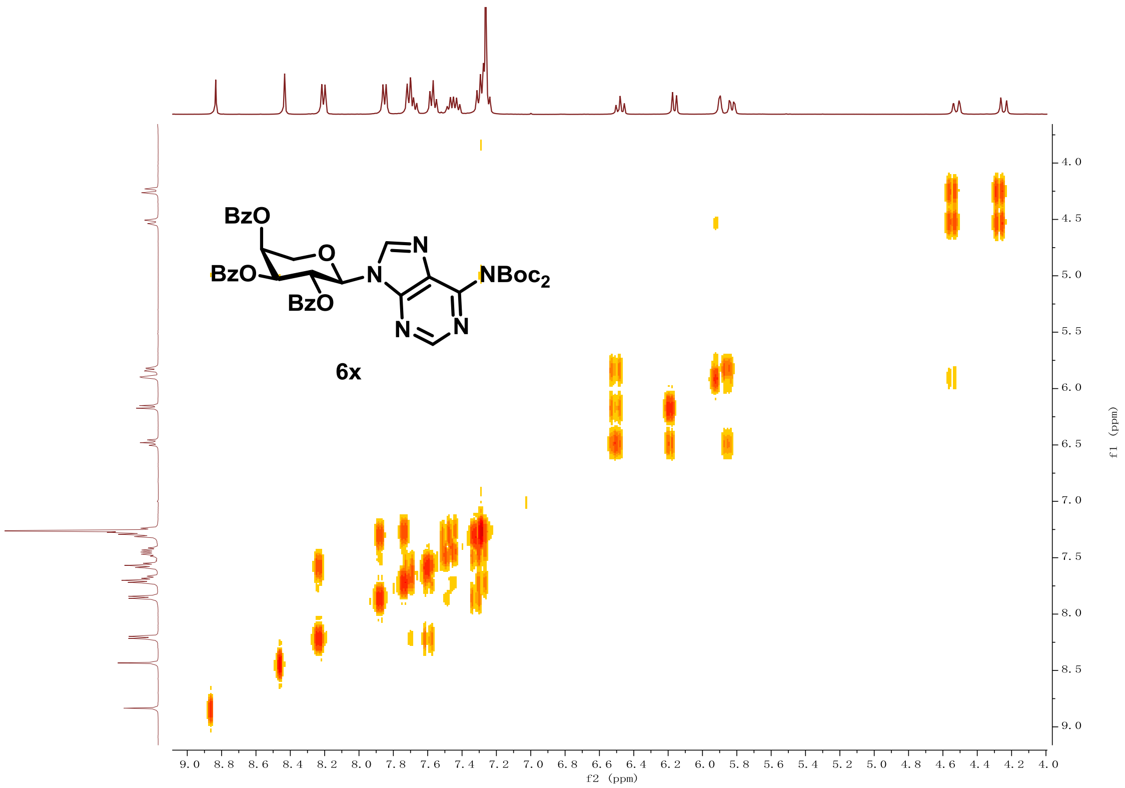

Supplementary Figure 227. COSY NMR Spectrum of Compound **6x**

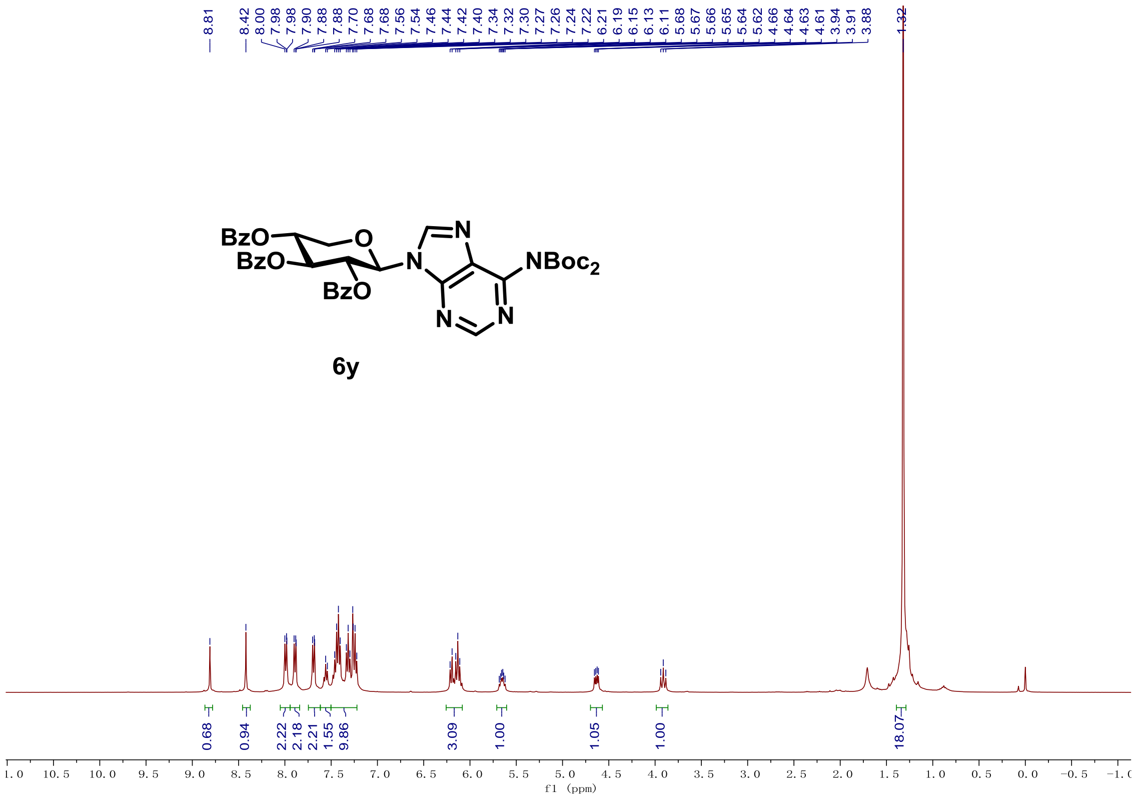

Supplementary Figure 228. ^{1}H NMR Spectrum of Compound **6y**

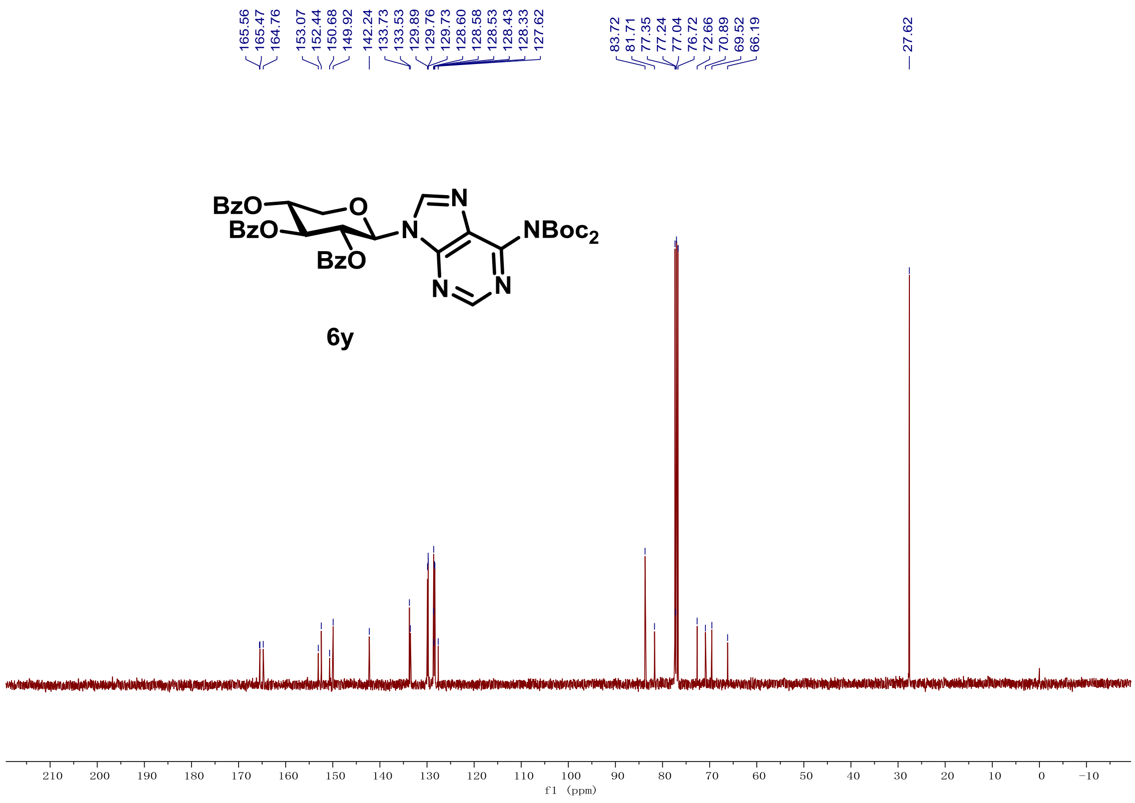

Supplementary Figure 229. ^{13}C NMR Spectrum of Compound **6y**

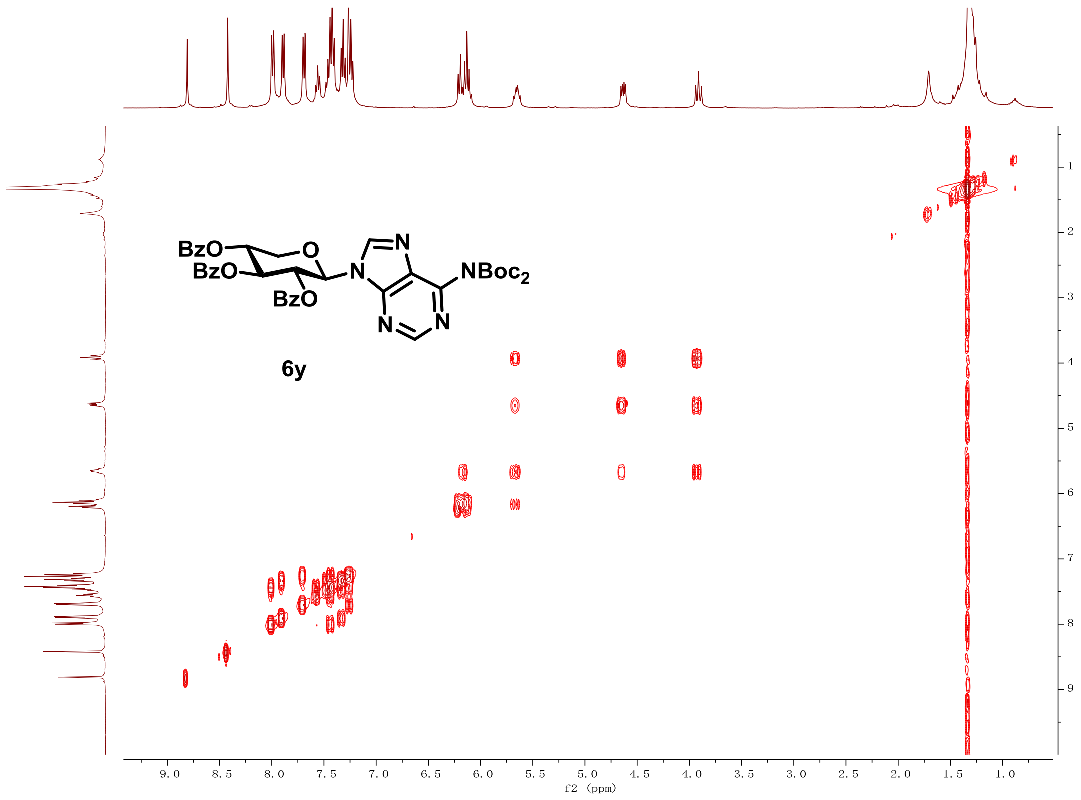

Supplementary Figure 230. COSY NMR Spectrum of Compound **6y**

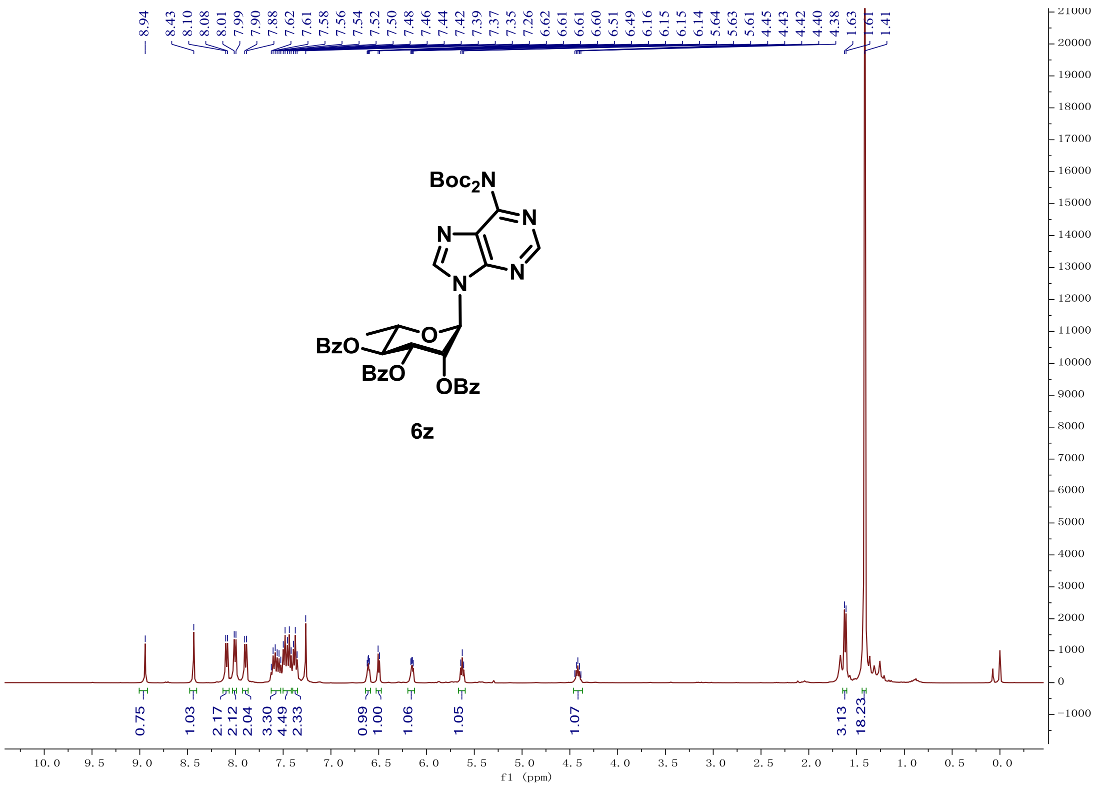

Supplementary Figure 231. ^{1}H NMR Spectrum of Compound **6z**

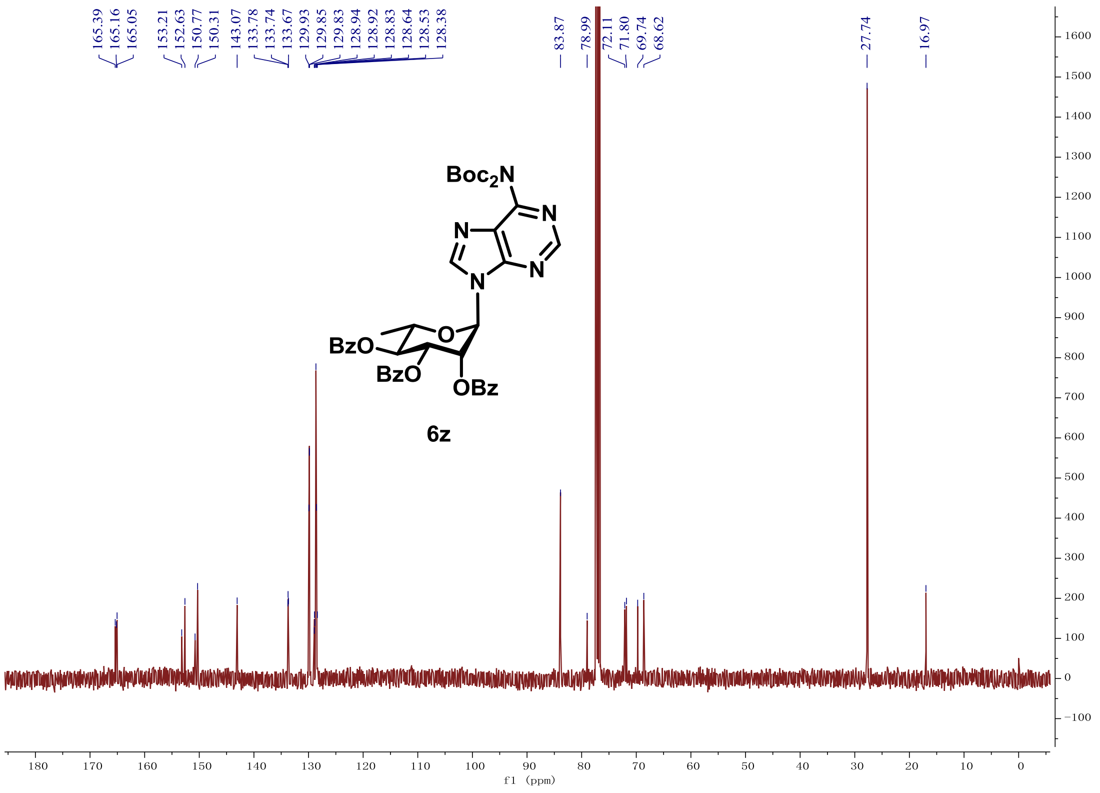

Supplementary Figure 232. ^{13}C NMR Spectrum of Compound **6z**

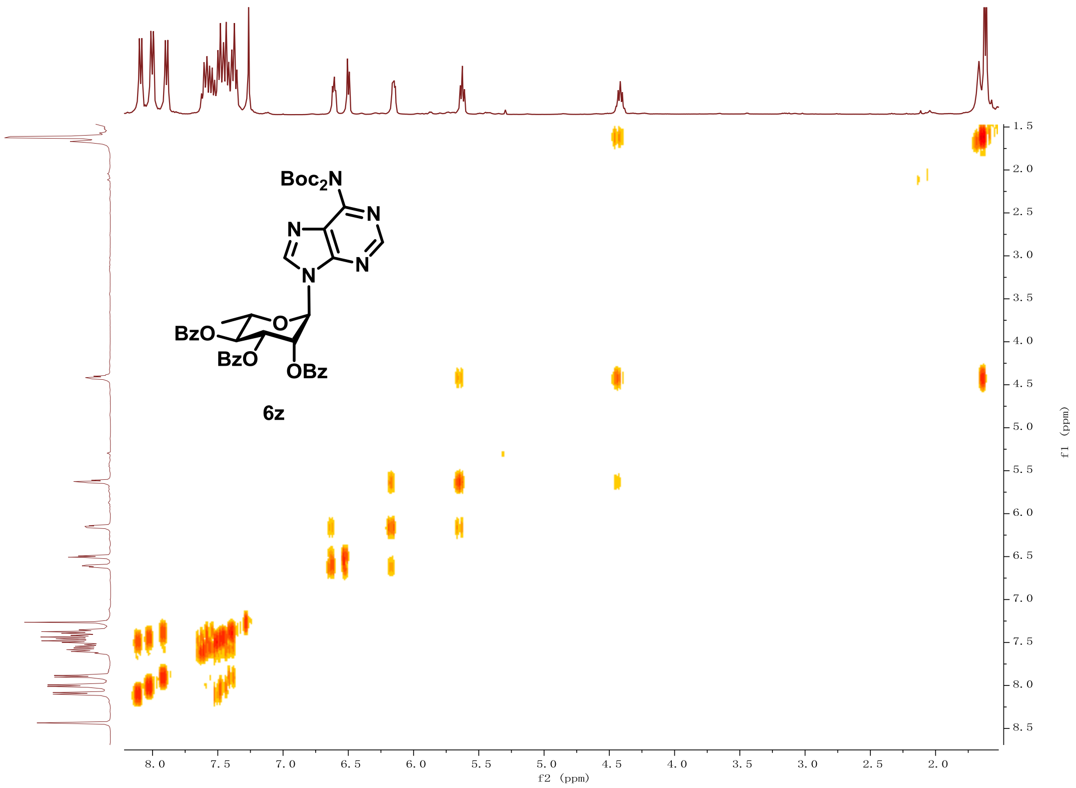

Supplementary Figure 233. COSY NMR Spectrum of Compound **6z**

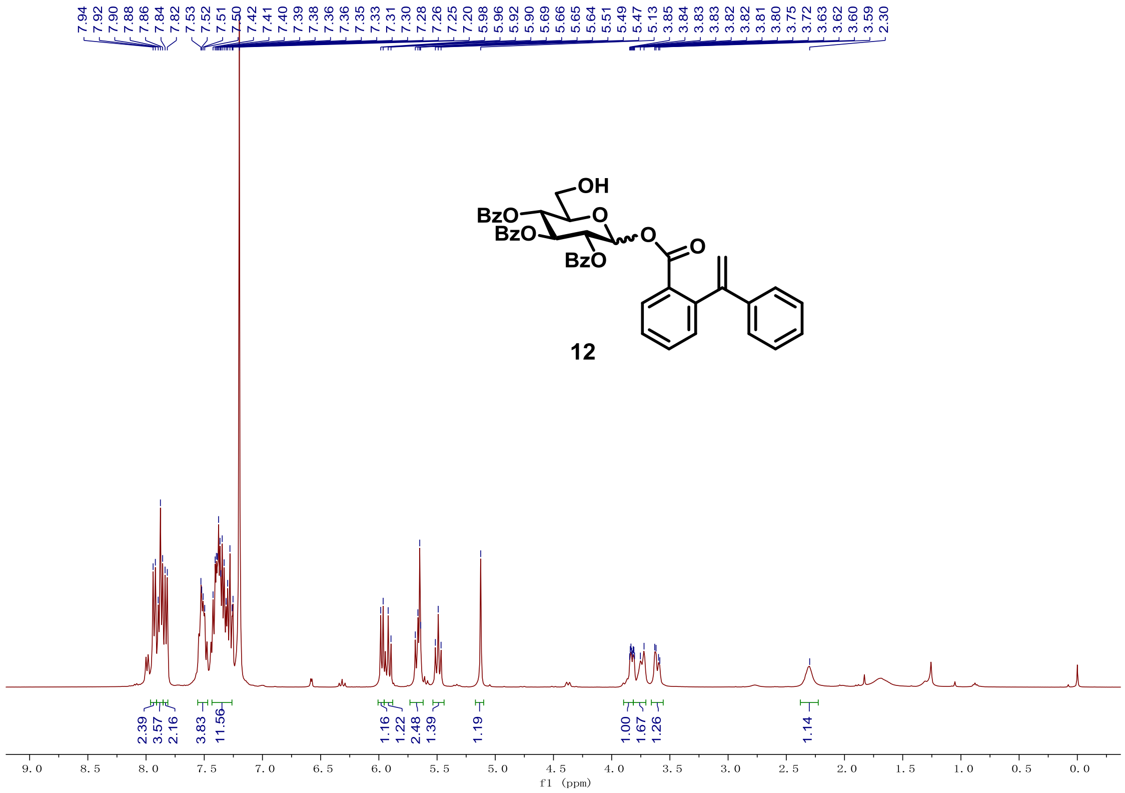

Supplementary Figure 234. ^{1}H NMR Spectrum of Compound **12**

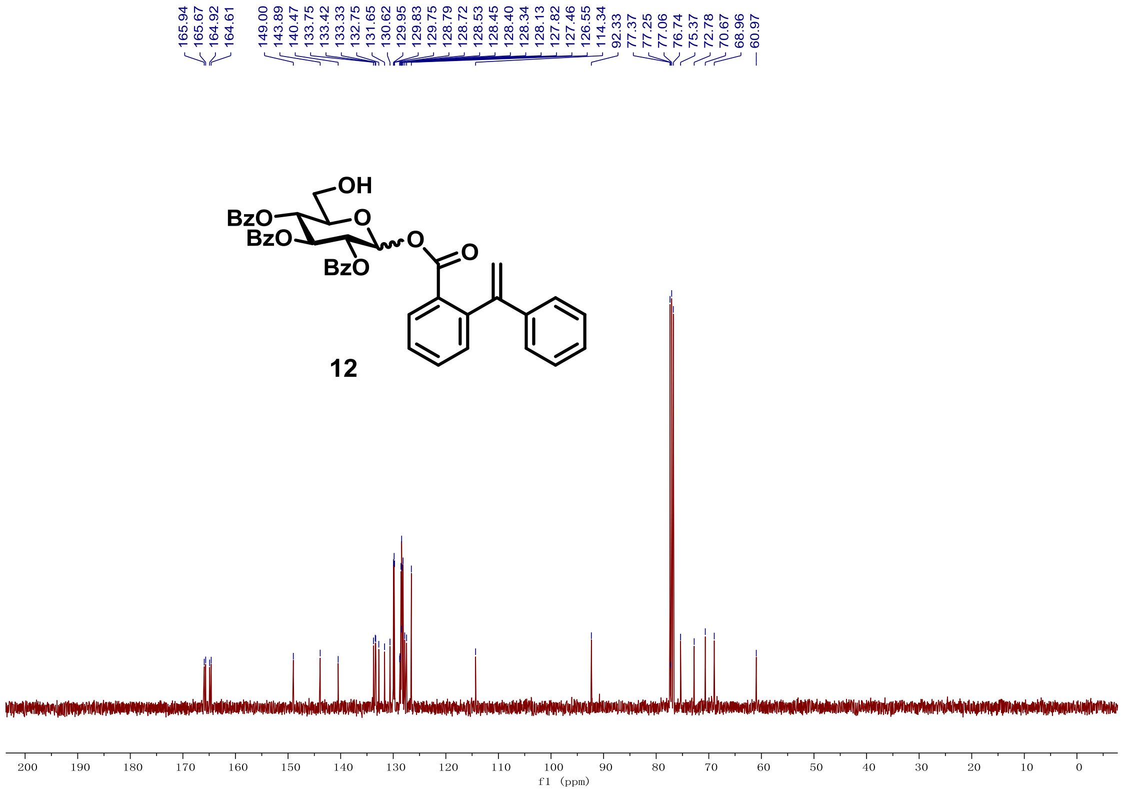

Supplementary Figure 235. ^{13}C NMR Spectrum of Compound **12**

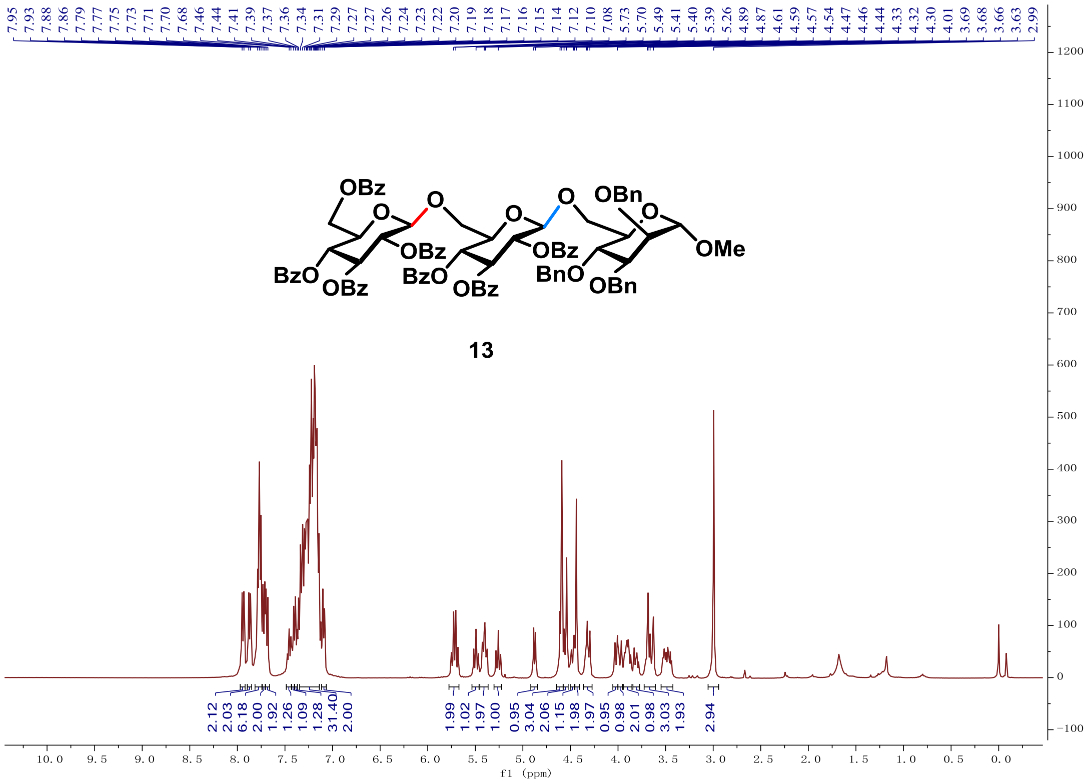

Supplementary Figure 236. ^{1}H NMR Spectrum of Compound **13**

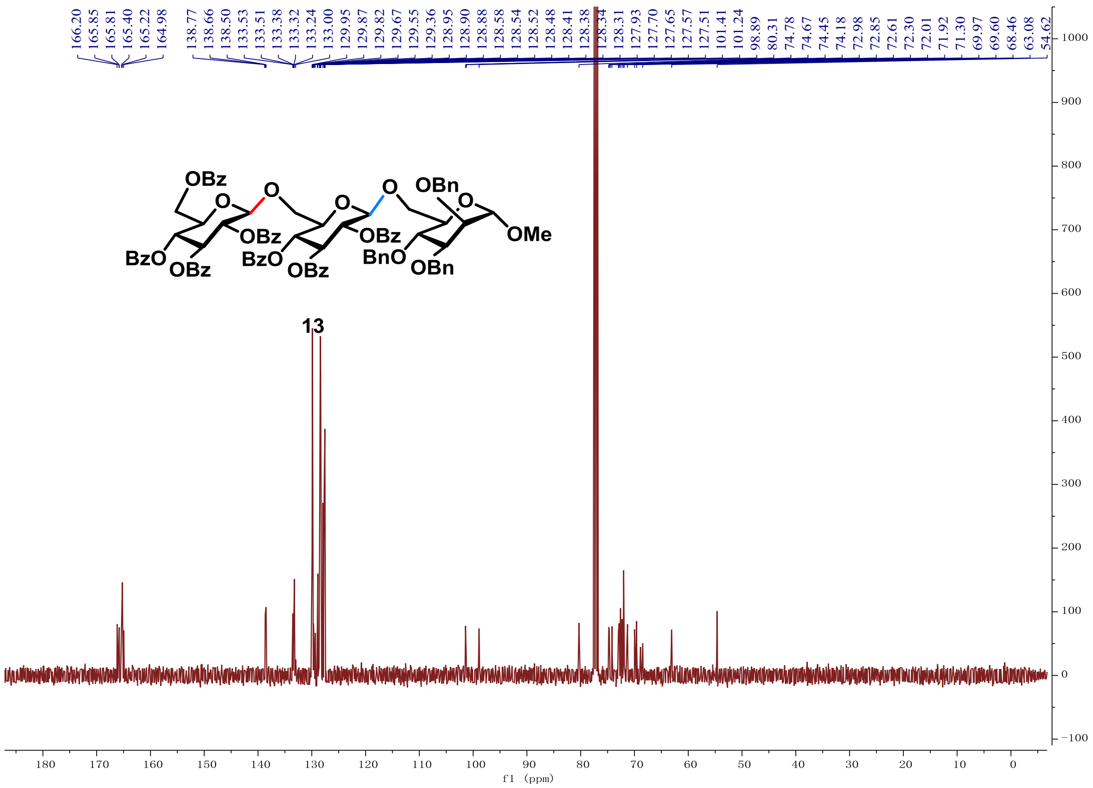

Supplementary Figure 237. ^{13}C NMR Spectrum of Compound **13**

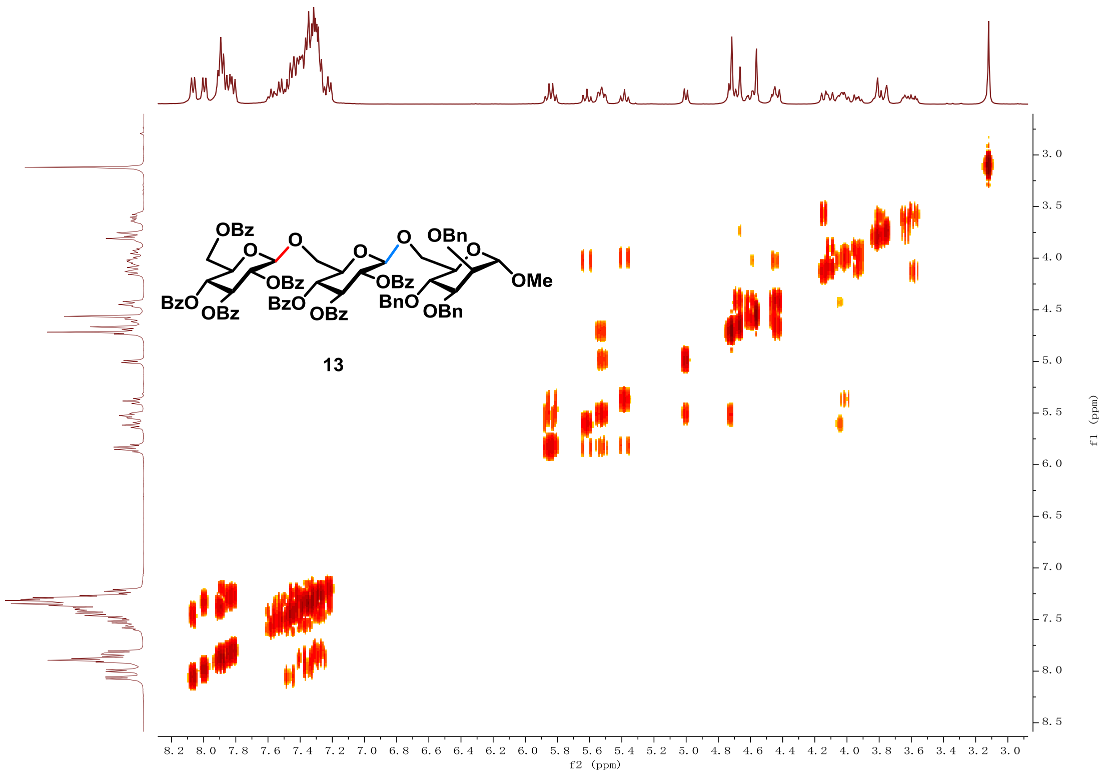

Supplementary Figure 238. COSY NMR Spectrum of Compound **13**

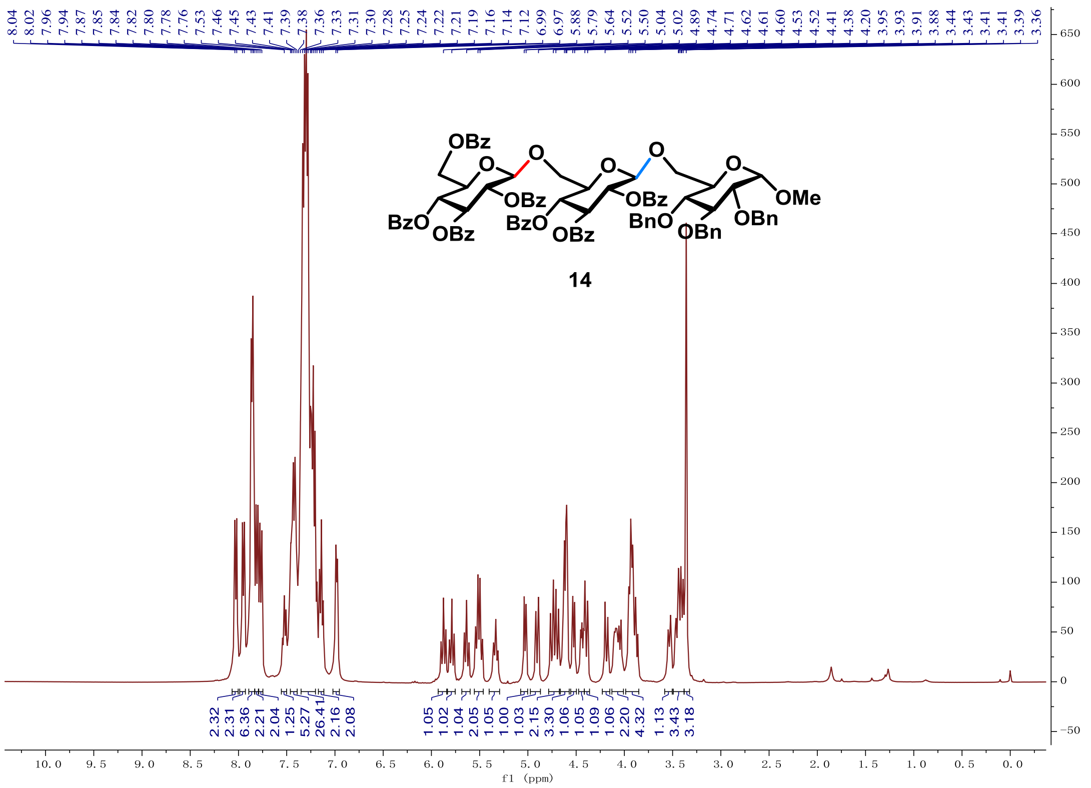

Supplementary Figure 239. ^{1}H NMR Spectrum of Compound **14**

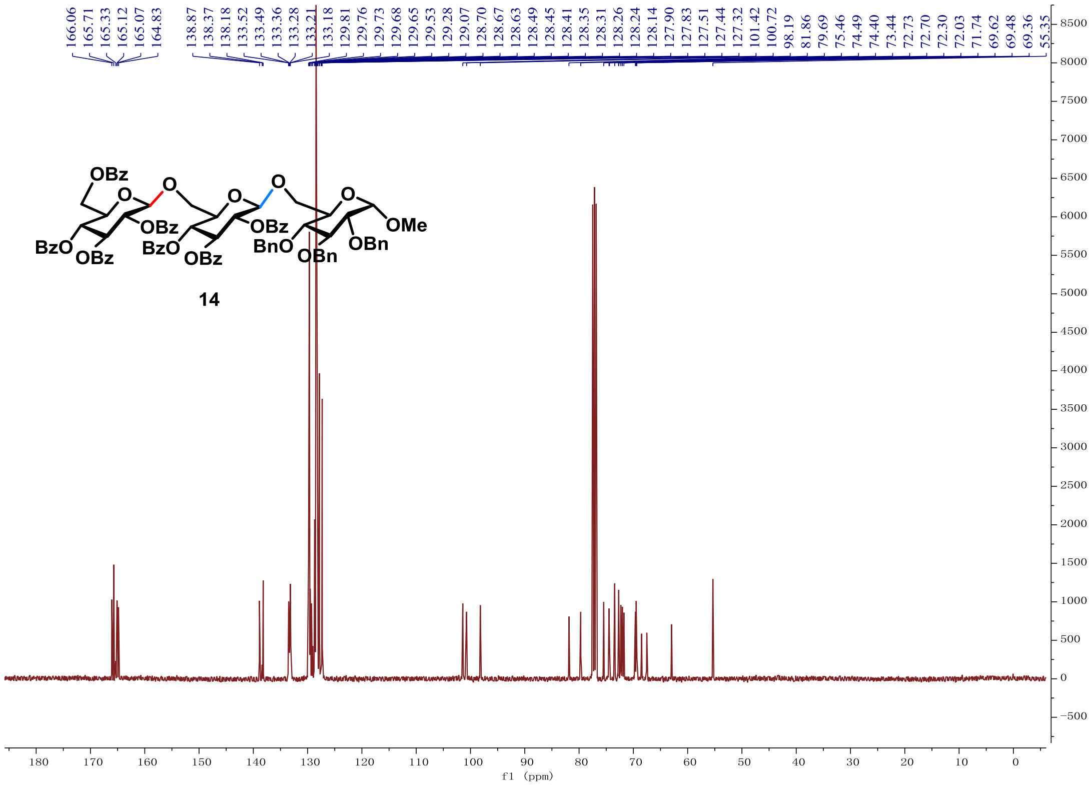

Supplementary Figure 240. ^{13}C NMR Spectrum of Compound **14**

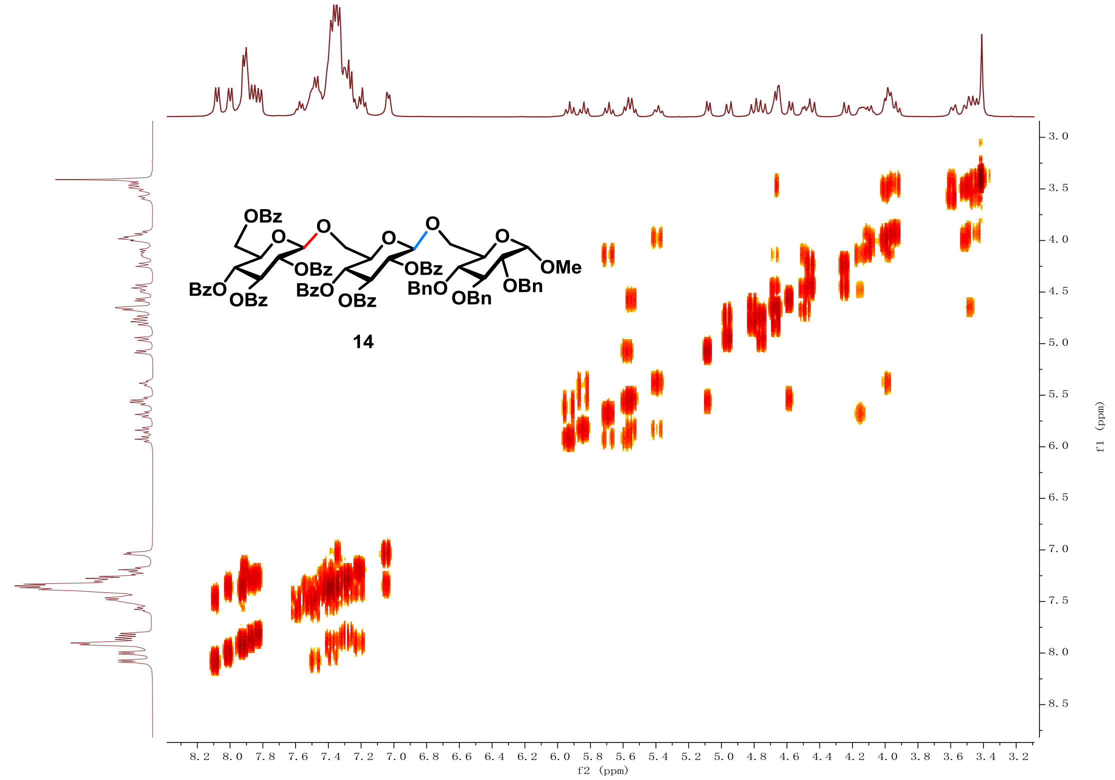

Supplementary Figure 241. COSY NMR Spectrum of Compound **14**

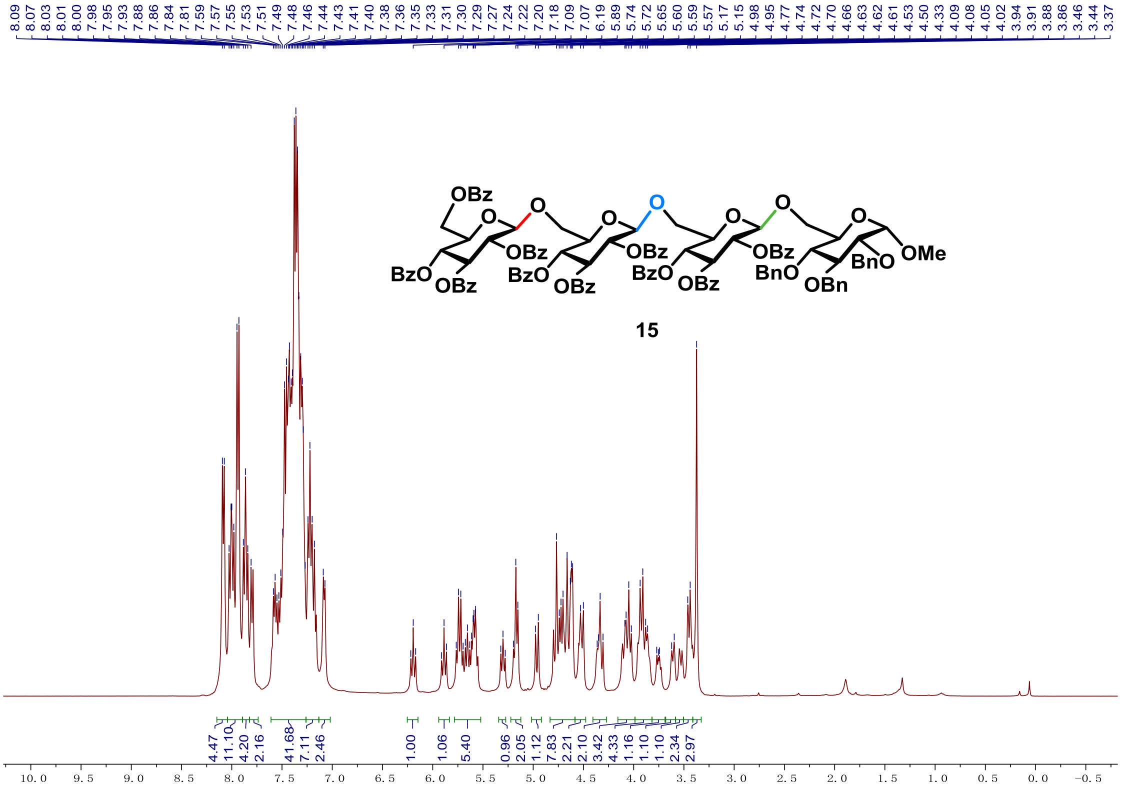

Supplementary Figure 242. ¹H NMR Spectrum of Compound **15**

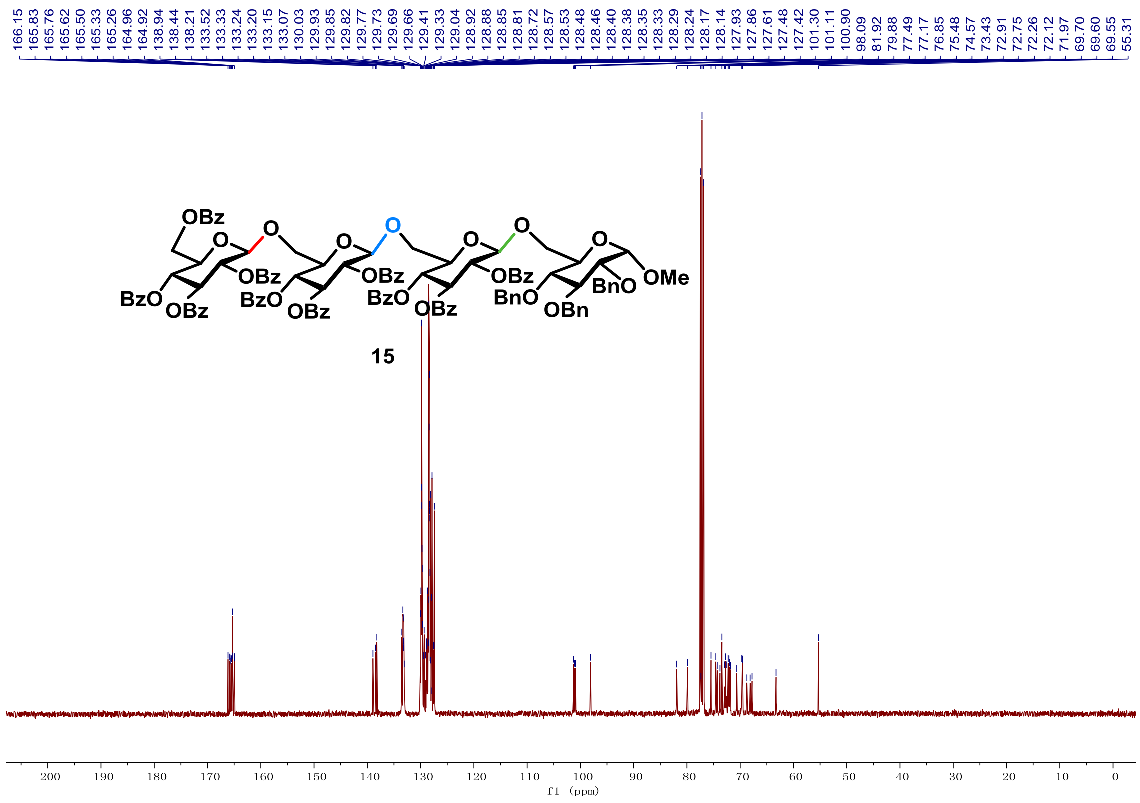

Supplementary Figure 243. ^{13}C NMR Spectrum of Compound **15**

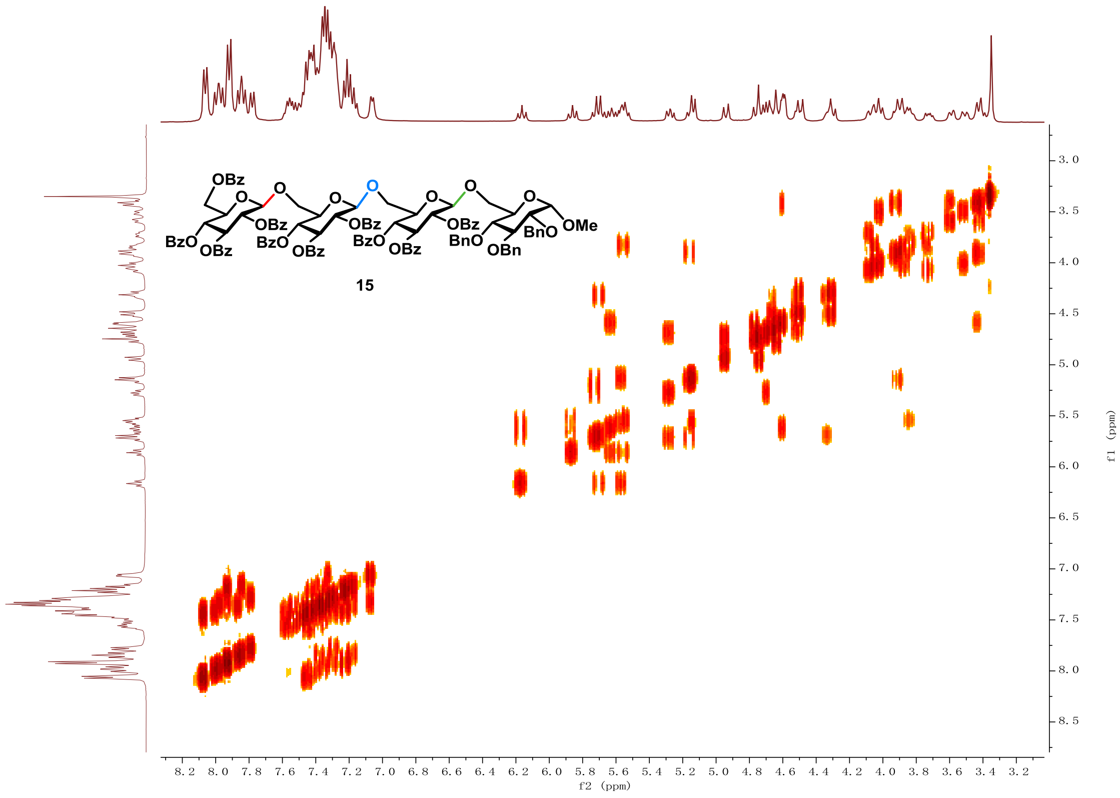

Supplementary Figure 244. COSY NMR Spectrum of Compound **15**

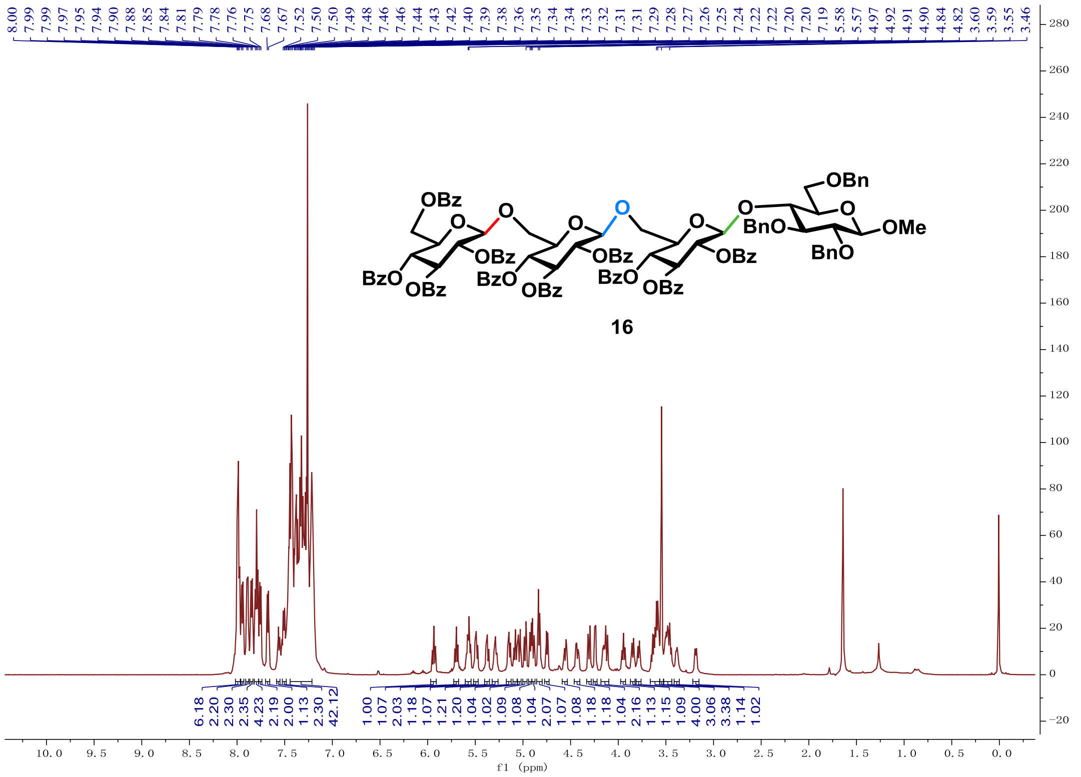

Supplementary Figure 245. ^{1}H NMR Spectrum of Compound **16**

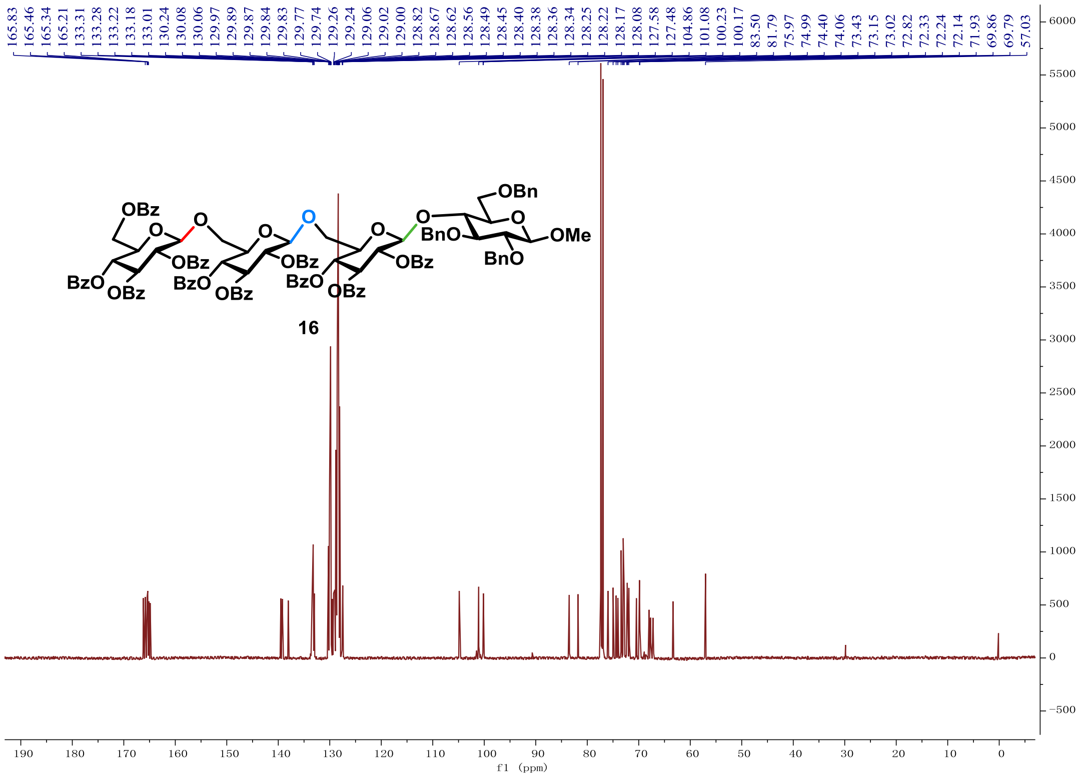

Supplementary Figure 246. ^{13}C NMR Spectrum of Compound **16**

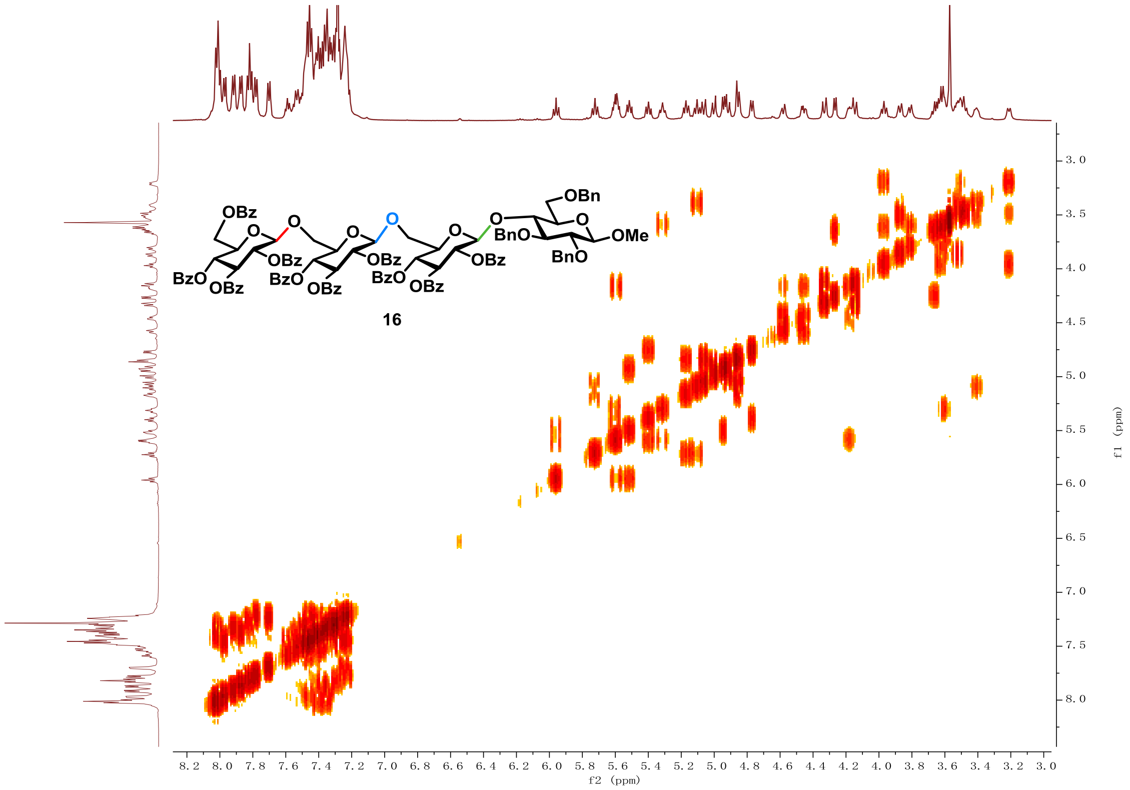

Supplementary Figure 247. COSY NMR Spectrum of Compound **16**

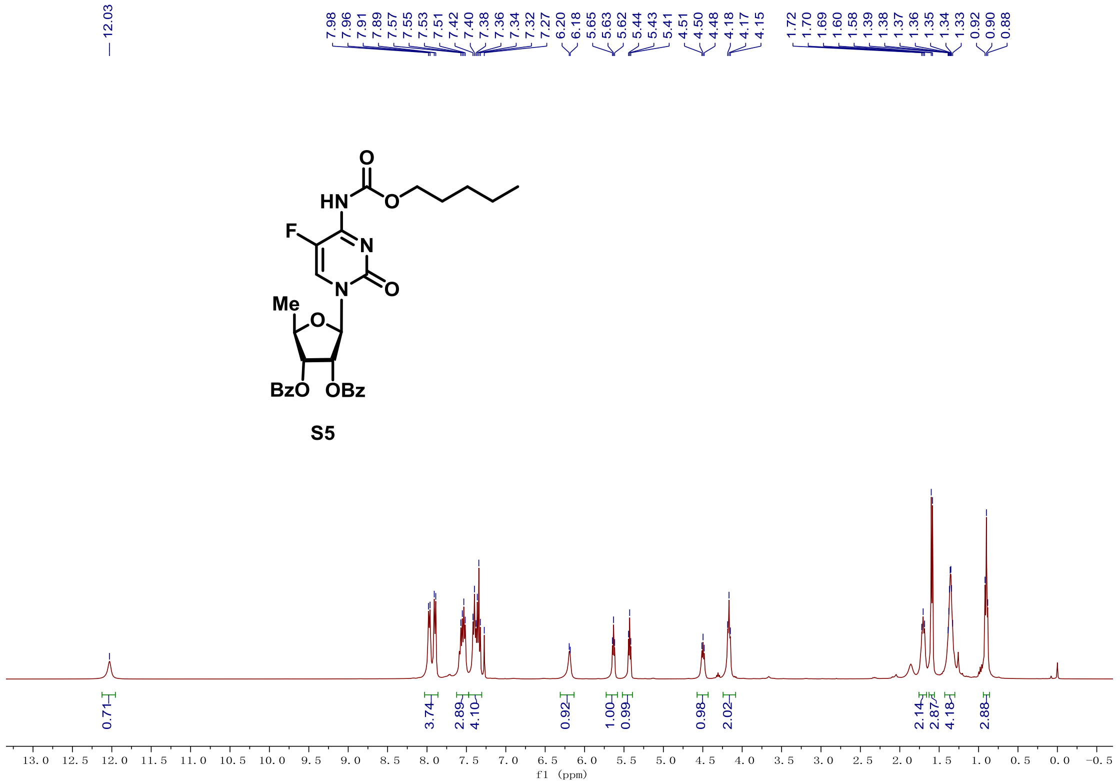

Supplementary Figure 248. ^{1}H NMR Spectrum of Compound **S5**

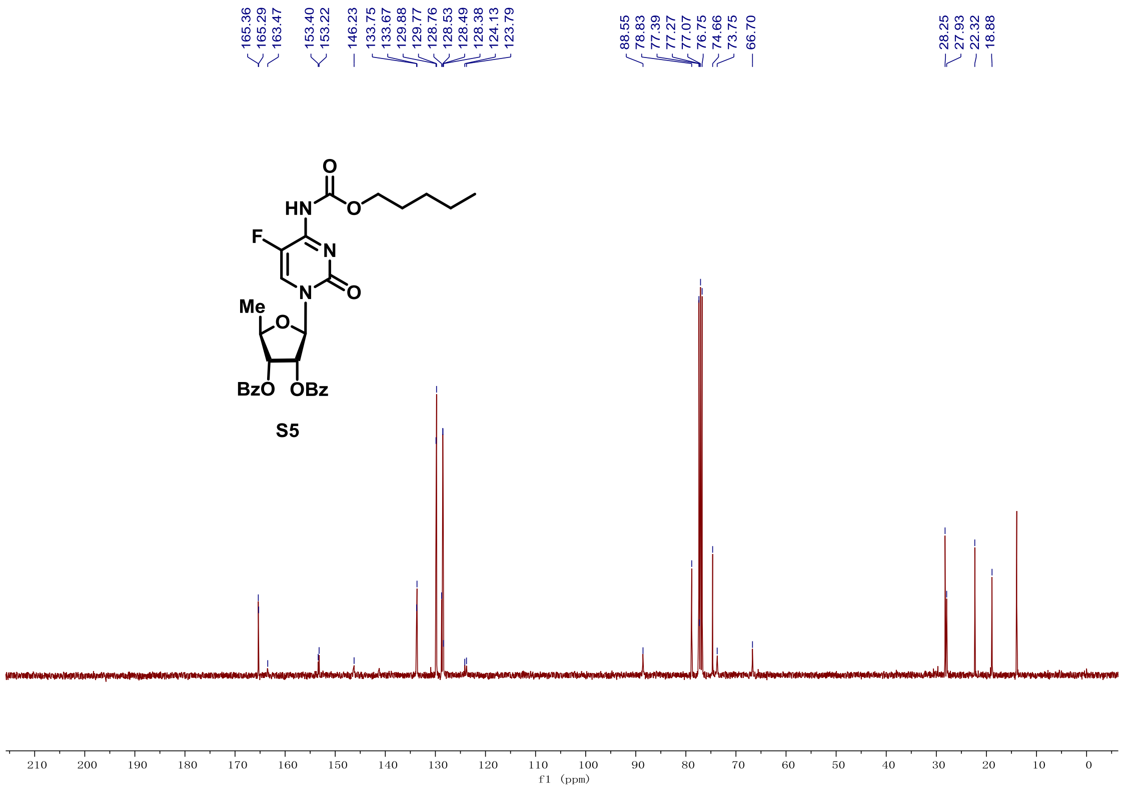

Supplementary Figure 249. ^{13}C NMR Spectrum of Compound **S5**

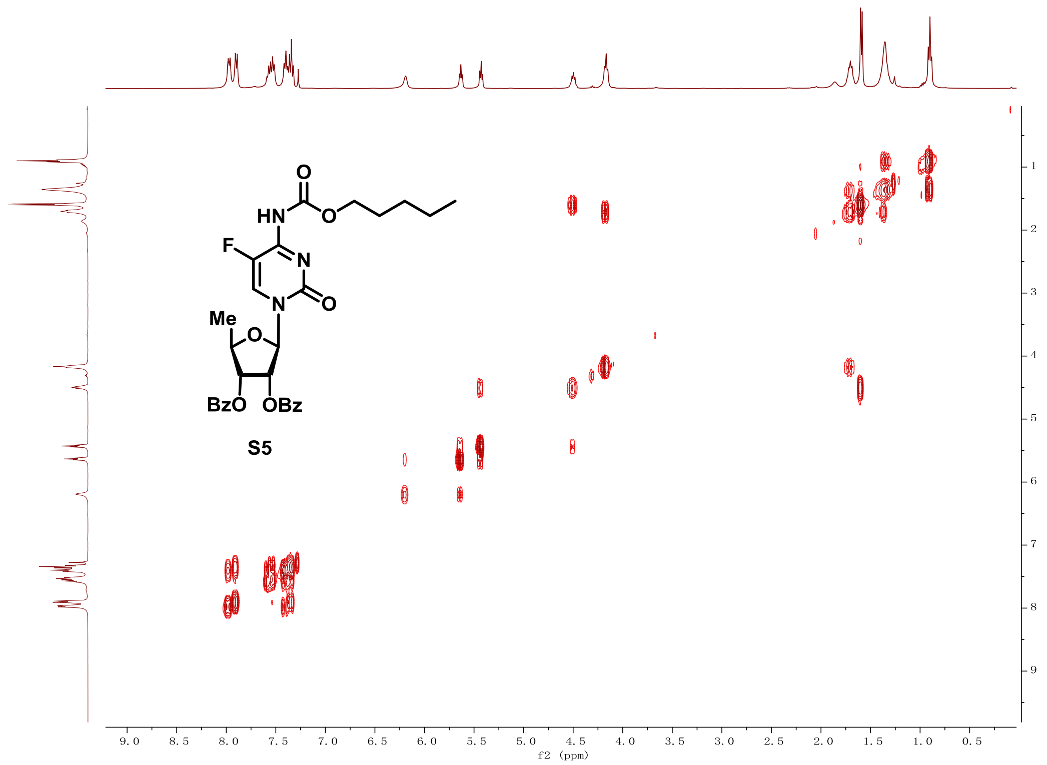

Supplementary Figure 250. COSY NMR Spectrum of Compound **S5**

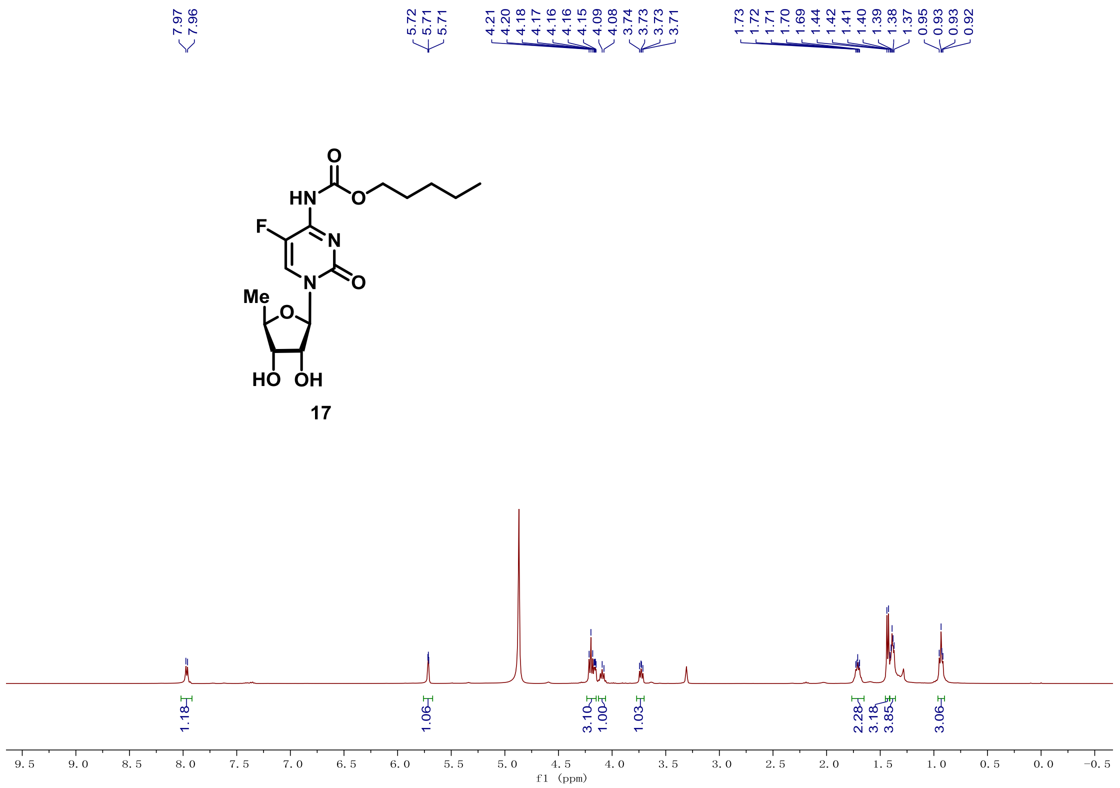

Supplementary Figure 251. ^{1}H NMR Spectrum of Compound **17**

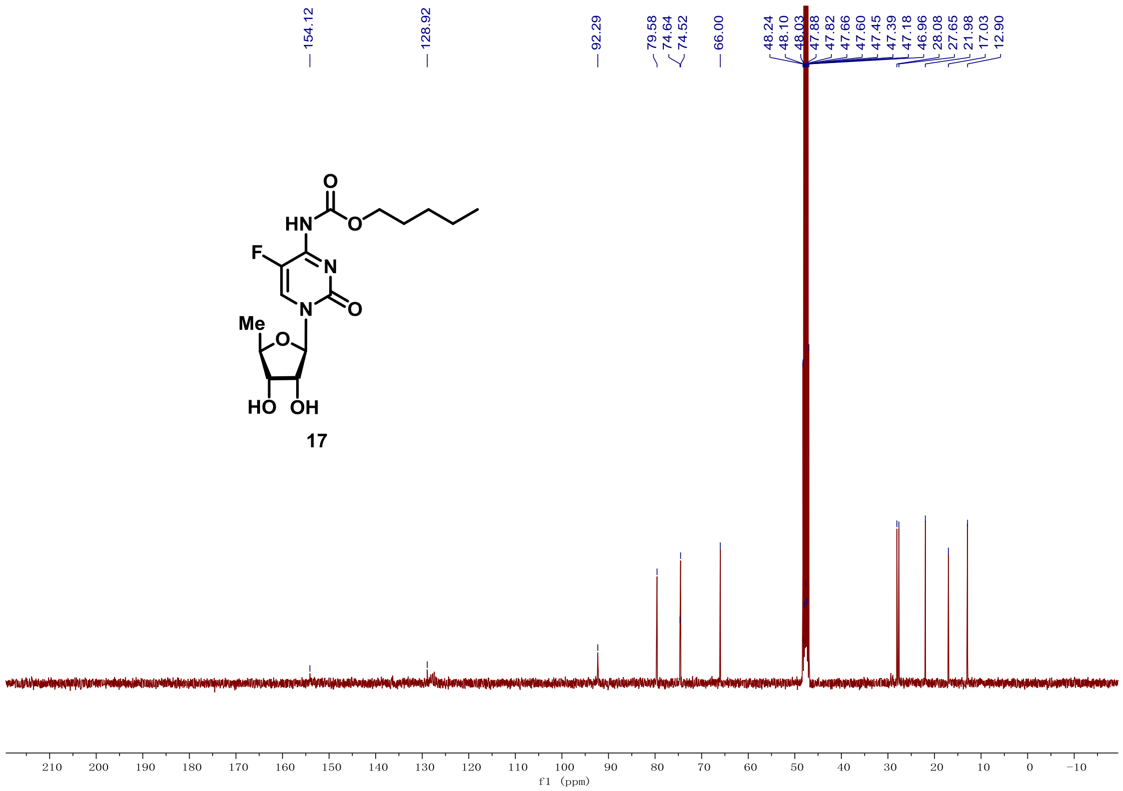

Supplementary Figure 252. ^{13}C NMR Spectrum of Compound **17**

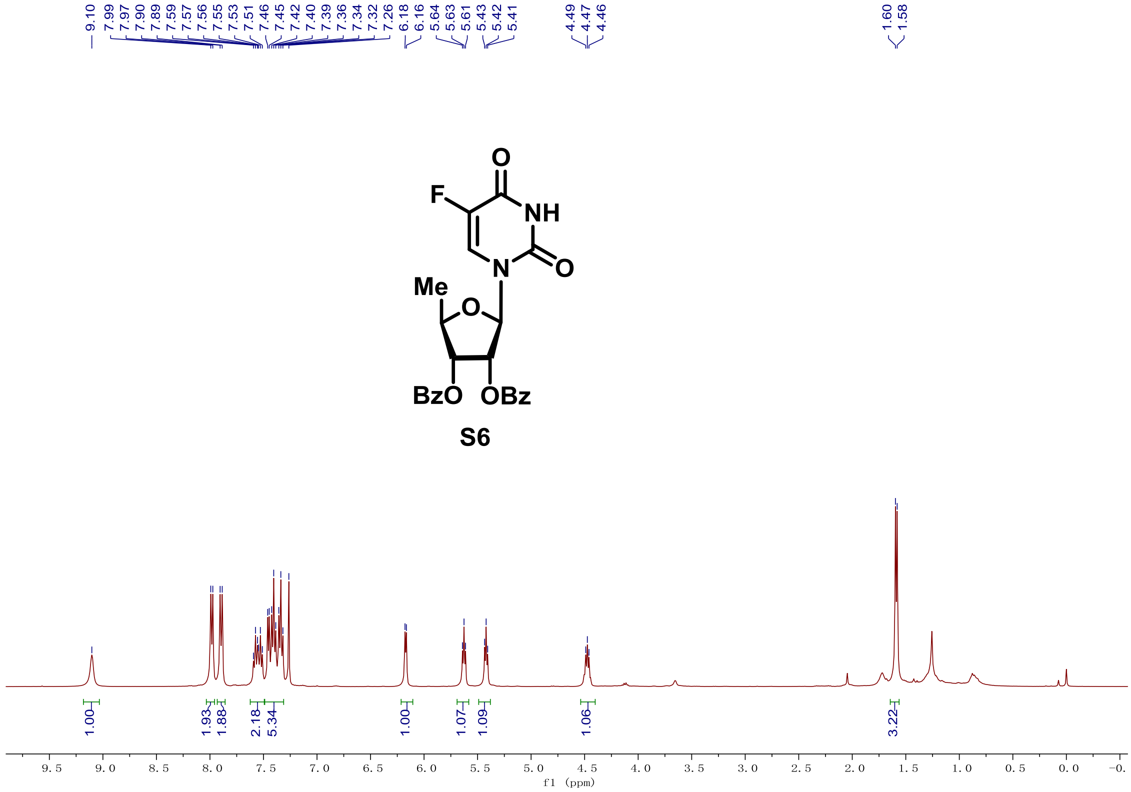

Supplementary Figure 253. ^{1}H NMR Spectrum of Compound **S6**

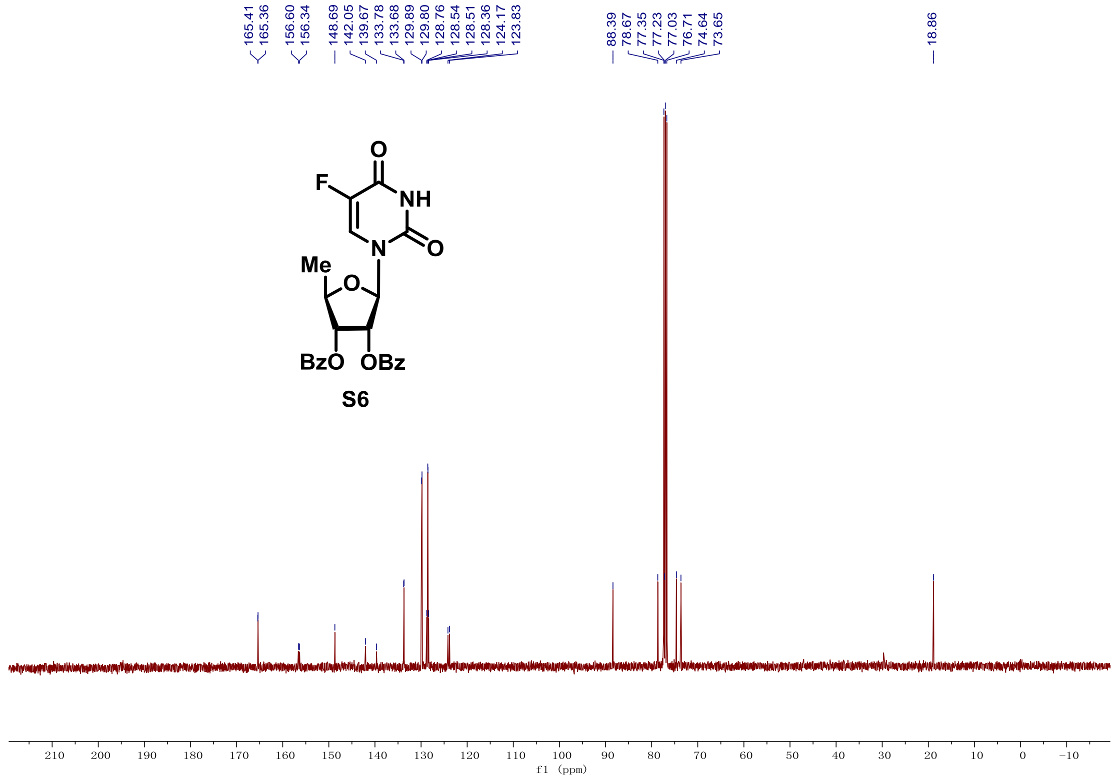

Supplementary Figure 254. ^{13}C NMR Spectrum of Compound **S6**

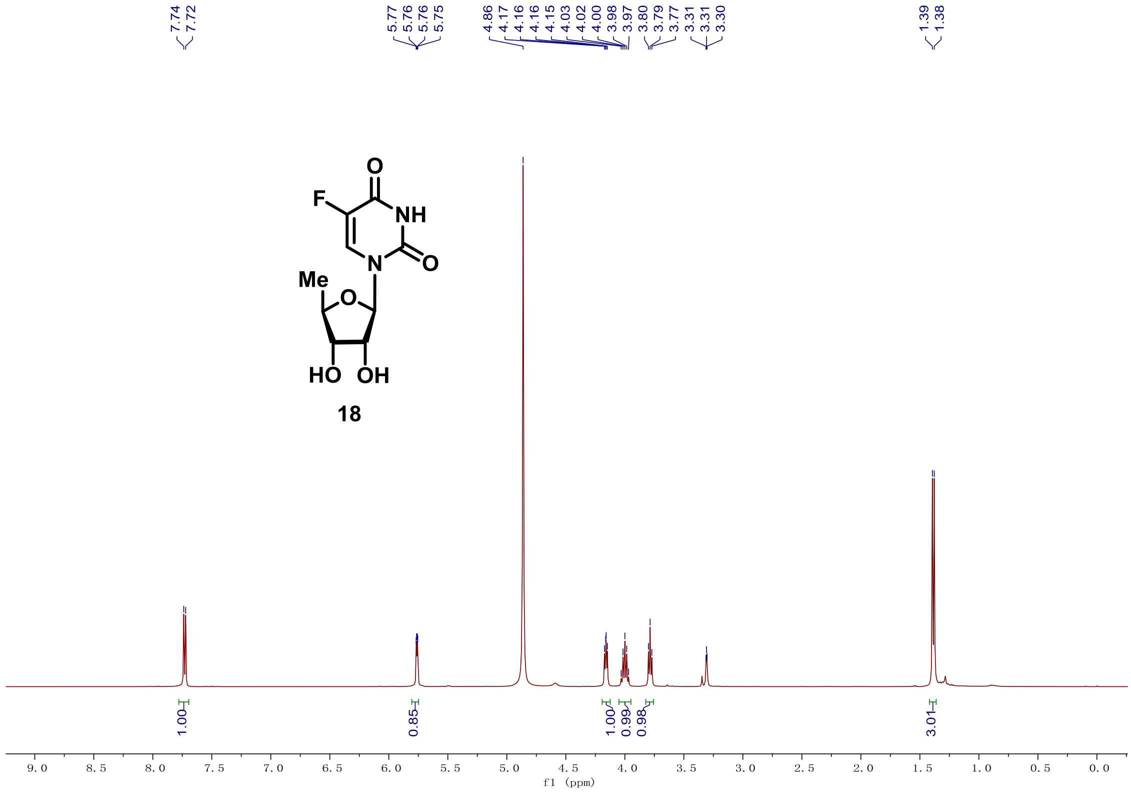

Supplementary Figure 255. ^{1}H NMR Spectrum of Compound **18**

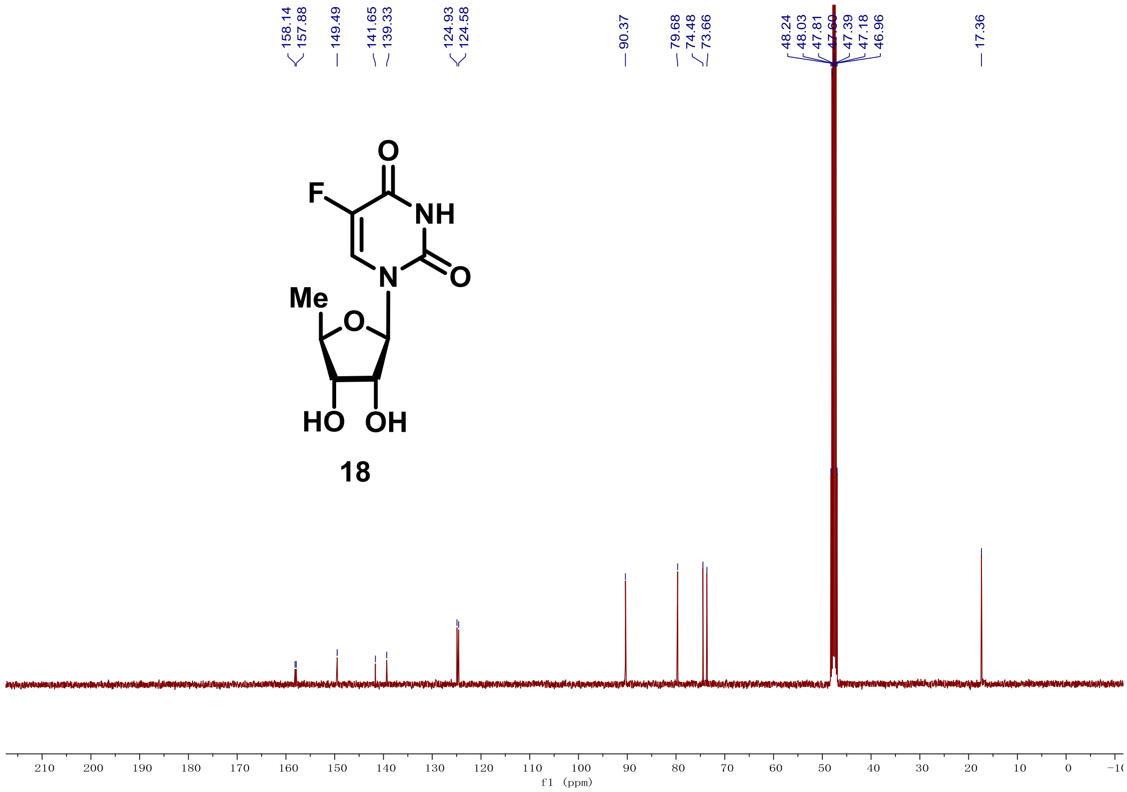

Supplementary Figure 256. ^{13}C NMR Spectrum of Compound **18**

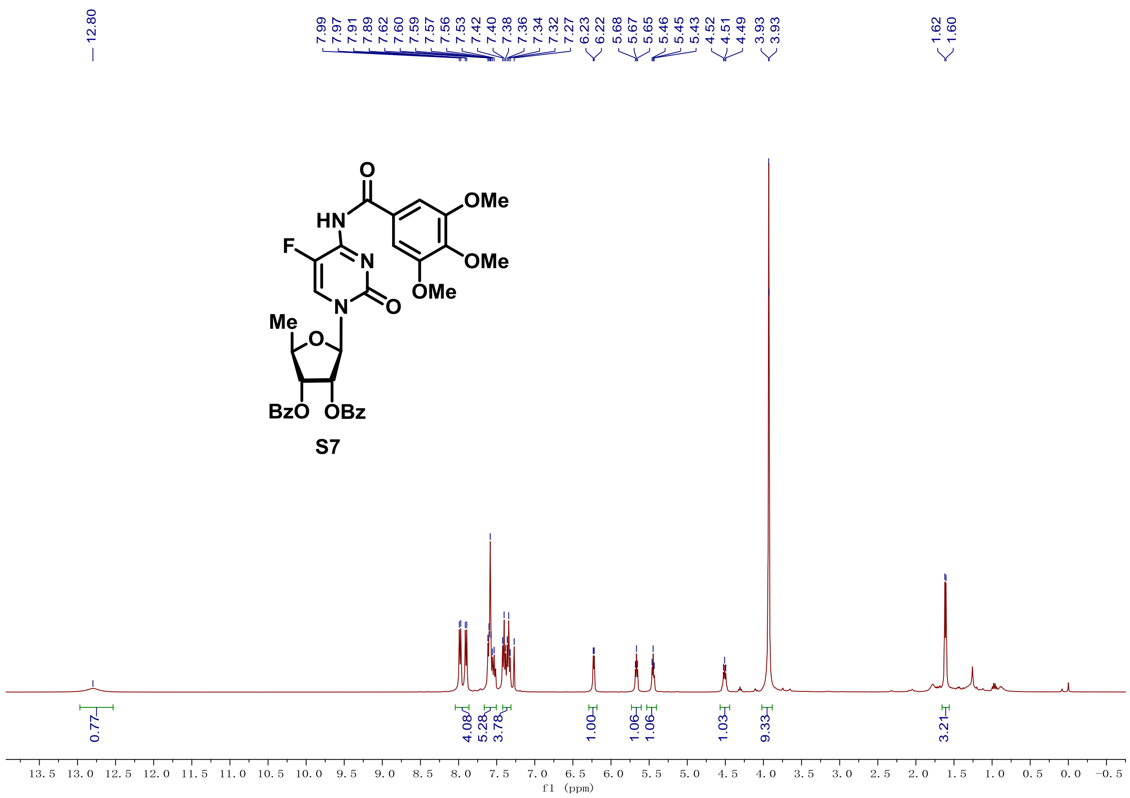

Supplementary Figure 257. ^{1}H NMR Spectrum of Compound **S7**

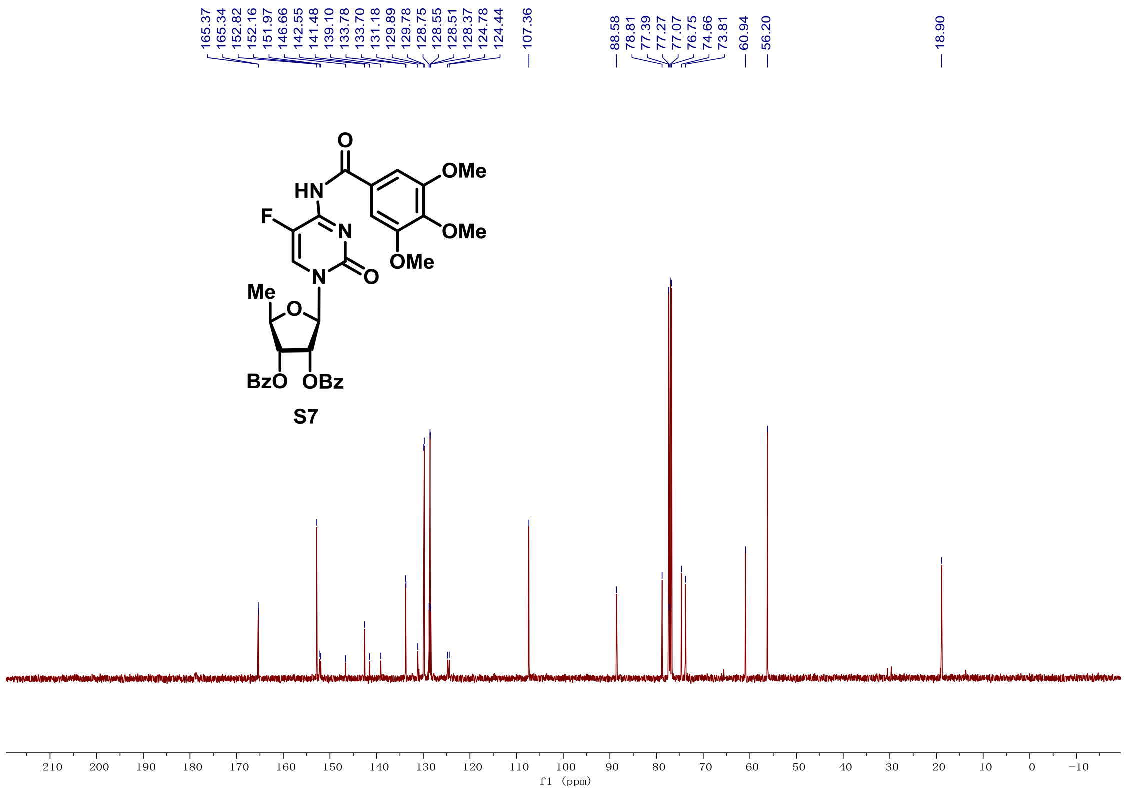

Supplementary Figure 258. ^{13}C NMR Spectrum of Compound **S7**

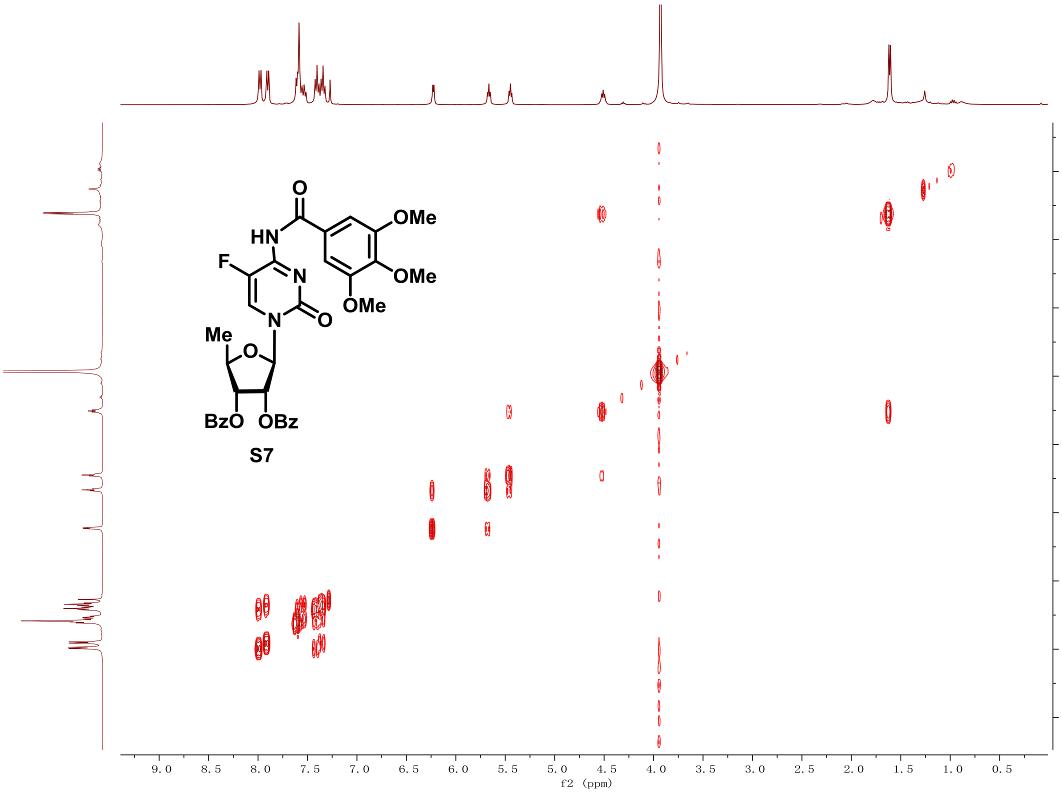

Supplementary Figure 259. COSY NMR Spectrum of Compound **S7**

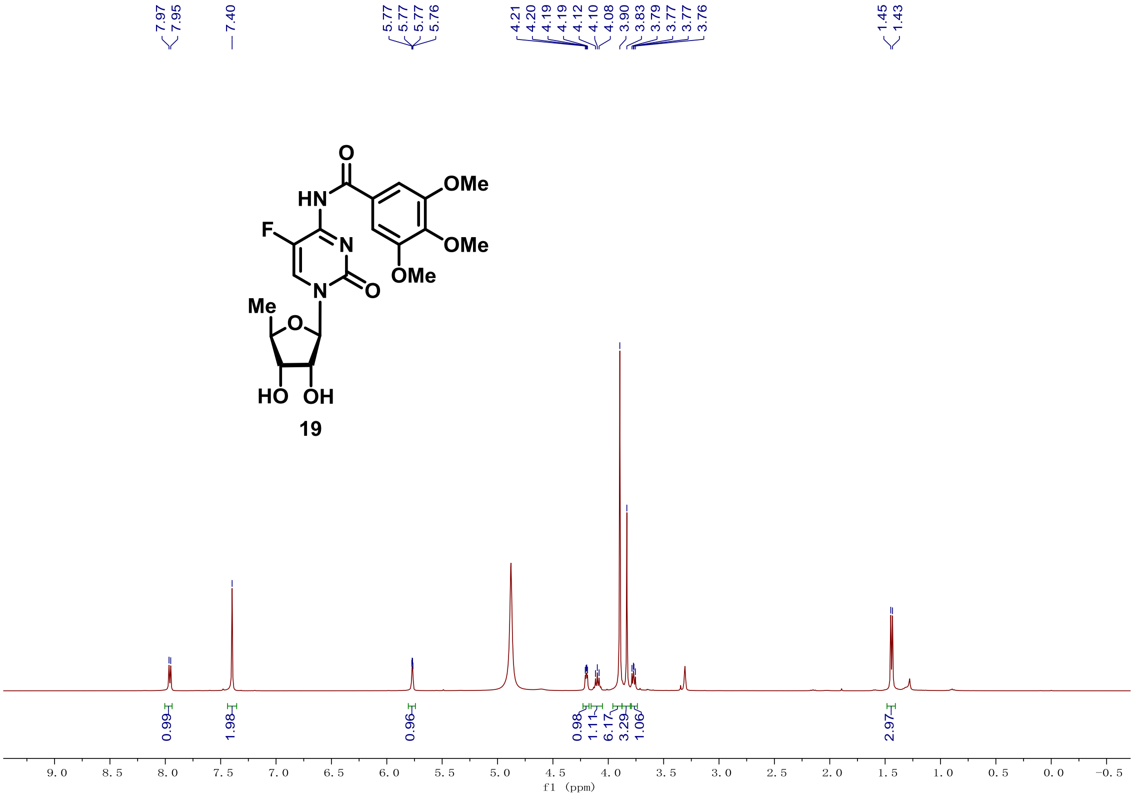

Supplementary Figure 260. ^{1}H NMR Spectrum of Compound **19**

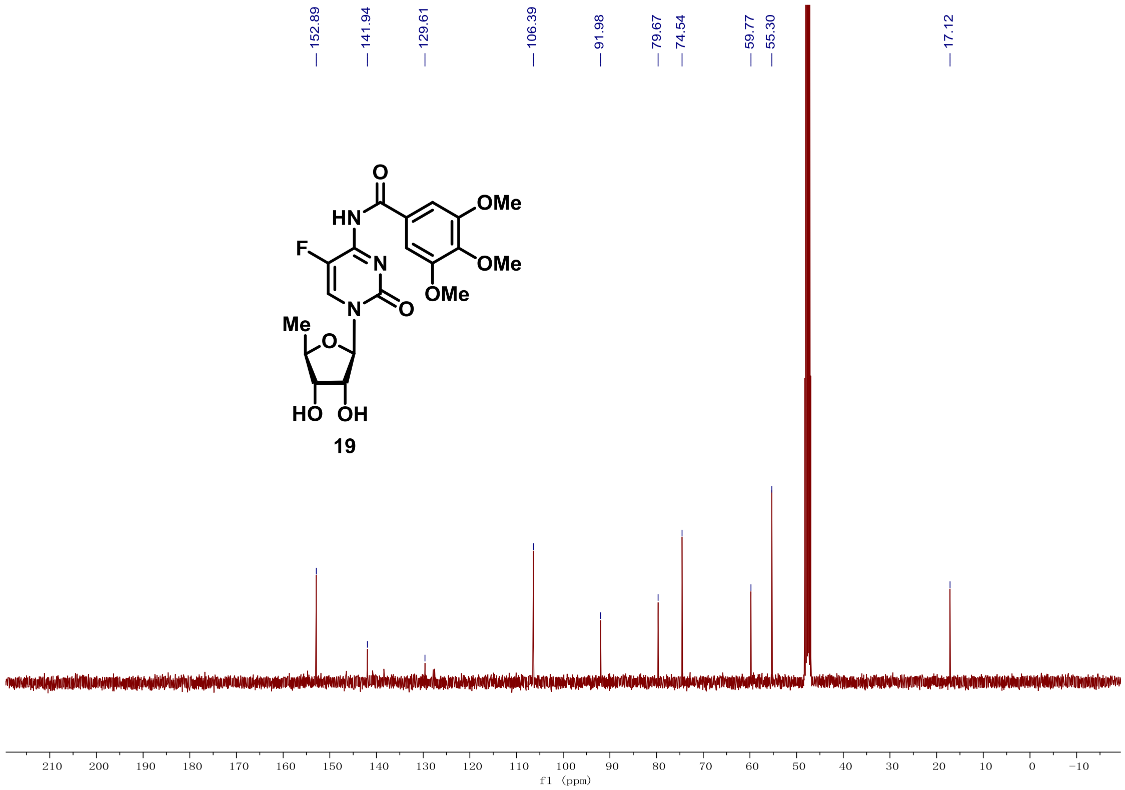

Supplementary Figure 261. ^{13}C NMR Spectrum of Compound **19**

Supplementary References

1. Song, S., Zhu, S., Yu, Y., Zhou, Q. Carboxy-directed asymmetric hydrogenation of 1,1-diarylethenes and 1,1-dialkylethenes. *Angew. Chem. Int. Ed.* **52**, 1556-1559 (2013).

2. Huo, G., Liu, C., Hui, Y., Chen, X., Xiao, D. Synthesis and structure-activity relationship of oleanolic mono- or di-glycosides against Magnaporthe oryzae. *Genetics & Molecular Research* **15**, gmr.15038998 (2016).

3. Santosh, V., Gopinath, J., Babu, P. V., Sainath, A. V. S., Reddy, A. V. R., Acetyl-D-glucopyranoside functionalized carbon nanotubes for the development of high performance ultrafiltration membranes. *Separation & Purification Technology* **191**, 134-143 (2017).

4. Lankalapalli, R. S., Baksa, A., Liliom, K., Bittman, R. Synthesis and properties of a photoactivatable analogue of psychosine (beta-Galactosylsphingosine). *ChemMedChem* **5**, 682-686 (2010).

5. Hartmann, M. et al. Saccharide-modified nanodiamond conjugates for the efficient detection and removal of pathogenic bacteria. *Chemistry - A European Journal* **18**, 6485-6492 (2012).

6. Doyle, L. M. et al. Stereoselective Epimerizations of Glycosyl Thiols. *Org. Lett.* **19**, 5802-5805 (2017).

7. Qi, S., Qingkun, Y., Shanshan, G., Quanlei, F., Qiang, X., Synthesis and enzymatic evaluation of phosphoramidon and its β anomer: Anomerization of α-l-rhamnose triacetate upon phosphitylation. *Bioorganic & Medicinal Chemistry* **21**, 6778-6787 (2013).

8. Wang, R. et al. Hafnium(IV) triflate as a potent catalyst for selective 1-O-deacetylation of peracetylated saccharides. *Carbohydr Res* **455**, 114-118 (2017).

9. Gauthier, C. et al. Synthesis of two natural betulinic acid saponins containing α-L-rhamnopyranosyl-(1 → 2)-α-L-arabinopyranose and their analogues. *Tetrahedron* **64**, 7386-7399 (2008).

10. Andersen, M. C., Kracun, S. K., Rydahl, M. G., Willats, W. G., Clausen, M. H. Synthesis of β-1,4-Linked Galactan Side-Chains of Rhamnogalacturonan€I. *Chem. Eur. J.* **22**, 11543-11548 (2016).

11. Lengeler, D., Weisz, K. Novel phthalimide nucleosides for the specific recognition of a CG Watson-Crick base pair. *Tetrahedron Letters* **42**, 1479-1481 (2001).

12. Pasari, S., Manmode, S., Walke, G., Hotha, S. A Versatile Synthesis of Pentacosafuranoside Subunit Reminiscent of Mycobacterial Arabinogalactan Employing One Strategic Glycosidation Protocol. *Chem.Eur. J.* **24**, 1128-1139 (2017).

13. Kraska, B.; Lichtel, R., Distribution of tautomers in acylated ketoses an important factor in ketoside synthesis. *Tetrahedron. Let*. **24**, 361-364 (1983).

14. Mach, M., Schlueter, U., Mathew, F., Fraser-Reid, B., Hazen, K. C. Comparing n-pentenyl orthoesters and n-pentenyl glycosides as alternative glycosyl donors. *Tetrahedron* **58**, 7345-7354 (2002).

15. Pitsch, S. An Efficient Synthesis of Enantiomeric Ribonucleic Acids from D-Glucose. *Helvetica* **80**, 2286-2314 (2010).

16. Wei, P. et al. Iodine Monochloride (ICl) as a Highly Efficient, Green Oxidant for the Oxidation of Alcohols to Corresponding Carbonyl Compounds. *Synthetic Communications* **45**, 1457-1470 (2015).

17. Rani, S., Agarwal, A., Vankar, Y. D. $LaCl_3$-$7H_2O$/NaI/benzyl alcohol: a novel reagent system for regioselective hydration of glycals: application in the synthesis of 1,6-dideoxynojirimycin. Tetrahedron Lett. **44**, 5001-5004 (2003).

18. Pischel, H., Wagner, G. Synthesis of 5'-modified nucleoside analogues of pyridone-(2) and 5,6-diphenylpyridazone-(3). 37. Glycosides from heterocyclic compounds. *Pharmazie*, **25**, 45-54 (1970).

19. Xia, M., Yao, W., Meng, X., Lou, Q., Li, Z., $Co_2(CO)_6$-propargyl cation mediates glycosylation reaction by using thioglycoside. *Tetrahedron Letters* **58**, 2389-2392 (2017).

20. Teruhiko, I., Yoshihiro, S., Takayuki, K., Seiki, S. Conversion of D-glucose to cyclitol with hydroxymethyl substituent via intramolecular silyl nitronate cycloaddition reaction: application to total synthesis of (+)-cyclophellitol. *Org. Lett.* **5**, 3879-3882 (2003).

21. Ge, S., Tu, Y., Xia, J., Sun, J. Synthetic Investigation toward the D-Ring-Functionalized Cytotoxic Oleanane-Type Saponins Pithedulosides D and E. *European Journal of Organic Chemistry* **2017**, 3929-3934 (2017).

22. Hevey, R., Ling, C. Studies on the 6-homologation of beta-D-idopyranosides. *Carbohydr. Res.* **445**, 65-74 (2017).

23. Garegg, P., Iversen, T., Oscarson, S. Monobenzylation of diols using phase-transfer catalysis. *Carbohydr. Res.* **50**, C12-C14 (1976).

24. Huang, X., Huang, L., Wang, H., Ye, X. Iterative one-pot synthesis of oligosaccharides. *Angew. Chem. Int. Ed. Engl.* **43**, 5221-5224 (2004).

25. Yan, M., Liu, Y., Chen, H., Ke, Y., Xu, Q., Cheng, M. Synthesis and antitumor activity of two natural N-acetylglucosamine-bearing triterpenoid saponins: lotoidoside D and E. *Bioorg. Med. Chem. Lett.* **16**, 4200-4204 (2006).

26. Zhang, Y. et al. Orthogonal One-Pot Synthesis of Oligosaccharides Based on Glycosyl ortho-Alkynylbenzoates. *Org. Lett.* **21**, 2335-2339 (2019).

27. Zhang, Q., Sun, J., Zhu, Y., Zhang, F., Yu, B. An Efficient Approach to the Synthesis of Nucleosides: Gold(I)-Catalyzed N-Glycosylation of Pyrimidines and Purines with Glycosyl ortho-Alkynyl Benzoates. *Angew. Chem. Int. Ed.* **50**, 4933-4936 (2011).

28. Shen, B., Jamison, T. Rapid Continuous Synthesis of 5'-Deoxyribonucleosides in Flow via Bronsted Acid Catalyzed Glycosylation. *Org. Lett.* **14**, 3348-3351 (2012).

29. Zhu, Y, Biao, Y. Characterization of the isochromen-4-yl-gold(I) intermediate in the gold(I)-catalyzed glycosidation of glycosyl ortho-alkynylbenzoates and enhancement of the catalytic efficiency thereof. *Angew. Chem. Int. Ed. Engl.* **123**, 8479-8482 (2011).

30. Nigel, A., Sergey, A., Robert, A. Efficient synthesis of methyl lycotetraoside, the tetrasaccharide constituent of the tomato defence glycoalkaloid alpha-tomatine. *Org. Bio. Chem.* **3**, 3201-3206 (2005).

31. Hu, Y. et al. o-(p-Methoxyphenylethynyl)phenyl Glycosides: Versatile New Glycosylation Donors for the Highly Efficient Construction of Glycosidic Linkages. *J. Am. Chem. Soc.***139**, 12736-12744 (2017).

32. Vidadala, S., Hotha, S. Methyl glycosides are identified as glycosyl donors for the synthesis of glycosides, disaccharides and oligosaccharides. *Chem. Commun.* **40**, 2505-2507 (2009).

33. Matteo, A., Gaspare, B., Alfonso, I., Marialuisa, S. Activation of glycoyl trihaloacetimidates with acid-washed molecular sieves in the glycosidation reaction. *Org. Lett.* **5**, 987-989 (2003).

34. Carthy, C., Tacke, M., Zhu, X. N-Trifluoromethylthiosaccharin/TMSOTf: A New Mild Promoter System for Thioglycoside Activation. *Eur. J. Org. Chem.* **2019**, 2729-2734 (2019).

35. Trujillo, M., Morales, E., Vazquez, J. Tetra-O-benzoylglucosylation: A New 1H Nuclear Magnetic Resonance Method for Determination of the Absolute Configuration of Secondary Alcohols. *J. Org. Chem.* **59**, 6637-6642 (1994).

36. Chem, X., Shen, D., Wang, Q., Yang, Y. Biao, Y. ortho-(Methyltosylaminoethynyl)benzyl glycosides as new glycosyl donors for latent-active glycosylation. *Chem. Commun.* **51**, 13957-13960 (2015).

37. Hu, Z., Tang, Y., Yu, B. Glycosylation with 3,5-Dimethyl-4-(2'-phenylethynylphenyl)phenyl (EPP) Glycosides via a Dearomative Activation Mechanism. *J. Am. Chem. Soc.* **141**, 4806-4810 (2019).

38. Liu, H., Liao, J., Hu, Y., Tu, Y., Sun, J. A Highly Efficient Approach To Construct (epi)-Podophyllotoxin-4-O-glycosidic Linkages as well as Its Application in Concise Syntheses of Etoposide and Teniposide. *Org. Lett.* **18**, 1294-1297 (2016).

39. Singh, Y., Demchenko, A. Koenigs-Knorr Glycosylation Reaction Catalyzed by Trimethylsilyl Trifluoromethanesulfonate. *Chem. Eur. J.* **25**, 1461-1465 (2019).

40. Neralkar, M. et al. Nucleofuge Generating Glycosidations by the Remote Activation of Hydroxybenzotriazolyl Glycosides. *J. Org. Chem.* **82**, 11494-11504 (2017).

41. Lian, G., Gao, Q., Lin, F. Synthesis of fructofuranosides: efficient glycosylation with N-phenyltrifluoroacetimidate as the leaving group. *Carbohydr. Res.* **343**, 2992-2996 (2008).

42. Shaw, M., Thakur, R., Kumar, A. Gold(III)-Catalyzed Glycosylation using Phenylpropiolate Glycosides: Phenylpropiolic Acid, An Easily Separable and Reusable Leaving Group. *J. Org. Chem.* **84**, 589-605 (2019).

43. Liao, J., Sun, J., Yu, B. Effective synthesis of nucleosides with glycosyl trifluoroacetimidates as donors. *Tetrahedron Letters* **49**, 5036-5038 (2008).

44. Sniady, A., Bedore, M. W., Jamison, T. F. One-Flow, Multistep Synthesis of Nucleosides by Brønsted Acid-Catalyzed Glycosylation. *Angew. Chem. Int. Ed.* **50**, 2155-2158 (2011).

45. Liu, G., Zhang, X., Xing, G. A General Method for *N*-Glycosylation of Nucleobases Promoted by (p-Tol)$_2$SO/Tf$_2$O with Thioglycoside as Donor. *Chem. Commun.* **46**, 12803-12806 (2016).

46. Lay, L., Cancogni, D. Exploring Glycosylation Reactions under Continuous-Flow Conditions. *Synlett* **25**, 2873-2878 (2014).

47. Yu, F. et al. Phenanthroline-Catalyzed Stereoretentive Glycosylations. *Angew. Chem. Int. Ed. Engl.* **58**, 6957-6961 (2019).

48. Peng, P., Schmidt, R. Acid-Base Catalysis in Glycosidations: A Nature Derived Alternative to the Generally Employed Methodology. *Acc. Chem. Res.* **50**, 1171-1183 (2017).

49. France, R. et al. Selective electrochemical glycosylation by reactivity tuning. *Org. Bio. Chem.* **2**, 2195-2202 (2004).

50. Wang, Y. et al. Synthesis of a chlorogenin glycoside library using an orthogonal protecting group strategy. *Carbohyd. Res.* **375**, 118-135 (2013).

51. Kotammagari, T., Gonnade, R., Bhattacharya, A. Biomimetic Total Synthesis of Angiopterlactone B and Other Potential Natural Products. *Org. Lett.* **19**, 3564-3567 (2017).

52. Liu, B., Zhang, F., Zhang, Y., Liu, G. A new approach for the synthesis of O-glycopeptides through a combination of solid-phase glycosylation and fluorous tagging chemistry (SHGPFT). *Org. Biomol. Chem.* **12**, 1892-1896 (2014).
